# Multi-Locus Next-Generation Sequence Typing of DNA Extracted From Pooled Colonies Detects Multiple Unrelated *Candida albicans* Strains in a Significant Proportion of Patient Samples

**DOI:** 10.3389/fmicb.2018.01179

**Published:** 2018-06-05

**Authors:** Ningxin Zhang, David Wheeler, Mauro Truglio, Cristina Lazzarini, Jenine Upritchard, Wendy McKinney, Karen Rogers, Anna Prigitano, Anna M. Tortorano, Richard D. Cannon, Roland S. Broadbent, Sally Roberts, Jan Schmid

**Affiliations:** ^1^Institute of Fundamental Sciences, Massey University, Palmerston North, New Zealand; ^2^Nextgen Bioinformatic Services, Palmerston North, New Zealand; ^3^Department of Biomedical Sciences for Health, Università degli Studi di Milano, Milan, Italy; ^4^Sir John Walsh Research Institute, University of Otago, Dunedin, New Zealand; ^5^LabPlus, Auckland District Health Board, Auckland, New Zealand; ^6^Department of Women’s and Children’s Health, University of Otago, Dunedin, New Zealand

**Keywords:** *Candida albicans*, next generation-multi locus sequence typing (NGS-MLST), detection of multiple strains in patient samples, candidemia, endogenous sources of infection, transmission, commensal colonization

## Abstract

The yeast *Candida albicans* is an important opportunistic human pathogen. For *C. albicans* strain typing or drug susceptibility testing, a single colony recovered from a patient sample is normally used. This is insufficient when multiple strains are present at the site sampled. How often this is the case is unclear. Previous studies, confined to oral, vaginal and vulvar samples, have yielded conflicting results and have assessed too small a number of colonies per sample to reliably detect the presence of multiple strains. We developed a next-generation sequencing (NGS) modification of the highly discriminatory *C. albicans* MLST (multilocus sequence typing) method, 100+1 NGS-MLST, for detection and typing of multiple strains in clinical samples. In 100+1 NGS-MLST, DNA is extracted from a pool of colonies from a patient sample and also from one of the colonies. MLST amplicons from both DNA preparations are analyzed by high-throughput sequencing. Using base call frequencies, our bespoke DALMATIONS software determines the MLST type of the single colony. If base call frequency differences between pool and single colony indicate the presence of an additional strain, the differences are used to computationally infer the second MLST type without the need for MLST of additional individual colonies. In mixes of previously typed pairs of strains, 100+1 NGS-MLST reliably detected a second strain. Inferred MLST types of second strains were always more similar to their real MLST types than to those of any of 59 other isolates (22 of 31 inferred types were identical to the real type). Using 100+1 NGS-MLST we found that 7/60 human samples, including three superficial candidiasis samples, contained two unrelated strains. In addition, at least one sample contained two highly similar variants of the same strain. The probability of samples containing unrelated strains appears to differ considerably between body sites. Our findings indicate the need for wider surveys to determine if, for some types of samples, routine testing for the presence of multiple strains is warranted. 100+1 NGS-MLST is effective for this purpose.

## Introduction

The yeast *Candida albicans* is a frequent commensal colonizer of humans, but can also cause disease, including life-threatening candidemia when it reaches the bloodstream in immunocompromised patients (Kim and Sudbery, 2011). Disease-causing isolates can originate from the patients’ colonizing strains (Reagan et al., 1990; Brillowska-Dabrowska et al., 2010) or be acquired from exogenous sources (Schmid et al., 1995).

Tracking sources of infection, or outbreaks, and decisions on antifungal therapy can necessitate typing and antifungal susceptibility testing of *C. albicans* recovered from patient samples. Commonly only one isolate per patient sample is assessed, derived from one of many *C. albicans* colonies recovered from the sample (White et al., 2002; McManus et al., 2012; Moorhouse et al., 2016). Even though the human body can be colonized by a variety of strains (Soll et al., 1991) this seemed justifiable, because the existing literature suggested that presence of multiple strains at the same site is unusual; when multiple strains were found, they were usually highly similar variants of one strain. Three studies, involving a total of 24 oral, vulvar and vaginal samples, found no evidence of unrelated strains in the same sample (Hellstein et al., 1993; Le Guennec et al., 1995; Lockhart et al., 1995). Jacobsen et al. (2008) found unrelated strains in only 2/32 samples (both oral). More recently, however, Choo et al. (2017) reported a considerably higher incidence (23%) of samples with multiple unrelated strains from dentures and saliva of denture wearers. A possible explanation for the differences between the latter and the former studies could be that multiple strains are present in some human samples more frequently than in others. If so it may be misleading to draw wider conclusions from the limited range of sample types analyzed to date. Another limitation of the existing data is that for the vast majority of samples fewer than 10 colonies were typed. As a result, strains present at frequencies of 10–20% would often have been missed.

The existing literature does thus not allow a reliable estimate of how often multiple strains are present in human samples. Analyzing only one colony per sample, clinical decisions could, not infrequently, be based on incomplete information.

We therefore developed a next-generation sequencing (NGS) modification of the existing highly discriminating *C. albicans* multilocus sequence typing (MLST) method (Bougnoux et al., 2003) for detection and simultaneous typing of multiple *C. albicans* strains in patient samples. We then used the method to estimate how often multiple strains coexist in the same site in the human body.

## Materials and Methods

### Isolation and Maintenance of Strains

Strains in this study included 60 previously characterized strains (Supplementary Table S1), chosen (Holland and Schmid, 2005) to best represent the genotypes of an international collection of 461 DNA fingerprinted commensal and disease-causing isolates, each derived from a single colony (Schmid et al., 1990, 1992, 1993, 1995, 1999; Soll et al., 1991); all of these strains had only been briefly cultured after their isolation and had subsequently been maintained as glycerol stocks at -80°C. Additional samples were collected from New Zealand patients diagnosed with candidemia (Supplementary Table S2), New Zealand mothers and their infants (Supplementary Table S3) and patients at an Italian hospital (Supplementary Table S4). Up to 100 *C. albicans* colonies, identified on CHROMagar^TM^ *Candida* (CHROMagar, France) were combined in glycerol stocks (Ausubel et al., 2014) which were then stored at -80°C. Ethical approval for collecting these samples had been obtained from the New Zealand Multi-regional Ethics Committee, application number MEC/12/06/059 and from the Ethics Committee of the Università degli Studi di Milano, application number 32/11.

### Generation of Strain Mixtures

To generate DNA samples from combinations of two known strains, aliquots of glycerol stocks of each strain were plated on YPD medium (Ausubel et al., 2014) and single colonies from each strain were picked with sterile toothpicks. Colonies from the strains were combined in different ratios (90 + 10, 80 + 20, and 50 + 50 colonies), in 1 ml of water. Cells were sedimented by a 5 min spin in a micro-centrifuge at 6000 × *g* and freeze-dried.

### DNA Extraction

Genomic DNA was extracted using a Qiagen DNeasy Plant Mini kit (Qiagen, Bio-Strategy Ltd., Auckland, New Zealand) following the manufacturer’s instructions. To obtain material for DNA extraction from single colony isolates and from single colonies from a patient sample, one colony was used to generate, after incubation at 30°C for 24 h, a patch on YPD agar (Ausubel et al., 2014). Patches were scraped off and ground in liquid nitrogen, using a pestle and mortar. To obtain *Candida* cells for DNA extraction from multiple-colony patient samples, an aliquot of glycerol stock was plated on YPD medium to create a lawn, which was then scraped off and ground in liquid nitrogen, using a pestle and mortar. As starting material for DNA extraction from artificial mixes of *C. albicans* strains 300 mg of freeze-dried cells was ground in liquid nitrogen.

### PCR Amplification of MLST Amplicons and Sequencing

For each sample seven MLST amplicons were amplified, using the primers shown in Supplementary Table S5, in a final volume of 25 μl containing 1 U *Taq* DNA polymerase (Qiagen), 5 μl of Q-buffer and 2.5 μl of 10x PCR buffer supplied by the manufacturer (Qiagen), 12 pmol of each primer, 250 μM of each dNTP (Roche Diagnostics, Auckland, New Zealand), and 10 ng DNA. The cycling conditions included an initial incubation for 2 min at 94°C, followed by 30 cycles of 45 s at 94°C, 30 s at 55°C, and 30 s at 72°C followed by a final 7 min extension step at 72°C. Reactions were carried out in an Eppendorf Mastercycler thermocycler (Eppendorf, Hamburg, Germany). Five microliters of each PCR reaction was loaded on a 2% agarose TAE gel to confirm successful amplification (Ausubel et al., 2014). The seven amplicons from each samples were pooled (4 μl of each PCR reaction) and the pools were submitted to New Zealand Genomics Limited (Dunedin, New Zealand) for a second round of PCR in which sample-specific primers were used to attach sample-specific barcodes (Nextera XT DNA Library Preparation kit, Illumina, San Diego, CA, United States) to the products. Sequencing was carried out an Illumina MiSeq instrument (≥ 2500 reads for each MLST amplicon in a sample).

### Extraction of MLST Sequence Information

Sequences were de-multiplexed using both sample-specific barcode and MLST-locus-specific sequence. Sequences were processed further using the software DALMATIONS, developed for this work. DALMATIONS performs the following tasks: For every sample, reads corresponding to each MLST amplicon are aligned using an allele sequence of laboratory strain SC5314 (Skrzypek et al., 2006). A quality-filtering step removes primer sequences as well as any reads that contain indels. The aligned reads are then used to calculate base call frequencies at each position of each amplicon. For DNA from a single colony, base calls are used to determine the MLST genotype. In doing so, if ≥95% of reads have the same base at a given position it is considered homozygous; otherwise it is considered heterozygous. For amplicons prepared from DNA derived from pools of multiple colonies, the MLST type information is extracted as follows. Using the previously determined MLST type of the single colony (sc strain), DALMATION simulates the base calls associated with a second strain (strain B) present in the pool, assuming percentages of strain B between 1 and 99%, in 1% steps. At each iteration it calculates, for each amplicon position, the base calls associated with the sc strain MLST type, then uses the residual base calls to assign a genotype to the inferred second strain, so as to minimize the differences between observed base calls and expected base calls, based on the genotype.

The software then identifies the most probable frequency and genotype of the second strain. It determines which inferred second strain frequency and genotype results in the lowest number of instances, across all positions in the seven amplicons, in which the difference between predicted and actual base calls is <5%. If there are several iterations that are equal in this respect, it finds the iteration leading to the smallest sum of all differences between expected and observed base calls, across all amplicon positions. In these calculations only informative positions are considered, i.e., sites at which >5% of the single colony and/or the pool base calls indicate the presence of a base different from that present in the SC5314 reference sequence. When attempting to infer the genotype of a second strain DALMATIONS is not restricted to assuming that the second strain is diploid. If, assuming diploidy, no second strain genotype can be found that reduces the difference between observed base calls expected base calls to ≤5%, DALMATIONS will explore if a better fit can be reached by assuming triploidy or tetraploidy at this position.

When the sequence data for the single colony are inconsistent with diploidy at a given position, the user is alerted and this position is not used for automatic inference of the second strain’s MLST type and frequency. A spreadsheet template (tri9.xsl) is provided with the software to assist the user to infer which, if any, ploidy levels > 2 would best explain the data. After inputting call frequencies and the frequency of the strains inferred from other loci, the spreadsheet calculates the differences between observed frequencies and those expected if one or both strains are aneuploids, with ploidy levels up to 4n at this locus.

Amplicon sequences are concatenated into one single fasta-formatted file. For indirectly inferred strains, the fasta header includes the percentage of colonies the strain is likely to represent and an unexplained data indicator (UDI) score. DALMATIONS calculates the latter by multiplying the highest percentage of base calls not explained at any position with the average percentage not explained, across all positions. The better the genotype and percentage of the inferred strain explain the observed base calls, the lower the UDI score. Manually edited positions (see above) are not considered in the automatically calculated UDI scores (in this case the user must calculate the UDI and edit the fasta heading accordingly). See Supplementary Data Sheet S1 for all MLST types we determined).

DALMATIONS is an open source software and the code can be found at github^1^.

The software Geneious, (Biomatters, Auckland, New Zealand) was used to calculate the number of differences between pairs of concatenated sequences and distance matrices. From these UPGMA dendograms (Sokal and Michener, 1958) were generated using the software R (R Foundation, Vienna, Austria).

## Results

### Underlying Principles of 100+1 NGS-MLST

Our aim was to develop a methodology that could detect the presence of multiple strains in a patient sample and determine their MLST types. We wanted to do so using base call frequencies in the thousands of sequence reads per amplicon generated by NGS (MiSeq; Illumina_Inc, 2017) sequencing of the seven established *C. albicans* MLST loci [*AAT1a, ACC1, ADP1, MPIb, SYA1, VPS13, ZWF1b* (Bougnoux et al., 2003)] in DNA extracted from pools of colonies recovered from a patient sample.

We hypothesized that it should be possible to detect multiple strains by sequencing amplicons amplified from DNA extracted from a pool of colonies and comparing their sequences to those derived from DNA from one of the colonies. If multiple strains are present in the sample, the pool should contain different strain-specific versions of each amplicon, and this should be reflected in base call frequencies. For example, if a sample contained two strains, one of them homozygous [*C. albicans* is diploid (Kim and Sudbery, 2011)] for C in position 279 of the *ACC1* amplicon, the other homozygous for T, with the second strain accounting for 30% of the colonies recovered from the sample, then in the pool sequences ∼70% of the base call should be C and ∼30% should be T. In contrast, base calls for a single colony should be ∼100% C or ∼100% T (depending on which of the two strains the single colony represents). If all colonies in the pool represent one strain, then base call frequencies for pool and single colony should be virtually identical.

If such comparisons indicated the presence of multiple strains, the MLST types of the additional strains could be obtained directly by MLST typing of individual colonies. However, colonies of different strains often have a similar morphology and different colonies of the same strain can have different morphology (Hunter, 1991). Thus, a large number of individual colonies would need to be MLST typed, which would be labor-intensive and costly. However, if, in addition to the strain represented by the single colony (sc strain) only one other strain (strain B) were present in the sample, it should be possible to infer strain B’s MLST type and its frequency from the pooled and single colony sequences by an iterative computational approach. This would involve assuming different frequencies of the second strain, and at each frequency testing if a second strain MLST type can be found that, if the strain were present at this frequency, would explain the sequencing data.

**FIGURE 1:**
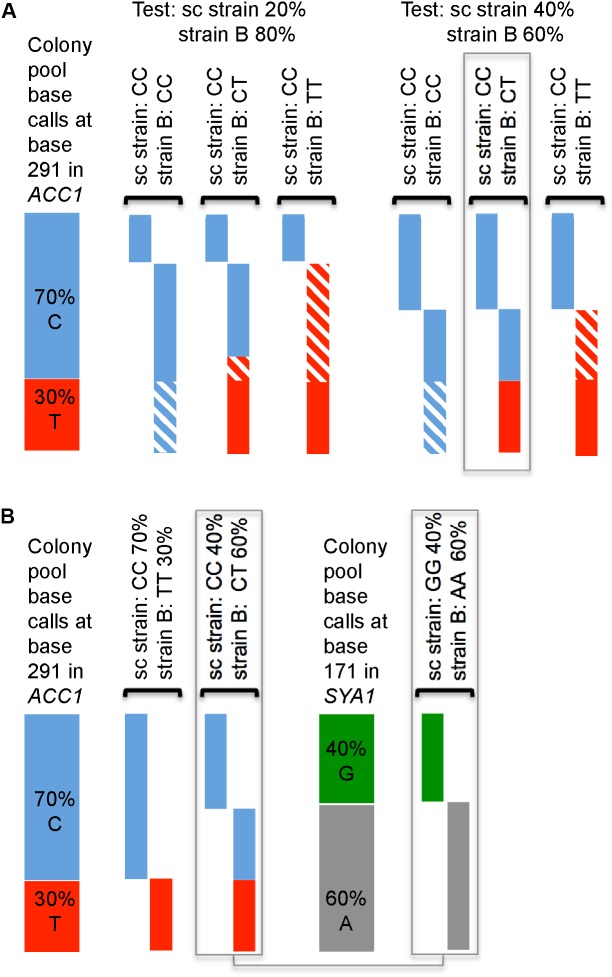
Principle of deducing the frequency and genotypes of two strains from base calls when sequencing DNA prepared from a mixture of colonies. **(A)** Predicted allele frequencies assuming that either 20 or 40% of cells in the sample were from the strain represented by the directly sequenced colony (sc strain; CC, i.e., C-homozygous) and assuming three possible genotypes of strain B (shown on top, above brackets), which are compared to observed base calls (at position 291 in the *ACC1* amplicon; 70% C/30% T; shown on the left). Contributions of each strain to expected base calls are visualized as boxes under the genotypes; striped parts of the bars indicate the differences between expected and observed frequencies. **(B)** Two possible frequencies of strain B would explain the pool base calls at *ACC1* 291, but only one frequency of strain B (60%) explains the base call frequencies at position 171 in the *SYA1* amplicon.

The process is illustrated in **Figure 1**. In the example shown, sequencing of the single colony indicates that one of the strains (the sc strain) is homozygous for C at one given base in one MLST amplicon (*ACC1* position 291). Of the pooled colony DNA sequencing reads, 70% indicate a C in this position, 30% a T. **Figure 1A** shows the outcome of assessing how well two assumed frequencies (80 and 60%) of the unknown strain (strain B), would fit the data. The first (80% frequency of strain B) is a very poor fit. In contrast, assuming a frequency of the unknown strain B of 60% explains the observed base calls perfectly, under the assumption that strain B is heterozygous (CT) at this position.

For position 291 in *ACC1* there exists a second explanation that matches the data, namely that strain B accounts for 30% and is T-homozygous (**Figure 1B**). Which alternative is correct can be determined because all polymorphic sites in all amplicons are considered. For position 171 in the *SYA1* amplicon, only a strain B frequency of 60% can explain the data (**Figure 1B**).

### Development of the 100+1 NGS-MLST Method

We wanted to use the MiSeq platform and combine large numbers of patient samples in a sequencing run, necessitating the inclusion of a sample-specific DNA barcode (Illumina_Inc, 2016) in each amplicon. We expected that only a minority of MiSeq reads would yield >250 bp of good-quality sequence. After subtracting the combined length of sample barcode and locus-specific primer sequence, this would be equivalent to ≥190 bp of informative sequence.

The informative amplicon sequences analyzed in conventional MLST are 373–491 bp, and thus too long for polymorphisms across each MLST locus to be well represented with the anticipated MiSeq read lengths. We therefore designed new primers (Supplementary Table S5) to generate amplicons with 343–375 bp informative sequence – a total of 2494 bp across all amplicons, compared to 2883 bp in conventional MLST (Bougnoux et al., 2003), and containing 85% of the polymorphic sites in the seven amplicons used in conventional MLST (as assessed by counting polymorphic sites in allele sequences in the *C. albicans* MLST data base at https://pubmlst.org/calbicans/).

We initially tested the performance of our methodology on single strains, some of which had been typed previously by conventional MLST, to assess (i) the impact of reduced amplicon lengths and of MiSeq sequencing on discriminatory power, (ii) whether our methodology gave results consistent with conventional MLST and (iii) how closely base call frequencies matched theoretical expectations. The latter would be crucial for detecting heterozygosity, as well as for detecting additional strains and for inferring their genotypes.

We sequenced the MLST amplicons of 54 unrelated strains from a set (Supplementary Table S1), chosen to best represent the genotypes of an international collection of 461 DNA fingerprinted commensal and disease-causing isolates. The discriminating power of our method was 0.9944 (in 8/1431 pairwise comparisons two unrelated isolates had the same MLST type; Supplementary Figure S1 and Supplementary Data Sheet S2), lower than that reported for the original method [0.9996 (Tavanti et al., 2005)]. We note that failure to discriminate between two isolates with our method only occurred if the isolates belonged to a group of genetically very similar strains [general purpose-genotype cluster (Schmid et al., 1999), corresponding to clade 1 (Tavanti et al., 2005) Supplementary Table S1 and Supplementary Data Sheet S2]. These difficult-to-distinguish strains comprised 64% of our strain set, but only 37% of the set used by Tavanti et al. (2005).

Twenty strains in our set had been previously typed by conventional MLST by Tavanti et al. (2005). Our MLST types matched theirs with the exception of 18 heterozygous SNPs. For all of these Sanger sequencing confirmed our results to be correct.

Base call frequencies closely matched theoretical expectations. We assessed this for all positions in all amplicons in 20 strains (49594 homozygous and 286 heterozygous sites). At homozygous sites, on average 99.9% of reads indicated the presence of the same nucleotide. The lowest frequency, observed for only one homozygous site, was 95%, and only 189 sites (0.4%) had frequencies of the predominant nucleotide of ≤97%. For heterozygous sites, the average difference between the number of calls expected for one of the two nucleotides present (50%) and calls observed was 1%. The maximum, observed once, was 3.5% (i.e., 46.5% of calls were for one base, 53.5% for the other) and only for 5/286 (1.7%) sites was the difference ≥ 3%.

Two heterozygous sites in one strain were excluded from the analysis as a 1:3 ratio of bases was observed, reproducibly, at these sites in the *ZWF1b* MLST amplicon. Aneuploidy is not uncommon in *C. albicans* (Selmecki et al., 2010) and these ratios indicate that this particular strain has 3 copies of this locus.

The close match of base calls with theoretical expectations suggested an avenue for lowering the cost and time requirements of the methodology when is it is not crucial to obtain complete results immediately, for instance in retrospective studies. Under such circumstances, rather than immediately typing both a colony pool and a single colony, initially only DNA prepared from a colony pool needs to be sequenced. Base call frequencies at all amplicon positions of either > 95% or 50 ± 5% would indicate that the pool represents only a single strain, and that its MLST type has been accurately captured. Base call frequencies outside these ranges indicate presence of a second strain and the need to sequence DNA from a single colony.

We developed software, DALMATIONS, that implemented the iterative approach for finding a second strain’s frequency and genotype outlined above. Because of stochastic variations, base call frequencies will not exactly align with theoretical expectations, and as a result DALMATIONS cannot infer a strain B frequency and genotype that perfectly matches the sequencing data. Rather, it determines which inferred strain B frequency and genotype results in the lowest number of instances in which the difference between predicted and actual base calls is <5%. If there are several iterations that are equal in this respect, it finds the iteration leading to the smallest sum of all differences between expectation and observed base calls, across all sites in all amplicons.

Circumstances might occur, such as sequencing errors or the presence of >2 strains in the sample, under which the inferred strain B genotype will be poorly predicted. When reporting the strain B genotype and its frequency that best matches the sequencing data, DALMATIONS therefore also provides an indication of mismatches between inferred genotype and frequency of strain B and the actual sequencing data. It records the average percentage of base calls not explained by the reported strain B genotype and frequency across all informative amplicon positions (i.e., positions used to infer strain B genotype) and the highest percentage of base calls at any one position not explained. It then multiplies the two values and reports the product as a UDI (unexplained data indicator) score. For example if, averaged across all positions, 3% of base calls are not explained, and the highest percentage unexplained at any one position is 5%, then the UDI is 15 (3 × 5).

### Accuracy of Inferring, From Base Calls, MLST Types Present in DNA Pools Extracted From Artificial Mixtures of Colonies of Two Strains

We next tested how accurately our 100+1 NGS-MLST method would infer the frequency and genotype of a second strain in a sample containing two strains. We did this using artificial mixtures of pairs of known strains, prepared either *in silico* by preparing sequencing read files containing different proportions of reads obtained from the sequencing of single colonies of two strains, or by mixing colonies from the strains and extracting DNA from these mixtures of cells.

**FIGURE 2:**
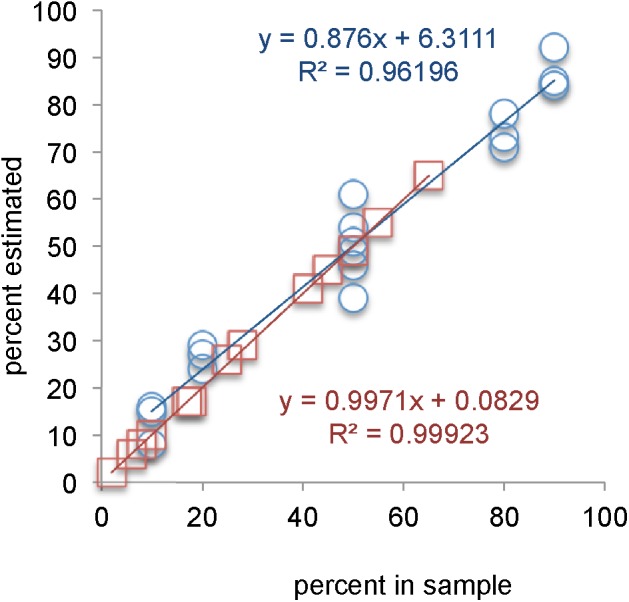
Frequencies of strains deduced from mixed samples versus percentage of reads in *in silico* artificial pairs (red line of best fit, squares) or versus the percentage of colonies used to prepare DNA from a mixture of two strains (blue lines of best fit, circles).

**FIGURE 3:**
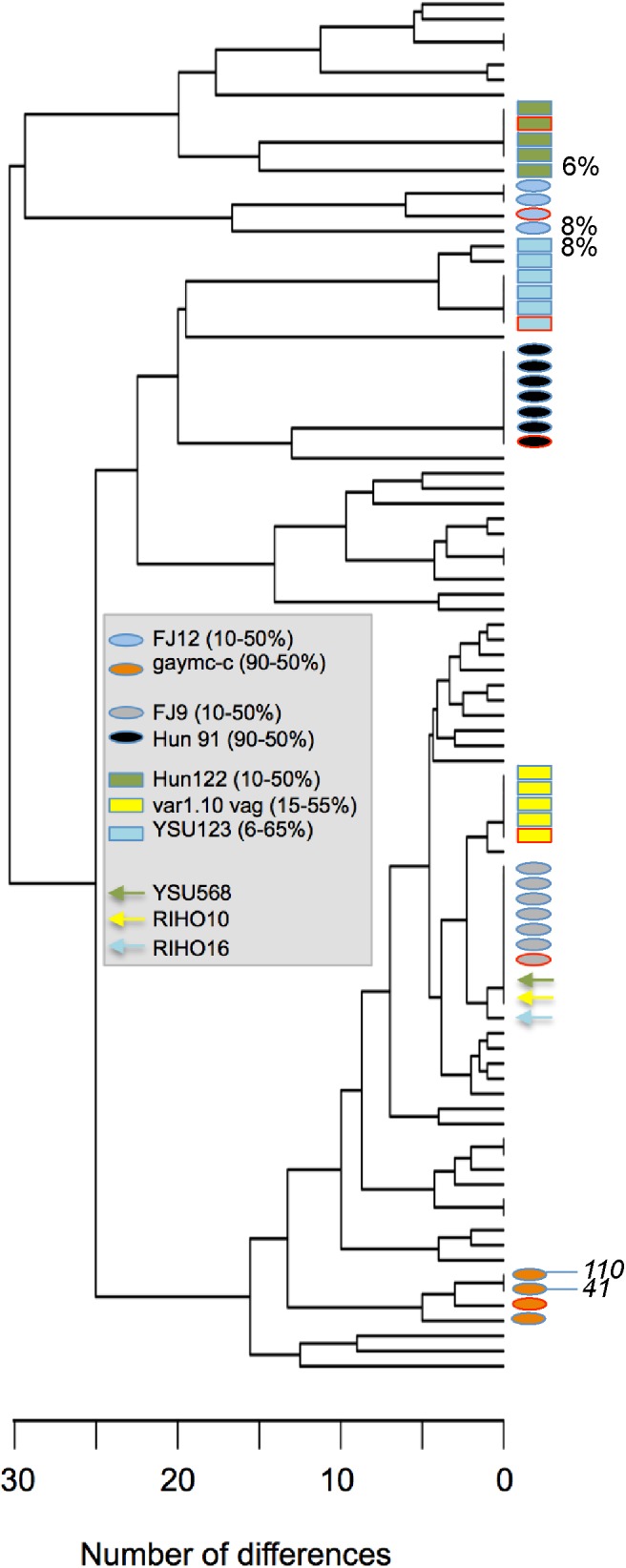
Similarity between genotypes inferred in artificial mixtures of pairs of strains and their real genotypes. The UPGMA dendrogram is based on the number of nucleotide differences between 60 isolates (listed in Supplementary Table S1) directly determined by sequencing, plus attempts to infer the genotypes of seven of these strains indirectly (each marked by a different colored symbol; the strains and their frequencies are shown in the gray box). Blue-bordered rectangles mark MLST types of strains inferred from mixtures generated *in silico* by combining reads from the sequencing of one single colony of each of two strains (the arrows indicate the single colony strain in the mixture used to infer the genotype of the other strain from the mixed sequence). Ovals indicate strains that were inferred from DNA preparations generated from a mixture of colonies of two strains. Red-bordered symbols mark the true genotype of the strain we attempted to infer. Percentages shown next to symbols on the right are the inferred percentages of the inferred strain when less than 10% of sequences were derived from the inferred strain. Number in italics are UDI scores (only scores exceeding 25 are shown).

In all 13 attempts conducted with *in silico* mixtures, with simulated frequencies of the second strain ranging from 6 to 65%, the DALMATIONS software reported the presence of a second strain at a frequency differing by no more than 1% from the frequency of reads representing the second strain in the mixture (**Figure 2**). In only three attempts were the inferred genotypes not identical to the real genotype (**Figure 3**). Of the latter, two were for strains simulated to be present at very low levels (6 and 8%). All inferred genotypes were closer to the real genotypes than to those of the 59 other strains from our global collection.

In 18 attempts to deduce the frequency of a second strain in DNA pools prepared from 10 + 90, 20 + 80, and 50 + 50 colonies of pairs of strains, the DALMATIONS software again inferred the numbers of colonies of the strains accurately. The inferred numbers never differed from the actual number of colonies of the second strain by more than 11 (average difference 5.3 ± 3.2 colonies; **Figure 2**). Twelve of 18 inferred second strain genotypes were an exact match to the actual genotype of the second strain (**Figure 3**). One instance in which a genotype was not accurately inferred was in a sample in which the concentration of the strain’s DNA was very low (8%), making inferences of its genotype difficult. In two other cases a high UDI (>15) would have alerted the user that the inferred genotype may not be reliable (**Figure 3**). Only these two UDI scores exceeded 15. Of the remaining 18 UDI scores most (13 scores) were below 10.

Excluding the two attempts to infer MLST types of strains present at a frequency < 10%, the differences between the inferred genotypes and the real genotypes were 3, 6, or 7 base calls, a small number of differences compared to the typical number of differences between unrelated strains: Differences between MLST types of pairs of unrelated strains of ≤3, ≤6, and ≤7 bases occurred with frequencies of 6, 17, and 19% respectively (Supplementary Figure S1). More importantly, all inferred genotypes of second strains inferred from DNA prepared from colony mixes were more similar to their real genotypes than to those of 59 other strains included in the analysis.

These experiments indicated that 100+1 NGS-MLST could accurately infer the frequency of a second strain in a sample and provide a good approximation of its genotype.

### 100+1 NGS-MLST Detects Multiple Unrelated Strains in 7/60 Human Samples

**FIGURE 4:**
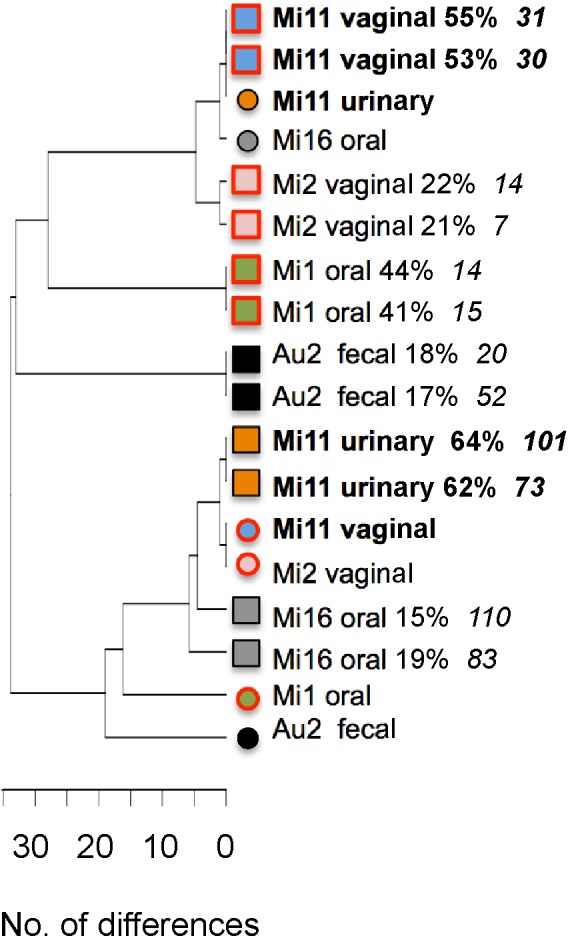
UPGMA tree showing differences between MLST types, directly and indirectly inferred, in six samples containing two unrelated *C. albicans* strains. Different samples are distinguished by different colored symbols. Circles mark the MLST type of the sc strain, i.e., the strain directly determined by sequencing a single colony. Squares indicate the outcome of two attempts to indirectly infer the genotype and frequency of the second strain in the sample. Labels include patient number and site sampled. For indirectly inferred MLST types this is followed by the estimated percentage of the strain in the sample and the UDI of the estimate. Strains from candidiasis samples are marked by red borders. MLST types in two samples from patient Mi11, both containing the same pair of strains, are highlighted by bold lettering.

**FIGURE 5:**
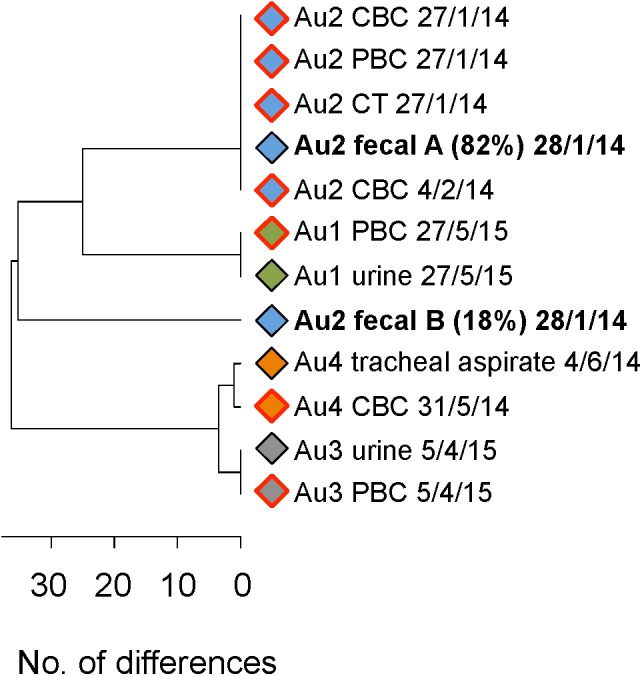
UPGMA tree showing MLST types of isolates from four candidemia patients. Isolates from the same patient are marked by rhombs of the same color. A red border indicates that the isolate is from a sterile site (PBC, peripheral blood culture; CBC, catheter blood culture; CT, catheter tip). Labels include patient number, sampling site and date of isolation. Labels of two strains in the patient Au2 fecal sample are in bold type and include the estimated percentages of the two strains.

**FIGURE 6:**
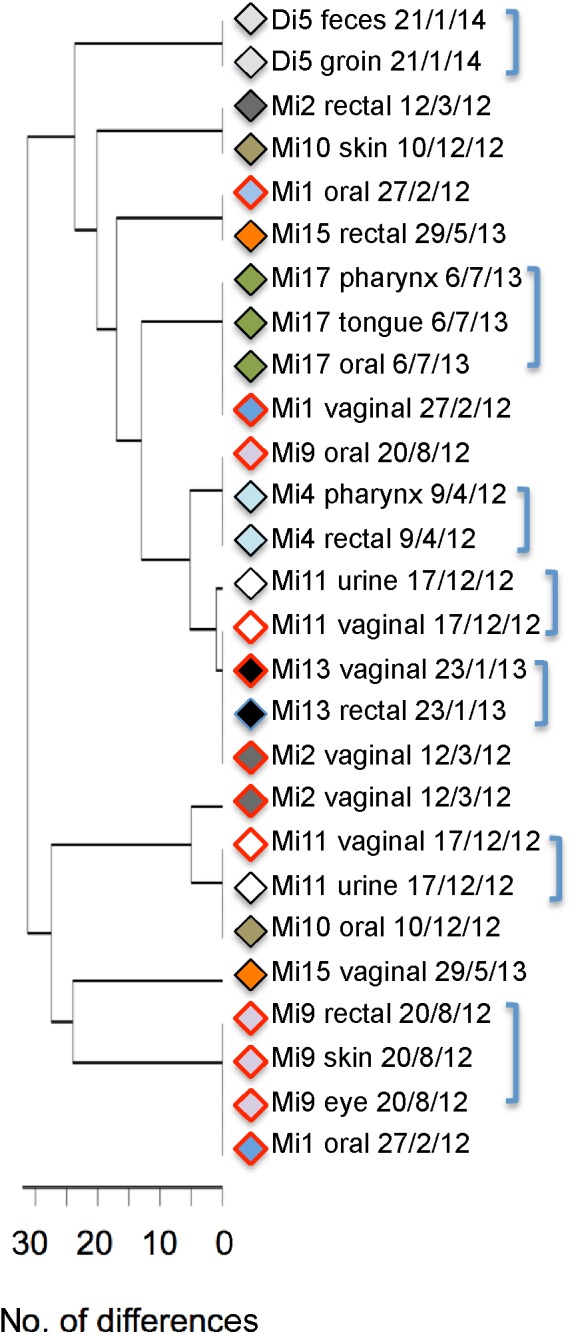
UPGMA tree showing MLST types of isolates from 10 patients who were sampled at different sites on the same day. Isolates from the same patient are marked by a symbol of the same color. Strains from candidiasis samples are marked by red borders. Labels include patient number, sampling site and date of isolation. Brackets mark instances in which multiple isolates from the same patient have the same (or extremely similar) MLST types.

**FIGURE 7:**
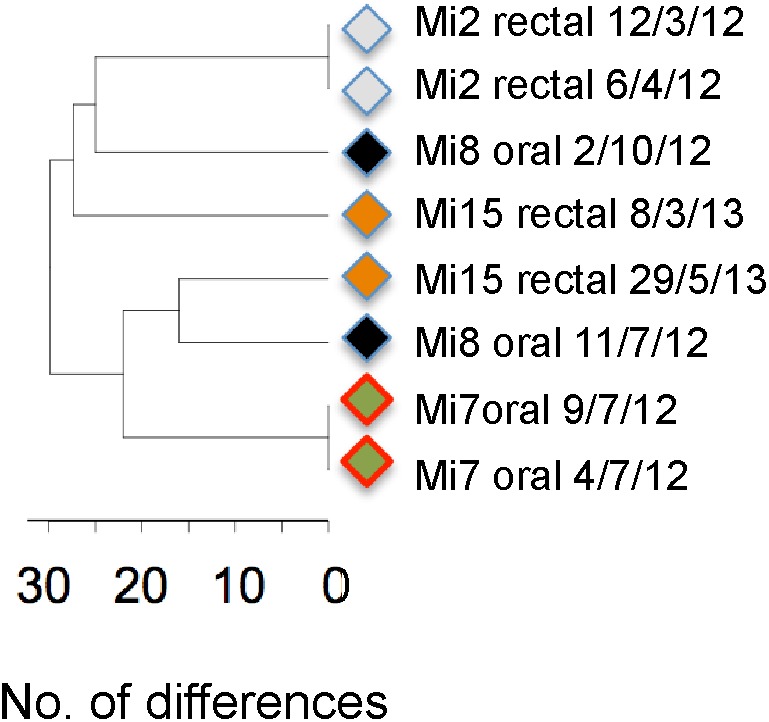
UPGMA tree based on MLST types of four patients for whom the same site was sampled on two dates. Strains from candidiasis samples are marked by red borders. Labels include patient number, sampling site and date of isolation.

To further test the methodology and to get a better estimate of how often human samples contain multiple strains, we used it on a selection of 60 human samples (**Figures 4–7** and Supplementary Tables S2–S4). Eleven samples were derived from four patients diagnosed with candidemia in Auckland, New Zealand, another 11 from the oral cavities of 9 healthy women about to give birth plus two from one of their infants in Dunedin, New Zealand. The remaining 38 were from non-sterile sites from 17 individuals at a hospital in Milan, Italy, including samples from sites that were merely colonized and sites where *C. albicans* caused disease.

We initially sequenced pooled colony samples (most samples contained ≥ 100 *C. albicans* colonies; the lowest number was 41 colonies). We identified 28 samples in which base call frequencies of 94% – 56% or 6% – 44% suggested the presence of multiple strains. However, in 21 of these, only one of the seven MLST loci had base call frequencies in these ranges, suggesting that, if two strains were present, one of them would be a closely related variant of the other, having suffered a loss of heterozygosity (LOH) at one locus. We subsequently typed a single colony from 9 of these 21 samples. In 8 of these, single colony base calls indicated > 2n ploidy at the *ACC1* locus, consistent with presence of a single aneuploid strain. In the one remaining sample the single colony confirmed the presence of two related strains, differing by an LOH at *AAT1*. Thus 53/60 samples contained either a single strain or two variants of one strain, distinguished by an LOH.

For six of the seven remaining samples, with base call frequencies in the colony pool of 94% – 56% and 6% – 44% at multiple loci, we sequenced a single colony to infer the genotype and frequency of the two strains present. To test the robustness of our inferences we did so twice, based on two sequencing runs of DNA from the colony pool. Both attempts gave highly similar, usually identical, results in terms of inferred genotype of the second strain (**Figure 4**). With one exception (sample Mi16) the two inferred genotypes were more similar to each other than to any other strain shown in the figure. In all samples both attempts to estimate the frequencies of the two strains gave comparable results. Thus inferred genotypes and frequency estimates were reproducible. For two samples the reliability of inferred genotypes was supported by the presence of an identical, or almost identical directly sequenced strain in another sample from the same individual (Mi11, highlighted in bold in **Figure 4**).

UDI values for the inferred genotypes (**Figure 4**) were often higher than those for inferred genotypes in our artificial pairs (see above). This could have been an indication that more than two strains were present in these samples: If three strains are present in a pool then attempts to infer only one additional strain would yield a compromise genotype, poorly matching the data. However, when we investigated the fit of the data for individual positions in the amplicons, we found that the high UDIs were caused by base calls at only one or two positions, usually in *ACC1*, suggestive of LOH at one locus, amplification or sequencing errors or highly unusual ploidy (even assuming triploidy or tetraploidy at this locus did not result in a good match of predicted and expected base calls). At all other positions base call frequencies predicted on the basis of the inferred genotypes closely matched the observed base call frequencies. Thus if a third strain was present at all, it would have been merely an LOH variant of one of the two strains we identified. Thus each of the six samples essentially contained two strains.

Application of 100+1 NGS-MLST to our set of patient samples, while not a systematic survey, also provided some insights into the nature of co-existing strain combinations, the degree to which different sites may favor coexistence of multiple distinct strains and in what ratios (**Table 1** and **Figures 4–7**). Similarity of MLST type does not seem to be a prerequisite for coexistence: members of each of the six pairs were more similar to a member of another pair than to the second member of the pair (**Figure 4**). In terms of which sites favor co-colonization, we encountered multiple strains at four of the nine sites we sampled, with frequencies of the less frequent strain ranging from 18% to 47% (average 31% ± 13%; **Table 1**). Our data suggest that multiple strains are less common in rectal swabs and blood, but that ∼1/3 of samples from feces, urine and vagina might contain multiple strains. Of the seven samples with multiple strains, four were from colonized sites at which *C. albicans* did not cause disease symptoms (Au2 fecal, DM6 oral, Mi11 urinary; Mi16 oral). The remaining three were from sites where *C. albicans* caused disease (**Figure 4**), namely oral candidiasis (in Mi1) and vaginitis (in Mi2 and Mi11), indicating that candidiasis at non-sterile sites can be caused by combinations of strains. In Mi11 a urinary tract sample contained the same two strains as the vaginitis sample and in similar frequencies (**Figure 4**); this suggests that a given combination of strains may be successful in different niches.

**Table 1:** Frequency of coexistence in different sampling sites.

Site	Frequency of multiple strains
Blood	0/7
Eye	0/1
Feces	1/2
Oropharyngeal	3/25
Rectal	0/11
Respiratory	0/1
Skin	0/3
Urine	1/4
Vagina	2/6
All sites	7/60 (12%)

We also verified that gaining reliable information about sources of candidemia requires typing of more than one colony: In all four Auckland candidemia patients MLST types at non-sterile sites matched those of a single MLST type in the bloodstream (**Figure 5**), indicating colonizing isolates as the source of candidemia. However, in one patient (Au2) the colonizing source of the blood strain might have been missed if only one colony from a fecal sample containing two strains had been MLST-typed.

Lastly, our samples highlight that different strains are often present in different non-sterile sites of the same individual and that the *C. albicans* flora at a given site can change fairly quickly. Of 10 patients sampled at different non-sterile sites on the same day, five carried different strains at different sites (**Figure 6**) and strains at a given site were replaced by other strains within 3–5 months (**Figure 7**; Mi8 and Mi15; strains did not change over a shorter periods in Mi2 and Mi7).

## Discussion

Our results indicate that human samples do not infrequently contain multiple *C. albicans* strains. We found this to be the case in 7/60 i.e., 12% of samples. However this average may not be particularly meaningful. Our data indicate that some types of samples contain multiple strains much more often – one likely reason for the range of frequencies of such samples (0–25%) reported in the literature (Hellstein et al., 1993; Le Guennec et al., 1995; Lockhart et al., 1995; Jacobsen et al., 2008; Choo et al., 2017). When multiple strains were present in our samples, one of them often represented less than ¼ of the cells. This suggests that the low number of colonies sampled in previous work (Hellstein et al., 1993; Le Guennec et al., 1995; Lockhart et al., 1995; Jacobsen et al., 2008; Choo et al., 2017) would have often prevented detection of the presence of multiple strains.

These considerations suggest that wider surveys are needed to establish how often multiple strains are present in different types of samples and if in some situations routine testing for the presence of multiple strains could be warranted. The 100+1 NGS-MLST method is an effective and reliable approach for doing so. In addition, the method provides accurate estimates of the frequency of the strains and determines their MLST types – we have shown that when two strains are present a reasonably accurate MLST type can usually be inferred indirectly for the second strain based on the pooled colony sample. We note that the underlying approach of comparing a single colony and a pool can, in principle, also be used to detect the presence of multiple strains using non-sequencing based *C. albicans* typing methods such as microsatellites (Sampaio et al., 2005), although it will probably be harder to deduce the second strain’s genotype and frequency. Our approach should also have applications in MLST typing of other pathogens – *C. albicans* is not the only species in which multiple strains can coexist in the same host niche (Cookson et al., 2017).

None of our samples contained more than two unrelated strains, but in principle it is possible to infer the genotypes of three strains in a sample using our methodology. One output of DALMATIONS is a table showing discrepancies between actual base calls and base calls expected for strain B at each informative amplicon position (i.e., variable positions used to infer strain B genotype) at 98 possible strain B1 frequencies (1–99%). If strain B actually represents two strains (B1 and B2) then there should be one frequency (F1) at which, for positions at which only B1 differs from the sc strain, expectations and observed base calls are a close match. There should be another such frequency (F2) for B2-specific polymorphisms and a third (F3) for polymorphisms common to B1 and B2 and distinguishing them from the sc strain. Using these frequencies and the known genotype of the sc strain, it is possible to infer genotypes and frequencies of all three strains (a description of the procedure is included in Supplementary Data Sheet S3).

100+1 NGS-MLST provides more information than conventional MLST sequencing of one colony per sample, and at a 40% lower sequencing cost (60% less if single colonies are only typed if read distribution in a pool indicates the presence of multiple strains; based on quotes from our local sequencing facility). Additional savings arise from a reduction in the number PCR product cleanups undertaken because in our method this is done for a mixture of all 7 PCR products for DNA from each colony or colony pool rather than for each product individually, as required for Sanger sequencing. The only drawback of 100+1 NGS-MLST is the reduced amplicon lengths, inherently associated with a reduction in discriminatory power. However, this is largely compensated by better reproducibility of typing for two reasons. One is that use of base call frequencies by 100+1 NGS-MLST is likely to be more reliable than chromatogram peak height (Odds et al., 2006) in determining whether a locus is heterozygous. In addition, 100+1 NGS-MLST typing is less affected by changes in MLST type that can be caused by LOH. *C. albicans* frequently suffers LOH events both in the host (Bougnoux et al., 2006) and during laboratory culture of *C. albicans* ((Odds et al., 2006); this study, data not shown). If LOH occurs, a single colony typed by conventional MLST may represent the LOH-altered MLST type. In contrast 100+NGS-MLST will also detect the original genotype, if it is still present in part of the culture. Thus our methodology is more likely than conventional MLST to produce exact matches when the same strain is present in different samples. As a result, the reproducibility-adjusted discriminatory power (Hunter, 1991) of 100+1 NGS-MLST may rival or even exceed that of conventional MLST, at least as far as the MLST type of the directly sequenced colony is concerned.

Aside from its obvious advantages for assessing origins of candidemia or antifungal resistance profiles, capturing all strains in a sample by detecting them with 100+1 NGS-MLST has other benefits. It enables genomic comparisons that may identify features that determine the potential of a strain to cause candidemia in humans – an important opportunity given that such features cannot be reliably identified in animal models (Odds et al., 2001; Schmid et al., 2011). Comparing the genomes of those members of pairs of co-colonizing strains at non-sterile sites that become etiological agents of candidemia, with those that do not (such as the two fecal strains in candidemia patient AU2) may reveal such pathogenicity determinants. Also, we have recently obtained evidence that the transition of a colonizing strain into a pathogen could involve mutation of highly mutable tandem repeat-containing *C. albicans* open reading frames [TR-ORFS (Zhou et al., 2017)]. If so, we would expect an increase in the frequency of virulence-enhancing TR-ORF alleles during the transition from colonization to disease in otherwise genetically identical *C. albicans* cells at the site. 100+1 NGS-MLST would be ideal for determining whether such shifts in TR-ORF allele frequency during pathogenesis are caused by mutation of an existing strain or signal that the disease is caused by an incoming unrelated strain with different TR-ORF alleles.

Multiple strains were as common in samples from non-sterile sites at which *C. albicans* caused disease as in samples from sites that were merely colonized [one of the few previously described samples with two unrelated strains was also derived from an oral candidiasis sample (Jacobsen et al., 2008)]. Thus even though *C. albicans* strains apparently differ in their potential to cause human disease and mortality (Schmid et al., 1999; Odds et al., 2007; Schmid et al., 2011) competition during pathogenesis does not necessarily eliminate all but one strain. The same might apply to candidemia. However, presumably candidemia usually involves a bottleneck in that only a small number of cells initially enter the bloodstream. This would often eliminate one strain (Kimura and Ohta, 1969) and be one of the reasons why only one strain tends to be isolated from bloodstream samples (Odds et al., 2006); we observed the same in our small set of bloodstream samples.

Our survey adds to the evidence (Soll et al., 1991; Jacobsen et al., 2008; Choo et al., 2017) that the commensal *Candida* microbiome of humans is complex and dynamic, involving multiple changing strains, sometimes coexisting at the same site. Commensalism receives less attention than pathogenesis, but the primary functions of *C. albicans* virulence factors and the selective pressures that led to their evolution are most likely exclusively linked to its commensal lifestyle (Schmid et al., 1999) – and thus easiest to understand in this context. Using 100+1 NGS-MLST to investigate how strains compete with each other can thus yield important new insights into *C. albicans*’ pathogenicity not only when applied to sites of infection, but also when used on commensal samples.

## Ethics Statement

This study was carried out in accordance with the recommendations of the Ethical Guidelines for Observational Studies of the New Zealand Multi-regional Ethics Committee and the Ethics Committee of the Università degli Studi di Milano. The protocol was approved by the New Zealand Multi-regional Ethics Committee and the Ethics Committee of the Università degli Studi di Milano. All subjects gave written informed consent in accordance with the Declaration of Helsinki.

## Author Contributions

NZ and CL prepared DNA. MT and DW assembled reads into sequences. CL, JU, WMK, KR, AP, AT, and SR obtained, processed, and curated clinical samples. RC, RB, SR, and JS planned and supervised the collection of clinical samples. JS, DW, and NZ developed and tested the DALMATIONS software. DW wrote the software code. JS led the project and wrote the manuscript, assisted by input from all authors.

## Conflict of Interest Statement

The authors declare that the research was conducted in the absence of any commercial or financial relationships that could be construed as a potential conflict of interest.

## Footnotes

1 https://github.com/dwheelerau/dalmations/
