# Supplementary material for: Multi-Locus Next-Generation Sequence Typing of DNA Extracted From Pooled Colonies Detects Multiple Unrelated Candida albicans Strains in a Significant Proportion of Patient Samples
Source: Front Microbiol. 2018 Jun 5;9:1179. doi: 10.3389/fmicb.2018.01179 (PMC5996278; doi:10.3389/fmicb.2018.01179)
Supplement: Supplementary file 6 [file Data_Sheet_1.DOCX]

**Supplementary Data Sheet S1. MLST types determined in this study**

MLST types are listed in fasta format, with sequences of the seven loci concatenated as follows: Bases 1 - 348, *AAT1*; bases 349 - 709, *ACC1*; bases 710 - 1061, *ADP1*; bases 1062 – 1416, *MPI*; bases 1417 – 1758, *SYA1*; bases 1759 - 2132, *VPS13*; bases 2133 – 2494, *ZWF1*.

Indirectly inferred MLST types are marked with "IND", followed by the estimated percentage of cells of this strain in the sample, followed by the UDI score.

No MLST type is shown for Dm6. The sample from this individual contained two strains but no single colony was sequenced to infer their MLST types.

>Au1_blood_(peripheral_blood_culture)_25/05/2015

AATTATTGAAATGGAATGATATTCCATTGGCTCCACCAGACAAAATTTTGGGTATTTCTGAAGCTTATAACAAYGATTCTAACCCTCAAAAAATCAATTTGGGGGTTGGTGCTTATAGAGATAATTCCGGTAAACCAATTATTTTCCCATCAGTTAAAAAAGCTGAAGAAATTTTATTGGGTAAAGAAACTGAAAAGGAATATACTGCCATTGTTGGTTCCAAAAATTTCCAATCAATTGTGAAAAATTTCATTTTCAACAATTCTAATAAAGATGCCAATGGTAAACAATTAATTGATGATGGTAGAATTGTTACTGCTCAAACCATCTCTGGTACTGGATCACTTAAACCTCTTATGGTGAAATTTTTGCTAAACATAGAGAACCAAATTTGGAAATTATTCGTGAGGTTGTTGATTCCAAACATATTGTTTTTGATGTGTTGGCACAATTCTTAATCAATCCAGACCCATGGGTTGCCATTGCTGCCGCTGAAGTTTATGTCAGACGTTCATACCGTGCTTATGATTTGGGTAMAATTGAATATCATGTTAATGACAGACTTCCTATTGTTGAATGGAAATTCAAGTTGGCTAATATGGGAGCCGCTGGTGTAAACGATGCTCAACAGGCTGCTGCTGCTGGTGGCGATGATTCGACATCTATGAAACATGCAGCTTCTGTGTCTGATTTGACCTTTGTTGTTGATTATTCATAAAAGYGAAATYCCAGGATACACTCTCCCCGATAATCCAAAGTTCACCCTTGGTAATTTGTTTGTAATAATTGGRGTCTTGTTGGTTTGTRTTTTAGCTGTTGTCTCTCTTTTGAGAAATATTAGTGAGTCRGCCTTGTTCAAGAAGAATGGGTATGAACCGTTGGATTCRGATCCTAGTGTCATGAACCWAAACTTYGAGCCTACAACATTGTCCTTTGAAGATATTAAATATGAGGTTACTGGTGGTCGACAAATTTTAAATGGAGTCTTTGGGTTTGTAAAACCAAGAGAATGTTTGGCTATAATGGGAGGTTCAGGTGCTGGTAAAACTACATTGTTGGATTTTAAACCTTTGGACCAATTGGCTAAGACTTTGACCACTGTTCCTGAATTGAATGAAATTATTGGGCAAGATTTAGTTGACGAATTTGTCAGTGGTATTAAACTACCAGCAGAAGTTGGAAGTCAAGATGATGTTAACAATAGAAAATTGTTGCAAAAAGTGTTTGGTAAATTAATGAACACTGATGATGACGTTATAAAACAACAAACAGCTAAATTACTTGAAAGAACAGAAAGGGAACCTCAAGTGTTCAAGGATATTGATTCTAGATTACCAGAGTTAATACAAGGATTAAACAAACAATTTCCTAATGACATCGGATTATTTTGTGGATGTCTCTTATTGAACCACGTTGMTTGAAAAAATTATCAATTTCCGTGTTGGATAAACAAAGATTGACTGAAAAATTCAATAAATTGGATAAATCCATTAAAGATAATTTGAAGGCTAAACAAAAAGAAGAAACCAAAAAGACTTTAGATGTGGTTAACAATTGGTTGAATGATAAAGAAAATGCTTCATCATTTTTGGTTGCTCACGTTCCAATTACTGCTAATGCCAAGGCAATCACTGAAGCCATTAATTTGATTAAAAAGCAAGATAAAACCAAATCAATTTATTTATTGACTGGTGAAACAGATAAAGTTGCTCATGGATGTTATGTTAGTGATGAAGCCATTGCCAAGGGTATTAATGCGAAAATCAAACCTTTTGAAGCACGAGCAATTAACTGGTCMACGGATCTTAATGCTGAGGTACATATTGAGCATTATATAAATATATTCAATTATGCACGATCATCTTGGGAGCCATTGGTTGAAWGTTGGCCAATAGCAGTTTACATGTCAAAATCCCGACACCCAAAGCCTCAATTATTAGTAGAGGTGATTTCTAGACAGGTAGCTCAAGTGACGCTTACATCCAAAGCAGTAGCATTGCTATCTCAAGTATCCGATTTGATTACTTCCRGAGAAAAATTAAAACCAAGAGGTGAAGATTACCCATACRTTATAGTGAATGAGACTGGTTTAGATTTGGAAGTTTGGAATGATGCAAAWGAATCCGAAACCAATGGAGTTCCAATTGTTTTACGTGCTGGTAAAGCTTTAGATGAAAGTAAAGTTGAAATTAGAATTCAATTTAAACCAGTCGCCAAGGGGATGTTTAAAGAAATTCAAAGAAATGAATTAGTTATTAGAGTACAACCAAATGAAGCCATTTATTTAAAAATTAATTCCAAAATCCCTGGAATTTCTACTGAAACTTCATTAACTGATTTAGATTTAACTTATGCTACTCGTTATTCTAAAGACTTTTGGATTCCTGAAGCTTATGAAGCATTAATTAGAGATTGTTATTTAGGTAATCATTCTAATTTTGTTAGAGATGATGAATTGGATGTTTCTTGGAAATTATTTACTCCATTATTGAAT

>Au1_urine_(nephrostomy_urine)_27/05/2015

AATTATTGAAATGGAATGATATTCCATTGGCTCCACCAGACAAAATTTTGGGTATTTCTGAAGCTTATAACAAYGATTCTAACCCTCAAAAAATCAATTTGGGGGTTGGTGCTTATAGAGATAATTCCGGTAAACCAATTATTTTCCCATCAGTTAAAAAAGCTGAAGAAATTTTATTGGGTAAAGAAACTGAAAAGGAATATACTGCCATTGTTGGTTCCAAAAATTTCCAATCAATTGTGAAAAATTTCATTTTCAACAATTCTAATAAAGATGCCAATGGTAAACAATTAATTGATGATGGTAGAATTGTTACTGCTCAAACCATCTCTGGTACTGGATCACTTAAACCTCTTATGGTGAAATTTTTGCTAAACATAGAGAACCAAATTTGGAAATTATTCGTGAGGTTGTTGATTCCAAACATATTGTTTTTGATGTGTTGGCACAATTCTTAATCAATCCAGACCCATGGGTTGCCATTGCTGCCGCTGAAGTTTATGTCAGACGTTCATACCGTGCTTATGATTTGGGTAMAATTGAATATCATGTTAATGACAGACTTCCTATTGTTGAATGGAAATTCAAGTTGGCTAATATGGGAGCCGCTGGTGTAAACGATGCTCAACAGGCTGCTGCTGCTGGTGGCGATGATTCGACATCTATGAAACATGCAGCTTCTGTGTCTGATTTGACCTTTGTTGTTGATTATTCATAAAAGYGAAATYCCAGGATACACTCTCCCCGATAATCCAAAGTTCACCCTTGGTAATTTGTTTGTAATAATTGGRGTCTTGTTGGTTTGTRTTTTAGCTGTTGTCTCTCTTTTGAGAAATATTAGTGAGTCRGCCTTGTTCAAGAAGAATGGGTATGAACCGTTGGATTCRGATCCTAGTGTCATGAACCWAAACTTYGAGCCTACAACATTGTCCTTTGAAGATATTAAATATGAGGTTACTGGTGGTCGACAAATTTTAAATGGAGTCTTTGGGTTTGTAAAACCAAGAGAATGTTTGGCTATAATGGGAGGTTCAGGTGCTGGTAAAACTACATTGTTGGATTTTAAACCTTTGGACCAATTGGCTAAGACTTTGACCACTGTTCCTGAATTGAATGAAATTATTGGGCAAGATTTAGTTGACGAATTTGTCAGTGGTATTAAACTACCAGCAGAAGTTGGAAGTCAAGATGATGTTAACAATAGAAAATTGTTGCAAAAAGTGTTTGGTAAATTAATGAACACTGATGATGACGTTATAAAACAACAAACAGCTAAATTACTTGAAAGAACAGAAAGGGAACCTCAAGTGTTCAAGGATATTGATTCTAGATTACCAGAGTTAATACAAGGATTAAACAAACAATTTCCTAATGACATCGGATTATTTTGTGGATGTCTCTTATTGAACCACGTTGMTTGAAAAAATTATCAATTTCCGTGTTGGATAAACAAAGATTGACTGAAAAATTCAATAAATTGGATAAATCCATTAAAGATAATTTGAAGGCTAAACAAAAAGAAGAAACCAAAAAGACTTTAGATGTGGTTAACAATTGGTTGAATGATAAAGAAAATGCTTCATCATTTTTGGTTGCTCACGTTCCAATTACTGCTAATGCCAAGGCAATCACTGAAGCCATTAATTTGATTAAAAAGCAAGATAAAACCAAATCAATTTATTTATTGACTGGTGAAACAGATAAAGTTGCTCATGGATGTTATGTTAGTGATGAAGCCATTGCCAAGGGTATTAATGCGAAAATCAAACCTTTTGAAGCACGAGCAATTAACTGGTCMACGGATCTTAATGCTGAGGTACATATTGAGCATTATATAAATATATTCAATTATGCACGATCATCTTGGGAGCCATTGGTTGAAWGTTGGCCAATAGCAGTTTACATGTCAAAATCCCGACACCCAAAGCCTCAATTATTAGTAGAGGTGATTTCTAGACAGGTAGCTCAAGTGACGCTTACATCCAAAGCAGTAGCATTGCTATCTCAAGTATCCGATTTGATTACTTCCRGAGAAAAATTAAAACCAAGAGGTGAAGATTACCCATACRTTATAGTGAATGAGACTGGTTTAGATTTGGAAGTTTGGAATGATGCAAAWGAATCCGAAACCAATGGAGTTCCAATTGTTTTACGTGCTGGTAAAGCTTTAGATGAAAGTAAAGTTGAAATTAGAATTCAATTTAAACCAGTCGCCAAGGGGATGTTTAAAGAAATTCAAAGAAATGAATTAGTTATTAGAGTACAACCAAATGAAGCCATTTATTTAAAAATTAATTCCAAAATCCCTGGAATTTCTACTGAAACTTCATTAACTGATTTAGATTTAACTTATGCTACTCGTTATTCTAAAGACTTTTGGATTCCTGAAGCTTATGAAGCATTAATTAGAGATTGTTATTTAGGTAATCATTCTAATTTTGTTAGAGATGATGAATTGGATGTTTCTTGGAAATTATTTACTCCATTATTGAAT

>Au2_blood_(catheter_blood_culture)_4/02/2014

AATTATTGAAATGGAATGATATTCCATTGGCTCCACCAGACAAAATTTTGGGTATTTCTGAAGCTTATAACAACGATTCTAACCCTCAAAAAATCAATTTGGGGGTTGGTGCTTATAGAGATAATTCCGGTAAACCAATTATTTTCCCATCAGTTAAAAAAGCTGAAGAAATTTTATTGGGTAAAGAAACTGAAAAGGAATATACTGCCATTGTTGGTTCCAAAAATTTCCAATCAATTGTGAAAAATTTCATTTTCAACAATTCTAATAAAGATGCCAATGGTAAACAATTAATTGATGATGGTAGAATTGTTACTGCTCAAACCATCTCTGGTACTGGATCACTTAAACCTCTTATGGTGAAATTTTTGCTAAACATAGAGAACCAAATTTGGAAATTATTCGTGAGGTTGTTGATTCCAAACATATTGTTTTTGATGTGTTGGCACAATTCTTAATCAATCCAGACCCATGGGTTGCCATTGCTGCCGCTGAAGTTTATGTCAGACGTTCATACCGTGCTTATGATTTGGGTAAAATTGAATATCATGTTAATGACAGACTTCCTATTGTTGAATGGAAATTCAAGTTGGCTAATATGGGAGCTGCTGGTGTAAACGATGCTCAACAGGCTGCTGCTGCCGGTGGCGATGATTCGACATCTATGAAACATGCAGCTTCTGTGTCTGATTTGACCTTTGTTGTTGATTATTCATAAAAGYGAAATYCCAGGATACACTCTCCCCGATAATCCAAAGTTCACCCTTGGTAATTTGTTTGTAATAATTGGRGTCTTGTTGGTTTGTGTTTTAGCTGTTGTCTCTCTTTTGAGAAATATTAGTGAGTCAGCCTTGTTCAAGAAGAATGGGTATGAACCGTTGGATTCAGATCCTAGTGTCATGAACCAAAACTTTGAGCCTACAACATTGTCCTTTGAAGATATTAAATATGAGGTTACTGGTGSTCGACAAATTTTAAATGGAGTCTTTGGGTTTGTAAAACCAAGAGAATGTTTGGCTATAATGGGAGGTTCAGGTGCTGGTAAAACTACATTGTTGGATTTTAAACCTTTGGACCAATTGGCTAAGACTTTGACCACTGTTCCTGAATTGAATGAAATTATTGGGCAAGATTTAGTTGACGAATTTGTCAGTGGTATTAAACTACCAGCAGAAGTTGGAAGTCAAGATGATGTTAACAATAGAAAATTGTTGCAAAAAGTGTTTGGTAAATTAATGAACACTGATGATGACGTTATAAAACAACAAACAGCTAAATTACTTGAAAGAACAGACAGAGAACCTCAAGTGTTCAAGGATATTGATTCTAGATTACCGGAGTTAATACAAAGATTAAACAAACAATTTCCTAATGACATCGGATTATTTTGTGGATGTCTCTTATTGAACCACGTTGMTTGAAAAAATTATCAATTTCCGTGTTGGATAAACAAAGATTGACTGAAAAATTCAATAAATTGGATAAATCCATYAAAGATAATTTGAAGGCTAAACAAAAAGAAGAAACCAAAAARACTTTAGATGTGGTTAAYAATTGGTTGAATGATAAAGAAAATGCTTCATCATTTTTGGTTGCTCACGTTCCAATTACTGCTAATGCCAAGGCAATCACTGAAGCCATTAATTTGATTAAAAAGCAAGATAAAACCAAATCAATTTATTTATTGACTGGTGAAACCGATAAAGTTGCTCATGGATGTTATGTTAGTGATGAAGCCATTGYCAAGGGTATTAATGCGAAAATCAAACCTTTTGAAGCACGAGCAATTAACTGGTCCACGGATCTTAATGCTGAGGTACATATTGAGCATTATATAAATATATTCAATTATGCACGATCATCTTGGGAGCCATTGGTTGAAAGTTGGCCAATAGCAGTTTACATGTCAAAATCCCGACACCCAAAGCCTCAATTATTAGTAGAGGTGATTTCTAGACAGGTAGCTCAAGTGACGCTTACATCCAAAGCRGTAGCATTGCTATCTCAAGTATCCGATTTGATTACTTCCRGAGAAAAATTAAAACCAAGAGGTGAAGATTACCCATACGTTATARTGAATGAGACTGGTTTAGATTTGGAAGTTTGGAATGATGCAAATGAATCCGAAACCAATGGAGTTCCAATTGTTTTACGTGCTGGTAAAGCTTTAGATGAAAGTAAAGTTGAAATTAGAATTCAATTTAAACCAGTCGCCAAGGGGATGTTTAAAGAAATTCAAAGAAATGAATTAGTTATTAGAGTACAACCAAATGAAGCCATTTATTTAAAAATTAATTCCAAAATCCCTGGAATTTCTACTGAAACTTCATTAACTGATTTAGATTTAACTTATGCTACTCGTTATTCTAAAGACTTTTGGATTCCTGAAGCTTATGAAGCATTAATTAGAGATTGTTATTTAGGTAATCATTCTAATTTTGTTAGAGATGATGAATTGGATGTTTCTTGGAAATTATTTACTCCATTATTGAAT

>Au2_blood_(catheter_blood_culture)_27/01/2014

AATTATTGAAATGGAATGATATTCCATTGGCTCCACCAGACAAAATTTTGGGTATTTCTGAAGCTTATAACAACGATTCTAACCCTCAAAAAATCAATTTGGGGGTTGGTGCTTATAGAGATAATTCCGGTAAACCAATTATTTTCCCATCAGTTAAAAAAGCTGAAGAAATTTTATTGGGTAAAGAAACTGAAAAGGAATATACTGCCATTGTTGGTTCCAAAAATTTCCAATCAATTGTGAAAAATTTCATTTTCAACAATTCTAATAAAGATGCCAATGGTAAACAATTAATTGATGATGGTAGAATTGTTACTGCTCAAACCATCTCTGGTACTGGATCACTTAAACCTCTTATGGTGAAATTTTTGCTAAACATAGAGAACCAAATTTGGAAATTATTCGTGAGGTTGTTGATTCCAAACATATTGTTTTTGATGTGTTGGCACAATTCTTAATCAATCCAGACCCATGGGTTGCCATTGCTGCCGCTGAAGTTTATGTCAGACGTTCATACCGTGCTTATGATTTGGGTAAAATTGAATATCATGTTAATGACAGACTTCCTATTGTTGAATGGAAATTCAAGTTGGCTAATATGGGAGCTGCTGGTGTAAACGATGCTCAACAGGCTGCTGCTGCCGGTGGCGATGATTCGACATCTATGAAACATGCAGCTTCTGTGTCTGATTTGACCTTTGTTGTTGATTATTCATAAAAGYGAAATYCCAGGATACACTCTCCCCGATAATCCAAAGTTCACCCTTGGTAATTTGTTTGTAATAATTGGRGTCTTGTTGGTTTGTGTTTTAGCTGTTGTCTCTCTTTTGAGAAATATTAGTGAGTCAGCCTTGTTCAAGAAGAATGGGTATGAACCGTTGGATTCAGATCCTAGTGTCATGAACCAAAACTTTGAGCCTACAACATTGTCCTTTGAAGATATTAAATATGAGGTTACTGGTGSTCGACAAATTTTAAATGGAGTCTTTGGGTTTGTAAAACCAAGAGAATGTTTGGCTATAATGGGAGGTTCAGGTGCTGGTAAAACTACATTGTTGGATTTTAAACCTTTGGACCAATTGGCTAAGACTTTGACCACTGTTCCTGAATTGAATGAAATTATTGGGCAAGATTTAGTTGACGAATTTGTCAGTGGTATTAAACTACCAGCAGAAGTTGGAAGTCAAGATGATGTTAACAATAGAAAATTGTTGCAAAAAGTGTTTGGTAAATTAATGAACACTGATGATGACGTTATAAAACAACAAACAGCTAAATTACTTGAAAGAACAGACAGAGAACCTCAAGTGTTCAAGGATATTGATTCTAGATTACCGGAGTTAATACAAAGATTAAACAAACAATTTCCTAATGACATCGGATTATTTTGTGGATGTCTCTTATTGAACCACGTTGMTTGAAAAAATTATCAATTTCCGTGTTGGATAAACAAAGATTGACTGAAAAATTCAATAAATTGGATAAATCCATYAAAGATAATTTGAAGGCTAAACAAAAAGAAGAAACCAAAAARACTTTAGATGTGGTTAAYAATTGGTTGAATGATAAAGAAAATGCTTCATCATTTTTGGTTGCTCACGTTCCAATTACTGCTAATGCCAAGGCAATCACTGAAGCCATTAATTTGATTAAAAAGCAAGATAAAACCAAATCAATTTATTTATTGACTGGTGAAACCGATAAAGTTGCTCATGGATGTTATGTTAGTGATGAAGCCATTGYCAAGGGTATTAATGCGAAAATCAAACCTTTTGAAGCACGAGCAATTAACTGGTCCACGGATCTTAATGCTGAGGTACATATTGAGCATTATATAAATATATTCAATTATGCACGATCATCTTGGGAGCCATTGGTTGAAAGTTGGCCAATAGCAGTTTACATGTCAAAATCCCGACACCCAAAGCCTCAATTATTAGTAGAGGTGATTTCTAGACAGGTAGCTCAAGTGACGCTTACATCCAAAGCRGTAGCATTGCTATCTCAAGTATCCGATTTGATTACTTCCRGAGAAAAATTAAAACCAAGAGGTGAAGATTACCCATACGTTATARTGAATGAGACTGGTTTAGATTTGGAAGTTTGGAATGATGCAAATGAATCCGAAACCAATGGAGTTCCAATTGTTTTACGTGCTGGTAAAGCTTTAGATGAAAGTAAAGTTGAAATTAGAATTCAATTTAAACCAGTCGCCAAGGGGATGTTTAAAGAAATTCAAAGAAATGAATTAGTTATTAGAGTACAACCAAATGAAGCCATTTATTTAAAAATTAATTCCAAAATCCCTGGAATTTCTACTGAAACTTCATTAACTGATTTAGATTTAACTTATGCTACTCGTTATTCTAAAGACTTTTGGATTCCTGAAGCTTATGAAGCATTAATTAGAGATTGTTATTTAGGTAATCATTCTAATTTTGTTAGAGATGATGAATTGGATGTTTCTTGGAAATTATTTACTCCATTATTGAAT

>Au2_blood_(catheter_tip)_27/01/201

AATTATTGAAATGGAATGATATTCCATTGGCTCCACCAGACAAAATTTTGGGTATTTCTGAAGCTTATAACAACGATTCTAACCCTCAAAAAATCAATTTGGGGGTTGGTGCTTATAGAGATAATTCCGGTAAACCAATTATTTTCCCATCAGTTAAAAAAGCTGAAGAAATTTTATTGGGTAAAGAAACTGAAAAGGAATATACTGCCATTGTTGGTTCCAAAAATTTCCAATCAATTGTGAAAAATTTCATTTTCAACAATTCTAATAAAGATGCCAATGGTAAACAATTAATTGATGATGGTAGAATTGTTACTGCTCAAACCATCTCTGGTACTGGATCACTTAAACCTCTTATGGTGAAATTTTTGCTAAACATAGAGAACCAAATTTGGAAATTATTCGTGAGGTTGTTGATTCCAAACATATTGTTTTTGATGTGTTGGCACAATTCTTAATCAATCCAGACCCATGGGTTGCCATTGCTGCCGCTGAAGTTTATGTCAGACGTTCATACCGTGCTTATGATTTGGGTAAAATTGAATATCATGTTAATGACAGACTTCCTATTGTTGAATGGAAATTCAAGTTGGCTAATATGGGAGCTGCTGGTGTAAACGATGCTCAACAGGCTGCTGCTGCCGGTGGCGATGATTCGACATCTATGAAACATGCAGCTTCTGTGTCTGATTTGACCTTTGTTGTTGATTATTCATAAAAGYGAAATYCCAGGATACACTCTCCCCGATAATCCAAAGTTCACCCTTGGTAATTTGTTTGTAATAATTGGRGTCTTGTTGGTTTGTGTTTTAGCTGTTGTCTCTCTTTTGAGAAATATTAGTGAGTCAGCCTTGTTCAAGAAGAATGGGTATGAACCGTTGGATTCAGATCCTAGTGTCATGAACCAAAACTTTGAGCCTACAACATTGTCCTTTGAAGATATTAAATATGAGGTTACTGGTGSTCGACAAATTTTAAATGGAGTCTTTGGGTTTGTAAAACCAAGAGAATGTTTGGCTATAATGGGAGGTTCAGGTGCTGGTAAAACTACATTGTTGGATTTTAAACCTTTGGACCAATTGGCTAAGACTTTGACCACTGTTCCTGAATTGAATGAAATTATTGGGCAAGATTTAGTTGACGAATTTGTCAGTGGTATTAAACTACCAGCAGAAGTTGGAAGTCAAGATGATGTTAACAATAGAAAATTGTTGCAAAAAGTGTTTGGTAAATTAATGAACACTGATGATGACGTTATAAAACAACAAACAGCTAAATTACTTGAAAGAACAGACAGAGAACCTCAAGTGTTCAAGGATATTGATTCTAGATTACCGGAGTTAATACAAAGATTAAACAAACAATTTCCTAATGACATCGGATTATTTTGTGGATGTCTCTTATTGAACCACGTTGMTTGAAAAAATTATCAATTTCCGTGTTGGATAAACAAAGATTGACTGAAAAATTCAATAAATTGGATAAATCCATYAAAGATAATTTGAAGGCTAAACAAAAAGAAGAAACCAAAAARACTTTAGATGTGGTTAAYAATTGGTTGAATGATAAAGAAAATGCTTCATCATTTTTGGTTGCTCACGTTCCAATTACTGCTAATGCCAAGGCAATCACTGAAGCCATTAATTTGATTAAAAAGCAAGATAAAACCAAATCAATTTATTTATTGACTGGTGAAACCGATAAAGTTGCTCATGGATGTTATGTTAGTGATGAAGCCATTGYCAAGGGTATTAATGCGAAAATCAAACCTTTTGAAGCACGAGCAATTAACTGGTCCACGGATCTTAATGCTGAGGTACATATTGAGCATTATATAAATATATTCAATTATGCACGATCATCTTGGGAGCCATTGGTTGAAAGTTGGCCAATAGCAGTTTACATGTCAAAATCCCGACACCCAAAGCCTCAATTATTAGTAGAGGTGATTTCTAGACAGGTAGCTCAAGTGACGCTTACATCCAAAGCRGTAGCATTGCTATCTCAAGTATCCGATTTGATTACTTCCRGAGAAAAATTAAAACCAAGAGGTGAAGATTACCCATACGTTATARTGAATGAGACTGGTTTAGATTTGGAAGTTTGGAATGATGCAAATGAATCCGAAACCAATGGAGTTCCAATTGTTTTACGTGCTGGTAAAGCTTTAGATGAAAGTAAAGTTGAAATTAGAATTCAATTTAAACCAGTCGCCAAGGGGATGTTTAAAGAAATTCAAAGAAATGAATTAGTTATTAGAGTACAACCAAATGAAGCCATTTATTTAAAAATTAATTCCAAAATCCCTGGAATTTCTACTGAAACTTCATTAACTGATTTAGATTTAACTTATGCTACTCGTTATTCTAAAGACTTTTGGATTCCTGAAGCTTATGAAGCATTAATTAGAGATTGTTATTTAGGTAATCATTCTAATTTTGTTAGAGATGATGAATTGGATGTTTCTTGGAAATTATTTACTCCATTATTGAAT

>Au2_blood_(peripheral_blood_culture)_4/02/2014

AATTATTGAAATGGAATGATATTCCATTGGCTCCACCAGACAAAATTTTGGGTATTTCTGAAGCTTATAACAACGATTCTAACCCTCAAAAAATCAATTTGGGGGTTGGTGCTTATAGAGATAATTCCGGTAAACCAATTATTTTCCCATCAGTTAAAAAAGCTGAAGAAATTTTATTGGGTAAAGAAACTGAAAAGGAATATACTGCCATTGTTGGTTCCAAAAATTTCCAATCAATTGTGAAAAATTTCATTTTCAACAATTCTAATAAAGATGCCAATGGTAAACAATTAATTGATGATGGTAGAATTGTTACTGCTCAAACCATCTCTGGTACTGGATCACTTAAACCTCTTATGGTGAAATTTTTGCTAAACATAGAGAACCAAATTTGGAAATTATTCGTGAGGTTGTTGATTCCAAACATATTGTTTTTGATGTGTTGGCACAATTCTTAATCAATCCAGACCCATGGGTTGCCATTGCTGCCGCTGAAGTTTATGTCAGACGTTCATACCGTGCTTATGATTTGGGTAAAATTGAATATCATGTTAATGACAGACTTCCTATTGTTGAATGGAAATTCAAGTTGGCTAATATGGGAGCTGCTGGTGTAAACGATGCTCAACAGGCTGCTGCTGCCGGTGGCGATGATTCGACATCTATGAAACATGCAGCTTCTGTGTCTGATTTGACCTTTGTTGTTGATTATTCATAAAAGYGAAATYCCAGGATACACTCTCCCCGATAATCCAAAGTTCACCCTTGGTAATTTGTTTGTAATAATTGGRGTCTTGTTGGTTTGTGTTTTAGCTGTTGTCTCTCTTTTGAGAAATATTAGTGAGTCAGCCTTGTTCAAGAAGAATGGGTATGAACCGTTGGATTCAGATCCTAGTGTCATGAACCAAAACTTTGAGCCTACAACATTGTCCTTTGAAGATATTAAATATGAGGTTACTGGTGSTCGACAAATTTTAAATGGAGTCTTTGGGTTTGTAAAACCAAGAGAATGTTTGGCTATAATGGGAGGTTCAGGTGCTGGTAAAACTACATTGTTGGATTTTAAACCTTTGGACCAATTGGCTAAGACTTTGACCACTGTTCCTGAATTGAATGAAATTATTGGGCAAGATTTAGTTGACGAATTTGTCAGTGGTATTAAACTACCAGCAGAAGTTGGAAGTCAAGATGATGTTAACAATAGAAAATTGTTGCAAAAAGTGTTTGGTAAATTAATGAACACTGATGATGACGTTATAAAACAACAAACAGCTAAATTACTTGAAAGAACAGACAGAGAACCTCAAGTGTTCAAGGATATTGATTCTAGATTACCGGAGTTAATACAAAGATTAAACAAACAATTTCCTAATGACATCGGATTATTTTGTGGATGTCTCTTATTGAACCACGTTGMTTGAAAAAATTATCAATTTCCGTGTTGGATAAACAAAGATTGACTGAAAAATTCAATAAATTGGATAAATCCATYAAAGATAATTTGAAGGCTAAACAAAAAGAAGAAACCAAAAARACTTTAGATGTGGTTAAYAATTGGTTGAATGATAAAGAAAATGCTTCATCATTTTTGGTTGCTCACGTTCCAATTACTGCTAATGCCAAGGCAATCACTGAAGCCATTAATTTGATTAAAAAGCAAGATAAAACCAAATCAATTTATTTATTGACTGGTGAAACCGATAAAGTTGCTCATGGATGTTATGTTAGTGATGAAGCCATTGYCAAGGGTATTAATGCGAAAATCAAACCTTTTGAAGCACGAGCAATTAACTGGTCCACGGATCTTAATGCTGAGGTACATATTGAGCATTATATAAATATATTCAATTATGCACGATCATCTTGGGAGCCATTGGTTGAAAGTTGGCCAATAGCAGTTTACATGTCAAAATCCCGACACCCAAAGCCTCAATTATTAGTAGAGGTGATTTCTAGACAGGTAGCTCAAGTGACGCTTACATCCAAAGCRGTAGCATTGCTATCTCAAGTATCCGATTTGATTACTTCCRGAGAAAAATTAAAACCAAGAGGTGAAGATTACCCATACGTTATARTGAATGAGACTGGTTTAGATTTGGAAGTTTGGAATGATGCAAATGAATCCGAAACCAATGGAGTTCCAATTGTTTTACGTGCTGGTAAAGCTTTAGATGAAAGTAAAGTTGAAATTAGAATTCAATTTAAACCAGTCGCCAAGGGGATGTTTAAAGAAATTCAAAGAAATGAATTAGTTATTAGAGTACAACCAAATGAAGCCATTTATTTAAAAATTAATTCCAAAATCCCTGGAATTTCTACTGAAACTTCATTAACTGATTTAGATTTAACTTATGCTACTCGTTATTCTAAAGACTTTTGGATTCCTGAAGCTTATGAAGCATTAATTAGAGATTGTTATTTAGGTAATCATTCTAATTTTGTTAGAGATGATGAATTGGATGTTTCTTGGAAATTATTTACTCCATTATTGAAT

>Au2_feces_28/01/2014

AATTATTGAAATGGAATGATATTCCATTGGCTCCACCAGACAAAATTTTGGGTATTTCTGAAGCTTATAACAACGATTCTAACCCTCAAAAAATCAATTTGGGGGTTGGTGCTTATAGAGATAATTCCGGTAAACCAATTATTTTCCCATCAGTTAAAAAAGCTGAAGAAATTTTATTGGGTAAAGAAACTGAAAAGGAATATACTGCCATTGTTGGTTCCAAAAATTTCCAATCAATTGTGAAAAATTTCATTTTCAACAATTCTAATAAAGATGCCAATGGTAAACAATTAATTGATGATGGTAGAATTGTTACTGCTCAAACCATCTCTGGTACTGGATCACTTAAACCTCTTATGGTGAAATTTTTGCTAAACATAGAGAACCAAATTTGGAAATTATTCGTGAGGTTGTTGATTCCAAACATATTGTTTTTGATGTGTTGGCACAATTCTTAATCAATCCAGACCCATGGGTTGCCATTGCTGCCGCTGAAGTTTATGTCAGACGTTCATACCGTGCTTATGATTTGGGTAAAATTGAATATCATGTTAATGACAGACTTCCTATTGTTGAATGGAAATTCAAGTTGGCTAATATGGGAGCTGCTGGTGTAAACGATGCTCAACAGGCTGCTGCTGCCGGTGGCGATGATTCGACATCTATGAAACATGCAGCTTCTGTGTCTGATTTGACCTTTGTTGTTGATTATTCATAAAAGYGAAATYCCAGGATACACTCTCCCCGATAATCCAAAGTTCACCCTTGGTAATTTGTTTGTAATAATTGGRGTCTTGTTGGTTTGTGTTTTAGCTGTTGTCTCTCTTTTGAGAAATATTAGTGAGTCAGCCTTGTTCAAGAAGAATGGGTATGAACCGTTGGATTCAGATCCTAGTGTCATGAACCAAAACTTTGAGCCTACAACATTGTCCTTTGAAGATATTAAATATGAGGTTACTGGTGSTCGACAAATTTTAAATGGAGTCTTTGGGTTTGTAAAACCAAGAGAATGTTTGGCTATAATGGGAGGTTCAGGTGCTGGTAAAACTACATTGTTGGATTTTAAACCTTTGGACCAATTGGCTAAGACTTTGACCACTGTTCCTGAATTGAATGAAATTATTGGGCAAGATTTAGTTGACGAATTTGTCAGTGGTATTAAACTACCAGCAGAAGTTGGAAGTCAAGATGATGTTAACAATAGAAAATTGTTGCAAAAAGTGTTTGGTAAATTAATGAACACTGATGATGACGTTATAAAACAACAAACAGCTAAATTACTTGAAAGAACAGACAGAGAACCTCAAGTGTTCAAGGATATTGATTCTAGATTACCGGAGTTAATACAAAGATTAAACAAACAATTTCCTAATGACATCGGATTATTTTGTGGATGTCTCTTATTGAACCACGTTGMTTGAAAAAATTATCAATTTCCGTGTTGGATAAACAAAGATTGACTGAAAAATTCAATAAATTGGATAAATCCATYAAAGATAATTTGAAGGCTAAACAAAAAGAAGAAACCAAAAARACTTTAGATGTGGTTAAYAATTGGTTGAATGATAAAGAAAATGCTTCATCATTTTTGGTTGCTCACGTTCCAATTACTGCTAATGCCAAGGCAATCACTGAAGCCATTAATTTGATTAAAAAGCAAGATAAAACCAAATCAATTTATTTATTGACTGGTGAAACCGATAAAGTTGCTCATGGATGTTATGTTAGTGATGAAGCCATTGYCAAGGGTATTAATGCGAAAATCAAACCTTTTGAAGCACGAGCAATTAACTGGTCCACGGATCTTAATGCTGAGGTACATATTGAGCATTATATAAATATATTCAATTATGCACGATCATCTTGGGAGCCATTGGTTGAAAGTTGGCCAATAGCAGTTTACATGTCAAAATCCCGACACCCAAAGCCTCAATTATTAGTAGAGGTGATTTCTAGACAGGTAGCTCAAGTGACGCTTACATCCAAAGCRGTAGCATTGCTATCTCAAGTATCCGATTTGATTACTTCCRGAGAAAAATTAAAACCAAGAGGTGAAGATTACCCATACGTTATARTGAATGAGACTGGTTTAGATTTGGAAGTTTGGAATGATGCAAATGAATCCGAAACCAATGGAGTTCCAATTGTTTTACGTGCTGGTAAAGCTTTAGATGAAAGTAAAGTTGAAATTAGAATTCAATTTAAACCAGTCGCCAAGGGGATGTTTAAAGAAATTCAAAGAAATGAATTAGTTATTAGAGTACAACCAAATGAAGCCATTTATTTAAAAATTAATTCCAAAATCCCTGGAATTTCTACTGAAACTTCATTAACTGATTTAGATTTAACTTATGCTACTCGTTATTCTAAAGACTTTTGGATTCCTGAAGCTTATGAAGCATTAATTAGAGATTGTTATTTAGGTAATCATTCTAATTTTGTTAGAGATGATGAATTGGATGTTTCTTGGAAATTATTTACTCCATTATTGAAT

>Au2_feces_28/01/2014:IND:17:51.9

AATTATTGAAATGGAATGATATTCCATTGGCTCCACCAGACAAGATTTTGGGTATTTCTGAAGCTTATAACAATGATTCTAACCCTCAAAAAGTCAATTTGGGGGTTGGTGCTTATAGAGATAATTCTGGTAAACCAATTATTTTCCCATCAGTTAAAAAAGCTGAAGAAATTTTATTGGGTAAAGAAACTGAAAAGGAATATACTGCCATTGTTGGTTCCAAAAATTTCCAATCAATTGTGAAAAATTTCATTTTCAACAATTCTAATAAAGATGCCAATGGTAAACAATTAATTGATGATGGTAGAATTGTTACTGCTCAAACCATYTCTGGTACTGGATCACTTAAACCTCTTATGGTGAAATTTTTGCTAAACATAGAGAACCAAATTTGGAAATTATTCGTGAGGTTGTTGATTCCAAACATATTGTTTTTGATGTGTTGGCACAATTCTTAATCAATCCAGACCCATGGGTTGCCATTGCTGCCGCTGAAGTTTATGTCAGACGTTCATACCGTGCTTATGATTTGGGTAAAATTGAATATCATGTTAATGACAGACTTCCTATTGTTGAATGGAAATTCAAGTTGGCTAATATGGGAGCYGCTGGTGTAAACGATGCTCAACAGGCTGCTGCTGCCGGTGGCGATGATTCGACATCTATGAAACATGCAGCTTCTGTGTCTGATTTGACCTTTGTTGTTGATTATTCATAAAAGYGAAATYCCAGGATACACTCTCCCCGATAATCCAAAGTTCACCCTTGGTAATTTGTTTGTAATAATTGGRGTCTTGTTGGTTTGTATTTTAGCTGTTGTCTCTCTTTTGAGAAATATTAGTGAGTCGGCCTTGTTCAAGAAGAATGGGTATGAACCGTTGGATTCGGATCCTAGTGTCATGAACCTAAACTTCGAGCCTACAACATTGTCCTTTGAAGATATTAAATATGAGGTTACTGGTGGTCGACAAATTTTAAATGGAGTCTTTGGGTTTGTAAAACCAAGAGAATGTTTGGCTATAATGGGAGGTTCAGGTGCTGGTAAAACTACATTGTTGGATTTTAAACCTTTGGACCAATTGGCTAAAACTTTGGCTACTGTTCCTGAATTGAATGAAATTATTGGTCAAGAATTAGTTGACGAATTTATCAGTGGTATTAAACTACCAGCAGAAGTTGGAAGTCAAGATGATGTTAACAATAGAAAATTGTTGCAAAAAGTGTTTGGTAAATTAATGAACACTGATGATGACGTTATAAAACAACAAACAGCTAAATTACTTGAAAGAACAGAAAGGCAACCTCAAGTGTTCAAGGATATTGATTCTAGATTACCGGAGTTAATACAAAGATTAAACAAACAATTTCCTAATGACATCGGATTATTTTGTGGATGTCTCTTATTGAACCACGTTGMTTGAAAAAATTATCAATTTCCGTGTTGGATAAACARAGATTGACTGAAAAATTCAATAAATTGGATAAATCCATYAAAGATAATTTGAAGGCTAAACAAAAAGAAGAAACCAAAAARACTTTAGATGTGGTTAACAATTGGTTGAATGATAAAGAAAATKCTTCATCATTTTTGGTTGCTCACGTTCCAATTACTGCTAATGCCAAGGCAATCACTGAAGCCATTAATTTGATTAAAAAGCAAGATAAAACCAAATCAATTTATTTATTGACTGGTGAAACCGATAAAGTTGCTCATGGATGTTATGTTAGTGATGAAGCCATTGYCAAGGGTATTAATGCGAAAATCAAACCTTTTGAAGCACGAGCAATTAACTGGTCMACGGATCTTAATGCTGAGGTACATATTGAGCATTATATAAATATATTCAATTATGCACGATCATCTTGGGAGCCATTGGTTGAAAGTTGGCCAATAGCAGTTTACATGTCAAAATCCCGACACCCAAAGCCTCAATTATTAGTAGAGGTGATTTCTAGACARGTAGCTCAAGTGACGCTTACATCCAAAGCAGTAGCATTGCTATCTCAAGTATCCGATTTGATTACTTCCGGAGAAAAATTAAAACCAAGAGGTGAAGATTACCCATACRTGATAGTGAATGAGACTGGTTTAGATTTGGAAGTTTGGAATGATGCAAAAGAATTCGAAACCAATGGAGTTCCAATTGTTTTACGTGCTGGTAAAGCTTTAGATGAAAGTAAAGTTGAAATTAGAATTCAATTTAAACCAGTCGCCAAGGGGATGTTTAAAGAAATTCAAAGAAATGAATTAGTTATTAGAGTACAACCAAATGAAGCCATTTATTTAAAAATTAATTCCAAAATCCCTGGGATTTCTACTGAAACTTCATTAACTGATTTAGATTTAACTTATGCTACTCGTTATTCTAAAGACTTTTGGATTCCTGAAGCTTATGAAGCATTAATTAGAGATTGCTATTTAGGTAATCATTCTAATTTTGTTAGAGATGATGAATTGGATGTTTCTTGGAAATTATTTACTCCATTATTGAAT

>Au2_feces_28/01/2014:IND:18:19.7

AATTATTGAAATGGAATGATATTCCATTGGCTCCACCAGACAAGATTTTGGGTATTTCTGAAGCTTATAACAATGATTCTAACCCTCAAAAAGTCAATTTGGGGGTTGGTGCTTATAGAGATAATTCTGGTAAACCAATTATTTTCCCATCAGTTAAAAAAGCTGAAGAAATTTTATTGGGTAAAGAAACTGAAAAGGAATATACTGCCATTGTTGGTTCCAAAAATTTCCAATCAATTGTGAAAAATTTCATTTTCAACAATTCTAATAAAGATGCCAATGGTAAACAATTAATTGATGATGGTAGAATTGTTACTGCTCAAACCATYTCTGGTACTGGATCACTTAAACCTCTTATGGTGAAATTTTTGCTAAACATAGAGAACCAAATTTGGAAATTATTCGTGAGGTTGTTGATTCCAAACATATTGTTTTTGATGTGTTGGCACAATTCTTAATCAATCCAGACCCATGGGTTGCCATTGCTGCCGCTGAAGTTTATGTCAGACGTTCATACCGTGCTTATGATTTGGGTAAAATTGAATATCATGTTAATGACAGACTTCCTATTGTTGAATGGAAATTCAAGTTGGCTAATATGGGAGCYGCTGGTGTAAACGATGCTCAACAGGCTGCTGCTGCCGGTGGCGATGATTCGACATCTATGAAACATGCAGCTTCTGTGTCTGATTTGACCTTTGTTGTTGATTATTCATAAAAGYGAAATYCCAGGATACACTCTCCCCGATAATCCAAAGTTCACCCTTGGTAATTTGTTTGTAATAATTGGRGTCTTGTTGGTTTGTATTTTAGCTGTTGTCTCTCTTTTGAGAAATATTAGTGAGTCGGCCTTGTTCAAGAAGAATGGGTATGAACCGTTGGATTCGGATCCTAGTGTCATGAACCTAAACTTCGAGCCTACAACATTGTCCTTTGAAGATATTAAATATGAGGTTACTGGTGGTCGACAAATTTTAAATGGAGTCTTTGGGTTTGTAAAACCAAGAGAATGTTTGGCTATAATGGGAGGTTCAGGTGCTGGTAAAACTACATTGTTGGATTTTAAACCTTTGGACCAATTGGCTAAAACTTTGGCTACTGTTCCTGAATTGAATGAAATTATTGGTCAAGAATTAGTTGACGAATTTATCAGTGGTATTAAACTACCAGCAGAAGTTGGAAGTCAAGATGATGTTAACAATAGAAAATTGTTGCAAAAAGTGTTTGGTAAATTAATGAACACTGATGATGACGTTATAAAACAACAAACAGCTAAATTACTTGAAAGAACAGAAAGGCAACCTCAAGTGTTCAAGGATATTGATTCTAGATTACCGGAGTTAATACAAAGATTAAACAAACAATTTCCTAATGACATCGGATTATTTTGTGGATGTCTCTTATTGAACCACGTTGMTTGAAAAAATTATCAATTTCCGTGTTGGATAAACARAGATTGACTGAAAAATTCAATAAATTGGATAAATCCATYAAAGATAATTTGAAGGCTAAACAAAAAGAAGAAACCAAAAARACTTTAGATGTGGTTAACAATTGGTTGAATGATAAAGAAAATKCTTCATCATTTTTGGTTGCTCACGTTCCAATTACTGCTAATGCCAAGGCAATCACTGAAGCCATTAATTTGATTAAAAAGCAAGATAAAACCAAATCAATTTATTTATTGACTGGTGAAACCGATAAAGTTGCTCATGGATGTTATGTTAGTGATGAAGCCATTGYCAAGGGTATTAATGCGAAAATCAAACCTTTTGAAGCACGAGCAATTAACTGGTCMACGGATCTTAATGCTGAGGTACATATTGAGCATTATATAAATATATTCAATTATGCACGATCATCTTGGGAGCCATTGGTTGAAAGTTGGCCAATAGCAGTTTACATGTCAAAATCCCGACACCCAAAGCCTCAATTATTAGTAGAGGTGATTTCTAGACARGTAGCTCAAGTGACGCTTACATCCAAAGCAGTAGCATTGCTATCTCAAGTATCCGATTTGATTACTTCCGGAGAAAAATTAAAACCAAGAGGTGAAGATTACCCATACRTGATAGTGAATGAGACTGGTTTAGATTTGGAAGTTTGGAATGATGCAAAAGAATTCGAAACCAATGGAGTTCCAATTGTTTTACGTGCTGGTAAAGCTTTAGATGAAAGTAAAGTTGAAATTAGAATTCAATTTAAACCAGTCGCCAAGGGGATGTTTAAAGAAATTCAAAGAAATGAATTAGTTATTAGAGTACAACCAAATGAAGCCATTTATTTAAAAATTAATTCCAAAATCCCTGGGATTTCTACTGAAACTTCATTAACTGATTTAGATTTAACTTATGCTACTCGTTATTCTAAAGACTTTTGGATTCCTGAAGCTTATGAAGCATTAATTAGAGATTGCTATTTAGGTAATCATTCTAATTTTGTTAGAGATGATGAATTGGATGTTTCTTGGAAATTATTTACTCCATTATTGAAT

>Au3_blood_(peripheral_blood_culture)_5/04/2015

AATTATTGAARTGGAATGATATTCCATTGGCTCCACCAGACAARATTTTGGGTATTTCTGAAGCTTATAACAATGATTCTAACCCTCAAAAARTCAATTTGGGGGTTGGTGCTTATAGAGATAATTCYGGTAAACCAATTATTTTCCCATCAGTTAAAAAAGCTGAAGAAATTTTATTGGGTAAAGAAACTGAAAAGGAATATACTGCCATTGTTGGTTCCAAAAATTTCCAATCAATTGTGAAAAATTTCATTTTCAACAATTCTAATAAAGATGCCAATGGTAAACAATTAATTGATGATGGTAGAATTGTTACTGCTCAAACCATCTCTGGTACTGGATCACTTAAACCTCTTATGGTGAAATTTTTGCTAAACATAGAGAACCAAATTTGGAAATTATTCGTGAGGTTGTTGATTCCAAACATATTGTTTTTGATGTGTTGGCACAATTCTTAATCAATCCAGACCCATGGGTTGCCATTGCTGCCGCTGAAGTTTATGTCAGACGTTCATACCGTGCTTATGATTTGGGTAAAATTGAATATCATGTTAATGACAGACTTCCTATTGTTGAATGGAAATTCAAGTTGGCTAATATGGGAGCCGCTGGTGTAAACGATGCTCAACAGGCTGCTGCTGCCGGTGGCGATGATTCGACATCTATGAAACATGCAGCTTCTGTGTCTGATTTGACCTTTGTTGTTGATTATTCATRAAAGCGAAATCCCAGGATACACTCTCCCCGATAATCCAAAGTTCACCCTTGGTAATTTGTTTGTAATAATTGGAGTCTTGTTGGTTTGTATTTTAGCTGTTGTCTCTCTTTTGAGAAATATTAGTGAGTCGGCCTTGTTCAAGAAGAATGGGTATGAACCGTTGGATTCGGATCCTAGTGTCATGAACCTAAACTTCGAGCCTACAACATTGTCCTTTGAAGATATTAAATATGAGGTTACTGGTGGTCGACAAATTTTAAATGGAGTCTTTGGGTTTGTAAAACCAAGAGAATGTTTGGCTATAATGGGAGGTTCAGGTGCTGGTAAAACTACATTGTTGGATTTTAAACCTTTGGACCAATTGGCTAARACTTTGRCYACTGTTCCTGAATTGAATGAAATTATTGGKCAAGAWTTAGTTGACGAATTTRTCAGTGGTATTAAACTACCAGCAGAAGTTGGAAGTCAAGATGATGTTAACAATAGAAAATTGTTGCAAAAAGTGTTTGGTAAATTAATGAACACTGATGATGACGTTATAAAACAACAAACAGCTAAATTACTTGAAAGAACAGACAGAGAACCTCAAGTGTTCAAGGATATTGATTCTAGATTACCGGAGTTAATACAAAGATTAAACAAACAATTTCCTAATGACATCGGATTATTTTGTGGATGTCTCTTATTGAACCACGTTGATTGAAAAAATTATCAATTTCCGTGTTGGATAAACAGAGATTGACTGAAAAATTCAATAAATTGGATAAATCCATCAAAGATAATTTGAAGGCTAAACAAAAAGAAGAAACCAAAAAAACTTTAGATGTGGTTAATAATTGGTTGAATGATAAAGAAAATGCTTCATCATTTTTGGTTGCTCACGTTCCAATTACTGCTAATGCCAAGGCAATCACTGAAGCCATTAATTTGATTAAAAAGCAAGATAAAACCAAATCAATTTATTTATTGACTGGTGAAACCGATAAAGTTGCTCATGGATGTTATGTTAGTGATGAAGCCATTGCCAAGGGTATTAATGCGAAAATCAAACCTTTTGAAGCACGAGCAATTAACTGGTCCACGGATCTTAATGCTGAGGTACATATTGAGCATTATATAAATATATTCAATTATGCACGATCATCTTGGGAGCCATTGGTTGAAAGTTGGCCAATAGCAGTTTACATGTCAAAATCCCGACACCCAAAGCCTCAATTATTAGTAGAGGTGATTTCTAGACAGGTAGCYCAAGTGACGCTTACATCCAAAGCAGTAGCATTGCTATCTCAAGTATCCGATTTGATTACTTCCRGAGAAAAATTAAAACCAAGAGGTGAAGATTACCCATACGTKATAGTGAATGAGACTGGTTTAGATTTGGAAGTTTGGAATGATGCAAAWGAATYCGAAACCAATGGAGTTCCAATTGTTTTACGTGCTGGTAAAGCTTTAGATGAAAGTAAAGTTGAAATTAGAATTCAATTTAAACCAGTCGCCAAGGGGATGTTTAAAGAAATTCAAAGAAATGAATTAGTTATTAGAGTACAACCAAATGAAGCCATTTATTTAAAAATTAATTCCAAAATCCCTGGAATTTCTACTGAAACTTCATTAACTGATTTAGATTTAACTTATGCTACTCGTTATTCTAAAGATTTTTGGATTCCTGAAGCTTATGAAGCATTAATTAGAGATTGTTATTTAGGTAATCATTCTAATTTTGTTAGAGATGATGAATTGGATGTTTCTTGGAAATTATTTACTCCATTATTGAAT

>Au3_urine_(midstream_urine)_5/04/2015

AATTATTGAARTGGAATGATATTCCATTGGCTCCACCAGACAARATTTTGGGTATTTCTGAAGCTTATAACAATGATTCTAACCCTCAAAAARTCAATTTGGGGGTTGGTGCTTATAGAGATAATTCYGGTAAACCAATTATTTTCCCATCAGTTAAAAAAGCTGAAGAAATTTTATTGGGTAAAGAAACTGAAAAGGAATATACTGCCATTGTTGGTTCCAAAAATTTCCAATCAATTGTGAAAAATTTCATTTTCAACAATTCTAATAAAGATGCCAATGGTAAACAATTAATTGATGATGGTAGAATTGTTACTGCTCAAACCATCTCTGGTACTGGATCACTTAAACCTCTTATGGTGAAATTTTTGCTAAACATAGAGAACCAAATTTGGAAATTATTCGTGAGGTTGTTGATTCCAAACATATTGTTTTTGATGTGTTGGCACAATTCTTAATCAATCCAGACCCATGGGTTGCCATTGCTGCCGCTGAAGTTTATGTCAGACGTTCATACCGTGCTTATGATTTGGGTAAAATTGAATATCATGTTAATGACAGACTTCCTATTGTTGAATGGAAATTCAAGTTGGCTAATATGGGAGCCGCTGGTGTAAACGATGCTCAACAGGCTGCTGCTGCCGGTGGCGATGATTCGACATCTATGAAACATGCAGCTTCTGTGTCTGATTTGACCTTTGTTGTTGATTATTCATRAAAGCGAAATCCCAGGATACACTCTCCCCGATAATCCAAAGTTCACCCTTGGTAATTTGTTTGTAATAATTGGAGTCTTGTTGGTTTGTATTTTAGCTGTTGTCTCTCTTTTGAGAAATATTAGTGAGTCGGCCTTGTTCAAGAAGAATGGGTATGAACCGTTGGATTCGGATCCTAGTGTCATGAACCTAAACTTCGAGCCTACAACATTGTCCTTTGAAGATATTAAATATGAGGTTACTGGTGGTCGACAAATTTTAAATGGAGTCTTTGGGTTTGTAAAACCAAGAGAATGTTTGGCTATAATGGGAGGTTCAGGTGCTGGTAAAACTACATTGTTGGATTTTAAACCTTTGGACCAATTGGCTAARACTTTGRCYACTGTTCCTGAATTGAATGAAATTATTGGKCAAGAWTTAGTTGACGAATTTRTCAGTGGTATTAAACTACCAGCAGAAGTTGGAAGTCAAGATGATGTTAACAATAGAAAATTGTTGCAAAAAGTGTTTGGTAAATTAATGAACACTGATGATGACGTTATAAAACAACAAACAGCTAAATTACTTGAAAGAACAGACAGAGAACCTCAAGTGTTCAAGGATATTGATTCTAGATTACCGGAGTTAATACAAAGATTAAACAAACAATTTCCTAATGACATCGGATTATTTTGTGGATGTCTCTTATTGAACCACGTTGATTGAAAAAATTATCAATTTCCGTGTTGGATAAACAGAGATTGACTGAAAAATTCAATAAATTGGATAAATCCATCAAAGATAATTTGAAGGCTAAACAAAAAGAAGAAACCAAAAAAACTTTAGATGTGGTTAATAATTGGTTGAATGATAAAGAAAATGCTTCATCATTTTTGGTTGCTCACGTTCCAATTACTGCTAATGCCAAGGCAATCACTGAAGCCATTAATTTGATTAAAAAGCAAGATAAAACCAAATCAATTTATTTATTGACTGGTGAAACCGATAAAGTTGCTCATGGATGTTATGTTAGTGATGAAGCCATTGCCAAGGGTATTAATGCGAAAATCAAACCTTTTGAAGCACGAGCAATTAACTGGTCCACGGATCTTAATGCTGAGGTACATATTGAGCATTATATAAATATATTCAATTATGCACGATCATCTTGGGAGCCATTGGTTGAAAGTTGGCCAATAGCAGTTTACATGTCAAAATCCCGACACCCAAAGCCTCAATTATTAGTAGAGGTGATTTCTAGACAGGTAGCYCAAGTGACGCTTACATCCAAAGCAGTAGCATTGCTATCTCAAGTATCCGATTTGATTACTTCCRGAGAAAAATTAAAACCAAGAGGTGAAGATTACCCATACGTKATAGTGAATGAGACTGGTTTAGATTTGGAAGTTTGGAATGATGCAAAWGAATYCGAAACCAATGGAGTTCCAATTGTTTTACGTGCTGGTAAAGCTTTAGATGAAAGTAAAGTTGAAATTAGAATTCAATTTAAACCAGTCGCCAAGGGGATGTTTAAAGAAATTCAAAGAAATGAATTAGTTATTAGAGTACAACCAAATGAAGCCATTTATTTAAAAATTAATTCCAAAATCCCTGGAATTTCTACTGAAACTTCATTAACTGATTTAGATTTAACTTATGCTACTCGTTATTCTAAAGATTTTTGGATTCCTGAAGCTTATGAAGCATTAATTAGAGATTGTTATTTAGGTAATCATTCTAATTTTGTTAGAGATGATGAATTGGATGTTTCTTGGAAATTATTTACTCCATTATTGAAT

>Au4_blood_(catheter_blood_culture)_31/05/2014

AATTATTGAARTGGAATGATATTCCATTGGCTCCACCAGACAARATTTTGGGTATTTCTGAAGCTTATAACAATGATTCTAACCCTCAAAAARTCAATTTGGGGGTTGGTGCTTATAGAGATAATTCYGGTAAACCAATTATTTTCCCATCAGTTAAAAAAGCTGAAGAAATTTTATTGGGTAAAGAAACTGAAAAGGAATATACTGCCATTGTTGGTTCCAAAAATTTCCAATCAATTGTGAAAAATTTCATTTTCAACAATTCTAATAAAGATGCCAATGGTAAACAATTAATTGATGATGGTAGAATTGTTACTGCTCAAACCATCTCTGGTACTGGATCACTTAAACCTCTTATGGTGAAATTTTTGCTAAACATAGAGAACCAAATTTGGAAATTATTCGTGAGGTTGTTGATTCCAAACATATTGTTTTTGATGTGTTGGCACAATTCTTAATCAATCCAGACCCATGGGTTGCCATTGCTGCCGCTGAAGTTTATGTCAGACGTTCATACCGTGCTTATGATTTGGGTAAAATTGAATATCATGTTAATGACAGACTTCCTATTGTTGAATGGAAATTCAAGTTGGCTAATATGGGAGCCGCTGGTGTAAACGATGCTCAACAGGCTGCTGCTGCCGGTGGCGATGATTCGACATCTATGAAACATGCAGCTTCTGTGTCTGATTTGACCTTTGTTGTTGATTATTCATGAAAGCGAAATCCCAGGATACACTCTCCCCGATAATCCAAAGTTCACCCTTGGTAATTTGTTTGTAATAATTGGAGTCTTGTTGGTTTGTATTTTAGCTGTTGTCTCTCTTTTGAGAAATATTAGTGAGTCGGCCTTGTTCAAGAAGAATGGGTATGAACCGTTGGATTCGGATCCTAGTGTCATGAACCTAAACTTCGAGCCTACAACATTGTCCTTTGAAGATATTAAATATGAGGTTACTGGTGGTCGACAAATTTTAAATGGAGTCTTTGGGTTTGTAAAACCAAGAGAATGTTTGGCTATAATGGGAGGTTCAGGTGCTGGTAAAACTACATTGTTGGATTTTAAACCTTTGGACCAATTGGCTAARACTTTGRCYACTGTTCCTGAATTGAATGAAATTATTGGKCAAGAWTTAGTTGACGAATTTRTCAGTGGTATTAAACTACCAGCAGAAGTTGGAAGTCAAGATGATGTTAACAATAGAAAATTGTTGCAAAAAGTGTTTGGTAAATTAATGAACACTGATGATGACGTTATAAAACAACAAACAGCTAAATTACTTGAAAGAACAGACAGAGAACCTCAAGTGTTCAAGGATATTGATTCTAGATTACCGGAGTTAATACAAAGATTAAACAAACAATTTCCTAATGACATCGGATTATTTTGTGGATGTCTCTTATTGAACCACGTTGATTGAAAAAATTATCAATTTCCGTGTTGGATAAACAGAGATTGACTGAAAAATTCAATAAATTGGATAAATCCATCAAAGATAATTTGAAGGCTAAACAAAAAGAAGAAACCAAAAAAACTTTAGATGTGGTTAATAATTGGTTGAATGATAAAGAAAATGCTTCATCATTTTTGGTTGCTCACGTTCCAATTACTGCTAATGCCAAGGCAATCACTGAAGCCATTAATTTGATTAAAAAGCAAGATAAAACCAAATCAATTTATTTATTGACTGGTGAAACCGATAAAGTTGCTCATGGATGTTATGTTAGTGATGAAGCCATTGCCAAGGGTATTAATGCGAAAATCAAACCTTTTGAAGCACGAGCAATTAACTGGTCCACGGATCTTAATGCTGAGGTACATATTGAGCATTATATAAATATATTCAATTATGCACGATCATCTTGGGAGCCATTGGTTGAAAGTTGGCCAATAGCAGTTTACATGTCAAAATCCCGACACCCAAAGCCTCAATTATTAGTAGAGGTGATTTCTAGACAGGTAGCYCAAGTGACGCTTACATCCAAAGCAGTAGCATTGCTATCTCAAGTATCCGATTTGATTACTTCCRGAGAAAAATTAAAACCAAGAGGTGAAGATTACCCATACGTKATAGTGAATGAGACTGGTTTAGATTTGGAAGTTTGGAATGATGCAAAWGAATYCGAAACCAATGGAGTTCCAATTGTTTTACGTGCTGGTAAAGCTTTAGATGAAAGTAAAGTTGAAATTAGAATTCAATTTAAACCAGTCGCCAAGGGGATGTTTAAAGAAATTCAAAGAAATGAATTAGTTATTAGAGTACAACCAAATGAAGCCATTTATTTAAAAATTAATTCCAAAATCCCTGGAATTTCTACTGAAACTTCATTAACTGATTTAGATTTAACTTATGCTACTCGTTATTCTAAAGACTTTTGGATTCCTGAAGCTTATGAAGCATTAATTAGAGATTGCTATTTAGGTAATCATTCTAATTTTGTTAGAGATGATGAATTGGATGTTTCTTGGAAATTATTTACTCCATTATTGAAT

>Au4_tracheal_aspirate_4/06/2014

AATTATTGAARTGGAATGATATTCCATTGGCTCCACCAGACAARATTTTGGGTATTTCTGAAGCTTATAACAATGATTCTAACCCTCAAAAARTCAATTTGGGGGTTGGTGCTTATAGAGATAATTCYGGTAAACCAATTATTTTCCCATCAGTTAAAAAAGCTGAAGAAATTTTATTGGGTAAAGAAACTGAAAAGGAATATACTGCCATTGTTGGTTCCAAAAATTTCCAATCAATTGTGAAAAATTTCATTTTCAACAATTCTAATAAAGATGCCAATGGTAAACAATTAATTGATGATGGTAGAATTGTTACTGCTCAAACCATCTCTGGTACTGGATCACTTAAACCTCTTATGGTGAAATTTTTGCTAAACATAGAGAACCAAATTTGGAAATTATTCGTGAGGTTGTTGATTCCAAACATATTGTTTTTGATGTGTTGGCACAATTCTTAATCAATCCAGACCCATGGGTTGCCATTGCTGCCGCTGAAGTTTATGTCAGACGTTCATACCGTGCTTATGATTTGGGTAAAATTGAATATCATGTTAATGACAGACTTCCTATTGTTGAATGGAAATTCAAGTTGGCTAATATGGGAGCCGCTGGTGTAAACGATGCTCAACAGGCTGCTGCTGCCGGTGGCGATGATTCGACATCTATGAAACATGCAGCTTCTGTGTCTGATTTGACCTTTGTTGTTGATTATTCATGAAAGCGAAATCCCAGGATACACTCTCCCCGATAATCCAAAGTTCACCCTTGGTAATTTGTTTGTAATAATTGGAGTCTTGTTGGTTTGTATTTTAGCTGTTGTCTCTCTTTTGAGAAATATTAGTGAGTCGGCCTTGTTCAAGAAGAATGGGTATGAACCGTTGGATTCGGATCCTAGTGTCATGAACCTAAACTTCGAGCCTACAACATTGTCCTTTGAAGATATTAAATATGAGGTTACTGGTGGTCGACAAATTTTAAATGGAGTCTTTGGGTTTGTAAAACCAAGAGAATGTTTGGCTATAATGGGAGGTTCAGGTGCTGGTAAAACTACATTGTTGGATTTTAAACCTTTGGACCAATTGGCTAARACTTTGRCYACTGTTCCTGAATTGAATGAAATTATTGGKCAAGAWTTAGTTGACGAATTTRTCAGTGGTATTAAACTACCAGCAGAAGTTGGAAGTCAAGATGATGTTAACAATAGAAAATTGTTGCAAAAAGTGTTTGGTAAATTAATGAACACTGATGATGACGTTWTAAAACAACAAACAGCTAAATTACTTGAAAGAACAGACAGAGAACCTCAAGTGTTCAAGGATATTGATTCTAGATTACCGGAGTTAATACAAAGATTAAACAAACAATTTCCTAATGACATCGGATTATTTTGTGGATGTCTCTTATTGAACCACGTTGATTGAAAAAATTATCAATTTCCGTGTTGGATAAACAGAGATTGACTGAAAAATTCAATAAATTGGATAAATCCATCAAAGATAATTTGAAGGCTAAACAAAAAGAAGAAACCAAAAAAACTTTAGATGTGGTTAATAATTGGTTGAATGATAAAGAAAATGCTTCATCATTTTTGGTTGCTCACGTTCCAATTACTGCTAATGCCAAGGCAATCACTGAAGCCATTAATTTGATTAAAAAGCAAGATAAAACCAAATCAATTTATTTATTGACTGGTGAAACCGATAAAGTTGCTCATGGATGTTATGTTAGTGATGAAGCCATTGCCAAGGGTATTAATGCGAAAATCAAACCTTTTGAAGCACGAGCAATTAACTGGTCCACGGATCTTAATGCTGAGGTACATATTGAGCATTATATAAATATATTCAATTATGCACGATCATCTTGGGAGCCATTGGTTGAAAGTTGGCCAATAGCAGTTTACATGTCAAAATCCCGACACCCAAAGCCTCAATTATTAGTAGAGGTGATTTCTAGACAGGTAGCYCAAGTGACGCTTACATCCAAAGCAGTAGCATTGCTATCTCAAGTATCCGATTTGATTACTTCCRGAGAAAAATTAAAACCAAGAGGTGAAGATTACCCATACGTKATAGTGAATGAGACTGGTTTAGATTTGGAAGTTTGGAATGATGCAAAWGAATYCGAAACCAATGGAGTTCCAATTGTTTTACGTGCTGGTAAAGCTTTAGATGAAAGTAAAGTTGAAATTAGAATTCAATTTAAACCAGTCGCCAAGGGGATGTTTAAAGAAATTCAAAGAAATGAATTAGTTATTAGAGTACAACCAAATGAAGCCATTTATTTAAAAATTAATTCCAAAATCCCTGGAATTTCTACTGAAACTTCATTAACTGATTTAGATTTAACTTATGCTACTCGTTATTCTAAAGACTTTTGGATTCCTGAAGCTTATGAAGCATTAATTAGAGATTGCTATTTAGGTAATCATTCTAATTTTGTTAGAGATGATGAATTGGATGTTTCTTGGAAATTATTTACTCCATTATTGAATTTATTTAAAAATTAATTCCAAAATCCCTGGAATTTCTACTGAAACTTCATTAACTGATTTAGATTTAACTTATGCTACTCGTTATTCTAAAGACTTTTGGATTCCTGAAGCTTATGAAGCATTAATTAGAGATTGCTATTTAGGTAATCATTCTAATTTTGTTAGAGATGATGAATTGGATGTTTCTTGGAAATTATTTACTCCATTATTGAAT

>Au19

AATTATTGAARTGGAATGATATTCCATTGGCTCCACCAGACAARATTTTGGGTATTTCTGAAGCTTATAACAATGATTCTAACCCTCAAAAARTCAATTTGGGGGTTGGTGCTTATAGAGATAATTCYGGTAAACCAATTATTTTCCCATCAGTTAAAAAAGCTGAAGAAATTTTATTGGGTAAAGAAACTGAAAAGGAATATACTGCCATTGTTGGTTCCAAAAATTTCCAATCAATTGTGAAAAATTTCATTTTCAACAATTCTAATAAAGATGCCAATGGTAAACAATTAATTGATGATGGTAGAATTGTTACTGCTCAAACCATCTCTGGTACTGGATCACTTAAACCTCTTATGGTGAAATTTTTGCTAAACATAGAGAACCAAATTTGGAAATTATTCGTGAGGTTGTTGATTCCAAACATATTGTTTTTGATGTGTTGGCACAATTCTTAATCAATCCAGACCCATGGGTTGCCATTGCTGCCGCTGAAGTTTATGTCAGACGTTCATACCGTGCTTATGATTTGGGTAAAATTGAATATCATGTTAATGACAGACTTCCTATTGTTGAATGGAAATTCAAGTTGGCTAATATGGGAGCCGCTGGTGTAAACGATGCTCAACAGGCTGCTGCTGCYGGTGGCGATGATTCGACATCTATGAAACATGCAGCTTCTGTGTCTGATTTGACCTTTGTTGTTGATTATTCATRAAAGCGAAATCCCAGGATACACTCTCCCCGATAATCCAAAGTTCACCCTTGGTAATTTGTTTGTAATAATTGGAGTCTTGTTGGTTTGTATTTTAGCTGTTGTCTCTCTTTTGAGAAATATTAGTGAGTCGGCCTTGTTCAAGAAGAATGGGTATGAACCGTTGGATTCGGATCCTAGTGTCATGAACCTAAACTTCGAGCCTACAACATTGTCCTTTGAAGATATTAAATATGAGGTTACTGGTGGTCGACAAATTTTAAATGGAGTCTTTGGGTTTGTAAAACCAAGAGAATGTTTGGCTATAATGGGAGGTTCAGGTGCTGGTAAAACTACATTGTTGGATTTTAAACCTTTGGACCAATTGGCTAARACTTTGRCYACTGTTCCTGAATTGAATGAAATTATTGGKCAAGAWTTAGTTGACGAATTTRTCAGTGGTATTAAACTACCAGCAGAAGTTGGAAGTCAAGATGATGTTAACAATAGAAAATTGTTGCAAAAAGTGTTTGGTAAATTAATGAACACTGATGATGACGTTATAAAACAACAAACAGCTAAATTACTTGAAAGAACAGACAGAGAACCTCAAGTGTTCAAGGATATTGATTCTAGATTACCGGAGTTAATACAAAGATTAAACAAACAATTTCCTAATGACATCGGATTATTTTGTGGATGTCTCTTATTGAACCACGTTGATTGAAAAAATTATCAATTTCCGTGTTGGATAAACAGAGATTGACTGAAAAATTCAATAAATTGGATAAATCCATCAAAGATAATTTGAAGGCTAAACAAAAAGAAGAAACCAAAAAAACTTTAGATGTGGTTAATAATTGGTTGAATGATAAAGAAAATGCTTCATCATTTTTGGTTGCTCACGTTCCAATTACTGCTAATGCCAAGGCAATCACTGAAGCCATTAATTTGATTAAAAAGCAAGATAAAACCAAATCAATTTATTTATTGACTGGTGAAACCGATAAAGTTGCTCATGGATGTTATGTTAGTGATGAAGCCATTGCCAAGGGTATTAATGCGAAAATCAAACCTTTTGAAGCACGAGCAATTAACTGGTCCACGGATCTTAATGCTGAGGTACATATTGAGCATTATATAAATATATTCAATTATGCACGATCATCTTGGGAGCCATTGGTTGAAAGTTGGCCAATAGCAGTTTACATGTCAAAATCCCGACACCCAAAGCCTCAATTATTAGTAGAGGTGATTTCTAGACAGGTAGCTCAAGTGACGCTTACATCCAAAGCAGTAGCATTGCTATCTCAAGTATCCGATTTGATTACTTCCRGAGAAAAATTAAAACCAAGAGGTGAAGATTACCCATACGTKATAGTGAATGAGACTGGTTTAGATTTGGAAGTTTGGAATGATGCAAAWGAATYCGAAACCAATGGAGTTCCAATTGTTTTACGTGCTGGTAAAGCTTTAGATGAAAGTAAAGTTGAAATTAGAATTCAATTTAAACCAGTCGCCAAGGGGATGTTTAAAGAAATTCAAAGAAATGAATTAGTTATTAGAGTACAACCAAATGAAGCCATTTATTTAAAAATTAATTCCAAAATCCCTGGAATTTCTACTGAAACTTCATTAACTGATTTAGATTTAACTTATGCTACTCGTTATTCTAAAGAYTTTTGGATTCCTGAAGCTTATGAAGCATTAATTAGAGATTGYTATTTAGGTAATCATTCTAATTTTGTTAGAGATGATGAATTGGATGTTTCTTGGAAATTATTTACTCCATTATTGAAT

>Au90

AATTATTGAARTGGAATGATATTCCATTGGCTCCACCAGACAARATTTTGGGTATTTCTGAAGCTTATAACAATGATTCTAACCCTCAAAAAGTCAATTTGGGGGTTGGTGCTTATAGAGATAATTCTGGTAAACCAATTATTTTCCCATCAGTTAAAAAAGCTGAAGAAATTTTATTGGGTAAAGAAACTGAAAAGGAATATACTGCCATTGTTGGTTCCAAAAATTTCCAATCAATTGTGAAAAATTTCATTTTCAACAATTCTAATAAAGATGCCAATGGTAAACAATTAATTGATGATGGTAGAATTGTTACTGCTCAAACCATCTCTGGTACTGGATCACTTAAACCTCTTATGGTGAAATTTTTGCTAAACATAGAGAACCAAATTTGGAAATTATTCGTGAGGTTGTTGATTCCAAACATATTGTTTTTGATGTGTTGGCACAATTCTTAATCAATCCAGACCCATGGGTTGCCATTGCTGCCGCTGAAGTTTATGTCAGACGTTCATACCGTGCTTATGATTTGGGTAAAATTGAATATCATGTTAATGACAGACTTCCTATTGTTGAATGGAAATTCAAGTTGGCTAATATGGGAGCCGCTGGTGTAAACGATGCTCAACAGGCTGCTGCTGCCGGTGGCGATGATTCGACATCTATGAAACATGCAGCTTCTGTGTCTGATTTGACCTTTGTTGTTGATTATTCATRAAAGCGAAATCCCAGGATACACTCTCCCCGATAATCCAAAGTTCACCCTTGGTAATTTGTTTGTAATAATTGGAGTCTTGTTGGTTTGTATTTTAGCTGTTGTCTCTCTTTTGAGAAATATTAGTGAGTCGGCCTTGTTCAAGAAGAATGGGTATGAACCGTTGGATTCGGATCCTAGTGTCATGAACCTAAACTTCGAGCCTACAACATTGTCCTTTGAAGATATTAAATATGAGGTTACTGGTGGTCGACAAATTTTAAATGGAGTCTTTGGGTTTGTAAAACCAAGAGAATGTTTGGCTATAATGGGAGGTTCAGGTGCTGGTAAAACTACATTGTTGGATTTTAAACCTTTGGACCAATTGGCTAARACTTTGRCYACTGTTCCTGAATTGAATGAAATTATTGGKCAAGAWTTAGTTGACGAATTTRTCAGTGGTATTAAACTACCAGCAGAAGTTGGAAGTCAAGATGATGTTAACAATAGAAAATTGTTGCAAAAAGTGTTTGGTAAATTAATGAACACTGATGATGACGTTATAAAACAACAAACAGCTAAATTACTTGAAAGAACAGACAGAGAACCTCAAGTGTTCAAGGATATTGATTCTAGATTACCGGAGTTAATACAAAGATTAAACAAACAATTTCCTAATGACATCGGATTATTTTGTGGATGTCTCTTATTGAACCACGTTGATTGAAAAAATTATCAATTTCCGTGTTGGATAAACAGAGATTGACTGAAAAATTCAATAAATTGGATAAATCCATCAAAGATAATTTGAAGGCTAAACAAAAAGAAGAAACCAAAAAAACTTTAGATGTGGTTAATAATTGGTTGAATGATAAAGAAAATGCTTCATCATTTTTGGTTGCTCACGTTCCAATTACTGCTAATGCCAAGGCAATCACTGAAGCCATTAATTTGATTAAAAAGCAAGATAAAACCAAATCAATTTATTTATTGACTGGTGAAACCGATAAAGTTGCTCATGGATGTTATGTTAGTGATGAAGCCATTGCCAAGGGTATTAATGCGAAAATCAAACCTTTTGAAGCACGAGCAATTAACTGGTCCACGGATCTTAATGCTGAGGTACATATTGAGCATTATATAAATATATTCAATTATGCACGATCATCTTGGGAGCCATTGGTTGAAAGTTGGCCAATAGCAGTTTACATGTCAAAATCCCGACACCCAAAGCCTCAATTATTAGTAGAGGTGATTTCTAGACAGGTAGCYCAAGTGACGCTTACATCCAAAGCAGTAGCATTGCTATCTCAAGTATCCGATTTGATTACTTCCRGAGAAAAATTAAAACCAAGAGGTGAAGATTACCCATACGTKATAGTGAATGAGACTGGTTTAGATTTGGAAGTTTGGAATGATGCAAAWGAATYCGAAACCAATGGAGTTCCAATTGTTTTACGTGCTGGTAAAGCTTTAGATGAAAGTAAAGTTGAAATTAGAATTCAATTTAAACCAGTCGCCAAGGGGATGTTTAAAGAAATTCAAAGAAATGAATTAGTTATTAGAGTACAACCAAATGAAGCCATTTATTTAAAAATTAATTCCAAAATCCCTGGAATTTCTACTGAAACTTCATTAACTGATTTAGATTTAACTTATGCTACTCGTTATTCTAAAGACTTTTGGATTCCTGAAGCTTATGAAGCATTAATTAGAGATTGCTATTTAGGTAATCATTCTAATTTTGTTAGAGATGATGAATTGGATGTTTCTTGGAAATTATTTACTCCATTATTGAAT

>cfr2.9vag

AATTATTGAARTGGAATGATATTCCATTGGCTCCACCAGACAARATTTTGGGTATTTCTGAAGCTTATAACAATGATTCTAACCCTCAAAAARTCAATTTGGGGGTTGGTGCTTATAGAGATAATTCYGGTAAACCAATTATTTTCCCATCAGTTAAAAAAGCTGAAGAAATTTTATTGGGTAAAGAAACTGAAAAGGAATATACTGCCATTGTTGGTTCCAAAAATTTCCAATCAATTGTGAAAAATTTCATTTTCAACAATTCTAATAAAGATGCCAATGGTAAACAATTAATTGATGATGGTAGAATTGTTACTGCTCAAACCATCTCTGGTACTGGATCACTTAAACCTCTTATGGTGAAATTTTTGCTAAACATAGAGAACCAAATTTGGAAATTATTCGTGAGGTTGTTGATTCCAAACATATTGTTTTTGATGTGTTGGCACAATTCTTAATCAATCCAGACCCATGGGTTGCCATTGCTGCCGCTGAAGTTTATGTCAGACGTTCATACCGTGCTTATGATTTGGGTAAAATTGAATATCATGTTAATGACAGACTTCCTATTGTTGAATGGAAATTCAAGTTGGCTAATATGGGAGCCGCTGGTGTAAACGATGCTCAACAGGCTGCTGCTGCCGGTGGCGATGATTCGACATCTATGAAACATGCAGCTTCTGTGTCTGATTTGACCTTTGTTGTTGATTATTCATAAAAGCGAAATCCCAGGATACACTCTCCCCGATAATCCAAAGTTCACCCTTGGTAATTTGTTTGTAATAATTGGAGTCTTGTTGGTTTGTATTTTAGCTGTTGTCTCTCTTTTGAGAAATATTAGTGAGTCGGCCTTGTTCAAGAAGAATGGGTATGAACCGTTGGATTCGGATCCTAGTGTCATGAACCTAAACTTCGAGCCTACAACATTGTCCTTTGAAGATATTAAATATGAGGTTACTGGTGGTCGACAAATTTTAAATGGAGTCTTTGGGTTTGTAAAACCAAGAGAATGTTTGGCTATAATGGGAGGTTCAGGTGCTGGTAAAACTACATTGTTGGATTTTAAACCTTTGGACCAATTGGCTAARACTTTGRCYACTGTTCCTGAATTGAATGAAATTATTGGKCAAGAWTTAGTTGACGAATTTRTCAGTGGTATTAAACTACCAGCAGAAGTTGGAAGTCAAGATGATGTTAACAATAGAAAATTGTTGCAAAAAGTGTTTGGTAAATTAATGAACACTGATGATGACGTTATAAAACAACAAACAGCTAAATTACTTGAAAGAACAGACAGAGAACCTCAAGTGTTCAAGGATATTGATTCTAGATTACCGGAGTTAATACAAAGATTAAACAAACAATTTCCTAATGACATCGGATTATTTTGTGGATGTCTCTTATTGAACCACGTTGATTGAAAAAATTATCAATTTCCGTGTTGGATAAACAGAGATTGACTGAAAAATTCAATAAATTGGATAAATCCATCAAAGATAATTTGAAGGCTAAACAAAAAGAAGAAACCAAAAAAACTTTAGATGTGGTTAATAATTGGTTGAATGATAAAGAAAATGCTTCATCATTTTTGGTTGCTCACGTTCCAATTACTGCTAATGCCAAGGCAATCACTGAAGCCATTAATTTGATTAAAAAGCAAGATAAAACCAAATCAATTTATTTATTGACTGGTGAAACCGATAAAGTTGCTCATGGATGTTATGTTAGTGATGAAGCCATTGCCAAGGGTATTAATGCGAAAATCAAACCTTTTGAAGCACGAGCAATTAACTGGTCCACGGATCTTAATGCTGAGGTACATATTGAGCATTATATAAATATATTCAATTATGCACGATCATCTTGGGAGCCATTGGTTGAAAGTTGGCCAATAGCAGTTTACATGTCAAAATCCCGACACCCAAAGCCTCAATTATTAGTAGAGGTGATTTCTAGACAGGTAGCYCAAGTGACGCTTACATCCAAAGCAGTAGCATTGCTATCTCAAGTATCCGATTTGATTACTTCCRGAGAAAAATTAAAACCAAGAGGTGAAGATTACCCATACRTKATAGTGAATGAGACTGGTTTAGATTTGGAAGTTTGGAATGATGCAAAWGAATYCGAAACCAATGGAGTTCCAATTGTTTTACGTGCTGGTAAAGCTTTAGATGAAAGTAAAGTTGAAATTAGAATTCAATTTAAACCAGTCGCCAAGGGGATGTTTAAAGAAATTCAAAGAAATGAATTAGTTATTAGAGTACAACCAAATGAAGCCATTTATTTAAAAATTAATTCCAAAATCCCTGGAATTTCTACTGAAACTTCATTAACTGATTTAGATTTAACTTATGCTACTCGTTATTCTAAAGAYTTTTGGATTCCTGAAGCTTATGAAGCATTAATTAGAGATTGYTATTTAGGTAATCATTCTAATTTTGTTAGAGATGATGAATTGGATGTTTCTTGGAAATTATTTACTCCATTATTGAAT

>CH3

AATTATTGAAATGGAATGATATTCCATTGGCYCCACCAGACAAGATTTTGGGTATTTCTGAAGCTTATAACAATGATTCTAACCCTCAAAAARTCAATTTGGGGGTTGGTGCTTATAGAGATAATTCTGGTAAACCAATTATTTTCCCATCAGTTAAAAAAGCTGAAGAAATTTTATTGGGTAAAGAAACTGAAAAGGAATATACTGCCATTGTTGGTTCCAAAAATTTCCAATCAATTGTGAAAAATTTCATTTTCAACAATTCTAATAAAGATGCCAATGGTAAACAATTAATTGATGATGGTAGAATTGTTACTGCTCAAACCATYTCTGGTACTGGATCACTTAAACCTCTTATGGTGAAATTTTTGCTAAACATAGAGAACCAAATTTGGAAATTATTCGTGAGGTTGTTGATTCCAAACATATTGTTTTTGATGTGTTGGCACAATTCTTAATCAATCCAGACCCATGGGTTGCCATTGCTGCCGCTGAAGTTTATGTCAGACGTTCATACCGTGCTTATGATTTGGGTAAAATTGAATATCATGTTAATGACAGACTTCCTATTGTTGAATGGAAATTCAAGTTGGCTAATATGGGAGCYGCTGGTGTAAACGATGCTCAACAGGCTGCTGCTGCCGGTGGCGATGATTCGACATCTATGAAACATGCAGCTTCTGTGTCTGATTTGACCTTTGTTGTTGATTATTCATAAAAGCGAAATCCCAGGATACACTCTCCCCGATAATCCAAAGTTCACCCTTGGTAATTTGTTTGTAATAATTGGAGTCTTGTTGGTTTGTATTTTAGCTGTTGTCTCTCTTTTGAGAAATATTAGTGAGTCGGCCTTGTTCAAGAAGAATGGGTATGAACCGTTGGATTCGGATCCTAGTRTCATGAACCTAAACTTCGAGCCTACAACATTGTCCTTTGAAGATATTAAATATGAGGTTACTGGTGGTCGACAAATTTTAAATGGAGTCTTTGGGTTTGTAAAACCAAGAGAATGTTTGGCTATAATGGGAGGTTCAGGTGCTGGTAAAACTACATTGTTGGATTTTAAACCTTTGGACCAATTGGCTAAGACTTTGACCACTGTTCCTGAATTGAATGAAATTATTGGGCAAGATTTAGTTGACGAATTTGTCAGTGGTATTAAACTACCAGCAGAAGTTGGAAGTCAAGATGATGTTAACAATAGAAAATTGTTGCAAAAAGTGTTTGGTAAATTAATGAACACTGATGATGACGTTATAAAACAACAAACAGCTAAATTACTTGAAAGAACAGACAGAGAACCTCAAGTGTTCAAGGATATTGATTCTAGATTACCGGAGTTAATACAAAGATTAAACAAACAATTTCCTAATGACATCGGATTATTTTGTGGATGTCTCTTATTGAACCACGTTGCTTGAAAAAATTATCAATTTCCGTGTTGGATAAACAAAGATTGACTGAAAAATTCAATAAATTGGATAAATCCATTAAAGATAATTTGAAGGCTAAACAAAAAGAAGAAACCAAAAAGACTTTAGATGTGGTTAACAATTGGTTGAATGATAAAGAAAATGCTTCATCATTTTTGGTTGCTCACGTTCCAATTACTGCTAATGCCAAGGCAATCACTGAAGCCATTAATTTGATTAAAAAGCAAGATAAAACCAAATCAATTTATTTATTGACTGGTGAAACCGATAAAGTTGCTCATGGATGTTATGTTAGTGATGAAGCCATTGTCAAGGGTATTAATGCGAAAATCAAACCTTTTGAAGCACGAGCAATTAACTGGTCCACGGATCTTAATGCTGAGGTACATATTGAGCATTATATAAATATATTCAATTATGCACGATCATCTTGGGAGCCATTGGTTGAAWGTTGGCCAATAGCAGTTTACATGTCAAAATCCCGACACCCAAAGCCTCAATTATTAGTAGAGGTGATTTCTAGACAGGTAGCTCAAGTGACGCTTACATCCAAAGCAGTAGCATTGCTATCTCAAGTATCCGATTTGATTACTTCCAGAGAAAAATTAAAACCAAGAGGTGAAGATTACCCATACGTTATAGTGAATGAGACTGGTTTAGATTTGGAAGTTTGGAATGATGCAAATGAATCCGAAACCAATGGAGTTCCAATTGTTTTACGTGCTGGTAAAGCTTTAGATGAAAGTAAAGTTGAAATTAGAATTCAATTTAAACCAGTCGCCAAGGGGATGTTTAAAGAAATTCAAAGAAATGAATTAGTTATTAGAGTACAACCAAATGAAGCCATTTATTTAAAAATTAATTCCAAAATCCCTGGAATTTCTACTGAAACTTCATTAACTGATTTAGATTTAACTTATGCTACTCGTTATTCTAAAGACTTTTGGATTCCTGAAGCTTATGAAGCATTAATTAGAGATTGTTATTTAGGTAATCATTCTAATTTTGTTAGAGATGATGAATTGGATGTTTCTTGGAAATTATTTACTCCATTATTGAAT

>CH35

AATTATTGAARTGGAATGATATTCCATTGGCTCCACCAGACAARATTTTGGGTATTTCTGAAGCTTATAACAATGATTCTAACCCTCAAAAAGTCAATTTGGGGGTTGGTGCTTATAGAGATAATTCTGGTAAACCAATTATTTTCCCATCAGTTAAAAAAGCTGAAGAAATTTTATTGGGTAAAGAAACTGAAAAGGAATATACTGCCATTGTTGGTTCCAAAAATTTCCAATCAATTGTGAAAAATTTCATTTTCAACAATTCTAATAAAGATGCCAATGGTAAACAATTAATTGATGATGGTAGAATTGTTACTGCTCAAACCATCTCTGGTACTGGATCACTTAAACCTCTTATGGTGAAATTTTTGCTAAACATAGAGAACCAAATTTGGAAATTATTCGTGAGGTTGTTGATTCCAAACATATTGTTTTTGATGTGTTGGCACAATTCTTAATCAATCCAGACCCATGGGTTGCCATTGCTGCCGCTGAAGTTTATGTCAGACGTTCATACCGTGCTTATGATTTGGGTAMAATTGAATATCATGTTAATGACAGACTTCCTATTGTTGAATGGAAATTCAAGTTGGCTAATATGGGAGCCGCTGGTGTAAACGATGCTCAACAGGCTGCTGCTGCYGGTGGCGATGATTCGACATCTATGAAACATGCAGCTTCTGTGTCTGATTTGACCTTTGTTGTTGATTATTCATRAAAGCGAAATCCCAGGATACACTCTCCCCGATAATCCAAAGTTCACCCTTGGTAATTTGTTTGTAATAATTGGAGTCTTGTTGGTTTGTATTTTAGCTGTTGTCTCTCTTTTGAGAAATATTAGTGAGTCGGCCTTGTTCAAGAAGAATGGGTATGAACCGTTGGATTCGGATCCTAGTGTCATGAACCTAAACTTCGAGCCTACAACATTGTCCTTTGAAGATATTAAATATGAGGTTACTGGTGGTCGACAAATTTTAAATGGAGTCTTTGGGTTTGTAAAACCAAGAGAATGTTTGGCTATAATGGGAGGTTCAGGTGCTGGTAAAACTACATTGTTGGATTTTAAACCTTTGGACCAATTGGCTAARACTTTGRCYACTGTTCCTGAATTGAATGAAATTATTGGKCAAGAWTTAGTTGACGAATTTRTCAGTGGTATTAAACTACCAGCAGAAGTTGGAAGTCAAGATGATGTTAACAATAGAAAATTGTTGCAAAAAGTGTTTGGTAAATTAATGAACACTGATGATGACGTTATAAAACAACAAACAGCTAAATTACTTGAAAGAACAGACAGAGAACCTCAAGTGTTCAAGGATATTGATTCTAGATTACCGGAGTTAATACAAAGATTAAACAAACAATTTCCTAATGACATCGGATTATTTTGTGGATGTCTCTTATTGAACCACGTTGATTGAAAAAATTATCAATTTCCGTGTTGGATAAACAGAGATTGACTGAAAAATTCAATAAATTGGATAAATCCATCAAAGATAATTTGAAGGCTAAACAAAAAGAAGAAACCAAAAAAACTTTAGATGTGGTTAATAATTGGTTGAATGATAAAGAAAATGCTTCATCATTTTTGGTTGCTCACGTTCCAATTACTGCTAATGCCAAGGCAATCACTGAAGCCATTAATTTGATTAAAAAGCAAGATAAAACCAAATCAATTTATTTATTGACTGGTGAAACCGATAAAGTTGCTCATGGATGTTATGTTAGTGATGAAGCCATTGCCAAGGGTATTAATGCGAAAATCAAACCTTTTGAAGCACGAGCAATTAACTGGTCCACGGATCTTAATGCTGAGGTACATATTGAGCATTATATAAATATATTCAATTATGCACGATCATCTTGGGAGCCATTGGTTGAAAGTTGGCCAATAGCAGTTTACATGTCAAAATCCCGACACCCAAAGCCTCAATTATTAGTAGAGGTGATTTCTAGACAGGTAGCTCAAGTGACGCTTACATCCAAAGCAGTAGCATTGCTATCTCAAKTATCCGATTTGATTACTTCCRGAGAAAAATTAAAACCAAGAGGTGAAGATTACCCATACGTKATAGTGAATGAGACTGGTTTAGATTTGGAAGTTTGGAATGATGCAAAWGAATYCGAAACCAATGGAGTTCCAATTGTTTTACGTGCTGGTAAAGCTTTAGATGAAAGTAAAGTTGAAATTAGAATTCAATTTAAACCAGTCGCCAAGGGGATGTTTAAAGAAATTCAAAGAAATGAATTAGTTATTAGAGTACAACCAAATGAAGCCATTTATTTAAAAATTAATTCCAAAATCCCTGGAATTTCTACTGAAACTTCATTAACTGATTTAGATTTAACTTATGCTACTCGTTATTCTAAAGAYTTTTGGATTCCTGAAGCTTATGAAGCATTAATTAGAGATTGYTATTTAGGTAATCATTCTAATTTTGTTAGAGATGATGAATTGGATGTTTCTTGGAAATTATTTACTCCATTATTGAAT

>CLB42

AATTATTGAARTGGAATGATATTCCATTGGCTCCACCAGACAARATTTTGGGTATTTCTGAAGCTTATAACAATGATTCTAACCCTCAAAAARTCAATTTGGGGGTTGGTGCTTATAGAGATAATTCYGGTAAACCAATTATTTTCCCATCAGTTAAAAAAGCTGAAGAAATTTTATTGGGTAAAGAAACTGAAAAGGAATATACTGCCATTGTTGGTTCCAAAAATTTCCAATCAATTGTGAAAAATTTCATTTTCAACAATTCTAATAAAGATGCCAATGGTAAACAATTAATTGATGATGGTAGAATTGTTACTGCTCAAACCATCTCTGGTACTGGATCACTTAAACCTCTTATGGTGAAATTTTTGCTAAACATAGAGAACCAAATTTGGAAATTATTCGTGAGGTTGTTGATTCCAAACATATTGTTTTTGATGTGTTGGCACAATTCTTAATCAATCCAGACCCATGGGTTGCCATTGCTGCCGCTGAAGTTTATGTCAGACGTTCATACCGTGCTTATGATTTGGGTAAAATTGAATATCATGTTAATGACAGACTTCCTATTGTTGAATGGAAATTCAAGTTGGCTAATATGGGAGCCGCTGGTGTAAACGATGCTCAACAGGCTGCTGCTGCCGGTGGCGATGATTCGACATCTATGAAACATGCAGCTTCTGTGTCTGATTTGACCTTTGTTGTTGATTATTCATGAAAGCGAAATCCCAGGATACACTCTCCCCGATAATCCAAAGTTCACCCTTGGTAATTTGTTTGTAATAATTGGAGTCTTGTTGGTTTGTATTTTAGCTGTTGTCTCTCTTTTGAGAAATATTAGTGAGTCGGCCTTGTTCAAGAAGAATGGGTATGAACCGTTGGATTCGGATCCTAGTGTCATGAACCTAAACTTCGAGCCTACAACATTGTCCTTTGAAGATATTAAATATGAGGTTACTGGTGGTCGACAAATTTTAAATGGAGTCTTTGGGTTTGTAAAACCAAGAGAATGTTTGGCTATAATGGGAGGTTCAGGTGCTGGTAAAACTACATTGTTGGATTTTAAACCTTTGGACCAATTGGCTAARACTTTGRCYACTGTTCCTGAATTGAATGAAATTATTGGKCAAGAWTTAGTTGACGAATTTRTCAGTGGTATTAAACTACCAGCAGAAGTTGGAAGTCAAGATGATGTTAACAATAGAAAATTGTTGCAAAAAGTGTTTGGTAAATTAATGAACACTGATGATGACGTTATAAAACAACAAACAGCTAAATTACTTGAAAGAACAGACAGAGAACCTCAAGTGTTCAAGGATATTGATTCTAGATTACCGGAGTTAATACAAAGATTAAACAAACAATTTCCTAATGACATCGGATTATTTTGTGGATGTCTCTTATTGAACCACGTTGATTGAAAAAATTATCAATTTCCGTGTTGGATAAACAGAGATTGACTGAAAAATTCAATAAATTGGATAAATCCATCAAAGATAATTTGAAGGCTAAACAAAAAGAAGAAACCAAAAAAACTTTAGATGTGGTTAATAATTGGTTGAATGATAAAGAAAATGCTTCATCATTTTTGGTTGCTCACGTTCCAATTACTGCTAATGCCAAGGCAATCACTGAAGCCATTAATTTGATTAAAAAGCAAGATAAAACCAAATCAATTTATTTATTGACTGGTGAAACCGATAAAGTTGCTCATGGATGTTATGTTAGTGATGAAGCCATTGCCAAGGGTATTAATGCGAAAATCAAACCTTTTGAAGCACGAGCAATTAACTGGTCCACGGATCTTAATGCTGAGGTACATATTGAGCATTATATAAATATATTCAATTATGCACGATCATCTTGGGAGCCATTGGTTGAAAGTTGGCCAATAGCAGTTTACATGTCAAAATCCCGACACCCAAAGCCTCAATTATTAGTAGAGGTGATTTCTAGACAGGTAGCYCAAGTGACGCTTACATCCAAAGCAGTAGCATTGCTATCTCAAGTATCCGATTTGATTACTTCCRGAGAAAAATTAAAACCAAGAGGTGAAGATTACCCATACGTKATAGTGAATGAGACTGGTTTAGATTTGGAAGTTTGGAATGATGCAAAWGAATYCGAAACCAATGGAGTTCCAATTGTTTTACGTGCTGGTAAAGCTTTAGATGAAAGTAAAGTTGAAATTAGAATTCAATTTAAACCAGTCGCCAAGGGGATGTTTAAAGAAATTCAAAGAAATGAATTAGTTATTAGAGTACAACCAAATGAAGCCATTTATTTAAAAATTAATTCCAAAATCCCTGGAATTTCTACTGAAACTTCATTAACTGATTTAGATTTAACTTATGCTACTCGTTATTCTAAAGAYTTTTGGATTCCTGAAGCTTATGAAGCATTAATTAGAGATTGYTATTTAGGTAATCATTCTAATTTTGTTAGAGATGATGAATTGGATGTTTCTTGGAAATTATTTACTCCATTATTGAAT

>CLB44

AATTATTGAAATGGAATGATATTCCATTGGCTCCACCAGACAAAATTTTGGGTATTTCTGAAGCTTATAACAATGATTCTAACCCTCAAAAAATCAATTTGGGGGTTGGTGCTTATAGAGATAATTCCGGTAAACCAATTATTTTCCCATCAGTTAAAAAAGCTGAAGAAATTTTATTGGGTAAAGAAACTGAAAAGGAATATACTGCCATTGTTGGTTCCAAAAATTTCCAATCAATTGTGAAAAATTTCATTTTCAACAATTCTAATAAAGATGCCAATGGTAAACAATTAATTGATGATGGTAGAATTGTTACTGCTCAAACCATCTCTGGTACTGGATCACTTAAACCTCTTATGGTGAAATTTTTGCTAAACATAGAGAACCAAATTTGGAAATTATTCGTGAGGTTGTTGATTCCAAACATATTGTTTTTGATGTGTTGGCACAATTCTTAATCAATCCAGACCCATGGGTTGCCATTGCTGCCGCTGAAGTTTATGTCAGACGTTCATACCGTGCTTATGATTTGGGTAAAATTGAATATCATGTTAATGACAGACTTCCTATTGTTGAATGGAAATTCAAGTTGGCTAATATGGGAGCYGCTGGTGTAAACGATGCTCAACAGGCTGCTGCTGCCGGTGGCGATGATTCGACATCTATGAAACATGCAGCTTCTGTGTCTGATTTGACCTTTGTTGTTGATTATTCATAAAAGYGAAATYCCAGGATACACTCTCCCCGATAATCCAAAGTTCACCCTTGGTAATTTGTTTGTAATAATTGGRGTCTTGTTGGTTTGTGTTTTAGCTGTTGTCTCTCTTTTGAGAAATATTAGTGAGTCRGCCTTGTTCAAGAAGAATGGGTATGAACCGTTGGATTCRGATCCTAGTGTCATGAACCWAAACTTCGAGCCTACAACATTGTCCTTTGAAGATATTAAATATGAGGTTACTGGTGGTCGACAAATTTTAAATGGAGTCTTTGGGTTTGTAAAACCAAGAGAATGTTTGGCTATAATGGGAGGTTCAGGTGCTGGTAAAACTACATTGTTGGATTTTAAACCTTTGGACCAATTGGCTAAGACTTTGACCACTGTTCCTGAATTGAATGAAATTATTGGGCAAGATTTAGTTGACGAATTTGTCAGTGGTATTAAACTACCAGCAGAAGTTGGAAGTCAAGATGATGTTAACAATAGAAAATTGTTGCAAAAAGTGTTTGGTAAATTAATGAACACTGATGATGACGTTATAAAACAACAAACAGCTAAATTACTTGAAAGAACAGAMAGRGAACCTCAAGTGTTCAAGGATATTGATTCTAGATTACCRGAGTTAATACAARGATTAAACAAACAATTTCCTAATGACATCGGATTATTTTGTGGATGTCTCTTATTGAACCACGTTGMTTGAAAAAATTATCAATTTCCGTGTTGGATAAACARAGATTGACTGAAAAATTCAATAAATTGGATAAATCCATYAAAGATAATTTGAAGGCTAAACAAAAAGAAGAAACCAAAAAAACTTTAGATGTGGTTAAYAATTGGTTGAATGATAAAGAAAATGCTTCATCATTTTTGGTTGCTCACGTTCCAATTACTGCTAATGCCAAGGCAATCACTGAAGCCATTAATTTGATTAAAAAGCAAGATAAAACCAAATCAATTTATTTATTGACTGGTGAAACCGATAAAGTTGCTCATGGATGTTATGTTAGTGATGAAGCCATTGYCAAGGGTATTAATGCGAAAATCAAACCTTTTGAAGCACGAGCAATTAACTGGTCMACGGATCTTAATGCTGAGGTACATATTGAGCATTATATAAATATATTCAATTATGCACGATCATCTTGGGAGCCATTGGTTGAAAGTTGGCCAATAGCAGTTTACATGTCAAAATCCCGACACCCAAAGCCTCAATTATTAGTAGAGGTGATTTCTAGACAGGTAGCTCAAGTGACGCTTACATCCAAAGCAGTAGCATTGCTATCTCAAGTATCCGATTTGATTACTTCCGGAGAAAAATTAAAACCAAGAGGTGAAGATTACCCATACRTTATAGTGAATGAGACTGGTTTAGATTTGGAAGTTTGGAATGATGCAAAWGAATCCGAAACCAATGGAGTTCCAATTGTTTTACGTGCTGGTAAAGCTTTAGATGAAAGTAAAGTTGAAATTAGAATTCAATTTAAACCAGTCGCCAAGGGGATGTTTAAAGAAATTCAAAGAAATGAATTAGTTATTAGAGTACAACCAAATGAAGCCATTTATTTAAAAATTAATTCCAAAATCCCTGGAATTTCTACTGAAACTTCATTAACTGATTTAGATTTAACTTATGCTACTCGTTATTCTAAAGAYTTTTGGATTCCTGAAGCTTATGAAGCATTAATTAGAGATTGTTATTTAGGTAATCATTCTAATTTTGTTAGAGATGATGAATTGGATGTTTCTTGGAAATTATTTACTCCATTATTGAAT

>cpr2.2fec

AATTATTGAARTGGAATGATATTCCATTGGCTCCACCAGACAARATTTTGGGTATTTCTGAAGCTTATAACAATGATTCTAACCCTCAAAAARTCAATTTGGGGGTTGGTGCTTATAGAGATAATTCYGGTAAACCAATTATTTTCCCATCAGTTAAAAAAGCTGAAGAAATTTTATTGGGTAAAGAAACTGAAAAGGAATATACTGCCATTGTTGGTTCCAAAAATTTCCAATCAATTGTGAAAAATTTCATTTTCAACAATTCTAATAAAGATGCCAATGGTAAACAATTAATTGATGATGGTAGAATTGTTACTGCTCAAACCATCTCTGGTACTGGATCACTTAAACCTCTTATGGTGAAATTTTTGCTAAACATAGAGAACCAAATTTGGAAATTATTCGTGAGGTTGTTGATTCCAAACATATTGTTTTTGATGTGTTGGCACAATTCTTAATCAATCCAGACCCATGGGTTGCCATTGCTGCCGCTGAAGTTTATGTCAGACGTTCATACCGTGCTTATGATTTGGGTAAAATTGAATATCATGTTAATGACAGACTTCCTATTGTTGAATGGAAATTCAAGTTGGCTAATATGGGAGCCGCTGGTGTAAACGATGCTCAACAGGCTGCTGCTGCCGGTGGCGATGATTCGACATCTATGAAACATGCAGCTTCTGTGTCTGATTTGACCTTTGTTGTTGATTATTCATRAAAGCGAAATCCCAGGATACACTCTCCCCGATAATCCAAAGTTCACCCTTGGTAATTTGTTTGTAATAATTGGAGTCTTGTTGGTTTGTATTTTAGCTGTTGTCTCTCTTTTGAGAAATATTAGTGAGTCGGCCTTGTTCAAGAAGAATGGGTATGAACCGTTGGATTCGGATCCTAGTGTCATGAACCTAAACTTCGAGCCTACAACATTGTCCTTTGAAGATATTAAATATGAGGTTACTGGTGGTCGACAAATTTTAAATGGAGTCTTTGGGTTTGTAAAACCAAGAGAATGTTTGGCTATAATGGGAGGTTCAGGTGCTGGTAAAACTACATTGTTGGATTTTAAACCTTTGGACCAATTGGCTAARACTTTGRCYACTGTTCCTGAATTGAATGAAATTATTGGKCAAGAWTTAGTTGACGAATTTRTCAGTGGTATTAAACTACCAGCAGAAGTTGGAAGTCAAGATGATGTTAACAATAGAAAATTGTTGCAAAAAGTGTTTGGTAAATTAATGAACACTGATGATGACGTTATAAAACAACAAACAGCTAAATTACTTGAAAGAACAGACAGAGAACCTCAAGTGTTCAAGGATATTGATTCTAGATTACCGGAGTTAATACAAAGATTAAACAAACAATTTCCTAATGACATCGGATTATTTTGTGGATGTCTCTTATTGAACCACGTTGATTGAAAAAATTATCAATTTCCGTGTTGGATAAACAGAGATTGACTGAAAAATTCAATAAATTGGATAAATCCATCAAAGATAATTTGAAGGCTAAACAAAAAGAAGAAACCAAAAAAACTTTAGATGTGGTTAATAATTGGTTGAATGATAAAGAAAATGCTTCATCATTTTTGGTTGCTCACGTTCCAATTACTGCTAATGCCAAGGCAATCACTGAAGCCATTAATTTGATTAAAAAGCAAGATAAAACCAAATCAATTTATTTATTGACTGGTGAAACCGATAAAGTTGCTCATGGATGTTATGTTAGTGATGAAGCCATTGCCAAGGGTATTAATGCGAAAATCAAACCTTTTGAAGCACGAGCAATTAACTGGTCCACGGATCTTAATGCTGAGGTACATATTGAGCATTATATAAATATATTCAATTATGCACGATCATCTTGGGAGCCATTGGTTGAAAGTTGGCCAATAGCAGTTTACATGTCAAAATCCCGACACCCAAAGCCTCAATTATTAGTAGAGGTGATTTCTAGACAGGTAGCCCAAGTGACGCTTACATCCAAAGCAGTAGCATTGCTATCTCAAGTATCCGATTTGATTACTTCCGGAGAAAAATTAAAACCAAGAGGTGAAGATTACCCATACGTTATAGTGAATGAGACTGGTTTAGATTTGGAAGTTTGGAATGATGCAAAAGAATCCGAAACCAATGGAGTTCCAATTGTTTTACGTGCTGGTAAAGCTTTAGATGAAAGTAAAGTTGAAATTAGAATTCAATTTAAACCAGTCGCCAAGGGGATGTTTAAAGAAATTCAAAGAAATGAATTAGTTATTAGAGTACAACCAAATGAAGCCATTTATTTAAAAATTAATTCCAAAATCCCTGGAATTTCTACTGAAACTTCATTAACTGATTTAGATTTAACTTATGCTACTCGTTATTCTAAAGAYTTTTGGATTCCTGAAGCTTATGAAGCATTAATTAGAGATTGYTATTTAGGTAATCATTCTAATTTTGTTAGAGATGATGAATTGGATGTTTCTTGGAAATTATTTACTCCATTATTGAAT

>Di5_feces_21/1/14

AATTATTGAARTGGAATGATATTCCATTGGCTCCACCAGACAAAATTTTGGGTATTTCTGAAGCTTATAACAATGATTCTAACCCTCAAAAAATCAATTTGGGGGTTGGTGCTTATAGAGATAATTCCGGTAAACCAATTATTTTCCCATCAGTTAAAAAAGCTGAAGAAATTTTATTGGGTAAAGAAACTGAAAAGGAATATACTGCCATTGTTGGTTCCAAAAATTTCCAATCAATTGTGAAAAATTTCATTTTCAACAATTCTAATAAAGATGCCAATGGTAAACAATTAATTGATGATGGTAGAATTGTTACTGCTCAAACCATCTCTGGTACTGGATCACTTAAACCTCTTATGGTGAAATTTTTGCTAAACATAGAGAACCAAATTTGGAAATTATTCGTGAGGTTGTTGATTCCAAACATATTGTTTTTGATGTGTTGGCACAATTCTTAATCAATCCAGACCCATGGGTTGCCATTGCTGCCGCTGAAGTTTATGTCAGACGTTCATACCGTGCTTATGATTTGGGTAAAATTGAATATCATGTTAATGACAGACTTCCTATTGTTGAATGGAAATTCAAGTTGGCTAATATGGGAGCCGCTGGTGTAAACGATGCTCAACAGGCTGCTGCTGCCGGTGGCGATGATTCGACATCTATGAAACATGCAGCTTCTGTGTCTGATTTGACCTTTGTTGTTGATTATTCATAAAAGYGAAATYCCAGGATACACTCTCCCCGATAATCCAAAGTTCACCCTTGGTAATTTGTTTGTAATAATTGGRGTCTTGTTGGTTTGTRTTTTAGCTGTTGTCTCTCTTTTGAGAAATATTAGTGAGTCRGCCTTGTTCAAGAAGAATGGGTATGAACCGTTGGATTCRGATCCTAGTGTCATGAACCWAAACTTYGAGCCTACAACATTGTCCTTTGAAGATATTAAATATGAGGTTACTGGTGGTCGACAAATTTTAAATGGAGTCTTTGGGTTTGTAAAACCAAGAGAATGTTTGGCTATAATGGGAGGTTCAGGTGCTGGTAAAACTACATTGTTGGATTTTAAACCTTTGGACCAATTGGCTAARACTTTGRCYACTGTTCCTGAATTGAATGAAATTATTGGKCAAGAWTTAGTTGACGAATTTRTCAGTGGTATTAAACTACCAGCAGAAGTTGGAAGTCAAGATGATGTTAACAATAGAAAATTGTTGCAAAAAGTGTTTGGTAAATTAATGAACACTGATGATGACGTTATAAAACAACAAACAGCTAAATTACTTGAAAGAACAGACAGAGAACCTCAAGTGTTCAAGGATATTGATTCTAGATTACCGGAGTTAATACAAAGATTAAACAAACAATTTCCTAATGACATCGGATTATTTTGTGGATGTCTCTTATTGAACCACGTTGMTTGAAAAAATTATCAATTTCCGTGTTGGATAAACARAGATTGACTGAAAAATTCAATAAATTGGATAAATCCATTAAAGATAATTTGAAGGCTAAACAAAAAGAAGAAACCAAAAAGACTTTAGATGTGGTTAACAATTGGTTGAATGATAAAGAAAATGCTTCATCATTTTTGGTTGCTCACGTTCCAATTACTGCTAATGCCAAGGCAATCACTGAAGCCATTAATTTGATTAAAAAGCAAGATAAAACCAAATCAATTTATTTATTGACTGGTGAAACMGATAAAGTTGCTCATGGATGTTATGTTAGTGATGAAGCCATTGCCAAGGGTATTAATGCGAAAATCAAACCTTTTGAAGCACGAGCAATTAACTGGTCMACGGATCTTAATGCTGAGGTACATATTGAGCATTATATAAATATATTCAATTATGCACGATCATCTTGGGAGCCATTGGTTGAAAGTTGGCCAATAGCAGTTTACATGTCAAAATCCCGACACCCAAAGCCTCAATTATTAGTAGAGGTGATTTCTAGACAGGTAGCTCAAGTGACGCTTACATCCAAAGCAGTAGCATTGCTATCTCAAGTATCCGATTTGATTACTTCCRGAGAAAAATTAAAACCAAGAGGTGAAGATTACCCATACRTKATAGTGAATGAGACTGGTTTAGATTTGGAAGTTTGGAATGATGCAAAWGAATYCGAAACCAATGGAGTTCCAATTGTTTTACGTGCTGGTAAAGCTTTAGATGAAAGTAAAGTTGAAATTAGAATTCAATTTAAACCAGTCGCCAAGGGGATGTTTAAAGAAATTCAAAGAAATGAATTAGTTATTAGAGTACAACCAAATGAAGCCATTTATTTAAAAATTAATTCCAAAATCCCTGGAATTTCTACTGAAACTTCATTAACTGATTTAGATTTAACTTATGCTACTCGTTATTCTAAAGATTTTTGGATTCCTGAAGCTTATGAAGCATTAATTAGAGATTGTTATTTAGGTAATCATTCTAATTTTGTTAGAGATGATGAATTGGATGTTTCTTGGAAATTATTTACTCCATTATTGAAT

>Di5_groin_21/1/15

AATTATTGAARTGGAATGATATTCCATTGGCTCCACCAGACAAAATTTTGGGTATTTCTGAAGCTTATAACAATGATTCTAACCCTCAAAAAATCAATTTGGGGGTTGGTGCTTATAGAGATAATTCCGGTAAACCAATTATTTTCCCATCAGTTAAAAAAGCTGAAGAAATTTTATTGGGTAAAGAAACTGAAAAGGAATATACTGCCATTGTTGGTTCCAAAAATTTCCAATCAATTGTGAAAAATTTCATTTTCAACAATTCTAATAAAGATGCCAATGGTAAACAATTAATTGATGATGGTAGAATTGTTACTGCTCAAACCATCTCTGGTACTGGATCACTTAAACCTCTTATGGTGAAATTTTTGCTAAACATAGAGAACCAAATTTGGAAATTATTCGTGAGGTTGTTGATTCCAAACATATTGTTTTTGATGTGTTGGCACAATTCTTAATCAATCCAGACCCATGGGTTGCCATTGCTGCCGCTGAAGTTTATGTCAGACGTTCATACCGTGCTTATGATTTGGGTAAAATTGAATATCATGTTAATGACAGACTTCCTATTGTTGAATGGAAATTCAAGTTGGCTAATATGGGAGCCGCTGGTGTAAACGATGCTCAACAGGCTGCTGCTGCCGGTGGCGATGATTCGACATCTATGAAACATGCAGCTTCTGTGTCTGATTTGACCTTTGTTGTTGATTATTCATAAAAGYGAAATYCCAGGATACACTCTCCCCGATAATCCAAAGTTCACCCTTGGTAATTTGTTTGTAATAATTGGRGTCTTGTTGGTTTGTRTTTTAGCTGTTGTCTCTCTTTTGAGAAATATTAGTGAGTCRGCCTTGTTCAAGAAGAATGGGTATGAACCGTTGGATTCRGATCCTAGTGTCATGAACCWAAACTTYGAGCCTACAACATTGTCCTTTGAAGATATTAAATATGAGGTTACTGGTGGTCGACAAATTTTAAATGGAGTCTTTGGGTTTGTAAAACCAAGAGAATGTTTGGCTATAATGGGAGGTTCAGGTGCTGGTAAAACTACATTGTTGGATTTTAAACCTTTGGACCAATTGGCTAARACTTTGRCYACTGTTCCTGAATTGAATGAAATTATTGGKCAAGAWTTAGTTGACGAATTTRTCAGTGGTATTAAACTACCAGCAGAAGTTGGAAGTCAAGATGATGTTAACAATAGAAAATTGTTGCAAAAAGTGTTTGGTAAATTAATGAACACTGATGATGACGTTATAAAACAACAAACAGCTAAATTACTTGAAAGAACAGACAGAGAACCTCAAGTGTTCAAGGATATTGATTCTAGATTACCGGAGTTAATACAAAGATTAAACAAACAATTTCCTAATGACATCGGATTATTTTGTGGATGTCTCTTATTGAACCACGTTGMTTGAAAAAATTATCAATTTCCGTGTTGGATAAACARAGATTGACTGAAAAATTCAATAAATTGGATAAATCCATTAAAGATAATTTGAAGGCTAAACAAAAAGAAGAAACCAAAAAGACTTTAGATGTGGTTAACAATTGGTTGAATGATAAAGAAAATGCTTCATCATTTTTGGTTGCTCACGTTCCAATTACTGCTAATGCCAAGGCAATCACTGAAGCCATTAATTTGATTAAAAAGCAAGATAAAACCAAATCAATTTATTTATTGACTGGTGAAACMGATAAAGTTGCTCATGGATGTTATGTTAGTGATGAAGCCATTGCCAAGGGTATTAATGCGAAAATCAAACCTTTTGAAGCACGAGCAATTAACTGGTCMACGGATCTTAATGCTGAGGTACATATTGAGCATTATATAAATATATTCAATTATGCACGATCATCTTGGGAGCCATTGGTTGAAAGTTGGCCAATAGCAGTTTACATGTCAAAATCCCGACACCCAAAGCCTCAATTATTAGTAGAGGTGATTTCTAGACAGGTAGCTCAAGTGACGCTTACATCCAAAGCAGTAGCATTGCTATCTCAAGTATCCGATTTGATTACTTCCRGAGAAAAATTAAAACCAAGAGGTGAAGATTACCCATACRTKATAGTGAATGAGACTGGTTTAGATTTGGAAGTTTGGAATGATGCAAAWGAATYCGAAACCAATGGAGTTCCAATTGTTTTACGTGCTGGTAAAGCTTTAGATGAAAGTAAAGTTGAAATTAGAATTCAATTTAAACCAGTCGCCAAGGGGATGTTTAAAGAAATTCAAAGAAATGAATTAGTTATTAGAGTACAACCAAATGAAGCCATTTATTTAAAAATTAATTCCAAAATCCCTGGAATTTCTACTGAAACTTCATTAACTGATTTAGATTTAACTTATGCTACTCGTTATTCTAAAGATTTTTGGATTCCTGAAGCTTATGAAGCATTAATTAGAGATTGTTATTTAGGTAATCATTCTAATTTTGTTAGAGATGATGAATTGGATGTTTCTTGGAAATTATTTACTCCATTATTGAAT

>Dm1_oral_6/12/13

AATTATTGAARTGGAATGATATTCCATTGGCTCCACCAGACAARATTTTGGGTATTTCTGAAGCTTATAACAATGATTCTAACCCTCAAAAARTCAATTTGGGGGTTGGTGCTTATAGAGATAATTCYGGTAAACCAATTATTTTCCCATCAGTTAAAAAAGCTGAAGAAATTTTATTGGGTAAAGAAACTGAAAAGGAATATACTGCCATTGTTGGTTCCAAAAATTTCCAATCAATTGTGAAAAATTTCATTTTCAACAATTCTAATAAAGATGCCAATGGTAAACAATTAATTGATGATGGTAGAATTGTTACTGCTCAAACCATCTCTGGTACTGGATCACTTAAACCTCTTATGGTGAAATTTTTGCTAAACATAGAGAACCAAATTTGGAAATTATTCGTGAGGTTGTTGATTCCAAACATATTGTTTTTGATGTGTTGGCACAATTCTTAATCAATCCAGACCCATGGGTTGCCATTGCTGCCGCTGAAGTTTATGTCAGACGTTCATACCGTGCTTATGATTTGGGTAAAATTGAATATCATGTTAATGACAGACTTCCTATTGTTGAATGGAAATTCAAGTTGGCTAATATGGGAGCCGCTGGTGTAAACGATGCTCAACAGGCTGCTGCTGCCGGTGGCGATGATTCGACATCTATGAAACATGCAGCTTCTGTGTCTGATTTGACCTTTGTTGTTGATTATTCATRAAAGCGAAATCCCAGGATACACTCTCCCCGATAATCCAAAGTTCACCCTTGGTAATTTGTTTGTAATAATTGGAGTCTTGTTGGTTTGTATTTTAGCTGTTGTCTCTCTTTTGAGAAATATTAGTGAGTCGGCCTTGTTCAAGAAGAATGGGTATGAACCGTTGGATTCGGATCCTAGTGTCATGAACCTAAACTTCGAGCCTACAACATTGTCCTTTGAAGATATTAAATATGAGGTTACTGGTGGTCGACAAATTTTAAATGGAGTCTTTGGGTTTGTAAAACCAAGAGAATGTTTGGCTATAATGGGAGGTTCAGGTGCTGGTAAAACTACATTGTTGGATTTTAAACCTTTGGACCAATTGGCTAARACTTTGRCYACTGTTCCTGAATTGAATGAAATTATTGGKCAAGAWTTAGTTGACGAATTTRTCAGTGGTATTAAACTACCAGCAGAAGTTGGAAGTCAAGATGATGTTAACAATAGAAAATTGTTGCAAAAAGTGTTTGGTAAATTAATGAACACTGATGATGACGTTATAAAACAACAAACAGCTAAATTACTTGAAAGAACAGACAGAGAACCTCAAGTGTTCAAGGATATTGATTCTAGATTACCGGAGTTAATACAAAGATTAAACAAACAATTTCCTAATGACATCGGATTATTTTGTGGATGTCTCTTATTGAACCACGTTGATTGAAAAAATTATCAATTTCCGTGTTGGATAAACAGAGATTGACTGAAAAATTCAATAAATTGGATAAATCCATCAAAGATAATTTGAAGGCTAAACAAAAAGAAGAAACCAAAAAAACTTTAGATGTGGTTAATAATTGGTTGAATGATAAAGAAAATGCTTCATCATTTTTGGTTGCTCACGTTCCAATTACTGCTAATGCCAAGGCAATCACTGAAGCCATTAATTTGATTAAAAAGCAAGATAAAACCAAATCAATTTATTTATTGACTGGTGAAACCGATAAAGTTGCTCATGGATGTTATGTTAGTGATGAAGCCATTGCCAAGGGTATTAATGCGAAAATCAAACCTTTTGAAGCACGAGCAATTAACTGGTCCACGGATCTTAATGCTGAGGTACATATTGAGCATTATATAAATATATTCAATTATGCACGATCATCTTGGGAGCCATTGGTTGAAAGTTGGCCAATAGCAGTTTACATGTCAAAATCCCGACACCCAAAGCCTCAATTATTAGTAGAGGTGATTTCTAGACAGGTAGCYCAAGTGACGCTTACATCCAAAGCAGTAGCATTGCTATCTCAAGTATCCGATTTGATTACTTCCRGAGAAAAATTAAAACCAAGAGGTGAAGATTACCCATACGTKATAGTGAATGAGACTGGTTTAGATTTGGAAGTTTGGAATGATGCAAAWGAATYCGAAACCAATGGAGTTCCAATTGTTTTACGTGCTGGTAAAGCTTTAGATGAAAGTAAAGTTGAAATTAGAATTCAATTTAAACCAGTCGCCAAGGGGATGTTTAAAGAAATTCAAAGAAATGAATTAGTTATTAGAGTACAACCAAATGAAGCCATTTATTTAAAAATTAATTCCAAAATCCCTGGAATTTCTACTGAAACTTCATTAACTGATTTAGATTTAACTTATGCTACTCGTTATTCTAAAGAYTTTTGGATTCCTGAAGCTTATGAAGCATTAATTAGAGATTGYTATTTAGGTAATCATTCTAATTTTGTTAGAGATGATGAATTGGATGTTTCTTGGAAATTATTTACTCCATTATTGAAT

>Dm2_oral_4/1/14

AATTATTGAARTGGAATGATATTCCATTGGCTCCACCAGACAARATTTTGGGTATTTCTGAAGCTTATAACAATGATTCTAACCCTCAAAAARTCAATTTGGGGGTTGGTGCTTATAGAGATAATTCYGGTAAACCAATTATTTTCCCATCAGTTAAAAAAGCTGAAGAAATTTTATTGGGTAAAGAAACTGAAAAGGAATATACTGCCATTGTTGGTTCCAAAAATTTCCAATCAATTGTGAAAAATTTCATTTTCAACAATTCTAATAAAGATGCCAATGGTAAACAATTAATTGATGATGGTAGAATTGTTACTGCTCAAACCATCTCTGGTACTGGATCACTTAAACCTCTTATGGTGAAATTTTTGCTAAACATAGAGAACCAAATTTGGAAATTATTCGTGAGGTTGTTGATTCCAAACATATTGTTTTTGATGTGTTGGCACAATTCTTAATCAATCCAGACCCATGGGTTGCCATTGCTGCCGCTGAAGTTTATGTCAGACGTTCATACCGTGCTTATGATTTGGGTAMAATTGAATATCATGTTAATGACAGACTTCCTATTGTTGAATGGAAATTCAAGTTGGCTAATATGGGAGCCGCTGGTGTAAACGATGCTCAACAGGCTGCTGCTGCYGGTGGCGATGATTCGACATCTATGAAACATGCAGCTTCTGTGTCTGATTTGACCTTTGTTGTTGATTATTCATRAAAGCGAAATCCCAGGATACACTCTCCCCGATAATCCAAAGTTCACCCTTGGTAATTTGTTTGTAATAATTGGAGTCTTGTTGGTTTGTATTTTAGCTGTTGTCTCTCTTTTGAGAAATATTAGTGAGTCGGCCTTGTTCAAGAAGAATGGGTATGAACCGTTGGATTCGGATCCTAGTGTCATGAACCTAAACTTCGAGCCTACAACATTGTCCTTTGAAGATATTAAATATGAGGTTACTGGTGGTCGACAAATTTTAAATGGAGTCTTTGGGTTTGTAAAACCAAGAGAATGTTTGGCTATAATGGGAGGTTCAGGTGCTGGTAAAACTACATTGTTGGATTTTAAACCTTTGGACCAATTGGCTAARACTTTGRCYACTGTTCCTGAATTGAATGAAATTATTGGKCAAGAWTTAGTTGACGAATTTRTCAGTGGTATTAAACTACCAGCAGAAGTTGGAAGTCAAGATGATGTTAACAATAGAAAATTGTTGCAAAAAGTGTTTGGTAAATTAATGAACACTGATGATGACGTTATAAAACAACAAACAGCTAAATTACTTGAAAGAACAGACAGAGAACCTCAAGTGTTCAAGGATATTGATTCTAGATTACCGGAGTTAATACAAAGATTAAACAAACAATTTCCTAATGACATCGGATTATTTTGTGGATGTCTCTTATTGAACCACGTTGATTGAAAAAATTATCAATTTCCGTGTTGGATAAACAGAGATTGACTGAAAAATTCAATAAATTGGATAAATCCATCAAAGATAATTTGAAGGCTAAACAAAAAGAAGAAACCAAAAAAACTTTAGATGTGGTTAATAATTGGTTGAATGATAAAGAAAATGCTTCATCATTTTTGGTTGCTCACGTTCCAATTACTGCTAATGCCAAGGCAATCACTGAAGCCATTAATTTGATTAAAAAGCAAGATAAAACCAAATCAATTTATTTATTGACTGGTGAAACCGATAAAGTTGCTCATGGATGTTATGTTAGTGATGAAGCCATTGCCAAGGGTATTAATGCGAAAATCAAACCTTTTGAAGCACGAGCAATTAACTGGTCCACGGATCTTAATGCTGAGGTACATATTGAGCATTATATAAATATATTCAATTATGCACGATCATCTTGGGAGCCATTGGTTGAAAGTTGGCCAATAGCAGTTTACATGTCAAAATCCCGACACCCAAAGCCTCAATTATTAGTAGAGGTGATTTCTAGACAGGTAGCYCAAGTGACGCTTACATCCAAAGCAGTAGCATTGCTATCTCAAGTATCCGATTTGATTACTTCCRGAGAAAAATTAAAACCAAGAGGTGAAGATTACCCATACGTKATAGTGAATGAGACTGGTTTAGATTTGGAAGTTTGGAATGATGCAAAWGAATYCGAAACCAATGGAGTTCCAATTGTTTTACGTGCTGGTAAAGCTTTAGATGAAAGTAAAGTTGAAATTAGAATTCAATTTAAACCAGTCGCCAAGGGGATGTTTAAAGAAATTCAAAGAAATGAATTAGTTATTAGAGTACAACCAAATGAAGCCATTTATTTAAAAATTAATTCCAAAATCCCTGGAATTTCTACTGAAACTTCATTAACTGATTTAGATTTAACTTATGCTACTCGTTATTCTAAAGAYTTTTGGATTCCTGAAGCTTATGAAGCATTAATTAGAGATTGYTATTTAGGTAATCATTCTAATTTTGTTAGAGATGATGAATTGGATGTTTCTTGGAAATTATTTACTCCATTATTGAAT

>Dm3_oral_10/1/14

AATTATTGAAATGGAATGATATTCCATTGGCTCCACCAGACAARATTTTGGGTATTTCTGAAGCTTATAACAATGATTCTAACCCTCAAAAARTCAATTTGGGGGTTGGTGCTTATAGAGATAATTCYGGTAAACCAATTATTTTCCCATCAGTTAAAAAAGCTGAAGAAATTTTATTGGGTAAAGAAACTGAAAAGGAATATACTGCCATTGTTGGTTCCAAAAATTTCCAATCAATTGTGAAAAATTTCATTTTCAACAATTCTAATAAAGATGCCAATGGTAAACAATTAATTGATGATGGTAGAATTGTTACTGCTCAAACCATCTCTGGTACTGGATCACTTAAACCTCTTATGGTGAAATTTTTGCTAAACATAGAGAACCAAATTTGGAAATTATTCGTGAGGTTGTTGATTCCAAACATATTGTTTTTGATGTGTTGGCACAATTCTTAATCAATCCAGACCCATGGGTTGCCATTGCTGCCGCTGAAGTTTATGTCAGACGTTCATACCGTGCTTATGATTTGGGTAAAATTGAATATCATGTTAATGACAGACTTCCTATTGTTGAATGGAAATTCAAGTTGGCTAATATGGGAGCCGCTGGTGTAAACGATGCTCAACAGGCTGCTGCTGCCGGTGGCGATGATTCGACATCTATGAAACATGCAGCTTCTGTGTCTGATTTGACCTTTGTTGTTGATTATTCATAAAAGCGAAATCCCAGGATACACTCTCCCCGATAATCCAAAGTTCACCCTTGGTAATTTGTTTGTAATAATTGGAGTCTTGTTGGTTTGTATTTTAGCTGTTGTCTCTCTTTTGAGAAATATTAGTGAGTCGGCCTTGTTCAAGAAGAATGGGTATGAACCGTTGGATTCGGATCCTAGTGTCATGAACCTAAACTTCGAGCCTACAACATTGTCCTTTGAAGATATTAAATATGAGGTTACTGGTGGTCGACAAATTTTAAATGGAGTCTTTGGGTTTGTAAAACCAAGAGAATGTTTGGCTATAATGGGAGGTTCAGGTGCTGGTAAAACTACATTGTTGGATTTTAAACCTTTGGACCAATTGGCTAAGACTTTGACCACTGTTCCTGAATTGAATGAAATTATTGGGCAAGATTTAGTTGACGAATTTGTCAGTGGTATTAAACTACCAGCAGAAGTTGGAAGTCAAGATGATGTTAACAATAGAAAATTGTTGCAAAAAGTGTTTGGTAAATTAATGAACACTGATGATGACGTTATAAAACAACAAACAGCTAAATTACTTGAAAGAAMAGACAGAGAACCTCAAGTGTTCAAGGATATTGATTCTAGATTACCGGAGTTAATACAAAGATTAAACAAACAATTTCCTAATGACATCGGATTATTTTGTGGATGTCTCTTATTGAACCACGTTGMTTGAAAAAATTATCAATTTCCGTGTTGGATAAACARAGATTGACTGAAAAATTCAATAAATTGGATAAATCCATYAAAGATAATTTGAAGGCTAAACAAAAAGAAGAAACCAAAAARACTTTAGATGTGGTTAAYAATTGGTTGAATGATAAAGAAAATGCTTCATCATTTTTGGTTGCTCACGTTCCAATTACTGCTAATGCCAAGGCAATCACTGAAGCCATTAATTTGATTAAAAAGCAAGATAAAACCAAATCAATTTATTTATTGACTGGTGAAACCGATAAAGTTGCTCATGGATGTTATGTTAGTGATGAAGCCATTGYCAAGGGTATTAATGCGAAAATCAAACCTTTTGAAGCACGAGCAATTAACTGGTCMACGGATCTTAATGCTGAGGTACATATTGAGCATTATATAAATATATTCAATTATGCACGATCATCTTGGGAGCCATTGGTTGAAAGTTGGCCAATAGCAGTTTACATGTCAAAATCCCGAYACCCAAAGCCTCAATTATTAGTAGAGGTGATTTCTAGACAGGTAGCTCAAGTGACGCTTACATCCAAAGCAGTAGCATTGCTATCTCAAGTATCCGATTTGATTACTTCCGGAGAAAAATTAAAACCAAGAGGTGAAGATTACCCATACGTTATAGTGAATGAGACTGGTTTAGATTTGGAAGTTTGGAATGATGCAAAAGAATCCGAAACCAATGGAGTTCCAATTGTTTTACGTGCTGGTAAAGCTTTAGATGAAAGTAAAGTTGAAATTAGAATTCAATTTAAACCAGTCGCCAAGGGGATGTTTAAAGAAATTCAAAGAAATGAATTAGTTATTAGAGTACAACCAAATGAAGCCATTTATTTAAAAATTAATTCCAAAATCCCTGGAATTTCTACTGAAACTTCATTAACTGATTTAGATTTAACTTATGCTACTCGTTATTCTAAAGAYTTTTGGATTCCTGAAGCTTATGAAGCATTAATTAGAGATTGYTATTTAGGTAATCATTCTAATTTTGTTAGAGATGATGAATTGGATGTTTCTTGGAAATTATTTACTCCATTATTGAAT

>Dm4_oral_28/1/14

AATTATTGAAATGGAATGATATTCCATTGGCYCCACCAGACAAGATTTTGGGTATTTCTGAAGCTTATAACAATGATTCTAACCCTCAAAAARTCAATTTGGGGGTTGGTGCTTATAGAGATAATTCTGGTAAACCAATTATTTTCCCATCAGTTAAAAAAGCTGAAGAAATTTTATTGGGTAAAGAAACTGAAAAGGAATATACTGCCATTGTTGGTTCCAAAAATTTCCAATCAATTGTGAAAAATTTCATTTTCAACAATTCTAATAAAGATGCCAATGGTAAACAATTAATTGATGATGGTAGAATTGTTACTGCTCAAACCATYTCTGGTACTGGATCACTTAAACCTCTTATGGTGAAATTTTTGCTAAACATAGAGAACCAAATTTGGAAATTATTCGTGAGGTTGTTGATTCCAAACATATTGTTTTTGATGTGTTGGCACAATTCTTAATCAATCCAGACCCATGGGTTGCCATTGCTGCCGCTGAAGTTTATGTCAGACGTTCATACCGTGCTTATGATTTGGGTAAAATTGAATATCATGTTAATGACAGACTTCCTATTGTTGAATGGAAATTCAAGTTGGCTAATATGGGAGCYGCTSGTGTAAACGATGCTCAACAGGCTGCTGCTGCCGGTGGCGATGATTCGACATCTATGAAACATGCAGCTTCTGTGTCTGATTTGACCTTTGTTGTTGATTATTCATAAAAGCGAAATCCCAGGATACACTCTCCCCGATAATCCAAAGTTCACCCTTGGTAATTTGTTTGTAATAATTGGAGTCTTGTTGGTTTGTATTTTAGCTGTTGTCTCTCTTTTGAGAAATATTAGTGAGTCGGCCTTGTTCAAGAAGAATGGGTATGAACCGTTGGATTCGGATCCTAGTRTCATGAACCTAAACTTCGAGCCTACAACATTGTCCTTTGAAGATATTAAATATGAGGTTACTGGTGGTCGACAAATTTTAAATGGAGTCTTTGGGTTTGTAAAACCAAGAGAATGTTTGGCTATAATGGGAGGTTCAGGTGCTGGTAAAACTACATTGTTGGATTTTAAACCTTTGGACCAATTGGCTAAGACTTTGACCACTGTTCCTGAATTGAATGAAATTATTGGGCAAGATTTAGTTGACGAATTTGTCAGTGGTATTAAACTACCAGCAGAAGTTGGAAGTCAAGATGATGTTAACAATAGAAAATTGTTGCAAAAAGTGTTTGGTAAATTAATGAACACTGATGATGACGTTATAAAACAACAAACAGCTAAATTACTTGAAAGAACAGACAGAGAACCTCAAGTGTTCAAGGATATTGATTCTAGATTACCGGAGTTAATACAAAGATTAAACAAACAATTTCCTAATGACATCGGATTATTTTGTGGATGTCTCTTATTGAACCACGTTGMTTGAAAAAATTATCAATTTCCGTGTTGGATAAACARAGATTGACTGAAAAATTCAATAAATTGGATAAATCCATYAAAGATAATTTGAAGGCTAAACAAAAAGAAGAAACCAAAAARACTTTAGATGTGGTTAAYAATTGGTTGAATGATAAAGAAAATGCTTCATCATTTTTGGTTGCTCACGTTCCAATTACTGCTAATGCCAAGGCAATCACTGAAGCCATTAATTTGATTAAAAAGCAAGATAAAACCAAATCAATTTATTTATTGACTGGTGAAACCGATAAAGTTGCTCATGGATGTTATGTTAGTGATGAAGCCATTGYCAAGGGTATTAATGCGAAAATCAAACCTTTTGAAGCACGAGCAATTAACTGGTCCACGGATCTTAATGCTGAGGTACATATTGAGCATTATATAAATATATTCAATTATGCACGATCATCTTGGGAGCCATTGGTTGAAWGTTGGCCAATAGCAGTTTACATGTCAAAATCCCGACACCCAAAGCCTCAATTATTAGTAGAGGTGATTTCTAGACAGGTAGCTCAAGTGACGCTTACATCCAAAGCAGTAGCATTGCTATCTCAAGTATCCGATTTGATTACTTCCAGAGAAAAATTAAAACCAAGAGGTGAAGATTACCCATACGTTATAGTGAATGAGACTGGTTTAGATTTGGAAGTTTGGAATGATGCAAATGAATCCGAAACCAATGGAGTTCCAATTGTTTTACGTGCTGGTAAAGCTTTAGATGAAAGTAAAGTTGAAATTAGAATTCAATTTAAACCAGTCGCCAAGGGGATGTTTAAAGAAATTCAAAGAAATGAATTAGTTATTAGAGTACAACCAAATGAAGCCATTTATTTAAAAATTAATTCCAAAATCCCTGGAATTTCTACTGAAACTTCATTAACTGATTTAGATTTAACTTATGCTACTCGTTATTCTAAAGACTTTTGGATTCCTGAAGCTTATGAAGCATTAATTAGAGATTGTTATTTAGGTAATCATTCTAATTTTGTTAGAGATGATGAATTGGATGTTTCTTGGAAATTATTTACTCCATTATTGAAT

>Dm5_oral_16/1/14

AATTATTGAARTGGAATGATATTCCATTGGCTCCACCAGACAAAATTTTGGGTATTTCTGAAGCTTATAACAATGATTCTAACCCTCAAAAAATCAATTTGGGGGTTGGTGCTTATAGAGATAATTCCGGTAAACCAATTATTTTCCCATCAGTTAAAAAAGCTGAAGAAATTTTATTGGGTAAAGAAACTGAAAAGGAATATACTGCCATTGTTGGTTCCAAAAATTTCCAATCAATTGTGAAAAATTTCATTTTCAACAATTCTAATAAAGATGCCAATGGTAAACAATTAATTGATGATGGTAGAATTGTTACTGCTCAAACCATCTCTGGTACTGGATCACTTAAACCTCTTATGGTGAAATTTTTGCTAAACATAGAGAACCAAATTTGGAAATTATTCGTGAGGTTGTTGATTCCAAACATATTGTTTTTGATGTGTTGGCACAATTCTTAATCAATCCAGACCCATGGGTTGCCATTGCTGCCGCTGAAGTTTATGTCAGACGTTCATACCGTGCTTATGATTTGGGTAAAATTGAATATCATGTTAATGACAGACTTCCTATTGTTGAATGGAAATTCAAGTTGGCTAATATGGGAGCCGCTGGTGTAAACGATGCTCAACAGGCTGCTGCTGCCGGTGGCGATGATTCGACATCTATGAAACATGCAGCTTCTGTGTCTGATTTGACCTTTGTTGTTGATTATTCATAAAAGYGAAATYCCAGGATACACTCTCCCCGATAATCCAAAGTTCACCCTTGGTAATTTGTTTGTAATAATTGGRGTCTTGTTGGTTTGTRTTTTAGCTGTTGTCTCTCTTTTGAGAAATATTAGTGAGTCRGCCTTGTTCAAGAAGAATGGGTATGAACCGTTGGATTCRGATCCTAGTGTCATGAACCWAAACTTYGAGCCTACAACATTGTCCTTTGAAGATATTAAATATGAGGTTACTGGTGGTCGACAAATTTTAAATGGAGTCTTTGGGTTTGTAAAACCAAGAGAATGTTTGGCTATAATGGGAGGTTCAGGTGCTGGTAAAACTACATTGTTGGATTTTAAACCTTTGGACCAATTGGCTAARACTTTGRCYACTGTTCCTGAATTGAATGAAATTATTGGKCAAGAWTTAGTTGACGAATTTRTCAGTGGTATTAAACTACCAGCAGAAGTTGGAAGTCAAGATGATGTTAACAATAGAAAATTGTTGCAAAAAGTGTTTGGTAAATTAATGAACACTGATGATGACGTTATAAAACAACAAACAGCTAAATTACTTGAAAGAACAGACAGAGAACCTCAAGTGTTCAAGGATATTGATTCTAGATTACCGGAGTTAATACAAAGATTAAACAAACAATTTCCTAATGACATCGGATTATTTTGTGGATGTCTCTTATTGAACCACGTTGMTTGAAAAAATTATCAATTTCCGTGTTGGATAAACARAGATTGACTGAAAAATTCAATAAATTGGATAAATCCATTAAAGATAATTTGAAGGCTAAACAAAAAGAAGAAACCAAAAAGACTTTAGATGTGGTTAACAATTGGTTGAATGATAAAGAAAATGCTTCATCATTTTTGGTTGCTCACGTTCCAATTACTGCTAATGCCAAGGCAATCACTGAAGCCATTAATTTGATTAAAAAGCAAGATAAAACCAAATCAATTTATTTATTGACTGGTGAAACMGATAAAGTTGCTCATGGATGTTATGTTAGTGATGAAGCCATTGCCAAGGGTATTAATGCGAAAATCAAACCTTTTGAAGCACGAGCAATTAACTGGTCMACGGATCTTAATGCTGAGGTACATATTGAGCATTATATAAATATATTCAATTATGCACGATCATCTTGGGAGCCATTGGTTGAAAGTTGGCCAATAGCAGTTTACATGTCAAAATCCCGACACCCAAAGCCTCAATTATTAGTAGAGGTGATTTCTAGACAGGTAGCTCAAGTGACGCTTACATCCAAAGCAGTAGCATTGCTATCTCAAGTATCCGATTTGATTACTTCCRGAGAAAAATTAAAACCAAGAGGTGAAGATTACCCATACRTKATAGTGAATGAGACTGGTTTAGATTTGGAAGTTTGGAATGATGCAAAWGAATYCGAAACCAATGGAGTTCCAATTGTTTTACGTGCTGGTAAAGCTTTAGATGAAAGTAAAGTTGAAATTAGAATTCAATTTAAACCAGTCGCCAAGGGGATGTTTAAAGAAATTCAAAGAAATGAATTAGTTATTAGAGTACAACCAAATGAAGCCATTTATTTAAAAATTAATTCCAAAATCCCTGGAATTTCTACTGAAACTTCATTAACTGATTTAGATTTAACTTATGCTACTCGTTATTCTAAAGATTTTTGGATTCCTGAAGCTTATGAAGCATTAATTAGAGATTGTTATTTAGGTAATCATTCTAATTTTGTTAGAGATGATGAATTGGATGTTTCTTGGAAATTATTTACTCCATTATTGAAT

>Dm7_oral_19/3/14

AATTATTGAAATGGAATGATATTCCATTGGCTCCACCAGACAAAATTTTGGGTATTTCTGAAGCTTATAACAACGATTCTAACCCTCAAAAAATCAATTTGGGGGTTGGTGCTTATAGAGATAATTCCGGTAAACCAATTATTTTCCCATCAGTTAAAAAAGCTGAAGAAATTTTATTGGGTAAAGAAACTGAAAAGGAATATACTGCCATTGTTGGTTCCAAAAATTTCCAATCAATTGTGAAAAATTTCATTTTCAACAATTCTAATAAAGATGCCAATGGTAAACAATTAATTGATGATGGTAGAATTGTTACTGCTCAAACCATCTCTGGTACTGGATCACTTAAACCTCTTATGGTGAAATTTTTGCTAAACATAGAGAACCAAATTTGGAAATTATTCGTGAGGTTGTTGATTCCAAACATATTGTTTTTGATGTGTTGGCACAATTCTTAATCAATCCAGACCCATGGGTTGCCATTGCTGCCGCTGAAGTTTATGTCAGACGTTCATACCGTGCTTATGATTTGGGTAMAATTGAATATCATGTTAATGACAGACTTCCTATTGTTGAATGGAAATTCAAGTTGGCTAATATGGGAGCYGCTGGTGTAAACGATGCTCAACAGGCTGCTGCTGCYGGTGGCGATGATTCGACATCTATGAAACATGCAGCTTCTGTGTCTGATTTGACCTTTGTTGTTGATTATTCATAAAAGCGAAATCCCAGGATACACTCTCCCCGATAATCCAAAGTTCACCCTTGGTAATTTGTTTGTAATAATTGGAGTCTTGTTGGTTTGTRTTTTAGCTGTTGTCTCTCTTTTGAGAAATATTAGTGAGTCGGCCTTGTTCAAGAAGAATGGGTATGAACCGTTGGATTCGGATCCTAGTGTCATGAACCTAAACTTCGAGCCTACAACATTGTCCTTTGAAGATATTAAATATGAGGTTACTGGTGGTCGACAAATTTTAAATGGAGTCTTTGGGTTTGTAAAACCAAGAGAATGTTTGGCTATAATGGGAGGTTCAGGTGCTGGTAAAACTACATTGTTGGATTTTAAACCTTTGGACCAATTGGCTAAGACTTTGACCACTGTTCCTGAATTGAATGAAATTATTGGGCAAGATTTAGTTGACGAATTTGTCAGTGGTATTAAACTACCAGCAGAAGTTGGAAGTCAAGATGATGTTAACAATAGAAAATTGTTGCAAAAAGTGTTTGGTAAATTAATGAACACTGATGATGACGTTATAAAACAACAAACAGCTAAATTACTTGAAAGAACAGACAGAGAACCTCAAGTGTTCAAGGATATTGATTCTAGATTACCGGAGTTAATACAAAGATTAAACAAACAATTTCCTAATGACATCGGATTATTTTGTGGATGTCTCTTATTGAACCACGTTGMTTGAAAAAATTATCAATTTCCGTGTTGGATAAACAAAGATTGACTGAAAAATTCAATAAATTGGATAAATCCATYAAAGATAATTTGAAGGCTAAACAAAAAGAAGAAACCAAAAARACTTTAGATGTGGTTAAYAATTGGTTGAATGATAAAGAAAATKCTTCATCATTTTTGGTTGCTCACGTTCCAATTACTGCTAATGCCAAGGCAATCACTGAAGCCATTAATTTGATTAAAAAGCAAGATAAAACCAAATCAATTTATTTATTGACTGGTGAAACCGATAAAGTTGCTCATGGATGTTATGTTAGTGATGAAGCCATTGTCAAGGGTATTAATGCGAAAATCAAACCTTTTGAAGCACGAGCAATTAACTGGTCCACGGATCTTAATGCTGAGGTACATATTGAGCATTATATAAATATATTCAATTATGCACGATCATCTTGGGAGCCATTGGTTGAAAGTTGGCCAATAGCAGTTTACATGTCAAAATCCCGACACCCAAAGCCTCAATTATTAGTAGAGGTGATTTCTAGACAGGTAGCTCAAGTGACGCTTACATCCAAAGCGGTAGCATTGCTATCTCAAGTATCCGATTTGATTACTTCCGGAGAAAAATTAAAACCAAGAGGTGAAGATTACCCATACGTKATAGTGAATGAGACTGGTTTAGATTTGGAAGTTTGGAATGATGCAAATGAATYCGAAACCAATGGAGTTCCAATTGTTTTACGTGCTGGTAAAGCTTTAGATGAAAGTAAAGTTGAAATTAGAATTCAATTTAAACCAGTCGCCAAGGGGATGTTTAAAGAAATTCAAAGAAATGAATTAGTTATTAGAGTACAACCAAATGAAGCCATTTATTTAAAAATTAATTCCAAAATCCCTGGAATTTCTACTGAAACTTCATTAACTGATTTAGATTTAACTTATGCTACTCGTTATTCTAAAGACTTTTGGATTCCTGAAGCTTATGAAGCATTAATTAGAGATTGCTATTTAGGTAATCATTCTAATTTTGTTAGAGATGATGAATTGGATGTTTCTTGGAAATTATTTACTCCATTATTGAAT

>Dm8_oral_17/10/14

AATTATTGAAATGGAATGATATTCCATTGGCCCCACCAGACAAGATTTTGGGTATTTCTGAAGCTTATAACAATGATTCTAACCCTCAAAAAATCAATTTGGGGGTTGGTGCTTATAGAGATAATTCTGGTAAACCAATTATTTTCCCATCAGTTAAAAAAGCTGAAGAAATTTTATTGGGTAAAGAAACTGAAAAGGAATATACTGCCATTGTTGGTTCCAAAAATTTCCAATCAATTGTGAAAAATTTCATTTTCAACAATTCTAATAAAGATGCCAATGGTAAACAATTAATTGATGATGGTAGAATTGTTACTGCTCAAACCATCTCTGGTACTGGATCACTTAAACCTCTTATGGTGAAATTTTTGCTAAACATAGAGAACCAAATTTGGAAATTATTCGTGAGGTTGTTGATTCCAAACATATTGTTTTTGATGTGTTGGCACAATTCTTAATCAATCCAGACCCATGGGTTGCCATTGCTGCCGCTGAAGTTTATGTCAGACGTTCATACCGTGCTTATGATTTGGGTAAAATTGAATATCATGTTAATGACAGACTTCCTATTGTTGAATGGAAATTCAAGTTGGCTAATATGGGAGCCGCTGGTGTAAACGATGCTCAACAGGCTGCTGCTGCCGGTGGCGATGATTCGACATCTATGAAACATGCAGCTTCTGTGTCTGATTTGACCTTTGTTGTTGATTATTCATAAAAGTGAAATTCCAGGATACACTCTCCCCGATAATCCAAAGTTCACCCTTGGTAATTTGTTTGTAATAATTGGGGTCTTGTTGGTTTGTRTTTTAGCTGTTGTCTCTCTTTTGAGAAATATTAGTGAGTCAGCCTTGTTCAAGAAGAATGGGTATGAACCGTTGGATTCAGATCCTAGTGTCATGAACCAAAACTTTGAGCCTACAACATTGTCCTTTGAAGATATTAAATATGAGGTTACTGGTGGTCGACAAATTTTAAATGGAGTCTTTGGGTTTGTAAAACCAAGAGAATGTTTGGCTATAATGGGAGGTTCAGGTGCTGGTAAAACTACATTGTTGGATTTTAAACCTTTGGACCAATTGGCTAAAACTTTGGCTACTGTTCCTGAATTGAATGAAATTATTGGTCAAGAATTAGTTGACGAATTTRTCAGTGGTATTAAACTACCAGCAGAAGTTGGAAGTCAAGATGATGTTAACAATAGAAAATTGTTGCAAAAAGTGTTTGGTAAATTAATGAACACTGATGATGACGTTATAAAACAACAAACAGCTAAATTACTTGAAAGAACAGACAGAGAACCTCAAGTGTTCAAGGATATTGATTCTAGATTACCGGAGTTAATACAAAGATTAAACAAACAATTTCCTAATGACATCGGATTATTTTGTGGATGTCTCTTATTGAACCACGTTGCTTGAAAAAATTATCAATTTCCGTGTTGGATAAACARAGATTGACTGAAAAATTCAATAAATTGGATAAATCCATYAAAGATAATTTGAAGGCTAAACAAAAAGAAGAAACCAAAAARACTTTAGATGTGGTTAAYAATTGGTTGAATGATAAAGAAAATGCTTCATCATTTTTGGTTGCTCACGTTCCAATTACTGCTAATGCCAAGGCAATCACTGAAGCCATTAATTTGATTAAAAAGCAAGATAAAACCAAATCAATTTATTTATTGACTGGTGAAACCGATAAAGTTGCTCATGGATGTTATGTTAGTGATGAAGCCATTGCCAAGGGTATTAATGCGAAAATCAAACCTTTTGAAGCACGAGCAATTAACTGGTCMACGGATCTTAATGCTGAGGTACATATTGAGCATTATATAAATATATTCAATTATGCACGATCATCTTGGGAGCCATTGGTTGAAAGTTGGCCAATAGCAGTTTACATGTCAAAATCCCGACACCCAAAGCCTCAATTATTAGTAGAGGTGATTTCTAGACAGGTAGCTCAAGTGACGCTTACATCCAAAGCAGTAGCATTGCTATCTCAAGTATCCGATTTGATTACTTCCRGAGAAAAATTAAAACCAAGAGGTGAAGATTACCCATACRTTATAGTGAATGAGACTGGTTTAGATTTGGAAGTTTGGAATGATGCAAAWGAATCCGAAACCAATGGAGTTCCAATTGTTTTACGTGCTGGTAAAGCTTTAGATGAAAGTAAAGTTGAAATTAGAATTCAATTTAAACCAGTYGCCAAGGGGATGTTTAAAGAAATTCAAAGAAATGAATTAGTTATTAGAGTACAACCAAATGAAGCCATTTATTTAAAAATTAATTCMAAAATCCCTGGRATTTCTACTGAAACTTCATTAACTGATTTAGATTTAACTTATGCTACTCGTTATTCTAAAGAYTTTTGGATTCCTGAAGCTTATGAAGCATTAATTAGAGATTGTTATTTAGGTAATCATTCTAATTTTGTTAGAGATGATGAATTGGATGTTTCTTGGAAATTATTTACTCCATTATTGAAT

>Dm9_oral_28/10/14

AATTATTGAAGTGGAATGATATTCCATTGGCTCCACCAGACAAAATTTTGGGTATTTCTGAAGCTTATAACAATGATTCTAACCCTCAAAAAATCAATTTGGGGGTTGGTGCTTATAGAGATAATTCCGGTAAACCAATTATTTTCCCATCAGTTAAAAAAGCTGAAGAAATTTTATTGGGTAAAGAAACTGAAAAGGAATATACTGCCATTGTTGGTTCCAAAAATTTCCAATCAATTGTGAAAAATTTCATTTTCAACAATTCTAATAAAGATGCCAATGGTAAACAATTAATTGATGATGGTAGAATTGTTACTGCTCAAACCATCTCTGGTACTGGATCACTTAAACCTCTTATGGTGAAATTTTTGCTAAACATAGAGAACCAAATTTGGAAATTATTCGTGAGGTTGTTGATTCCAAACATATTGTTTTTGATGTGTTGGCACAATTCTTAATCAATCCAGACCCATGGGTTGCCATTGCTGCCGCTGAAGTTTATGTCAGACGTTCATACCGTGCTTATGATTTGGGTAAAATTGAATATCATGTTAATGACAGACTTCCTATTGTTGAATGGAAATTCAAGTTGGCTAATATGGGAGCCGCTGGTGTAAACGATGCTCAACAGGCTGCTGCTGCCGGTGGCGATGATTCGACATCTATGAAACATGCAGCTTCTGTGTCTGATTTGACCTTTGTTGTTGATTATTCATAAAAGTGAAATTCCAGGATACACTCTCCCCGATAATCCAAAGTTCACCCTTGGTAATTTGTTTGTAATAATTGGGGTCTTGTTGGTTTGTRTTTTAGCTGTTGTCTCTCTTTTGAGAAATATTAGTGAGTCAGCCTTGTTCAAGAAGAATGGGTATGAACCGTTGGATTCAGATCCTAGTGTCATGAACCAAAACTTTGAGCCTACAACATTGTCCTTTGAAGATATTAAATATGAGGTTACTGGTGGTCGACAAATTTTAAATGGAGTCTTTGGGTTTGTAAAACCAAGAGAATGTTTGGCTATAATGGGAGGTTCAGGTGCTGGTAAAACTACATTGTTGGATTTTAAACCTTTGGACCAATTGGCTAAAACTTTGGCTACTGTTCCTGAATTGAATGAAATTATTGGTCAAGAATTAGTTGACGAATTTRTCAGTGGTATTAAACTACCAGCAGAAGTTGGAAGTCAAGATGATGTTAACAATAGAAAATTGTTGCAAAAAGTGTTTGGTAAATTAATGAACACTGATGATGACGTTATAAAACAACAAACAGCTAAATTACTTGAAAGAACAGACAGAGAACCTCAAGTGTTCAAGGATATTGATTCTAGATTACCGGAGTTAATACAAAGATTAAACAAACAATTTCCTAATGACATCGGATTATTTTGTGGATGTCTCTTATTGAACCACGTTGCTTGAAAAAATTATCAATTTCCGTGTTGGATAAACARAGATTGACTGAAAAATTCAATAAATTGGATAAATCCATYAAAGATAATTTGAAGGCTAAACAAAAAGAAGAAACCAAAAARACTTTAGATGTGGTTAAYAATTGGTTGAATGATAAAGAAAATGCTTCATCATTTTTGGTTGCTCACGTTCCAATTACTGCTAATGCCAAGGCAATCACTGAAGCCATTAATTTGATTAAAAAGCAAGATAAAACCAAATCAATTTATTTATTGACTGGTGAAACCGATAAAGTTGCTCATGGATGTTATGTTAGTGATGAAGCCATTGCCAAGGGTATTAATGCGAAAATCAAACCTTTTGAAGCACGAGCAATTAACTGGTCMACGGATCTTAATGCTGAGGTACATATTGAGCATTATATAAATATATTCAATTATGCACGATCATCTTGGGAGCCATTGGTTGAAAGTTGGCCAATAGCAGTTTACATGTCAAAATCCCGACACCCAAAGCCTCAATTATTAGTAGAGGTGATTTCTAGACAGGTAGCTCAAGTGACGCTTACATCCAAAGCAGTAGCATTGCTATCTCAAGTATCCGATTTGATTACTTCCRGAGAAAAATTAAAACCAAGAGGTGAAGATTACCCATACRTTATAGTGAATGAGACTGGTTTAGATTTGGAAGTTTGGAATGATGCAAAWGAATCCGAAACCAATGGAGTTCCAATTGTTTTACGTGCTGGTAAAGCTTTAGATGAAAGTAAAGTTGAAATTAGAATTCAATTTAAACCAGTYGCCAAGGGGATGTTTAAAGAAATTCAAAGAAATGAATTAGTTATTAGAGTACAACCAAATGAAGCCATTTATTTAAAAATTAATTCMAAAATCCCTGGRATTTCTACTGAAACTTCATTAACTGATTTAGATTTAACTTATGCTACTCGTTATTCTAAAGAYTTTTGGATTCCTGAAGCTTATGAAGCATTAATTAGAGATTGTTATTTAGGTAATCATTCTAATTTTGTTAGAGATGATGAATTGGATGTTTCTTGGAAATTATTTACTCCATTATTGAAT

>FJ9_S8:IND:29:5.9

AATTATTGAARTGGAATGATATTCCATTGGCTCCACCAGACAARATTTTGGGTATTTCTGAAGCTTATAACAATGATTCTAACCCTCAAAAARTCAATTTGGGGGTTGGTGCTTATAGAGATAATTCYGGTAAACCAATTATTTTCCCATCAGTTAAAAAAGCTGAAGAAATTTTATTGGGTAAAGAAACTGAAAAGGAATATACTGCCATTGTTGGTTCCAAAAATTTCCAATCAATTGTGAAAAATTTCATTTTCAACAATTCTAATAAAGATGCCAATGGTAAACAATTAATTGATGATGGTAGAATTGTTACTGCTCAAACCATCTCTGGTACTGGATCACTTAAACCTCTTATGGTGAAATTTTTGCTAAACATAGAGAACCAAATTTGGAAATTATTCGTGAGGTTGTTGATTCCAAACATATTGTTTTTGATGTGTTGGCACAATTCTTAATCAATCCAGACCCATGGGTTGCCATTGCTGCCGCTGAAGTTTATGTCAGACGTTCATACCGTGCTTATGATTTGGGTAMAATTGAATATCATGTTAATGACAGACTTCCTATTGTTGAATGGAAATTCAAGTTGGCTAATATGGGAGCCGCTGGTGTAAACGATGCTCAACAGGCTGCTGCTGCYGGTGGCGATGATTCGACATCTATGAAACATGCAGCTTCTGTGTCTGATTTGACCTTTGTTGTTGATTATTCATRAAAGCGAAATCCCAGGATACACTCTCCCCGATAATCCAAAGTTCACCCTTGGTAATTTGTTTGTAATAATTGGAGTCTTGTTGGTTTGTATTTTAGCTGTTGTCTCTCTTTTGAGAAATATTAGTGAGTCGGCCTTGTTCAAGAAGAATGGGTATGAACCGTTGGATTCGGATCCTAGTGTCATGAACCTAAACTTCGAGCCTACAACATTGTCCTTTGAAGATATTAAATATGAGGTTACTGGTGGTCGACAAATTTTAAATGGAGTCTTTGGGTTTGTAAAACCAAGAGAATGTTTGGCTATAATGGGAGGTTCAGGTGCTGGTAAAACTACATTGTTGGATTTTAAACCTTTGGACCAATTGGCTAARACTTTGRCYACTGTTCCTGAATTGAATGAAATTATTGGKCAAGAWTTAGTTGACGAATTTRTCAGTGGTATTAAACTACCAGCAGAAGTTGGAAGTCAAGATGATGTTAACAATAGAAAATTGTTGCAAAAAGTGTTTGGTAAATTAATGAACACTGATGATGACGTTATAAAACAACAAACAGCTAAATTACTTGAAAGAACAGACAGAGAACCTCAAGTGTTCAAGGATATTGATTCTAGATTACCGGAGTTAATACAAAGATTAAACAAACAATTTCCTAATGACATCGGATTATTTTGTGGATGTCTCTTATTGAACCACGTTGATTGAAAAAATTATCAATTTCCGTGTTGGATAAACAGAGATTGACTGAAAAATTCAATAAATTGGATAAATCCATCAAAGATAATTTGAAGGCTAAACAAAAAGAAGAAACCAAAAAAACTTTAGATGTGGTTAATAATTGGTTGAATGATAAAGAAAATGCTTCATCATTTTTGGTTGCTCACGTTCCAATTACTGCTAATGCCAAGGCAATCACTGAAGCCATTAATTTGATTAAAAAGCAAGATAAAACCAAATCAATTTATTTATTGACTGGTGAAACCGATAAAGTTGCTCATGGATGTTATGTTAGTGATGAAGCCATTGCCAAGGGTATTAATGCGAAAATCAAACCTTTTGAAGCACGAGCAATTAACTGGTCCACGGATCTTAATGCTGAGGTACATATTGAGCATTATATAAATATATTCAATTATGCACGATCATCTTGGGAGCCATTGGTTGAAAGTTGGCCAATAGCAGTTTACATGTCAAAATCCCGACACCCAAAGCCTCAATTATTAGTAGAGGTGATTTCTAGACAGGTAGCYCAAGTGACGCTTACATCCAAAGCAGTAGCATTGCTATCTCAAGTATCCGATTTGATTACTTCCRGAGAAAAATTAAAACCAAGAGGTGAAGATTACCCATACGTKATAGTGAATGAGACTGGTTTAGATTTGGAAGTTTGGAATGATGCAAAWGAATYCGAAACCAATGGAGTTCCAATTGTTTTACGTGCTGGTAAAGCTTTAGATGAAAGTAAAGTTGAAATTAGAATTCAATTTAAACCAGTCGCCAAGGGGATGTTTAAAGAAATTCAAAGAAATGAATTAGTTATTAGAGTACAACCAAATGAAGCCATTTATTTAAAAATTAATTCCAAAATCCCTGGAATTTCTACTGAAACTTCATTAACTGATTTAGATTTAACTTATGCTACTCGTTATTCTAAAGAYTTTTGGATTCCTGAAGCTTATGAAGCATTAATTAGAGATTGYTATTTAGGTAATCATTCTAATTTTGTTAGAGATGATGAATTGGATGTTTCTTGGAAATTATTTACTCCATTATTGAAT

>FJ9:IND:15:4.0

AATTATTGAARTGGAATGATATTCCATTGGCTCCACCAGACAARATTTTGGGTATTTCTGAAGCTTATAACAATGATTCTAACCCTCAAAAARTCAATTTGGGGGTTGGTGCTTATAGAGATAATTCYGGTAAACCAATTATTTTCCCATCAGTTAAAAAAGCTGAAGAAATTTTATTGGGTAAAGAAACTGAAAAGGAATATACTGCCATTGTTGGTTCCAAAAATTTCCAATCAATTGTGAAAAATTTCATTTTCAACAATTCTAATAAAGATGCCAATGGTAAACAATTAATTGATGATGGTAGAATTGTTACTGCTCAAACCATCTCTGGTACTGGATCACTTAAACCTCTTATGGTGAAATTTTTGCTAAACATAGAGAACCAAATTTGGAAATTATTCGTGAGGTTGTTGATTCCAAACATATTGTTTTTGATGTGTTGGCACAATTCTTAATCAATCCAGACCCATGGGTTGCCATTGCTGCCGCTGAAGTTTATGTCAGACGTTCATACCGTGCTTATGATTTGGGTAMAATTGAATATCATGTTAATGACAGACTTCCTATTGTTGAATGGAAATTCAAGTTGGCTAATATGGGAGCCGCTGGTGTAAACGATGCTCAACAGGCTGCTGCTGCYGGTGGCGATGATTCGACATCTATGAAACATGCAGCTTCTGTGTCTGATTTGACCTTTGTTGTTGATTATTCATRAAAGCGAAATCCCAGGATACACTCTCCCCGATAATCCAAAGTTCACCCTTGGTAATTTGTTTGTAATAATTGGAGTCTTGTTGGTTTGTATTTTAGCTGTTGTCTCTCTTTTGAGAAATATTAGTGAGTCGGCCTTGTTCAAGAAGAATGGGTATGAACCGTTGGATTCGGATCCTAGTGTCATGAACCTAAACTTCGAGCCTACAACATTGTCCTTTGAAGATATTAAATATGAGGTTACTGGTGGTCGACAAATTTTAAATGGAGTCTTTGGGTTTGTAAAACCAAGAGAATGTTTGGCTATAATGGGAGGTTCAGGTGCTGGTAAAACTACATTGTTGGATTTTAAACCTTTGGACCAATTGGCTAARACTTTGRCYACTGTTCCTGAATTGAATGAAATTATTGGKCAAGAWTTAGTTGACGAATTTRTCAGTGGTATTAAACTACCAGCAGAAGTTGGAAGTCAAGATGATGTTAACAATAGAAAATTGTTGCAAAAAGTGTTTGGTAAATTAATGAACACTGATGATGACGTTATAAAACAACAAACAGCTAAATTACTTGAAAGAACAGACAGAGAACCTCAAGTGTTCAAGGATATTGATTCTAGATTACCGGAGTTAATACAAAGATTAAACAAACAATTTCCTAATGACATCGGATTATTTTGTGGATGTCTCTTATTGAACCACGTTGATTGAAAAAATTATCAATTTCCGTGTTGGATAAACAGAGATTGACTGAAAAATTCAATAAATTGGATAAATCCATCAAAGATAATTTGAAGGCTAAACAAAAAGAAGAAACCAAAAAAACTTTAGATGTGGTTAATAATTGGTTGAATGATAAAGAAAATGCTTCATCATTTTTGGTTGCTCACGTTCCAATTACTGCTAATGCCAAGGCAATCACTGAAGCCATTAATTTGATTAAAAAGCAAGATAAAACCAAATCAATTTATTTATTGACTGGTGAAACCGATAAAGTTGCTCATGGATGTTATGTTAGTGATGAAGCCATTGCCAAGGGTATTAATGCGAAAATCAAACCTTTTGAAGCACGAGCAATTAACTGGTCCACGGATCTTAATGCTGAGGTACATATTGAGCATTATATAAATATATTCAATTATGCACGATCATCTTGGGAGCCATTGGTTGAAAGTTGGCCAATAGCAGTTTACATGTCAAAATCCCGACACCCAAAGCCTCAATTATTAGTAGAGGTGATTTCTAGACAGGTAGCYCAAGTGACGCTTACATCCAAAGCAGTAGCATTGCTATCTCAAGTATCCGATTTGATTACTTCCRGAGAAAAATTAAAACCAAGAGGTGAAGATTACCCATACGTKATAGTGAATGAGACTGGTTTAGATTTGGAAGTTTGGAATGATGCAAAWGAATYCGAAACCAATGGAGTTCCAATTGTTTTACGTGCTGGTAAAGCTTTAGATGAAAGTAAAGTTGAAATTAGAATTCAATTTAAACCAGTCGCCAAGGGGATGTTTAAAGAAATTCAAAGAAATGAATTAGTTATTAGAGTACAACCAAATGAAGCCATTTATTTAAAAATTAATTCCAAAATCCCTGGAATTTCTACTGAAACTTCATTAACTGATTTAGATTTAACTTATGCTACTCGTTATTCTAAAGAYTTTTGGATTCCTGAAGCTTATGAAGCATTAATTAGAGATTGYTATTTAGGTAATCATTCTAATTTTGTTAGAGATGATGAATTGGATGTTTCTTGGAAATTATTTACTCCATTATTGAAT

>FJ9:IND:16:5.5

AATTATTGAARTGGAATGATATTCCATTGGCTCCACCAGACAARATTTTGGGTATTTCTGAAGCTTATAACAATGATTCTAACCCTCAAAAARTCAATTTGGGGGTTGGTGCTTATAGAGATAATTCYGGTAAACCAATTATTTTCCCATCAGTTAAAAAAGCTGAAGAAATTTTATTGGGTAAAGAAACTGAAAAGGAATATACTGCCATTGTTGGTTCCAAAAATTTCCAATCAATTGTGAAAAATTTCATTTTCAACAATTCTAATAAAGATGCCAATGGTAAACAATTAATTGATGATGGTAGAATTGTTACTGCTCAAACCATCTCTGGTACTGGATCACTTAAACCTCTTATGGTGAAATTTTTGCTAAACATAGAGAACCAAATTTGGAAATTATTCGTGAGGTTGTTGATTCCAAACATATTGTTTTTGATGTGTTGGCACAATTCTTAATCAATCCAGACCCATGGGTTGCCATTGCTGCCGCTGAAGTTTATGTCAGACGTTCATACCGTGCTTATGATTTGGGTAMAATTGAATATCATGTTAATGACAGACTTCCTATTGTTGAATGGAAATTCAAGTTGGCTAATATGGGAGCCGCTGGTGTAAACGATGCTCAACAGGCTGCTGCTGCYGGTGGCGATGATTCGACATCTATGAAACATGCAGCTTCTGTGTCTGATTTGACCTTTGTTGTTGATTATTCATRAAAGCGAAATCCCAGGATACACTCTCCCCGATAATCCAAAGTTCACCCTTGGTAATTTGTTTGTAATAATTGGAGTCTTGTTGGTTTGTATTTTAGCTGTTGTCTCTCTTTTGAGAAATATTAGTGAGTCGGCCTTGTTCAAGAAGAATGGGTATGAACCGTTGGATTCGGATCCTAGTGTCATGAACCTAAACTTCGAGCCTACAACATTGTCCTTTGAAGATATTAAATATGAGGTTACTGGTGGTCGACAAATTTTAAATGGAGTCTTTGGGTTTGTAAAACCAAGAGAATGTTTGGCTATAATGGGAGGTTCAGGTGCTGGTAAAACTACATTGTTGGATTTTAAACCTTTGGACCAATTGGCTAARACTTTGRCYACTGTTCCTGAATTGAATGAAATTATTGGKCAAGAWTTAGTTGACGAATTTRTCAGTGGTATTAAACTACCAGCAGAAGTTGGAAGTCAAGATGATGTTAACAATAGAAAATTGTTGCAAAAAGTGTTTGGTAAATTAATGAACACTGATGATGACGTTATAAAACAACAAACAGCTAAATTACTTGAAAGAACAGACAGAGAACCTCAAGTGTTCAAGGATATTGATTCTAGATTACCGGAGTTAATACAAAGATTAAACAAACAATTTCCTAATGACATCGGATTATTTTGTGGATGTCTCTTATTGAACCACGTTGATTGAAAAAATTATCAATTTCCGTGTTGGATAAACAGAGATTGACTGAAAAATTCAATAAATTGGATAAATCCATCAAAGATAATTTGAAGGCTAAACAAAAAGAAGAAACCAAAAAAACTTTAGATGTGGTTAATAATTGGTTGAATGATAAAGAAAATGCTTCATCATTTTTGGTTGCTCACGTTCCAATTACTGCTAATGCCAAGGCAATCACTGAAGCCATTAATTTGATTAAAAAGCAAGATAAAACCAAATCAATTTATTTATTGACTGGTGAAACCGATAAAGTTGCTCATGGATGTTATGTTAGTGATGAAGCCATTGCCAAGGGTATTAATGCGAAAATCAAACCTTTTGAAGCACGAGCAATTAACTGGTCCACGGATCTTAATGCTGAGGTACATATTGAGCATTATATAAATATATTCAATTATGCACGATCATCTTGGGAGCCATTGGTTGAAAGTTGGCCAATAGCAGTTTACATGTCAAAATCCCGACACCCAAAGCCTCAATTATTAGTAGAGGTGATTTCTAGACAGGTAGCYCAAGTGACGCTTACATCCAAAGCAGTAGCATTGCTATCTCAAGTATCCGATTTGATTACTTCCRGAGAAAAATTAAAACCAAGAGGTGAAGATTACCCATACGTKATAGTGAATGAGACTGGTTTAGATTTGGAAGTTTGGAATGATGCAAAWGAATYCGAAACCAATGGAGTTCCAATTGTTTTACGTGCTGGTAAAGCTTTAGATGAAAGTAAAGTTGAAATTAGAATTCAATTTAAACCAGTCGCCAAGGGGATGTTTAAAGAAATTCAAAGAAATGAATTAGTTATTAGAGTACAACCAAATGAAGCCATTTATTTAAAAATTAATTCCAAAATCCCTGGAATTTCTACTGAAACTTCATTAACTGATTTAGATTTAACTTATGCTACTCGTTATTCTAAAGAYTTTTGGATTCCTGAAGCTTATGAAGCATTAATTAGAGATTGYTATTTAGGTAATCATTCTAATTTTGTTAGAGATGATGAATTGGATGTTTCTTGGAAATTATTTACTCCATTATTGAAT

>FJ9:IND:27:8.4

AATTATTGAARTGGAATGATATTCCATTGGCTCCACCAGACAARATTTTGGGTATTTCTGAAGCTTATAACAATGATTCTAACCCTCAAAAARTCAATTTGGGGGTTGGTGCTTATAGAGATAATTCYGGTAAACCAATTATTTTCCCATCAGTTAAAAAAGCTGAAGAAATTTTATTGGGTAAAGAAACTGAAAAGGAATATACTGCCATTGTTGGTTCCAAAAATTTCCAATCAATTGTGAAAAATTTCATTTTCAACAATTCTAATAAAGATGCCAATGGTAAACAATTAATTGATGATGGTAGAATTGTTACTGCTCAAACCATCTCTGGTACTGGATCACTTAAACCTCTTATGGTGAAATTTTTGCTAAACATAGAGAACCAAATTTGGAAATTATTCGTGAGGTTGTTGATTCCAAACATATTGTTTTTGATGTGTTGGCACAATTCTTAATCAATCCAGACCCATGGGTTGCCATTGCTGCCGCTGAAGTTTATGTCAGACGTTCATACCGTGCTTATGATTTGGGTAMAATTGAATATCATGTTAATGACAGACTTCCTATTGTTGAATGGAAATTCAAGTTGGCTAATATGGGAGCCGCTGGTGTAAACGATGCTCAACAGGCTGCTGCTGCYGGTGGCGATGATTCGACATCTATGAAACATGCAGCTTCTGTGTCTGATTTGACCTTTGTTGTTGATTATTCATRAAAGCGAAATCCCAGGATACACTCTCCCCGATAATCCAAAGTTCACCCTTGGTAATTTGTTTGTAATAATTGGAGTCTTGTTGGTTTGTATTTTAGCTGTTGTCTCTCTTTTGAGAAATATTAGTGAGTCGGCCTTGTTCAAGAAGAATGGGTATGAACCGTTGGATTCGGATCCTAGTGTCATGAACCTAAACTTCGAGCCTACAACATTGTCCTTTGAAGATATTAAATATGAGGTTACTGGTGGTCGACAAATTTTAAATGGAGTCTTTGGGTTTGTAAAACCAAGAGAATGTTTGGCTATAATGGGAGGTTCAGGTGCTGGTAAAACTACATTGTTGGATTTTAAACCTTTGGACCAATTGGCTAARACTTTGRCYACTGTTCCTGAATTGAATGAAATTATTGGKCAAGAWTTAGTTGACGAATTTRTCAGTGGTATTAAACTACCAGCAGAAGTTGGAAGTCAAGATGATGTTAACAATAGAAAATTGTTGCAAAAAGTGTTTGGTAAATTAATGAACACTGATGATGACGTTATAAAACAACAAACAGCTAAATTACTTGAAAGAACAGACAGAGAACCTCAAGTGTTCAAGGATATTGATTCTAGATTACCGGAGTTAATACAAAGATTAAACAAACAATTTCCTAATGACATCGGATTATTTTGTGGATGTCTCTTATTGAACCACGTTGATTGAAAAAATTATCAATTTCCGTGTTGGATAAACAGAGATTGACTGAAAAATTCAATAAATTGGATAAATCCATCAAAGATAATTTGAAGGCTAAACAAAAAGAAGAAACCAAAAAAACTTTAGATGTGGTTAATAATTGGTTGAATGATAAAGAAAATGCTTCATCATTTTTGGTTGCTCACGTTCCAATTACTGCTAATGCCAAGGCAATCACTGAAGCCATTAATTTGATTAAAAAGCAAGATAAAACCAAATCAATTTATTTATTGACTGGTGAAACCGATAAAGTTGCTCATGGATGTTATGTTAGTGATGAAGCCATTGCCAAGGGTATTAATGCGAAAATCAAACCTTTTGAAGCACGAGCAATTAACTGGTCCACGGATCTTAATGCTGAGGTACATATTGAGCATTATATAAATATATTCAATTATGCACGATCATCTTGGGAGCCATTGGTTGAAAGTTGGCCAATAGCAGTTTACATGTCAAAATCCCGACACCCAAAGCCTCAATTATTAGTAGAGGTGATTTCTAGACAGGTAGCYCAAGTGACGCTTACATCCAAAGCAGTAGCATTGCTATCTCAAGTATCCGATTTGATTACTTCCRGAGAAAAATTAAAACCAAGAGGTGAAGATTACCCATACGTKATAGTGAATGAGACTGGTTTAGATTTGGAAGTTTGGAATGATGCAAAWGAATYCGAAACCAATGGAGTTCCAATTGTTTTACGTGCTGGTAAAGCTTTAGATGAAAGTAAAGTTGAAATTAGAATTCAATTTAAACCAGTCGCCAAGGGGATGTTTAAAGAAATTCAAAGAAATGAATTAGTTATTAGAGTACAACCAAATGAAGCCATTTATTTAAAAATTAATTCCAAAATCCCTGGAATTTCTACTGAAACTTCATTAACTGATTTAGATTTAACTTATGCTACTCGTTATTCTAAAGAYTTTTGGATTCCTGAAGCTTATGAAGCATTAATTAGAGATTGYTATTTAGGTAATCATTCTAATTTTGTTAGAGATGATGAATTGGATGTTTCTTGGAAATTATTTACTCCATTATTGAAT

>FJ9:IND:54:12.6

AATTATTGAARTGGAATGATATTCCATTGGCTCCACCAGACAARATTTTGGGTATTTCTGAAGCTTATAACAATGATTCTAACCCTCAAAAARTCAATTTGGGGGTTGGTGCTTATAGAGATAATTCYGGTAAACCAATTATTTTCCCATCAGTTAAAAAAGCTGAAGAAATTTTATTGGGTAAAGAAACTGAAAAGGAATATACTGCCATTGTTGGTTCCAAAAATTTCCAATCAATTGTGAAAAATTTCATTTTCAACAATTCTAATAAAGATGCCAATGGTAAACAATTAATTGATGATGGTAGAATTGTTACTGCTCAAACCATCTCTGGTACTGGATCACTTAAACCTCTTATGGTGAAATTTTTGCTAAACATAGAGAACCAAATTTGGAAATTATTCGTGAGGTTGTTGATTCCAAACATATTGTTTTTGATGTGTTGGCACAATTCTTAATCAATCCAGACCCATGGGTTGCCATTGCTGCCGCTGAAGTTTATGTCAGACGTTCATACCGTGCTTATGATTTGGGTAMAATTGAATATCATGTTAATGACAGACTTCCTATTGTTGAATGGAAATTCAAGTTGGCTAATATGGGAGCCGCTGGTGTAAACGATGCTCAACAGGCTGCTGCTGCYGGTGGCGATGATTCGACATCTATGAAACATGCAGCTTCTGTGTCTGATTTGACCTTTGTTGTTGATTATTCATRAAAGCGAAATCCCAGGATACACTCTCCCCGATAATCCAAAGTTCACCCTTGGTAATTTGTTTGTAATAATTGGAGTCTTGTTGGTTTGTATTTTAGCTGTTGTCTCTCTTTTGAGAAATATTAGTGAGTCGGCCTTGTTCAAGAAGAATGGGTATGAACCGTTGGATTCGGATCCTAGTGTCATGAACCTAAACTTCGAGCCTACAACATTGTCCTTTGAAGATATTAAATATGAGGTTACTGGTGGTCGACAAATTTTAAATGGAGTCTTTGGGTTTGTAAAACCAAGAGAATGTTTGGCTATAATGGGAGGTTCAGGTGCTGGTAAAACTACATTGTTGGATTTTAAACCTTTGGACCAATTGGCTAARACTTTGRCYACTGTTCCTGAATTGAATGAAATTATTGGKCAAGAWTTAGTTGACGAATTTRTCAGTGGTATTAAACTACCAGCAGAAGTTGGAAGTCAAGATGATGTTAACAATAGAAAATTGTTGCAAAAAGTGTTTGGTAAATTAATGAACACTGATGATGACGTTATAAAACAACAAACAGCTAAATTACTTGAAAGAACAGACAGAGAACCTCAAGTGTTCAAGGATATTGATTCTAGATTACCGGAGTTAATACAAAGATTAAACAAACAATTTCCTAATGACATCGGATTATTTTGTGGATGTCTCTTATTGAACCACGTTGATTGAAAAAATTATCAATTTCCGTGTTGGATAAACAGAGATTGACTGAAAAATTCAATAAATTGGATAAATCCATCAAAGATAATTTGAAGGCTAAACAAAAAGAAGAAACCAAAAAAACTTTAGATGTGGTTAATAATTGGTTGAATGATAAAGAAAATGCTTCATCATTTTTGGTTGCTCACGTTCCAATTACTGCTAATGCCAAGGCAATCACTGAAGCCATTAATTTGATTAAAAAGCAAGATAAAACCAAATCAATTTATTTATTGACTGGTGAAACCGATAAAGTTGCTCATGGATGTTATGTTAGTGATGAAGCCATTGCCAAGGGTATTAATGCGAAAATCAAACCTTTTGAAGCACGAGCAATTAACTGGTCCACGGATCTTAATGCTGAGGTACATATTGAGCATTATATAAATATATTCAATTATGCACGATCATCTTGGGAGCCATTGGTTGAAAGTTGGCCAATAGCAGTTTACATGTCAAAATCCCGACACCCAAAGCCTCAATTATTAGTAGAGGTGATTTCTAGACAGGTAGCYCAAGTGACGCTTACATCCAAAGCAGTAGCATTGCTATCTCAAGTATCCGATTTGATTACTTCCRGAGAAAAATTAAAACCAAGAGGTGAAGATTACCCATACGTKATAGTGAATGAGACTGGTTTAGATTTGGAAGTTTGGAATGATGCAAAWGAATYCGAAACCAATGGAGTTCCAATTGTTTTACGTGCTGGTAAAGCTTTAGATGAAAGTAAAGTTGAAATTAGAATTCAATTTAAACCAGTCGCCAAGGGGATGTTTAAAGAAATTCAAAGAAATGAATTAGTTATTAGAGTACAACCAAATGAAGCCATTTATTTAAAAATTAATTCCAAAATCCCTGGAATTTCTACTGAAACTTCATTAACTGATTTAGATTTAACTTATGCTACTCGTTATTCTAAAGAYTTTTGGATTCCTGAAGCTTATGAAGCATTAATTAGAGATTGYTATTTAGGTAATCATTCTAATTTTGTTAGAGATGATGAATTGGATGTTTCTTGGAAATTATTTACTCCATTATTGAAT

>FJ9:IND:61:4.2

AATTATTGAARTGGAATGATATTCCATTGGCTCCACCAGACAARATTTTGGGTATTTCTGAAGCTTATAACAATGATTCTAACCCTCAAAAARTCAATTTGGGGGTTGGTGCTTATAGAGATAATTCYGGTAAACCAATTATTTTCCCATCAGTTAAAAAAGCTGAAGAAATTTTATTGGGTAAAGAAACTGAAAAGGAATATACTGCCATTGTTGGTTCCAAAAATTTCCAATCAATTGTGAAAAATTTCATTTTCAACAATTCTAATAAAGATGCCAATGGTAAACAATTAATTGATGATGGTAGAATTGTTACTGCTCAAACCATCTCTGGTACTGGATCACTTAAACCTCTTATGGTGAAATTTTTGCTAAACATAGAGAACCAAATTTGGAAATTATTCGTGAGGTTGTTGATTCCAAACATATTGTTTTTGATGTGTTGGCACAATTCTTAATCAATCCAGACCCATGGGTTGCCATTGCTGCCGCTGAAGTTTATGTCAGACGTTCATACCGTGCTTATGATTTGGGTAMAATTGAATATCATGTTAATGACAGACTTCCTATTGTTGAATGGAAATTCAAGTTGGCTAATATGGGAGCCGCTGGTGTAAACGATGCTCAACAGGCTGCTGCTGCYGGTGGCGATGATTCGACATCTATGAAACATGCAGCTTCTGTGTCTGATTTGACCTTTGTTGTTGATTATTCATRAAAGCGAAATCCCAGGATACACTCTCCCCGATAATCCAAAGTTCACCCTTGGTAATTTGTTTGTAATAATTGGAGTCTTGTTGGTTTGTATTTTAGCTGTTGTCTCTCTTTTGAGAAATATTAGTGAGTCGGCCTTGTTCAAGAAGAATGGGTATGAACCGTTGGATTCGGATCCTAGTGTCATGAACCTAAACTTCGAGCCTACAACATTGTCCTTTGAAGATATTAAATATGAGGTTACTGGTGGTCGACAAATTTTAAATGGAGTCTTTGGGTTTGTAAAACCAAGAGAATGTTTGGCTATAATGGGAGGTTCAGGTGCTGGTAAAACTACATTGTTGGATTTTAAACCTTTGGACCAATTGGCTAARACTTTGRCYACTGTTCCTGAATTGAATGAAATTATTGGKCAAGAWTTAGTTGACGAATTTRTCAGTGGTATTAAACTACCAGCAGAAGTTGGAAGTCAAGATGATGTTAACAATAGAAAATTGTTGCAAAAAGTGTTTGGTAAATTAATGAACACTGATGATGACGTTATAAAACAACAAACAGCTAAATTACTTGAAAGAACAGACAGAGAACCTCAAGTGTTCAAGGATATTGATTCTAGATTACCGGAGTTAATACAAAGATTAAACAAACAATTTCCTAATGACATCGGATTATTTTGTGGATGTCTCTTATTGAACCACGTTGATTGAAAAAATTATCAATTTCCGTGTTGGATAAACAGAGATTGACTGAAAAATTCAATAAATTGGATAAATCCATCAAAGATAATTTGAAGGCTAAACAAAAAGAAGAAACCAAAAAAACTTTAGATGTGGTTAATAATTGGTTGAATGATAAAGAAAATGCTTCATCATTTTTGGTTGCTCACGTTCCAATTACTGCTAATGCCAAGGCAATCACTGAAGCCATTAATTTGATTAAAAAGCAAGATAAAACCAAATCAATTTATTTATTGACTGGTGAAACCGATAAAGTTGCTCATGGATGTTATGTTAGTGATGAAGCCATTGCCAAGGGTATTAATGCGAAAATCAAACCTTTTGAAGCACGAGCAATTAACTGGTCCACGGATCTTAATGCTGAGGTACATATTGAGCATTATATAAATATATTCAATTATGCACGATCATCTTGGGAGCCATTGGTTGAAAGTTGGCCAATAGCAGTTTACATGTCAAAATCCCGACACCCAAAGCCTCAATTATTAGTAGAGGTGATTTCTAGACAGGTAGCYCAAGTGACGCTTACATCCAAAGCAGTAGCATTGCTATCTCAAGTATCCGATTTGATTACTTCCRGAGAAAAATTAAAACCAAGAGGTGAAGATTACCCATACGTKATAGTGAATGAGACTGGTTTAGATTTGGAAGTTTGGAATGATGCAAAWGAATYCGAAACCAATGGAGTTCCAATTGTTTTACGTGCTGGTAAAGCTTTAGATGAAAGTAAAGTTGAAATTAGAATTCAATTTAAACCAGTCGCCAAGGGGATGTTTAAAGAAATTCAAAGAAATGAATTAGTTATTAGAGTACAACCAAATGAAGCCATTTATTTAAAAATTAATTCCAAAATCCCTGGAATTTCTACTGAAACTTCATTAACTGATTTAGATTTAACTTATGCTACTCGTTATTCTAAAGAYTTTTGGATTCCTGAAGCTTATGAAGCATTAATTAGAGATTGYTATTTAGGTAATCATTCTAATTTTGTTAGAGATGATGAATTGGATGTTTCTTGGAAATTATTTACTCCATTATTGAAT

>FJ9:SC

AATTATTGAARTGGAATGATATTCCATTGGCTCCACCAGACAARATTTTGGGTATTTCTGAAGCTTATAACAATGATTCTAACCCTCAAAAARTCAATTTGGGGGTTGGTGCTTATAGAGATAATTCYGGTAAACCAATTATTTTCCCATCAGTTAAAAAAGCTGAAGAAATTTTATTGGGTAAAGAAACTGAAAAGGAATATACTGCCATTGTTGGTTCCAAAAATTTCCAATCAATTGTGAAAAATTTCATTTTCAACAATTCTAATAAAGATGCCAATGGTAAACAATTAATTGATGATGGTAGAATTGTTACTGCTCAAACCATCTCTGGTACTGGATCACTTAAACCTCTTATGGTGAAATTTTTGCTAAACATAGAGAACCAAATTTGGAAATTATTCGTGAGGTTGTTGATTCCAAACATATTGTTTTTGATGTGTTGGCACAATTCTTAATCAATCCAGACCCATGGGTTGCCATTGCTGCCGCTGAAGTTTATGTCAGACGTTCATACCGTGCTTATGATTTGGGTAMAATTGAATATCATGTTAATGACAGACTTCCTATTGTTGAATGGAAATTCAAGTTGGCTAATATGGGAGCCGCTGGTGTAAACGATGCTCAACAGGCTGCTGCTGCYGGTGGCGATGATTCGACATCTATGAAACATGCAGCTTCTGTGTCTGATTTGACCTTTGTTGTTGATTATTCATRAAAGCGAAATCCCAGGATACACTCTCCCCGATAATCCAAAGTTCACCCTTGGTAATTTGTTTGTAATAATTGGAGTCTTGTTGGTTTGTATTTTAGCTGTTGTCTCTCTTTTGAGAAATATTAGTGAGTCGGCCTTGTTCAAGAAGAATGGGTATGAACCGTTGGATTCGGATCCTAGTGTCATGAACCTAAACTTCGAGCCTACAACATTGTCCTTTGAAGATATTAAATATGAGGTTACTGGTGGTCGACAAATTTTAAATGGAGTCTTTGGGTTTGTAAAACCAAGAGAATGTTTGGCTATAATGGGAGGTTCAGGTGCTGGTAAAACTACATTGTTGGATTTTAAACCTTTGGACCAATTGGCTAARACTTTGRCYACTGTTCCTGAATTGAATGAAATTATTGGKCAAGAWTTAGTTGACGAATTTRTCAGTGGTATTAAACTACCAGCAGAAGTTGGAAGTCAAGATGATGTTAACAATAGAAAATTGTTGCAAAAAGTGTTTGGTAAATTAATGAACACTGATGATGACGTTATAAAACAACAAACAGCTAAATTACTTGAAAGAACAGACAGAGAACCTCAAGTGTTCAAGGATATTGATTCTAGATTACCGGAGTTAATACAAAGATTAAACAAACAATTTCCTAATGACATCGGATTATTTTGTGGATGTCTCTTATTGAACCACGTTGATTGAAAAAATTATCAATTTCCGTGTTGGATAAACAGAGATTGACTGAAAAATTCAATAAATTGGATAAATCCATCAAAGATAATTTGAAGGCTAAACAAAAAGAAGAAACCAAAAAAACTTTAGATGTGGTTAATAATTGGTTGAATGATAAAGAAAATGCTTCATCATTTTTGGTTGCTCACGTTCCAATTACTGCTAATGCCAAGGCAATCACTGAAGCCATTAATTTGATTAAAAAGCAAGATAAAACCAAATCAATTTATTTATTGACTGGTGAAACCGATAAAGTTGCTCATGGATGTTATGTTAGTGATGAAGCCATTGCCAAGGGTATTAATGCGAAAATCAAACCTTTTGAAGCACGAGCAATTAACTGGTCCACGGATCTTAATGCTGAGGTACATATTGAGCATTATATAAATATATTCAATTATGCACGATCATCTTGGGAGCCATTGGTTGAAAGTTGGCCAATAGCAGTTTACATGTCAAAATCCCGACACCCAAAGCCTCAATTATTAGTAGAGGTGATTTCTAGACAGGTAGCYCAAGTGACGCTTACATCCAAAGCAGTAGCATTGCTATCTCAAGTATCCGATTTGATTACTTCCRGAGAAAAATTAAAACCAAGAGGTGAAGATTACCCATACGTKATAGTGAATGAGACTGGTTTAGATTTGGAAGTTTGGAATGATGCAAAWGAATYCGAAACCAATGGAGTTCCAATTGTTTTACGTGCTGGTAAAGCTTTAGATGAAAGTAAAGTTGAAATTAGAATTCAATTTAAACCAGTCGCCAAGGGGATGTTTAAAGAAATTCAAAGAAATGAATTAGTTATTAGAGTACAACCAAATGAAGCCATTTATTTAAAAATTAATTCCAAAATCCCTGGAATTTCTACTGAAACTTCATTAACTGATTTAGATTTAACTTATGCTACTCGTTATTCTAAAGAYTTTTGGATTCCTGAAGCTTATGAAGCATTAATTAGAGATTGYTATTTAGGTAATCATTCTAATTTTGTTAGAGATGATGAATTGGATGTTTCTTGGAAATTATTTACTCCATTATTGAAT

>Fj12:IND:8:6.7

AATTATTGAAATGGAATGATATTCCATTGGCTCCACCAGACAAGATTTTGGGTATTTCTGAAGCTTATAACAATGATTCTAACCCTCAAAAAGTCAATTTGGGGGTTGGTGCTTATAGAGATAATTCTGGTAAACCAATTATTTTCCCATCAGTTAAAAAAGCTGAAGAAATTTTATTGGGTAAAGAAACTGAAAAGGAATATACTGCCATTGTTGGTTCCAAAAATTTCCAATCAATTGTGAAAAATTTCATTTTCAACAATTCTAATAAAGATGCCAATGGTAAACAATTAATTGATGATGGTAGAATTGTTACTGCTCAAACCATCTCTGGTACTGGATCACTTAAACCTCTTATGGTGAAATTTTTGCTAAACATAGAGAACCAAATTTGGAAATTATTCGTGAGGTTGTTGATTCCAAACATATTGTTTTTGATGTGTTGGCACAATTCTTAATCAATCCAGACCCATGGGTTGCCATTGCTGCCGCTGAAGTTTATGTCAGACGTTCATACCGTGCTTATGATTTGGGTAAAATTGAATATCATGTTAATGACAGACTTCCTATTGTTGAATGGAAATTCAAGTTGGCTAATATGGGAGCCGCTGGTGTAAACGATGCTCAACAGGCTGCTGCTGCCGGTGGCGATGATTCGACATCTATGAAACATGCAGCTTCTGTGTCTGATTTGACCTTTGTTGTTGATTATTCATAAAAGTGAAATTCCAGGATACACTCTCCCCGATAATCCAAAGTTCACCCTTGGTAATTTGTTTGTAATAATTGGGGTCTTGTTGGTTTGTATTTTAGCTGTTGTCTCTCTTTTGAGAAATATTAGTGAGTCAGCCTTGTTCAAGAAGAATGGGTATGAACCGTTGGATTCAGATCCTAGTGTCATGAACCAAAACTTTGAGCCTACAACATTGTCCTTTGAAGATATTAAATATGAGGTTACTGGTGGTCGACAAATTTTAAATGGAGTCTTTGGGTTTGTAAAACCAAGAGAATGTTTGGCTATAATGGGAGGTTCAGGTGCTGGTAAAACTACATTGTTGGATTTTAAACCTTTGGACCAATTGGCTAAAACTTTGRCYACTGTTCCTGAATTGAATGAAATTATTGGKCAAGAATTAGTTGACGAATTTRTCAGTGGTATTAAACTACCAGCAGAAGTTGGAAGTCAAGATGATGTTAACAATAGAAAATTGTTGCAAAAAGTGTTTGGTAAATTAATGAACACTGATGATGACGTTATAAAACAACAAACAGCTAAATTACTTGAAAGAACAGACAGAGAACCTCAAGTGTTCAAGGATATTGATTCTAGATTACCGGAGTTAATACAAAGATTAAACAAACAATTTCCTAATGACATCGGATTATTTTGTGGATGTCTCTTATTGAACCACGTTGATTGAAAAAATTATCAATTTCCGTGTTGGATAAACAGAGATTGACTGAAAAATTCAATAAATTGGATAAATCCATCAAAGATAATTTGAAGGCTAAACAAAAAGAAGAAACCAAAAAAACTTTAGATGTGGTTAATAATTGGTTGAATGATAAAGAAAATGCTTCATCATTTTTGGTTGCTCACGTTCCAATTACTGCTAATGCCAAGGCAATCACTGAAGCCATTAATTTGATTAAAAAGCAAGATAAAACCAAATCAATTTATTTATTGACTGGTGAAACCGATAAAGTTGCTCATGGATGTTATGTTAGTGATGAAGCCATTGCCAAGGGTATTAATGCGAAAATCAAACCTTTTGAAGCACGAGCAATTAACTGGTCCACGGATCTTAATGCTGAGGTACATATTGAGCATTATATAAATATATTCAATTATGCACGATCATCTTGGGAGCCATTGGTTGAAAGTTGGCCAATAGCAGTTTACATGTCAAAATCCCGACACCCAAAGCCTCAATTATTAGTAGAGGTGATTTCTAGACAGGTAGCTCAAGTGACGCTTACATCCAAAGCAGTAGCATTGCTATCTCAAGTATCCGATTTGATTACTTCCRGAGAAAAATTAAAACCAAGAGGTGAAGATTACCCATACGTTATAGTGAATGAGACTGGTTTAGATTTGGAAGTTTGGAATGATGCAAAWGAATCCGAAACCAATGGAGTTCCAATTGTTTTACGTGCTGGTAAAGCTTTAGATGAAAGTAAAGTTGAAATTAGAATTCAATTTAAACCAGTCGCCAAGGGGATGTTTAAAGAAATTCAAAGAAATGAATTAGTTATTAGAGTACAACCAAATGAAGCCATTTATTTAAAAATTAATTCCAAAATCCCTGGAATTTCTACTGAAACTTCATTAACTGATTTAGATTTAACTTATGCTACTCGTTATTCTAAAGAYTTTTGGATTCCTGAAGCTTATGAAGCATTAATTAGAGATTGTTATTTAGGTAATCATTCTAATTTTGTTAGAGATGATGAATTGGATGTTTCTTGGAAATTATTTACTCCATTATTGAAT

>Fj12:IND:24:10.3

AATTATTGAARTGGAATGATATTCCATTGGCTCCACCAGACAAGATTTTGGGTATTTCTGAAGCTTATAACAAYGATTCTAACCCTCAAAAAGTCAATTTGGGGGTTGGTGCTTATAGAGATAATTCYGGTAAACCAATTATTTTCCCATCAGTTAAAAAAGCTGAAGAAATTTTATTGGGTAAAGAAACTGAAAAGGAATATACTGCCATTGTTGGTTCCAAAAATTTCCAATCAATTGTGAAAAATTTCATTTTCAACAATTCTAATAAAGATGCCAATGGTAAACAATTAATTGATGATGGTAGAATTGTTACTGCTCAAACCATCTCTGGTACTGGATCACTTAAACCTCTTATGGTGAAATTTTTGCTAAACATAGAGAACCAAATTTGGAAATTATTCGTGAGGTTGTTGATTCCAAACATATTGTTTTTGATGTGTTGGCACAATTCTTAATCAATCCAGACCCATGGGTTGCCATTGCTGCCGCTGAAGTTTATGTCAGACGTTCATACCGTGCTTATGATTTGGGTAAAATTGAATATCATGTTAATGACAGACTTCCTATTGTTGAATGGAAATTCAAGTTGGCTAATATGGGAGCCGCTGGTGTAAACGATGCTCAACAGGCTGCTGCTGCCGGTGGCGATGATTCGACATCTATGAAACATGCAGCTTCTGTGTCTGATTTGACCTTTGTTGTTGATTATTCATAAAAGTGAAATTCCAGGATACACTCTCCCCGATAATCCAAAGTTCACCCTTGGTAATTTGTTTGTAATAATTGGGGTCTTGTTGGTTTGTRTTTTAGCTGTTGTCTCTCTTTTGAGAAATATTAGTGAGTCAGCCTTGTTCAAGAAGAATGGGTATGAACCGTTGGATTCAGATCCTAGTGTCATGAACCAAAACTTTGAGCCTACAACATTGTCCTTTGAAGATATTAAATATGAGGTTACTGGTGGTCGACAAATTTTAAATGGAGTCTTTGGGTTTGTAAAACCAAGAGAATGTTTGGCTATAATGGGAGGTTCAGGTGCTGGTAAAACTACATTGTTGGATTTTAAACCTTTGGACCAATTGGCTAAAACTTTGGCTACTGTTCCTGAATTGAATGAAATTATTGGTCAAGAATTAGTTGACGAATTTRTCAGTGGTATTAAACTACCAGCAGAAGTTGGAAGTCAAGATGATGTTAACAATAGAAAATTGTTGCAAAAAGTGTTTGGTAAATTAATGAACACTGATGATGACGTTATAAAACAACAAACAGCTAAATTACTTGAAAGAACAGACAGAGAACCTCAAGTGTTCAAGGATATTGATTCTAGATTACCGGAGTTAATACAAAGATTAAACAAACAATTTCCTAATGACATCGGATTATTTTGTGGATGTCTCTTATTGAACCACGTTGATTGAAAAAATTATCAATTTCCGTGTTGGATAAACARAGATTGACTGAAAAATTCAATAAATTGGATAAATCCATYAAAGATAATTTGAAGGCTAAACAAAAAGAAGAAACCAAAAARACTTTAGATGTGGTTAAYAATTGGTTGAATGATAAAGAAAATGCTTCATCATTTTTGGTTGCTCACGTTCCAATTACTGCTAATGCCAAGGCAATCACTGAAGCCATTAATTTGATTAAAAAGCAAGATAAAACCAAATCAATTTATTTATTGACTGGTGAAACCGATAAAGTTGCTCATGGATGTTATGTTAGTGATGAAGCCATTGCCAAGGGTATTAATGCGAAAATCAAACCTTTTGAAGCACGAGCAATTAACTGGTCMACGGATCTTAATGCTGAGGTACATATTGAGCATTATATAAATATATTCAATTATGCACGATCATCTTGGGAGCCATTGGTTGAAAGTTGGCCAATAGCAGTTTACATGTCAAAATCCCGACACCCAAAGCCTCAATTATTAGTAGAGGTGATTTCTAGACAGGTAGCTCAAGTGACGCTTACATCCAAAGCAGTAGCATTGCTATCTCAAGTATCCGATTTGATTACTTCCRGAGAAAAATTAAAACCAAGAGGTGAAGATTACCCATACRTTATAGTGAATGAGACTGGTTTAGATTTGGAAGTTTGGAATGATGCAAAWGAATCCGAAACCAATGGAGTTCCAATTGTTTTACGTGCTGGTAAAGCTTTAGATGAAAGTAAAGTTGAAATTAGAATTCAATTTAAACCAGTYGCCAAGGGGATGTTTAAAGAAATTCAAAGAAATGAATTAGTTATTAGAGTACAACCAAATGAAGCCATTTATTTAAAAATTAATTCMAAAATCCCTGGRATTTCTACTGAAACTTCATTAACTGATTTAGATTTAACTTATGCTACTCGTTATTCTAAAGAYTTTTGGATTCCTGAAGCTTATGAAGCATTAATTAGAGATTGTTATTTAGGTAATCATTCTAATTTTGTTAGAGATGATGAATTGGATGTTTCTTGGAAATTATTTACTCCATTATTGAAT

>Fj12:IND:49:6.0

AATTATTGAARTGGAATGATATTCCATTGGCTCCACCAGACAAGATTTTGGGTATTTCTGAAGCTTATAACAAYGATTCTAACCCTCAAAAAGTCAATTTGGGGGTTGGTGCTTATAGAGATAATTCYGGTAAACCAATTATTTTCCCATCAGTTAAAAAAGCTGAAGAAATTTTATTGGGTAAAGAAACTGAAAAGGAATATACTGCCATTGTTGGTTCCAAAAATTTCCAATCAATTGTGAAAAATTTCATTTTCAACAATTCTAATAAAGATGCCAATGGTAAACAATTAATTGATGATGGTAGAATTGTTACTGCTCAAACCATCTCTGGTACTGGATCACTTAAACCTCTTATGGTGAAATTTTTGCTAAACATAGAGAACCAAATTTGGAAATTATTCGTGAGGTTGTTGATTCCAAACATATTGTTTTTGATGTGTTGGCACAATTCTTAATCAATCCAGACCCATGGGTTGCCATTGCTGCCGCTGAAGTTTATGTCAGACGTTCATACCGTGCTTATGATTTGGGTAAAATTGAATATCATGTTAATGACAGACTTCCTATTGTTGAATGGAAATTCAAGTTGGCTAATATGGGAGCCGCTGGTGTAAACGATGCTCAACAGGCTGCTGCTGCCGGTGGCGATGATTCGACATCTATGAAACATGCAGCTTCTGTGTCTGATTTGACCTTTGTTGTTGATTATTCATAAAAGTGAAATTCCAGGATACACTCTCCCCGATAATCCAAAGTTCACCCTTGGTAATTTGTTTGTAATAATTGGGGTCTTGTTGGTTTGTRTTTTAGCTGTTGTCTCTCTTTTGAGAAATATTAGTGAGTCAGCCTTGTTCAAGAAGAATGGGTATGAACCGTTGGATTCAGATCCTAGTGTCATGAACCAAAACTTTGAGCCTACAACATTGTCCTTTGAAGATATTAAATATGAGGTTACTGGTGGTCGACAAATTTTAAATGGAGTCTTTGGGTTTGTAAAACCAAGAGAATGTTTGGCTATAATGGGAGGTTCAGGTGCTGGTAAAACTACATTGTTGGATTTTAAACCTTTGGACCAATTGGCTAAAACTTTGGCTACTGTTCCTGAATTGAATGAAATTATTGGTCAAGAATTAGTTGACGAATTTRTCAGTGGTATTAAACTACCAGCAGAAGTTGGAAGTCAAGATGATGTTAACAATAGAAAATTGTTGCAAAAAGTGTTTGGTAAATTAATGAACACTGATGATGACGTTATAAAACAACAAACAGCTAAATTACTTGAAAGAACAGACAGAGAACCTCAAGTGTTCAAGGATATTGATTCTAGATTACCGGAGTTAATACAAAGATTAAACAAACAATTTCCTAATGACATCGGATTATTTTGTGGATGTCTCTTATTGAACCACGTTGATTGAAAAAATTATCAATTTCCGTGTTGGATAAACARAGATTGACTGAAAAATTCAATAAATTGGATAAATCCATYAAAGATAATTTGAAGGCTAAACAAAAAGAAGAAACCAAAAARACTTTAGATGTGGTTAAYAATTGGTTGAATGATAAAGAAAATGCTTCATCATTTTTGGTTGCTCACGTTCCAATTACTGCTAATGCCAAGGCAATCACTGAAGCCATTAATTTGATTAAAAAGCAAGATAAAACCAAATCAATTTATTTATTGACTGGTGAAACCGATAAAGTTGCTCATGGATGTTATGTTAGTGATGAAGCCATTGCCAAGGGTATTAATGCGAAAATCAAACCTTTTGAAGCACGAGCAATTAACTGGTCMACGGATCTTAATGCTGAGGTACATATTGAGCATTATATAAATATATTCAATTATGCACGATCATCTTGGGAGCCATTGGTTGAAAGTTGGCCAATAGCAGTTTACATGTCAAAATCCCGACACCCAAAGCCTCAATTATTAGTAGAGGTGATTTCTAGACAGGTAGCTCAAGTGACGCTTACATCCAAAGCAGTAGCATTGCTATCTCAAGTATCCGATTTGATTACTTCCRGAGAAAAATTAAAACCAAGAGGTGAAGATTACCCATACRTTATAGTGAATGAGACTGGTTTAGATTTGGAAGTTTGGAATGATGCAAAWGAATCCGAAACCAATGGAGTTCCAATTGTTTTACGTGCTGGTAAAGCTTTAGATGAAAGTAAAGTTGAAATTAGAATTCAATTTAAACCAGTYGCCAAGGGGATGTTTAAAGAAATTCAAAGAAATGAATTAGTTATTAGAGTACAACCAAATGAAGCCATTTATTTAAAAATTAATTCMAAAATCCCTGGRATTTCTACTGAAACTTCATTAACTGATTTAGATTTAACTTATGCTACTCGTTATTCTAAAGAYTTTTGGATTCCTGAAGCTTATGAAGCATTAATTAGAGATTGTTATTTAGGTAATCATTCTAATTTTGTTAGAGATGATGAATTGGATGTTTCTTGGAAATTATTTACTCCATTATTGAAT

>FJ12:SC

AATTATTGAAGTGGAATGATATTCCATTGGCTCCACCAGACAAAATTTTGGGTATTTCTGAAGCTTATAACAATGATTCTAACCCTCAAAAAATCAATTTGGGGGTTGGTGCTTATAGAGATAATTCCGGTAAACCAATTATTTTCCCATCAGTTAAAAAAGCTGAAGAAATTTTATTGGGTAAAGAAACTGAAAAGGAATATACTGCCATTGTTGGTTCCAAAAATTTCCAATCAATTGTGAAAAATTTCATTTTCAACAATTCTAATAAAGATGCCAATGGTAAACAATTAATTGATGATGGTAGAATTGTTACTGCTCAAACCATCTCTGGTACTGGATCACTTAAACCTCTTATGGTGAAATTTTTGCTAAACATAGAGAACCAAATTTGGAAATTATTCGTGAGGTTGTTGATTCCAAACATATTGTTTTTGATGTGTTGGCACAATTCTTAATCAATCCAGACCCATGGGTTGCCATTGCTGCCGCTGAAGTTTATGTCAGACGTTCATACCGTGCTTATGATTTGGGTAAAATTGAATATCATGTTAATGACAGACTTCCTATTGTTGAATGGAAATTCAAGTTGGCTAATATGGGAGCCGCTGGTGTAAACGATGCTCAACAGGCTGCTGCTGCCGGTGGCGATGATTCGACATCTATGAAACATGCAGCTTCTGTGTCTGATTTGACCTTTGTTGTTGATTATTCATAAAAGTGAAATTCCAGGATACACTCTCCCCGATAATCCAAAGTTCACCCTTGGTAATTTGTTTGTAATAATTGGGGTCTTGTTGGTTTGTRTTTTAGCTGTTGTCTCTCTTTTGAGAAATATTAGTGAGTCAGCCTTGTTCAAGAAGAATGGGTATGAACCGTTGGATTCAGATCCTAGTGTCATGAACCAAAACTTTGAGCCTACAACATTGTCCTTTGAAGATATTAAATATGAGGTTACTGGTGGTCGACAAATTTTAAATGGAGTCTTTGGGTTTGTAAAACCAAGAGAATGTTTGGCTATAATGGGAGGTTCAGGTGCTGGTAAAACTACATTGTTGGATTTTAAACCTTTGGACCAATTGGCTAAAACTTTGGCTACTGTTCCTGAATTGAATGAAATTATTGGTCAAGAATTAGTTGACGAATTTRTCAGTGGTATTAAACTACCAGCAGAAGTTGGAAGTCAAGATGATGTTAACAATAGAAAATTGTTGCAAAAAGTGTTTGGTAAATTAATGAACACTGATGATGACGTTATAAAACAACAAACAGCTAAATTACTTGAAAGAACAGACAGAGAACCTCAAGTGTTCAAGGATATTGATTCTAGATTACCGGAGTTAATACAAAGATTAAACAAACAATTTCCTAATGACATCGGATTATTTTGTGGATGTCTCTTATTGAACCACGTTGCTTGAAAAAATTATCAATTTCCGTGTTGGATAAACARAGATTGACTGAAAAATTCAATAAATTGGATAAATCCATYAAAGATAATTTGAAGGCTAAACAAAAAGAAGAAACCAAAAARACTTTAGATGTGGTTAAYAATTGGTTGAATGATAAAGAAAATGCTTCATCATTTTTGGTTGCTCACGTTCCAATTACTGCTAATGCCAAGGCAATCACTGAAGCCATTAATTTGATTAAAAAGCAAGATAAAACCAAATCAATTTATTTATTGACTGGTGAAACCGATAAAGTTGCTCATGGATGTTATGTTAGTGATGAAGCCATTGCCAAGGGTATTAATGCGAAAATCAAACCTTTTGAAGCACGAGCAATTAACTGGTCMACGGATCTTAATGCTGAGGTACATATTGAGCATTATATAAATATATTCAATTATGCACGATCATCTTGGGAGCCATTGGTTGAAAGTTGGCCAATAGCAGTTTACATGTCAAAATCCCGACACCCAAAGCCTCAATTATTAGTAGAGGTGATTTCTAGACAGGTAGCTCAAGTGACGCTTACATCCAAAGCAGTAGCATTGCTATCTCAAGTATCCGATTTGATTACTTCCRGAGAAAAATTAAAACCAAGAGGTGAAGATTACCCATACRTTATAGTGAATGAGACTGGTTTAGATTTGGAAGTTTGGAATGATGCAAAWGAATCCGAAACCAATGGAGTTCCAATTGTTTTACGTGCTGGTAAAGCTTTAGATGAAAGTAAAGTTGAAATTAGAATTCAATTTAAACCAGTYGCCAAGGGGATGTTTAAAGAAATTCAAAGAAATGAATTAGTTATTAGAGTACAACCAAATGAAGCCATTTATTTAAAAATTAATTCMAAAATCCCTGGRATTTCTACTGAAACTTCATTAACTGATTTAGATTTAACTTATGCTACTCGTTATTCTAAAGAYTTTTGGATTCCTGAAGCTTATGAAGCATTAATTAGAGATTGTTATTTAGGTAATCATTCTAATTTTGTTAGAGATGATGAATTGGATGTTTCTTGGAAATTATTTACTCCATTATTGAAT

>FJ26

AATTATTGAARTGGAATGATATTCCATTGGCTCCACCAGACAARATTTTGGGTATTTCTGAAGCTTATAACAATGATTCTAACCCTCAAAAARTCAATTTGGGGGTTGGTGCTTATAGAGATAATTCYGGTAAACCAATTATTTTCCCATCAGTTAAAAAAGCTGAAGAAATTTTATTGGGTAAAGAAACTGAAAAGGAATATACTGCCATTGTTGGTTCCAAAAATTTCCAATCAATTGTGAAAAATTTCATTTTCAACAATTCTAATAAAGATGCCAATGGTAAACAATTAATTGATGATGGTAGAATTGTTACTGCTCAAACCATCTCTGGTACTGGATCACTTAAACCTCTTATGGTGAAATTTTTGCTAAACATAGAGAACCAAATTTGGAAATTATTCGTGAGGTTGTTGATTCCAAACATATTGTTTTTGATGTGTTGGCACAATTCTTAATCAATCCAGACCCATGGGTTGCCATTGCTGCCGCTGAAGTTTATGTCAGACGTTCATACCGTGCTTATGATTTGGGTAAAATTGAATATCATGTTAATGACAGACTTCCTATTGTTGAATGGAAATTCAAGTTGGCTAATATGGGAGCCGCTGGTGTAAACGATGCTCAACAGGCTGCTGCTGCCGGTGGCGATGATTCGACATCTATGAAACATGCAGCTTCTGTGTCTGATTTGACCTTTGTTGTTGATTATTCATRAAAGCGAAATCCCAGGATACACTCTCCCCGATAATCCAAAGTTCACCCTTGGTAATTTGTTTGTAATAATTGGAGTCTTGTTGGTTTGTATTTTAGCTGTTGTCTCTCTTTTGAGAAATATTAGTGAGTCGGCCTTGTTCAAGAAGAATGGGTATGAACCGTTGGATTCGGATCCTAGTGTCATGAACCTAAACTTCGAGCCTACAACATTGTCCTTTGAAGATATTAAATATGAGGTTACTGGTGGTCGACAAATTTTAAATGGAGTCTTTGGGTTTGTAAAACCAAGAGAATGTTTGGCTATAATGGGAGGTTCAGGTGCTGGTAAAACTACATTGTTGGATTTTAAACCTTTGGACCAATTGGCTAARACTTTGRCYACTGTTCCTGAATTGAATGAAATTATTGGKCAAGAWTTAGTTGACGAATTTRTCAGTGGTATTAAACTACCAGCAGAAGTTGGAAGTCAAGATGATGTTAACAATAGAAAATTGTTGCAAAAAGTGTTTGGTAAATTAATGAACACTGATGATGACGTTATAAAACAACAAACAGCTAAATTACTTGAAAGAACAGACAGAGAACCTCAAGTGTTCAAGGATATTGATTCTAGATTACCGGAGTTAATACAAAGATTAAACAAACAATTTCCTAATGACATCGGATTATTTTGTGGATGTCTCTTATTGAACCACGTTGATTGAAAAAATTATCAATTTCCGTGTTGGATAAACAGAGATTGACTGAAAAATTCAATAAATTGGATAAATCCATCAAAGATAATTTGAAGGCTAAACAAAAAGAAGAAACCAAAAAAACTTTAGATGTGGTTAATAATTGGTTGAATGATAAAGAAAATGCTTCATCATTTTTGGTTGCTCACGTTCCAATTACTGCTAATGCCAAGGCAATCACTGAAGCCATTAATTTGATTAAAAAGCAAGATAAAACCAAATCAATTTATTTATTGACTGGTGAAACCGATAAAGTTGCTCATGGATGTTATGTTAGTGATGAAGCCATTGCCAAGGGTATTAATGCGAAAATCAAACCTTTTGAAGCACGAGCAATTAACTGGTCCACGGATCTTAATGCTGAGGTACATATTGAGCATTATATAAATATATTCAATTATGCACGATCATCTTGGGAGCCATTGGTTGAAAGTTGGCCAATAGCAGTTTACATGTCAAAATCCCGACACCCAAAGCCTCAATTATTAGTAGAGGTGATTTCTAGACAGGTAGCYCAAGTGACGCTTACATCCAAAGCAGTAGCATTGCTATCTCAAGTATCCGATTTGATTACTTCCRGAGAAAAATTAAAACCAAGAGGTGAAGATTACCCATACGTKATAGTGAATGAGACTGGTTTAGATTTGGAAGTTTGGAATGATGCAAAWGAATYCGAAACCAATGGAGTTCCAATTGTTTTACGTGCTGGTAAAGCTTTAGATGAAAGTAAAGTTGAAATTAGAATTCAATTTAAACCAGTCGCCAAGGGGATGTTTAAAGAAATTCAAAGAAATGAATTAGTTATTAGAGTACAACCAAATGAAGCCATTTATTTAAAAATTAATTCCAAAATCCCTGGAATTTCTACTGAAACTTCATTAACTGATTTAGATTTAACTTATGCTACTCGTTATTCTAAAGAYTTTTGGATTCCTGAAGCTTATGAAGCATTAATTAGAGATTGYTATTTAGGTAATCATTCTAATTTTGTTAGAGATGATGAATTGGATGTTTCTTGGAAATTATTTACTCCATTATTGAAT

>gaymc_c

AATTATTGAAATGGAATGATATTCCATTGGCTCCACCAGACAAAATTTTGGGTATTTCTGAAGCTTATAACAACGATTCTAACCCTCAAAAAATCAATTTGGGGGTTGGTGCTTATAGAGATAATTCTGGTAAACCAATTATTTTCCCATCAGTTAAAAAAGCTGAAGAAATTTTATTGGGTAAAGAAACTGAAAAGGAATATACTGCCATTGTTGGTTCCAAAAATTTCCAATCAATTGTGAAAAATTTCATTTTCAACAATTCTAATAAAGATGCCAATGGTAAACAATTAATTGATGATGGTAGAATTGTTACTGCTCAAACCATCTCTGGTACTGGATCACTTAAACCTCTTATGGTGAAATTTTTGCTAAACATAGAGAACCAAATTTGGAAATTATTCGTGAGGTTGTTGATTCCAAACATATTGTTTTTGATGTGTTGGCACAATTCTTAATCAATCCAGACCCATGGGTTGCCATTGCTGCCGCTGAAGTTTATGTCAGACGTTCATACCGTGCTTATGATTTGGGTACAATTGAATATCATGTTAATGACAGACTTCCTATTGTTGAATGGAAATTCAAGTTGGCTAATATGGGAGCCGCTGGTGTAAACGATGCTCAACAGGCTGCTGCTGCTGGTGGCGATGATTCGACATCTATGAAACATGCAGCTTCTGTGTCTGATTTGACCTTTGTTGTTGATTATTCATAAAAGCGAAATCCCAGGATACACTCTCCCCGATAATCCAAAGTTCACCCTTGGTAATTTGTTTGTAATAATTGGAGTCTTGTTGGTTTGTATTTTAGCTGTTGTCTCTCTTTTGAGAAATATTAGTGAGTCGGCCTTGTTCAAGAAGAATGGGTATGAACCGTTGGATTCGGATCCTAGTGTCATGAACCTAAACTTCGAGCCTACAACATTGTCCTTTGAAGATATTAAATATGAGGTTACTGGTGGTCGACAAATTTTAAATGGAGTCTTTGGGTTTGTAAAACCAAGAGAATGTTTGGCTATAATGGGAGGTTCAGGTGCTGGTAAAACTACATTGTTGGATTTTAAACCTTTGGACCAATTGGCTAARACTTTGRCYACTGTTCCTGAATTGAATGAAATTATTGGKCAAGAWTTAGTTGACGAATTTRTCAGTGGTATTAAACTACCAGCAGAAGTTGGAAGTCAAGATGATGTTAACAATAGAAAATTGTTGCAAAAAGTGTTTGGTAAATTAATGAACACTGATGATGACGTTATAAAACAACAAACAGCTAAATTACTTGAAAGAACAGACAGAGAACCTCAAGTGTTCAAGGATATTGATTCTAGATTACCGGAGTTAATACAAAGATTAAACAAACAATTTCCTAATGACATCGGATTATTTTGTGGATGTCTCTTATTGAACCACGTTGCTTGAAAAAATTATCAATTTCCGTGTTGGATAAACAGAGATTGACTGAAAAATTCAATAAATTGGATAAATCCATTAAAGATAATTTGAAGGCTAAACAAAAAGAAGAAACCAAAAAGACTTTAGATGTGGTTAACAATTGGTTGAATGATAAAGAAAATGCTTCATCATTTTTGGTTGCTCACGTTCCAATTACTGCTAATGCCAAGGCAATCACTGAAGCCATTAATTTGATTAAAAAGCAAGATAAAACCAAATCAATTTATTTATTGACTGGTGAAACCGATAAAGTTGCTCATGGATGTTATGTTAGTGATGAAGCCATTGCCAAGGGTATTAATGCGAAAATCAAACCTTTTGAAGCACGAGCAATTAACTGGTCCACGGATCTTAATGCTGAGGTACATATTGAGCATTATATAAATATATTCAATTATGCACGATCATCTTGGGAGCCATTGGTTGAAAGTTGGCCAATAGCAGTTTACATGTCAAAATCCCGACACCCAAAGCCTCAATTATTAGTAGAGGTGATTTCTAGACAGGTAGCTCAAGTGACGCTTACATCCAAAGCAGTAGCATTGCTATCTCAAGTATCCGATTTGATTACTTCCRGAGAAAAATTAAAACCAAGAGGTGAAGATTACCCATACGTKATAGTGAATGAGACTSGTTTAGATTTGGAAGTTTGGAATGATGCAAAWGAATYCGAAACCAATGGAGTTCCAATTGTTTTACGTGCTGGTAAAGCTTTAGATGAAAGTAAAGTTGAAATTAGAATTCAATTTAAACCAGTCGCCAAGGGGATGTTTAAAGAAATTCAAAGAAATGAATTAGTTATTAGAGTACAACCAAATGAAGCCATTTATTTAAAAATTAATTCCAAAATCCCTGGAATTTCTACTGAAACTTCATTAACTGATTTAGATTTAACTTATGCTACTCGTTATTCTAAAGAYTTTTGGATTCCTGAAGCTTATGAAGCATTAATTAGAGATTGYTATTTAGGTAATCATTCTAATTTTGTTAGAGATGATGAATTGGATGTTTCTTGGAAATTATTTACTCCATTATTGAAT

>gaymc_c:IND:51:110.6

AATTATTGAAATGGAATGATATTCCATTGGCTCCACCAGACAAGATTTTGGGTATTTCTGAAGCTTATAACAACGATTCTAACCCTCAAAAAGTCAATTTGGGGGTTGGTGCTTATAGAGATAATTCTGGTAAACCAATTATTTTCCCATCAGTTAAAAAAGCTGAAGAAATTTTATTGGGTAAAGAAACTGAAAAGGAATATACTGCCATTGTTGGTTCCAAAAATTTCCAATCAATTGTGAAAAATTTCATTTTCAACAATTCTAATAAAGATGCCAATGGTAAACAATTAATTGATGATGGTAGAATTGTTACTGCTCAAACCATCTCTGGTACTGGATCACTTAAACCTCTTATGGTGAAATTTTTGCTAAACATAGAGAACCAAATTTGGAAATTATTCGTGAGGTTGTTGATTCCAAACATATTGTTTTTGATGTGTTGGCACAATTCTTAATCAATCCAGACCCATGGGTTGCCATTGCTGCCGCTGAAGTTTATGTCAGACGTTCATACCGTGCTTATGATTTGGGTACAATTGAATATCATGTTAATGACAGACTTCCTATTGTTGAATGGAAATTCAAGTTGGCTAATATGGGAGCCGCTGGTGTAAACGATGCTCAACAGGCTGCTGCTGCTGGTGGCGATGATTCGACATCTATGAAACATGCAGCTTCTGTGTCTGATTTGACCTTTGTTGTTGATTATTCATAAAAGCGAAATCCCAGGATACACTCTCCCCGATAATCCAAAGTTCACCCTTGGTAATTTGTTTGTAATAATTGGAGTCTTGTTGGTTTGTATTTTAGCTGTTGTCTCTCTTTTGAGAAATATTAGTGAGTCGGCCTTGTTCAAGAAGAATGGGTATGAACCGTTGGATTCGGATCCTAGTGTCATGAACCTAAACTTCGAGCCTACAACATTGTCCTTTGAAGATATTAAATATGAGGTTACTGGTGGTCGACAAATTTTAAATGGAGTCTTTGGGTTTGTAAAACCAAGAGAATGTTTGGCTATAATGGGAGGTTCAGGTGCTGGTAAAACTACATTGTTGGATTTTAAACCTTTGGACCAATTGGCTAARACTTTGRCYACTGTTCCTGAATTGAATGAAATTATTGGKCAAGAWTTAGTTGACGAATTTRTCAGTGGTATTAAACTACCAGCAGAAGTTGGAAGTCAAGATGATGTTAACAATAGAAAATTGTTGCAAAAAGTGTTTGGTAAATTAATGAACACTGATGATGACGTTATAAAACAACAAACAGCTAAATTACTTGAAAGAACAGACAGAGAACCTCAAGTGTTCAAGGATATTGATTCTAGATTACCGGAGTTAATACAAAGATTAAACAAACAATTTCCTAATGACATCGGATTATTTTGTGGATGTCTCTTATTGAACCACGTTGATTGAAAAAATTATCAATTTCCGTGTTGGATAAACAGAGATTGACTGAAAAATTCAATAAATTGGATAAATCCATTAAAGATAATTTGAAGGCTAAACAAAAAGAAGAAACCAAAAAGACTTTAGATGTGGTTAACAATTGGTTGAATGATAAAGAAAATGCTTCATCATTTTTGGTTGCTCACGTTCCAATTACTGCTAATGCCAAGGCAATCACTGAAGCCATTAATTTGATTAAAAAGCAAGATAAAACCAAATCAATTTATTTATTGACTGGTGAAACCGATAAAGTTGCTCATGGATGTTATGTTAGTGATGAAGCCATTGCCAAGGGTATTAATGCGAAAATCAAACCTTTTGAAGCACGAGCAATTAACTGGTCCACGGATCTTAATGCTGAGGTACATATTGAGCATTATATAAATATATTCAATTATGCACGATCATCTTGGGAGCCATTGGTTGAAAGTTGGCCAATAGCAGTTTACATGTCAAAATCCCGACACCCAAAGCCTCAATTATTAGTAGAGGTGATTTCTAGACAGGTAGCTCAAGTGACGCTTACATCCAAAGCAGTAGCATTGCTATCTCAAGTATCCGATTTGATTACTTCCRGAGAAAAATTAAAACCAAGAGGTGAAGATTACCCATACGTKATAGTGAATGAGACTSGTTTAGATTTGGAAGTTTGGAATGATGCAAAWGAATYCGAAACCAATGGAGTTCCAATTGTTTTACGTGCTGGTAAAGCTTTAGATGAAAGTAAAGTTGAAATTAGAATTCAATTTAAACCAGTCGCCAAGGGGATGTTTAAAGAAATTCAAAGAAATGAATTAGTTATTAGAGTACAACCAAATGAAGCCATTTATTTAAAAATTAATTCCAAAATCCCTGGAATTTCTACTGAAACTTCATTAACTGATTTAGATTTAACTTATGCTACTCGTTATTCTAAAGAYTTTTGGATTCCTGAAGCTTATGAAGCATTAATTAGAGATTGYTATTTAGGTAATCATTCTAATTTTGTTAGAGATGATGAATTGGATGTTTCTTGGAAATTATTTACTCCATTATTGAAT

>gaymc_c:IND:78:41.1

AATTATTGAAATGGAATGATATTCCATTGGCTCCACCAGACAAGATTTTGGGTATTTCTGAAGCTTATAACAACGATTCTAACCCTCAAAAAGTCAATTTGGGGGTTGGTGCTTATAGAGATAATTCTGGTAAACCAATTATTTTCCCATCAGTTAAAAAAGCTGAAGAAATTTTATTGGGTAAAGAAACTGAAAAGGAATATACTGCCATTGTTGGTTCCAAAAATTTCCAATCAATTGTGAAAAATTTCATTTTCAACAATTCTAATAAAGATGCCAATGGTAAACAATTAATTGATGATGGTAGAATTGTTACTGCTCAAACCATCTCTGGTACTGGATCACTTAAACCTCTTATGGTGAAATTTTTGCTAAACATAGAGAACCAAATTTGGAAATTATTCGTGAGGTTGTTGATTCCAAACATATTGTTTTTGATGTGTTGGCACAATTCTTAATCAATCCAGACCCATGGGTTGCCATTGCTGCCGCTGAAGTTTATGTCAGACGTTCATACCGTGCTTATGATTTGGGTACAATTGAATATCATGTTAATGACAGACTTCCTATTGTTGAATGGAAATTCAAGTTGGCTAATATGGGAGCCGCTGGTGTAAACGATGCTCAACAGGCTGCTGCTGCTGGTGGCGATGATTCGACATCTATGAAACATGCAGCTTCTGTGTCTGATTTGACCTTTGTTGTTGATTATTCATAAAAGCGAAATCCCAGGATACACTCTCCCCGATAATCCAAAGTTCACCCTTGGTAATTTGTTTGTAATAATTGGAGTCTTGTTGGTTTGTATTTTAGCTGTTGTCTCTCTTTTGAGAAATATTAGTGAGTCGGCCTTGTTCAAGAAGAATGGGTATGAACCGTTGGATTCGGATCCTAGTGTCATGAACCTAAACTTCGAGCCTACAACATTGTCCTTTGAAGATATTAAATATGAGGTTACTGGTGGTCGACAAATTTTAAATGGAGTCTTTGGGTTTGTAAAACCAAGAGAATGTTTGGCTATAATGGGAGGTTCAGGTGCTGGTAAAACTACATTGTTGGATTTTAAACCTTTGGACCAATTGGCTAARACTTTGRCYACTGTTCCTGAATTGAATGAAATTATTGGKCAAGAWTTAGTTGACGAATTTRTCAGTGGTATTAAACTACCAGCAGAAGTTGGAAGTCAAGATGATGTTAACAATAGAAAATTGTTGCAAAAAGTGTTTGGTAAATTAATGAACACTGATGATGACGTTATAAAACAACAAACAGCTAAATTACTTGAAAGAACAGACAGAGAACCTCAAGTGTTCAAGGATATTGATTCTAGATTACCGGAGTTAATACAAAGATTAAACAAACAATTTCCTAATGACATCGGATTATTTTGTGGATGTCTCTTATTGAACCACGTTGATTGAAAAAATTATCAATTTCCGTGTTGGATAAACAGAGATTGACTGAAAAATTCAATAAATTGGATAAATCCATTAAAGATAATTTGAAGGCTAAACAAAAAGAAGAAACCAAAAAGACTTTAGATGTGGTTAACAATTGGTTGAATGATAAAGAAAATGCTTCATCATTTTTGGTTGCTCACGTTCCAATTACTGCTAATGCCAAGGCAATCACTGAAGCCATTAATTTGATTAAAAAGCAAGATAAAACCAAATCAATTTATTTATTGACTGGTGAAACCGATAAAGTTGCTCATGGATGTTATGTTAGTGATGAAGCCATTGCCAAGGGTATTAATGCGAAAATCAAACCTTTTGAAGCACGAGCAATTAACTGGTCCACGGATCTTAATGCTGAGGTACATATTGAGCATTATATAAATATATTCAATTATGCACGATCATCTTGGGAGCCATTGGTTGAAAGTTGGCCAATAGCAGTTTACATGTCAAAATCCCGACACCCAAAGCCTCAATTATTAGTAGAGGTGATTTCTAGACAGGTAGCTCAAGTGACGCTTACATCCAAAGCAGTAGCATTGCTATCTCAAGTATCCGATTTGATTACTTCCRGAGAAAAATTAAAACCAAGAGGTGAAGATTACCCATACGTKATAGTGAATGAGACTSGTTTAGATTTGGAAGTTTGGAATGATGCAAAWGAATYCGAAACCAATGGAGTTCCAATTGTTTTACGTGCTGGTAAAGCTTTAGATGAAAGTAAAGTTGAAATTAGAATTCAATTTAAACCAGTCGCCAAGGGGATGTTTAAAGAAATTCAAAGAAATGAATTAGTTATTAGAGTACAACCAAATGAAGCCATTTATTTAAAAATTAATTCCAAAATCCCTGGAATTTCTACTGAAACTTCATTAACTGATTTAGATTTAACTTATGCTACTCGTTATTCTAAAGAYTTTTGGATTCCTGAAGCTTATGAAGCATTAATTAGAGATTGYTATTTAGGTAATCATTCTAATTTTGTTAGAGATGATGAATTGGATGTTTCTTGGAAATTATTTACTCCATTATTGAAT

>gaymc_c:IND:92:6.7

AATTATTGAAATGGAATGATATTCCATTGGCTCCACCAGACAAGATTTTGGGTATTTCTGAAGCTTATAACAATGATTCTAACCCTCAAAAAGTCAATTTGGGGGTTGGTGCTTATAGAGATAATTCTGGTAAACCAATTATTTTCCCATCAGTTAAAAAAGCTGAAGAAATTTTATTGGGTAAAGAAACTGAAAAGGAATATACTGCCATTGTTGGTTCCAAAAATTTCCAATCAATTGTGAAAAATTTCATTTTCAACAATTCTAATAAAGATGCCAATGGTAAACAATTAATTGATGATGGTAGAATTGTTACTGCTCAAACCATCTCTGGTACTGGATCACTTAAACCTCTTATGGTGAAATTTTTGCTAAACATAGAGAACCAAATTTGGAAATTATTCGTGAGGTTGTTGATTCCAAACATATTGTTTTTGATGTGTTGGCACAATTCTTAATCAATCCAGACCCATGGGTTGCCATTGCTGCCGCTGAAGTTTATGTCAGACGTTCATACCGTGCTTATGATTTGGGTACAATTGAATATCATGTTAATGACAGACTTCCTATTGTTGAATGGAAATTCAAGTTGGCTAATATGGGAGCCGCTGGTGTAAACGATGCTCAACAGGCTGCTGCTGCTGGTGGCGATGATTCGACATCTATGAAACATGCAGCTTCTGTGTCTGATTTGACCTTTGTTGTTGATTATTCATAAAAGCGAAATCCCAGGATACACTCTCCCCGATAATCCAAAGTTCACCCTTGGTAATTTGTTTGTAATAATTGGAGTCTTGTTGGTTTGTATTTTAGCTGTTGTCTCTCTTTTGAGAAATATTAGTGAGTCGGCCTTGTTCAAGAAGAATGGGTATGAACCGTTGGATTCGGATCCTAGTGTCATGAACCTAAACTTCGAGCCTACAACATTGTCCTTTGAAGATATTAAATATGAGGTTACTGGTGGTCGACAAATTTTAAATGGAGTCTTTGGGTTTGTAAAACCAAGAGAATGTTTGGCTATAATGGGAGGTTCAGGTGCTGGTAAAACTACATTGTTGGATTTTAAACCTTTGGACCAATTGGCTAARACTTTGRCYACTGTTCCTGAATTGAATGAAATTATTGGKCAAGAWTTAGTTGACGAATTTRTCAGTGGTATTAAACTACCAGCAGAAGTTGGAAGTCAAGATGATGTTAACAATAGAAAATTGTTGCAAAAAGTGTTTGGTAAATTAATGAACACTGATGATGACGTTATAAAACAACAAACAGCTAAATTACTTGAAAGAACAGACAGAGAACCTCAAGTGTTCAAGGATATTGATTCTAGATTACCGGAGTTAATACAAAGATTAAACAAACAATTTCCTAATGACATCGGATTATTTTGTGGATGTCTCTTATTGAACCACGTTGATTGAAAAAATTATCAATTTCCGTGTTGGATAAACAGAGATTGACTGAAAAATTCAATAAATTGGATAAATCCATCAAAGATAATTTGAAGGCTAAACAAAAAGAAGAAACCAAAAAAACTTTAGATGTGGTTAATAATTGGTTGAATGATAAAGAAAATGCTTCATCATTTTTGGTTGCTCACGTTCCAATTACTGCTAATGCCAAGGCAATCACTGAAGCCATTAATTTGATTAAAAAGCAAGATAAAACCAAATCAATTTATTTATTGACTGGTGAAACCGATAAAGTTGCTCATGGATGTTATGTTAGTGATGAAGCCATTGCCAAGGGTATTAATGCGAAAATCAAACCTTTTGAAGCACGAGCAATTAACTGGTCCACGGATCTTAATGCTGAGGTACATATTGAGCATTATATAAATATATTCAATTATGCACGATCATCTTGGGAGCCATTGGTTGAAAGTTGGCCAATAGCAGTTTACATGTCAAAATCCCGACACCCAAAGCCTCAATTATTAGTAGAGGTGATTTCTAGACAGGTAGCTCAAGTGACGCTTACATCCAAAGCAGTAGCATTGCTATCTCAAGTATCCGATTTGATTACTTCCRGAGAAAAATTAAAACCAAGAGGTGAAGATTACCCATACGTKATAGTGAATGAGACTSGTTTAGATTTGGAAGTTTGGAATGATGCAAAWGAATYCGAAACCAATGGAGTTCCAATTGTTTTACGTGCTGGTAAAGCTTTAGATGAAAGTAAAGTTGAAATTAGAATTCAATTTAAACCAGTCGCCAAGGGGATGTTTAAAGAAATTCAAAGAAATGAATTAGTTATTAGAGTACAACCAAATGAAGCCATTTATTTAAAAATTAATTCCAAAATCCCTGGAATTTCTACTGAAACTTCATTAACTGATTTAGATTTAACTTATGCTACTCGTTATTCTAAAGAYTTTTGGATTCCTGAAGCTTATGAAGCATTAATTAGAGATTGYTATTTAGGTAATCATTCTAATTTTGTTAGAGATGATGAATTGGATGTTTCTTGGAAATTATTTACTCCATTATTGAAT

>HMHc1

AATTATTGAAATGGAATGATATTCCATTGGCTCCACCAGACAAGATTTTGGGTATTTCTGAAGCTTATAACAATGATTCTAACCCTCAAAAAGTCAATTTGGGGGTTGGTGCTTATAGAGATAATTCTGGTAAACCAATTATTTTCCCATCAGTTAAAAAAGCTGAAGAAATTTTATTGGGTAAAGAAACTGAAAAGGAATATACTGCCATTGTTGGTTCCAAAAATTTCCAATCAATTGTGAAAAATTTCATTTTCAACAATTCTAATAAAGATGCCAATGGTAAACAATTAATTGATGATGGTAGAATTGTTACTGCTCAAACCATCTCTGGTACTGGATCACTTAAACCTCTTATGGTGAAATTTTTGCTAAACATAGAGAACCAAATTTGGAAATTATTCGTGAGGTTGTTGATTCCAAACATATTGTTTTTGATGTGTTGGCACAATTCTTAATCAATCCAGACCCATGGGTTGCCATTGCTGCCGCTGAAGTTTATGTCAGACGTTCATACCGTGCTTATGATTTGGGTACAATTGAATATCATGTTAATGACAGACTTCCTATTGTTGAATGGAAATTCAAGTTGGCTAATATGGGAGCCGCTGGTGTAAACGATGCTCAACAGGCTGCTGCTGCTGGTGGCGATGATTCGACATCTATGAAACATGCAGCTTCTGTGTCTGATTTGACCTTTGTTGTTGATTATTCATRAAAGCGAAATCCCAGGATACACTCTCCCCGATAATCCAAAGTTCACCCTTGGTAATTTGTTTGTAATAATTGGAGTCTTGTTGGTTTGTATTTTAGCTGTTGTCTCTCTTTTGAGAAATATTAGTGAGTCGGCCTTGTTCAAGAAGAATGGGTATGAACCGTTGGATTCGGATCCTAGTGTCATGAACCTAAACTTCGAGCCTACAACATTGTCCTTTGAAGATATTAAATATGAGGTTACTGGTGGTCGACAAATTTTAAATGGAGTCTTTGGGTTTGTAAAACCAAGAGAATGTTTGGCTATAATGGGAGGTTCAGGTGCTGGTAAAACTACATTGTTGGATTTTAAACCTTTGGACCAATTGGCTAAAACTTTGGCTACTGTTCCTGAATTGAATGAAATTATTGGTCAAGAATTAGTTGACGAATTTATCAGTGGTATTAAACTACCAGCAGAAGTTGGAAGTCAAGATGATGTTAACAATAGAAAATTGTTGCAAAAAGTGTTTGGTAAATTAATGAACACTGATGATGACGTTATAAAACAACAAACAGCTAAATTACTTGAAAGAACAGACAGAGAACCTCAAGTGTTCAAGGATATTGATTCTAGATTACCGGAGTTAATACAAAGATTAAACAAACAATTTCCTAATGACATCGGATTATTTTGTGGATGTCTCTTATTGAACCACGTTGATTGAAAAAATTATCAATTTCCGTGTTGGATAAACAGAGATTGACTGAAAAATTCAATAAATTGGATAAATCCATCAAAGATAATTTGAAGGCTAAACAAAAAGAAGAAACCAAAAAAACTTTAGATGTGGTTAATAATTGGTTGAATGATAAAGAAAATGCTTCATCATTTTTGGTTGCTCACGTTCCAATTACTGCTAATGCCAAGGCAATCACTGAAGCCATTAATTTGATTAAAAAGCAAGATAAAACCAAATCAATTTATTTATTGACTGGTGAAACCGATAAAGTTGCTCATGGATGTTATGTTAGTGATGAAGCCATTGCCAAGGGTATTAATGCGAAAATCAAACCTTTTGAAGCACGAGCAATTAACTGGTCCACGGATCTTAATGCTGAGGTACATATTGAGCATTATATAAATATATTCAATTATGCACGATCATCTTGGGAGCCATTGGTTGAAAGTTGGCCAATAGCAGTTTACATGTCAAAATCCCGACACCCAAAGCCTCAATTATTAGTAGAGGTGATTTCTAGACAGGTAGCTCAAGTGACGCTTACATCCAAAGCAGTAGCATTGCTATCTCAAGTATCCGATTTGATTACTTCCGGAGAAAAATTAAAACCAAGAGGTGAAGATTACCCATACGTTATAGTGAATGAGACTGGTTTAGATTTGGAAGTTTGGAATGATGCAAAAGAATCCGAAACCAATGGAGTTCCAATTGTTTTACGTGCTGGTAAAGCTTTAGATGAAAGTAAAGTTGAAATTAGAATTCAATTTAAACCAGTCGCCAAGGGGATGTTTAAAGAAATTCAAAGAAATGAATTAGTTATTAGAGTACAACCAAATGAAGCCATTTATTTAAAAATTAATTCCAAAATCCCTGGAATTTCTACTGAAACTTCATTAACTGATTTAGATTTAACTTATGCTACTCGTTATTCTAAAGACTTTTGGATTCCTGAAGCTTATGAAGCATTAATTAGAGATTGCTATTTAGGTAATCATTCTAATTTTGTTAGAGATGATGAATTGGATGTTTCTTGGAAATTATTTACTCCATTATTGAAT

>HMHc2

AATTATTGAARTGGAATGATATTCCATTGGCTCCACCAGACAARATTTTGGGTATTTCTGAAGCTTATAACAATGATTCTAACCCTCAAAAAGTCAATTTGGGGGTTGGTGCTTATAGAGATAATTCTGGTAAACCAATTATTTTCCCATCAGTTAAAAAAGCTGAAGAAATTTTATTGGGTAAAGAAACTGAAAAGGAATATACTGCCATTGTTGGTTCCAAAAATTTCCAATCAATTGTGAAAAATTTCATTTTCAACAATTCTAATAAAGATGCCAATGGTAAACAATTAATTGATGATGGTAGAATTGTTACTGCTCAAACCATCTCTGGTACTGGATCACTTAAACCTCTTATGGTGAAATTTTTGCTAAACATAGAGAACCAAATTTGGAAATTATTCGTGAGGTTGTTGATTCCAAACATATTGTTTTTGATGTGTTGGCACAATTCTTAATCAATCCAGACCCATGGGTTGCCATTGCTGCCGCTGAAGTTTATGTCAGACGTTCATACCGTGCTTATGATTTGGGTAAAATTGAATATCATGTTAATGACAGACTTCCTATTGTTGAATGGAAATTCAAGTTGGCTAATATGGGAGCCGCTGGTGTAAACGATGCTCAACAGGCTGCTGCTGCYGGTGGCGATGATTCGACATCTATGAAACATGCAGCTTCTGTGTCTGATTTGACCTTTGTTGTTGATTATTCATGAAAGCGAAATCCCAGGATACACTCTCCCCGATAATCCAAAGTTCACCCTTGGTAATTTGTTTGTAATAATTGGAGTCTTGTTGGTTTGTATTTTAGCTGTTGTCTCTCTTTTGAGAAATATTAGTGAGTCGGCCTTGTTCAAGAAGAATGGGTATGAACCGTTGGATTCGGATCCTAGTGTCATGAACCTAAACTTCGAGCCTACAACATTGTCCTTTGAAGATATTAAATATGAGGTTACTGGTGGTCGACAAATTTTAAATGGAGTCTTTGGGTTTGTAAAACCAAGAGAATGTTTGGCTATAATGGGAGGTTCAGGTGCTGGTAAAACTACATTGTTGGATTTTAAACCTTTGGACCAATTGGCTAARACTTTGRCYACTGTTCCTGAATTGAATGAAATTATTGGKCAAGAWTTAGTTGACGAATTTRTCAGTGGTATTAAACTACCAGCAGAAGTTGGAAGTCAAGATGATGTTAACAATAGAAAATTGTTGCAAAAAGTGTTTGGTAAATTAATGAACACTGATGATGACGTTATAAAACAACAAACAGCTAAATTACTTGAAAGAACAGACAGAGAACCTCAAGTGTTCAAGGATATTGATTCTAGATTACCGGAGTTAATACAAAGATTAAACAAACAATTTCCTAATGACATCGGATTATTTTGTGGATGTCTCTTATTGAACCACGTTGATTGAAAAAATTATCAATTTCCGTGTTGGATAAACAGAGATTGACTGAAAAATTCAATAAATTGGATAAATCCATCAAAGATAATTTGAAGGCTAAACAAAAAGAAGAAACCAAAAAAACTTTAGATGTGGTTAATAATTGGTTGAATGATAAAGAAAATGCTTCATCATTTTTGGTTGCTCACGTTCCAATTACTGCTAATGCCAAGGCAATCACTGAAGCCATTAATTTGATTAAAAAGCAAGATAAAACCAAATCAATTTATTTATTGACTGGTGAAACCGATAAAGTTGCTCATGGATGTTATGTTAGTGATGAAGCCATTGCCAAGGGTATTAATGCGAAAATCAAACCTTTTGAAGCACGAGCAATTAACTGGTCCACGGATCTTAATGCTGAGGTACATATTGAGCATTATATAAATATATTCAATTATGCACGATCATCTTGGGAGCCATTGGTTGAAAGTTGGCCAATAGCAGTTTACATGTCAAAATCCCGACACCCAAAGCCTCAATTATTAGTAGAGGTGATTTCTAGACAGGTAGCYCAAGTGACGCTTACATCCAAAGCAGTAGCATTGCTATCTCAAGTATCCGATTTGATTACTTCCRGAGAAAAATTAAAACCAAGAGGTGAAGATTACCCATACGTKATAGTGAATGAGACTGGTTTAGATTTGGAAGTTTGGAATGATGCAAAWGAATYCGAAACCAATGGAGTTCCAATTGTTTTACGTGCTGGTAAAGCTTTAGATGAAAGTAAAGTTGAAATTAGAATTCAATTTAAACCAGTCGCCAAGGGGATGTTTAAATAAATTCAAAGAAATGAATTAGTTATTAGAGTACAACCAAATGAAGCCATTTATTTAAAAATTAATTCCAAAATCCCTGGAATTTCTACTGAAACTTCATTAACTGATTTAGATTTAACTTATGCTACTCGTTATTCTAAAGAYTTTTGGATTCCTGAAGCTTATGAAGCATTAATTAGAGATTGYTATTTAGGTAATCATTCTAATTTTGTTAGAGATGATGAATTGGATGTTTCTTGGAAATTATTTACTCCATTATTGAAT

>HMHc4

AATTATTGAARTGGAATGATATTCCATTGGCTCCACCAGACAARATTTTGGGTATTTCTGAAGCTTATAACAATGATTCTAACCCTCAAAAAGTCAATTTGGGGGTTGGTGCTTATAGAGATAATTCTGGTAAACCAATTATTTTCCCATCAGTTAAAAAAGCTGAAGAAATTTTATTGGGTAAAGAAACTGAAAAGGAATATACTGCCATTGTTGGTTCCAAAAATTTCCAATCAATTGTGAAAAATTTCATTTTCAACAATTCTAATAAAGATGCCAATGGTAAACAATTAATTGATGATGGTAGAATTGTTACTGCTCAAACCATCTCTGGTACTGGATCACTTAAACCTCTTATGGTGAAATTTTTGCTAAACATAGAGAACCAAATTTGGAAATTATTCGTGAGGTTGTTGATTCCAAACATATTGTTTTTGATGTGTTGGCACAATTCTTAATCAATCCAGACCCATGGGTTGCCATTGCTGCCGCTGAAGTTTATGTCAGACGTTCATACCGTGCTTATGATTTGGGTAAAATTGAATATCATGTTAATGACAGACTTCCTATTGTTGAATGGAAATTCAAGTTGGCTAATATGGGAGCCGCTGGTGTAAACGATGCTCAACAGGCTGCTGCTGCYGGTGGCGATGATTCGACATCTATGAAACATGCAGCTTCTGTGTCTGATTTGACCTTTGTTGTTGATTATTCATRAAAGCGAAATCCCAGGATACACTCTCCCCGATAATCCAAAGTTCACCCTTGGTAATTTGTTTGTAATAATTGGAGTCTTGTTGGTTTGTATTTTAGCTGTTGTCTCTCTTTTGAGAAATATTAGTGAGTCGGCCTTGTTCAAGAAGAATGGGTATGAACCGTTGGATTCGGATCCTAGTGTCATGAACCTAAACTTCGAGCCTACAACATTGTCCTTTGAAGATATTAAATATGAGGTTACTGGTGGTCGACAAATTTTAAATGGAGTCTTTGGGTTTGTAAAACCAAGAGAATGTTTGGCTATAATGGGAGGTTCAGGTGCTGGTAAAACTACATTGTTGGATTTTAAACCTTTGGACCAATTGGCTAARACTTTGRCYACTGTTCCTGAATTGAATGAAATTATTGGKCAAGAWTTAGTTGACGAATTTRTCAGTGGTATTAAACTACCAGCAGAAGTTGGAAGTCAAGATGATGTTAACAATAGAAAATTGTTGCAAAAAGTGTTTGGTAAATTAATGAACACTGATGATGACGTTATAAAACAACAAACAGCTAAATTACTTGAAAGAACAGACAGAGAACCTCAAGTGTTCAAGGATATTGATTCTAGATTACCGGAGTTAATACAAAGATTAAACAAACAATTTCCTAATGACATCGGATTATTTTGTGGATGTCTCTTATTGAACCACGTTGATTGAAAAAATTATCAATTTCCGTGTTGGATAAACAGAGATTGACTGAAAAATTCAATAAATTGGATAAATCCATCAAAGATAATTTGAAGGCTAAACAAAAAGAAGAAACCAAAAAAACTTTAGATGTGGTTAATAATTGGTTGAATGATAAAGAAAATGCTTCATCATTTTTGGTTGCTCACGTTCCAATTACTGCTAATGCCAAGGCAATCACTGAAGCCATTAATTTGATTAAAAAGCAAGATAAAACCAAATCAATTTATTTATTGACTGGTGAAACCGATAAAGTTGCTCATGGATGTTATGTTAGTGATGAAGCCATTGCCAAGGGTATTAATGCGAAAATCAAACCTTTTGAAGCACGAGCAATTAACTGGTCCACGGATCTTAATGCTGAGGTACATATTGAGCATTATATAAATATATTCAATTATGCACGATCATCTTGGGAGCCATTGGTTGAAAGTTGGCCAATAGCAGTTTACATGTCAAAATCCCGACACCCAAAGCCTCAATTATTAGTAGAGGTGATTTCTAGACAGGTAGCYCAAGTGACGCTTACATCCAAAGCAGTAGCATTGCTATCTCAAGTATCCGATTTGATTACTTCCRGAGAAAAATTAAAACCAAGAGGTGAAGATTACCCATACGTKATAGTGAATGAGACTGGTTTAGATTTGGAAGTTTGGAATGATGCAAAWGAATYCGAAACCAATGGAGTTCCAATTGTTTTACGTGCTGGTAAAGCTTTAGATGAAAGTAAAGTTGAAATTAGAATTCAATTTAAACCAGTCGCCAAGGGGATGTTTAAAGAAATTCAAAGAAATGAATTAGTTATTAGAGTACAACCAAATGAAGCCATTTATTTAAAAATTAATTCCAAAATCCCTGGAATTTCTACTGAAACTTCATTAACTGATTTAGATTTAACTTATGCTACTCGTTATTCTAAAGAYTTTTGGATTCCTGAAGCTTATGAAGCATTAATTAGAGATTGYTATTTAGGTAATCATTCTAATTTTGTTAGAGATGATGAATTGGATGTTTCTTGGAAATTATTTACTCCATTATTGAAT

>HMHc5

AATTATTGAAATGGAATGATATTCCATTGGCTCCACCAGACAAAATTTTGGGTATTTCTGAAGCTTATAACAACGATTCTAACCCTCAAAAAATCAATTTGGGGGTTGGTGCTTATAGAGATAATTCCGGTAAACCAATTATTTTCCCATCAGTTAAAAAAGCTGAAGAAATTTTATTGGGTAAAGAAACTGAAAAGGAATATACTGCCATTGTTGGTTCCAAAAATTTCCAATCAATTGTGAAAAATTTCATTTTCAACAATTCTAATAAAGATGCCAATGGTAAACAATTAATTGATGATGGTAGAATTGTTACTGCTCAAACCATCTCTGGTACTGGATCACTTAAACCTCTTATGGTGAAATTTTTGCTAAACATAGAGAACCAAATTTGGAAATTATTCGTGAGGTTGTTGATTCCAAACATATTGTTTTTGATGTGTTGGCACAATTCTTAATCAATCCAGACCCATGGGTTGCCATTGCTGCCGCTGAAGTTTATGTCAGACGTTCATACCGTGCTTATGATTTGGGTAAAATTGAATATCATGTTAATGACAGACTTCCTATTGTTGAATGGAAATTCAAGTTGGCTAATATGGGAGCYGCTGGTGTAAACGATGCTCAACAGGCTGCTGCTGCYGGTGGCGATGATTCGACATCTATGAAACATGCAGCTTCTGTGTCTGATTTGACCTTTGTTGTTGATTATTCATAAAAGCGAAATCCCAGGATACACTCTCCCCGATAATCCAAAGTTCACCCTTGGTAATTTGTTTGTAATAATTGGAGTCTTGTTGGTTTGTRTTTTAGCTGTTGTCTCTCTTTTGAGAAATATTAGTGAGTCGGCCTTGTTCAAGAAGAATGGGTATGAACCGTTGGATTCGGATCCTAGTGTCATGAACCTAAACTTCGAGCCTACAACATTGTCCTTTGAAGATATTAAATATGAGGTTACTGGTGGTCGACAAATTTTAAATGGAGTCTTTGGGTTTGTAAAACCAAGAGAATGTTTGGCTATAATGGGAGGTTCAGGTGCTGGTAAAACTACATTGTTGGATTTTAAACCTTTGGACCAATTGGCTAAGACTTTGACCACTGTTCCTGAATTGAATGAAATTATTGGGCAAGATTTAGTTGACGAATTTGTCAGTGGTATTAAACTACCAGCAGAAGTTGGAAGTCAAGATGATGTTAACAATAGAAAATTGTTGCAAAAAGTGTTTGGTAAATTAATGAACACTGATGATGACGTTATAAAACAACAAACAGCTAAATTACTTGAAAGAACAGACAGAGAACCTCAAGTGTTCAAGGATATTGATTCTAGATTACCGGAGTTAATACAAAGATTAAACAAACAATTTCCTAATGACATCGGATTATTTTGTGGATGTCTCTTATTGAACCACGTTGMTTGAAAAAATTATCAATTTCCGTGTTGGATAAACAAAGATTGACTGAAAAATTCAATAAATTGGATAAATCCATYAAAGATAATTTGAAGGCTAAACAAAAAGAAGAAACCAAAAAGACTTTAGATGTGGTTAACAATTGGTTGAATGATAAAGAAAATGCTTCATCATTTTTGGTTGCTCACGTTCCAATTACTGCTAATGCCAAGGCAATCACTGAAGCCATTAATTTGATTAAAAAGCAAGATAAAACCAAATCAATTTATTTATTGACTGGTGAAACCGATAAAGTTGCTCATGGATGTTATGTTAGTGATGAAGCCATTGTCAAGGGTATTAATGCGAAAATCAAACCTTTTGAAGCACGAGCAATTAACTGGTCCACGGATCTTAATGCTGAGGTACATATTGAGCATTATATAAATATATTCAATTATGCACGATCATCTTGGGAGCCATTGGTTGAAAGTTGGCCAATAGCAGTTTACATGTCAAAATCCCGACACCCAAAGCCTCAATTATTAGTAGAGGTGATTTCTAGACAGGTAGCTCAAGTGACGCTTACATCCAAAGCRGTAGCATTGCTATCTCAAGTATCCGATTTGATTACTTCCRGAGAAAAATTAAAACCAAGAGGTGAAGATTACCCATACGTKATAGTGAATGAGACTGGTTTAGATTTGGAAGTTTGGAATGATGCAAATGAATYCGAAACCAATGGAGTTCCAATTGTTTTACGTGCTGGTAAAGCTTTAGATGAAAGTAAAGTTGAAATTAGAATTCAATTTAAACCAGTCGCCAAGGGGATGTTTAAAGAAATTCAAAGAAATGAATTAGTTATTAGAGTACAACCAAATGAAGCCATTTATTTAAAAATTAATTCCAAAATCCCTGGAATTTCTACTGAAACTTCATTAACTGATTTAGATTTAACTTATGCTACTCGTTATTCTAAAGACTTTTGGATTCCTGAAGCTTATGAAGCATTAATTAGAGATTGTTATTTAGGTAATCATTCTAATTTTGTTAGAGATGATGAATTGGATGTTTCTTGGAAATTATTTACTCCATTATTGAAT

>HMHc6

AATTATTGAAATGGAATGATATTCCATTGGCTCCACCAGACAAGATTTTGGGTATTTCTGAAGCTTATAACAATGATTCTAACCCTCAAAAAGTCAATTTGGGGGTTGGTGCTTATAGAGATAATTCTGGTAAACCAATTATTTTCCCATCAGTTAAAAAAGCTGAAGAAATTTTATTGGGTAAAGAAACTGAAAAGGAATATACTGCCATTGTTGGTTCCAAAAATTTCCAATCAATTGTGAAAAATTTCATTTTCAACAATTCTAATAAAGATGCCAATGGTAAACAATTAATTGATGATGGTAGAATTGTTACTGCTCAAACCATCTCTGGTACTGGATCACTTAAACCTCTTATGGTGAAATTTTTGCTAAACATAGAGAACCAAATTTGGAAATTATTCGTGAGGTTGTTGATTCCAAACATATTGTTTTTGATGTGTTGGCACAATTCTTAATCAATCCAGACCCATGGGTTGCCATTGCTGCCGCTGAAGTTTATGTCAGACGTTCATACCGTGCTTATGATTTGGGTACAATTGAATATCATGTTAATGACAGACTTCCTATTGTTGAATGGAAATTCAAGTTGGCTAATATGGGAGCCGCTGGTGTAAACGATGCTCAACAGGCTGCTGCTGCTGGTGGCGATGATTCGACATCTATGAAACATGCAGCTTCTGTGTCTGATTTGACCTTTGTTGTTGATTATTCATRAAAGCGAAATCCCAGGATACACTCTCCCCGATAATCCAAAGTTCACCCTTGGTAATTTGTTTGTAATAATTGGAGTCTTGTTGGTTTGTATTTTAGCTGTTGTCTCTCTTTTGAGAAATATTAGTGAGTCGGCCTTGTTCAAGAAGAATGGGTATGAACCGTTGGATTCGGATCCTAGTGTCATGAACCTAAACTTCGAGCCTACAACATTGTCCTTTGAAGATATTAAATATGAGGTTACTGGTGGTCGACAAATTTTAAATGGAGTCTTTGGGTTTGTAAAACCAAGAGAATGTTTGGCTATAATGGGAGGTTCAGGTGCTGGTAAAACTACATTGTTGGATTTTAAACCTTTGGACCAATTGGCTAAAACTTTGGCTACTGTTCCTGAATTGAATGAAATTATTGGTCAAGAATTAGTTGACGAATTTATCAGTGGTATTAAACTACCAGCAGAAGTTGGAAGTCAAGATGATGTTAACAATAGAAAATTGTTGCAAAAAGTGTTTGGTAAATTAATGAACACTGATGATGACGTTATAAAACAACAAACAGCTAAATTACTTGAAAGAACAGACAGAGAACCTCAAGTGTTCAAGGATATTGATTCTAGATTACCGGAGTTAATACAAAGATTAAACAAACAATTTCCTAATGACATCGGATTATTTTGTGGATGTCTCTTATTGAACCACGTTGATTGAAAAAATTATCAATTTCCGTGTTGGATAAACAGAGATTGACTGAAAAATTCAATAAATTGGATAAATCCATCAAAGATAATTTGAAGGCTAAACAAAAAGAAGAAACCAAAAAAACTTTAGATGTGGTTAATAATTGGTTGAATGATAAAGAAAATGCTTCATCATTTTTGGTTGCTCACGTTCCAATTACTGCTAATGCCAAGGCAATCACTGAAGCCATTAATTTGATTAAAAAGCAAGATAAAACCAAATCAATTTATTTATTGACTGGTGAAACCGATAAAGTTGCTCATGGATGTTATGTTAGTGATGAAGCCATTGCCAAGGGTATTAATGCGAAAATCAAACCTTTTGAAGCACGAGCAATTAACTGGTCCACGGATCTTAATGCTGAGGTACATATTGAGCATTATATAAATATATTCAATTATGCACGATCATCTTGGGAGCCATTGGTTGAAAGTTGGCCAATAGCAGTTTACATGTCAAAATCCCGACACCCAAAGCCTCAATTATTAGTAGAGGTGATTTCTAGACAGGTAGCYCAAGTGACGCTTACATCCAAAGCAGTAGCATTGCTATCTCAAKTATCCGATTTGATTACTTCCRGAGAAAAATTAAAACCAAGAGGTGAAGATTACCCATACGTKATAGTGAATGAGACTGGTTTAGATTTGGAAGTTTGGAATGATGCAAAWGAATYCGAAACCAATGGAGTTCCAATTGTTTTACGTGCTGGTAAAGCTTTAGATGAAAGTAAAGTTGAAATTAGAATTCAATTTAAACCAGTCGCYAAGGGGATGTTTAAAGAAATTCAAAGAAATGAATTAGTTATTAGAGTACAACCAAATGAAGCCATTTATTTAAAAATTAATTCCAAAATCCCTGGAATTTCTACTGAAACTTCATTAACTGATTTAGATTTAACTTATGCTACTCGTTATTCTAAAGAYTTTTGGATTCCTGAAGCTTATGAAGCATTAATTAGAGATTGYTATTTAGGTAATCATTCTAATTTTGTTAGAGATGATGAATTGGATGTTTCTTGGAAATTATTTACTCCATTATTGAAT

>HMHc9

AATTATTGAARTGGAATGATATTCCATTGGCTCCACCAGACAARATTTTGGGTATTTCTGAAGCTTATAACAATGATTCTAACCCTCAAAAARTCAATTTGGGGGTTGGTGCTTATAGAGATAATTCYGGTAAACCAATTATTTTCCCATCAGTTAAAAAAGCTGAAGAAATTTTATTGGGTAAAGAAACTGAAAAGGAATATACTGCCATTGTTGGTTCCAAAAATTTCCAATCAATTGTGAAAAATTTCATTTTCAACAATTCTAATAAAGATGCCAATGGTAAACAATTAATTGATGATGGTAGAATTGTTACTGCTCAAACCATCTCTGGTACTGGATCACTTAAACCTCTTATGGTGAAATTTTTGCTAAACATAGAGAACCAAATTTGGAAATTATTCGTGAGGTTGTTGATTCCAAACATATTGTTTTTGATGTGTTGGCACAATTCTTAATCAATCCAGACCCATGGGTTGCCATTGCTGCCGCTGAAGTTTATGTCAGACGTTCATACCGTGCTTATGATTTGGGTAMAATTGAATATCATGTTAATGACAGACTTCCTATTGTTGAATGGAAATTCAAGTTGGCTAATATGGGAGCCGCTGGTGTAAACGATGCTCAACAGGCTGCTGCTGCYGGTGGCGATGATTCGACATCTATGAAACATGCAGCTTCTGTGTCTGATTTGACCTTTGTTGTTGATTATTCATRAAAGCGAAATCCCAGGATACACTCTCCCCGATAATCCAAAGTTCACCCTTGGTAATTTGTTTGTAATAATTGGAGTCTTGTTGGTTTGTATTTTAGCTGTTGTCTCTCTTTTGAGAAATATTAGTGAGTCGGCCTTGTTCAAGAAGAATGGGTATGAACCGTTGGATTCGGATCCTAGTGTCATGAACCTAAACTTCGAGCCTACAACATTGTCCTTTGAAGATATTAAATATGAGGTTACTGGTGGTCGACAAATTTTAAATGGAGTCTTTGGGTTTGTAAAACCAAGAGAATGTTTGGCTATAATGGGAGGTTCAGGTGCTGGTAAAACTACATTGTTGGATTTTAAACCTTTGGACCAATTGGCTAARACTTTGRCYACTGTTCCTGAATTGAATGAAATTATTGGKCAAGAWTTAGTTGACGAATTTRTCAGTGGTATTAAACTACCAGCAGAAGTTGGAAGTCAAGATGATGTTAACAATAGAAAATTGTTGCAAAAAGTGTTTGGTAAATTAATGAACACTGATGATGACGTTATAAAACAACAAACAGCTAAATTACTTGAAAGAACAGACAGAGAACCTCAAGTGTTCAAGGATATTGATTCTAGATTACCGGAGTTAATACAAAGATTAAACAAACAATTTCCTAATGACATCGGATTATTTTGTGGATGTCTCTTATTGAACCACGTTGATTGAAAAAATTATCAATTTCCGTGTTGGATAAACAGAGATTGACTGAAAAATTCAATAAATTGGATAAATCCATCAAAGATAATTTGAAGGCTAAACAAAAAGAAGAAACCAAAAAAACTTTAGATGTGGTTAATAATTGGTTGAATGATAAAGAAAATGCTTCATCATTTTTGGTTGCTCACGTTCCAATTACTGCTAATGCCAAGGCAATCACTGAAGCCATTAATTTGATTAAAAAGCAAGATAAAACCAAATCAATTTATTTATTGACTGGTGAAACCGATAAAGTTGCTCATGGATGTTATGTTAGTGATGAAGCCATTGCCAAGGGTATTAATGCGAAAATCAAACCTTTTGAAGCACGAGCAATTAACTGGTCCACGGATCTTAATGCTGAGGTACATATTGAGCATTATATAAATATATTCAATTATGCACGATCATCTTGGGAGCCATTGGTTGAAAGTTGGCCAATAGCAGTTTACATGTCAAAATCCCGACACCCAAAGCCTCAATTATTAGTAGAGGTGATTTCTAGACAGGTAGCCCAAGTGACGCTTACATCCAAAGCAGTAGCATTGCTATCTCAAGTATCCGATTTGATTACTTCCGGAGAAAAATTAAAACCAAGAGGTGAAGATTACCCATACGTTATAGTGAATGAGACTGGTTTAGATTTGGAAGTTTGGAATGATGCAAAAGAATCCGAAACCAATGGAGTTCCAATTGTTTTACGTGCTGGTAAAGCTTTAGATGAAAGTAAAGTTGAAATTAGAATTCAATTTAAACCAGTCGCCAAGGGGATGTTTAAAGAAATTCAAAGAAATGAATTAGTTATTAGAGTACAACCAAATGAAGCCATTTATTTAAAAATTAATTCCAAAATCCCTGGAATTTCTACTGAAACTTCATTAACTGATTTAGATTTAACTTATGCTACTCGTTATTCTAAAGAYTTTTGGATTCCTGAAGCTTATGAAGCATTAATTAGAGATTGYTATTTAGGTAATCATTCTAATTTTGTTAGAGATGATGAATTGGATGTTTCTTGGAAATTATTTACTCCATTATTGAAT

>hp6ch

AATTATTGAAATGGAATGATATTCCATTGGCTCCACCAGACAAAATTTTGGGTATTTCTGAAGCTTATAACAACGATTCTAACCCTCAAAAAATCAATTTGGGGGTTGGTGCTTATAGAGATAATTCCGGTAAACCAATTATTTTCCCATCAGTTAAAAAAGCTGAAGAAATTTTATTGGGTAAAGAAACTGAAAAGGAATATACTGCCATTGTTGGTTCCAAAAATTTCCAATCAATTGTGAAAAATTTCATTTTCAACAATTCTAATAAAGATGCCAATGGTAAACAATTAATTGATGATGGTAGAATTGTTACTGCTCAAACCATCTCTGGTACTGGATCACTTAAACCTCTTATGGTGAAATTTTTGCTAAACATAGAGAACCAAATTTGGAAATTATTCGTGAGGTTGTTGATTCCAAACATATTGTTTTTGATGTGTTGGCACAATTCTTAATCAATCCAGACCCATGGGTTGCCATTGCTGCCGCTGAAGTTTATGTCAGACGTTCATACCGTGCTTATGATTTGGGTAAAATTGAATATCATGTTAATGACAGACTTCCTATTGTTGAATGGAAATTCAAGTTGGCTAATATGGGAGCTGCTGGTGTAAACGATGCTCAACAGGCTGCTGCTGCCGGTGGCGATGATTCGACATCTATGAAACATGCAGCTTCTGTGTCTGATTTGACCTTTGTTGTTGATTATTCATAAAAGCGAAATCCCAGGATACACTCTCCCCGATAATCCAAAGTTCACCCTTGGTAATTTGTTTGTAATAATTGGAGTCTTGTTGGTTTGTATTTTAGCTGTTGTCTCTCTTTTGAGAAATATTAGTGAGTCGGCCTTGTTCAAGAAGAATGGGTATGAACCGTTGGATTCGGATCCTAGTGTCATGAACCTAAACTTCGAGCCTACAACATTGTCCTTTGAAGATATTAAATATGAGGTTACTGGTGGTCGACAAATTTTAAATGGAGTCTTTGGGTTTGTAAAACCAAGAGAATGTTTGGCTATAATGGGAGGTTCAGGTGCTGGTAAAACTACATTGTTGGATTTTAAACCTTTGGACCAATTGGCTAAGACTTTGACCACTGTTCCTGAATTGAATGAAATTATTGGGCAAGATTTAGTTGACGAATTTGTCAGTGGTATTAAACTACCAGCAGAAGTTGGAAGTCAAGATGATGTTAACAATAGAAAATTGTTGCAAAAAGTGTTTGGTAAATTAATGAACACTGATGATGACGTTATAAAACAACAAACAGCTAAATTACTTGAAAGAACAGACAGAGAACCTCAAGTGTTCAAGGATATTGATTCTAGATTACCGGAGTTAATACAAAGATTAAACAAACAATTTCCTAATGACATCGGATTATTTTGTGGATGTCTCTTATTGAACCACGTTGMTTGAAAAAATTATCAATTTCCGTGTTGGATAAACAAAGATTGACTGAAAAATTCAATAAATTGGATAAATCCATYAAAGATAATTTGAAGGCTAAACAAAAAGAAGAAACCAAAAARACTTTAGATGTGGTTAAYAATTGGTTGAATGATAAAGAAAATKCTTCATCATTTTTGGTTGCTCACGTTCCAATTACTGCTAATGCCAAGGCAATCACTGAAGCCATTAATTTGATTAAAAAGCAAGATAAAACCAAATCAATTTATTTATTGACTGGTGAAACCGATAAAGTTGCTCATGGATGTTATGTTAGTGATGAAGCCATTGTCAAGGGTATTAATGCGAAAATCAAACCTTTTGAAGCACGAGCAATTAACTGGTCCACGGATCTTAATGCTGAGGTACATATTGAGCATTATATAAATATATTCAATTATGCACGATCATCTTGGGAGCCATTGGTTGAAAGTTGGCCAATAGCAGTTTACATGTCAAAATCCCGACACCCAAAGCCTCAATTATTAGTAGAGGTGATTTCTAGACAGGTAGCTCAAGTGACGCTTACATCCAAAGCGGTAGCATTGCTATCTCAAGTATCCGATTTGATTACTTCCGGAGAAAAATTAAAACCAAGAGGTGAAGATTACCCATACGTTATAGTGAATGAGACTGGTTTAGATTTGGAAGTTTGGAATGATGCAAATGAATCCGAAACCAATGGAGTTCCAATTGTTTTACGTGCTGGTAAAGCTTTAGATGAAAGTAAAGTTGAAATTAGAATTCAATTTAAACCAGTCGCCAAGGGGATGTTTAAAGAAATTCAAAGAAATGAATTAGTTATTAGAGTACAACCAAATGAAGCCATTTATTTAAAAATTAATTCCAAAATCCCTGGAATTTCTACTGAAACTTCATTAACTGATTTAGATTTAACTTATGCTACTCGTTATTCTAAAGACTTTTGGATTCCTGAAGCTTATGAAGCATTAATTAGAGATTGYTATTTAGGTAATCATTCTAATTTTGTTAGAGATGATGAATTGGATGTTTCTTGGAAATTATTTACTCCATTATTGAAT

>hp11an

AATTATTGAARTGGAATGATATTCCATTGGCTCCACCAGACAARATTTTGGGTATTTCTGAAGCTTATAACAATGATTCTAACCCTCAAAAAGTCAATTTGGGGGTTGGTGCTTATAGAGATAATTCTGGTAAACCAATTATTTTCCCATCAGTTAAAAAAGCTGAAGAAATTTTATTGGGTAAAGAAACTGAAAAGGAATATACTGCCATTGTTGGTTCCAAAAATTTCCAATCAATTGTGAAAAATTTCATTTTCAACAATTCTAATAAAGATGCCAATGGTAAACAATTAATTGATGATGGTAGAATTGTTACTGCTCAAACCATCTCTGGTACTGGATCACTTAAACCTCTTATGGTGAAATTTTTGCTAAACATAGAGAACCAAATTTGGAAATTATTCGTGAGGTTGTTGATTCCAAACATATTGTTTTTGATGTGTTGGCACAATTCTTAATCAATCCAGACCCATGGGTTGCCATTGCTGCCGCTGAAGTTTATGTCAGACGTTCATACCGTGCTTATGATTTGGGTAMAATTGAATATCATGTTAATGACAGACTTCCTATTGTTGAATGGAAATTCAAGTTGGCTAATATGGGAGCCGCTGGTGTAAACGATGCTCAACAGGCTGCTGCTGCYGGTGGCGATGATTCGACATCTATGAAACATGCAGCTTCTGTGTCTGATTTGACCTTTGTTGTTGATTATTCATRAAAGCGAAATCCCAGGATACACTCTCCCCGATAATCCAAAGTTCACCCTTGGTAATTTGTTTGTAATAATTGGAGTCTTGTTGGTTTGTATTTTAGCTGTTGTCTCTCTTTTGAGAAATATTAGTGAGTCGGCCTTGTTCAAGAAGAATGGGTATGAACCGTTGGATTCGGATCCTAGTGTCATGAACCTAAACTTCGAGCCTACAACATTGTCCTTTGAAGATATTAAATATGAGGTTACTGGTGGTCGACAAATTTTAAATGGAGTCTTTGGGTTTGTAAAACCAAGAGAATGTTTGGCTATAATGGGAGGTTCAGGTGCTGGTAAAACTACATTGTTGGATTTTAAACCTTTGGACCAATTGGCTAARACTTTGRCYACTGTTCCTGAATTGAATGAAATTATTGGKCAAGAWTTAGTTGACGAATTTRTCAGTGGTATTAAACTACCAGCAGAAGTTGGAAGTCAAGATGATGTTAACAATAGAAAATTGTTGCAAAAAGTGTTTGGTAAATTAATGAACACTGATGATGACGTTATAAAACAACAAACAGCTAAATTACTTGAAAGAACAGACAGAGAACCTCAAGTGTTCAAGGATATTGATTCTAGATTACCGGAGTTAATACAAAGATTAAACAAACAATTTCCTAATGACATCGGATTATTTTGTGGATGTCTCTTATTGAACCACGTTGATTGAAAAAATTATCAATTTCCGTGTTGGATAAACAGAGATTGACTGAAAAATTCAATAAATTGGATAAATCCATCAAAGATAATTTGAAGGCTAAACAAAAAGAAGAAACCAAAAAAACTTTAGATGTGGTTAATAATTGGTTGAATGATAAAGAAAATGCTTCATCATTTTTGGTTGCTCACGTTCCAATTACTGCTAATGCCAAGGCAATCACTGAAGCCATTAATTTGATTAAAAAGCAAGATAAAACCAAATCAATTTATTTATTGACTGGTGAAACCGATAAAGTTGCTCATGGATGTTATGTTAGTGATGAAGCCATTGCCAAGGGTATTAATGCGAAAATCAAACCTTTTGAAGCACGAGCAATTAACTGGTCCACGGATCTTAATGCTGAGGTACATATTGAGCATTATATAAATATATTCAATTATGCACGATCATCTTGGGAGCCATTGGTTGAAAGTTGGCCAATAGCAGTTTACATGTCAAAATCCCGACACCCAAAGCCTCAATTATTAGTAGAGGTGATTTCTAGACAGGTAGCTCAAGTGACGCTTACATCCAAAGCAGTAGCATTGCTATCTCAAGTATCCGATTTGATTACTTCCRGAGAAAAATTAAAACCAAGAGGTGAAGATTACCCATACGTKATAGTGAATGAGACTGGTTTAGATTTGGAAGTTTGGAATGATGCAAAWGAATYCGAAACCAATGGAGTTCCAATTGTTTTACGTGCTGGTAAAGCTTTAGATGAAAGTAAAGTTGAAATTAGAATTCAATTTAAACCAGTCGCCAAGGGGATGTTTAAAGAAATTCAAAGAAATGAATTAGTTATTAGAGTACAACCAAATGAAGCCATTTATTTAAAAATTAATTCCAAAATCCCTGGAATTTCTACTGAAACTTCATTAACTGATTTAGATTTAACTTATGCTACTCGTTATTCTAAAGACTTTTGGATTCCTGAAGCTTATGAAGCATTAATTAGAGATTGCTATTTAGGTAATCATTCTAATTTTGTTAGAGATGATGAATTGGATGTTTCTTGGAAATTATTTACTCCATTATTGAAT

>hp11vw

AATTATTGAARTGGAATGATATTCCATTGGCTCCACCAGACAARATTTTGGGTATTTCTGAAGCTTATAACAATGATTCTAACCCTCAAAAARTCAATTTGGGGGTTGGTGCTTATAGAGATAATTCYGGTAAACCAATTATTTTCCCATCAGTTAAAAAAGCTGAAGAAATTTTATTGGGTAAAGAAACTGAAAAGGAATATACTGCCATTGTTGGTTCCAAAAATTTCCAATCAATTGTGAAAAATTTCATTTTCAACAATTCTAATAAAGATGCCAATGGTAAACAATTAATTGATGATGGTAGAATTGTTACTGCTCAAACCATCTCTGGTACTGGATCACTTAAACCTCTTATGGTGAAATTTTTGCTAAACATAGAGAACCAAATTTGGAAATTATTCGTGAGGTTGTTGATTCCAAACATATTGTTTTTGATGTGTTGGCACAATTCTTAATCAATCCAGACCCATGGGTTGCCATTGCTGCCGCTGAAGTTTATGTCAGACGTTCATACCGTGCTTATGATTTGGGTAAAATTGAATATCATGTTAATGACAGACTTCCTATTGTTGAATGGAAATTCAAGTTGGCTAATATGGGAGCCGCTGGTGTAAACGATGCTCAACAGGCTGCTGCTGCCGGTGGCGATGATTCGACATCTATGAAACATGCAGCTTCTGTGTCTGATTTGACCTTTGTTGTTGATTATTCATAAAAGCGAAATCCCAGGATACACTCTCCCCGATAATCCAAAGTTCACCCTTGGTAATTTGTTTGTAATAATTGGAGTCTTGTTGGTTTGTATTTTAGCTGTTGTCTCTCTTTTGAGAAATATTAGTGAGTCGGCCTTGTTCAAGAAGAATGGGTATGAACCGTTGGATTCGGATCCTAGTGTCATGAACCTAAACTTCGAGCCTACAACATTGTCCTTTGAAGATATTAAATATGAGGTTACTGGTGGTCGACAAATTTTAAATGGAGTCTTTGGGTTTGTAAAACCAAGAGAATGTTTGGCTATAATGGGAGGTTCAGGTGCTGGTAAAACTACATTGTTGGATTTTAAACCTTTGGACCAATTGGCTAARACTTTGRCYACTGTTCCTGAATTGAATGAAATTATTGGKCAAGAWTTAGTTGACGAATTTRTCAGTGGTATTAAACTACCAGCAGAAGTTGGAAGTCAAGATGATGTTAACAATAGAAAATTGTTGCAAAAAGTGTTTGGTAAATTAATGAACACTGATGATGACGTTATAAAACAACAAACAGCTAAATTACTTGAAAGAACAGACAGAGAACCTCAAGTGTTCAAGGATATTGATTCTAGATTACCGGAGTTAATACAAAGATTAAACAAACAATTTCCTAATGACATCGGATTATTTTGTGGATGTCTCTTATTGAACCACGTTGATTGAAAAAATTATCAATTTCCGTGTTGGATAAACAGAGATTGACTGAAAAATTCAATAAATTGGATAAATCCATCAAAGATAATTTGAAGGCTAAACAAAAAGAAGAAACCAAAAAAACTTTAGATGTGGTTAATAATTGGTTGAATGATAAAGAAAATGCTTCATCATTTTTGGTTGCTCACGTTCCAATTACTGCTAATGCCAAGGCAATCACTGAAGCCATTAATTTGATTAAAAAGCAAGATAAAACCAAATCAATTTATTTATTGACTGGTGAAACCGATAAAGTTGCTCATGGATGTTATGTTAGTGATGAAGCCATTGCCAAGGGTATTAATGCGAAAATCAAACCTTTTGAAGCACGAGCAATTAACTGGTCCACGGATCTTAATGCTGAGGTACATATTGAGCATTATATAAATATATTCAATTATGCACGATCATCTTGGGAGCCATTGGTTGAAAGTTGGCCAATAGCAGTTTACATGTCAAAATCCCGACACCCAAAGCCTCAATTATTAGTAGAGGTGATTTCTAGACAGGTAGCYCAAGTGACGCTTACATCCAAAGCAGTAGCATTGCTATCTCAAGTATCCGATTTGATTACTTCCRGAGAAAAATTAAAACCAAGAGGTGAAGATTACCCATACGTKATAGTGAATGAGACTGGTTTAGATTTGGAAGTTTGGAATGATGCAAAWGAATYCGAAACCAATGGAGTTCCAATTGTTTTACGTGCTGGTAAAGCTTTAGATGAAAGTAAAGTTGAAATTAGAATTCAATTTAAACCAGTCGCCAAGGGGATGTTTAAAGAAATTCAAAGAAATGAATTAGTTATTAGAGTACAACCAAATGAAGCCATTTATTTAAAAATTAATTCCAAAATCCCTGGAATTTCTACTGAAACTTCATTAACTGATTTAGATTTAACTTATGCTACTCGTTATTCTAAAGAYTTTTGGATTCCTGAAGCTTATGAAGCATTAATTAGAGATTGYTATTTAGGTAATCATTCTAATTTTGTTAGAGATGATGAATTGGATGTTTCTTGGAAATTATTTACTCCATTATTGAAT

>hp12bt

AATTATTGAARTGGAATGATATTCCATTGGCTCCACCAGACAARATTTTGGGTATTTCTGAAGCTTATAACAATGATTCTAACCCTCAAAAARTCAATTTGGGGGTTGGTGCTTATAGAGATAATTCYGGTAAACCAATTATTTTCCCATCAGTTAAAAAAGCTGAAGAAATTTTATTGGGTAAAGAAACTGAAAAGGAATATACTGCCATTGTTGGTTCCAAAAATTTCCAATCAATTGTGAAAAATTTCATTTTCAACAATTCTAATAAAGATGCCAATGGTAAACAATTAATTGATGATGGTAGAATTGTTACTGCTCAAACCATCTCTGGTACTGGATCACTTAAACCTCTTATGGTGAAATTTTTGCTAAACATAGAGAACCAAATTTGGAAATTATTCGTGAGGTTGTTGATTCCAAACATATTGTTTTTGATGTGTTGGCACAATTCTTAATCAATCCAGACCCATGGGTTGCCATTGCTGCCGCTGAAGTTTATGTCAGACGTTCATACCGTGCTTATGATTTGGGTAMAATTGAATATCATGTTAATGACAGACTTCCTATTGTTGAATGGAAATTCAAGTTGGCTAATATGGGAGCCGCTGGTGTAAACGATGCTCAACAGGCTGCTGCTGCYGGTGGCGATGATTCGACATCTATGAAACATGCAGCTTCTGTGTCTGATTTGACCTTTGTTGTTGATTATTCATRAAAGCGAAATCCCAGGATACACTCTCCCCGATAATCCAAAGTTCACCCTTGGTAATTTGTTTGTAATAATTGGAGTCTTGTTGGTTTGTATTTTAGCTGTTGTCTCTCTTTTGAGAAATATTAGTGAGTCGGCCTTGTTCAAGAAGAATGGGTATGAACCGTTGGATTCGGATCCTAGTGTCATGAACCTAAACTTCGAGCCTACAACATTGTCCTTTGAAGATATTAAATATGAGGTTACTGGTGGTCGACAAATTTTAAATGGAGTCTTTGGGTTTGTAAAACCAAGAGAATGTTTGGCTATAATGGGAGGTTCAGGTGCTGGTAAAACTACATTGTTGGATTTTAAACCTTTGGACCAATTGGCTAAGACTTTGACCACTGTTCCTGAATTGAATGAAATTATTGGGCAAGATTTAGTTGACGAATTTGTCAGTGGTATTAAACTACCAGCAGAAGTTGGAAGTCAAGATGATGTTAACAATAGAAAATTGTTGCAAAAAGTGTTTGGTAAATTAATGAACACTGATGATGACGTTATAAAACAACAAACAGCTAAATTACTTGAAAGAACAGACAGAGAACCTCAAGTGTTCAAGGATATTGATTCTAGATTACCGGAGTTAATACAAAGATTAAACAAACAATTTCCTAATGACATCGGATTATTTTGTGGATGTCTCTTATTGAACCACGTTGATTGAAAAAATTATCAATTTCCGTGTTGGATAAACAGAGATTGACTGAAAAATTCAATAAATTGGATAAATCCATCAAAGATAATTTGAAGGCTAAACAAAAAGAAGAAACCAAAAAAACTTTAGATGTGGTTAATAATTGGTTGAATGATAAAGAAAATGCTTCATCATTTTTGGTTGCTCACGTTCCAATTACTGCTAATGCCAAGGCAATCACTGAAGCCATTAATTTGATTAAAAAGCAAGATAAAACCAAATCAATTTATTTATTGACTGGTGAAACCGATAAAGTTGCTCATGGATGTTATGTTAGTGATGAAGCCATTGCCAAGGGTATTAATGCGAAAATCAAACCTTTTGAAGCACGAGCAATTAACTGGTCCACGGATCTTAATGCTGAGGTACATATTGAGCATTATATAAATATATTCAATTATGCACGATCATCTTGGGAGCCATTGGTTGAAAGTTGGCCAATAGCAGTTTACATGTCAAAATCCCGACACCCAAAGCCTCAATTATTAGTAGAGGTGATTTCTAGACAGGTAGCYCAAGTGACGCTTACATCCAAAGCAGTAGCATTGCTATCTCAAGTATCCGATTTGATTACTTCCGGAGAAAAATTAAAACCAAGAGGTGAAGATTACCCATACGTKATAGTGAATGAGACTGGTTTAGATTTGGAAGTTTGGAATGATGCAAAWGAATYCGAAACCAATGGAGTTCCAATTGTTTTACGTGCTGGTAAAGCTTTAGATGAAAGTAAAGTTGAAATTAGAATTCAATTTAAACCAGTCGCCAAGGGGATGTTTAAAKAAATTCAAAGAAATGAATTAGTTATTAGAGTACAACCAAATGAAGCCATTTATTTAAAAATTAATTCCAAAATCCCTGGAATTTCTACTGAAACTTCATTAACTGATTTAGATTTAACTTATGCTACTCGTTATTCTAAAGAYTTTTGGATTCCTGAAGCTTATGAAGCATTAATTAGAGATTGYTATTTAGGTAATCATTCTAATTTTGTTAGAGATGATGAATTGGATGTTTCTTGGAAATTATTTACTCCATTATTGAAT

>hp13vu

AATTATTGAAATGGAATGATATTCCATTGGCTCCACCAGACAAAATTTTGGGTATTTCTGAAGCTTATAACAAYGATTCTAACCCTCAAAAAATCAATTTGGGGGTTGGTGCTTATAGAGATAATTCCGGTAAACCAATTATTTTCCCATCAGTTAAAAAAGCTGAAGAAATTTTATTGGGTAAAGAAACTGAAAAGGAATATACTGCCATTGTTGGTTCCAAAAATTTCCAATCAATTGTGAAAAATTTCATTTTCAACAATTCTAATAAAGATGCCAATGGTAAACAATTAATTGATGATGGTAGAATTGTTACTGCTCAAACCATCTCTGGTACTGGATCACTTAAACCTCTTATGGTGAAATTTTTGCTAAACATAGAGAACCAAATTTGGAAATTATTCGTGAGGTTGTTGATTCCAAACATATTGTTTTTGATGTGTTGGCACAATTCTTAATCAATCCAGACCCATGGGTTGCCATTGCTGCCGCTGAAGTTTATGTCAGACGTTCATACCGTGCTTATGATTTGGGTACAATTGAATATCATGTTAATGACAGACTTCCTATTGTTGAATGGAAATTCAAGTTGGCTAATATGGGAGCCGCTGGTGTAAACGATGCTCAACAGGCTGCTGCTGCTGGTGGCGATGATTCGACATCTATGAAACATGCAGCTTCTGTGTCTGATTTGACCTTTGTTGTTGATTATTCATAAAAGCGAAATCCCAGGATACACTCTCCCCGATAATCCAAAGTTCACCCTTGGTAATTTGTTTGTAATAATTGGAGTCTTGTTGGTTTGTATTTTAGCTGTTGTCTCTCTTTTGAGAAATATTAGTGAGTCGGCCTTGTTCAAGAAGAATGGGTATGAACCGTTGGATTCGGATCCTAGTGTCATGAACCTAAACTTCGAGCCTACAACATTGTCCTTTGAAGATATTAAATATGAGGTTACTGGTGGTCGACAAATTTTAAATGGAGTCTTTGGGTTTGTAAAACCAAGAGAATGTTTGGCTATAATGGGAGGTTCAGGTGCTGGTAAAACTACATTGTTGGATTTTAAACCTTTGGACCAATTGGCTAARACTTTGRCYACTGTTCCTGAATTGAATGAAATTATTGGKCAAGAWTTAGTTGACGAATTTRTCAGTGGTATTAAACTACCAGCAGAAGTTGGAAGTCAAGATGATGTTAACAATAGAAAATTGTTGCAAAAAGTGTTTGGTAAATTAATGAACACTGATGATGACGTTATAAAACAACAAACAGCTAAATTACTTGAAAGAACAGACAGAGAACCTCAAGTGTTCAAGGATATTGATTCTAGATTACCGGAGTTAATACAAAGATTAAACAAACAATTTCCTAATGACATCGGATTATTTTGTGGATGTCTCTTATTGAACCACGTTGMTTGAAAAAATTATCAATTTCCGTGTTGGATAAACAAAGATTGACTGAAAAATTCAATAAATTGGATAAATCCATYAAAGATAATTTGAAGGCTAAACAAAAAGAAGAAACCAAAAAGACTTTAGATGTGGTTAACAATTGGTTGAATGATAAAGAAAATGCTTCATCATTTTTGGTTGCTCACGTTCCAATTACTGCTAATGCCAAGGCAATCACTGAAGCCATTAATTTGATTAAAAAGCAAGATAAAACCAAATCAATTTATTTATTGACTGGTGAAACCGATAAAGTTGCTCATGGATGTTATGTTAGTGATGAAGCCATTGTCAAGGGTATTAATGCGAAAATCAAACCTTTTGAAGCACGAGCAATTAACTGGTCCACGGATCTTAATGCTGAGGTACATATTGAGCATTATATAAATATATTCAATTATGCACGATCATCTTGGGAGCCATTGGTTGAAAGTTGGCCAATAGCAGTTTACATGTCAAAATCCCGACACCCAAAGCCTCAATTATTAGTAGAGGTGATTTCTAGACAGGTAGCTCAAGTGACGCTTACATCCAAAGCGGTAGCATTGCTATCTCAAGTATCCGATTTGATTACTTCCGGAGAAAAATTAAAACCAAGAGGTGAAGATTACCCATACGTTATAGTGAATGAGACTGGTTTAGATTTGGAAGTTTGGAATGATGCAAATGAATCCGAAACCAATGGAGTTCCAATTGTTTTACGTGCTGGTAAAGCTTTAGATGAAAGTAAAGTTGAAATTAGAATTCAATTTAAACCAGTCGCCAAGGGGATGTTTAAAGAAATTCAAAGAAATGAATTAGTTATTAGAGTACAACCAAATGAAGCCATTTATTTAAAAATTAATTCCAAAATCCCTGGAATTTCTACTGAAACTTCATTAACTGATTTAGATTTAACTTATGCTACTCGTTATTCTAAAGACTTTTGGATTCCTGAAGCTTATGAAGCATTAATTAGAGATTGCTATTTAGGTAATCATTCTAATTTTGTTAGAGATGATGAATTGGATGTTTCTTGGAAATTATTTACTCCATTATTGAAT

>hp13vw

AATTATTGAAATGGAATGATATTCCATTGGCTCCACCAGACAAAATTTTGGGTATTTCTGAAGCTTATAACAAYGATTCTAACCCTCAAAAAATCAATTTGGGGGTTGGTGCTTATAGAGATAATTCCGGTAAACCAATTATTTTCCCATCAGTTAAAAAAGCTGAAGAAATTTTATTGGGTAAAGAAACTGAAAAGGAATATACTGCCATTGTTGGTTCCAAAAATTTCCAATCAATTGTGAAAAATTTCATTTTCAACAATTCTAATAAAGATGCCAATGGTAAACAATTAATTGATGATGGTAGAATTGTTACTGCTCAAACCATCTCTGGTACTGGATCACTTAAACCTCTTATGGTGAAATTTTKGCTAAACATAGAGAACCAAATTTGGAAATTATTCGTGAGGTTGTTGATTCCAAACATATTGTTTTTGATGTGTTGGCACAATTCTTAATCAATCCAGACCCATGGGTTGCCATTGCTGCCGCTGAAGTTTATGTCAGACGTTCATACCGTGCTTATGATTTGGGTACAATTGAATATCATGTTAATGACAGACTTCCTATTGTTGAATGGAAATTCAAGTTGGCTAATATGGGAGCCGCTGGTGTAAACGATGCTCAACAGGCTGCTGCTGCTGGTGGCGATGATTCGACATCTATGAAACATGCAGCTTCTGTGTCTGATTTGACCTTTGTTGTTGATTATTCATAAAAGCGAAATCCCAGGATACACTCTCCCCGATAATCCAAAGTTCACCCTTGGTAATTTGTTTGTAATAATTGGAGTCTTGTTGGTTTGTATTTTAGCTGTTGTCTCTCTTTTGAGAAATATTAGTGAGTCGGCCTTGTTCAAGAAGAATGGGTATGAACCGTTGGATTCGGATCCTAGTGTCATGAACCTAAACTTCGAGCCTACAACATTGTCCTTTGAAGATATTAAATATGAGGTTACTGGTGGTCGACAAATTTTAAATGGAGTCTTTGGGTTTGTAAAACCAAGAGAATGTTTGGCTATAATGGGAGGTTCAGGTGCTGGTAAAACTACATTGTTGGATTTTAAACCTTTGGACCAATTGGCTAARACTTTGRCYACTGTTCCTGAATTGAATGAAATTATTGGKCAAGAWTTAGTTGACGAATTTRTCAGTGGTATTAAACTACCAGCAGAAGTTGGAAGTCAAGATGATGTTAACAATAGAAAATTGTTGCAAAAAGTGTTTGGTAAATTAATGAACACTGATGATGACGTTATAAAACAACAAACAGCTAAATTACTTGAAAGAACAGACAGAGAACCTCAAGTGTTCAAGGATATTGATTCTAGATTACCGGAGTTAATACAAAGATTAAACAAACAATTTCCTAATGACATCGGATTATTTTGTGGATGTCTCTTATTGAACCACGTTGMTTGAAAAAATTATCAATTTCCGTGTTGGATAAACAAAGATTGACTGAAAAATTCAATAAATTGGATAAATCCATYAAAGATAATTTGAAGGCTAAACAAAAAGAAGAAACCAAAAARACTTTAGATGTGGTTAAYAATTGGTTGAATGATAAAGAAAATKCTTCATCATTTTTGGTTGCTCACGTTCCAATTACTGCTAATGCCAAGGCAATCACTGAAGCCATTAATTTGATTAAAAAGCAAGATAAAACCAAATCAATTTATTTATTGACTGGTGAAACCGATAAAGTTGCTCATGGATGTTATGTTAGTGATGAAGCCATTGTCAAGGGTATTAATGCGAAAATCAAACCTTTTGAAGCACGAGCAATTAACTGGTCCACGGATCTTAATGCTGAGGTACATATTGAGCATTATATAAATATATTCAATTATGCACGATCATCTTGGGAGCCATTGGTTGAAAGTTGGCCAATAGCAGTTTACATGTCAAAATCCCGACACCCAAAGCCTCAATTATTAGTAGAGGTGATTTCTAGACAGGTAGCTCAAGTGACGCTTACATCCAAAGCGGTAGCATTGCTATCTCAAGTATCCGATTTGATTACTTCCGGAGAAAAATTAAAACCAAGAGGTGAAGATTACCCATACGTTATAGTGAATGAGACTGGTTTAGATTTGGAAGTTTGGAATGATGCAAATGAATCCGAAACCAATGGAGTTCCAATTGTTTTACGTGCTGGTAAAGCTTTAGATGAAAGTAAAGTTGAAATTAGAATTCAATTTAAACCAGTCGCCAAGGGGATGTTTAAAGAAATTCAAAGAAATGAATTAGTTATTAGAGTACAACCAAATGAAGCCATTTATTTAAAAATTAATTCCAAAATCCCTGGAATTTCTACTGAAACTTCATTAACTGATTTAGATTTAACTTATGCTACTCGTTATTCTAAAGACTTTTGGATTCCTGAAGCTTATGAAGCATTAATTAGAGATTGCTATTTAGGTAATCATTCTAATTTTGTTAGAGATGATGAATTGGATGTTTCTTGGAAATTATTTACTCCATTATTGAAT

>hp25bt

AATTATTGAAATGGAATGATATTCCATTGGCTCCACCAGACAAAATTTTGGGTATTTCTGAAGCTTATAACAACGATTCTAACCCTCAAAAAATCAATTTGGGGGTTGGTGCTTATAGAGATAATTCCGGTAAACCAATTATTTTCCCATCAGTTAAAAAAGCTGAAGAAATTTTATTGGGTAAAGAAACTGAAAAGGAATATACTGCCATTGTTGGTTCCAAAAATTTCCAATCAATTGTGAAAAATTTCATTTTCAACAATTCTAATAAAGATGCCAATGGTAAACAATTAATTGATGATGGTAGAATTGTTACTGCTCAAACCATCTCTGGTACTGGATCACTTAAACCTCTTATGGTGAAATTTTTGCTAAACATAGAGAACCAAATTTGGAAATTATTCGTGAGGTTGTTGATTCCAAACATATTGTTTTTGATGTGTTGGCACAATTCTTAATCAATCCAGACCCATGGGTTGCCATTGCTGCCGCTGAAGTTTATGTCAGACGTTCATACCGTGCTTATGATTTGGGTAMAATTGAATATCATGTTAATGACAGACTTCCTATTGTTGAATGGAAATTCAAGTTGGCTAATATGGGAGCYGCTGGTGTAAACGATGCTCAACAGGCTGCTGCTGCYGGTGGCGATGATTCGACATCTATGAAACATGCAGCTTCTGTGTCTGATTTGACCTTTGTTGTTGATTATTCATAAAAGCGAAATCCCAGGATACACTCTCCCCGATAATCCAAAGTTCACCCTTGGTAATTTGTTTGTAATAATTGGAGTCTTGTTGGTTTGTGTTTTAGCTGTTGTCTCTCTTTTGAGAAATATTAGTGAGTCGGCCTTGTTCAAGAAGAATGGGTATGAACCGTTGGATTCGGATCCTAGTGTCATGAACCTAAACTTCGAGCCTACAACATTGTCCTTTGAAGATATTAAATATGAGGTTACTGGTGGTCGACAAATTTTAAATGGAGTCTTTGGGTTTGTAAAACCAAGAGAATGTTTGGCTATAATGGGAGGTTCAGGTGCTGGTAAAACTACATTGTTGGATTTTAAACCTTTGGACCAATTGGCTAAGACTTTGACCACTGTTCCTGAATTGAATGAAATTATTGGGCAAGATTTAGTTGACGAATTTGTCAGTGGTATTAAACTACCAGCAGAAGTTGGAAGTCAAGATGATGTTAACAATAGAAAATTGTTGCAAAAAGTGTTTGGTAAATTAATGAACACTGATGATGACGTTATAAAACAACAAACAGCTAAATTACTTGAAAGAACAGACAGAGAACCTCAAGTGTTCAAGGATATTGATTCTAGATTACCGGAGTTAATACAAAGATTAAACAAACAATTTCCTAATGACATCGGATTATTTTGTGGATGTCTCTTATTGAACCACGTTGMTTGAAAAAATTATCAATTTCCGTGTTGGATAAACAAAGATTGACTGAAAAATTCAATAAATTGGATAAATCCATYAAAGATAATTTGAAGGCTAAACAAAAAGAAGAAACCAAAAARACTTTAGATGTGGTTAAYAATTGGTTGAATGATAAAGAAAATKCTTCATCATTTTTGGTTGCTCACGTTCCAATTACTGCTAATGCCAAGGCAATCACTGAAGCCATTAATTTGATTAAAAAGCAAGATAAAACCAAATCAATTTATTTATTGACTGGTGAAACCGATAAAGTTGCTCATGGATGTTATGTTAGTGATGAAGCCATTGTCAAGGGTATTAATGCGAAAATCAAACCTTTTGAAGCACGAGCAATTAACTGGTCCACGGATCTTAATGCTGAGGTACATATTGAGCATTATATAAATATATTCAATTATGCACGATCATCTTGGGAGCCATTGGTTGAAAGTTGGCCAATAGCAGTTTACATGTCAAAATCCCGACACCCAAAGCCTCAATTATTAGTAGAGGTGATTTCTAGACAGGTAGCTCAAGTGACGCTTACATCCAAAGCGGTAGCATTGCTATCTCAAGTATCCGATTTGATTACTTCCGGAGAAAAATTAAAACCAAGAGGTGAAGATTACCCATACGTTATAGTGAATGAGACTGGTTTAGATTTGGAAGTTTGGAATGATGCAAATGAATCCGAAACCAATGGAGTTCCAATTGTTTTACGTGCTGGTAAAGCTTTAGATGAAAGTAAAGTTGAAATTAGAATTCAATTTAAACCAGTCGCCAAGGGGATGTTTAAAGAAATTCAAAGAAATGAATTAGTTATTAGAGTACAACCAAATGAAGCCATTTATTTAAAAATTAATTCCAAAATCCCTGGAATTTCTACTGAAACTTCATTAACTGATTTAGATTTAACTTATGCTACTCGTTATTCTAAAGACTTTTGGATTCCTGAAGCTTATGAAGCATTAATTAGAGATTGYTATTTAGGTAATCATTCTAATTTTGTTAGAGATGATGAATTGGATGTTTCTTGGAAATTATTTACTCCATTATTGAAT

>hp25st

AATTATTGAAATGGAATGATATTCCATTGGCTCCACCAGACAAAATTTTGGGTATTTCTGAAGCTTATAACAACGATTCTAACCCTCAAAAAATCAATTTGGGGGTTGGTGCTTATAGAGATAATTCCGGTAAACCAATTATTTTCCCATCAGTTAAAAAAGCTGAAGAAATTTTATTGGGTAAAGAAACTGAAAAGGAATATACTGCCATTGTTGGTTCCAAAAATTTCCAATCAATTGTGAAAAATTTCATTTTCAACAATTCTAATAAAGATGCCAATGGTAAACAATTAATTGATGATGGTAGAATTGTTACTGCTCAAACCATCTCTGGTACTGGATCACTTAAACCTCTTATGGTGAAATTTTKGCTAAACATAGAGAACCAAATTTGGAAATTATTCGTGAGGTTGTTGATTCCAAACATATTGTTTTTGATGTGTTGGCACAATTCTTAATCAATCCAGACCCATGGGTTGCCATTGCTGCCGCTGAAGTTTATGTCAGACGTTCATACCGTGCTTATGATTTGGGTAMAATTGAATATCATGTTAATGACAGACTTCCTATTGTTGAATGGAAATTCAAGTTGGCTAATATGGGAGCYGCTGGTGTAAACGATGCTCAACAGGCTGCTGCTGCYGGTGGCGATGATTCGACATCTATGAAACATGCAGCTTCTGTGTCTGATTTGACCTTTGTTGTTGATTATTCATAAAAGCGAAATCCCAGGATACACTCTCCCCGATAATCCAAAGTTCACCCTTGGTAATTTGTTTGTAATAATTGGAGTCTTGTTGGTTTGTGTTTTAGCTGTTGTCTCTCTTTTGAGAAATATTAGTGAGTCGGCCTTGTTCAAGAAGAATGGGTATGAACCGTTGGATTCGGATCCTAGTGTCATGAACCTAAACTTCGAGCCTACAACATTGTCCTTTGAAGATATTAAATATGAGGTTACTGGTGGTCGACAAATTTTAAATGGAGTCTTTGGGTTTGTAAAACCAAGAGAATGTTTGGCTATAATGGGAGGTTCAGGTGCTGGTAAAACTACATTGTTGGATTTTAAACCTTTGGACCAATTGGCTAAGACTTTGACCACTGTTCCTGAATTGAATGAAATTATTGGGCAAGATTTAGTTGACGAATTTGTCAGTGGTATTAAACTACCAGCAGAAGTTGGAAGTCAAGATGATGTTAACAATAGAAAATTGTTGCAAAAAGTGTTTGGTAAATTAATGAACACTGATGATGACGTTATAAAACAACAAACAGCTAAATTACTTGAAAGAACAGACAGAGAACCTCAAGTGTTCAAGGATATTGATTCTAGATTACCGGAGTTAATACAAAGATTAAACAAACAATTTCCTAATGACATCGGATTATTTTGTGGATGTCTCTTATTGAACCACGTTGMTTGAAAAAATTATCAATTTCCGTGTTGGATAAACAAAGATTGACTGAAAAATTCAATAAATTGGATAAATCCATYAAAGATAATTTGAAGGCTAAACAAAAAGAAGAAACCAAAAARACTTTAGATGTGGTTAAYAATTGGTTGAATGATAAAGAAAATKCTTCATCATTTTTGGTTGCTCACGTTCCAATTACTGCTAATGCCAAGGCAATCACTGAAGCCATTAATTTGATTAAAAAGCAAGATAAAACCAAATCAATTTATTTATTGACTGGTGAAACCGATAAAGTTGCTCATGGATGTTATGTTAGTGATGAAGCCATTGTCAAGGGTATTAATGCGAAAATCAAACCTTTTGAAGCACGAGCAATTAACTGGTCCACGGATCTTAATGCTGAGGTACATATTGAGCATTATATAAATATATTCAATTATGCACGATCATCTTGGGAGCCATTGGTTGAAAGTTGGCCAATAGCAGTTTACATGTCAAAATCCCGACACCCAAAGCCTCAATTATTAGTAGAGGTGATTTCTAGACAGGTAGCTCAAGTGACGCTTACATCCAAAGCGGTAGCATTGCTATCTCAAGTATCCGATTTGATTACTTCCGGAGAAAAATTAAAACCAAGAGGTGAAGATTACCCATACGTTATAGTGAATGAGACTGGTTTAGATTTGGAAGTTTGGAATGATGCAAATGAATCCGAAACCAATGGAGTTCCAATTGTTTTACGTGCTGGTAAAGCTTTAGATGAAAGTAAAGTTGAAATTAGAATTCAATTTAAACCAGTCGCCAAGGGGATGTTTAAAGAAATTCAAAGAAATGAATTAGTTATTAGAGTACAACCAAATGAAGCCATTTATTTAAAAATTAATTCCAAAATCCCTGGAATTTCTACTGAAACTTCATTAACTGATTTAGATTTAACTTATGCTACTCGTTATTCTAAAGACTTTTGGATTCCTGAAGCTTATGAAGCATTAATTAGAGATTGYTATTTAGGTAATCATTCTAATTTTGTTAGAGATGATGAATTGGATGTTTCTTGGAAATTATTTACTCCATTATTGAAT

>hp31an

AATTATTGAARTGGAATGATATTCCATTGGCTCCACCAGACAARATTTTGGGTATTTCTGAAGCTTATAACAATGATTCTAACCCTCAAAAAGTCAATTTGGGGGTTGGTGCTTATAGAGATAATTCTGGTAAACCAATTATTTTCCCATCAGTTAAAAAAGCTGAAGAAATTTTATTGGGTAAAGAAACTGAAAAGGAATATACTGCCATTGTTGGTTCCAAAAATTTCCAATCAATTGTGAAAAATTTCATTTTCAACAATTCTAATAAAGATGCCAATGGTAAACAATTAATTGATGATGGTAGAATTGTTACTGCTCAAACCATCTCTGGTACTGGATCACTTAAACCTCTTATGGTGAAATTTTTGCTAAACATAGAGAACCAAATTTGGAAATTATTCGTGAGGTTGTTGATTCCAAACATATTGTTTTTGATGTGTTGGCACAATTCTTAATCAATCCAGACCCATGGGTTGCCATTGCTGCCGCTGAAGTTTATGTCAGACGTTCATACCGTGCTTATGATTTGGGTAAAATTGAATATCATGTTAATGACAGACTTCCTATTGTTGAATGGAAATTCAAGTTGGCTAATATGGGAGCCGCTGGTGTAAACGATGCTCAACAGGCTGCTGCTGCCGGTGGCGATGATTCGACATCTATGAAACATGCAGCTTCTGTGTCTGATTTGACCTTTGTTGTTGATTATTCATRAAAGCGAAATCCCAGGATACACTCTCCCCGATAATCCAAAGTTCACCCTTGGTAATTTGTTTGTAATAATTGGAGTCTTGTTGGTTTGTATTTTAGCTGTTGTCTCTCTTTTGAGAAATATTAGTGAGTCGGCCTTGTTCAAGAAGAATGGGTATGAACCGTTGGATTCGGATCCTAGTGTCATGAACCTAAACTTCGAGCCTACAACATTGTCCTTTGAAGATATTAAATATGAGGTTACTGGTGGTCGACAAATTTTAAATGGAGTCTTTGGGTTTGTAAAACCAAGAGAATGTTTGGCTATAATGGGAGGTTCAGGTGCTGGTAAAACTACATTGTTGGATTTTAAACCTTTGGACCAATTGGCTAARACTTTGRCYACTGTTCCTGAATTGAATGAAATTATTGGKCAAGAWTTAGTTGACGAATTTRTCAGTGGTATTAAACTACCAGCAGAAGTTGGAAGTCAAGATGATGTTAACAATAGAAAATTGTTGCAAAAAGTGTTTGGTAAATTAATGAACACTGATGATGACGTTATAAAACAACAAACAGCTAAATTACTTGAAAGAACAGACAGAGAACCTCAAGTGTTCAAGGATATTGATTCTAGATTACCGGAGTTAATACAAAGATTAAACAAACAATTTCCTAATGACATCGGATTATTTTGTGGATGTCTCTTATTGAACCACGTTGATTGAAAAAATTATCAATTTCCGTGTTGGATAAACAGAGATTGACTGAAAAATTCAATAAATTGGATAAATCCATCAAAGATAATTTGAAGGCTAAACAAAAAGAAGAAACCAAAAAAACTTTAGATGTGGTTAATAATTGGTTGAATGATAAAGAAAATGCTTCATCATTTTTGGTTGCTCACGTTCCAATTACTGCTAATGCCAAGGCAATCACTGAAGCCATTAATTTGATTAAAAAGCAAGATAAAACCAAATCAATTTATTTATTGACTGGTGAAACCGATAAAGTTGCTCATGGATGTTATGTTAGTGATGAAGCCATTGCCAAGGGTATTAATGCGAAAATCAAACCTTTTGAAGCACGAGCAATTAACTGGTCCACGGATCTTAATGCTGAGGTACATATTGAGCATTATATAAATATATTCAATTATGCACGATCATCTTGGGAGCCATTGGTTGAAAGTTGGCCAATAGCAGTTTACATGTCAAAATCCCGACACCCAAAGCCTCAATTATTAGTAGAGGTGATTTCTAGACAGGTAGCYCAAGTGACGCTTACATCCAAAGCAGTAGCATTGCTATCTCAAGTATCCGATTTGATTACTTCCRGAGAAAAATTAAAACCAAGAGGTGAAGATTACCCATACGTKATAGTGAATGAGACTGGTTTAGATTTGGAAGTTTGGAATGATGCAAAWGAATYCGAAACCAATGGAGTTCCAATTGTTTTACGTGCTGGTAAAGCTTTAGATGAAAGTAAAGTTGAAATTAGAATTCAATTTAAACCAGTCGCCAAGGGGATGTTTAAAGAAATTCAAAGAAATGAATTAGTTATTAGAGTACAACCAAATGAAGCCATTTATTTAAAAATTAATTCCAAAATCCCTGGAATTTCTACTGAAACTTCATTAACTGATTTAGATTTAACTTATGCTACTCGTTATTCTAAAGAYTTTTGGATTCCTGAAGCTTATGAAGCATTAATTAGAGATTGYTATTTAGGTAATCATTCTAATTTTGTTAGAGATGATGAATTGGATGTTTCTTGGAAATTATTTACTCCATTATTGAAT

>hp36bt

AATTATTGAAATGGAATGATATTCCATTGGCTCCACCAGACAAAATTTTGGGTATTTCTGAAGCTTATAACAATGATTCTAACCCTCAAAAAATCAATTTGGGGGTTGGTGCTTATAGAGATAATTCCGGTAAACCAATTATTTTCCCATCAGTTAAAAAAGCTGAAGAAATTTTATTGGGTAAAGAAACTGAAAAGGAATATACTGCCATTGTTGGTTCCAAAAATTTCCAATCAATTGTGAAAAATTTCATTTTCAACAATTCTAATAAAGATGCCAATGGTAAACAATTAATTGATGATGGTAGAATTGTTACTGCTCAAACCATCTCTGGTACTGGATCACTTAAACCTCTTATGGTGAAATTTTKGCTAAACATAGAGAACCAAATTTGGAAATTATTCGTGAGGTTGTTGATTCCAAACATATTGTTTTTGATGTGTTGGCACAATTCTTAATCAATCCAGACCCATGGGTTGCCATTGCTGCCGCTGAAGTTTATGTCAGACGTTCATACCGTGCTTATGATTTGGGTAAAATTGAATATCATGTTAATGACAGACTTCCTATTGTTGAATGGAAATTCAAGTTGGCTAATATGGGAGCCGCTGGTGTAAACGATGCTCAACAGGCTGCTGCTGCCGGTGGCGATGATTCGACATCTATGAAACATGCAGCTTCTGTGTCTGATTTGACCTTTGTTGTTGATTATTCATAAAAGYGAAATYCCAGGATACACTCTCCCCGATAATCCAAAGTTCACCCTTGGTAATTTGTTTGTAATAATTGGRGTCTTGTTGGTTTGTGTTTTAGCTGTTGTCTCTCTTTTGAGAAATATTAGTGAGTCRGCCTTGTTCAAGAAGAATGGGTATGAACCGTTGGATTCRGATCCTAGTGTCATGAACCWAAACTTYGAGCCTACAACATTGTCCTTTGAAGATATTAAATATGAGGTTACTGGTGGTCGACAAATTTTAAATGGAGTCTTTGGGTTTGTAAAACCAAGAGAATGTTTGGCTATAATGGGAGGTTCAGGTGCTGGTAAAACTACATTGTTGGATTTTAAACCTTTGGACCAATTGGCTAAGACTTTGACCACTGTTCCTGAATTGAATGAAATTATTGGGCAAGATTTAGTTGACGAATTTGTCAGTGGTATTAAACTACCAGCAGAAGTTGGAAGTCAAGATGATGTTAACAATAGAAAATTGTTGCAAAAAGTGTTTGGTAAATTAATGAACACTGATGATGACGTTATAAAACAACAAACAGCTAAATTACTTGAAAGAACAGACAGAGAACCTCAAGTGTTCAAGGATATTGATTCTAGATTACCGGAGTTAATACAAAGATTAAACAAACAATTTCCTAATGACATCGGATTATTTTGTGGATGTCTCTTATTGAACCACGTTGMTTGAAAAAATTATCAATTTCCGTGTTGGATAAACARAGATTGACTGAAAAATTCAATAAATTGGATAAATCCATYAAAGATAATTTGAAGGCTAAACAAAAAGAAGAAACCAAAAARACTTTAGATGTGGTTAAYAATTGGTTGAATGATAAAGAAAATGCTTCATCATTTTTGGTTGCTCACGTTCCAATTACTGCTAATGCCAAGGCAATCACTGAAGCCATTAATTTGATTAAAAAGCAAGATAAAACCAAATCAATTTATTTATTGACTGGTGAAACCGATAAAGTTGCTCATGGATGTTATGTTAGTGATGAAGCCATTGYCAAGGGTATTAATGCGAAAATCAAACCTTTTGAAGCACGAGCAATTAACTGGTCMACGGATCTTAATGCTGAGGTACATATTGAGCATTATATAAATATATTCAATTATGCACGATCATCTTGGGAGCCATTGGTTGAAAGTTGGCCAATAGCAGTTTACATGTCAAAATCCCGACACCCAAAGCCTCAATTATTAGTAGAGGTGATTTCTAGACAGGTAGCTCAAGTGACGCTTACATCCAAAGCRGTAGCATTGCTATCTCAAGTATCCGATTTGATTACTTCCGGAGAAAAATTAAAACCAAGAGGTGAAGATTACCCATACRTTATAGTGAATGAGACTGGTTTAGATTTGGAAGTTTGGAATGATGCAAAWGAATCCGAAACCAATGGAGTTCCAATTGTTTTACGTGCTGGTAAAGCTTTAGATGAAAGTAAAGTTGAAATTAGAATTCAATTTAAACCAGTCGCCAAGGGGATGTTTAAAGAAATTCAAAGAAATGAATTAGTTATTAGAGTACAACCAAATGAAGCCATTTATTTAAAAATTAATTCCAAAATCCCTGGAATTTCTACTGAAACTTCATTAACTGATTTAGATTTAACTTATGCTACTCGTTATTCTAAAGATTTTTGGATTCCTGAAGCTTATGAAGCATTAATTAGAGATTGTTATTTAGGTAATCATTCTAATTTTGTTAGAGATGATGAATTGGATGTTTCTTGGAAATTATTTACTCCATTATTGAAT

>hp36vu

AATTATTGAAATGGAATGATATTCCATTGGCTCCACCAGACAAAATTTTGGGTATTTCTGAAGCTTATAACAATGATTCTAACCCTCAAAAAATCAATTTGGGGGTTGGTGCTTATAGAGATAATTCCGGTAAACCAATTATTTTCCCATCAGTTAAAAAAGCTGAAGAAATTTTATTGGGTAAAGAAACTGAAAAGGAATATACTGCCATTGTTGGTTCCAAAAATTTCCAATCAATTGTGAAAAATTTCATTTTCAACAATTCTAATAAAGATGCCAATGGTAAACAATTAATTGATGATGGTAGAATTGTTACTGCTCAAACCATCTCTGGTACTGGATCACTTAAACCTCTTATGGTGAAATTTTTGCTAAACATAGAGAACCAAATTTGGAAATTATTCGTGAGGTTGTTGATTCCAAACATATTGTTTTTGATGTGTTGGCACAATTCTTAATCAATCCAGACCCATGGGTTGCCATTGCTGCCGCTGAAGTTTATGTCAGACGTTCATACCGTGCTTATGATTTGGGTAAAATTGAATATCATGTTAATGACAGACTTCCTATTGTTGAATGGAAATTCAAGTTGGCTAATATGGGAGCCGCTGGTGTAAACGATGCTCAACAGGCTGCTGCTGCCGGTGGCGATGATTCGACATCTATGAAACATGCAGCTTCTGTGTCTGATTTGACCTTTGTTGTTGATTATTCATAAAAGYGAAATYCCAGGATACACTCTCCCCGATAATCCAAAGTTCACCCTTGGTAATTTGTTTGTAATAATTGGRGTCTTGTTGGTTTGTGTTTTAGCTGTTGTCTCTCTTTTGAGAAATATTAGTGAGTCRGCCTTGTTCAAGAAGAATGGGTATGAACCGTTGGATTCRGATCCTAGTGTCATGAACCWAAACTTYGAGCCTACAACATTGTCCTTTGAAGATATTAAATATGAGGTTACTGGTGGTCGACAAATTTTAAATGGAGTCTTTGGGTTTGTAAAACCAAGAGAATGTTTGGCTATAATGGGAGGTTCAGGTGCTGGTAAAACTACATTGTTGGATTTTAAACCTTTGGACCAATTGGCTAAGACTTTGACCACTGTTCCTGAATTGAATGAAATTATTGGGCAAGATTTAGTTGACGAATTTGTCAGTGGTATTAAACTACCAGCAGAAGTTGGAAGTCAAGATGATGTTAACAATAGAAAATTGTTGCAAAAAGTGTTTGGTAAATTAATGAACACTGATGATGACGTTATAAAACAACAAACAGCTAAATTACTTGAAAGAACAGACAGAGAACCTCAAGTGTTCAAGGATATTGATTCTAGATTACCGGAGTTAATACAAAGATTAAACAAACAATTTCCTAATGACATCGGATTATTTTGTGGATGTCTCTTATTGAACCACGTTGMTTGAAAAAATTATCAATTTCCGTGTTGGATAAACARAGATTGACTGAAAAATTCAATAAATTGGATAAATCCATYAAAGATAATTTGAAGGCTAAACAAAAAGAAGAAACCAAAAARACTTTAGATGTGGTTAAYAATTGGTTGAATGATAAAGAAAATGCTTCATCATTTTTGGTTGCTCACGTTCCAATTACTGCTAATGCCAAGGCAATCACTGAAGCCATTAATTTGATTAAAAAGCAAGATAAAACCAAATCAATTTATTTATTGACTGGTGAAACCGATAAAGTTGCTCATGGATGTTATGTTAGTGATGAAGCCATTGYCAAGGGTATTAATGCGAAAATCAAACCTTTTGAAGCACGAGCAATTAACTGGTCMACGGATCTTAATGCTGAGGTACATATTGAGCATTATATAAATATATTCAATTATGCACGATCATCTTGGGAGCCATTGGTTGAAAGTTGGCCAATAGCAGTTTACATGTCAAAATCCCGACACCCAAAGCCTCAATTATTAGTAGAGGTGATTTCTAGACAGGTAGCTCAAGTGACGCTTACATCCAAAGCRGTAGCATTGCTATCTCAAGTATCCGATTTGATTACTTCCGGAGAAAAATTAAAACCAAGAGGTGAAGATTACCCATACRTTATAGTGAATGAGACTGGTTTAGATTTGGAAGTTTGGAATGATGCAAAWGAATCCGAAACCAATGGAGTTCCAATTGTTTTACGTGCTGGTAAAGCTTTAGATGAAAGTAAAGTTGAAATTAGAATTCAATTTAAACCAGTCGCCAAGGGGATGTTTAAAGAAATTCAAAGAAATGAATTAGTTATTAGAGTACAACCAAATGAAGCCATTTATTTAAAAATTAATTCCAAAATCCCTGGAATTTCTACTGAAACTTCATTAACTGATTTAGATTTAACTTATGCTACTCGTTATTCTAAAGATTTTTGGATTCCTGAAGCTTATGAAGCATTAATTAGAGATTGTTATTTAGGTAATCATTCTAATTTTGTTAGAGATGATGAATTGGATGTTTCTTGGAAATTATTTACTCCATTATTGAAT

>hp38an

AATTATTGAARTGGAATGATATTCCATTGGCTCCACCAGACAARATTTTGGGTATTTCTGAAGCTTATAACAATGATTCTAACCCTCAAAAARTCAATTTGGGGGTTGGTGCTTATAGAGATAATTCTGGTAAACCAATTATTTTCCCATCAGTTAAAAAAGCTGAAGAAATTTTATTGGGTAAAGAAACTGAAAAGGAATATACTGCCATTGTTGGTTCCAAAAATTTCCAATCAATTGTGAAAAATTTCATTTTCAACAATTCTAATAAAGATGCCAATGGTAAACAATTAATTGATGATGGTAGAATTGTTACTGCTCAAACCATCTCTGGTACTGGATCACTTAAACCTCTTATGGTGAAATTTTTGCTAAACATAGAGAACCAAATTTGGAAATTATTCGTGAGGTTGTTGATTCCAAACATATTGTTTTTGATGTGTTGGCACAATTCTTAATCAATCCAGACCCATGGGTTGCCATTGCTGCCGCTGAAGTTTATGTCAGACGTTCATACCGTGCTTATGATTTGGGTAAAATTGAATATCATGTTAATGACAGACTTCCTATTGTTGAATGGAAATTCAAGTTGGCTAATATGGGAGCCGCTGGTGTAAACGATGCTCAACAGGCTGCTGCTGCYGGTGGCGATGATTCGACATCTATGAAACATGCAGCTTCTGTGTCTGATTTGACCTTTGTTGTTGATTATTCATRAAAGCGAAATCCCAGGATACACTCTCCCCGATAATCCAAAGTTCACCCTTGGTAATTTGTTTGTAATAATTGGAGTCTTGTTGGTTTGTATTTTAGCTGTTGTCTCTCTTTTGAGAAATATTAGTGAGTCGGCCTTGTTCAAGAAGAATGGGTATGAACCGTTGGATTCGGATCCTAGTGTCATGAACCTAAACTTCGAGCCTACAACATTGTCCTTTGAAGATATTAAATATGAGGTTACTGGTGGTCGACAAATTTTAAATGGAGTCTTTGGGTTTGTAAAACCAAGAGAATGTTTGGCTATAATGGGAGGTTCAGGTGCTGGTAAAACTACATTGTTGGATTTTAAACCTTTGGACCAATTGGCTAARACTTTGRCYACTGTTCCTGAATTGAATGAAATTATTGGKCAAGAWTTAGTTGACGAATTTRTCAGTGGTATTAAACTACCAGCAGAAGTTGGAAGTCAAGATGATGTTAACAATAGAAAATTGTTGCAAAAAGTGTTTGGTAAATTAATGAACACTGATGATGACGTTATAAAACAACAAACAGCTAAATTACTTGAAAGAACAGACAGAGAACCTCAAGTGTTCAAGGATATTGATTCTAGATTACCGGAGTTAATACAAAGATTAAACAAACAATTTCCTAATGACATCGGATTATTTTGTGGATGTCTCTTATTGAACCACGTTGATTGAAAAAATTATCAATTTCCGTGTTGGATAAACAGAGATTGACTGAAAAATTCAATAAATTGGATAAATCCATCAAAGATAATTTGAAGGCTAAACAAAAAGAAGAAACCAAAAAAACTTTAGATGTGGTTAATAATTGGTTGAATGATAAAGAAAATGCTTCATCATTTTTGGTTGCTCACGTTCCAATTACTGCTAATGCCAAGGCAATCACTGAAGCCATTAATTTGATTAAAAAGCAAGATAAAACCAAATCAATTTATTTATTGACTGGTGAAACCGATAAAGTTGCTCATGGATGTTATGTTAGTGATGAAGCCATTGCCAAGGGTATTAATGCGAAAATCAAACCTTTTGAAGCACGAGCAATTAACTGGTCCACGGATCTTAATGCTGAGGTACATATTGAGCATTATATAAATATATTCAATTATGCACGATCATCTTGGGAGCCATTGGTTGAAAGTTGGCCAATAGCAGTTTACATGTCAAAATCCCGACACCCAAAGCCTCAATTATTAGTAGAGGTGATTTCTAGACAGGTAGCTCAAGTGACGCTTACATCCAAAGCAGTAGCATTGCTATCTCAAGTATCCGATTTGATTACTTCCRGAGAAAAATTAAAACCAAGAGGTGAAGATTACCCATACGTKATAGTGAATGAGACTGGTTTAGATTTGGAAGTTTGGAATGATGCAAAWGAATYCGAAACCAATGGAGTTCCAATTGTTTTACGTGCTGGTAAAGCTTTAGATGAAAGTAAAGTTGAAATTAGAATTCAATTTAAACCAGTCGCCAAGGGGATGTTTAAAGAAATTCAAAGAAATGAATTAGTTATTAGAGTACAACCAAATGAAGCCATTTATTTAAAAATTAATTCCAAAATCCCTGGAATTTCTACTGAAACTTCATTAACTGATTTAGATTTAACTTATGCTACTCGTTATTCTAAAGAYTTTTGGATTCCTGAAGCTTATGAAGCATTAATTAGAGATTGYTATTTAGGTAATCATTCTAATTTTGTTAGAGATGATGAATTGGATGTTTCTTGGAAATTATTTACTCCATTATTGAAT

>hp38bt

AATTATTGAARTGGAATGATATTCCATTGGCTCCACCAGACAARATTTTGGGTATTTCTGAAGCTTATAACAATGATTCTAACCCTCAAAAARTCAATTTGGGGGTTGGTGCTTATAGAGATAATTCTGGTAAACCAATTATTTTCCCATCAGTTAAAAAAGCTGAAGAAATTTTATTGGGTAAAGAAACTGAAAAGGAATATACTGCCATTGTTGGTTCCAAAAATTTCCAATCAATTGTGAAAAATTTCATTTTCAACAATTCTAATAAAGATGCCAATGGTAAACAATTAATTGATGATGGTAGAATTGTTACTGCTCAAACCATCTCTGGTACTGGATCACTTAAACCTCTTATGGTGAAATTTTTGCTAAACATAGAGAACCAAATTTGGAAATTATTCGTGAGGTTGTTGATTCCAAACATATTGTTTTTGATGTGTTGGCACAATTCTTAATCAATCCAGACCCATGGGTTGCCATTGCTGCCGCTGAAGTTTATGTCAGACGTTCATACCGTGCTTATGATTTGGGTAMAATTGAATATCATGTTAATGACAGACTTCCTATTGTTGAATGGAAATTCAAGTTGGCTAATATGGGAGCCGCTGGTGTAAACGATGCTCAACAGGCTGCTGCTGCYGGTGGCGATGATTCGACATCTATGAAACATGCAGCTTCTGTGTCTGATTTGACCTTTGTTGTTGATTATTCATRAAAGCGAAATCCCAGGATACACTCTCCCCGATAATCCAAAGTTCACCCTTGGTAATTTGTTTGTAATAATTGGAGTCTTGTTGGTTTGTATTTTAGCTGTTGTCTCTCTTTTGAGAAATATTAGTGAGTCGGCCTTGTTCAAGAAGAATGGGTATGAACCGTTGGATTCGGATCCTAGTGTCATGAACCTAAACTTCGAGCCTACAACATTGTCCTTTGAAGATATTAAATATGAGGTTACTGGTGGTCGACAAATTTTAAATGGAGTCTTTGGGTTTGTAAAACCAAGAGAATGTTTGGCTATAATGGGAGGTTCAGGTGCTGGTAAAACTACATTGTTGGATTTTAAACCTTTGGACCAATTGGCTAARACTTTGRCYACTGTTCCTGAATTGAATGAAATTATTGGKCAAGAWTTAGTTGACGAATTTRTCAGTGGTATTAAACTACCAGCAGAAGTTGGAAGTCAAGATGATGTTAACAATAGAAAATTGTTGCAAAAAGTGTTTGGTAAATTAATGAACACTGATGATGACGTTATAAAACAACAAACAGCTAAATTACTTGAAAGAACAGACAGAGAACCTCAAGTGTTCAAGGATATTGATTCTAGATTACCGGAGTTAATACAAAGATTAAACAAACAATTTCCTAATGACATCGGATTATTTTGTGGATGTCTCTTATTGAACCACGTTGATTGAAAAAATTATCAATTTCCGTGTTGGATAAACAGAGATTGACTGAAAAATTCAATAAATTGGATAAATCCATCAAAGATAATTTGAAGGCTAAACAAAAAGAAGAAACCAAAAAAACTTTAGATGTGGTTAATAATTGGTTGAATGATAAAGAAAATGCTTCATCATTTTTGGTTGCTCACGTTCCAATTACTGCTAATGCCAAGGCAATCACTGAAGCCATTAATTTGATTAAAAAGCAAGATAAAACCAAATCAATTTATTTATTGACTGGTGAAACCGATAAAGTTGCTCATGGATGTTATGTTAGTGATGAAGCCATTGCCAAGGGTATTAATGCGAAAATCAAACCTTTTGAAGCACGAGCAATTAACTGGTCCACGGATCTTAATGCTGAGGTACATATTGAGCATTATATAAATATATTCAATTATGCACGATCATCTTGGGAGCCATTGGTTGAAAGTTGGCCAATAGCAGTTTACATGTCAAAATCCCGACACCCAAAGCCTCAATTATTAGTAGAGGTGATTTCTAGACAGGTAGCYCAAGTGACGCTTACATCCAAAGCAGTAGCATTGCTATCTCAAGTATCCGATTTGATTACTTCCRGAGAAAAATTAAAACCAAGAGGTGAAGATTACCCATACGTKATAGTGAATGAGACTGGTTTAGATTTGGAAGTTTGGAATGATGCAAAWGAATYCGAAACCAATGGAGTTCCAATTGTTTTACGTGCTGGTAAAGCTTTAGATGAAAGTAAAGTTGAAATTAGAATTCAATTTAAACCAGTCGCCAAGGGGATGTTTAAAGAAATTCAAAGAAATGAATTAGTTATTAGAGTACAACCAAATGAAGCCATTTATTTAAAAATTAATTCCAAAATCCCTGGAATTTCTACTGAAACTTCATTAACTGATTTAGATTTAACTTATGCTACTCGTTATTCTAAAGACTTTTGGATTCCTGAAGCTTATGAAGCATTAATTAGAGATTGCTATTTAGGTAATCATTCTAATTTTGTTAGAGATGATGAATTGGATGTTTCTTGGAAATTATTTACTCCATTATTGAAT

>hp41bt

AATTATTGAAATGGAATGATATTCCATTGGCTCCACCAGACAAAATTTTGGGTATTTCTGAAGCTTATAACAATGATTCTAACCCTCAAAAAATCAATTTGGGGGTTGGTGCTTATAGAGATAATTCCGGTAAACCAATTATTTTCCCATCAGTTAAAAAAGCTGAAGAAATTTTATTGGGTAAAGAAACTGAAAAGGAATATACTGCCATTGTTGGTTCCAAAAATTTCCAATCAATTGTGAAAAATTTCATTTTCAACAATTCTAATAAAGATGCCAATGGTAAACAATTAATTGATGATGGTAGAATTGTTACTGCTCAAACCATCTCTGGTACTGGATCACTTAAACCTCTTATGGTGAAATTTTTGCTAAACATAGAGAACCAAATTTGGAAATTATTCGTGAGGTTGTTGATTCCAAACATATTGTTTTTGATGTGTTGGCACAATTCTTAATCAATCCAGACCCATGGGTTGCCATTGCTGCCGCTGAAGTTTATGTCAGACGTTCATACCGTGCTTATGATTTGGGTAAAATTGAATATCATGTTAATGACAGACTTCCTATTGTTGAATGGAAATTCAAGTTGGCTAATATGGGAGCTGCTGGTGTAAACGATGCTCAACAGGCTGCTGCTGCCGGTGGCGATGATTCGACATCTATGAAACATGCAGCTTCTGTGTCTGATTTGACCTTTGTTGTTGATTATTCATAAAAGYGAAATYCCAGGATACACTCTCCCCGATAATCCAAAGTTCACCCTTGGTAATTTGTTTGTAATAATTGGRGTCTTGTTGGTTTGTGTTTTAGCTGTTGTCTCTCTTTTGAGAAATATTAGTGAGTCRGCCTTGTTCAAGAAGAATGGGTATGAACCGTTGGATTCRGATCCTAGTGTCATGAACCWAAACTTYGAGCCTACAACATTGTCCTTTGAAGATATTAAATATGAGGTTACTGGTGGTCGACAAATTTTAAATGGAGTCTTTGGGTTTGTAAAACCAAGAGAATGTTTGGCTATAATGGGAGGTTCAGGTGCTGGTAAAACTACATTGTTGGATTTTAAACCTTTGGACCAATTGGCTAAGACTTTGACCACTGTTCCTGAATTGAATGAAATTATTGGGCAAGATTTAGTTGACGAATTTGTCAGTGGTATTAAACTACCAGCAGAAGTTGGAAGTCAAGATGATGTTAACAATAGAAAATTGTTGCAAAAAGTGTTTGGTAAATTAATGAACACTGATGATGACGTTATAAAACAACAAACAGCTAAATTACTTGAAAGAACAGAMAGRGAACCTCAAGTGTTCAAGGATATTGATTCTAGATTACCRGAGTTAATACAARGATTAAACAAACAATTTCCTAATGACATCGGATTATTTTGTGGATGTCTCTTATTGAACCACGTTGMTTGAAAAAATTATCAATTTCCGTGTTGGATAAACARAGATTGACTGAAAAATTCAATAAATTGGATAAATCCATYAAAGATAATTTGAAGGCTAAACAAAAAGAAGAAACCAAAAARACTTTAGATGTGGTTAAYAATTGGTTGAATGATAAAGAAAATGCTTCATCATTTTTGGTTGCTCACGTTCCAATTACTGCTAATGCCAAGGCAATCACTGAAGCCATTAATTTGATTAAAAAGCAAGATAAAACCAAATCAATTTATTTATTGACTGGTGAAACCGATAAAGTTGCTCATGGATGTTATGTTAGTGATGAAGCCATTGYCAAGGGTATTAATGCGAAAATCAAACCTTTTGAAGCACGAGCAATTAACTGGTCAACGGATCTTAATGCTGAGGTACATATTGAGCATTATATAAATATATTCAATTATGCACGATCATCTTGGGAGCCATTGGTTGAAAGTTGGCCAATAGCAGTTTACATGTCAAAATCCCGACACCCAAAGCCTCAATTATTAGTAGAGGTGATTTCTAGACAGGTAGCTCAAGTGACGCTTACATCCAAAGCAGTAGCATTGCTATCTCAAGTATCCGATTTGATTACTTCCGGAGAAAAATTAAAACCAAGAGGTGAAGATTACCCATACATTATAGTGAATGAGACTGGTTTAGATTTGGAAGTTTGGAATGATGCAAAAGAATCCGAAACCAATGGAGTTCCAATTGTTTTACGTGCTGGTAAAGCTTTAGATGAAAGTAAAGTTGAAATTAGAATTCAATTTAAACCAGTCGCCAAGGGGATGTTTAAAGAAATTCAAAGAAATGAATTAGTTATTAGAGTACAACCAAATGAAGCCATTTATTTAAAAATTAATTCCAAAATCCCTGGAATTTCTACTGAAACTTCATTAACTGATTTAGATTTAACTTATGCTACTCGTTATTCTAAAGAYTTTTGGATTCCTGAAGCTTATGAAGCATTAATTAGAGATTGTTATTTAGGTAATCATTCTAATTTTGTTAGAGATGATGAATTGGATGTTTCTTGGAAATTATTTACTCCATTATTGAAT

>hp41vu

AATTATTGAAATGGAATGATATTCCATTGGCTCCACCAGACAAAATTTTGGGTATTTCTGAAGCTTATAACAATGATTCTAACCCTCAAAAAATCAATTTGGGGGTTGGTGCTTATAGAGATAATTCCGGTAAACCAATTATTTTCCCATCAGTTAAAAAAGCTGAAGAAATTTTATTGGGTAAAGAAACTGAAAAGGAATATACTGCCATTGTTGGTTCCAAAAATTTCCAATCAATTGTGAAAAATTTCATTTTCAACAATTCTAATAAAGATGCCAATGGTAAACAATTAATTGATGATGGTAGAATTGTTACTGCTCAAACCATCTCTGGTACTGGATCACTTAAACCTCTTATGGTGAAATTTTTGCTAAACATAGAGAACCAAATTTGGAAATTATTCGTGAGGTTGTTGATTCCAAACATATTGTTTTTGATGTGTTGGCACAATTCTTAATCAATCCAGACCCATGGGTTGCCATTGCTGCCGCTGAAGTTTATGTCAGACGTTCATACCGTGCTTATGATTTGGGTAAAATTGAATATCATGTTAATGACAGACTTCCTATTGTTGAATGGAAATTCAAGTTGGCTAATATGGGAGCTGCTGGTGTAAACGATGCTCAACAGGCTGCTGCTGCCGGTGGCGATGATTCGACATCTATGAAACATGCAGCTTCTGTGTCTGATTTGACCTTTGTTGTTGATTATTCATAAAAGYGAAATYCCAGGATACACTCTCCCCGATAATCCAAAGTTCACCCTTGGTAATTTGTTTGTAATAATTGGRGTCTTGTTGGTTTGTGTTTTAGCTGTTGTCTCTCTTTTGAGAAATATTAGTGAGTCRGCCTTGTTCAAGAAGAATGGGTATGAACCGTTGGATTCRGATCCTAGTGTCATGAACCWAAACTTYGAGCCTACAACATTGTCCTTTGAAGATATTAAATATGAGGTTACTGGTGGTCGACAAATTTTAAATGGAGTCTTTGGGTTTGTAAAACCAAGAGAATGTTTGGCTATAATGGGAGGTTCAGGTGCTGGTAAAACTACATTGTTGGATTTTAAACCTTTGGACCAATTGGCTAAGACTTTGACCACTGTTCCTGAATTGAATGAAATTATTGGGCAAGATTTAGTTGACGAATTTGTCAGTGGTATTAAACTACCAGCAGAAGTTGGAAGTCAAGATGATGTTAACAATAGAAAATTGTTGCAAAAAGTGTTTGGTAAATTAATGAACACTGATGATGACGTTATAAAACAACAAACAGCTAAATTACTTGAAAGAACAGAMAGRGAACCTCAAGTGTTCAAGGATATTGATTCTAGATTACCRGAGTTAATACAARGATTAAACAAACAATTTCCTAATGACATCGGATTATTTTGTGGATGTCTCTTATTGAACCACGTTGMTTGAAAAAATTATCAATTTCCGTGTTGGATAAACARAGATTGACTGAAAAATTCAATAAATTGGATAAATCCATYAAAGATAATTTGAAGGCTAAACAAAAAGAAGAAACCAAAAARACTTTAGATGTGGTTAAYAATTGGTTGAATGATAAAGAAAATGCTTCATCATTTTTGGTTGCTCACGTTCCAATTACTGCTAATGCCAAGGCAATCACTGAAGCCATTAATTTGATTAAAAAGCAAGATAAAACCAAATCAATTTATTTATTGACTGGTGAAACCGATAAAGTTGCTCATGGATGTTATGTTAGTGATGAAGCCATTGYCAAGGGTATTAATGCGAAAATCAAACCTTTTGAAGCACGAGCAATTAACTGGTCAACGGATCTTAATGCTGAGGTACATATTGAGCATTATATAAATATATTCAATTATGCACGATCATCTTGGGAGCCATTGGTTGAAAGTTGGCCAATAGCAGTTTACATGTCAAAATCCCGACACCCAAAGCCTCAATTATTAGTAGAGGTGATTTCTAGACAGGTAGCTCAAGTGACGCTTACATCCAAAGCAGTAGCATTGCTATCTCAAGTATCCGATTTGATTACTTCCGGAGAAAAATTAAAACCAAGAGGTGAAGATTACCCATACATTATAGTGAATGAGACTGGTTTAGATTTGGAAGTTTGGAATGATGCAAAAGAATCCGAAACCAATGGAGTTCCAATTGTTTTACGTGCTGGTAAAGCTTTAGATGAAAGTAAAGTTGAAATTAGAATTCAATTTAAACCAGTCGCCAAGGGGATGTTTAAAGAAATTCAAAGAAATGAATTAGTTATTAGAGTACAACCAAATGAAGCCATTTATTTAAAAATTAATTCCAAAATCCCTGGAATTTCTACTGAAACTTCATTAACTGATTTAGATTTAACTTATGCTACTCGTTATTCTAAAGAYTTTTGGATTCCTGAAGCTTATGAAGCATTAATTAGAGATTGTTATTTAGGTAATCATTCTAATTTTGTTAGAGATGATGAATTGGATGTTTCTTGGAAATTATTTACTCCATTATTGAAT

>hp50an

AATTATTGAAATGGAATGATATTCCATTGGCTCCACCAGACAAAATTTTGGGTATTTCTGAAGCTTATAACAACGATTCTAACCCTCAAAAAATCAATTTGGGGGTTGGTGCTTATAGAGATAATTCCGGTAAACCAATTATTTTCCCATCAGTTAAAAAAGCTGAAGAAATTTTATTGGGTAAAGAAACTGAAAAGGAATATACTGCCATTGTTGGTTCCAAAAATTTCCAATCAATTGTGAAAAATTTCATTTTCAACAATTCTAATAAAGATGCCAATGGTAAACAATTAATTGATGATGGTAGAATTGTTACTGCTCAAACCATCTCTGGTACTGGATCACTTAAACCTCTTATGGTGAAATTTTTGCTAAACATAGAGAACCAAATTTGGAAATTATTCGTGAGGTTGTTGATTCCAAACATATTGTTTTTGATGTGTTGGCACAATTCTTAATCAATCCAGACCCATGGGTTGCCATTGCTGCCGCTGAAGTTTATGTCAGACGTTCATACCGTGCTTATGATTTGGGTACAATTGAATATCATGTTAATGACAGACTTCCTATTGTTGAATGGAAATTCAAGTTGGCTAATATGGGAGCCGCTGGTGTAAACGATGCTCAACAGGCTGCTGCTGCTGGTGGCGATGATTCGACATCTATGAAACATGCAGCTTCTGTGTCTGATTTGACCTTTGTTGTTGATTATTCATAAAAGCGAAATCCCAGGATACACTCTCCCCGATAATCCAAAGTTCACCCTTGGTAATTTGTTTGTAATAATTGGAGTCTTGTTGGTTTGTGTTTTAGCTGTTGTCTCTCTTTTGAGAAATATTAGTGAGTCGGCCTTGTTCAAGAAGAATGGGTATGAACCGTTGGATTCGGATCCTAGTGTCATGAACCTAAACTTCGAGCCTACAACATTGTCCTTTGAAGATATTAAATATGAGGTTACTGGTGGTCGACAAATTTTAAATGGAGTCTTTGGGTTTGTAAAACCAAGAGAATGTTTGGCTATAATGGGAGGTTCAGGTGCTGGTAAAACTACATTGTTGGATTTTAAACCTTTGGACCAATTGGCTAAGACTTTGACCACTGTTCCTGAATTGAATGAAATTATTGGGCAAGATTTAGTTGACGAATTTGTCAGTGGTATTAAACTACCAGCAGAAGTTGGAAGTCAAGATGATGTTAACAATAGAAAATTGTTGCAAAAAGTGTTTGGTAAATTAATGAACACTGATGATGACGTTATAAAACAACAAACAGCTAAATTACTTGAAAGAACAGACAGAGAACCTCAAGTGTTCAAGGATATTGATTCTAGATTACCGGAGTTAATACAAAGATTAAACAAACAATTTCCTAATGACATCGGATTATTTTGTGGATGTCTCTTATTGAACCACGTTGMTTGAAAAAATTATCAATTTCCGTGTTGGATAAACAAAGATTGACTGAAAAATTCAATAAATTGGATAAATCCATYAAAGATAATTTGAAGGCTAAACAAAAAGAAGAAACCAAAAARACTTTAGATGTGGTTAAYAATTGGTTGAATGATAAAGAAAATKCTTCATCATTTTTGGTTGCTCACGTTCCAATTACTGCTAATGCCAAGGCAATCACTGAAGCCATTAATTTGATTAAAAAGCAAGATAAAACCAAATCAATTTATTTATTGACTGGTGAAACCGATAAAGTTGCTCATGGATGTTATGTTAGTGATGAAGCCATTGTCAAGGGTATTAATGCGAAAATCAAACCTTTTGAAGCACGAGCAATTAACTGGTCCACGGATCTTAATGCTGAGGTACATATTGAGCATTATATAAATATATTCAATTATGCACGATCATCTTGGGAGCCATTGGTTGAAAGTTGGCCAATAGCAGTTTACATGTCAAAATCCCGACACCCAAAGCCTCAATTATTAGTAGAGGTGATTTCTAGACAGGTAGCTCAAGTGACGCTTACATCCAAAGCGGTAGCATTGCTATCTCAAGTATCCGATTTGATTACTTCCGGAGAAAAATTAAAACCAAGAGGTGAAGATTACCCATACGTTATAGTGAATGAGACTGGTTTAGATTTGGAAGTTTGGAATGATGCAAATGAATCCGAAACCAATGGAGTTCCAATTGTTTTACGTGCTGGTAAAGCTTTAGATGAAAGTAAAGTTGAAATTAGAATTCAATTTAAACCAGTCGCCAAGGGGATGTTTAAAGAAATTCAAAGAAATGAATTAGTTATTAGAGTACAACCAAATGAAGCCATTTATTTAAAAATTAATTCCAAAATCCCTGGAATTTCTACTGAAACTTCATTAACTGATTTAGATTTAACTTATGCTACTCGTTATTCTAAAGACTTTTGGATTCCTGAAGCTTATGAAGCATTAATTAGAGATTGYTATTTAGGTAATCATTCTAATTTTGTTAGAGATGATGAATTGGATGTTTCTTGGAAATTATTTACTCCATTATTGAAT

>hp50ch

AATTATTGAAATGGAATGATATTCCATTGGCTCCACCAGACAAAATTTTGGGTATTTCTGAAGCTTATAACAACGATTCTAACCCTCAAAAAATCAATTTGGGGGTTGGTGCTTATAGAGATAATTCCGGTAAACCAATTATTTTCCCATCAGTTAAAAAAGCTGAAGAAATTTTATTGGGTAAAGAAACTGAAAAGGAATATACTGCCATTGTTGGTTCCAAAAATTTCCAATCAATTGTGAAAAATTTCATTTTCAACAATTCTAATAAAGATGCCAATGGTAAACAATTAATTGATGATGGTAGAATTGTTACTGCTCAAACCATCTCTGGTACTGGATCACTTAAACCTCTTATGGTGAAATTTTTGCTAAACATAGAGAACCAAATTTGGAAATTATTCGTGAGGTTGTTGATTCCAAACATATTGTTTTTGATGTGTTGGCACAATTCTTAATCAATCCAGACCCATGGGTTGCCATTGCTGCCGCTGAAGTTTATGTCAGACGTTCATACCGTGCTTATGATTTGGGTACAATTGAATATCATGTTAATGACAGACTTCCTATTGTTGAATGGAAATTCAAGTTGGCTAATATGGGAGCCGCTGGTGTAAACGATGCTCAACAGGCTGCTGCTGCTGGTGGCGATGATTCGACATCTATGAAACATGCAGCTTCTGTGTCTGATTTGACCTTTGTTGTTGATTATTCATAAAAGCGAAATCCCAGGATACACTCTCCCCGATAATCCAAAGTTCACCCTTGGTAATTTGTTTGTAATAATTGGAGTCTTGTTGGTTTGTGTTTTAGCTGTTGTCTCTCTTTTGAGAAATATTAGTGAGTCGGCCTTGTTCAAGAAGAATGGGTATGAACCGTTGGATTCGGATCCTAGTGTCATGAACCTAAACTTCGAGCCTACAACATTGTCCTTTGAAGATATTAAATATGAGGTTACTGGTGGTCGACAAATTTTAAATGGAGTCTTTGGGTTTGTAAAACCAAGAGAATGTTTGGCTATAATGGGAGGTTCAGGTGCTGGTAAAACTACATTGTTGGATTTTAAACCTTTGGACCAATTGGCTAAGACTTTGACCACTGTTCCTGAATTGAATGAAATTATTGGGCAAGATTTAGTTGACGAATTTGTCAGTGGTATTAAACTACCAGCAGAAGTTGGAAGTCAAGATGATGTTAACAATAGAAAATTGTTGCAAAAAGTGTTTGGTAAATTAATGAACACTGATGATGACGTTATAAAACAACAAACAGCTAAATTACTTGAAAGAACAGACAGAGAACCTCAAGTGTTCAAGGATATTGATTCTAGATTACCGGAGTTAATACAAAGATTAAACAAACAATTTCCTAATGACATCGGATTATTTTGTGGATGTCTCTTATTGAACCACGTTGMTTGAAAAAATTATCAATTTCCGTGTTGGATAAACAAAGATTGACTGAAAAATTCAATAAATTGGATAAATCCATYAAAGATAATTTGAAGGCTAAACAAAAAGAAGAAACCAAAAARACTTTAGATGTGGTTAAYAATTGGTTGAATGATAAAGAAAATKCTTCATCATTTTTGGTTGCTCACGTTCCAATTACTGCTAATGCCAAGGCAATCACTGAAGCCATTAATTTGATTAAAAAGCAAGATAAAACCAAATCAATTTATTTATTGACTGGTGAAACCGATAAAGTTGCTCATGGATGTTATGTTAGTGATGAAGCCATTGTCAAGGGTATTAATGCGAAAATCAAACCTTTTGAAGCACGAGCAATTAACTGGTCCACGGATCTTAATGCTGAGGTACATATTGAGCATTATATAAATATATTCAATTATGCACGATCATCTTGGGAGCCATTGGTTGAAAGTTGGCCAATAGCAGTTTACATGTCAAAATCCCGACACCCAAAGCCTCAATTATTAGTAGAGGTGATTTCTAGACAGGTAGCTCAAGTGACGCTTACATCCAAAGCGGTAGCATTGCTATCTCAAGTATCCGATTTGATTACTTCCGGAGAAAAATTAAAACCAAGAGGTGAAGATTACCCATACGTTATAGTGAATGAGACTGGTTTAGATTTGGAAGTTTGGAATGATGCAAATGAATCCGAAACCAATGGAGTTCCAATTGTTTTACGTGCTGGTAAAGCTTTAGATGAAAGTAAAGTTGAAATTAGAATTCAATTTAAACCAGTCGCCAAGGGGATGTTTAAAGAAATTCAAAGAAATGAATTAGTTATTAGAGTACAACCAAATGAAGCCATTTATTTAAAAATTAATTCCAAAATCCCTGGAATTTCTACTGAAACTTCATTAACTGATTTAGATTTAACTTATGCTACTCGTTATTCTAAAGACTTTTGGATTCCTGAAGCTTATGAAGCATTAATTAGAGATTGYTATTTAGGTAATCATTCTAATTTTGTTAGAGATGATGAATTGGATGTTTCTTGGAAATTATTTACTCCATTATTGAAT

>HUN91

AATTATTGAAATGGAATGATATTCCATTGGCYCCACCAGACAARATTTTGGGTATTTCTGAAGCTTATAACAATGATTCTAACCCTCAAAAAATCAATTTGGGGGTTGGTGCTTATAGAGATAATTCYGGTAAACCAATTATTTTCCCATCAGTTAAAAAAGCTGAAGAAATTTTATTGGGTAAAGAAACTGAAAAGGAATATACTGCCATTGTTGGTTCCAAAAATTTCCAATCAATTGTGAAAAATTTCATTTTCAACAATTCTAATAAAGATGCCAATGGTAAACAATTAATTGATGATGGTAGAATTGTTACTGCTCAAACCATCTCTGGTACTGGATCACTTAAACCTCTTATGGTGAAATTTTTGCTAAACATAGAGAACCAAATTTGGAAATTATTCGTGAGGTTGTTGATTCCAAACATATTGTTTTTGATGTGTTGGCACAATTCTTAATCAATCCAGACCCATGGGTTGCCATTGCTGCCGCTGAAGTTTATGTCAGACGTTCATACCGTGCTTATGATTTGGGTAAAATTGAATATCATGTTAATGACAGACTTCCTATTGTTGAATGGAAATTCAAGTTGGCTAATATGGGAGCTGCTGGTGTAAACGATGCTCAACAGGCTGCTGCTGCCGGTGGCGATGATTCGACATCTATGAAACATGCAGCTTCTGTGTCTGATTTGACCTTTGTTGTTGATTATTCATAAAAGCGAAATCCCAGGATACACTCTCCCCGATAATCCAAAGTTCACCCTTGGTAATTTGTTTGTAATAATTGGAGTCTTGTTGGTTTGTATTTTAGCTGTTGTCTCTCTTTTGAGAAATATTAGTGAGTCGGCCTTGTTCAAGAAGAATGGGTATGAACCGTTGGATTCGGATCCTAGTGTCATGAACCTAAACTTCGAGCCTACAACATTGTCCTTTGAAGATATTAAATATGAGGTTACTGGTGGTCGACAAATTTTAAATGGAGTCTTTGGGTTTGTAAAACCAAGAGAATGTTTGGCTATAATGGGAGGTTCAGGTGCTGGTAAAACTACATTGTTGGATTTTAAACCTTTGGACCAATTGGCTAAGACTTTGACCACTGTTCCTGAATTGAATGAAATTATTGGGCAAGATTTAGTTGACGAATTTGTCAGTGGTATTAAACTACCAGCAGAAGTTGGAAGTCAAGATGATGTTAACAATAGAAAATTGTTGCAAAAAGTGTTTGGTAAATTAATGAACACTGATGATGACGTTATAAAACAACAAACAGCTAAATTACTTGAAAGAACAGACAGAGAACCTCAAGTGTTCAAGGATATTGATTCTAGATTACCGGAGTTAATACAAAGATTAAACAAACAATTTCCTAATGACATCGGATTATTTTGTGGATGTCTCTTATTGAACCACGTTGMTTGAAAAAATTATCAATTTCCGTGTTGGATAAACAAAGATTGACTGAAAAATTCAATAAATTGGATAAATCCATTAAAGATAATTTGAAGGCTAAACAAAAAGAAGAAACCAAAAAGACTTTAGATGTGGTTAACAATTGGTTGAATGATAAAGAAAATGCTTCATCATTTTTGGTTGCTCACGTTCCAATTACTGCTAATGCCAAGGCAATCACTGAAGCCATTAATTTGATTAAAAAGCAAGATAAAACCAAATCAATTTATTTATTGACTGGTGAAACMGATAAAGTTGCTCATGGATGTTATGTTAGTGATGAAGCCATTGYYAAGGGTATTAATGCGAAAATCAAACCTTTTGAAGCACGAGCAATTAACTGGTCCACGGATCTTAATGCTGAGGTACATATTGAGCATTATATAAATATATTCAATTATGCACGATCATCTTGGGAGCCATTGGTTGAAAGTTGGCCAATAGCAGTTTACATGTCAAAATCCCGACACCCAAAGCCTCAATTATTAGTAGAGGTGATTTCTAGACAGGTAGCTCAAGTGACGCTTACATCCAAAGCAGTAGCATTGCTATCTCAAGTATCCGATTTGATTACTTCCAGAGAAAAATTAAAACCAAGAGGTGAAGATTACCCATACGTTATAATGAATGAGACTGGTTTAGATTTGGAAGTTTGGAATGATGCAAATGAATCCGAAACCAATGGAGTTCCAATTGTTTTACGTGCTGGTAAAGCTTTAGATGAAAGTAAAGTTGAAATTAGAATTCAATTTAAACCAGTCGCCAAGGGGATGTTTAAAGAAATTCAAAGAAATGAATTAGTTATTAGAGTACAACCAAATGAAGCCATTTATTTAAAAATTAATTCCAAAATCCCTGGAATTTCTACTGAAACTTCATTAACTGATTTAGATTTAACTTATGCTACTCGTTATTCTAAAGATTTTTGGATTCCTGAAGCTTATGAAGCATTAATTAGAGATTGTTATTTAGGTAATCATTCTAATTTTGTTAGAGATGATGAATTGGATGTTTCTTGGAAATTATTTACTCCATTATTGAAT

>HUN91:IND:39:4.2

AATTATTGAAATGGAATGATATTCCATTGGCYCCACCAGACAARATTTTGGGTATTTCTGAAGCTTATAACAATGATTCTAACCCTCAAAAAATCAATTTGGGGGTTGGTGCTTATAGAGATAATTCYGGTAAACCAATTATTTTCCCATCAGTTAAAAAAGCTGAAGAAATTTTATTGGGTAAAGAAACTGAAAAGGAATATACTGCCATTGTTGGTTCCAAAAATTTCCAATCAATTGTGAAAAATTTCATTTTCAACAATTCTAATAAAGATGCCAATGGTAAACAATTAATTGATGATGGTAGAATTGTTACTGCTCAAACCATCTCTGGTACTGGATCACTTAAACCTCTTATGGTGAAATTTTTGCTAAACATAGAGAACCAAATTTGGAAATTATTCGTGAGGTTGTTGATTCCAAACATATTGTTTTTGATGTGTTGGCACAATTCTTAATCAATCCAGACCCATGGGTTGCCATTGCTGCCGCTGAAGTTTATGTCAGACGTTCATACCGTGCTTATGATTTGGGTAAAATTGAATATCATGTTAATGACAGACTTCCTATTGTTGAATGGAAATTCAAGTTGGCTAATATGGGAGCTGCTGGTGTAAACGATGCTCAACAGGCTGCTGCTGCCGGTGGCGATGATTCGACATCTATGAAACATGCAGCTTCTGTGTCTGATTTGACCTTTGTTGTTGATTATTCATAAAAGCGAAATCCCAGGATACACTCTCCCCGATAATCCAAAGTTCACCCTTGGTAATTTGTTTGTAATAATTGGAGTCTTGTTGGTTTGTATTTTAGCTGTTGTCTCTCTTTTGAGAAATATTAGTGAGTCGGCCTTGTTCAAGAAGAATGGGTATGAACCGTTGGATTCGGATCCTAGTGTCATGAACCTAAACTTCGAGCCTACAACATTGTCCTTTGAAGATATTAAATATGAGGTTACTGGTGGTCGACAAATTTTAAATGGAGTCTTTGGGTTTGTAAAACCAAGAGAATGTTTGGCTATAATGGGAGGTTCAGGTGCTGGTAAAACTACATTGTTGGATTTTAAACCTTTGGACCAATTGGCTAAGACTTTGACCACTGTTCCTGAATTGAATGAAATTATTGGGCAAGATTTAGTTGACGAATTTGTCAGTGGTATTAAACTACCAGCAGAAGTTGGAAGTCAAGATGATGTTAACAATAGAAAATTGTTGCAAAAAGTGTTTGGTAAATTAATGAACACTGATGATGACGTTATAAAACAACAAACAGCTAAATTACTTGAAAGAACAGACAGAGAACCTCAAGTGTTCAAGGATATTGATTCTAGATTACCGGAGTTAATACAAAGATTAAACAAACAATTTCCTAATGACATCGGATTATTTTGTGGATGTCTCTTATTGAACCACGTTGMTTGAAAAAATTATCAATTTCCGTGTTGGATAAACAAAGATTGACTGAAAAATTCAATAAATTGGATAAATCCATTAAAGATAATTTGAAGGCTAAACAAAAAGAAGAAACCAAAAAGACTTTAGATGTGGTTAACAATTGGTTGAATGATAAAGAAAATGCTTCATCATTTTTGGTTGCTCACGTTCCAATTACTGCTAATGCCAAGGCAATCACTGAAGCCATTAATTTGATTAAAAAGCAAGATAAAACCAAATCAATTTATTTATTGACTGGTGAAACMGATAAAGTTGCTCATGGATGTTATGTTAGTGATGAAGCCATTGYYAAGGGTATTAATGCGAAAATCAAACCTTTTGAAGCACGAGCAATTAACTGGTCCACGGATCTTAATGCTGAGGTACATATTGAGCATTATATAAATATATTCAATTATGCACGATCATCTTGGGAGCCATTGGTTGAAAGTTGGCCAATAGCAGTTTACATGTCAAAATCCCGACACCCAAAGCCTCAATTATTAGTAGAGGTGATTTCTAGACAGGTAGCTCAAGTGACGCTTACATCCAAAGCAGTAGCATTGCTATCTCAAGTATCCGATTTGATTACTTCCAGAGAAAAATTAAAACCAAGAGGTGAAGATTACCCATACGTTATAATGAATGAGACTGGTTTAGATTTGGAAGTTTGGAATGATGCAAATGAATCCGAAACCAATGGAGTTCCAATTGTTTTACGTGCTGGTAAAGCTTTAGATGAAAGTAAAGTTGAAATTAGAATTCAATTTAAACCAGTCGCCAAGGGGATGTTTAAAGAAATTCAAAGAAATGAATTAGTTATTAGAGTACAACCAAATGAAGCCATTTATTTAAAAATTAATTCCAAAATCCCTGGAATTTCTACTGAAACTTCATTAACTGATTTAGATTTAACTTATGCTACTCGTTATTCTAAAGATTTTTGGATTCCTGAAGCTTATGAAGCATTAATTAGAGATTGTTATTTAGGTAATCATTCTAATTTTGTTAGAGATGATGAATTGGATGTTTCTTGGAAATTATTTACTCCATTATTGAAT

>HUN91:IND:46:12.6

AATTATTGAAATGGAATGATATTCCATTGGCYCCACCAGACAARATTTTGGGTATTTCTGAAGCTTATAACAATGATTCTAACCCTCAAAAAATCAATTTGGGGGTTGGTGCTTATAGAGATAATTCYGGTAAACCAATTATTTTCCCATCAGTTAAAAAAGCTGAAGAAATTTTATTGGGTAAAGAAACTGAAAAGGAATATACTGCCATTGTTGGTTCCAAAAATTTCCAATCAATTGTGAAAAATTTCATTTTCAACAATTCTAATAAAGATGCCAATGGTAAACAATTAATTGATGATGGTAGAATTGTTACTGCTCAAACCATCTCTGGTACTGGATCACTTAAACCTCTTATGGTGAAATTTTTGCTAAACATAGAGAACCAAATTTGGAAATTATTCGTGAGGTTGTTGATTCCAAACATATTGTTTTTGATGTGTTGGCACAATTCTTAATCAATCCAGACCCATGGGTTGCCATTGCTGCCGCTGAAGTTTATGTCAGACGTTCATACCGTGCTTATGATTTGGGTAAAATTGAATATCATGTTAATGACAGACTTCCTATTGTTGAATGGAAATTCAAGTTGGCTAATATGGGAGCTGCTGGTGTAAACGATGCTCAACAGGCTGCTGCTGCCGGTGGCGATGATTCGACATCTATGAAACATGCAGCTTCTGTGTCTGATTTGACCTTTGTTGTTGATTATTCATAAAAGCGAAATCCCAGGATACACTCTCCCCGATAATCCAAAGTTCACCCTTGGTAATTTGTTTGTAATAATTGGAGTCTTGTTGGTTTGTATTTTAGCTGTTGTCTCTCTTTTGAGAAATATTAGTGAGTCGGCCTTGTTCAAGAAGAATGGGTATGAACCGTTGGATTCGGATCCTAGTGTCATGAACCTAAACTTCGAGCCTACAACATTGTCCTTTGAAGATATTAAATATGAGGTTACTGGTGGTCGACAAATTTTAAATGGAGTCTTTGGGTTTGTAAAACCAAGAGAATGTTTGGCTATAATGGGAGGTTCAGGTGCTGGTAAAACTACATTGTTGGATTTTAAACCTTTGGACCAATTGGCTAAGACTTTGACCACTGTTCCTGAATTGAATGAAATTATTGGGCAAGATTTAGTTGACGAATTTGTCAGTGGTATTAAACTACCAGCAGAAGTTGGAAGTCAAGATGATGTTAACAATAGAAAATTGTTGCAAAAAGTGTTTGGTAAATTAATGAACACTGATGATGACGTTATAAAACAACAAACAGCTAAATTACTTGAAAGAACAGACAGAGAACCTCAAGTGTTCAAGGATATTGATTCTAGATTACCGGAGTTAATACAAAGATTAAACAAACAATTTCCTAATGACATCGGATTATTTTGTGGATGTCTCTTATTGAACCACGTTGMTTGAAAAAATTATCAATTTCCGTGTTGGATAAACAAAGATTGACTGAAAAATTCAATAAATTGGATAAATCCATTAAAGATAATTTGAAGGCTAAACAAAAAGAAGAAACCAAAAAGACTTTAGATGTGGTTAACAATTGGTTGAATGATAAAGAAAATGCTTCATCATTTTTGGTTGCTCACGTTCCAATTACTGCTAATGCCAAGGCAATCACTGAAGCCATTAATTTGATTAAAAAGCAAGATAAAACCAAATCAATTTATTTATTGACTGGTGAAACMGATAAAGTTGCTCATGGATGTTATGTTAGTGATGAAGCCATTGYYAAGGGTATTAATGCGAAAATCAAACCTTTTGAAGCACGAGCAATTAACTGGTCCACGGATCTTAATGCTGAGGTACATATTGAGCATTATATAAATATATTCAATTATGCACGATCATCTTGGGAGCCATTGGTTGAAAGTTGGCCAATAGCAGTTTACATGTCAAAATCCCGACACCCAAAGCCTCAATTATTAGTAGAGGTGATTTCTAGACAGGTAGCTCAAGTGACGCTTACATCCAAAGCAGTAGCATTGCTATCTCAAGTATCCGATTTGATTACTTCCAGAGAAAAATTAAAACCAAGAGGTGAAGATTACCCATACGTTATAATGAATGAGACTGGTTTAGATTTGGAAGTTTGGAATGATGCAAATGAATCCGAAACCAATGGAGTTCCAATTGTTTTACGTGCTGGTAAAGCTTTAGATGAAAGTAAAGTTGAAATTAGAATTCAATTTAAACCAGTCGCCAAGGGGATGTTTAAAGAAATTCAAAGAAATGAATTAGTTATTAGAGTACAACCAAATGAAGCCATTTATTTAAAAATTAATTCCAAAATCCCTGGAATTTCTACTGAAACTTCATTAACTGATTTAGATTTAACTTATGCTACTCGTTATTCTAAAGATTTTTGGATTCCTGAAGCTTATGAAGCATTAATTAGAGATTGTTATTTAGGTAATCATTCTAATTTTGTTAGAGATGATGAATTGGATGTTTCTTGGAAATTATTTACTCCATTATTGAAT

>HUN91:IND:71:5.9

AATTATTGAAATGGAATGATATTCCATTGGCYCCACCAGACAARATTTTGGGTATTTCTGAAGCTTATAACAATGATTCTAACCCTCAAAAAATCAATTTGGGGGTTGGTGCTTATAGAGATAATTCYGGTAAACCAATTATTTTCCCATCAGTTAAAAAAGCTGAAGAAATTTTATTGGGTAAAGAAACTGAAAAGGAATATACTGCCATTGTTGGTTCCAAAAATTTCCAATCAATTGTGAAAAATTTCATTTTCAACAATTCTAATAAAGATGCCAATGGTAAACAATTAATTGATGATGGTAGAATTGTTACTGCTCAAACCATCTCTGGTACTGGATCACTTAAACCTCTTATGGTGAAATTTTTGCTAAACATAGAGAACCAAATTTGGAAATTATTCGTGAGGTTGTTGATTCCAAACATATTGTTTTTGATGTGTTGGCACAATTCTTAATCAATCCAGACCCATGGGTTGCCATTGCTGCCGCTGAAGTTTATGTCAGACGTTCATACCGTGCTTATGATTTGGGTAAAATTGAATATCATGTTAATGACAGACTTCCTATTGTTGAATGGAAATTCAAGTTGGCTAATATGGGAGCTGCTGGTGTAAACGATGCTCAACAGGCTGCTGCTGCCGGTGGCGATGATTCGACATCTATGAAACATGCAGCTTCTGTGTCTGATTTGACCTTTGTTGTTGATTATTCATAAAAGCGAAATCCCAGGATACACTCTCCCCGATAATCCAAAGTTCACCCTTGGTAATTTGTTTGTAATAATTGGAGTCTTGTTGGTTTGTATTTTAGCTGTTGTCTCTCTTTTGAGAAATATTAGTGAGTCGGCCTTGTTCAAGAAGAATGGGTATGAACCGTTGGATTCGGATCCTAGTGTCATGAACCTAAACTTCGAGCCTACAACATTGTCCTTTGAAGATATTAAATATGAGGTTACTGGTGGTCGACAAATTTTAAATGGAGTCTTTGGGTTTGTAAAACCAAGAGAATGTTTGGCTATAATGGGAGGTTCAGGTGCTGGTAAAACTACATTGTTGGATTTTAAACCTTTGGACCAATTGGCTAAGACTTTGACCACTGTTCCTGAATTGAATGAAATTATTGGGCAAGATTTAGTTGACGAATTTGTCAGTGGTATTAAACTACCAGCAGAAGTTGGAAGTCAAGATGATGTTAACAATAGAAAATTGTTGCAAAAAGTGTTTGGTAAATTAATGAACACTGATGATGACGTTATAAAACAACAAACAGCTAAATTACTTGAAAGAACAGACAGAGAACCTCAAGTGTTCAAGGATATTGATTCTAGATTACCGGAGTTAATACAAAGATTAAACAAACAATTTCCTAATGACATCGGATTATTTTGTGGATGTCTCTTATTGAACCACGTTGMTTGAAAAAATTATCAATTTCCGTGTTGGATAAACAAAGATTGACTGAAAAATTCAATAAATTGGATAAATCCATTAAAGATAATTTGAAGGCTAAACAAAAAGAAGAAACCAAAAAGACTTTAGATGTGGTTAACAATTGGTTGAATGATAAAGAAAATGCTTCATCATTTTTGGTTGCTCACGTTCCAATTACTGCTAATGCCAAGGCAATCACTGAAGCCATTAATTTGATTAAAAAGCAAGATAAAACCAAATCAATTTATTTATTGACTGGTGAAACMGATAAAGTTGCTCATGGATGTTATGTTAGTGATGAAGCCATTGYYAAGGGTATTAATGCGAAAATCAAACCTTTTGAAGCACGAGCAATTAACTGGTCCACGGATCTTAATGCTGAGGTACATATTGAGCATTATATAAATATATTCAATTATGCACGATCATCTTGGGAGCCATTGGTTGAAAGTTGGCCAATAGCAGTTTACATGTCAAAATCCCGACACCCAAAGCCTCAATTATTAGTAGAGGTGATTTCTAGACAGGTAGCTCAAGTGACGCTTACATCCAAAGCAGTAGCATTGCTATCTCAAGTATCCGATTTGATTACTTCCAGAGAAAAATTAAAACCAAGAGGTGAAGATTACCCATACGTTATAATGAATGAGACTGGTTTAGATTTGGAAGTTTGGAATGATGCAAATGAATCCGAAACCAATGGAGTTCCAATTGTTTTACGTGCTGGTAAAGCTTTAGATGAAAGTAAAGTTGAAATTAGAATTCAATTTAAACCAGTCGCCAAGGGGATGTTTAAAGAAATTCAAAGAAATGAATTAGTTATTAGAGTACAACCAAATGAAGCCATTTATTTAAAAATTAATTCCAAAATCCCTGGAATTTCTACTGAAACTTCATTAACTGATTTAGATTTAACTTATGCTACTCGTTATTCTAAAGATTTTTGGATTCCTGAAGCTTATGAAGCATTAATTAGAGATTGTTATTTAGGTAATCATTCTAATTTTGTTAGAGATGATGAATTGGATGTTTCTTGGAAATTATTTACTCCATTATTGAAT

>HUN91:IND:73:8.4

AATTATTGAAATGGAATGATATTCCATTGGCYCCACCAGACAARATTTTGGGTATTTCTGAAGCTTATAACAATGATTCTAACCCTCAAAAAATCAATTTGGGGGTTGGTGCTTATAGAGATAATTCYGGTAAACCAATTATTTTCCCATCAGTTAAAAAAGCTGAAGAAATTTTATTGGGTAAAGAAACTGAAAAGGAATATACTGCCATTGTTGGTTCCAAAAATTTCCAATCAATTGTGAAAAATTTCATTTTCAACAATTCTAATAAAGATGCCAATGGTAAACAATTAATTGATGATGGTAGAATTGTTACTGCTCAAACCATCTCTGGTACTGGATCACTTAAACCTCTTATGGTGAAATTTTTGCTAAACATAGAGAACCAAATTTGGAAATTATTCGTGAGGTTGTTGATTCCAAACATATTGTTTTTGATGTGTTGGCACAATTCTTAATCAATCCAGACCCATGGGTTGCCATTGCTGCCGCTGAAGTTTATGTCAGACGTTCATACCGTGCTTATGATTTGGGTAAAATTGAATATCATGTTAATGACAGACTTCCTATTGTTGAATGGAAATTCAAGTTGGCTAATATGGGAGCTGCTGGTGTAAACGATGCTCAACAGGCTGCTGCTGCCGGTGGCGATGATTCGACATCTATGAAACATGCAGCTTCTGTGTCTGATTTGACCTTTGTTGTTGATTATTCATAAAAGCGAAATCCCAGGATACACTCTCCCCGATAATCCAAAGTTCACCCTTGGTAATTTGTTTGTAATAATTGGAGTCTTGTTGGTTTGTATTTTAGCTGTTGTCTCTCTTTTGAGAAATATTAGTGAGTCGGCCTTGTTCAAGAAGAATGGGTATGAACCGTTGGATTCGGATCCTAGTGTCATGAACCTAAACTTCGAGCCTACAACATTGTCCTTTGAAGATATTAAATATGAGGTTACTGGTGGTCGACAAATTTTAAATGGAGTCTTTGGGTTTGTAAAACCAAGAGAATGTTTGGCTATAATGGGAGGTTCAGGTGCTGGTAAAACTACATTGTTGGATTTTAAACCTTTGGACCAATTGGCTAAGACTTTGACCACTGTTCCTGAATTGAATGAAATTATTGGGCAAGATTTAGTTGACGAATTTGTCAGTGGTATTAAACTACCAGCAGAAGTTGGAAGTCAAGATGATGTTAACAATAGAAAATTGTTGCAAAAAGTGTTTGGTAAATTAATGAACACTGATGATGACGTTATAAAACAACAAACAGCTAAATTACTTGAAAGAACAGACAGAGAACCTCAAGTGTTCAAGGATATTGATTCTAGATTACCGGAGTTAATACAAAGATTAAACAAACAATTTCCTAATGACATCGGATTATTTTGTGGATGTCTCTTATTGAACCACGTTGMTTGAAAAAATTATCAATTTCCGTGTTGGATAAACAAAGATTGACTGAAAAATTCAATAAATTGGATAAATCCATTAAAGATAATTTGAAGGCTAAACAAAAAGAAGAAACCAAAAAGACTTTAGATGTGGTTAACAATTGGTTGAATGATAAAGAAAATGCTTCATCATTTTTGGTTGCTCACGTTCCAATTACTGCTAATGCCAAGGCAATCACTGAAGCCATTAATTTGATTAAAAAGCAAGATAAAACCAAATCAATTTATTTATTGACTGGTGAAACMGATAAAGTTGCTCATGGATGTTATGTTAGTGATGAAGCCATTGYYAAGGGTATTAATGCGAAAATCAAACCTTTTGAAGCACGAGCAATTAACTGGTCCACGGATCTTAATGCTGAGGTACATATTGAGCATTATATAAATATATTCAATTATGCACGATCATCTTGGGAGCCATTGGTTGAAAGTTGGCCAATAGCAGTTTACATGTCAAAATCCCGACACCCAAAGCCTCAATTATTAGTAGAGGTGATTTCTAGACAGGTAGCTCAAGTGACGCTTACATCCAAAGCAGTAGCATTGCTATCTCAAGTATCCGATTTGATTACTTCCAGAGAAAAATTAAAACCAAGAGGTGAAGATTACCCATACGTTATAATGAATGAGACTGGTTTAGATTTGGAAGTTTGGAATGATGCAAATGAATCCGAAACCAATGGAGTTCCAATTGTTTTACGTGCTGGTAAAGCTTTAGATGAAAGTAAAGTTGAAATTAGAATTCAATTTAAACCAGTCGCCAAGGGGATGTTTAAAGAAATTCAAAGAAATGAATTAGTTATTAGAGTACAACCAAATGAAGCCATTTATTTAAAAATTAATTCCAAAATCCCTGGAATTTCTACTGAAACTTCATTAACTGATTTAGATTTAACTTATGCTACTCGTTATTCTAAAGATTTTTGGATTCCTGAAGCTTATGAAGCATTAATTAGAGATTGTTATTTAGGTAATCATTCTAATTTTGTTAGAGATGATGAATTGGATGTTTCTTGGAAATTATTTACTCCATTATTGAAT

>HUN91:IND:84:5.5

AATTATTGAAATGGAATGATATTCCATTGGCYCCACCAGACAARATTTTGGGTATTTCTGAAGCTTATAACAATGATTCTAACCCTCAAAAAATCAATTTGGGGGTTGGTGCTTATAGAGATAATTCYGGTAAACCAATTATTTTCCCATCAGTTAAAAAAGCTGAAGAAATTTTATTGGGTAAAGAAACTGAAAAGGAATATACTGCCATTGTTGGTTCCAAAAATTTCCAATCAATTGTGAAAAATTTCATTTTCAACAATTCTAATAAAGATGCCAATGGTAAACAATTAATTGATGATGGTAGAATTGTTACTGCTCAAACCATCTCTGGTACTGGATCACTTAAACCTCTTATGGTGAAATTTTTGCTAAACATAGAGAACCAAATTTGGAAATTATTCGTGAGGTTGTTGATTCCAAACATATTGTTTTTGATGTGTTGGCACAATTCTTAATCAATCCAGACCCATGGGTTGCCATTGCTGCCGCTGAAGTTTATGTCAGACGTTCATACCGTGCTTATGATTTGGGTAAAATTGAATATCATGTTAATGACAGACTTCCTATTGTTGAATGGAAATTCAAGTTGGCTAATATGGGAGCTGCTGGTGTAAACGATGCTCAACAGGCTGCTGCTGCCGGTGGCGATGATTCGACATCTATGAAACATGCAGCTTCTGTGTCTGATTTGACCTTTGTTGTTGATTATTCATAAAAGCGAAATCCCAGGATACACTCTCCCCGATAATCCAAAGTTCACCCTTGGTAATTTGTTTGTAATAATTGGAGTCTTGTTGGTTTGTATTTTAGCTGTTGTCTCTCTTTTGAGAAATATTAGTGAGTCGGCCTTGTTCAAGAAGAATGGGTATGAACCGTTGGATTCGGATCCTAGTGTCATGAACCTAAACTTCGAGCCTACAACATTGTCCTTTGAAGATATTAAATATGAGGTTACTGGTGGTCGACAAATTTTAAATGGAGTCTTTGGGTTTGTAAAACCAAGAGAATGTTTGGCTATAATGGGAGGTTCAGGTGCTGGTAAAACTACATTGTTGGATTTTAAACCTTTGGACCAATTGGCTAAGACTTTGACCACTGTTCCTGAATTGAATGAAATTATTGGGCAAGATTTAGTTGACGAATTTGTCAGTGGTATTAAACTACCAGCAGAAGTTGGAAGTCAAGATGATGTTAACAATAGAAAATTGTTGCAAAAAGTGTTTGGTAAATTAATGAACACTGATGATGACGTTATAAAACAACAAACAGCTAAATTACTTGAAAGAACAGACAGAGAACCTCAAGTGTTCAAGGATATTGATTCTAGATTACCGGAGTTAATACAAAGATTAAACAAACAATTTCCTAATGACATCGGATTATTTTGTGGATGTCTCTTATTGAACCACGTTGMTTGAAAAAATTATCAATTTCCGTGTTGGATAAACAAAGATTGACTGAAAAATTCAATAAATTGGATAAATCCATTAAAGATAATTTGAAGGCTAAACAAAAAGAAGAAACCAAAAAGACTTTAGATGTGGTTAACAATTGGTTGAATGATAAAGAAAATGCTTCATCATTTTTGGTTGCTCACGTTCCAATTACTGCTAATGCCAAGGCAATCACTGAAGCCATTAATTTGATTAAAAAGCAAGATAAAACCAAATCAATTTATTTATTGACTGGTGAAACMGATAAAGTTGCTCATGGATGTTATGTTAGTGATGAAGCCATTGYYAAGGGTATTAATGCGAAAATCAAACCTTTTGAAGCACGAGCAATTAACTGGTCCACGGATCTTAATGCTGAGGTACATATTGAGCATTATATAAATATATTCAATTATGCACGATCATCTTGGGAGCCATTGGTTGAAAGTTGGCCAATAGCAGTTTACATGTCAAAATCCCGACACCCAAAGCCTCAATTATTAGTAGAGGTGATTTCTAGACAGGTAGCTCAAGTGACGCTTACATCCAAAGCAGTAGCATTGCTATCTCAAGTATCCGATTTGATTACTTCCAGAGAAAAATTAAAACCAAGAGGTGAAGATTACCCATACGTTATAATGAATGAGACTGGTTTAGATTTGGAAGTTTGGAATGATGCAAATGAATCCGAAACCAATGGAGTTCCAATTGTTTTACGTGCTGGTAAAGCTTTAGATGAAAGTAAAGTTGAAATTAGAATTCAATTTAAACCAGTCGCCAAGGGGATGTTTAAAGAAATTCAAAGAAATGAATTAGTTATTAGAGTACAACCAAATGAAGCCATTTATTTAAAAATTAATTCCAAAATCCCTGGAATTTCTACTGAAACTTCATTAACTGATTTAGATTTAACTTATGCTACTCGTTATTCTAAAGATTTTTGGATTCCTGAAGCTTATGAAGCATTAATTAGAGATTGTTATTTAGGTAATCATTCTAATTTTGTTAGAGATGATGAATTGGATGTTTCTTGGAAATTATTTACTCCATTATTGAAT

>HUN91:IND:85:4.0

AATTATTGAAATGGAATGATATTCCATTGGCYCCACCAGACAARATTTTGGGTATTTCTGAAGCTTATAACAATGATTCTAACCCTCAAAAAATCAATTTGGGGGTTGGTGCTTATAGAGATAATTCYGGTAAACCAATTATTTTCCCATCAGTTAAAAAAGCTGAAGAAATTTTATTGGGTAAAGAAACTGAAAAGGAATATACTGCCATTGTTGGTTCCAAAAATTTCCAATCAATTGTGAAAAATTTCATTTTCAACAATTCTAATAAAGATGCCAATGGTAAACAATTAATTGATGATGGTAGAATTGTTACTGCTCAAACCATCTCTGGTACTGGATCACTTAAACCTCTTATGGTGAAATTTTTGCTAAACATAGAGAACCAAATTTGGAAATTATTCGTGAGGTTGTTGATTCCAAACATATTGTTTTTGATGTGTTGGCACAATTCTTAATCAATCCAGACCCATGGGTTGCCATTGCTGCCGCTGAAGTTTATGTCAGACGTTCATACCGTGCTTATGATTTGGGTAAAATTGAATATCATGTTAATGACAGACTTCCTATTGTTGAATGGAAATTCAAGTTGGCTAATATGGGAGCTGCTGGTGTAAACGATGCTCAACAGGCTGCTGCTGCCGGTGGCGATGATTCGACATCTATGAAACATGCAGCTTCTGTGTCTGATTTGACCTTTGTTGTTGATTATTCATAAAAGCGAAATCCCAGGATACACTCTCCCCGATAATCCAAAGTTCACCCTTGGTAATTTGTTTGTAATAATTGGAGTCTTGTTGGTTTGTATTTTAGCTGTTGTCTCTCTTTTGAGAAATATTAGTGAGTCGGCCTTGTTCAAGAAGAATGGGTATGAACCGTTGGATTCGGATCCTAGTGTCATGAACCTAAACTTCGAGCCTACAACATTGTCCTTTGAAGATATTAAATATGAGGTTACTGGTGGTCGACAAATTTTAAATGGAGTCTTTGGGTTTGTAAAACCAAGAGAATGTTTGGCTATAATGGGAGGTTCAGGTGCTGGTAAAACTACATTGTTGGATTTTAAACCTTTGGACCAATTGGCTAAGACTTTGACCACTGTTCCTGAATTGAATGAAATTATTGGGCAAGATTTAGTTGACGAATTTGTCAGTGGTATTAAACTACCAGCAGAAGTTGGAAGTCAAGATGATGTTAACAATAGAAAATTGTTGCAAAAAGTGTTTGGTAAATTAATGAACACTGATGATGACGTTATAAAACAACAAACAGCTAAATTACTTGAAAGAACAGACAGAGAACCTCAAGTGTTCAAGGATATTGATTCTAGATTACCGGAGTTAATACAAAGATTAAACAAACAATTTCCTAATGACATCGGATTATTTTGTGGATGTCTCTTATTGAACCACGTTGMTTGAAAAAATTATCAATTTCCGTGTTGGATAAACAAAGATTGACTGAAAAATTCAATAAATTGGATAAATCCATTAAAGATAATTTGAAGGCTAAACAAAAAGAAGAAACCAAAAAGACTTTAGATGTGGTTAACAATTGGTTGAATGATAAAGAAAATGCTTCATCATTTTTGGTTGCTCACGTTCCAATTACTGCTAATGCCAAGGCAATCACTGAAGCCATTAATTTGATTAAAAAGCAAGATAAAACCAAATCAATTTATTTATTGACTGGTGAAACMGATAAAGTTGCTCATGGATGTTATGTTAGTGATGAAGCCATTGYYAAGGGTATTAATGCGAAAATCAAACCTTTTGAAGCACGAGCAATTAACTGGTCCACGGATCTTAATGCTGAGGTACATATTGAGCATTATATAAATATATTCAATTATGCACGATCATCTTGGGAGCCATTGGTTGAAAGTTGGCCAATAGCAGTTTACATGTCAAAATCCCGACACCCAAAGCCTCAATTATTAGTAGAGGTGATTTCTAGACAGGTAGCTCAAGTGACGCTTACATCCAAAGCAGTAGCATTGCTATCTCAAGTATCCGATTTGATTACTTCCAGAGAAAAATTAAAACCAAGAGGTGAAGATTACCCATACGTTATAATGAATGAGACTGGTTTAGATTTGGAAGTTTGGAATGATGCAAATGAATCCGAAACCAATGGAGTTCCAATTGTTTTACGTGCTGGTAAAGCTTTAGATGAAAGTAAAGTTGAAATTAGAATTCAATTTAAACCAGTCGCCAAGGGGATGTTTAAAGAAATTCAAAGAAATGAATTAGTTATTAGAGTACAACCAAATGAAGCCATTTATTTAAAAATTAATTCCAAAATCCCTGGAATTTCTACTGAAACTTCATTAACTGATTTAGATTTAACTTATGCTACTCGTTATTCTAAAGATTTTTGGATTCCTGAAGCTTATGAAGCATTAATTAGAGATTGTTATTTAGGTAATCATTCTAATTTTGTTAGAGATGATGAATTGGATGTTTCTTGGAAATTATTTACTCCATTATTGAAT

>HUN93

AATTATTGAARTGGAATGATATTCCATTGGCTCCACCAGACAARATTTTGGGTATTTCTGAAGCTTATAACAATGATTCTAACCCTCAAAAARTCAATTTGGGGGTTGGTGCTTATAGAGATAATTCYGGTAAACCAATTATTTTCCCATCAGTTAAAAAAGCTGAAGAAATTTTATTGGGTAAAGAAACTGAAAAGGAATATACTGCCATTGTTGGTTCCAAAAATTTCCAATCAATTGTGAAAAATTTCATTTTCAACAATTCTAATAAAGATGCCAATGGTAAACAATTAATTGATGATGGTAGAATTGTTACTGCTCAAACCATCTCTGGTACTGGATCACTTAAACCTCTTATGGTGAAATTTTTGCTAAACATAGAGAACCAAATTTGGAAATTATTCGTGAGGTTGTTGATTCCAAACATATTGTTTTTGATGTGTTGGCACAATTCTTAATCAATCCAGACCCATGGGTTGCCATTGCTGCCGCTGAAGTTTATGTCAGACGTTCATACCGTGCTTATGATTTGGGTAMAATTGAATATCATGTTAATGACAGACTTCCTATTGTTGAATGGAAATTCAAGTTGGCTAATATGGGAGCCGCTGGTGTAAACGATGCTCAACAGGCTGCTGCTGCYGGTGGCGATGATTCGACATCTATGAAACATGCAGCTTCTGTGTCTGATTTGACCTTTGTTGTTGATTATTCATRAAAGCGAAATCCCAGGATACACTCTCCCCGATAATCCAAAGTTCACCCTTGGTAATTTGTTTGTAATAATTGGAGTCTTGTTGGTTTGTATTTTAGCTGTTGTCTCTCTTTTGAGAAATATTAGTGAGTCGGCCTTGTTCAAGAAGAATGGGTATGAACCGTTGGATTCGGATCCTAGTGTCATGAACCTAAACTTCGAGCCTACAACATTGTCCTTTGAAGATATTAAATATGAGGTTACTGGTGGTCGACAAATTTTAAATGGAGTCTTTGGGTTTGTAAAACCAAGAGAATGTTTGGCTATAATGGGAGGTTCAGGTGCTGGTAAAACTACATTGTTGGATTTTAAACCTTTGGACCAATTGGCTAARACTTTGRCYACTGTTCCTGAATTGAATGAAATTATTGGGCAAGATTTAGTTGACGAATTTGTCAGTGGTATTAAACTACCAGCAGAAGTTGGAAGTCAAGATGATGTTAACAATAGAAAATTGTTGCAAAAAGTGTTTGGTAAATTAATGAACACTGATGATGACGTTATAAAACAACAAACAGCTAAATTACTTGAAAGAACAGACAGAGAACCTCAAGTGTTCAAGGATATTGATTCTAGATTACCGGAGTTAATACAAAGATTAAACAAACAATTTCCTAATGACATCGGATTATTTTGTGGATGTCTCTTATTGAACCACGTTGATTGAAAAAATTATCAATTTCCGTGTTGGATAAACAGAGATTGACTGAAAAATTCAATAAATTGGATAAATCCATCAAAGATAATTTGAAGGCTAAACAAAAAGAAGAAACCAAAAAAACTTTAGATGTGGTTAATAATTGGTTGAATGATAAAGAAAATGCTTCATCATTTTTGGTTGCTCACGTTCCAATTACTGCTAATGCCAAGGCAATCACTGAAGCCATTAATTTGATTAAAAAGCAAGATAAAACCAAATCAATTTATTTATTGACTGGTGAAACCGATAAAGTTGCTCATGGATGTTATGTTAGTGATGAAGCCATTGCCAAGGGTATTAATGCGAAAATCAAACCTTTTGAAGCACGAGCAATTAACTGGTCCACGGATCTTAATGCTGAGGTACATATTGAGCATTATATAAATATATTCAATTATGCACGATCATCTTGGGAGCCATTGGTTGAAAGTTGGCCAATAGCAGTTTACATGTCAAAATCCCGACACCCAAAGCCTCAATTATTAGTAGAGGTGATTTCTAGACAGGTAGCCCAAGTGACGCTTACATCCAAAGCAGTAGCATTGCTATCTCAAGTATCCGATTTGATTACTTCCGGAGAAAAATTAAAACCAAGAGGTGAAGATTACCCATACGTTATAGTGAATGAGACTGGTTTAGATTTGGAAGTTTGGAATGATGCAAAAGAATCCGAAACCAATGGAGTTCCAATTGTTTTACGTGCTGGTAAAGCTTTAGATGAAAGTAAAGTTGAAATTAGAATTCAATTTAAACCAGTCGCCAAGGGGATGTTTAAAGAAATTCAAAGAAATGAATTAGTTATTAGAGTACAACCAAATGAAGCCATTTATTTAAAAATTAATTCCAAAATCCCTGGAATTTCTACTGAAACTTCATTAACTGATTTAGATTTAACTTATGCTACTCGTTATTCTAAAGAYTTTTGGATTCCTGAAGCTTATGAAGCATTAATTAGAGATTGYTATTTAGGTAATCATTCTAATTTTGTTAGAGATGATGAATTGGATGTTTCTTGGAAATTATTTACTCCATTATTGAAT

>HUN95

AATTATTGAARTGGAATGATATTCCATTGGCTCCACCAGACAARATTTTGGGTATTTCTGAAGCTTATAACAATGATTCTAACCCTCAAAAARTCAATTTGGGGGTTGGTGCTTATAGAGATAATTCYGGTAAACCAATTATTTTCCCATCAGTTAAAAAAGCTGAAGAAATTTTATTGGGTAAAGAAACTGAAAAGGAATATACTGCCATTGTTGGTTCCAAAAATTTCCAATCAATTGTGAAAAATTTCATTTTCAACAATTCTAATAAAGATGCCAATGGTAAACAATTAATTGATGATGGTAGAATTGTTACTGCTCAAACCATCTCTGGTACTGGATCACTTAAACCTCTTATGGTGAAATTTTTGCTAAACATAGAGAACCAAATTTGGAAATTATTCGTGAGGTTGTTGATTCCAAACATATTGTTTTTGATGTGTTGGCACAATTCTTAATCAATCCAGACCCATGGGTTGCCATTGCTGCCGCTGAAGTTTATGTCAGACGTTCATACCGTGCTTATGATTTGGGTAMAATTGAATATCATGTTAATGACAGACTTCCTATTGTTGAATGGAAATTCAAGTTGGCTAATATGGGAGCCGCTGGTGTAAACGATGCTCAACAGGCTGCTGCTGCYGGTGGCGATGATTCGACATCTATGAAACATGCAGCTTCTGTGTCTGATTTGACCTTTGTTGTTGATTATTCATRAAAGCGAAATCCCAGGATACACTCTCCCCGATAATCCAAAGTTCACCCTTGGTAATTTGTTTGTAATAATTGGAGTCTTGTTGGTTTGTATTTTAGCTGTTGTCTCTCTTTTGAGAAATATTAGTGAGTCGGCCTTGTTCAAGAAGAATGGGTATGAACCGTTGGATTCGGATCCTAGTGTCATGAACCTAAACTTCGAGCCTACAACATTGTCCTTTGAAGATATTAAATATGAGGTTACTGGTGGTCGACAAATTTTAAATGGAGTCTTTGGGTTTGTAAAACCAAGAGAATGTTTGGCTATAATGGGAGGTTCAGGTGCTGGTAAAACTACATTGTTGGATTTTAAACCTTTGGACCAATTGGCTAAGACTTTGACCACTGTTCCTGAATTGAATGAAATTATTGGGCAAGATTTAGTTGACGAATTTGTCAGTGGTATTAAACTACCAGCAGAAGTTGGAAGTCAAGATGATGTTAACAATAGAAAATTGTTGCAAAAAGTGTTTGGTAAATTAATGAACACTGATGATGACGTTATAAAACAACAAACAGCTAAATTACTTGAAAGAACAGACAGAGAACCTCAAGTGTTCAAGGATATTGATTCTAGATTACCGGAGTTAATACAAAGATTAAACAAACAATTTCCTAATGACATCGGATTATTTTGTGGATGTCTCTTATTGAACCACGTTGATTGAAAAAATTATCAATTTCCGTGTTGGATAAACAGAGATTGACTGAAAAATTCAATAAATTGGATAAATCCATCAAAGATAATTTGAAGGCTAAACAAAAAGAAGAAACCAAAAAAACTTTAGATGTGGTTAATAATTGGTTGAATGATAAAGAAAATGCTTCATCATTTTTGGTTGCTCACGTTCCAATTACTGCTAATGCCAAGGCAATCACTGAAGCYATTAATTTGATTAAAAAGCAAGATAAAACCAAATCAATTTATTTATTGACTGGTGAAACCGATAAAGTTGCTCATGGATGTTATGTTAGTGATGAAGCCATTGCCAAGGGTATTAATGCGAAAATCAAACCTTTTGAAGCACGAGCAATTAACTGGTCCACGGATCTTAATGCTGAGGTACATATTGAGCATTATATAAATATATTCAATTATGCACGATCATCTTGGGAGCCATTGGTTGAAAGTTGGCCAATAGCAGTTTACATGTCAAAATCCCGACACCCAAAGCCTCAATTATTAGTAGAGGTGATTTCTAGACAGGTAGCYCAAGTGACGCTTACATCCAAAGCAGTAGCATTGCTATCTCAAGTATCCGATTTGATTACTTCCRGAGAAAAATTAAAACCAAGAGGTGAAGATTACCCATACGTKATAGTGAATGAGACTGGTTTAGATTTGGAAGTTTGGAATGATGCAAAWGAATYCGAAACCAATGGAGTTCCAATTGTTTTACGTGCTGGTAAAGCTTTAGATGAAAGTAAAGTTGAAATTAGAATTCAATTTAAACCAGTCGCCAAGGGGATGTTTAAAGAAATTCAAAGAAATGAATTAGTTATTAGAGTACAACCAAATGAAGCCATTTATTTAAAAATTAATTCCAAAATCCCTGGAATTTCTACTGAAACTTCATTAACTGATTTAGATTTAACTTATGCTACTCGTTATTCTAAAGATTTTTGGATTCCTGAAGCTTATGAAGCATTAATTAGAGATTGTTATTTAGGTAATCATTCTAATTTTGTTAGAGATGATGAATTGGATGTTTCTTGGAAATTATTTACTCCATTATTGAAT

>HUN96

AATTATTGAARTGGAATGATATTCCATTGGCTCCACCAGACAARATTTTGGGTATTTCTGAAGCTTATAACAATGATTCTAACCCTCAAAAARTCAATTTGGGGGTTGGTGCTTATAGAGATAATTCYGGTAAACCAATTATTTTCCCATCAGTTAAAAAAGCTGAAGAAATTTTATTGGGTAAAGAAACTGAAAAGGAATATACTGCCATTGTTGGTTCCAAAAATTTCCAATCAATTGTGAAAAATTTCATTTTCAACAATTCTAATAAAGATGCCAATGGTAAACAATTAATTGATGATGGTAGAATTGTTACTGCTCAAACCATCTCTGGTACTGGATCACTTAAACCTCTTATGGTGAAATTTTTGCTAAACATAGAGAACCAAATTTGGAAATTATTCGTGAGGTTGTTGATTCCAAACATATTGTTTTTGATGTGTTGGCACAATTCTTAATCAATCCAGACCCATGGGTTGCCATTGCTGCCGCTGAAGTTTATGTCAGACGTTCATACCGTGCTTATGATTTGGGTAMAATTGAATATCATGTTAATGACAGACTTCCTATTGTTGAATGGAAATTCAAGTTGGCTAATATGGGAGCCGCTGGTGTAAACGATGCTCAACAGGCTGCTGCTGCYGGTGGCGATGATTCGACATCTATGAAACATGCAGCTTCTGTGTCTGATTTGACCTTTGTTGTTGATTATTCATRAAAGCGAAATCCCAGGATACACTCTCCCCGATAATCCAAAGTTCACCCTTGGTAATTTGTTTGTAATAATTGGAGTCTTGTTGGTTTGTATTTTAGCTGTTGTCTCTCTTTTGAGAAATATTAGTGAGTCGGCCTTGTTCAAGAAGAATGGGTATGAACCGTTGGATTCGGATCCTAGTGTCATGAACCTAAACTTCGAGCCTACAACATTGTCCTTTGAAGATATTAAATATGAGGTTACTGGTGGTCGACAAATTTTAAATGGAGTCTTTGGGTTTGTAAAACCAAGAGAATGTTTGGCTATAATGGGAGGTTCAGGTGCTGGTAAAACTACATTGTTGGATTTTAAACCTTTGGACCAATTGGCTAARACTTTGRCYACTGTTCCTGAATTGAATGAAATTATTGGGCAAGATTTAGTTGACGAATTTGTCAGTGGTATTAAACTACCAGCAGAAGTTGGAAGTCAAGATGATGTTAACAATAGAAAATTGTTGCAAAAAGTGTTTGGTAAATTAATGAACACTGATGATGACGTTATAAAACAACAAACAGCTAAATTACTTGAAAGAACAGACAGAGAACCTCAAGTGTTCAAGGATATTGATTCTAGATTACCGGAGTTAATACAAAGATTAAACAAACAATTTCCTAATGACATCGGATTATTTTGTGGATGTCTCTTATTGAACCACGTTGATTGAAAAAATTATCAATTTCCGTGTTGGATAAACAGAGATTGACTGAAAAATTCAATAAATTGGATAAATCCATCAAAGATAATTTGAAGGCTAAACAAAAAGAAGAAACCAAAAAAACTTTAGATGTGGTTAATAATTGGTTGAATGATAAAGAAAATGCTTCATCATTTTTGGTTGCTCACGTTCCAATTACTGCTAATGCCAAGGCAATCACTGAAGCCATTAATTTGATTAAAAAGCAAGATAAAACCAAATCAATTTATTTATTGACTGGTGAAACCGATAAAGTTGCTCATGGATGTTATGTTAGTGATGAAGCCATTGCCAAGGGTATTAATGCGAAAATCAAACCTTTTGAAGCACGAGCAATTAACTGGTCCACGGATCTTAATGCTGAGGTACATATTGAGCATTATATAAATATATTCAATTATGCACGATCATCTTGGGAGCCATTGGTTGAAAGTTGGCCAATAGCAGTTTACATGTCAAAATCCCGACACCCAAAGCCTCAATTATTAGTAGAGGTGATTTCTAGACAGGTAGCCCAAGTGACGCTTACATCCAAAGCAGTAGCATTGCTATCTCAAGTATCCGATTTGATTACTTCCGGAGAAAAATTAAAACCAAGAGGTGAAGATTACCCATACGTTATAGTGAATGAGACTGGTTTAGATTTGGAAGTTTGGAATGATGCAAAAGAATCCGAAACCAATGGAGTTCCAATTGTTTTACGTGCTGGTAAAGCTTTAGATGAAAGTAAAGTTGAAATTAGAATTCAATTTAAACCAGTCGCCAAGGGGATGTTTAAAGAAATTCAAAGAAATGAATTAGTTATTAGAGTACAACCAAATGAAGCCATTTATTTAAAAATTAATTCCAAAATCCCTGGAATTTCTACTGAAACTTCATTAACTGATTTAGATTTAACTTATGCTACTCGTTATTCTAAAGAYTTTTGGATTCCTGAAGCTTATGAAGCATTAATTAGAGATTGYTATTTAGGTAATCATTCTAATTTTGTTAGAGATGATGAATTGGATGTTTCTTGGAAATTATTTACTCCATTATTGAAT

>HUN122

AATTATTGAARTGGAATGATATTCCATTGGCTCCACCAGACAAAATTTTGGGTATTTCTGAAGCTTATAACAATGATTCTAACCCTCAAAAAATCAATTTGGGGGTTGGTGCTTATAGAGATAATTCCGGTAAACCAATTATTTTCCCATCAGTTAAAAAAGCTGAAGAAATTTTATTGGGTAAAGAAACTGAAAAGGAATATACTGCCATTGTTGGTTCCAAAAATTTCCAATCAATTGTGAAAAATTTCATTTTCAACAATTCTAATAAAGATGCCAATGGTAAACAATTAATTGATGATGGTAGAATTGTTACTGCTCAAACCATCTCTGGTACTGGATCACTTAAACCTCTTATGGTGAAATTTTTGCTAAACATAGAGAACCAAATTTGGAAATTATTCGTGAGGTTGTTGATTCCAAACATATTGTTTTTGATGTGTTGGCACAATTCTTAATCAATCCAGACCCATGGGTTGCCATTGCTGCCGCTGAAGTTTATGTCAGACGTTCATACCGTGCTTATGATTTGGGTAAAATTGAATATCATGTTAATGACAGACTTCCTATTGTTGAATGGAAATTCAAGTTGGCTAATATGGGAGCCGCTGGTGTAAACGATGCTCAACAGGCTGCTGCTGCCGGTGGCGATGATTCGACATCTATGAAACATGCAGCTTCTGTGTCTGATTTGACCTTTGTTGTTGATTATTCATAAAAGYGAAATYCCAGGATACACTCTCCCCGATAATCCAAAGTTCACCCTTGGTAATTTGTTTGTAATAATTGGRGTCTTGTTGGTTTGTRTTTTAGCTGTTGTCTCTCTTTTGAGAAATATTAGTGAGTCRGCCTTGTTCAAGAAGAATGGGTATGAACCGTTGGATTCRGATCCTAGTGTCATGAACCWAAACTTYGAGCCTACAACATTGTCCTTTGAAGATATTAAATATGAGGTTACTGGTGGTCGACAAATTTTAAATGGAGTCTTTGGGTTTGTAAAACCAAGAGAATGTTTGGCTATAATGGGAGGTTCAGGTGCTGGTAAAACTACATTGTTGGATTTTAAACCTTTGGACCAATTGGCTAAGACTTTGACCACTGTTCCTGAATTGAATGAAATTATTGGGCAAGATTTAGTTGACGAATTTGTCAGTGGTATTAAACTACCAGCAGAAGTTGGAAGTCAAGATGATGTTAACAATAGAAAATTGTTGCAAAAAGTGTTTGGTAAATTAATGAACACTGATGATGACGTTATAAAACAACAAACAGCTAAATTACTTGAAAGAACAGACAGAGAACCTCAAGTGTTCAAGGATATTGATTCTAGATTACCGGAGTTAATACAAAGATTAAACAAACAATTTCCTAATGACATCGGATTATTTTGTGGATGTCTCTTATTGAACCACGTTGATTGAAAAAATTATCAATTTCCGTGTTGGATAAACAAAGATTGACTGAAAAATTCAATAAATTGGATAAATCCATTAAAGATAATTTGAAGGCTAAACAAAAAGAAGAAACCAAAAAGACTTTAGATGTGGTTAACAATTGGTTGAATGATAAAGAAAATGCTTCATCATTTTTGGTTGCTCACGTTCCAATTACTGCTAATGCCAAGGCAATCACTGAAGCCATTAATTTGATTAAAAAGCAAGATAAAACCAAATCAATTTATTTATTGACTGGTGAAACAGATAAAGTTGCTCATGGATGTTATGTTAGTGATGAAGCCATTGCCAAGGGTATTAATGCGAAAATCAAACCTTTTGAAGCACGAGCAATTAACTGGTCMACGGATCTTAATGCTGAGGTACATATTGAGCATTATATAAATATATTCAATTATGCACGATCATCTTGGGAGCCATTGGTTGAAAGTTGGCCAATAGCAGTTTACATGTCAAAATCCCGACACCCAAAGCCTCAATTATTAGTAGAGGTGATTTCTAGACAGGTAGCTCAAGTGACGCTTACATCCAAAGCAGTAGCATTGCTATCTCAAGTATCCGATTTGATTACTTCCRGAGAAAAATTAAAACCAAGAGGTGAAGATTACCCATACRTKATAGTGAATGAGACTGGTTTAGATTTGGAAGTTTGGAATGATGCAAAWGAATYCGAAACCAATGGAGTTCCAATTGTTTTACGTGCTGGTAAAGCTTTAGATGAAAGTAAAGTTGAAATTAGAATTCAATTTAAACCAGTCGCCAAGGGGATGTTTAAAGAAATTCAAAGAAATGAATTAGTTATTAGAGTACAACCAAATGAAGCCATTTATTTAAAAATTAATTCCAAAATCCCTGGAATTTCTACTGAAACTTCATTAACTGATTTAGATTTAACTTATGCTACTCGTTATTCTAAAGATTTTTGGATTCCTGAAGCTTATGAAGCATTAATTAGAGATTGTTATTTAGGTAATCATTCTAATTTTGTTAGAGATGATGAATTGGATGTTTCTTGGAAATTATTTACTCCATTATTGAAT

>HUN122:IND:6:4.7

AATTATTGAARTGGAATGATATTCCATTGGCTCCACCAGACAAAATTTTGGGTATTTCTGAAGCTTATAACAATGATTCTAACCCTCAAAAAATCAATTTGGGGGTTGGTGCTTATAGAGATAATTCCGGTAAACCAATTATTTTCCCATCAGTTAAAAAAGCTGAAGAAATTTTATTGGGTAAAGAAACTGAAAAGGAATATACTGCCATTGTTGGTTCCAAAAATTTCCAATCAATTGTGAAAAATTTCATTTTCAACAATTCTAATAAAGATGCCAATGGTAAACAATTAATTGATGATGGTAGAATTGTTACTGCTCAAACCATCTCTGGTACTGGATCACTTAAACCTCTTATGGTGAAATTTTTGCTAAACATAGAGAACCAAATTTGGAAATTATTCGTGAGGTTGTTGATTCCAAACATATTGTTTTTGATGTGTTGGCACAATTCTTAATCAATCCAGACCCATGGGTTGCCATTGCTGCCGCTGAAGTTTATGTCAGACGTTCATACCGTGCTTATGATTTGGGTAAAATTGAATATCATGTTAATGACAGACTTCCTATTGTTGAATGGAAATTCAAGTTGGCTAATATGGGAGCCGCTGGTGTAAACGATGCTCAACAGGCTGCTGCTGCCGGTGGCGATGATTCGACATCTATGAAACATGCAGCTTCTGTGTCTGATTTGACCTTTGTTGTTGATTATTCATAAAAGCGAAATCCCAGGATACACTCTCCCCGATAATCCAAAGTTCACCCTTGGTAATTTGTTTGTAATAATTGGAGTCTTGTTGGTTTGTATTTTAGCTGTTGTCTCTCTTTTGAGAAATATTAGTGAGTCGGCCTTGTTCAAGAAGAATGGGTATGAACCGTTGGATTCGGATCCTAGTGTCATGAACCTAAACTTCGAGCCTACAACATTGTCCTTTGAAGATATTAAATATGAGGTTACTGGTGGTCGACAAATTTTAAATGGAGTCTTTGGGTTTGTAAAACCAAGAGAATGTTTGGCTATAATGGGAGGTTCAGGTGCTGGTAAAACTACATTGTTGGATTTTAAACCTTTGGACCAATTGGCTAAGACTTTGACCACTGTTCCTGAATTGAATGAAATTATTGGGCAAGATTTAGTTGACGAATTTGTCAGTGGTATTAAACTACCAGCAGAAGTTGGAAGTCAAGATGATGTTAACAATAGAAAATTGTTGCAAAAAGTGTTTGGTAAATTAATGAACACTGATGATGACGTTATAAAACAACAAACAGCTAAATTACTTGAAAGAACAGACAGAGAACCTCAAGTGTTCAAGGATATTGATTCTAGATTACCGGAGTTAATACAAAGATTAAACAAACAATTTCCTAATGACATCGGATTATTTTGTGGATGTCTCTTATTGAACCACGTTGATTGAAAAAATTATCAATTTCCGTGTTGGATAAACAAAGATTGACTGAAAAATTCAATAAATTGGATAAATCCATCAAAGATAATTTGAAGGCTAAACAAAAAGAAGAAACCAAAAAAACTTTAGATGTGGTTAATAATTGGTTGAATGATAAAGAAAATGCTTCATCATTTTTGGTTGCTCACGTTCCAATTACTGCTAATGCCAAGGCAATCACTGAAGCCATTAATTTGATTAAAAAGCAAGATAAAACCAAATCAATTTATTTATTGACTGGTGAAACAGATAAAGTTGCTCATGGATGTTATGTTAGTGATGAAGCCATTGCCAAGGGTATTAATGCGAAAATCAAACCTTTTGAAGCACGAGCAATTAACTGGTCCACGGATCTTAATGCTGAGGTACATATTGAGCATTATATAAATATATTCAATTATGCACGATCATCTTGGGAGCCATTGGTTGAAAGTTGGCCAATAGCAGTTTACATGTCAAAATCCCGACACCCAAAGCCTCAATTATTAGTAGAGGTGATTTCTAGACAGGTAGCTCAAGTGACGCTTACATCCAAAGCAGTAGCATTGCTATCTCAAGTATCCGATTTGATTACTTCCRGAGAAAAATTAAAACCAAGAGGTGAAGATTACCCATACGTKATAGTGAATGAGACTGGTTTAGATTTGGAAGTTTGGAATGATGCAAAWGAATYCGAAACCAATGGAGTTCCAATTGTTTTACGTGCTGGTAAAGCTTTAGATGAAAGTAAAGTTGAAATTAGAATTCAATTTAAACCAGTCGCCAAGGGGATGTTTAAAGAAATTCAAAGAAATGAATTAGTTATTAGAGTACAACCAAATGAAGCCATTTATTTAAAAATTAATTCCAAAATCCCTGGAATTTCTACTGAAACTTCATTAACTGATTTAGATTTAACTTATGCTACTCGTTATTCTAAAGAYTTTTGGATTCCTGAAGCTTATGAAGCATTAATTAGAGATTGYTATTTAGGTAATCATTCTAATTTTGTTAGAGATGATGAATTGGATGTTTCTTGGAAATTATTTACTCCATTATTGAAT

>HUN122:IND:17:7.8

AATTATTGAARTGGAATGATATTCCATTGGCTCCACCAGACAAAATTTTGGGTATTTCTGAAGCTTATAACAATGATTCTAACCCTCAAAAAATCAATTTGGGGGTTGGTGCTTATAGAGATAATTCCGGTAAACCAATTATTTTCCCATCAGTTAAAAAAGCTGAAGAAATTTTATTGGGTAAAGAAACTGAAAAGGAATATACTGCCATTGTTGGTTCCAAAAATTTCCAATCAATTGTGAAAAATTTCATTTTCAACAATTCTAATAAAGATGCCAATGGTAAACAATTAATTGATGATGGTAGAATTGTTACTGCTCAAACCATCTCTGGTACTGGATCACTTAAACCTCTTATGGTGAAATTTTTGCTAAACATAGAGAACCAAATTTGGAAATTATTCGTGAGGTTGTTGATTCCAAACATATTGTTTTTGATGTGTTGGCACAATTCTTAATCAATCCAGACCCATGGGTTGCCATTGCTGCCGCTGAAGTTTATGTCAGACGTTCATACCGTGCTTATGATTTGGGTAAAATTGAATATCATGTTAATGACAGACTTCCTATTGTTGAATGGAAATTCAAGTTGGCTAATATGGGAGCCGCTGGTGTAAACGATGCTCAACAGGCTGCTGCTGCCGGTGGCGATGATTCGACATCTATGAAACATGCAGCTTCTGTGTCTGATTTGACCTTTGTTGTTGATTATTCATAAAAGYGAAATYCCAGGATACACTCTCCCCGATAATCCAAAGTTCACCCTTGGTAATTTGTTTGTAATAATTGGRGTCTTGTTGGTTTGTRTTTTAGCTGTTGTCTCTCTTTTGAGAAATATTAGTGAGTCRGCCTTGTTCAAGAAGAATGGGTATGAACCGTTGGATTCRGATCCTAGTGTCATGAACCWAAACTTYGAGCCTACAACATTGTCCTTTGAAGATATTAAATATGAGGTTACTGGTGGTCGACAAATTTTAAATGGAGTCTTTGGGTTTGTAAAACCAAGAGAATGTTTGGCTATAATGGGAGGTTCAGGTGCTGGTAAAACTACATTGTTGGATTTTAAACCTTTGGACCAATTGGCTAAGACTTTGACCACTGTTCCTGAATTGAATGAAATTATTGGGCAAGATTTAGTTGACGAATTTGTCAGTGGTATTAAACTACCAGCAGAAGTTGGAAGTCAAGATGATGTTAACAATAGAAAATTGTTGCAAAAAGTGTTTGGTAAATTAATGAACACTGATGATGACGTTATAAAACAACAAACAGCTAAATTACTTGAAAGAACAGACAGAGAACCTCAAGTGTTCAAGGATATTGATTCTAGATTACCGGAGTTAATACAAAGATTAAACAAACAATTTCCTAATGACATCGGATTATTTTGTGGATGTCTCTTATTGAACCACGTTGATTGAAAAAATTATCAATTTCCGTGTTGGATAAACAAAGATTGACTGAAAAATTCAATAAATTGGATAAATCCATTAAAGATAATTTGAAGGCTAAACAAAAAGAAGAAACCAAAAAGACTTTAGATGTGGTTAACAATTGGTTGAATGATAAAGAAAATGCTTCATCATTTTTGGTTGCTCACGTTCCAATTACTGCTAATGCCAAGGCAATCACTGAAGCCATTAATTTGATTAAAAAGCAAGATAAAACCAAATCAATTTATTTATTGACTGGTGAAACAGATAAAGTTGCTCATGGATGTTATGTTAGTGATGAAGCCATTGCCAAGGGTATTAATGCGAAAATCAAACCTTTTGAAGCACGAGCAATTAACTGGTCMACGGATCTTAATGCTGAGGTACATATTGAGCATTATATAAATATATTCAATTATGCACGATCATCTTGGGAGCCATTGGTTGAAAGTTGGCCAATAGCAGTTTACATGTCAAAATCCCGACACCCAAAGCCTCAATTATTAGTAGAGGTGATTTCTAGACAGGTAGCTCAAGTGACGCTTACATCCAAAGCAGTAGCATTGCTATCTCAAGTATCCGATTTGATTACTTCCRGAGAAAAATTAAAACCAAGAGGTGAAGATTACCCATACRTKATAGTGAATGAGACTGGTTTAGATTTGGAAGTTTGGAATGATGCAAAWGAATYCGAAACCAATGGAGTTCCAATTGTTTTACGTGCTGGTAAAGCTTTAGATGAAAGTAAAGTTGAAATTAGAATTCAATTTAAACCAGTCGCCAAGGGGATGTTTAAAGAAATTCAAAGAAATGAATTAGTTATTAGAGTACAACCAAATGAAGCCATTTATTTAAAAATTAATTCCAAAATCCCTGGAATTTCTACTGAAACTTCATTAACTGATTTAGATTTAACTTATGCTACTCGTTATTCTAAAGATTTTTGGATTCCTGAAGCTTATGAAGCATTAATTAGAGATTGTTATTTAGGTAATCATTCTAATTTTGTTAGAGATGATGAATTGGATGTTTCTTGGAAATTATTTACTCCATTATTGAAT

>HUN122:IND:41:4.4

AATTATTGAARTGGAATGATATTCCATTGGCTCCACCAGACAAAATTTTGGGTATTTCTGAAGCTTATAACAATGATTCTAACCCTCAAAAAATCAATTTGGGGGTTGGTGCTTATAGAGATAATTCCGGTAAACCAATTATTTTCCCATCAGTTAAAAAAGCTGAAGAAATTTTATTGGGTAAAGAAACTGAAAAGGAATATACTGCCATTGTTGGTTCCAAAAATTTCCAATCAATTGTGAAAAATTTCATTTTCAACAATTCTAATAAAGATGCCAATGGTAAACAATTAATTGATGATGGTAGAATTGTTACTGCTCAAACCATCTCTGGTACTGGATCACTTAAACCTCTTATGGTGAAATTTTTGCTAAACATAGAGAACCAAATTTGGAAATTATTCGTGAGGTTGTTGATTCCAAACATATTGTTTTTGATGTGTTGGCACAATTCTTAATCAATCCAGACCCATGGGTTGCCATTGCTGCCGCTGAAGTTTATGTCAGACGTTCATACCGTGCTTATGATTTGGGTAAAATTGAATATCATGTTAATGACAGACTTCCTATTGTTGAATGGAAATTCAAGTTGGCTAATATGGGAGCCGCTGGTGTAAACGATGCTCAACAGGCTGCTGCTGCCGGTGGCGATGATTCGACATCTATGAAACATGCAGCTTCTGTGTCTGATTTGACCTTTGTTGTTGATTATTCATAAAAGYGAAATYCCAGGATACACTCTCCCCGATAATCCAAAGTTCACCCTTGGTAATTTGTTTGTAATAATTGGRGTCTTGTTGGTTTGTRTTTTAGCTGTTGTCTCTCTTTTGAGAAATATTAGTGAGTCRGCCTTGTTCAAGAAGAATGGGTATGAACCGTTGGATTCRGATCCTAGTGTCATGAACCWAAACTTYGAGCCTACAACATTGTCCTTTGAAGATATTAAATATGAGGTTACTGGTGGTCGACAAATTTTAAATGGAGTCTTTGGGTTTGTAAAACCAAGAGAATGTTTGGCTATAATGGGAGGTTCAGGTGCTGGTAAAACTACATTGTTGGATTTTAAACCTTTGGACCAATTGGCTAAGACTTTGACCACTGTTCCTGAATTGAATGAAATTATTGGGCAAGATTTAGTTGACGAATTTGTCAGTGGTATTAAACTACCAGCAGAAGTTGGAAGTCAAGATGATGTTAACAATAGAAAATTGTTGCAAAAAGTGTTTGGTAAATTAATGAACACTGATGATGACGTTATAAAACAACAAACAGCTAAATTACTTGAAAGAACAGACAGAGAACCTCAAGTGTTCAAGGATATTGATTCTAGATTACCGGAGTTAATACAAAGATTAAACAAACAATTTCCTAATGACATCGGATTATTTTGTGGATGTCTCTTATTGAACCACGTTGATTGAAAAAATTATCAATTTCCGTGTTGGATAAACAAAGATTGACTGAAAAATTCAATAAATTGGATAAATCCATTAAAGATAATTTGAAGGCTAAACAAAAAGAAGAAACCAAAAAGACTTTAGATGTGGTTAACAATTGGTTGAATGATAAAGAAAATGCTTCATCATTTTTGGTTGCTCACGTTCCAATTACTGCTAATGCCAAGGCAATCACTGAAGCCATTAATTTGATTAAAAAGCAAGATAAAACCAAATCAATTTATTTATTGACTGGTGAAACAGATAAAGTTGCTCATGGATGTTATGTTAGTGATGAAGCCATTGCCAAGGGTATTAATGCGAAAATCAAACCTTTTGAAGCACGAGCAATTAACTGGTCMACGGATCTTAATGCTGAGGTACATATTGAGCATTATATAAATATATTCAATTATGCACGATCATCTTGGGAGCCATTGGTTGAAAGTTGGCCAATAGCAGTTTACATGTCAAAATCCCGACACCCAAAGCCTCAATTATTAGTAGAGGTGATTTCTAGACAGGTAGCTCAAGTGACGCTTACATCCAAAGCAGTAGCATTGCTATCTCAAGTATCCGATTTGATTACTTCCRGAGAAAAATTAAAACCAAGAGGTGAAGATTACCCATACRTKATAGTGAATGAGACTGGTTTAGATTTGGAAGTTTGGAATGATGCAAAWGAATYCGAAACCAATGGAGTTCCAATTGTTTTACGTGCTGGTAAAGCTTTAGATGAAAGTAAAGTTGAAATTAGAATTCAATTTAAACCAGTCGCCAAGGGGATGTTTAAAGAAATTCAAAGAAATGAATTAGTTATTAGAGTACAACCAAATGAAGCCATTTATTTAAAAATTAATTCCAAAATCCCTGGAATTTCTACTGAAACTTCATTAACTGATTTAGATTTAACTTATGCTACTCGTTATTCTAAAGATTTTTGGATTCCTGAAGCTTATGAAGCATTAATTAGAGATTGTTATTTAGGTAATCATTCTAATTTTGTTAGAGATGATGAATTGGATGTTTCTTGGAAATTATTTACTCCATTATTGAAT

>HUN122:IND:65:3.0

AATTATTGAARTGGAATGATATTCCATTGGCTCCACCAGACAAAATTTTGGGTATTTCTGAAGCTTATAACAATGATTCTAACCCTCAAAAAATCAATTTGGGGGTTGGTGCTTATAGAGATAATTCCGGTAAACCAATTATTTTCCCATCAGTTAAAAAAGCTGAAGAAATTTTATTGGGTAAAGAAACTGAAAAGGAATATACTGCCATTGTTGGTTCCAAAAATTTCCAATCAATTGTGAAAAATTTCATTTTCAACAATTCTAATAAAGATGCCAATGGTAAACAATTAATTGATGATGGTAGAATTGTTACTGCTCAAACCATCTCTGGTACTGGATCACTTAAACCTCTTATGGTGAAATTTTTGCTAAACATAGAGAACCAAATTTGGAAATTATTCGTGAGGTTGTTGATTCCAAACATATTGTTTTTGATGTGTTGGCACAATTCTTAATCAATCCAGACCCATGGGTTGCCATTGCTGCCGCTGAAGTTTATGTCAGACGTTCATACCGTGCTTATGATTTGGGTAAAATTGAATATCATGTTAATGACAGACTTCCTATTGTTGAATGGAAATTCAAGTTGGCTAATATGGGAGCCGCTGGTGTAAACGATGCTCAACAGGCTGCTGCTGCCGGTGGCGATGATTCGACATCTATGAAACATGCAGCTTCTGTGTCTGATTTGACCTTTGTTGTTGATTATTCATAAAAGYGAAATYCCAGGATACACTCTCCCCGATAATCCAAAGTTCACCCTTGGTAATTTGTTTGTAATAATTGGRGTCTTGTTGGTTTGTRTTTTAGCTGTTGTCTCTCTTTTGAGAAATATTAGTGAGTCRGCCTTGTTCAAGAAGAATGGGTATGAACCGTTGGATTCRGATCCTAGTGTCATGAACCWAAACTTYGAGCCTACAACATTGTCCTTTGAAGATATTAAATATGAGGTTACTGGTGGTCGACAAATTTTAAATGGAGTCTTTGGGTTTGTAAAACCAAGAGAATGTTTGGCTATAATGGGAGGTTCAGGTGCTGGTAAAACTACATTGTTGGATTTTAAACCTTTGGACCAATTGGCTAAGACTTTGACCACTGTTCCTGAATTGAATGAAATTATTGGGCAAGATTTAGTTGACGAATTTGTCAGTGGTATTAAACTACCAGCAGAAGTTGGAAGTCAAGATGATGTTAACAATAGAAAATTGTTGCAAAAAGTGTTTGGTAAATTAATGAACACTGATGATGACGTTATAAAACAACAAACAGCTAAATTACTTGAAAGAACAGACAGAGAACCTCAAGTGTTCAAGGATATTGATTCTAGATTACCGGAGTTAATACAAAGATTAAACAAACAATTTCCTAATGACATCGGATTATTTTGTGGATGTCTCTTATTGAACCACGTTGATTGAAAAAATTATCAATTTCCGTGTTGGATAAACAAAGATTGACTGAAAAATTCAATAAATTGGATAAATCCATTAAAGATAATTTGAAGGCTAAACAAAAAGAAGAAACCAAAAAGACTTTAGATGTGGTTAACAATTGGTTGAATGATAAAGAAAATGCTTCATCATTTTTGGTTGCTCACGTTCCAATTACTGCTAATGCCAAGGCAATCACTGAAGCCATTAATTTGATTAAAAAGCAAGATAAAACCAAATCAATTTATTTATTGACTGGTGAAACAGATAAAGTTGCTCATGGATGTTATGTTAGTGATGAAGCCATTGCCAAGGGTATTAATGCGAAAATCAAACCTTTTGAAGCACGAGCAATTAACTGGTCMACGGATCTTAATGCTGAGGTACATATTGAGCATTATATAAATATATTCAATTATGCACGATCATCTTGGGAGCCATTGGTTGAAAGTTGGCCAATAGCAGTTTACATGTCAAAATCCCGACACCCAAAGCCTCAATTATTAGTAGAGGTGATTTCTAGACAGGTAGCTCAAGTGACGCTTACATCCAAAGCAGTAGCATTGCTATCTCAAGTATCCGATTTGATTACTTCCRGAGAAAAATTAAAACCAAGAGGTGAAGATTACCCATACRTKATAGTGAATGAGACTGGTTTAGATTTGGAAGTTTGGAATGATGCAAAWGAATYCGAAACCAATGGAGTTCCAATTGTTTTACGTGCTGGTAAAGCTTTAGATGAAAGTAAAGTTGAAATTAGAATTCAATTTAAACCAGTCGCCAAGGGGATGTTTAAAGAAATTCAAAGAAATGAATTAGTTATTAGAGTACAACCAAATGAAGCCATTTATTTAAAAATTAATTCCAAAATCCCTGGAATTTCTACTGAAACTTCATTAACTGATTTAGATTTAACTTATGCTACTCGTTATTCTAAAGATTTTTGGATTCCTGAAGCTTATGAAGCATTAATTAGAGATTGTTATTTAGGTAATCATTCTAATTTTGTTAGAGATGATGAATTGGATGTTTCTTGGAAATTATTTACTCCATTATTGAAT

>HUN123

AATTATTGAAATGGAATGATATTCCATTGGCTCCACCAGACAAAATTTTGGGTATTTCTGAAGCTTATAACAACGATTCTAACCCTCAAAAAATCAATTTGGGGGTTGGTGCTTATAGAGATAATTCCGGTAAACCAATTATTTTCCCATCAGTTAAAAAAGCTGAAGAAATTTTATTGGGTAAAGAAACTGAAAAGGAATATACTGCCATTGTTGGTTCCAAAAATTTCCAATCAATTGTGAAAAATTTCATTTTCAACAATTCTAATAAAGATGCCAATGGTAAACAATTAATTGATGATGGTAGAATTGTTACTGCTCAAACCATCTCTGGTACTGGATCACTTAAACCTCTTATGGTGAAATTTTTGCTAAACATAGAGAACCAAATTTGGAAATTATTCGTGAGGTTGTTGATTCCAAACATATTGTTTTTGATGTGTTGGCACAATTCTTAATCAATCCAGACCCATGGGTTGCCATTGCTGCCGCTGAAGTTTATGTCAGACGTTCATACCGTGCTTATGATTTGGGTACAATTGAATATCATGTTAATGACAGACTTCCTATTGTTGAATGGAAATTCAAGTTGGCTAATATGGGAGCCGCTGGTGTAAACGATGCTCAACAGGCTGCTGCTGCTGGTGGCGATGATTCGACATCTATGAAACATGCAGCTTCTGTGTCTGATTTGACCTTTGTTGTTGATTATTCATAAAAGCGAAATCCCAGGATACACTCTCCCCGATAATCCAAAGTTCACCCTTGGTAATTTGTTTGTAATAATTGGAGTCTTGTTGGTTTGTATTTTAGCTGTTGTCTCTCTTTTGAGAAATATTAGTGAGTCGGCCTTGTTCAAGAAGAATGGGTATGAACCGTTGGATTCGGATCCTAGTGTCATGAACCTAAACTTCGAGCCTACAACATTGTCCTTTGAAGATATTAAATATGAGGTTACTGGTGGTCGACAAATTTTAAATGGAGTCTTTGGGTTTGTAAAACCAAGAGAATGTTTGGCTATAATGGGAGGTTCAGGTGCTGGTAAAACTACATTGTTGGATTTTAAACCTTTGGACCAATTGGCTAAGACTTTGACCACTGTTCCTGAATTGAATGAAATTATTGGGCAAGATTTAGTTGACGAATTTGTCAGTGGTATTAAACTACCAGCAGAAGTTGGAAGTCAAGATGATGTTAACAATAGAAAATTGTTGCAAAAAGTGTTTGGTAAATTAATGAACACTGATGATGACGTTATAAAACAACAAACAGCTAAATTACTTGAAAGAACAGACAGAGAACCTCAAGTGTTCAAGGATATTGATTCTAGATTACCGGAGTTAATACAAAGATTAAACAAACAATTTCCTAATGACATCGGATTATTTTGTGGATGTCTCTTATTGAACCACGTTGMTTGAAAAAATTATCAATTTCCGTGTTGGATAAACAAAGATTGACTGAAAAATTCAATAAATTGGATAAATCCATCAAAGATAATTTGAAGGCTAAACAAAAAGAAGAAACCAAAAAAACTTTAGATGTGGTTAATAATTGGTTGAATGATAAAGAAAATTCTTCATCATTTTTGGTTGCTCACGTTCCAATTACTGCTAATGCCAAGGCAATCACTGAAGCCATTAATTTGATTAAAAAGCAAGATAAAACCAAATCAATTTATTTATTGACTGGTGAAACCGATAAAGTTGCTCATGGATGTTATGTTAGTGATGAAGCCATTGTCAAGGGTATTAATGCGAAAATCAAACCTTTTGAAGCACGAGCAATTAACTGGTCCACGGATCTTAATGCTGAGGTACATATTGAGCATTATATAAATATATTCAATTATGCACGATCATCTTGGGAGCCATTGGTTGAAAGTTGGCCAATAGCAGTTTACATGTCAAAATCCCGACACCCAAAGCCTCAATTATTAGTAGAGGTGATTTCTAGACAGGTAGCTCAAGTGACGCTTACATCCAAAGCRGTAGCATTGCTATCTCAAGTATCCGATTTGATTACTTCCRGAGAAAAATTAAAACCAAGAGGTGAAGATTACCCATACGTKATAGTGAATGAGACTGGTTTAGATTTGGAAGTTTGGAATGATGCAAATGAATYCGAAACCAATGGAGTTCCAATTGTTTTACGTGCTGGTAAAGCTTTAGATGAAAGTAAAGTTGAAATTAGAATTCAATTTAAACCAGTCGCCAAGGGGATGTTTAAAGAAATTCAAAGAAATGAATTAGTTATTAGAGTACAACCAAATGAAGCCATTTATTTAAAAATTAATTCCAAAATCCCTGGAATTTCTACTGAAACTTCATTAACTGATTTAGATTTAACTTATGCTACTCGTTATTCTAAAGACTTTTGGATTCCTGAAGCTTATGAAGCATTAATTAGAGATTGYTATTTAGGTAATCATTCTAATTTTGTTAGAGATGATGAATTGGATGTTTCTTGGAAATTATTTACTCCATTATTGAAT

>HUN127

AATTATTGAARTGGAATGATATTCCATTGGCTCCACCAGACAARATTTTGGGTATTTCTGAAGCTTATAACAATGATTCTAACCCTCAAAAARTCAATTTGGGGGTTGGTGCTTATAGAGATAATTCYGGTAAACCAATTATTTTCCCATCAGTTAAAAAAGCTGAAGAAATTTTATTGGGTAAAGAAACTGAAAAGGAATATACTGCCATTGTTGGTTCCAAAAATTTCCAATCAATTGTGAAAAATTTCATTTTCAACAATTCTAATAAAGATGCCAATGGTAAACAATTAATTGATGATGGTAGAATTGTTACTGCTCAAACCATCTCTGGTACTGGATCACTTAAACCTCTTATGGTGAAATTTTTGCTAAACATAGAGAACCAAATTTGGAAATTATTCGTGAGGTTGTTGATTCCAAACATATTGTTTTTGATGTGTTGGCACAATTCTTAATCAATCCAGACCCATGGGTTGCCATTGCTGCCGCTGAAGTTTATGTCAGACGTTCATACCGTGCTTATGATTTGGGTAMAATTGAATATCATGTTAATGACAGACTTCCTATTGTTGAATGGAAATTCAAGTTGGCTAATATGGGAGCCGCTGGTGTAAACGATGCTCAACAGGCTGCTGCTGCYGGTGGCGATGATTCGACATCTATGAAACATGCAGCTTCTGTGTCTGATTTGACCTTTGTTGTTGATTATTCATRAAAGCGAAATCCCAGGATACACTCTCCCCGATAATCCAAAGTTCACCCTTGGTAATTTGTTTGTAATAATTGGAGTCTTGTTGGTTTGTATTTTAGCTGTTGTCTCTCTTTTGAGAAATATTAGTGAGTCGGCCTTGTTCAAGAAGAATGGGTATGAACCGTTGGATTCGGATCCTAGTGTCATGAACCTAAACTTCGAGCCTACAACATTGTCCTTTGAAGATATTAAATATGAGGTTACTGGTGGTCGACAAATTTTAAATGGAGTCTTTGGGTTTGTAAAACCAAGAGAATGTTTGGCTATAATGGGAGGTTCAGGTGCTGGTAAAACTACATTGTTGGATTTTAAACCTTTGGACCAATTGGCTAARACTTTGRCYACTGTTCCTGAATTGAATGAAATTATTGGKCAAGAWTTAGTTGACGAATTTRTCAGTGGTATTAAACTACCAGCAGAAGTTGGAAGTCAAGATGATGTTAACAATAGAAAATTGTTGCAAAAAGTGTTTGGTAAATTAATGAACACTGATGATGACGTTATAAAACAACAAACAGCTAAATTACTTGAAAGAACAGACAGAGAACCTCAAGTGTTCAAGGATATTGATTCTAGATTACCGGAGTTAATACAAAGATTAAACAAACAATTTCCTAATGACATCGGATTATTTTGTGGATGTCTCTTATTGAACCACGTTGATTGAAAAAATTATCAATTTCCGTGTTGGATAAACAGAGATTGACTGAAAAATTCAATAAATTGGATAAATCCATCAAAGATAATTTGAAGGCTAAACAAAAAGAAGAAACCAAAAAAACTTTAGATGTGGTTAATAATTGGTTGAATGATAAAGAAAATGCTTCATCATTTTTGGTTGCTCACGTTCCAATTACTGCTAATGCCAAGGCAATCACTGAAGCCATTAATTTGATTAAAAAGCAAGATAAAACCAAATCAATTTATTTATTGACTGGTGAAACCGATAAAGTTGCTCATGGATGTTATGTTAGTGATGAAGCCATTGCCAAGGGTATTAATGCGAAAATCAAACCTTTTGAAGCACGAGCAATTAACTGGTCCACGGATCTTAATGCTGAGGTACATATTGAGCATTATATAAATATATTCAATTATGCACGATCATCTTGGGAGCCATTGGTTGAAAGTTGGCCAATAGCAGTTTACATGTCAAAATCCCGACACCCAAAGCCTCAATTATTAGTAGAGGTGATTTCTAGACAGGTAGCCCAAGTGACGCTTACATCCAAAGCAGTAGCATTGCTATCTCAAGTATCCGATTTGATTACTTCCGGAGAAAAATTAAAACCAAGAGGTGAAGATTACCCATACGTTATAGTGAATGAGACTGGTTTAGATTTGGAAGTTTGGAATGATGCAAAAGAATCCGAAACCAATGGAGTTCCAATTGTTTTACGTGCTGGTAAAGCTTTAGATGAAAGTAAAGTTGAAATTAGAATTCAATTTAAACCAGTCGCCAAGGGGATGTTTAAAGAAATTCAAAGAAATGAATTAGTTATTAGAGTACAACCAAATGAAGCCATTTATTTAAAAATTAATTCCAAAATCCCTGGAATTTCTACTGAAACTTCATTAACTGATTTAGATTTAACTTATGCTACTCGTTATTCTAAAGAYTTTTGGATTCCTGAAGCTTATGAAGCATTAATTAGAGATTGYTATTTAGGTAATCATTCTAATTTTGTTAGAGATGATGAATTGGATGTTTCTTGGAAATTATTTACTCCATTATTGAAT

>Mi1_oral_27/02/2012

AATTATTGAAGTGGAATGATATTCCATTGGCTCCACCAGACAAAATTTTGGGTATTTCTGAAGCTTATAACAATGATTCTAACCCTCAAAAAATCAATTTGGGGGTTGGTGCTTATAGAGATAATTCCGGTAAACCAATTATTTTCCCATCAGTTAAAAAAGCTGAAGAAATTTTATTGGGTAAAGAAACTGAAAAGGAATATACTGCCATTGTTGGTTCCAAAAATTTCCAATCAATTGTGAAAAATTTCATTTTCAACAATTCTAATAAAGATGCCAATGGTAAACAATTAATTGATGATGGTAGAATTGTTACTGCTCAAACCATCTCTGGTACTGGATCACTTAAACCTCWTATGGTGAAATTTTTGCTAAACATAGAGAACCAAATTTGGAAATTATTCGTGAGGTTGTTGATTCCAAACATATTGTTTTTGATGTGTTGGCACAATTCTTAATCAATCCAGACCCATGGGTTGCCATTGCTGCCGCTGAAGTTTATGTCAGACGTTCATACCGTGCTTATGATTTGGGTAAAATTGAATATCATGTTAATGACAGACTTCCTATTGTTGAATGGAAATTCAAGTTGGCTAATATGGGAGCCGCTGGTGTAAACGATGCTCAACAGGCTGCTGCTGCCGGTGGCGATGATTCGACATCTATGAAACATGCAGCTTCTGTGTCTGATTTGACCTTTGTTGTTGATTATTCATAAAAGYGAAATYCCAGGATACACTCTCCCCGATAATCCAAAGTTCACCCTTGGTAATTTGTTTGTAATAATTGGRGTCTTGTTGGTTTGTRTTTTAGCTGTTGTCTCTCTTTTGAGAAATATTAGTGAGTCRGCCTTGTTCAAGAAGAATGGGTATGAACCGTTGGATTCRGATCCTAGTGTCATGAACCWAAACTTYGAGCCTACAACATTGTCCTTTGAAGATATTAAATATGAGGTTACTGGTGGTCGACAAATTTTAAATGGAGTCTTTGGGTTTGTAAAACCAAGAGAATGTTTGGCTATAATGGGAGGTTCAGGTGCTGGTAAAACTACATTGTTGGATTTTAAACCTTTGGACCAATTRGCTAAGACTTTGACCACTGTTCCTGAATTGAATGAAATTATTGGGCAAGATTTAGTTGACGAATTTGTCAGTGGTATTAAACTACCAGCAGAAGTTGGAAGTCAAGATGATGTTAACAATAGAAAATTGTTGCAAAAAGTGTTTGGTAAATTAATGAACACTGATGATGACGTTATAAAACAACAAACAGCTAAATTACTTGAAAGAACAGACAGAGAACCTCAAGTGTTCAAGGATATTGATTCTAGATTACCGGAGTTAATACAAAGATTAAACAAACAATTTCCTAATGACATCGGATTATTTTGTGGATGTCTCTTATTGAACCACGTTGCTTGAAAAAATTATCAATTTCCGTGTTGGATAAACARAGATTGACTGAAAAATTCAATAAATTGGATAAATCCATTAAAGATAATTTGAAGGCTAAACAAAAAGAAGAAACCAAAAAGACTTTAGATGTGGTTAACAATTGGTTGAATGATAAAGAAAATGCTTCATCATTTTTGGTTGCTCACGTTCCAATTACTGCTAATGCCAAGGCAATCACTGAAGCCATTAATTTGATTAAAAAGCAAGATAAAACCAAATCAATTTATTTATTGACTGGTGAAACCGATAAAGTTGCTCATGGATGTTATGTTAGTGATGAAGCCATTGTCAAGGGTATTAATGCGAAAATCAAACCTTTTGAAGCACGAGCAATTAACTGGTCCACGGATCTTAATGCTGAGGTACATATTGAGCATTATATAAATATATTCAATTATGCACGATCATCTTGGGAGCCATTGGTTGAAAGTTGGCCAATAGCAGTTTACATGTCAAAATCCCGACACCCAAAGCCTCAATTATTAGTAGAGGTGATTTCTAGACAGGTAGCYCAAGTGACGCTTACATCCAAAGCRGTAGCATTGCTATCTCAAGTATCCGATTTGATTACTTCCGGAGAAAAATTAAAACCAAGAGGTGAAGATTACCCATACGTTATAGTGAATGAGACTGGTTTAGATTTGGAAGTTTGGAATGATGCAAAWGAATCCGAAACCAATGGAGTTCCAATTGTTTTACGTGCTGGTAAAGCTTTAGATGAAAGTAAAGTTGAAATTAGAATTCAATTTAAACCAGTCGCCAAGGGGATGTTTAAAGAAATTCAAAGAAATGAATTAGTTATTAGAGTACAACCAAATGAAGCCATTTATTTAAAAATTAATTCCAAAATCCCTGGAATTTCTACTGAAACTTCATTAACTGATTTAGATTTAACTTATGCTACTCGTTATTCTAAAGACTTTTGGATTCCTGAAGCTTATGAAGCATTAATTAGAGATTGYTATTTAGGTAATCATTCTAATTTTGTTAGAGATGATGAATTGGATGTTTCTTGGAAATTATTTACTCCATTATTGAAT

>Mi1_oral_27/02/2012:IND:41:14.8

AATTATTGAARTGGAATGATATTCCATTGGCTCCACCAGACAARATTTTGGGTATTTCTGAAGCTTATAACAATGATTCTAACCCTCAAAAAATCAATTTGGGGGTTGGTGCTTATAGAGATAATTCYGGTAAACCAATTATTTTCCCATCAGTTAAAAAAGCTGAAGAAATTTTATTGGGTAAAGAAACTGAAAAGGAATATACTGCCATTGTTGGTTCCAAAAATTTCCAATCAATTGTGAAAAATTTCATTTTCAACAATTCTAATAAAGATGCCAATGGTAAACAATTAATTGATGATGGTAGAATTGTTACTGCTCAAACCATCTCTGGTACTGGATCACTTAAACCTCTTATGGTGAAATTTTTGCTAAACATAGAGAACCAAATTTGGAAATTATTCGTGAGGTTGTTGATTCCAAACATATTGTTTTTGATGTGTTGGCACAATTCTTAATCAATCCAGACCCATGGGTTGCCATTGCTGCCGCTGAAGTTTATGTCAGACGTTCATACCGTGCTTATGATTTGGGTAAAATTGAATATCATGTTAATGACAGACTTCCTATTGTTGAATGGAAATTCAAGTTGGCTAATATGGGAGCCGCTGGTGTAAACGATGCTCAACAGGCTGCTGCTGCCGGTGGCGATGATTCGACATCTATGAAACATGCAGCTTCTGTGTCTGATTTGACCTTTGTTGTTGATTATTCATRAAAGCGAAATCCCAGGATACACTCTCCCCGATAATCCAAAGTTCACCCTTGGTAATTTGTTTGTAATAATTGGAGTCTTGTTGGTTTGTATTTTAGCTGTTGTCTCTCTTTTGAGAAATATTAGTGAGTCGGCCTTGTTCAAGAAGAATGGGTATGAACCGTTGGATTCGGATCCTAGTGTCATGAACCTAAACTTCGAGCCTACAACATTGTCCTTTGAAGATATTAAATATGAGGTTACTGGTGGTCGACAAATTTTAAATGGAGTCTTTGGGTTTGTAAAACCAAGAGAATGTTTGGCTATAATGGGAGGTTCAGGTGCTGGTAAAACTACATTGTTGGATTTTAAACCTTTGGACCAATTGGCTAAAACTTTGGCTACTGTTCCTGAATTGAATGAAATTATTGGTCAAGAATTAGTTGACGAATTTRTCAGTGGTATTAAACTACCAGCAGAAGTTGGAAGTCAAGATGATGTTAACAATAGAAAATTGTTGCAAAAAGTGTTTGGTAAATTAATGAACACTGATGATGACGTTATAAAACAACAAACAGCTAAATTACTTGAAAGAACAGACAGAGAACCTCAAGTGTTCAAGGATATTGATTCTAGATTACCGGAGTTAATACAAAGATTAAACAAACAATTTCCTAATGACATCGGATTATTTTGTGGATGTCTCTTATTGAACCACGTTGMTTGAAAAAATTATCAATTTCCGTGTTGGATAAACARAGATTGACTGAAAAATTCAATAAATTGGATAAATCCATCAAAGATAATTTGAAGGCTAAACAAAAAGAAGAAACCAAAAAAACTTTAGATGTGGTTAAYAATTGGTTGAATGATAAAGAAAATKCTTCATCATTTTTGGTTGCTCACGTTCCAATTACTGCTAATGCCAAGGCAATCACTGAAGCCATTAATTTGATTAAAAAGCAAGATAAAACCAAATCAATTTATTTATTGACTGGTGAAACCGATAAAGTTGCTCATGGATGTTATGTTAGTGATGAAGCCATTGCCAAGGGTATTAATGCGAAAATCAAACCTTTTGAAGCACGAGCAATTAACTGGTCCACGGATCTTAATGCTGAGGTACATATTGAGCATTATATAAATATATTCAATTATGCACGATCATCTTGGGAGCCATTGGTTGAAAGTTGGCCAATAGCAGTTTACATGTCAAAATCCCGACACCCAAAGCCTCAATTATTAGTAGAGGTGATTTCTAGACAGGTAGCYCAAGTGACGCTTACATCCAAAGCAGTAGCATTGCTATCTCAAGTATCCGATTTGATTACTTCCRGAGAAAAATTAAAACCAAGAGGTGAAGATTACCCATACGTTATAGTGAATGAGACTGGTTTAGATTTGGAAGTTTGGAATGATGCAAAWGAATCCGAAACCAATGGAGTTCCAATTGTTTTACGTGCTGGTAAAGCTTTAGATGAAAGTAAAGTTGAAATTAGAATTCAATTTAAACCAGTCGCCAAGGGGATGTTTAAAGAAATTCAAAGAAATGAATTAGTTATTAGAGTACAACCAAATGAAGCCATTTATTTAAAAATTAATTCCAAAATCCCTGGRATTTCTACTGAAACTTCATTAACTGATTTAGATTTAACTTATGCTACTCGTTATTCTAAAGATTTTTGGATTCCTGAAGCTTATGAAGCATTAATTAGAGATTGTTATTTAGGTAATCATTCTAATTTTGTTAGAGATGATGAATTGGATGTTTCTTGGAAATTATTTACTCCATTATTGAAT

>Mi1_oral_27/02/2012:IND:44:14.3

AATTATTGAARTGGAATGATATTCCATTGGCTCCACCAGACAARATTTTGGGTATTTCTGAAGCTTATAACAATGATTCTAACCCTCAAAAAATCAATTTGGGGGTTGGTGCTTATAGAGATAATTCYGGTAAACCAATTATTTTCCCATCAGTTAAAAAAGCTGAAGAAATTTTATTGGGTAAAGAAACTGAAAAGGAATATACTGCCATTGTTGGTTCCAAAAATTTCCAATCAATTGTGAAAAATTTCATTTTCAACAATTCTAATAAAGATGCCAATGGTAAACAATTAATTGATGATGGTAGAATTGTTACTGCTCAAACCATCTCTGGTACTGGATCACTTAAACCTCTTATGGTGAAATTTTTGCTAAACATAGAGAACCAAATTTGGAAATTATTCGTGAGGTTGTTGATTCCAAACATATTGTTTTTGATGTGTTGGCACAATTCTTAATCAATCCAGACCCATGGGTTGCCATTGCTGCCGCTGAAGTTTATGTCAGACGTTCATACCGTGCTTATGATTTGGGTAAAATTGAATATCATGTTAATGACAGACTTCCTATTGTTGAATGGAAATTCAAGTTGGCTAATATGGGAGCCGCTGGTGTAAACGATGCTCAACAGGCTGCTGCTGCCGGTGGCGATGATTCGACATCTATGAAACATGCAGCTTCTGTGTCTGATTTGACCTTTGTTGTTGATTATTCATRAAAGCGAAATCCCAGGATACACTCTCCCCGATAATCCAAAGTTCACCCTTGGTAATTTGTTTGTAATAATTGGAGTCTTGTTGGTTTGTATTTTAGCTGTTGTCTCTCTTTTGAGAAATATTAGTGAGTCGGCCTTGTTCAAGAAGAATGGGTATGAACCGTTGGATTCGGATCCTAGTGTCATGAACCTAAACTTCGAGCCTACAACATTGTCCTTTGAAGATATTAAATATGAGGTTACTGGTGGTCGACAAATTTTAAATGGAGTCTTTGGGTTTGTAAAACCAAGAGAATGTTTGGCTATAATGGGAGGTTCAGGTGCTGGTAAAACTACATTGTTGGATTTTAAACCTTTGGACCAATTGGCTAAAACTTTGGCTACTGTTCCTGAATTGAATGAAATTATTGGTCAAGAATTAGTTGACGAATTTRTCAGTGGTATTAAACTACCAGCAGAAGTTGGAAGTCAAGATGATGTTAACAATAGAAAATTGTTGCAAAAAGTGTTTGGTAAATTAATGAACACTGATGATGACGTTATAAAACAACAAACAGCTAAATTACTTGAAAGAACAGACAGAGAACCTCAAGTGTTCAAGGATATTGATTCTAGATTACCGGAGTTAATACAAAGATTAAACAAACAATTTCCTAATGACATCGGATTATTTTGTGGATGTCTCTTATTGAACCACGTTGMTTGAAAAAATTATCAATTTCCGTGTTGGATAAACARAGATTGACTGAAAAATTCAATAAATTGGATAAATCCATCAAAGATAATTTGAAGGCTAAACAAAAAGAAGAAACCAAAAAAACTTTAGATGTGGTTAAYAATTGGTTGAATGATAAAGAAAATKCTTCATCATTTTTGGTTGCTCACGTTCCAATTACTGCTAATGCCAAGGCAATCACTGAAGCCATTAATTTGATTAAAAAGCAAGATAAAACCAAATCAATTTATTTATTGACTGGTGAAACCGATAAAGTTGCTCATGGATGTTATGTTAGTGATGAAGCCATTGCCAAGGGTATTAATGCGAAAATCAAACCTTTTGAAGCACGAGCAATTAACTGGTCCACGGATCTTAATGCTGAGGTACATATTGAGCATTATATAAATATATTCAATTATGCACGATCATCTTGGGAGCCATTGGTTGAAAGTTGGCCAATAGCAGTTTACATGTCAAAATCCCGACACCCAAAGCCTCAATTATTAGTAGAGGTGATTTCTAGACAGGTAGCYCAAGTGACGCTTACATCCAAAGCAGTAGCATTGCTATCTCAAGTATCCGATTTGATTACTTCCRGAGAAAAATTAAAACCAAGAGGTGAAGATTACCCATACGTTATAGTGAATGAGACTGGTTTAGATTTGGAAGTTTGGAATGATGCAAAWGAATCCGAAACCAATGGAGTTCCAATTGTTTTACGTGCTGGTAAAGCTTTAGATGAAAGTAAAGTTGAAATTAGAATTCAATTTAAACCAGTCGCCAAGGGGATGTTTAAAGAAATTCAAAGAAATGAATTAGTTATTAGAGTACAACCAAATGAAGCCATTTATTTAAAAATTAATTCCAAAATCCCTGGRATTTCTACTGAAACTTCATTAACTGATTTAGATTTAACTTATGCTACTCGTTATTCTAAAGATTTTTGGATTCCTGAAGCTTATGAAGCATTAATTAGAGATTGTTATTTAGGTAATCATTCTAATTTTGTTAGAGATGATGAATTGGATGTTTCTTGGAAATTATTTACTCCATTATTGAAT

>Mi1_vaginal_27/02/2012

AATTATTGAAATGGAATGATATTCCATTGGCTCCACCAGACAAAATTTTGGGTATTTCTGAAGCTTATAACAATGATTCTAACCCTCAAAAAATCAATTTGGGGGTTGGTGCTTATAGAGATAATTCCGGTAAACCAATTATTTTCCCATCAGTTAAAAAAGCTGAAGAAATTTTATTGGGTAAAGAAACTGAAAAGGAATATACTGCCATTGTTGGTTCCAAAAATTTCCAATCAATTGTGAAAAATTTCATTTTCAACAATTCTAATAAAGATGCCAATGGTAAACAATTAATTGATGATGGTAGAATTGTTACTGCTCAAACCATCTCTGGTACTGGATCACTTAAACCTCTTATGGTGAAATTTTTGCTAAACATAGAGAACCAAATTTGGAAATTATTCGTGAGGTTGTTGATTCCAAACATATTGTTTTTGATGTGTTGGCACAATTCTTAATCAATCCAGACCCATGGGTTGCCATTGCTGCCGCTGAAGTTTATGTCAGACGTTCATACCGTGCTTATGATTTGGGTAAAATTGAATATCATGTTAATGACAGACTTCCTATTGTTGAATGGAAATTCAAGTTGGCTAATATGGGAGCYGCTGGTGTAAACGATGCTCAACAGGCTGCTGCTGCCGGTGGCGATGATTCGACATCTATGAAACATGCAGCTTCTGTGTCTGATTTGACCTTTGTTGTTGATTATTCATAAAAGYGAAATYCCAGGATACACTCTCCCCGATAATCCAAAGTTCACCCTTGGTAATTTGTTTGTAATAATTGGRGTCTTGTTGGTTTGTGTTTTAGCTGTTGTCTCTCTTTTGAGAAATATTAGTGAGTCRGCCTTGTTCAAGAAGAATGGGTATGAACCGTTGGATTCRGATCCTAGTGTCATGAACCWAAACTTYGAGCCTACAACATTGTCCTTTGAAGATATTAAATATGAGGTTACTGGTGGTCGACAAATTTTAAATGGAGTCTTTGGGTTTGTAAAACCAAGAGAATGTTTGGCTATAATGGGAGGTTCAGGTGCTGGTAAAACTACATTGTTGGATTTTAAACCTTTGGACCAATTGGCTAAGACTTTGACCACTGTTCCTGAATTGAATGAAATTATTGGGCAAGATTTAGTTGACGAATTTGTCAGTGGTATTAAACTACCAGCAGAAGTTGGAAGTCAAGATGATGTTAACAATAGAAAATTGTTGCAAAAAGTGTTTGGTAAATTAATGAACACTGATGATGACGTTATAAAACAACAAACAGCTAAATTACTTGAAAGAACAGAMAGRGAACCTCAAGTGTTCAAGGATATTGATTCTAGATTACCRGAGTTAATACAARGATTAAACAAACAATTTCCTAATGACATCGGATTATTTTGTGGATGTCTCTTATTGAACCACGTTGMTTGAAAAAATTATCAATTTCCGTGTTGGATAAACARAGATTGACTGAAAAATTCAATAAATTGGATAAATCCATYAAAGATAATTTGAAGGCTAAACAAAAAGAAGAAACCAAAAARACTTTAGATGTGGTTAAYAATTGGTTGAATGATAAAGAAAATGCTTCATCATTTTTGGTTGCTCACGTTCCAATTACTGCTAATGCCAAGGCAATCACTGAAGCCATTAATTTGATTAAAAAGCAAGATAAAACCAAATCAATTTATTTATTGACTGGTGAAACCGATAAAGTTGCTCATGGATGTTATGTTAGTGATGAAGCCATTGYCAAGGGTATTAATGCGAAAATCAAACCTTTTGAAGCACGAGCAATTAACTGGTCMACGGATCTTAATGCTGAGGTACATATTGAGCATTATATAAATATATTCAATTATGCACGATCATCTTGGGAGCCATTGGTTGAAAGTTGGCCAATAGCAGTTTACATGTCAAAATCCCGACACCCAAAGCCTCAATTATTAGTAGAGGTGATTTCTAGACAGGTAGCTCAAGTGACGCTTACATCCAAAGCRGTAGCATTGCTATCTCAAGTATCCGATTTGATTACTTCCGRAGAAAAATTAAAACCAAGAGGTGAAGATTACCCATACRTTATAGTGAATGAGACTGGTTTAGATTTGGAAGTTTGGAATGATGCAAAWGAATCCGAAACCAATGGAGTTCCAATTGTTTTACGTGCTGGTAAAGCTTTAGATGAAAGTAAAGTTGAAATTAGAATTCAATTTAAACCAGTCGCCAAGGGGATGTTTAAAGAAATTCAAAGAAATGAATTAGTTATTAGAGTACAACCAAATGAAGCCATTTATTTAAAAATTAATTCCAAAATCCCTGGAATTTCTACTGAAACTTCATTAACTGATTTAGATTTAACTTATGCTACTCGTTATTCTAAAGAYTTTTGGATTCCTGAAGCTTATGAAGCATTAATTAGAGATTGTTATTTAGGTAATCATTCTAATTTTGTTAGAGATGATGAATTGGATGTTTCTTGGAAATTATTTACTCCATTATTGAAT

>Mi2_rectal_6/04/2012

AATTATTGAAATGGAATGATATTCCATTGGCTCCACCAGACAARATTTTGGGTATTTCTGAAGCTTATAACAATGATTCTAACCCTCAAAAARTCAATTTGGGGGTTGGTGCTTATAGAGATAATTCYGGTAAACCAATTATTTTCCCATCAGTTAAAAAAGCTGAAGAAATTTTATTGGGTAAAGAAACTGAAAAGGAATATACTGCCATTGTTGGTTCCAAAAATTTCCAATCAATTGTGAAAAATTTCATTTTCAACAATTCTAATAAAGATGCCAATGGTAAACAATTAATTGATGATGGTAGAATTGTTACTGCTCAAACCATCTCTGGTACTGGATCACTTAAACCTCTTATGGTGAAATTTTTGCTAAACATAGAGAACCAAATTTGGAAATTATTCGTGAGGTTGTTGATTCCAAACATATTGTTTTTGATGTGTTGGCACAATTCTTAATCAATCCAGACCCATGGGTTGCCATTGCTGCCGCTGAAGTTTATGTCAGACGTTCATACCGTGCTTATGATTTGGGTAAAATTGAATATCATGTTAATGACAGACTTCCTATTGTTGAATGGAAATTCAAGTTGGCTAATATGGGAGCCGCTGGTGTAAACGATGCTCAACAGGCTGCTGCTGCCGGTGGCGATGATTCGACATCTATGAAACATGCAGCTTCTGTGTCTGATTTGACCTTTGTTGTTGATTATTCATAAAAGYGAAATYCCAGGATACACTCTCCCCGATAATCCAAAGTTCACCCTTGGTAATTTGTTTGTAATAATTGGRGTCTTGTTGGTTTGTRTTTTAGCTGTTGTCTCTCTTTTGAGAAATATTAGTGAGTCRGCCTTGTTCAAGAAGAATGGGTATGAACCGTTGGATTCRGATCCTAGTGTCATGAACCWAAACTTYGAGCCTACAACATTGTCCTTTGAAGATATTAAATATGAGGTTACTGGTGGTCGACAAATTTTAAATGGAGTCTTTGGGTTTGTAAAACCAAGAGAATGTTTGGCTATAATGGGAGGTTCAGGTGCTGGTAAAACTACATTGTTGGATTTTAAACCTTTGGACCAATTGGCTAARACTTTGRCYACTGTTCCTGAATTGAATGAAATTATTGGKCAAGAWTTAGTTGACGAATTTGTCAGTGGTATTAAACTACCAGCAGAAGTTGGAAGTCAAGATGATGTTAACAATAGAAAATTGTTGCAAAAAGTGTTTGGTAAATTAATGAACACTGATGATGACGTTATAAAACAACAAACAGCTAAATTACTTGAAAGAACAGAMAGRGAACCTCAAGTGTTCAAGGATATTGATTCTAGATTACCRGAGTTAATACAARGATTAAACAAACAATTTCCTAATGACATCGGATTATTTTGTGGATGTCTCTTATTGAACCACGTTGMTTGAAAAAATTATCAATTTCCGTGTTGGATAAACARAGATTGACTGAAAAATTCAATAAATTGGATAAATCCATYAAAGATAATTTGAAGGCTAAACAAAAAGAAGAAACCAAAAARACTTTAGATGTGGTTAAYAATTGGTTGAATGATAAAGAAAATGCTTCATCATTTTTGGTTGCTCACGTTCCAATTACTGCTAATGCCAAGGCAATCACTGAAGCCATTAATTTGATTAAAAAGCAAGATAAAACCAAATCAATTTATTTATTGACTGGTGAAACCGATAAAGTTGCTCATGGATGTTATGTTAGTGATGAAGCCATTGYCAAGGGTATTAATGCGAAAATCAAACCTTTTGAAGCACGAGCAATTAACTGGTCCACGGATCTTAATGCTGAGGTACATATTGAGCATTATATAAATATATTCAATTATGCACGATCATCTTGGGAGCCATTGGTTGAAAGTTGGCCAATAGCAGTTTACATGTCAAAATCCCGACACCCAAAGCCTCAATTATTAGTAGAGGTGATTTCTAGACAGGTAGCTCAAGTGACGCTTACATCCAAAGCGGTAGCATTGCTATCTCAAGTATCCGATTTGATTACTTCCGAAGAAAAATTAAAACCAAGAGGTGAAGATTACCCATACGTTATAGTGAATGAGACTGGTTTAGATTTGGAAGTTTGGAATGATGCAAATGAATCCGAAACCAATGGAGTTCCAATTGTTTTACGTGCTGGTAAAGCTTTAGATGAAAGTAAAGTTGAAATTAGAATTCAATTTAAACCAGTCGCCAAGGGGATGTTTAAAGAAATTCAAAGAAATGAATTAGTTATTAGAGTACAACCAAATGAAGCCATTTATTTAAAAATTAATTCCAAAATCCCTGGAATTTCTACTGAAACTTCATTAACTGATTTAGATTTAACTTATGCTACTCGTTATTCTAAAGAYTTTTGGATTCCTGAAGCTTATGAAGCATTAATTAGAGATTGYTATTTAGGTAATCATTCTAATTTTGTTAGAGATGATGAATTGGATGTTTCTTGGAAATTATTTACTCCATTATTGAAT

>Mi2_rectal_12/03/2012

AATTATTGAAATGGAATGATATTCCATTGGCTCCACCAGACAARATTTTGGGTATTTCTGAAGCTTATAACAATGATTCTAACCCTCAAAAARTCAATTTGGGGGTTGGTGCTTATAGAGATAATTCYGGTAAACCAATTATTTTCCCATCAGTTAAAAAAGCTGAAGAAATTTTATTGGGTAAAGAAACTGAAAAGGAATATACTGCCATTGTTGGTTCCAAAAATTTCCAATCAATTGTGAAAAATTTCATTTTCAACAATTCTAATAAAGATGCCAATGGTAAACAATTAATTGATGATGGTAGAATTGTTACTGCTCAAACCATCTCTGGTACTGGATCACTTAAACCTCTTATGGTGAAATTTTTGCTAAACATAGAGAACCAAATTTGGAAATTATTCGTGAGGTTGTTGATTCCAAACATATTGTTTTTGATGTGTTGGCACAATTCTTAATCAATCCAGACCCATGGGTTGCCATTGCTGCCGCTGAAGTTTATGTCAGACGTTCATACCGTGCTTATGATTTGGGTAAAATTGAATATCATGTTAATGACAGACTTCCTATTGTTGAATGGAAATTCAAGTTGGCTAATATGGGAGCCGCTGGTGTAAACGATGCTCAACAGGCTGCTGCTGCCGGTGGCGATGATTCGACATCTATGAAACATGCAGCTTCTGTGTCTGATTTGACCTTTGTTGTTGATTATTCATAAAAGYGAAATYCCAGGATACACTCTCCCCGATAATCCAAAGTTCACCCTTGGTAATTTGTTTGTAATAATTGGRGTCTTGTTGGTTTGTRTTTTAGCTGTTGTCTCTCTTTTGAGAAATATTAGTGAGTCRGCCTTGTTCAAGAAGAATGGGTATGAACCGTTGGATTCRGATCCTAGTGTCATGAACCWAAACTTYGAGCCTACAACATTGTCCTTTGAAGATATTAAATATGAGGTTACTGGTGGTCGACAAATTTTAAATGGAGTCTTTGGGTTTGTAAAACCAAGAGAATGTTTGGCTATAATGGGAGGTTCAGGTGCTGGTAAAACTACATTGTTGGATTTTAAACCTTTGGACCAATTGGCTAARACTTTGRCYACTGTTCCTGAATTGAATGAAATTATTGGKCAAGAWTTAGTTGACGAATTTGTCAGTGGTATTAAACTACCAGCAGAAGTTGGAAGTCAAGATGATGTTAACAATAGAAAATTGTTGCAAAAAGTGTTTGGTAAATTAATGAACACTGATGATGACGTTATAAAACAACAAACAGCTAAATTACTTGAAAGAACAGAMAGRGAACCTCAAGTGTTCAAGGATATTGATTCTAGATTACCRGAGTTAATACAARGATTAAACAAACAATTTCCTAATGACATCGGATTATTTTGTGGATGTCTCTTATTGAACCACGTTGMTTGAAAAAATTATCAATTTCCGTGTTGGATAAACARAGATTGACTGAAAAATTCAATAAATTGGATAAATCCATYAAAGATAATTTGAAGGCTAAACAAAAAGAAGAAACCAAAAARACTTTAGATGTGGTTAAYAATTGGTTGAATGATAAAGAAAATGCTTCATCATTTTTGGTTGCTCACGTTCCAATTACTGCTAATGCCAAGGCAATCACTGAAGCCATTAATTTGATTAAAAAGCAAGATAAAACCAAATCAATTTATTTATTGACTGGTGAAACCGATAAAGTTGCTCATGGATGTTATGTTAGTGATGAAGCCATTGYCAAGGGTATTAATGCGAAAATCAAACCTTTTGAAGCACGAGCAATTAACTGGTCCACGGATCTTAATGCTGAGGTACATATTGAGCATTATATAAATATATTCAATTATGCACGATCATCTTGGGAGCCATTGGTTGAAAGTTGGCCAATAGCAGTTTACATGTCAAAATCCCGACACCCAAAGCCTCAATTATTAGTAGAGGTGATTTCTAGACAGGTAGCTCAAGTGACGCTTACATCCAAAGCGGTAGCATTGCTATCTCAAGTATCCGATTTGATTACTTCCGAAGAAAAATTAAAACCAAGAGGTGAAGATTACCCATACGTTATAGTGAATGAGACTGGTTTAGATTTGGAAGTTTGGAATGATGCAAATGAATCCGAAACCAATGGAGTTCCAATTGTTTTACGTGCTGGTAAAGCTTTAGATGAAAGTAAAGTTGAAATTAGAATTCAATTTAAACCAGTCGCCAAGGGGATGTTTAAAGAAATTCAAAGAAATGAATTAGTTATTAGAGTACAACCAAATGAAGCCATTTATTTAAAAATTAATTCCAAAATCCCTGGAATTTCTACTGAAACTTCATTAACTGATTTAGATTTAACTTATGCTACTCGTTATTCTAAAGAYTTTTGGATTCCTGAAGCTTATGAAGCATTAATTAGAGATTGYTATTTAGGTAATCATTCTAATTTTGTTAGAGATGATGAATTGGATGTTTCTTGGAAATTATTTACTCCATTATTGAAT

>Mi2_vaginal_12/03/2011

AATTATTGAAATGGAATGATATTCCATTGGCTCCACCAGACAAAATTTTGGGTATTTCTGAAGCTTATAACAATGATTCTAACCCTCAAAAAATCAATTTGGGGGTTGGTGCTTATAGAGATAATTCCGGTAAACCAATTATTTTCCCATCAGTTAAAAAAGCTGAAGAAATTTTATTGGGTAAAGAAACTGAAAAGGAATATACTGCCATTGTTGGTTCCAAAAATTTCCAATCAATTGTGAAAAATTTCATTTTCAACAATTCTAATAAAGATGCCAATGGTAAACAATTAATTGATGATGGTAGAATTGTTACTGCTCAAACCATCTCTGGTACTGGATCACTTAAACCTCTTATGGTGAAATTTTTGCTAAACATAGAGAACCAAATTTGGAAATTATTCGTGAGGTTGTTGATTCCAAACATATTGTTTTTGATGTGTTGGCACAATTCTTAATCAATCCAGACCCATGGGTTGCCATTGCTGCCGCTGAAGTTTATGTCAGACGTTCATACCGTGCTTATGATTTGGGTAAAATTGAATATCATGTTAATGACAGACTTCCTATTGTTGAATGGAAATTCAAGTTGGCTAATATGGGAGCYGCTGGTGTAAACGATGCTCAACAGGCTGCTGCTGCCGGTGGCGATGATTCGACATCTATGAAACATGCAGCTTCTGTGTCTGATTTGACCTTTGTTGTTGATTATTCATAAAAGYGAAATYCCAGGATACACTCTCCCCGATAATCCAAAGTTCACCCTTGGTAATTTGTTTGTAATAATTGGRGTCTTGTTGGTTTGTRTTTTAGCTGTTGTCTCTCTTTTGAGAAATATTAGTGAGTCRGCCTTGTTCAAGAAGAATGGGTATGAACCGTTGGATTCRGATCCTAGTGTCATGAACCWAAACTTYGAGCCTACAACATTGTCCTTTGAAGATATTAAATATGAGGTTACTGGTGGTCGACAAATTTTAAATGGAGTCTTTGGGTTTGTAAAACCAAGAGAATGTTTGGCTATAATGGGAGGTTCAGGTGCTGGTAAAACTACATTGTTGGATTTTAAACCTTTGGACCAATTRGCTAAGACTTTGACCACTGTTCCTGAATTGAATGAAATTATTGGGCAAGATTTAGTTGACGAATTTGTCAGTGGTATTAAACTACCAGCAGAAGTTGGAAGTCAAGATGATGTTAACAATAGAAAATTGTTGCAAAAAGTGTTTGGTAAATTAATGAACACTGATGATGACGTTATAAAACAACAAACAGCTAAATTACTTGAAAGAACAGACAGAGAACCTCAAGTGTTCAAGGATATTGATTCTAGATTACCGGAGTTAATACAAAGATTAAACAAACAATTTCCTAATGACATCGGATTATTTTGTGGATGTCTCTTATTGAACCACGTTGMTTGAAAAAATTATCAATTTCCGTGTTGGATAAACARAGATTGACTGAAAAATTCAATAAATTGGATAAATCCATYAAAGATAATTTGAAGGCTAAACAAAAAGAAGAAACCAAAAARACTTTAGATGTGGTTAAYAATTGGTTGAATGATAAAGAAAATGCTTCATCATTTTTGGTTGCTCACGTTCCAATTACTGCTAATGCCAAGGCAATCACTGAAGCCATTAATTTGATTAAAAAGCAAGATAAAACCAAATCAATTTATTTATTGACTGGTGAAACCGATAAAGTTGCTCATGGATGTTATGTTAGTGATGAAGCCATTGYCAAGGGTATTAATGCGAAAATCAAACCTTTTGAAGCACGAGCAATTAACTGGTCCACGGATCTTAATGCTGAGGTACATATTGAGCATTATATAAATATATTCAATTATGCACGATCATCTTGGGAGCCATTGGTTGAAAGTTGGCCAATAGCAGTTTACATGTCAAAATCCCGACACCCAAAGCCTCAATTATTAGTAGAGGTGATTTCTAGACAGRTAGCTCAAGTGACGCTTACATCCAAAGCGGTAGCATTGCTATCTCAAGTATCCGATTTGATTACTTCCGRAGAAAAATTAAAACCAAGAGGTGAAGATTACCCATACGTTATAGTGAATGARACTGGTTTAGATTTGGAAGTTTGGAATGATGCAAAWGAATCCGAAACCAATGGAGTTCCAATTGTTTTACGTGCTGGTAAAGCTTTAGATGAAAGTAAAGTTGAAATTAGAATTCAATTTAAACCAGTCGCCAAGGGGATGTTTAAAGAAATTCAAAGAAATGAATTAGTTATTAGAGTACAACCAAATGAAGCCATTTATTTAAAAATTAATTCCAAAATCCCTGGAATTTCTACTGAAACTTCATTAACTGATTTAGATTTAACTTATGCTACTCGTTATTCTAAAGAYTTTTGGATTCCTGAAGCTTATGAAGCATTAATTAGAGATTGTTATTTAGGTAATCATTCTAATTTTGTTAGAGATGATGAATTGGATGTTTCTTGGAAATTATTTACTCCATTATTGAAT

>Mi2_vaginal_12/03/2011:IND:21:6.6

AATTATTGAAATGGAATGATATTCCATTGGCCCCACCAGACAAGATTTTGGGTATTTCTGAAGCTTATAACAATGATTCTAACCCTCAAAAAATCAATTTGGGGGTTGGTGCTTATAGAGATAATTCTGGTAAACCAATTATTTTCCCATCAGTTAAAAAAGCTGAAGAAATTTTATTGGGTAAAGAAACTGAAAAGGAATATACTGCCATTGTTGGTTCCAAAAATTTCCAATCAATTGTGAAAAATTTCATTTTCAACAATTCTAATAAAGATGCCAATGGTAAACAATTAATTGATGATGGTAGAATTGTTACTGCTCAAACCATCTCTGGTACTGGATCACTTAAACCTCTTATGGTGAAATTTTTGCTAAACATAGAGAACCAAATTTGGAAATTATTCGTGAGGTTGTTGATTCCAAACATATTGTTTTTGATGTGTTGGCACAATTCTTAATCAATCCAGACCCATGGGTTGCCATTGCTGCCGCTGAAGTTTATGTCAGACGTTCATACCGTGCTTATGATTTGGGTAAAATTGAATATCATGTTAATGACAGACTTCCTATTGTTGAATGGAAATTCAAGTTGGCTAATATGGGAGCYGCTGGTGTAAACGATGCTCAACAGGCTGCTGCTGCCGGTGGCGATGATTCGACATCTATGAAACATGCAGCTTCTGTGTCTGATTTGACCTTTGTTGTTGATTATTCATAAAAGCGAAATCCCAGGATACACTCTCCCCGATAATCCAAAGTTCACCCTTGGTAATTTGTTTGTAATAATTGGAGTCTTGTTGGTTTGTATTTTAGCTGTTGTCTCTCTTTTGAGAAATATTAGTGAGTCGGCCTTGTTCAAGAAGAATGGGTATGAACCGTTGGATTCGGATCCTAGTGTCATGAACCTAAACTTCGAGCCTACAACATTGTCCTTTGAAGATATTAAATATGAGGTTACTGGTGGTCGACAAATTTTAAATGGAGTCTTTGGGTTTGTAAAACCAAGAGAATGTTTGGCTATAATGGGAGGTTCAGGTGCTGGTAAAACTACATTGTTGGATTTTAAACCTTTGGACCAATTGGCTAARACTTTGRCYACTGTTCCTGAATTGAATGAAATTATTGGKCAAGAWTTAGTTGACGAATTTRTCAGTGGTATTAAACTACCAGCAGAAGTTGGAAGTCAAGATGATGTTAACAATAGAAAATTGTTGCAAAAAGTGTTTGGTAAATTAATGAACACTGATGATGACGTTATAAAACAACAAACAGCTAAATTACTTGAAAGAACAGAMAGRGAACCTCAAGTGTTCAAGGATATTGATTCTAGATTACCRGAGTTAATACAARGATTAAACAAACAATTTCCTAATGACATCGGATTATTTTGTGGATGTCTCTTATTGAACCACGTTGCTTGAAAAAATTATCAATTTCCGTGTTGGATAAACAGAGATTGACTGAAAAATTCAATAAATTGGATAAATCCATTAAAGATAATTTGAAGGCTAAACAAAAAGAAGAAACCAAAAAGACTTTAGATGTGGTTAACAATTGGTTGAATGATAAAGAAAATGCTTCATCATTTTTGGTTGCTCACGTTCCAATTACTGCTAATGCCAAGGCAATCACTGAAGCCATTAATTTGATTAAAAAGCAAGATAAAACCAAATCAATTTATTTATTGACTGGTGAAACCGATAAAGTTGCTCATGGATGTTATGTTAGTGATGAAGCCATTGTCAAGGGTATTAATGCGAAAATCAAACCTTTTGAAGCACGAGCAATTAACTGGTCCACGGATCTTAATGCTGAGGTACATATTGAGCATTATATAAATATATTCAATTATGCACGATCATCTTGGGAGCCATTGGTTGAAAGTTGGCCAATAGCAGTTTACATGTCAAAATCCCGACACCCAAAGCCTCAATTATTAGTAGAGGTGATTTCTAGACAGRTAGCCCAAGTGACGCTTACATCCAAAGCAGTAGCATTGCTATCTCAAGTATCCGATTTGATTACTTCCGRAGAAAAATTAAAACCAAGAGGTGAAGATTACCCATACGTTATAGTGAATGARACTGGTTTAGATTTGGAAGTTTGGAATGATGCAAAAGAATCCGAAACCAATGGAGTTCCAATTGTTTTACGTGCTGGTAAAGCTTTAGATGAAAGTAAAGTTGAAATTAGAATTCAATTTAAACCAGTCGCCAAGGGGATGTTTAAAGAAATTCAAAGAAATGAATTAGTTATTAGAGTACAACCAAATGAAGCCATTTATTTAAAAATTAATTCCAAAATCCCTGGAATTTCTACTGAAACTTCATTAACTGATTTAGATTTAACTTATGCTACTCGTTATTCTAAAGAYTTTTGGATTCCTGAAGCTTATGAAGCATTAATTAGAGATTGTTATTTAGGTAATCATTCTAATTTTGTTAGAGATGATGAATTGGATGTTTCTTGGAAATTATTTACTCCATTATTGAAT

>Mi2_vaginal_12/03/2011:IND:22:13.8

AATTATTGAAATGGAATGATATTCCATTGGCCCCACCAGACAAGATTTTGGGTATTTCTGAAGCTTATAACAATGATTCTAACCCTCAAAAAATCAATTTGGGGGTTGGTGCTTATAGAGATAATTCTGGTAAACCAATTATTTTCCCATCAGTTAAAAAAGCTGAAGAAATTTTATTGGGTAAAGAAACTGAAAAGGAATATACTGCCATTGTTGGTTCCAAAAATTTCCAATCAATTGTGAAAAATTTCATTTTCAACAATTCTAATAAAGATGCCAATGGTAAACAATTAATTGATGATGGTAGAATTGTTACTGCTCAAACCATCTCTGGTACTGGATCACTTAAACCTCTTATGGTGAAATTTTTGCTAAACATAGAGAACCAAATTTGGAAATTATTCGTGAGGTTGTTGATTCCAAACATATTGTTTTTGATGTGTTGGCACAATTCTTAATCAATCCAGACCCATGGGTTGCCATTGCTGCCGCTGAAGTTTATGTCAGACGTTCATACCGTGCTTATGATTTGGGTAAAATTGAATATCATGTTAATGACAGACTTCCTATTGTTGAATGGAAATTCAAGTTGGCTAATATGGGAGCYGCTGGTGTAAACGATGCTCAACAGGCTGCTGCTGCCGGTGGCGATGATTCGACATCTATGAAACATGCAGCTTCTGTGTCTGATTTGACCTTTGTTGTTGATTATTCATAAAAGCGAAATCCCAGGATACACTCTCCCCGATAATCCAAAGTTCACCCTTGGTAATTTGTTTGTAATAATTGGAGTCTTGTTGGTTTGTATTTTAGCTGTTGTCTCTCTTTTGAGAAATATTAGTGAGTCGGCCTTGTTCAAGAAGAATGGGTATGAACCGTTGGATTCGGATCCTAGTGTCATGAACCTAAACTTCGAGCCTACAACATTGTCCTTTGAAGATATTAAATATGAGGTTACTGGTGGTCGACAAATTTTAAATGGAGTCTTTGGGTTTGTAAAACCAAGAGAATGTTTGGCTATAATGGGAGGTTCAGGTGCTGGTAAAACTACATTGTTGGATTTTAAACCTTTGGACCAATTGGCTAARACTTTGRCYACTGTTCCTGAATTGAATGAAATTATTGGKCAAGAWTTAGTTGACGAATTTRTCAGTGGTATTAAACTACCAGCAGAAGTTGGAAGTCAAGATGATGTTAACAATAGAAAATTGTTGCAAAAAGTGTTTGGTAAATTAATGAACACTGATGATGACGTTATAAAACAACAAACAGCTAAATTACTTGAAAGAACAGAMAGRGAACCTCAAGTGTTCAAGGATATTGATTCTAGATTACCRGAGTTAATACAARGATTAAACAAACAATTTCCTAATGACATCGGATTATTTTGTGGATGTCTCTTATTGAACCACGTTGCTTGAAAAAATTATCAATTTCCGTGTTGGATAAACAGAGATTGACTGAAAAATTCAATAAATTGGATAAATCCATTAAAGATAATTTGAAGGCTAAACAAAAAGAAGAAACCAAAAAGACTTTAGATGTGGTTAACAATTGGTTGAATGATAAAGAAAATGCTTCATCATTTTTGGTTGCTCACGTTCCAATTACTGCTAATGCCAAGGCAATCACTGAAGCCATTAATTTGATTAAAAAGCAAGATAAAACCAAATCAATTTATTTATTGACTGGTGAAACCGATAAAGTTGCTCATGGATGTTATGTTAGTGATGAAGCCATTGTCAAGGGTATTAATGCGAAAATCAAACCTTTTGAAGCACGAGCAATTAACTGGTCCACGGATCTTAATGCTGAGGTACATATTGAGCATTATATAAATATATTCAATTATGCACGATCATCTTGGGAGCCATTGGTTGAAAGTTGGCCAATAGCAGTTTACATGTCAAAATCCCGACACCCAAAGCCTCAATTATTAGTAGAGGTGATTTCTAGACAGRTAGCCCAAGTGACGCTTACATCCAAAGCAGTAGCATTGCTATCTCAAGTATCCGATTTGATTACTTCCGRAGAAAAATTAAAACCAAGAGGTGAAGATTACCCATACGTTATAGTGAATGAGACTGGTTTAGATTTGGAAGTTTGGAATGATGCAAAAGAATCCGAAACCAATGGAGTTCCAATTGTTTTACGTGCTGGTAAAGCTTTAGATGAAAGTAAAGTTGAAATTAGAATTCAATTTAAACCAGTCGCCAAGGGGATGTTTAAAGAAATTCAAAGAAATGAATTAGTTATTAGAGTACAACCAAATGAAGCCATTTATTTAAAAATTAATTCCAAAATCCCTGGAATTTCTACTGAAACTTCATTAACTGATTTAGATTTAACTTATGCTACTCGTTATTCTAAAGAYTTTTGGATTCCTGAAGCTTATGAAGCATTAATTAGAGATTGTTATTTAGGTAATCATTCTAATTTTGTTAGAGATGATGAATTGGATGTTTCTTGGAAATTATTTACTCCATTATTGAAT

>Mi3_pharynx_15/03/2012

AATTATTGAAGTGGAATGATATTCCATTGGCTCCACCAGACAAAATTTTGGGTATTTCTGAAGCTTATAACAATGATTCTAACCCTCAAAAAATCAATTTGGGGGTTGGTGCTTATAGAGATAATTCCGGTAAACCAATTATTTTCCCATCAGTTAAAAAAGCTGAAGAAATTTTATTGGGTAAAGAAACTGAAAAGGAATATACTGCCATTGTTGGTTCCAAAAATTTCCAATCAATTGTGAAAAATTTCATTTTCAACAATTCTAATAAAGATGCCAATGGTAAACAATTAATTGATGATGGTAGAATTGTTACTGCTCAAACCATCTCTGGTACTGGATCACTTAAACCTCWTATGGTGAAATTTTTGCTAAACATAGAGAACCAAATTTGGAAATTATTCGTGAGGTTGTTGATTCCAAACATATTGTTTTTGATGTGTTGGCACAATTCTTAATCAATCCAGACCCATGGGTTGCCATTGCTGCCGCTGAAGTTTATGTCAGACGTTCATACCGTGCTTATGATTTGGGTAAAATTGAATATCATGTTAATGACAGACTTCCTATTGTTGAATGGAAATTCAAGTTGGCTAATATGGGAGCCGCTGGTGTAAACGATGCTCAACAGGCTGCTGCTGCCGGTGGCGATGATTCGACATCTATGAAACATGCAGCTTCTGTGTCTGATTTGACCTTTGTTGTTGATTATTCATAAAAGYGAAATYCCAGGATACACTCTCCCCGATAATCCAAAGTTCACCCTTGGTAATTTGTTTGTAATAATTGGRGTCTTGTTGGTTTGTRTTTTAGCTGTTGTCTCTCTTTTGAGAAATATTAGTGAGTCRGCCTTGTTCAAGAAGAATGGGTATGAACCGTTGGATTCRGATCCTAGTGTCATGAACCWAAACTTYGAGCCTACAACATTGTCCTTTGAAGATATTAAATATGAGGTTACTGGTGGTCGACAAATTTTAAATGGAGTCTTTGGGTTTGTAAAACCAAGAGAATGTTTGGCTATAATGGGAGGTTCAGGTGCTGGTAAAACTACATTGTTGGATTTTAAACCTTTGGACCAATTRGCTAAGACTTTGACCACTGTTCCTGAATTGAATGAAATTATTGGGCAAGATTTAGTTGACGAATTTGTCAGTGGTATTAAACTACCAGCAGAAGTTGGAAGTCAAGATGATGTTAACAATAGAAAATTGTTGCAAAAAGTGTTTGGTAAATTAATGAACACTGATGATGACGTTATAAAACAACAAACAGCTAAATTACTTGAAAGAACAGACAGAGAACCTCAAGTGTTCAAGGATATTGATTCTAGATTACCGGAGTTAATACAAAGATTAAACAAACAATTTCCTAATGACATCGGATTATTTTGTGGATGTCTCTTATTGAACCACGTTGCTTGAAAAAATTATCAATTTCCGTGTTGGATAAACARAGATTGACTGAAAAATTCAATAAATTGGATAAATCCATTAAAGATAATTTGAAGGCTAAACAAAAAGAAGAAACCAAAAAGACTTTAGATGTGGTTAACAATTGGTTGAATGATAAAGAAAATGCTTCATCATTTTTGGTTGCTCACGTTCCAATTACTGCTAATGCCAAGGCAATCACTGAAGCCATTAATTTGATTAAAAAGCAAGATAAAACCAAATCAATTTATTTATTGACTGGTGAAACCGATAAAGTTGCTCATGGATGTTATGTTAGTGATGAAGCCATTGTCAAGGGTATTAATGCGAAAATCAAACCTTTTGAAGCACGAGCAATTAACTGGTCCACGGATCTTAATGCTGAGGTACATATTGAGCATTATATAAATATATTCAATTATGCACGATCATCTTGGGAGCCATTGGTTGAAAGTTGGCCAATAGCAGTTTACATGTCAAAATCCCGACACCCAAAGCCTCAATTATTAGTAGAGGTGATTTCTAGACAGGTAGCYCAAGTGACGCTTACATCCAAAGCRGTAGCATTGCTATCTCAAGTATCCGATTTGATTACTTCCGGAGAAAAATTAAAACCAAGAGGTGAAGATTACCCATACGTTATAGTGAATGAGACTGGTTTAGATTTGGAAGTTTGGAATGATGCAAAWGAATCCGAAACCAATGGAGTTCCAATTGTTTTACGTGCTGGTAAAGCTTTAGATGAAAGTAAAGTTGAAATTAGAATTCAATTTAAACCAGTCGCCAAGGGGATGTTTAAAGAAATTCAAAGAAATGAATTAGTTATTAGAGTACAACCAAATGAAGCCATTTATTTAAAAATTAATTCCAAAATCCCTGGAATTTCTACTGAAACTTCATTAACTGATTTAGATTTAACTTATGCTACTCGTTATTCTAAAGACTTTTGGATTCCTGAAGCTTATGAAGCATTAATTAGAGATTGYTATTTAGGTAATCATTCTAATTTTGTTAGAGATGATGAATTGGATGTTTCTTGGAAATTATTTACTCCATTATTGAAT

>Mi3_pharynx_19/03/2012

AATTATTGAARTGGAATGATATTCCATTGGCTCCACCAGACAARATTTTGGGTATTTCTGAAGCTTATAACAATGATTCTAACCCTCAAAAARTCAATTTGGGGGTTGGTGCTTATAGAGATAATTCYGGTAAACCAATTATTTTCCCATCAGTTAAAAAAGCTGAAGAAATTTTATTGGGTAAAGAAACTGAAAAGGAATATACTGCCATTGTTGGTTCCAAAAATTTCCAATCAATTGTGAAAAATTTCATTTTCAACAATTCTAATAAAGATGCCAATGGTAAACAATTAATTGATGATGGTAGAATTGTTACTGCTCAAACCATCTCTGGTACTGGATCACTTAAACCTCTTATGGTGAAATTTTTGCTAAACATAGAGAACCAAATTTGGAAATTATTCGTGAGGTTGTTGATTCCAAACATATTGTTTTTGATGTGTTGGCACAATTCTTAATCAATCCAGACCCATGGGTTGCCATTGCTGCCGCTGAAGTTTATGTCAGACGTTCATACCGTGCTTATGATTTGGGTAMAATTGAATATCATGTTAATGACAGACTTCCTATTGTTGAATGGAAATTCAAGTTGGCTAATATGGGAGCCGCTGGTGTAAACGATGCTCAACAGGCTGCTGCTGCCGGTGGCGATGATTCGACATCTATGAAACATGCAGCTTCTGTGTCTGATTTGACCTTTGTTGTTGATTATTCATRAAAGCGAAATCCCAGGATACACTCTCCCCGATAATCCAAAGTTCACCCTTGGTAATTTGTTTGTAATAATTGGAGTCTTGTTGGTTTGTATTTTAGCTGTTGTCTCTCTTTTGAGAAATATTAGTGAGTCGGCCTTGTTCAAGAAGAATGGGTATGAACCGTTGGATTCGGATCCTAGTGTCATGAACCTAAACTTCGAGCCTACAACATTGTCCTTTGAAGATATTAAATATGAGGTTACTGGTGGTCGACAAATTTTAAATGGAGTCTTTGGGTTTGTAAAACCAAGAGAATGTTTGGCTATAATGGGAGGTTCAGGTGCTGGTAAAACTACATTGTTGGATTTTAAACCTTTGGACCAATTGGCTAARACTTTGRCYACTGTTCCTGAATTGAATGAAATTATTGGKCAAGAWTTAGTTGACGAATTTRTCAGTGGTATTAAACTACCAGCAGAAGTTGGAAGTCAAGATGATGTTAACAATAGAAAATTGTTGCAAAAAGTGTTTGGTAAATTAATGAACACTGATGATGACGTTATAAAACAACAAACAGCTAAATTACTTGAAAGAACAGACAGAGAACCTCAAGTGTTCAAGGATATTGATTCTAGATTACCGGAGTTAATACAAAGATTAAACAAACAATTTCCTAATGACATCGGATTATTTTGTGGATGTCTCTTATTGAACCACGTTGATTGAAAAAATTATCAATTTCCGTGTTGGATAAACAGAGATTGACTGAAAAATTCAATAAATTGGATAAATCCATCAAAGATAATTTGAAGGCTAAACAAAAAGAAGAAACCAAAAAAACTTTAGATGTGGTTAATAATTGGTTGAATGATAAAGAAAATGCTTCATCATTTTTGGTTGCTCACGTTCCAATTACTGCTAATGCCAAGGCAATCACTGAAGCCATTAATTTGATTAAAAAGCAAGATAAAACCAAATCAATTTATTTATTGACTGGTGAAACCGATAAAGTTGCTCATGGATGTTATGTTAGTGATGAAGCCATTGCCAAGGGTATTAATGCGAAAATCAAACCTTTTGAAGCACGAGCAATTAACTGGTCCACGGATCTTAATGCTGAGGTACATATTGAGCATTATATAAATATATTCAATTATGCACGATCATCTTGGGAGCCATTGGTTGAAAGTTGGCCAATAGCAGTTTACATGTCAAAATCCCGACACCCAAAGCCTCAATTATTAGTAGAGGTGATTTCTAGACAGGTAGCCCAAGTGACGCTTACATCCAAAGCAGTAGCATTGCTATCTCAAGTATCCGATTTGATTACTTCCGGAGAAAAATTAAAACCAAGAGGTGAAGATTACCCATACGTTATAGTGAATGAGACTGGTTTAGATTTGGAAGTTTGGAATGATGCAAAAGAATCCGAAACCAATGGAGTTCCAATTGTTTTACGTGCTGGTAAAGCTTTAGATGAAAGTAAAGTTGAAATTAGAATTCAATTTAAACCAGTCGCCAAGGGGATGTTTAAAGAAATTCAAAGAAATGAATTAGTTATTAGAGTACAACCAAATGAAGCCATTTATTTAAAAATTAATTCCAAAATCCCTGGAATTTCTACTGAAACTTCATTAACTGATTTAGATTTAACTTATGCTACTCGTTATTCTAAAGAYTTTTGGATTCCTGAAGCTTATGAAGCATTAATTAGAGATTGYTATTTAGGTAATCATTCTAATTTTGTTAGAGATGATGAATTGGATGTTTCTTGGAAATTATTTACTCCATTATTGAAT

>Mi4_pharynx_9/04/2012

AATTATTGAAATGGAATGATATTCCATTGGCTCCACCAGACAAAATTTTGGGTATTTCTGAAGCTTATAACAATGATTCTAACCCTCAAAAAATCAATTTGGGGGTTGGTGCTTATAGAGATAATTCCGGTAAACCAATTATTTTCCCATCAGTTAAAAAAGCTGAAGAAATTTTATTGGGTAAAGAAACTGAAAAGGAATATACTGCCATTGTTGGTTCCAAAAATTTCCAATCAATTGTGAAAAATTTCATTTTCAACAATTCTAATAAAGATGCCAATGGTAAACAATTAATTGATGATGGTAGAATTGTTACTGCTCAAACCATCTCTGGTACTGGATCACTTAAACCTCTTATGGTGAAATTTTTGCTAAACATAGAGAACCAAATTTGGAAATTATTCGTGAGGTTGTTGATTCCAAACATATTGTTTTTGATGTGTTGGCACAATTCTTAATCAATCCAGACCCATGGGTTGCCATTGCTGCCGCTGAAGTTTATGTCAGACGTTCATACCGTGCTTATGATTTGGGTAAAATTGAATATCATGTTAATGACAGACTTCCTATTGTTGAATGGAAATTCAAGTTGGCTAATATGGGAGCYGCTGGTGTAAACGATGCTCAACAGGCTGCTGCTGCCGGTGGCGATGATTCGACATCTATGAAACATGCAGCTTCTGTGTCTGATTTGACCTTTGTTGTTGATTATTCATAAAAGYGAAATYCCAGGATACACTCTCCCCGATAATCCAAAGTTCACCCTTGGTAATTTGTTTGTAATAATTGGRGTCTTGTTGGTTTGTRTTTTAGCTGTTGTCTCTCTTTTGAGAAATATTAGTGAGTCRGCCTTGTTCAAGAAGAATGGGTATGAACCGTTGGATTCRGATCCTAGTGTCATGAACCWAAACTTYGAGCCTACAACATTGTCCTTTGAAGATATTAAATATGAGGTTACTGGTGGTCGACAAATTTTAAATGGAGTCTTTGGGTTTGTAAAACCAAGAGAATGTTTGGCTATAATGGGAGGTTCAGGTGCTGGTAAAACTACATTGTTGGATTTTAAACCTTTGGACCAATTRGCTAAGACTTTGACCACTGTTCCTGAATTGAATGAAATTATTGGGCAAGATTTAGTTGACGAATTTGTCAGTGGTATTAAACTACCAGCAGAAGTTGGAAGTCAAGATGATGTTAACAATAGAAAATTGTTGCAAAAAGTGTTTGGTAAATTAATGAACACTGATGATGACGTTATAAAACAACAAACAGCTAAATTACTTGAAAGAACAGACAGAGAACCTCAAGTGTTCAAGGATATTGATTCTAGATTACCGGAGTTAATACAAAGATTAAACAAACAATTTCCTAATGACATCGGATTATTTTGTGGATGTCTCTTATTGAACCACGTTGMTTGAAAAAATTATCAATTTCCGTGTTGGATAAACAGAGATTGACTGAAAAATTCAATAAATTGGATAAATCCATCAAAGATAATTTGAAGGCTAAACAAAAAGAAGAAACCAAAAAAACTTTAGATGTGGTTAATAATTGGTTGAATGATAAAGAAAATGCTTCATCATTTTTGGTTGCTCACGTTCCAATTACTGCTAATGCCAAGGCAATCACTGAAGCCATTAATTTGATTAAAAAGCAAGATAAAACCAAATCAATTTATTTATTGACTGGTGAAACCGATAAAGTTGCTCATGGATGTTATGTTAGTGATGAAGCCATTGCCAAGGGTATTAATGCGAAAATCAAACCTTTTGAAGCACGAGCAATTAACTGGTCCACGGATCTTAATGCTGAGGTACATATTGAGCATTATATAAATATATTCAATTATGCACGATCATCTTGGGAGCCATTGGTTGAAAGTTGGCCAATAGCAGTTTACATGTCAAAATCCCGACACCCAAAGCCTCAATTATTAGTAGAGGTGATTTCTAGACAGRTAGCTCAAGTGACGCTTACATCCAAAGCGGTAGCATTGCTATCTCAAGTATCCGATTTGATTACTTCCGRAGAAAAATTAAAACCAAGAGGTGAAGATTACCCATACGTTATAGTGAATGARACTGGTTTAGATTTGGAAGTTTGGAATGATGCAAAWGAATCCGAAACCAATGGAGTTCCAATTGTTTTACGTGCTGGTAAAGCTTTAGATGAAAGTAAAGTTGAAATTAGAATTCAATTTAAACCAGTCGCCAAGGGGATGTTTAAAGAAATTCAAAGAAATGAATTAGTTATTAGAGTACAACCAAATGAAGCCATTTATTTAAAAATTAATTCCAAAATCCCTGGAATTTCTACTGAAACTTCATTAACTGATTTAGATTTAACTTATGCTACTCGTTATTCTAAAGAYTTTTGGATTCCTGAAGCTTATGAAGCATTAATTAGAGATTGTTATTTAGGTAATCATTCTAATTTTGTTAGAGATGATGAATTGGATGTTTCTTGGAAATTATTTACTCCATTATTGAAT

>Mi4_rectal_9/04/2012

AATTATTGAAATGGAATGATATTCCATTGGCTCCACCAGACAAAATTTTGGGTATTTCTGAAGCTTATAACAATGATTCTAACCCTCAAAAAATCAATTTGGGGGTTGGTGCTTATAGAGATAATTCCGGTAAACCAATTATTTTCCCATCAGTTAAAAAAGCTGAAGAAATTTTATTGGGTAAAGAAACTGAAAAGGAATATACTGCCATTGTTGGTTCCAAAAATTTCCAATCAATTGTGAAAAATTTCATTTTCAACAATTCTAATAAAGATGCCAATGGTAAACAATTAATTGATGATGGTAGAATTGTTACTGCTCAAACCATCTCTGGTACTGGATCACTTAAACCTCTTATGGTGAAATTTTTGCTAAACATAGAGAACCAAATTTGGAAATTATTCGTGAGGTTGTTGATTCCAAACATATTGTTTTTGATGTGTTGGCACAATTCTTAATCAATCCAGACCCATGGGTTGCCATTGCTGCCGCTGAAGTTTATGTCAGACGTTCATACCGTGCTTATGATTTGGGTAAAATTGAATATCATGTTAATGACAGACTTCCTATTGTTGAATGGAAATTCAAGTTGGCTAATATGGGAGCYGCTGGTGTAAACGATGCTCAACAGGCTGCTGCTGCCGGTGGCGATGATTCGACATCTATGAAACATGCAGCTTCTGTGTCTGATTTGACCTTTGTTGTTGATTATTCATAAAAGYGAAATYCCAGGATACACTCTCCCCGATAATCCAAAGTTCACCCTTGGTAATTTGTTTGTAATAATTGGRGTCTTGTTGGTTTGTRTTTTAGCTGTTGTCTCTCTTTTGAGAAATATTAGTGAGTCRGCCTTGTTCAAGAAGAATGGGTATGAACCGTTGGATTCRGATCCTAGTGTCATGAACCWAAACTTYGAGCCTACAACATTGTCCTTTGAAGATATTAAATATGAGGTTACTGGTGGTCGACAAATTTTAAATGGAGTCTTTGGGTTTGTAAAACCAAGAGAATGTTTGGCTATAATGGGAGGTTCAGGTGCTGGTAAAACTACATTGTTGGATTTTAAACCTTTGGACCAATTRGCTAAGACTTTGACCACTGTTCCTGAATTGAATGAAATTATTGGGCAAGATTTAGTTGACGAATTTGTCAGTGGTATTAAACTACCAGCAGAAGTTGGAAGTCAAGATGATGTTAACAATAGAAAATTGTTGCAAAAAGTGTTTGGTAAATTAATGAACACTGATGATGACGTTATAAAACAACAAACAGCTAAATTACTTGAAAGAACAGACAGAGAACCTCAAGTGTTCAAGGATATTGATTCTAGATTACCGGAGTTAATACAAAGATTAAACAAACAATTTCCTAATGACATCGGATTATTTTGTGGATGTCTCTTATTGAACCACGTTGMTTGAAAAAATTATCAATTTCCGTGTTGGATAAACAGAGATTGACTGAAAAATTCAATAAATTGGATAAATCCATCAAAGATAATTTGAAGGCTAAACAAAAAGAAGAAACCAAAAAAACTTTAGATGTGGTTAATAATTGGTTGAATGATAAAGAAAATGCTTCATCATTTTTGGTTGCTCACGTTCCAATTACTGCTAATGCCAAGGCAATCACTGAAGCCATTAATTTGATTAAAAAGCAAGATAAAACCAAATCAATTTATTTATTGACTGGTGAAACCGATAAAGTTGCTCATGGATGTTATGTTAGTGATGAAGCCATTGCCAAGGGTATTAATGCGAAAATCAAACCTTTTGAAGCACGAGCAATTAACTGGTCCACGGATCTTAATGCTGAGGTACATATTGAGCATTATATAAATATATTCAATTATGCACGATCATCTTGGGAGCCATTGGTTGAAAGTTGGCCAATAGCAGTTTACATGTCAAAATCCCGACACCCAAAGCCTCAATTATTAGTAGAGGTGATTTCTAGACAGRTAGCTCAAGTGACGCTTACATCCAAAGCGGTAGCATTGCTATCTCAAGTATCCGATTTGATTACTTCCGRAGAAAAATTAAAACCAAGAGGTGAAGATTACCCATACGTTATAGTGAATGARACTGGTTTAGATTTGGAAGTTTGGAATGATGCAAAWGAATCCGAAACCAATGGAGTTCCAATTGTTTTACGTGCTGGTAAAGCTTTAGATGAAAGTAAAGTTGAAATTAGAATTCAATTTAAACCAGTCGCCAAGGGGATGTTTAAAGAAATTCAAAGAAATGAATTAGTTATTAGAGTACAACCAAATGAAGCCATTTATTTAAAAATTAATTCCAAAATCCCTGGAATTTCTACTGAAACTTCATTAACTGATTTAGATTTAACTTATGCTACTCGTTATTCTAAAGAYTTTTGGATTCCTGAAGCTTATGAAGCATTAATTAGAGATTGTTATTTAGGTAATCATTCTAATTTTGTTAGAGATGATGAATTGGATGTTTCTTGGAAATTATTTACTCCATTATTGAAT

>Mi5_rectal_31/05/2012

AATTATTGAAATGGAATGATATTCCATTGGCTCCACCAGACAAGATTTTGGGTATTTCTGAAGCTTATAACAATGATTCTAACCCTCAAAAAGTCAATTTGGGGGTTGGTGCTTATAGAGATAATTCTGGTAAACCAATTATTTTCCCATCAGTTAAAAAAGCTGAAGAAATTTTATTGGGTAAAGAAACTGAAAAGGAATATACTGCCATTGTTGGTTCCAAAAATTTCCAATCAATTGTGAAAAATTTCATTTTCAACAATTCTAATAAAGATGCCAATGGTAAACAATTAATTGATGATGGTAGAATTGTTACTGCTCAAACCATCTCTGGTACTGGATCACTTAAACCTCTTATGGTGAAATTTTTGCTAAACATAGAGAACCAAATTTGGAAATTATTCGTGAGGTTGTTGATTCCAAACATATTGTTTTTGATGTGTTGGCACAATTCTTAATCAATCCAGACCCATGGGTTGCCATTGCTGCCGCTGAAGTTTATGTCAGACGTTCATACCGTGCTTATGATTTGGGTACAATTGAATATCATGTTAATGACAGACTTCCTATTGTTGAATGGAAATTCAAGTTGGCTAATATGGGAGCCGCTGGTGTAAACGATGCTCAACAGGCTGCTGCTGCTGGTGGCGATGATTCGACATCTATGAAACATGCAGCTTCTGTGTCTGATTTGACCTTTGTTGTTGATTATTCATRAAAGCGAAATCCCAGGATACACTCTCCCCGATAATCCAAAGTTCACCCTTGGTAATTTGTTTGTAATAATTGGAGTCTTGTTGGTTTGTATTTTAGCTGTTGTCTCTCTTTTGAGAAATATTAGTGAGTCGGCCTTGTTCAAGAAGAATGGGTATGAACCGTTGGATTCGGATCCTAGTGTCATGAACCTAAACTTCGAGCCTACAACATTGTCCTTTGAAGATATTAAATATGAGGTTACTGGTGGTCGACAAATTTTAAATGGAGTCTTTGGGTTTGTAAAACCAAGAGAATGTTTGGCTATAATGGGAGGTTCAGGTGCTGGTAAAACTACATTGTTGGATTTTAAACCTTTGGACCAATTGGCTAARACTTTGRCYACTGTTCCTGAATTGAATGAAATTATTGGKCAAGAWTTAGTTGACGAATTTRTCAGTGGTATTAAACTACCAGCAGAAGTTGGAAGTCAAGATGATGTTAACAATAGAAAATTGTTGCAAAAAGTGTTTGGTAAATTAATGAACACTGATGATGACGTTATAAAACAACAAACAGCTAAATTACTTGAAAGAACAGACAGAGAACCTCAAGTGTTCAAGGATATTGATTCTAGATTACCGGAGTTAATACAAAGATTAAACAAACAATTTCCTAATGACATCGGATTATTTTGTGGATGTCTCTTATTGAACCACGTTGATTGAAAAAATTATCAATTTCCGTGTTGGATAAACAGAGATTGACTGAAAAATTCAATAAATTGGATAAATCCATCAAAGATAATTTGAAGGCTAAACAAAAAGAAGAAACCAAAAAAACTTTAGATGTGGTTAATAATTGGTTGAATGATAAAGAAAATGCTTCATCATTTTTGGTTGCTCACGTTCCAATTACTGCTAATGCCMARGSMAWYMMYKRARSCMWTWATTTGATTAAAAAGCAAGATAAAACCAAATCAATTTATTTATTGACTGGTGAAACCGATAAAGTTGCTCATGGATGTTATGTTAGTGATGAAGCCATTGCCAAGGGTATTAATGCGAAAATCAAACCTTTTGAAGCACGAGCAATTAACTGGTCCACGGATCTTAATGCTGAGGTACATATTGAGCATTATATAAATATATTCAATTATGCACGATCATCTTGGGAGCCATTGGTTGAAAGTTGGCCAATAGCAGTTTACATGTCAAAATCCCGACACCCAAAGCCTCAATTATTAGTAGAGGTGATTTCTAGACAGGTAGCYCAAGTGACGCTTACATCCAAAGCAGTAGCATTGCTATCTCAAGTATCCGATTTGATTACTTCCRGAGAAAAATTAAAACCAAGAGGTGAAGATTACCCATACGTKATAGTGAATGAGACTGGTTTAGATTTGGAAGTTTGGAATGATGCAAAWGAATYCGAAACCAATGGAGTTCCAATTGTTTTACGTGCTGGTAAAGCTTTAGATGAAAGTAAAGTTGAAATTAGAATTCAATTTAAACCAGTCGCCAAGGGGATGTTTAAAGAAATTCAAAGAAATGAATTAGTTATTAGAGTACAACCAAATGAAGCCATTTATTTAAAAATTAATTCCAAAATCCCTGGAATTTCTACTGAAACTTCATTAACTGATTTAGATTTAACTTATGCTACTCGTTATTCTAAAGAYTTTTGGATTCCTGAAGCTTATGAAGCATTAATTAGAGATTGYTATTTAGGTAATCATTCTAATTTTGTTAGAGATGATGAATTGGATGTTTCTTGGAAATTATTTACTCCATTATTGAAT

>Mi6_oral_22/06/2012

AATTATTGAAATGGAATGATATTCCATTGGCTCCACCAGACAAAATTTTGGGTATTTCTGAAGCTTATAACAATGATTCTAACCCTCAAAAAATCAATTTGGGGGTTGGTGCTTATAGAGATAATTCCGGTAAACCAATTATTTTCCCATCAGTTAAAAAAGCTGAAGAAATTTTATTGGGTAAAGAAACTGAAAAGGAATATACTGCCATTGTTGGTTCCAAAAATTTCCAATCAATTGTGAAAAATTTCATTTTCAACAATTCTAATAAAGATGCCAATGGTAAACAATTAATTGATGATGGTAGAATTGTTACTGCTCAAACCATCTCTGGTACTGGATCACTTAAACCTCTTATGGTGAAATTTTTGCTAAACATAGAGAACCAAATTTGGAAATTATTCGTGAGGTTGTTGATTCCAAACATATTGTTTTTGATGTGTTGGCACAATTCTTAATCAATCCAGACCCATGGGTTGCCATTGCTGCCGCTGAAGTTTATGTCAGACGTTCATACCGTGCTTATGATTTGGGTAAAATTGAATATCATGTTAATGACAGACTTCCTATTGTTGAATGGAAATTCAAGTTGGCTAATATGGGAGCTGCTGGTGTAAACGATGCTCAACAGGCTGCTGCTGCCGGTGGCGATGATTCGACATCTATGAAACATGCAGCTTCTGTGTCTGATTTGACCTTTGTTGTTGATTATTCATAAAAGYGAAATYCCAGGATACACTCTCCCCGATAATCCAAAGTTCACCCTTGGTAATTTGTTTGTAATAATTGGRGTCTTGTTGGTTTGTRTTTTAGCTGTTGTCTCTCTTTTGAGAAATATTAGTGAGTCRGCCTTGTTCAAGAAGAATGGGTATGAACCGTTGGATTCRGATCCTAGTGTCATGAACCWAAACTTYGAGCCTACAACATTGTCCTTTGAAGATATTAAATATGAGGTTACTGGTGGTCGACAAATTTTAAATGGAGTCTTTGGGTTTGTAAAACCAAGAGAATGTTTGGCTATAATGGGAGGTTCAGGTGCTGGTAAAACTACATTGTTGGATTTTAAACCTTTGGACCAATTRGCTAAGACTTTGACCACTGTTCCTGAATTGAATGAAATTATTGGGCAAGATTTAGTTGACGAATTTGTCAGTGGTATTAAACTACCAGCAGAAGTTGGAAGTCAAGATGATGTTAACAATAGAAAATTGTTGCAAAAAGTGTTTGGTAAATTAATGAACACTGATGATGACGTTATAAAACAACAAACAGCTAAATTACTTGAAAGAACAGACAGAGAACCTCAAGTGTTCAAGGATATTGATTCTAGATTACCGGAGTTAATACAAAGATTAAACAAACAATTTCCTAATGACATCGGATTATTTTGTGGATGTCTCTTATTGAACCACGTTGMTTGAAAAAATTATCAATTTCCGTGTTGGATAAACAGAGATTGACTGAAAAATTCAATAAATTGGATAAATCCATCAAAGATAATTTGAAGGCTAAACAAAAAGAAGAAACCAAAAAAACTTTAGATGTGGTTAATAATTGGTTGAATGATAAAGAAAATGCTTCATCATTTTTGGTTGCTCACGTTCCAATTACTGCTAATGCCAAGGCAATCACTGAAGCCATTAATTTGATTAAAAAGCAAGATAAAACCAAATCAATTTATTTATTGACTGGTGAAACCGATAAAGTTGCTCATGGATGTTATGTTAGTGATGAAGCCATTGCCAAGGGTATTAATGCGAAAATCAAACCTTTTGAAGCACGAGCAATTAACTGGTCCACGGATCTTAATGCTGAGGTACATATTGAGCATTATATAAATATATTCAATTATGCACGATCATCTTGGGAGCCATTGGTTGAAAGTTGGCCAATAGCAGTTTACATGTCAAAATCCCGACACCCAAAGCCTCAATTATTAGTAGAGGTGATTTCTAGACAGRTAGCTCAAGTGACGCTTACATCCAAAGCGGTAGCATTGCTATCTCAAGTATCCGATTTGATTACTTCCGRAGAAAAATTAAAACCAAGAGGTGAAGATTACCCATACGTTATAGTGAATGARACTGGTTTAGATTTGGAAGTTTGGAATGATGCAAAWGAATCCGAAACCAATGGAGTTCCAATTGTTTTACGTGCTGGTAAAGCTTTAGATGAAAGTAAAGTTGAAATTAGAATTCAATTTAAACCAGTCGCCAAGGGGATGTTTAAAGAAATTCAAAGAAATGAATTAGTTATTAGAGTACAACCAAATGAAGCCATTTATTTAAAAATTAATTCCAAAATCCCTGGAATTTCTACTGAAACTTCATTAACTGATTTAGATTTAACTTATGCTACTCGTTATTCTAAAGAYTTTTGGATTCCTGAAGCTTATGAAGCATTAATTAGAGATTGTTATTTAGGTAATCATTCTAATTTTGTTAGAGATGATGAATTGGATGTTTCTTGGAAATTATTTACTCCATTATTGAAT

>Mi6_rectal_19/07/2012

AATTATTGAAATGGAATGATATTCCATTGGCYCCACCAGACAAGATTTTGGGTATTTCTGAAGCTTATAACAATGATTCTAACCCTCAAAAARTCAATTTGGGGGTTGGTGCTTATAGAGATAATTCTGGTAAACCAATTATTTTCCCATCAGTTAAAAAAGCTGAAGAAATTTTATTGGGTAAAGAAACTGAAAAGGAATATACTGCCATTGTTGGTTCCAAAAATTTCCAATCAATTGTGAAAAATTTCATTTTCAACAATTCTAATAAAGATGCCAATGGTAAACAATTAATTGATGATGGTAGAATTGTTACTGCTCAAACCATYTCTGGTACTGGATCACTTAAACCTCTTATGGTGAAATTTTTGCTAAACATAGAGAACCAAATTTGGAAATTATTCGTGAGGTTGTTGATTCCAAACATATTGTTTTTGATGTGTTGGCACAATTCTTAATCAATCCAGACCCATGGGTTGCCATTGCTGCCGCTGAAGTTTATGTCAGACGTTCATACCGTGCTTATGATTTGGGTAAAATTGAATATCATGTTAATGACAGACTTCCTATTGTTGAATGGAAATTCAAGTTGGCTAATATGGGAGCYGCTGGTGTAAACGATGCTCAACAGGCTGCTGCTGCCGGTGGCGATGATTCGACATCTATGAAACATGCAGCTTCTGTGTCTGATTTGACCTTTGTTGTTGATTATTCATAAAAGCGAAATCCCAGGATACACTCTCCCCGATAATCCAAAGTTCACCCTTGGTAATTTGTTTGTAATAATTGGAGTCTTGTTGGTTTGTATTTTAGCTGTTGTCTCTCTTTTGAGAAATATTAGTGAGTCGGCCTTGTTCAAGAAGAATGGGTATGAACCGTTGGATTCGGATCCTAGTRTCATGAACCTAAACTTCGAGCCTACAACATTGTCCTTTGAAGATATTAAATATGAGGTTACTGGTGGTCGACAAATTTTAAATGGAGTCTTTGGGTTTGTAAAACCAAGAGAATGTTTGGCTATAATGGGAGGTTCAGGTGCTGGTAAAACTACATTGTTGGATTTTAAACCTTTGGACCAATTGGCTAAGACTTTGACCACTGTTCCTGAATTGAATGAAATTATTGGGCAAGATTTAGTTGACGAATTTGTCAGTGGTATTAAACTACCAGCAGAAGTTGGAAGTCAAGATGATGTTAACAATAGAAAATTGTTGCAAAAAGTGTTTGGTAAATTAATGAACACTGATGATGACGTTATAAAACAACAAACAGCTAAATTACTTGAAAGAACAGACAGAGAACCTCAAGTGTTCAAGGATATTGATTCTAGATTACCGGAGTTAATACAAAGATTAAACAAACAATTTCCTAATGACATCGGATTATTTTGTGGATGTCTCTTATTGAACCACGTTGMTTGAAAAAATTATCAATTTCCGTGTTGGATAAACARAGATTGACTGAAAAATTCAATAAATTGGATAAATCCATYAAAGATAATTTGAAGGCTAAACAAAAAGAAGAAACCAAAAARACTTTAGATGTGGTTAAYAATTGGTTGAATGATAAAGAAAATGCTTCATCATTTTTGGTTGCTCACGTTCCAATTACTGCTAATGCCAAGGCAATCACTGAAGCCATTAATTTGATTAAAAAGCAAGATAAAACCAAATCAATTTATTTATTGACTGGTGAAACCGATAAAGTTGCTCATGGATGTTATGTTAGTGATGAAGCCATTGYCAAGGGTATTAATGCGAAAATCAAACCTTTTGAAGCACGAGCAATTAACTGGTCCACGGATCTTAATGCTGAGGTACATATTGAGCATTATATAAATATATTCAATTATGCACGATCATCTTGGGAGCCATTGGTTGAAAGTTGGCCAATAGCAGTTTACATGTCAAAATCCCGACACCCAAAGCCTCAATTATTAGTAGAGGTGATTTCTAGACAGGTAGCTCAAGTGACGCTTACATCCAAAGCAGTAGCATTGCTATCTCAAGTATCCGATTTGATTACTTCCAGAGAAAAATTAAAACCAAGAGGTGAAGATTACCCATACGTTATAGTGAATGAGACTGGTTTAGATTTGGAAGTTTGGAATGATGCAAATGAATCCGAAACCAATGGAGTTCCAATTGTTTTACGTGCTGGTAAAGCTTTAGATGAAAGTAAAGTTGAAATTAGAATTCAATTTAAACCAGTCGCCAAGGGGATGTTTAAAGAAATTCAAAGAAATGAATTAGTTATTAGAGTACAACCAAATGAAGCCATTTATTTAAAAATTAATTCCAAAATCCCTGGAATTTCTACTGAAACTTCATTAACTGATTTAGATTTAACTTATGCTACTCGTTATTCTAAAGACTTTTGGATTCCTGAAGCTTATGAAGCATTAATTAGAGATTGTTATTTAGGTAATCATTCTAATTTTGTTAGAGATGATGAATTGGATGTTTCTTGGAAATTATTTACTCCATTATTGAAT

>Mi7_oral_4/07/2012

AATTATTGAAATGGAATGATATTCCATTGGCTCCACCAGACAAAATTTTGGGTATTTCTGAAGCTTATAACAAYGATTCTAACCCTCAAAAAATCAATTTGGGGGTTGGTGCTTATAGAGATAATTCCGGTAAACCAATTATTTTCCCATCAGTTAAAAAAGCTGAAGAAATTTTATTGGGTAAAGAAACTGAAAAGGAATATACTGCCATTGTTGGTTCCAAAAATTTCCAATCAATTGTGAAAAATTTCATTTTCAACAATTCTAATAAAGATGCCAATGGTAAACAATTAATTGATGATGGTAGAATTGTTACTGCTCAAACCATCTCTGGTACTGGATCACTTAAACCTCTTATGGTGAAATTTTTGCTAAACATAGAGAACCAAATTTGGAAATTATTCGTGAGGTTGTTGATTCCAAACATATTGTTTTTGATGTGTTGGCACAATTCTTAATCAATCCAGACCCATGGGTTGCCATTGCTGCCGCTGAAGTTTATGTCAGACGTTCATACCGTGCTTATGATTTGGGTAMAATTGAATATCATGTTAATGACAGACTTCCTATTGTTGAATGGAAATTCAAGTTGGCTAATATGGGAGCCGCTGGTGTAAACGATGCTCAACAGGCTGCTGCTGCTGGTGGCGATGATTCGACATCTATGAAACATGCAGCTTCTGTGTCTGATTTGACCTTTGTTGTTGATTATTCATAAAAGYGAAATYCCAGGATACACTCTCCCCGATAATCCAAAGTTCACCCTTGGTAATTTGTTTGTAATAATTGGRGTCTTGTTGGTTTGTRTTTTAGCTGTTGTCTCTCTTTTGAGAAATATTAGTGAGTCRGCCTTGTTCAAGAAGAATGGGTATGAACCGTTGGATTCRGATCCTAGTGTCATGAACCWAAACTTYGAGCCTACAACATTGTCCTTTGAAGATATTAAATATGAGGTTACTGGTGGTCGACAAATTTTAAATGGAGTCTTTGGGTTTGTAAAACCAAGAGAATGTTTGGCTATAATGGGAGGTTCAGGTGCTGGTAAAACTACATTGTTGGATTTTAAACCTTTGGACCAATTGGCTAAGACTTTGACCACTGTTCCTGAATTGAATGAAATTATTGGGCAAGATTTAGTTGACGAATTTGTCAGTGGTATTAAACTACCAGCAGAAGTTGGAAGTCAAGATGATGTTAACAATAGAAAATTGTTGCAAAAAGTGTTTGGTAAATTAATGAACACTGATGATGACGTTATAAAACAACAAACAGCTAAATTACTTGAAAGAACAGAAAGGGAACCTCAAGTGTTCAAGGATATTGATTCTAGATTACCAGAGTTAATACAAGGATTAAACAAACAATTTCCTAATGACATCGGATTATTTTGTGGATGTCTCTTATTGAACCACGTTGMTTGAAAAAATTATCAATTTCCGTGTTGGATAAACAAAGATTGACTGAAAAATTCAATAAATTGGATAAATCCATTAAAGATAATTTGAAGGCTAAACAAAAAGAAGAAACCAAAAAGACTTTAGATGTGGTTAACAATTGGTTGAATGATAAAGAAAATGCTTCATCATTTTTGGTTGCTCACGTTCCAATTACTGCTAATGCCAAGGCAATCACTGAAGCCATTAATTTGATTAAAAAGCAAGATAAAACCAAATCAATTTATTTATTGACTGGTGAAACAGATAAAGTTGCTCATGGATGTTATGTTAGTGATGAAGCCATTGCCAAGGGTATTAATGCGAAAATCAAACCTTTTGAAGCACGAGCAATTAACTGGTCAACGGATCTTAATGCTGAGGTACATATTGAGCATTATATAAATATATTCAATTATGCACGATCATCTTGGGAGCCATTGGTTGAAWGTTGGCCAATAGCAGTTTACATGTCAAAATCCCGACACCCAAAGCCTCAATTATTAGTAGAGGTGATTTCTAGACAGGTAGCTCAAGTGACGCTTACATCCAAAGCAGTAGCATTGCTATCTCAAGTATCCGATTTGATTACTTCCRGAGAAAAATTAAAACCAAGAGGTGAAGATTACCCATACRTTATAGTGAATGAGACTGGTTTAGATTTGGAAGTTTGGAATGATGCAAAWGAATCCGAAACCAATGGAGTTCCAATTGTTTTACGTGCTGGTAAAGCTTTAGATGAAAGTAAAGTTGAAATTAGAATTCAATTTAAACCAGTCGCCAAGGGGATGTTTAAAGAAATTCAAAGAAATGAATTAGTTATTAGAGTACAACCAAATGAAGCCATTTATTTAAAAATTAATTCCAAAATCCCTGGAATTTCTACTGAAACTTCATTAACTGATTTAGATTTAACTTATGCTACTCGTTATTCTAAAGACTTTTGGATTCCTGAAGCTTATGAAGCATTAATTAGAGATTGTTATTTAGGTAATCATTCTAATTTTGTTAGAGATGATGAATTGGATGTTTCTTGGAAATTATTTACTCCATTATTGAAT

>Mi7_oral_9/07/2012

AATTATTGAAATGGAATGATATTCCATTGGCTCCACCAGACAAAATTTTGGGTATTTCTGAAGCTTATAACAAYGATTCTAACCCTCAAAAAATCAATTTGGGGGTTGGTGCTTATAGAGATAATTCCGGTAAACCAATTATTTTCCCATCAGTTAAAAAAGCTGAAGAAATTTTATTGGGTAAAGAAACTGAAAAGGAATATACTGCCATTGTTGGTTCCAAAAATTTCCAATCAATTGTGAAAAATTTCATTTTCAACAATTCTAATAAAGATGCCAATGGTAAACAATTAATTGATGATGGTAGAATTGTTACTGCTCAAACCATCTCTGGTACTGGATCACTTAAACCTCTTATGGTGAAATTTTTGCTAAACATAGAGAACCAAATTTGGAAATTATTCGTGAGGTTGTTGATTCCAAACATATTGTTTTTGATGTGTTGGCACAATTCTTAATCAATCCAGACCCATGGGTTGCCATTGCTGCCGCTGAAGTTTATGTCAGACGTTCATACCGTGCTTATGATTTGGGTAMAATTGAATATCATGTTAATGACAGACTTCCTATTGTTGAATGGAAATTCAAGTTGGCTAATATGGGAGCCGCTGGTGTAAACGATGCTCAACAGGCTGCTGCTGCTGGTGGCGATGATTCGACATCTATGAAACATGCAGCTTCTGTGTCTGATTTGACCTTTGTTGTTGATTATTCATAAAAGYGAAATYCCAGGATACACTCTCCCCGATAATCCAAAGTTCACCCTTGGTAATTTGTTTGTAATAATTGGRGTCTTGTTGGTTTGTRTTTTAGCTGTTGTCTCTCTTTTGAGAAATATTAGTGAGTCRGCCTTGTTCAAGAAGAATGGGTATGAACCGTTGGATTCRGATCCTAGTGTCATGAACCWAAACTTYGAGCCTACAACATTGTCCTTTGAAGATATTAAATATGAGGTTACTGGTGGTCGACAAATTTTAAATGGAGTCTTTGGGTTTGTAAAACCAAGAGAATGTTTGGCTATAATGGGAGGTTCAGGTGCTGGTAAAACTACATTGTTGGATTTTAAACCTTTGGACCAATTGGCTAAGACTTTGACCACTGTTCCTGAATTGAATGAAATTATTGGGCAAGATTTAGTTGACGAATTTGTCAGTGGTATTAAACTACCAGCAGAAGTTGGAAGTCAAGATGATGTTAACAATAGAAAATTGTTGCAAAAAGTGTTTGGTAAATTAATGAACACTGATGATGACGTTATAAAACAACAAACAGCTAAATTACTTGAAAGAACAGAAAGGGAACCTCAAGTGTTCAAGGATATTGATTCTAGATTACCAGAGTTAATACAAGGATTAAACAAACAATTTCCTAATGACATCGGATTATTTTGTGGATGTCTCTTATTGAACCACGTTGMTTGAAAAAATTATCAATTTCCGTGTTGGATAAACAAAGATTGACTGAAAAATTCAATAAATTGGATAAATCCATTAAAGATAATTTGAAGGCTAAACAAAAAGAAGAAACCAAAAAGACTTTAGATGTGGTTAACAATTGGTTGAATGATAAAGAAAATGCTTCATCATTTTTGGTTGCTCACGTTCCAATTACTGCTAATGCCAAGGCAATCACTGAAGCCATTAATTTGATTAAAAAGCAAGATAAAACCAAATCAATTTATTTATTGACTGGTGAAACAGATAAAGTTGCTCATGGATGTTATGTTAGTGATGAAGCCATTGCCAAGGGTATTAATGCGAAAATCAAACCTTTTGAAGCACGAGCAATTAACTGGTCAACGGATCTTAATGCTGAGGTACATATTGAGCATTATATAAATATATTCAATTATGCACGATCATCTTGGGAGCCATTGGTTGAAWGTTGGCCAATAGCAGTTTACATGTCAAAATCCCGACACCCAAAGCCTCAATTATTAGTAGAGGTGATTTCTAGACAGGTAGCTCAAGTGACGCTTACATCCAAAGCAGTAGCATTGCTATCTCAAGTATCCGATTTGATTACTTCCRGAGAAAAATTAAAACCAAGAGGTGAAGATTACCCATACRTTATAGTGAATGAGACTGGTTTAGATTTGGAAGTTTGGAATGATGCAAAWGAATCCGAAACCAATGGAGTTCCAATTGTTTTACGTGCTGGTAAAGCTTTAGATGAAAGTAAAGTTGAAATTAGAATTCAATTTAAACCAGTCGCCAAGGGGATGTTTAAAGAAATTCAAAGAAATGAATTAGTTATTAGAGTACAACCAAATGAAGCCATTTATTTAAAAATTAATTCCAAAATCCCTGGAATTTCTACTGAAACTTCATTAACTGATTTAGATTTAACTTATGCTACTCGTTATTCTAAAGACTTTTGGATTCCTGAAGCTTATGAAGCATTAATTAGAGATTGTTATTTAGGTAATCATTCTAATTTTGTTAGAGATGATGAATTGGATGTTTCTTGGAAATTATTTACTCCATTATTGAAT

>Mi8_oral_2/10/2012

AATTATTGAAATGGAATGATATTCCATTGGCCCCACCAGACAAGATTTTGGGTATTTCTGAAGCTTATAACAATGATTCTAACCCTCAAAAAATCAATTTGGGGGTTGGTGCTTATAGAGATAATTCTGGTAAACCAATTATTTTCCCATCAGTTAAAAAAGCTGAAGAAATTTTATTGGGTAAAGAAACTGAAAAGGAATATACTGCCATTGTTGGTTCCAAAAATTTCCAATCAATTGTGAAAAATTTCATTTTCAACAATTCTAATAAAGATGCCAATGGTAAACAATTAATTGATGATGGTAGAATTGTTACTGCTCAAACCATCTCTGGTACTGGATCACTTAAACCTCTTATGGTGAAATTTTTGCTAAACATAGAGAACCAAATTTGGAAATTATTCGTGARGTTGTTGATTCCAAACATATTGTTTTTGATGTGTTGGCACAATTCTTAATCAATCCAGACCCATGGGTTGCCATTGCTGCCGCTGAAGTTTATGTCAGACGTTCATACCGTGCTTATGATTTGGGTAAAATTGAATATCATGTTAATGACAGACTTCCTATTGTTGAATGGAAATTCAAGTTGGCTAATATGGGAGCCGCTGGTGTAAACGATGCTCAACAGGCTGCTGCTGCCGGTGGCGATGATTCGACATCTATGAAACATGCAGCTTCTGTGTCTGATTTGACCTTTGTTGTTGATTATTCATAAAAGCGAAATCCCAGGATACACTCTCCCCGATAATCCAAAGTTCACCCTTGGTAATTTGTTTGTAATAATTGGAGTCTTGTTGGTTTGTATTTTAGCTGTTGTCTCTCTTTTGAGAAATATTAGTGAGTCGGCCTTGTTCAAGAAGAATGGGTATGAACCGTTGGATTCGGATCCTAGTGTCATGAACCTAAACTTCGAGCCTACAACATTGTCCTTTGAAGATATTAAATATGAGGTTACTGGTGGTCGACAAATTTTAAATGGAGTCTTTGGGTTTGTAAAACCAAGAGAATGTTTGGCTATAATGGGAGGTTCAGGTGCTGGTAAAACTACATTGTTGGATTTTAAACCTTTGGACCAATTGGCTAARACTTTGRCYACTGTTCCTGAATTGAATGAAATTATTGGKCAAGAWTTAGTTGACGAATTTRTCAGTGGTATTAAACTACCAGCAGAAGTTGGAAGTCAAGATGATGTTAACAATAGAAAATTGTTGCAAAAAGTGTTTGGTAAATTAATGAACACTGATGATGACGTTATAAAACAACAAACAGCTAAATTACTTGAAAGAACAGAMAGRGAACCTCAAGTGTTCAAGGATATTGATTCTAGATTACCRGAGTTAATACAARGATTAAACAAACAATTTCCTAATGACATCGGATTATTTTGTGGATGTCTCTTATTGAACCACGTTGCTTGAAAAAATTATCAATTTCCGTGTTGGATAAACAGAGATTGACTGAAAAATTCAATAAATTGGATAAATCCATTAAAGATAATTTGAAGGCTAAACAAAAAGAAGAAACCAAAAAGACTTTAGATGTGGTTAACAATTGGTTGAATGATAAAGAAAATGCTTCATCATTTTTGGTTGCTCACGTTCCAATTACTGCTAATGCCAAGGCAATCACTGAAGCCATTAATTTGATTAAAAAGCAAGATAAAACCAAATCAATTTATTTATTGACTGGTGAAACCGATAAAGTTGCTCATGGATGTTATGTTAGTGATGAAGCCATTGTCAAGGGTATTAATGCGAAAATCAAACCTTTTGAAGCACGAGCAATTAACTGGTCCACGGATCTTAATGCTGAGGTACATATTGAGCATTATATAAATATATTCAATTATGCACGATCATCTTGGGAGCCATTGGTTGAAAGTTGGCCAATAGCAGTTTACATGTCAAAATCCCGACACCCAAAGCCTCAATTATTAGTAGAGGTGATTTCTAGACAGGTAGCCCAAGTGACGCTTACATCCAAAGCAGTAGCATTGCTATCTCAAGTATCCGATTTGATTACTTCCGGAGAAAAATTAAAACCAAGAGGTGAAGATTACCCATACGTTATAGTGAATGAGACTGGTTTAGATTTGGAAGTTTGGAATGATGCAAAAGAATCCGAAACCAATGGAGTTCCAATTGTTTTACGTGCTGGTAAAGCTTTAGATGAAAGTAAAGTTGAAATTAGAATTCAATTTAAACCAGTCGCCAAGGGGATGTTTAAAGAAATTCAAAGAAATGAATTAGTTATTAGAGTACAACCAAATGAAGCCATTTATTTAAAAATTAATTCCAAAATCCCTGGAATTTCTACTGAAACTTCATTAACTGATTTAGATTTAACTTATGCTACTCGTTATTCTAAAGAYTTTTGGATTCCTGAAGCTTATGAAGCATTAATTAGAGATTGTTATTTAGGTAATCATTCTAATTTTGTTAGAGATGATGAATTGGATGTTTCTTGGAAATTATTTACTCCATTATTGAAT

>Mi8_oral_11/07/2012

AATTATTGAAATGGAATGATATTCCATTGGCTCCACCAGACAAAATTTTGGGTATTTCTGAAGCTTATAACAATGATTCTAACCCTCAAAAAATCAATTTGGGGGTTGGTGCTTATAGAGATAATTCCGGTAAACCAATTATTTTCCCATCAGTTAAAAAAGCTGAAGAAATTTTATTGGGTAAAGAAACTGAAAAGGAATATACTGCCATTGTTGGTTCCAAAAATTTCCAATCAATTGTGAAAAATTTCATTTTCAACAATTCTAATAAAGATGCCAATGGTAAACAATTAATTGATGATGGTAGAATTGTTACTGCTCAAACCATCTCTGGTACTGGATCACTTAAACCTCTTATGGTGAAATTTTTGCTAAACATAGAGAACCAAATTTGGAAATTATTCGTGAGGTTGTTGATTCCAAACATATTGTTTTTGATGTGTTGGCACAATTCTTAATCAATCCAGACCCATGGGTTGCCATTGCTGCCGCTGAAGTTTATGTCAGACGTTCATACCGTGCTTATGATTTGGGTAAAATTGAATATCATGTTAATGACAGACTTCCTATTGTTGAATGGAAATTCAAGTTGGCTAATATGGGAGCYGCTGGTGTAAACGATGCTCAACAGGCTGCTGCTGCCGGTGGCGATGATTCGACATCTATGAAACATGCAGCTTCTGTGTCTGATTTGACCTTTGTTGTTGATTATTCATAAAAGYGAAATYCCAGGATACACTCTCCCCGATAATCCAAAGTTCACCCTTGGTAATTTGTTTGTAATAATTGGRGTCTTGTTGGTTTGTRTTTTAGCTGTTGTCTCTCTTTTGAGAAATATTAGTGAGTCRGCCTTGTTCAAGAAGAATGGGTATGAACCGTTGGATTCRGATCCTAGTGTCATGAACCWAAACTTYGAGCCTACAACATTGTCCTTTGAAGATATTAAATATGAGGTTACTGGTGGTCGACAAATTTTAAATGGAGTCTTTGGGTTTGTAAAACCAAGAGAATGTTTGGCTATAATGGGAGGTTCAGGTGCTGGTAAAACTACATTGTTGGATTTTAAACCTTTGGACCAATTRGCTAAGACTTTGACCACTGTTCCTGAATTGAATGAAATTATTGGGCAAGATTTAGTTGACGAATTTGTCAGTGGTATTAAACTACCAGCAGAAGTTGGAAGTCAAGATGATGTTAACAATAGAAAATTGTTGCAAAAAGTGTTTGGTAAATTAATGAACACTGATGATGACGTTATAAAACAACAAACAGCTAAATTACTTGAAAGAACAGACAGAGAACCTCAAGTGTTCAAGGATATTGATTCTAGATTACCGGAGTTAATACAAAGATTAAACAAACAATTTCCTAATGACATCGGATTATTTTGTGGATGTCTCTTATTGAACCACGTTGMTTGAAAAAATTATCAATTTCCGTGTTGGATAAACAGAGATTGACTGAAAAATTCAATAAATTGGATAAATCCATCAAAGATAATTTGAAGGCTAAACAAAAAGAAGAAACCAAAAAAACTTTAGATGTGGTTAATAATTGGTTGAATGATAAAGAAAATGCTTCATCATTTTTGGTTGCTCACGTTCCAATTACTGCTAATGCCAAGGCAATCACTGAAGCCATTAATTTGATTAAAAAGCAAGATAAAACCAAATCAATTTATTTATTGACTGGTGAAACCGATAAAGTTGCTCATGGATGTTATGTTAGTGATGAAGCCATTGCCAAGGGTATTAATGCGAAAATCAAACCTTTTGAAGCACGAGCAATTAACTGGTCCACGGATCTTAATGCTGAGGTACATATTGAGCATTATATAAATATATTCAATTATGCACGATCATCTTGGGAGCCATTGGTTGAAAGTTGGCCAATAGCAGTTTACATGTCAAAATCCCGACACCCAAAGCCTCAATTATTAGTAGAGGTGATTTCTAGACAGRTAGCTCAAGTGACGCTTACATCCAAAGCGGTAGCATTGCTATCTCAAGTATCCGATTTGATTACTTCCGRAGAAAAATTAAAACCAAGAGGTGAAGATTACCCATACGTTATAGTGAATGARACTGGTTTAGATTTGGAAGTTTGGAATGATGCAAAWGAATCCGAAACCAATGGAGTTCCAATTGTTTTACGTGCTGGTAAAGCTTTAGATGAAAGTAAAGTTGAAATTAGAATTCAATTTAAACCAGTCGCCAAGGGGATGTTTAAAGAAATTCAAAGAAATGAATTAGTTATTAGAGTACAACCAAATGAAGCCATTTATTTAAAAATTAATTCCAAAATCCCTGGAATTTCTACTGAAACTTCATTAACTGATTTAGATTTAACTTATGCTACTCGTTATTCTAAAGAYTTTTGGATTCCTGAAGCTTATGAAGCATTAATTAGAGATTGTTATTTAGGTAATCATTCTAATTTTGTTAGAGATGATGAATTGGATGTTTCTTGGAAATTATTTACTCCATTATTGAAT

>Mi9_eye_20/08/2012

AATTATTGAARTGGAATGATATTCCATTGGCTCCACCAGACAARATTTTGGGTATTTCTGAAGCTTATAACAATGATTCTAACCCTCAAAAAATCAATTTGGGGGTTGGTGCTTATAGAGATAATTCYGGTAAACCAATTATTTTCCCATCAGTTAAAAAAGCTGAAGAAATTTTATTGGGTAAAGAAACTGAAAAGGAATATACTGCCATTGTTGGTTCCAAAAATTTCCAATCAATTGTGAAAAATTTCATTTTCAACAATTCTAATAAAGATGCCAATGGTAAACAATTAATTGATGATGGTAGAATTGTTACTGCTCAAACCATCTCTGGTACTGGATCACTTAAACCTCTTATGGTGAAATTTTTGCTAAACATAGAGAACCAAATTTGGAAATTATTCGTGAGGTTGTTGATTCCAAACATATTGTTTTTGATGTGTTGGCACAATTCTTAATCAATCCAGACCCATGGGTTGCCATTGCTGCCGCTGAAGTTTATGTCAGACGTTCATACCGTGCTTATGATTTGGGTAAAATTGAATATCATGTTAATGACAGACTTCCTATTGTTGAATGGAAATTCAAGTTGGCTAATATGGGAGCCGCTGGTGTAAACGATGCTCAACAGGCTGCTGCTGCCGGTGGCGATGATTCGACATCTATGAAACATGCAGCTTCTGTGTCTGATTTGACCTTTGTTGTTGATTATTCATRAAAGCGAAATCCCAGGATACACTCTCCCCGATAATCCAAAGTTCACCCTTGGTAATTTGTTTGTAATAATTGGAGTCTTGTTGGTTTGTATTTTAGCTGTTGTCTCTCTTTTGAGAAATATTAGTGAGTCGGCCTTGTTCAAGAAGAATGGGTATGAACCGTTGGATTCGGATCCTAGTGTCATGAACCTAAACTTCGAGCCTACAACATTGTCCTTTGAAGATATTAAATATGAGGTTACTGGTGGTCGACAAATTTTAAATGGAGTCTTTGGGTTTGTAAAACCAAGAGAATGTTTGGCTATAATGGGAGGTTCAGGTGCTGGTAAAACTACATTGTTGGATTTTAAACCTTTGGACCAATTGGCTAAAACTTTGGCTACTGTTCCTGAATTGAATGAAATTATTGGTCAAGAATTAGTTGACGAATTTRTCAGTGGTATTAAACTACCAGCAGAAGTTGGAAGTCAAGATGATGTTAACAATAGAAAATTGTTGCAAAAAGTGTTTGGTAAATTAATGAACACTGATGATGACGTTATAAAACAACAAACAGCTAAATTACTTGAAAGAACAGACAGAGAACCTCAAGTGTTCAAGGATATTGATTCTAGATTACCGGAGTTAATACAAAGATTAAACAAACAATTTCCTAATGACATCGGATTATTTTGTGGATGTCTCTTATTGAACCACGTTGMTTGAAAAAATTATCAATTTCCGTGTTGGATAAACARAGATTGACTGAAAAATTCAATAAATTGGATAAATCCATCAAAGATAATTTGAAGGCTAAACAAAAAGAAGAAACCAAAAAAACTTTAGATGTGGTTAAYAATTGGTTGAATGATAAAGAAAATKCTTCATCATTTTTGGTTGCTCACGTTCCAATTACTGCTAATGCCAAGGCAATCACTGAAGCCATTAATTTGATTAAAAAGCAAGATAAAACCAAATCAATTTATTTATTGACTGGTGAAACCGATAAAGTTGCTCATGGATGTTATGTTAGTGATGAAGCCATTGCCAAGGGTATTAATGCGAAAATCAAACCTTTTGAAGCACGAGCAATTAACTGGTCCACGGATCTTAATGCTGAGGTACATATTGAGCATTATATAAATATATTCAATTATGCACGATCATCTTGGGAGCCATTGGTTGAAAGTTGGCCAATAGCAGTTTACATGTCAAAATCCCGACACCCAAAGCCTCAATTATTAGTAGAGGTGATTTCTAGACAGGTAGCYCAAGTGACGCTTACATCCAAAGCAGTAGCATTGCTATCTCAAGTATCCGATTTGATTACTTCCRGAGAAAAATTAAAACCAAGAGGTGAAGATTACCCATACGTTATAGTGAATGAGACTGGTTTAGATTTGGAAGTTTGGAATGATGCAAAWGAATCCGAAACCAATGGAGTTCCAATTGTTTTACGTGCTGGTAAAGCTTTAGATGAAAGTAAAGTTGAAATTAGAATTCAATTTAAACCAGTCGCCAAGGGGATGTTTAAAGAAATTCAAAGAAATGAATTAGTTATTAGAGTACAACCAAATGAAGCCATTTATTTAAAAATTAATTCCAAAATCCCTGGRATTTCTACTGAAACTTCATTAACTGATTTAGATTTAACTTATGCTACTCGTTATTCTAAAGATTTTTGGATTCCTGAAGCTTATGAAGCATTAATTAGAGATTGTTATTTAGGTAATCATTCTAATTTTGTTAGAGATGATGAATTGGATGTTTCTTGGAAATTATTTACTCCATTATTGAAT

>Mi9_oral_20/08/2012

AATTATTGAAATGGAATGATATTCCATTGGCTCCACCAGACAAAATTTTGGGTATTTCTGAAGCTTATAACAATGATTCTAACCCTCAAAAAATCAATTTGGGGGTTGGTGCTTATAGAGATAATTCCGGTAAACCAATTATTTTCCCATCAGTTAAAAAAGCTGAAGAAATTTTATTGGGTAAAGAAACTGAAAAGGAATATACTGCCATTGTTGGTTCCAAAAATTTCCAATCAATTGTGAAAAATTTCATTTTCAACAATTCTAATAAAGATGCCAATGGTAAACAATTAATTGATGATGGTAGAATTGTTACTGCTCAAACCATCTCTGGTACTGGATCACTTAAACCTCTTATGGTGAAATTTTTGCTAAACATAGAGAACCAAATTTGGAAATTATTCGTGAGGTTGTTGATTCCAAACATATTGTTTTTGATGTGTTGGCACAATTCTTAATCAATCCAGACCCATGGGTTGCCATTGCTGCCGCTGAAGTTTATGTCAGACGTTCATACCGTGCTTATGATTTGGGTAAAATTGAATATCATGTTAATGACAGACTTCCTATTGTTGAATGGAAATTCAAGTTGGCTAATATGGGAGCYGCTGGTGTAAACGATGCTCAACAGGCTGCTGCTGCCGGTGGCGATGATTCGACATCTATGAAACATGCAGCTTCTGTGTCTGATTTGACCTTTGTTGTTGATTATTCATAAAAGYGAAATYCCAGGATACACTCTCCCCGATAATCCAAAGTTCACCCTTGGTAATTTGTTTGTAATAATTGGRGTCTTGTTGGTTTGTRTTTTAGCTGTTGTCTCTCTTTTGAGAAATATTAGTGAGTCRGCCTTGTTCAAGAAGAATGGGTATGAACCGTTGGATTCRGATCCTAGTGTCATGAACCWAAACTTYGAGCCTACAACATTGTCCTTTGAAGATATTAAATATGAGGTTACTGGTGGTCGACAAATTTTAAATGGAGTCTTTGGGTTTGTAAAACCAAGAGAATGTTTGGCTATAATGGGAGGTTCAGGTGCTGGTAAAACTACATTGTTGGATTTTAAACCTTTGGACCAATTRGCTAAGACTTTGACCACTGTTCCTGAATTGAATGAAATTATTGGGCAAGATTTAGTTGACGAATTTGTCAGTGGTATTAAACTACCAGCAGAAGTTGGAAGTCAAGATGATGTTAACAATAGAAAATTGTTGCAAAAAGTGTTTGGTAAATTAATGAACACTGATGATGACGTTATAAAACAACAAACAGCTAAATTACTTGAAAGAACAGACAGAGAACCTCAAGTGTTCAAGGATATTGATTCTAGATTACCGGAGTTAATACAAAGATTAAACAAACAATTTCCTAATGACATCGGATTATTTTGTGGATGTCTCTTATTGAACCACGTTGMTTGAAAAAATTATCAATTTCCGTGTTGGATAAACAGAGATTGACTGAAAAATTCAATAAATTGGATAAATCCATCAAAGATAATTTGAAGGCTAAACAAAAAGAAGAAACCAAAAAAACTTTAGATGTGGTTAATAATTGGTTGAATGATAAAGAAAATGCTTCATCATTTTTGGTTGCTCACGTTCCAATTACTGCTAATGCCAAGGCAATCACTGAAGCCATTAATTTGATTAAAAAGCAAGATAAAACCAAATCAATTTATTTATTGACTGGTGAAACCGATAAAGTTGCTCATGGATGTTATGTTAGTGATGAAGCCATTGCCAAGGGTATTAATGCGAAAATCAAACCTTTTGAAGCACGAGCAATTAACTGGTCCACGGATCTTAATGCTGAGGTACATATTGAGCATTATATAAATATATTCAATTATGCACGATCATCTTGGGAGCCATTGGTTGAAAGTTGGCCAATAGCAGTTTACATGTCAAAATCCCGACACCCAAAGCCTCAATTATTAGTAGAGGTGATTTCTAGACAGRTAGCTCAAGTGACGCTTACATCCAAAGCGGTAGCATTGCTATCTCAAGTATCCGATTTGATTACTTCCGRAGAAAAATTAAAACCAAGAGGTGAAGATTACCCATACGTTATAGTGAATGARACTGGTTTAGATTTGGAAGTTTGGAATGATGCAAAWGAATCCGAAACCAATGGAGTTCCAATTGTTTTACGTGCTGGTAAAGCTTTAGATGAAAGTAAAGTTGAAATTAGAATTCAATTTAAACCAGTCGCCAAGGGGATGTTTAAAGAAATTCAAAGAAATGAATTAGTTATTAGAGTACAACCAAATGAAGCCATTTATTTAAAAATTAATTCCAAAATCCCTGGAATTTCTACTGAAACTTCATTAACTGATTTAGATTTAACTTATGCTACTCGTTATTCTAAAGAYTTTTGGATTCCTGAAGCTTATGAAGCATTAATTAGAGATTGTTATTTAGGTAATCATTCTAATTTTGTTAGAGATGATGAATTGGATGTTTCTTGGAAATTATTTACTCCATTATTGAAT

>Mi9_rectal_20/08/2012

AATTATTGAARTGGAATGATATTCCATTGGCTCCACCAGACAARATTTTGGGTATTTCTGAAGCTTATAACAATGATTCTAACCCTCAAAAAATCAATTTGGGGGTTGGTGCTTATAGAGATAATTCYGGTAAACCAATTATTTTCCCATCAGTTAAAAAAGCTGAAGAAATTTTATTGGGTAAAGAAACTGAAAAGGAATATACTGCCATTGTTGGTTCCAAAAATTTCCAATCAATTGTGAAAAATTTCATTTTCAACAATTCTAATAAAGATGCCAATGGTAAACAATTAATTGATGATGGTAGAATTGTTACTGCTCAAACCATCTCTGGTACTGGATCACTTAAACCTCTTATGGTGAAATTTTTGCTAAACATAGAGAACCAAATTTGGAAATTATTCGTGAGGTTGTTGATTCCAAACATATTGTTTTTGATGTGTTGGCACAATTCTTAATCAATCCAGACCCATGGGTTGCCATTGCTGCCGCTGAAGTTTATGTCAGACGTTCATACCGTGCTTATGATTTGGGTAAAATTGAATATCATGTTAATGACAGACTTCCTATTGTTGAATGGAAATTCAAGTTGGCTAATATGGGAGCCGCTGGTGTAAACGATGCTCAACAGGCTGCTGCTGCCGGTGGCGATGATTCGACATCTATGAAACATGCAGCTTCTGTGTCTGATTTGACCTTTGTTGTTGATTATTCATRAAAGCGAAATCCCAGGATACACTCTCCCCGATAATCCAAAGTTCACCCTTGGTAATTTGTTTGTAATAATTGGAGTCTTGTTGGTTTGTATTTTAGCTGTTGTCTCTCTTTTGAGAAATATTAGTGAGTCGGCCTTGTTCAAGAAGAATGGGTATGAACCGTTGGATTCGGATCCTAGTGTCATGAACCTAAACTTCGAGCCTACAACATTGTCCTTTGAAGATATTAAATATGAGGTTACTGGTGGTCGACAAATTTTAAATGGAGTCTTTGGGTTTGTAAAACCAAGAGAATGTTTGGCTATAATGGGAGGTTCAGGTGCTGGTAAAACTACATTGTTGGATTTTAAACCTTTGGACCAATTGGCTAAAACTTTGGCTACTGTTCCTGAATTGAATGAAATTATTGGTCAAGAATTAGTTGACGAATTTRTCAGTGGTATTAAACTACCAGCAGAAGTTGGAAGTCAAGATGATGTTAACAATAGAAAATTGTTGCAAAAAGTGTTTGGTAAATTAATGAACACTGATGATGACGTTATAAAACAACAAACAGCTAAATTACTTGAAAGAACAGACAGAGAACCTCAAGTGTTCAAGGATATTGATTCTAGATTACCGGAGTTAATACAAAGATTAAACAAACAATTTCCTAATGACATCGGATTATTTTGTGGATGTCTCTTATTGAACCACGTTGMTTGAAAAAATTATCAATTTCCGTGTTGGATAAACARAGATTGACTGAAAAATTCAATAAATTGGATAAATCCATCAAAGATAATTTGAAGGCTAAACAAAAAGAAGAAACCAAAAAAACTTTAGATGTGGTTAAYAATTGGTTGAATGATAAAGAAAATKCTTCATCATTTTTGGTTGCTCACGTTCCAATTACTGCTAATGCCAAGGCAATCACTGAAGCCATTAATTTGATTAAAAAGCAAGATAAAACCAAATCAATTTATTTATTGACTGGTGAAACCGATAAAGTTGCTCATGGATGTTATGTTAGTGATGAAGCCATTGCCAAGGGTATTAATGCGAAAATCAAACCTTTTGAAGCACGAGCAATTAACTGGTCCACGGATCTTAATGCTGAGGTACATATTGAGCATTATATAAATATATTCAATTATGCACGATCATCTTGGGAGCCATTGGTTGAAAGTTGGCCAATAGCAGTTTACATGTCAAAATCCCGACACCCAAAGCCTCAATTATTAGTAGAGGTGATTTCTAGACAGGTAGCYCAAGTGACGCTTACATCCAAAGCAGTAGCATTGCTATCTCAAGTATCCGATTTGATTACTTCCRGAGAAAAATTAAAACCAAGAGGTGAAGATTACCCATACGTTATAGTGAATGAGACTGGTTTAGATTTGGAAGTTTGGAATGATGCAAAWGAATCCGAAACCAATGGAGTTCCAATTGTTTTACGTGCTGGTAAAGCTTTAGATGAAAGTAAAGTTGAAATTAGAATTCAATTTAAACCAGTCGCCAAGGGGATGTTTAAAGAAATTCAAAGAAATGAATTAGTTATTAGAGTACAACCAAATGAAGCCATTTATTTAAAAATTAATTCCAAAATCCCTGGRATTTCTACTGAAACTTCATTAACTGATTTAGATTTAACTTATGCTACTCGTTATTCTAAAGATTTTTGGATTCCTGAAGCTTATGAAGCATTAATTAGAGATTGTTATTTAGGTAATCATTCTAATTTTGTTAGAGATGATGAATTGGATGTTTCTTGGAAATTATTTACTCCATTATTGAAT

>Mi9_skin_20/08/2012

AATTATTGAARTGGAATGATATTCCATTGGCTCCACCAGACAARATTTTGGGTATTTCTGAAGCTTATAACAATGATTCTAACCCTCAAAAAATCAATTTGGGGGTTGGTGCTTATAGAGATAATTCYGGTAAACCAATTATTTTCCCATCAGTTAAAAAAGCTGAAGAAATTTTATTGGGTAAAGAAACTGAAAAGGAATATACTGCCATTGTTGGTTCCAAAAATTTCCAATCAATTGTGAAAAATTTCATTTTCAACAATTCTAATAAAGATGCCAATGGTAAACAATTAATTGATGATGGTAGAATTGTTACTGCTCAAACCATCTCTGGTACTGGATCACTTAAACCTCTTATGGTGAAATTTTTGCTAAACATAGAGAACCAAATTTGGAAATTATTCGTGAGGTTGTTGATTCCAAACATATTGTTTTTGATGTGTTGGCACAATTCTTAATCAATCCAGACCCATGGGTTGCCATTGCTGCCGCTGAAGTTTATGTCAGACGTTCATACCGTGCTTATGATTTGGGTAAAATTGAATATCATGTTAATGACAGACTTCCTATTGTTGAATGGAAATTCAAGTTGGCTAATATGGGAGCCGCTGGTGTAAACGATGCTCAACAGGCTGCTGCTGCCGGTGGCGATGATTCGACATCTATGAAACATGCAGCTTCTGTGTCTGATTTGACCTTTGTTGTTGATTATTCATRAAAGCGAAATCCCAGGATACACTCTCCCCGATAATCCAAAGTTCACCCTTGGTAATTTGTTTGTAATAATTGGAGTCTTGTTGGTTTGTATTTTAGCTGTTGTCTCTCTTTTGAGAAATATTAGTGAGTCGGCCTTGTTCAAGAAGAATGGGTATGAACCGTTGGATTCGGATCCTAGTGTCATGAACCTAAACTTCGAGCCTACAACATTGTCCTTTGAAGATATTAAATATGAGGTTACTGGTGGTCGACAAATTTTAAATGGAGTCTTTGGGTTTGTAAAACCAAGAGAATGTTTGGCTATAATGGGAGGTTCAGGTGCTGGTAAAACTACATTGTTGGATTTTAAACCTTTGGACCAATTGGCTAAAACTTTGGCTACTGTTCCTGAATTGAATGAAATTATTGGTCAAGAATTAGTTGACGAATTTRTCAGTGGTATTAAACTACCAGCAGAAGTTGGAAGTCAAGATGATGTTAACAATAGAAAATTGTTGCAAAAAGTGTTTGGTAAATTAATGAACACTGATGATGACGTTATAAAACAACAAACAGCTAAATTACTTGAAAGAACAGACAGAGAACCTCAAGTGTTCAAGGATATTGATTCTAGATTACCGGAGTTAATACAAAGATTAAACAAACAATTTCCTAATGACATCGGATTATTTTGTGGATGTCTCTTATTGAACCACGTTGMTTGAAAAAATTATCAATTTCCGTGTTGGATAAACARAGATTGACTGAAAAATTCAATAAATTGGATAAATCCATCAAAGATAATTTGAAGGCTAAACAAAAAGAAGAAACCAAAAAAACTTTAGATGTGGTTAAYAATTGGTTGAATGATAAAGAAAATKCTTCATCATTTTTGGTTGCTCACGTTCCAATTACTGCTAATGCCAAGGCAATCACTGAAGCCATTAATTTGATTAAAAAGCAAGATAAAACCAAATCAATTTATTTATTGACTGGTGAAACCGATAAAGTTGCTCATGGATGTTATGTTAGTGATGAAGCCATTGCCAAGGGTATTAATGCGAAAATCAAACCTTTTGAAGCACGAGCAATTAACTGGTCCACGGATCTTAATGCTGAGGTACATATTGAGCATTATATAAATATATTCAATTATGCACGATCATCTTGGGAGCCATTGGTTGAAAGTTGGCCAATAGCAGTTTACATGTCAAAATCCCGACACCCAAAGCCTCAATTATTAGTAGAGGTGATTTCTAGACAGGTAGCYCAAGTGACGCTTACATCCAAAGCAGTAGCATTGCTATCTCAAGTATCCGATTTGATTACTTCCRGAGAAAAATTAAAACCAAGAGGTGAAGATTACCCATACGTTATAGTGAATGAGACTGGTTTAGATTTGGAAGTTTGGAATGATGCAAAWGAATCCGAAACCAATGGAGTTCCAATTGTTTTACGTGCTGGTAAAGCTTTAGATGAAAGTAAAGTTGAAATTAGAATTCAATTTAAACCAGTCGCCAAGGGGATGTTTAAAGAAATTCAAAGAAATGAATTAGTTATTAGAGTACAACCAAATGAAGCCATTTATTTAAAAATTAATTCCAAAATCCCTGGRATTTCTACTGAAACTTCATTAACTGATTTAGATTTAACTTATGCTACTCGTTATTCTAAAGATTTTTGGATTCCTGAAGCTTATGAAGCATTAATTAGAGATTGTTATTTAGGTAATCATTCTAATTTTGTTAGAGATGATGAATTGGATGTTTCTTGGAAATTATTTACTCCATTATTGAAT

>Mi10_oral_10/12/2012

AATTATTGAAATGGAATGATATTCCATTGGCCCCACCAGACAAGATTTTGGGTATTTCTGAAGCTTATAACAATGATTCTAACCCTCAAAAAATCAATTTGGGGGTTGGTGCTTATAGAGATAATTCTGGTAAACCAATTATTTTCCCATCAGTTAAAAAAGCTGAAGAAATTTTATTGGGTAAAGAAACTGAAAAGGAATATACTGCCATTGTTGGTTCCAAAAATTTCCAATCAATTGTGAAAAATTTCATTTTCAACAATTCTAATAAAGATGCCAATGGTAAACAATTAATTGATGATGGTAGAATTGTTACTGCTCAAACCATCTCTGGTACTGGATCACTTAAACCTCTTATGGTGAAATTTTTGCTAAACATAGAGAACCAAATTTGGAAATTATTCGTGARGTTGTTGATTCCAAACATATTGTTTTTGATGTGTTGGCACAATTCTTAATCAATCCAGACCCATGGGTTGCCATTGCTGCCGCTGAAGTTTATGTCAGACGTTCATACCGTGCTTATGATTTGGGTAAAATTGAATATCATGTTAATGACAGACTTCCTATTGTTGAATGGAAATTCAAGTTGGCTAATATGGGAGCCGCTGGTGTAAACGATGCTCAACAGGCTGCTGCTGCCGGTGGCGATGATTCGACATCTATGAAACATGCAGCTTCTGTGTCTGATTTGACCTTTGTTGTTGATTATTCATAAAAGCGAAATCCCAGGATACACTCTCCCCGATAATCCAAAGTTCACCCTTGGTAATTTGTTTGTAATAATTGGAGTCTTGTTGGTTTGTATTTTAGCTGTTGTCTCTCTTTTGAGAAATATTAGTGAGTCGGCCTTGTTCAAGAAGAATGGGTATGAACCGTTGGATTCGGATCCTAGTGTCATGAACCTAAACTTCGAGCCTACAACATTGTCCTTTGAAGATATTAAATATGAGGTTACTGGTGGTCGACAAATTTTAAATGGAGTCTTTGGGTTTGTAAAACCAAGAGAATGTTTGGCTATAATGGGAGGTTCAGGTGCTGGTAAAACTACATTGTTGGATTTTAAACCTTTGGACCAATTGGCTAARACTTTGRCYACTGTTCCTGAATTGAATGAAATTATTGGKCAAGAWTTAGTTGACGAATTTRTCAGTGGTATTAAACTACCAGCAGAAGTTGGAAGTCAAGATGATGTTAACAATAGAAAATTGTTGCAAAAAGTGTTTGGTAAATTAATGAACACTGATGATGACGTTATAAAACAACAAACAGCTAAATTACTTGAAAGAACAGAMAGRGAACCTCAAGTGTTCAAGGATATTGATTCTAGATTACCRGAGTTAATACAARGATTAAACAAACAATTTCCTAATGACATCGGATTATTTTGTGGATGTCTCTTATTGAACCACGTTGCTTGAAAAAATTATCAATTTCCGTGTTGGATAAACAGAGATTGACTGAAAAATTCAATAAATTGGATAAATCCATTAAAGATAATTTGAAGGCTAAACAAAAAGAAGAAACCAAAAAGACTTTAGATGTGGTTAACAATTGGTTGAATGATAAAGAAAATGCTTCATCATTTTTGGTTGCTCACGTTCCAATTACTGCTAATGCCAAGGCAATCACTGAAGCCATTAATTTGATTAAAAAGCAAGATAAAACCAAATCAATTTATTTATTGACTGGTGAAACCGATAAAGTTGCTCATGGATGTTATGTTAGTGATGAAGCCATTGTCAAGGGTATTAATGCGAAAATCAAACCTTTTGAAGCACGAGCAATTAACTGGTCCACGGATCTTAATGCTGAGGTACATATTGAGCATTATATAAATATATTCAATTATGCACGATCATCTTGGGAGCCATTGGTTGAAAGTTGGCCAATAGCAGTTTACATGTCAAAATCCCGACACCCAAAGCCTCAATTATTAGTAGAGGTGATTTCTAGACAGGTAGCCCAAGTGACGCTTACATCCAAAGCAGTAGCATTGCTATCTCAAGTATCCGATTTGATTACTTCCGGAGAAAAATTAAAACCAAGAGGTGAAGATTACCCATACGTTATAGTGAATGAGACTGGTTTAGATTTGGAAGTTTGGAATGATGCAAAAGAATCCGAAACCAATGGAGTTCCAATTGTTTTACGTGCTGGTAAAGCTTTAGATGAAAGTAAAGTTGAAATTAGAATTCAATTTAAACCAGTCGCCAAGGGGATGTTTAAAGAAATTCAAAGAAATGAATTAGTTATTAGAGTACAACCAAATGAAGCCATTTATTTAAAAATTAATTCCAAAATCCCTGGAATTTCTACTGAAACTTCATTAACTGATTTAGATTTAACTTATGCTACTCGTTATTCTAAAGAYTTTTGGATTCCTGAAGCTTATGAAGCATTAATTAGAGATTGTTATTTAGGTAATCATTCTAATTTTGTTAGAGATGATGAATTGGATGTTTCTTGGAAATTATTTACTCCATTATTGAAT

>Mi10_rectal_10/12/2012

AATTATTGAAATGGAATGATATTCCATTGGCTCCACCAGACAARATTTTGGGTATTTCTGAAGCTTATAACAATGATTCTAACCCTCAAAAARTCAATTTGGGGGTTGGTGCTTATAGAGATAATTCYGGTAAACCAATTATTTTCCCATCAGTTAAAAAAGCTGAAGAAATTTTATTGGGTAAAGAAACTGAAAAGGAATATACTGCCATTGTTGGTTCCAAAAATTTCCAATCAATTGTGAAAAATTTCATTTTCAACAATTCTAATAAAGATGCCAATGGTAAACAATTAATTGATGATGGTAGAATTGTTACTGCTCAAACCATCTCTGGTACTGGATCACTTAAACCTCTTATGGTGAAATTTTTGCTAAACATAGAGAACCAAATTTGGAAATTATTCGTGAGGTTGTTGATTCCAAACATATTGTTTTTGATGTGTTGGCACAATTCTTAATCAATCCAGACCCATGGGTTGCCATTGCTGCCGCTGAAGTTTATGTCAGACGTTCATACCGTGCTTATGATTTGGGTAAAATTGAATATCATGTTAATGACAGACTTCCTATTGTTGAATGGAAATTCAAGTTGGCTAATATGGGAGCCGCTGGTGTAAACGATGCTCAACAGGCTGCTGCTGCCGGTGGCGATGATTCGACATCTATGAAACATGCAGCTTCTGTGTCTGATTTGACCTTTGTTGTTGATTATTCATAAAAGYGAAATYCCAGGATACACTCTCCCCGATAATCCAAAGTTCACCCTTGGTAATTTGTTTGTAATAATTGGRGTCTTGTTGGTTTGTRTTTTAGCTGTTGTCTCTCTTTTGAGAAATATTAGTGAGTCRGCCTTGTTCAAGAAGAATGGGTATGAACCGTTGGATTCRGATCCTAGTGTCATGAACCWAAACTTYGAGCCTACAACATTGTCCTTTGAAGATATTAAATATGAGGTTACTGGTGGTCGACAAATTTTAAATGGAGTCTTTGGGTTTGTAAAACCAAGAGAATGTTTGGCTATAATGGGAGGTTCAGGTGCTGGTAAAACTACATTGTTGGATTTTAAACCTTTGGACCAATTGGCTAARACTTTGRCYACTGTTCCTGAATTGAATGAAATTATTGGKCAAGAWTTAGTTGACGAATTTGTCAGTGGTATTAAACTACCAGCAGAAGTTGGAAGTCAAGATGATGTTAACAATAGAAAATTGTTGCAAAAAGTGTTTGGTAAATTAATGAACACTGATGATGACGTTATAAAACAACAAACAGCTAAATTACTTGAAAGAACAGAMAGRGAACCTCAAGTGTTCAAGGATATTGATTCTAGATTACCRGAGTTAATACAARGATTAAACAAACAATTTCCTAATGACATCGGATTATTTTGTGGATGTCTCTTATTGAACCACGTTGMTTGAAAAAATTATCAATTTCCGTGTTGGATAAACARAGATTGACTGAAAAATTCAATAAATTGGATAAATCCATYAAAGATAATTTGAAGGCTAAACAAAAAGAAGAAACCAAAAARACTTTAGATGTGGTTAAYAATTGGTTGAATGATAAAGAAAATGCTTCATCATTTTTGGTTGCTCACGTTCCAATTACTGCTAATGCCAAGGCAATCACTGAAGCCATTAATTTGATTAAAAAGCAAGATAAAACCAAATCAATTTATTTATTGACTGGTGAAACCGATAAAGTTGCTCATGGATGTTATGTTAGTGATGAAGCCATTGYCAAGGGTATTAATGCGAAAATCAAACCTTTTGAAGCACGAGCAATTAACTGGTCCACGGATCTTAATGCTGAGGTACATATTGAGCATTATATAAATATATTCAATTATGCACGATCATCTTGGGAGCCATTGGTTGAAAGTTGGCCAATAGCAGTTTACATGTCAAAATCCCGACACCCAAAGCCTCAATTATTAGTAGAGGTGATTTCTAGACAGGTAGCTCAAGTGACGCTTACATCCAAAGCGGTAGCATTGCTATCTCAAGTATCCGATTTGATTACTTCCGAAGAAAAATTAAAACCAAGAGGTGAAGATTACCCATACGTTATAGTGAATGAGACTGGTTTAGATTTGGAAGTTTGGAATGATGCAAATGAATCCGAAACCAATGGAGTTCCAATTGTTTTACGTGCTGGTAAAGCTTTAGATGAAAGTAAAGTTGAAATTAGAATTCAATTTAAACCAGTCGCCAAGGGGATGTTTAAAGAAATTCAAAGAAATGAATTAGTTATTAGAGTACAACCAAATGAAGCCATTTATTTAAAAATTAATTCCAAAATCCCTGGAATTTCTACTGAAACTTCATTAACTGATTTAGATTTAACTTATGCTACTCGTTATTCTAAAGAYTTTTGGATTCCTGAAGCTTATGAAGCATTAATTAGAGATTGYTATTTAGGTAATCATTCTAATTTTGTTAGAGATGATGAATTGGATGTTTCTTGGAAATTATTTACTCCATTATTGAAT

>Mi11_urine_17/12/2012

AATTATTGAAATGGAATGATATTCCATTGGCCCCACCAGACAAGATTTTGGGTATTTCTGAAGCTTATAACAATGATTCTAACCCTCAAAAAATCAATTTGGGGGTTGGTGCTTATAGAGATAATTCTGGTAAACCAATTATTTTCCCATCAGTTAAAAAAGCTGAAGAAATTTTATTGGGTAAAGAAACTGAAAAGGAATATACTGCCATTGTTGGTTCCAAAAATTTCCAATCAATTGTGAAAAATTTCATTTTCAACAATTCTAATAAAGATGCCAATGGTAAACAATTAATTGATGATGGTAGAATTGTTACTGCTCAAACCATCTCTGGTACTGGATCACTTAAACCTCTTATGGTGAAATTTTTGCTAAACATAGAGAACCAAATTTGGAAATTATTCGTGARGTTGTTGATTCCAAACATATTGTTTTTGATGTGTTGGCACAATTCTTAATCAATCCAGACCCATGGGTTGCCATTGCTGCCGCTGAAGTTTATGTCAGACGTTCATACCGTGCTTATGATTTGGGTAAAATTGAATATCATGTTAATGACAGACTTCCTATTGTTGAATGGAAATTCAAGTTGGCTAATATGGGAGCCGCTGGTGTAAACGATGCTCAACAGGCTGCTGCTGCCGGTGGCGATGATTCGACATCTATGAAACATGCAGCTTCTGTGTCTGATTTGACCTTTGTTGTTGATTATTCATAAAAGCGAAATCCCAGGATACACTCTCCCCGATAATCCAAAGTTCACCCTTGGTAATTTGTTTGTAATAATTGGAGTCTTGTTGGTTTGTATTTTAGCTGTTGTCTCTCTTTTGAGAAATATTAGTGAGTCGGCCTTGTTCAAGAAGAATGGGTATGAACCGTTGGATTCGGATCCTAGTGTCATGAACCTAAACTTCGAGCCTACAACATTGTCCTTTGAAGATATTAAATATGAGGTTACTGGTGGTCGACAAATTTTAAATGGAGTCTTTGGGTTTGTAAAACCAAGAGAATGTTTGGCTATAATGGGAGGTTCAGGTGCTGGTAAAACTACATTGTTGGATTTTAAACCTTTGGACCAATTGGCTAARACTTTGRCYACTGTTCCTGAATTGAATGAAATTATTGGKCAAGAWTTAGTTGACGAATTTRTCAGTGGTATTAAACTACCAGCAGAAGTTGGAAGTCAAGATGATGTTAACAATAGAAAATTGTTGCAAAAAGTGTTTGGTAAATTAATGAACACTGATGATGACGTTATAAAACAACAAACAGCTAAATTACTTGAAAGAACAGAMAGRGAACCTCAAGTGTTCAAGGATATTGATTCTAGATTACCRGAGTTAATACAARGATTAAACAAACAATTTCCTAATGACATCGGATTATTTTGTGGATGTCTCTTATTGAACCACGTTGCTTGAAAAAATTATCAATTTCCGTGTTGGATAAACAGAGATTGACTGAAAAATTCAATAAATTGGATAAATCCATTAAAGATAATTTGAAGGCTAAACAAAAAGAAGAAACCAAAAAGACTTTAGATGTGGTTAACAATTGGTTGAATGATAAAGAAAATGCTTCATCATTTTTGGTTGCTCACGTTCCAATTACTGCTAATGCCAAGGCAATCACTGAAGCCATTAATTTGATTAAAAAGCAAGATAAAACCAAATCAATTTATTTATTGACTGGTGAAACCGATAAAGTTGCTCATGGATGTTATGTTAGTGATGAAGCCATTGTCAAGGGTATTAATGCGAAAATCAAACCTTTTGAAGCACGAGCAATTAACTGGTCCACGGATCTTAATGCTGAGGTACATATTGAGCATTATATAAATATATTCAATTATGCACGATCATCTTGGGAGCCATTGGTTGAAAGTTGGCCAATAGCAGTTTACATGTCAAAATCCCGACACCCAAAGCCTCAATTATTAGTAGAGGTGATTTCTAGACAGGTAGCCCAAGTGACGCTTACATCCAAAGCAGTAGCATTGCTATCTCAAGTATCCGATTTGATTACTTCCGGAGAAAAATTAAAACCAAGAGGTGAAGATTACCCATACGTTATAGTGAATGAGACTGGTTTAGATTTGGAAGTTTGGAATGATGCAAAAGAATCCGAAACCAATGGAGTTCCAATTGTTTTACGTGCTGGTAAAGCTTTAGATGAAAGTAAAGTTGAAATTAGAATTCAATTTAAACCAGTCGCCAAGGGGATGTTTAAAGAAATTCAAAGAAATGAATTAGTTATTAGAGTACAACCAAATGAAGCCATTTATTTAAAAATTAATTCCAAAATCCCTGGAATTTCTACTGAAACTTCATTAACTGATTTAGATTTAACTTATGCTACTCGTTATTCTAAAGAYTTTTGGATTCCTGAAGCTTATGAAGCATTAATTAGAGATTGTTATTTAGGTAATCATTCTAATTTTGTTAGAGATGATGAATTGGATGTTTCTTGGAAATTATTTACTCCATTATTGAAT

>Mi11_urine_17/12/2012:IND:62:72.6

AATTATTGAAATGGAATGATATTCCATTGGCTCCACCAGACAAAATTTTGGGTATTTCTGAAGCTTATAACAATGATTCTAACCCTCAAAAAATCAATTTGGGGGTTGGTGCTTATAGAGATAATTCCGGTAAACCAATTATTTTCCCATCAGTTAAAAAAGCTGAAGAAATTTTATTGGGTAAAGAAACTGAAAAGGAATATACTGCCATTGTTGGTTCCAAAAATTTCCAATCAATTGTGAAAAATTTCATTTTCAACAATTCTAATAAAGATGCCAATGGTAAACAATTAATTGATGATGGTAGAATTGTTACTGCTCAAACCATCTCTGGTACTGGATCACTTAAACCTCTTATGGTGAAATTTTTGCTAAACATAGAGAACCAAATTTGGAAATTATTCGTGARGTTGTTGATTCCAAACATATTGTTTTTGATGTGTTGGCACAATTCTTAATCAATCCAGACCCATGGGTTGCCATTGCTGCCGCTGAAGTTTATGTCAGACGTTCATACCGTGCTTATGATTTGGGTAAAATTGAATATCATGTTAATGACAGACTTCCTATTGTTGAATGGAAATTCAAGTTGGCTAATATGGGAGCYGCTGGTGTAAACGATGCTCAACAGGCTGCTGCTGCCGGTGGCGATGATTCGACATCTATGAAACATGCAGCTTCTGTGTCTGATTTGACCTTTGTTGTTGATTATTCATAAAAGYGAAATYCCAGGATACACTCTCCCCGATAATCCAAAGTTCACCCTTGGTAATTTGTTTGTAATAATTGGRGTCTTGTTGGTTTGTRTTTTAGCTGTTGTCTCTCTTTTGAGAAATATTAGTGAGTCRGCCTTGTTCAAGAAGAATGGGTATGAACCGTTGGATTCRGATCCTAGTGTCATGAACCWAAACTTYGAGCCTACAACATTGTCCTTTGAAGATATTAAATATGAGGTTACTGGTGGTCGACAAATTTTAAATGGAGTCTTTGGGTTTGTAAAACCAAGAGAATGTTTGGCTATAATGGGAGGTTCAGGTGCTGGTAAAACTACATTGTTGGATTTTAAACCTTTGGACCAATTRGCTAAGACTTTGACCACTGTTCCTGAATTGAATGAAATTATTGGGCAAGATTTAGTTGACGAATTTGTCAGTGGTATTAAACTACCAGCAGAAGTTGGAAGTCAAGATGATGTTAACAATAGAAAATTGTTGCAAAAAGTGTTTGGTAAATTAATGAACACTGATGATGACGTTATAAAACAACAAACAGCTAAATTACTTGAAAGAACAGACAGAGAACCTCAAGTGTTCAAGGATATTGATTCTAGATTACCGGAGTTAATACAAAGATTAAACAAACAATTTCCTAATGACATCGGATTATTTTGTGGATGTCTCTTATTGAACCACGTTGMTTGAAAAAATTATCAATTTCCGTGTTGGATAAACARAGATTGACTGAAAAATTCAATAAATTGGATAAATCCATYAAAGATAATTTGAAGGCTAAACAAAAAGAAGAAACCAAAAARACTTTAGATGTGGTTAAYAATTGGTTGAATGATAAAGAAAATGCTTCATCATTTTTGGTTGCTCACGTTCCAATTACTGCTAATGCCAAGGCAATCACTGAAGCCATTAATTTGATTAAAAAGCAAGATAAAACCAAATCAATTTATTTATTGACTGGTGAAACCGATAAAGTTGCTCATGGATGTTATGTTAGTGATGAAGCCATTGYCAAGGGTATTAATGCGAAAATCAAACCTTTTGAAGCACGAGCAATTAACTGGTCCACGGATCTTAATGCTGAGGTACATATTGAGCATTATATAAATATATTCAATTATGCACGATCATCTTGGGAGCCATTGGTTGAAAGTTGGCCAATAGCAGTTTACATGTCAAAATCCCGACACCCAAAGCCTCAATTATTAGTAGAGGTGATTTCTAGACAGRTAGCTCAAGTGACGCTTACATCCAAAGCGGTAGCATTGCTATCTCAAGTATCCGATTTGATTACTTCCGRAGAAAAATTAAAACCAAGAGGTGAAGATTACCCATACGTTATAGTGAATGARACTGGTTTAGATTTGGAAGTTTGGAATGATGCAAAWGAATCCGAAACCAATGGAGTTCCAATTGTTTTACGTGCTGGTAAAGCTTTAGATGAAAGTAAAGTTGAAATTAGAATTCAATTTAAACCAGTCGCCAAGGGGATGTTTAAAGAAATTCAAAGAAATGAATTAGTTATTAGAGTACAACCAAATGAAGCCATTTATTTAAAAATTAATTCCAAAATCCCTGGAATTTCTACTGAAACTTCATTAACTGATTTAGATTTAACTTATGCTACTCGTTATTCTAAAGAYTTTTGGATTCCTGAAGCTTATGAAGCATTAATTAGAGATTGTTATTTAGGTAATCATTCTAATTTTGTTAGAGATGATGAATTGGATGTTTCTTGGAAATTATTTACTCCATTATTGAAT

>Mi11_urine_17/12/2012:IND:64:100.6

AATTATTGAAATGGAATGATATTCCATTGGCTCCACCAGACAAAATTTTGGGTATTTCTGAAGCTTATAACAATGATTCTAACCCTCAAAAAATCAATTTGGGGGTTGGTGCTTATAGAGATAATTCCGGTAAACCAATTATTTTCCCATCAGTTAAAAAAGCTGAAGAAATTTTATTGGGTAAAGAAACTGAAAAGGAATATACTGCCATTGTTGGTTCCAAAAATTTCCAATCAATTGTGAAAAATTTCATTTTCAACAATTCTAATAAAGATGCCAATGGTAAACAATTAATTGATGATGGTAGAATTGTTACTGCTCAAACCATCTCTGGTACTGGATCACTTAAACCTCTTATGGTGAAATTTTTGCTAAACATAGAGAACCAAATTTGGAAATTATTCGTGARGTTGTTGATTCCAAACATATTGTTTTTGATGTGTTGGCACAATTCTTAATCAATCCAGACCCATGGGTTGCCATTGCTGCCGCTGAAGTTTATGTCAGACGTTCATACCGTGCTTATGATTTGGGTAAAATTGAATATCATGTTAATGACAGACTTCCTATTGTTGAATGGAAATTCAAGTTGGCTAATATGGGAGCYGCTGGTGTAAACGATGCTCAACAGGCTGCTGCTGCCGGTGGCGATGATTCGACATCTATGAAACATGCAGCTTCTGTGTCTGATTTGACCTTTGTTGTTGATTATTCATAAAAGYGAAATYCCAGGATACACTCTCCCCGATAATCCAAAGTTCACCCTTGGTAATTTGTTTGTAATAATTGGRGTCTTGTTGGTTTGTRTTTTAGCTGTTGTCTCTCTTTTGAGAAATATTAGTGAGTCRGCCTTGTTCAAGAAGAATGGGTATGAACCGTTGGATTCRGATCCTAGTGTCATGAACCWAAACTTYGAGCCTACAACATTGTCCTTTGAAGATATTAAATATGAGGTTACTGGTGGTCGACAAATTTTAAATGGAGTCTTTGGGTTTGTAAAACCAAGAGAATGTTTGGCTATAATGGGAGGTTCAGGTGCTGGTAAAACTACATTGTTGGATTTTAAACCTTTGGACCAATTRGCTAAGACTTTGACCACTGTTCCTGAATTGAATGAAATTATTGGGCAAGATTTAGTTGACGAATTTGTCAGTGGTATTAAACTACCAGCAGAAGTTGGAAGTCAAGATGATGTTAACAATAGAAAATTGTTGCAAAAAGTGTTTGGTAAATTAATGAACACTGATGATGACGTTATAAAACAACAAACAGCTAAATTACTTGAAAGAACAGACAGAGAACCTCAAGTGTTCAAGGATATTGATTCTAGATTACCGGAGTTAATACAAAGATTAAACAAACAATTTCCTAATGACATCGGATTATTTTGTGGATGTCTCTTATTGAACCACGTTGMTTGAAAAAATTATCAATTTCCGTGTTGGATAAACARAGATTGACTGAAAAATTCAATAAATTGGATAAATCCATYAAAGATAATTTGAAGGCTAAACAAAAAGAAGAAACCAAAAARACTTTAGATGTGGTTAAYAATTGGTTGAATGATAAAGAAAATGCTTCATCATTTTTGGTTGCTCACGTTCCAATTACTGCTAATGCCAAGGCAATCACTGAAGCCATTAATTTGATTAAAAAGCAAGATAAAACCAAATCAATTTATTTATTGACTGGTGAAACCGATAAAGTTGCTCATGGATGTTATGTTAGTGATGAAGCCATTGYCAAGGGTATTAATGCGAAAATCAAACCTTTTGAAGCACGAGCAATTAACTGGTCCACGGATCTTAATGCTGAGGTACATATTGAGCATTATATAAATATATTCAATTATGCACGATCATCTTGGGAGCCATTGGTTGAAAGTTGGCCAATAGCAGTTTACATGTCAAAATCCCGACACCCAAAGCCTCAATTATTAGTAGAGGTGATTTCTAGACAGRTAGCTCAAGTGACGCTTACATCCAAAGCGGTAGCATTGCTATCTCAAGTATCCGATTTGATTACTTCCGRAGAAAAATTAAAACCAAGAGGTGAAGATTACCCATACGTTATAGTGAATGARACTGGTTTAGATTTGGAAGTTTGGAATGATGCAAAWGAATCCGAAACCAATGGAGTTCCAATTGTTTTACGTGCTGGTAAAGCTTTAGATGAAAGTAAAGTTGAAATTAGAATTCAATTTAAACCAGTCGCCAAGGGGATGTTTAAAGAAATTCAAAGAAATGAATTAGTTATTAGAGTACAACCAAATGAAGCCATTTATTTAAAAATTAATTCCAAAATCCCTGGAATTTCTACTGAAACTTCATTAACTGATTTAGATTTAACTTATGCTACTCGTTATTCTAAAGAYTTTTGGATTCCTGAAGCTTATGAAGCATTAATTAGAGATTGTTATTTAGGTAATCATTCTAATTTTGTTAGAGATGATGAATTGGATGTTTCTTGGAAATTATTTACTCCATTATTGAAT

>Mi11_vaginal_17/12/2012

AATTATTGAAATGGAATGATATTCCATTGGCTCCACCAGACAAAATTTTGGGTATTTCTGAAGCTTATAACAATGATTCTAACCCTCAAAAAATCAATTTGGGGGTTGGTGCTTATAGAGATAATTCCGGTAAACCAATTATTTTCCCATCAGTTAAAAAAGCTGAAGAAATTTTATTGGGTAAAGAAACTGAAAAGGAATATACTGCCATTGTTGGTTCCAAAAATTTCCAATCAATTGTGAAAAATTTCATTTTCAACAATTCTAATAAAGATGCCAATGGTAAACAATTAATTGATGATGGTAGAATTGTTACTGCTCAAACCATCTCTGGTACTGGATCACTTAAACCTCTTATGGTGAAATTTTTGCTAAACATAGAGAACCAAATTTGGAAATTATTCGTGAGGTTGTTGATTCCAAACATATTGTTTTTGATGTGTTGGCACAATTCTTAATCAATCCAGACCCATGGGTTGCCATTGCTGCCGCTGAAGTTTATGTCAGACGTTCATACCGTGCTTATGATTTGGGTAAAATTGAATATCATGTTAATGACAGACTTCCTATTGTTGAATGGAAATTCAAGTTGGCTAATATGGGAGCYGCTGGTGTAAACGATGCTCAACAGGCTGCTGCTGCCGGTGGCGATGATTCGACATCTATGAAACATGCAGCTTCTGTGTCTGATTTGACCTTTGTTGTTGATTATTCATAAAAGYGAAATYCCAGGATACACTCTCCCCGATAATCCAAAGTTCACCCTTGGTAATTTGTTTGTAATAATTGGRGTCTTGTTGGTTTGTRTTTTAGCTGTTGTCTCTCTTTTGAGAAATATTAGTGAGTCRGCCTTGTTCAAGAAGAATGGGTATGAACCGTTGGATTCRGATCCTAGTGTCATGAACCWAAACTTYGAGCCTACAACATTGTCCTTTGAAGATATTAAATATGAGGTTACTGGTGGTCGACAAATTTTAAATGGAGTCTTTGGGTTTGTAAAACCAAGAGAATGTTTGGCTATAATGGGAGGTTCAGGTGCTGGTAAAACTACATTGTTGGATTTTAAACCTTTGGACCAATTRGCTAAGACTTTGACCACTGTTCCTGAATTGAATGAAATTATTGGGCAAGATTTAGTTGACGAATTTGTCAGTGGTATTAAACTACCAGCAGAAGTTGGAAGTCAAGATGATGTTAACAATAGAAAATTGTTGCAAAAAGTGTTTGGTAAATTAATGAACACTGATGATGACGTTATAAAACAACAAACAGCTAAATTACTTGAAAGAACAGACAGAGAACCTCAAGTGTTCAAGGATATTGATTCTAGATTACCGGAGTTAATACAAAGATTAAACAAACAATTTCCTAATGACATCGGATTATTTTGTGGATGTCTCTTATTGAACCACGTTGMTTGAAAAAATTATCAATTTCCGTGTTGGATAAACARAGATTGACTGAAAAATTCAATAAATTGGATAAATCCATYAAAGATAATTTGAAGGCTAAACAAAAAGAAGAAACCAAAAARACTTTAGATGTGGTTAAYAATTGGTTGAATGATAAAGAAAATGCTTCATCATTTTTGGTTGCTCACGTTCCAATTACTGCTAATGCCAAGGCAATCACTGAAGCCATTAATTTGATTAAAAAGCAAGATAAAACCAAATCAATTTATTTATTGACTGGTGAAACCGATAAAGTTGCTCATGGATGTTATGTTAGTGATGAAGCCATTGYCAAGGGTATTAATGCGAAAATCAAACCTTTTGAAGCACGAGCAATTAACTGGTCCACGGATCTTAATGCTGAGGTACATATTGAGCATTATATAAATATATTCAATTATGCACGATCATCTTGGGAGCCATTGGTTGAAAGTTGGCCAATAGCAGTTTACATGTCAAAATCCCGACACCCAAAGCCTCAATTATTAGTAGAGGTGATTTCTAGACAGRTAGCTCAAGTGACGCTTACATCCAAAGCGGTAGCATTGCTATCTCAAGTATCCGATTTGATTACTTCCGRAGAAAAATTAAAACCAAGAGGTGAAGATTACCCATACGTTATAGTGAATGARACTGGTTTAGATTTGGAAGTTTGGAATGATGCAAAWGAATCCGAAACCAATGGAGTTCCAATTGTTTTACGTGCTGGTAAAGCTTTAGATGAAAGTAAAGTTGAAATTAGAATTCAATTTAAACCAGTCGCCAAGGGGATGTTTAAAGAAATTCAAAGAAATGAATTAGTTATTAGAGTACAACCAAATGAAGCCATTTATTTAAAAATTAATTCCAAAATCCCTGGAATTTCTACTGAAACTTCATTAACTGATTTAGATTTAACTTATGCTACTCGTTATTCTAAAGAYTTTTGGATTCCTGAAGCTTATGAAGCATTAATTAGAGATTGTTATTTAGGTAATCATTCTAATTTTGTTAGAGATGATGAATTGGATGTTTCTTGGAAATTATTTACTCCATTATTGAAT

>Mi11_vaginal_17/12/2012:IND:30.2

AATTATTGAAATGGAATGATATTCCATTGGCCCCACCAGACAAGATTTTGGGTATTTCTGAAGCTTATAACAATGATTCTAACCCTCAAAAAATCAATTTGGGGGTTGGTGCTTATAGAGATAATTCTGGTAAACCAATTATTTTCCCATCAGTTAAAAAAGCTGAAGAAATTTTATTGGGTAAAGAAACTGAAAAGGAATATACTGCCATTGTTGGTTCCAAAAATTTCCAATCAATTGTGAAAAATTTCATTTTCAACAATTCTAATAAAGATGCCAATGGTAAACAATTAATTGATGATGGTAGAATTGTTACTGCTCAAACCATCTCTGGTACTGGATCACTTAAACCTCTTATGGTGAAATTTTTGCTAAACATAGAGAACCAAATTTGGAAATTATTCGTGARGTTGTTGATTCCAAACATATTGTTTTTGATGTGTTGGCACAATTCTTAATCAATCCAGACCCATGGGTTGCCATTGCTGCCGCTGAAGTTTATGTCAGACGTTCATACCGTGCTTATGATTTGGGTAAAATTGAATATCATGTTAATGACAGACTTCCTATTGTTGAATGGAAATTCAAGTTGGCTAATATGGGAGCCGCTGGTGTAAACGATGCTCAACAGGCTGCTGCTGCCGGTGGCGATGATTCGACATCTATGAAACATGCAGCTTCTGTGTCTGATTTGACCTTTGTTGTTGATTATTCATAAAAGCGAAATCCCAGGATACACTCTCCCCGATAATCCAAAGTTCACCCTTGGTAATTTGTTTGTAATAATTGGAGTCTTGTTGGTTTGTATTTTAGCTGTTGTCTCTCTTTTGAGAAATATTAGTGAGTCGGCCTTGTTCAAGAAGAATGGGTATGAACCGTTGGATTCGGATCCTAGTGTCATGAACCTAAACTTCGAGCCTACAACATTGTCCTTTGAAGATATTAAATATGAGGTTACTGGTGGTCGACAAATTTTAAATGGAGTCTTTGGGTTTGTAAAACCAAGAGAATGTTTGGCTATAATGGGAGGTTCAGGTGCTGGTAAAACTACATTGTTGGATTTTAAACCTTTGGACCAATTGGCTAARACTTTGRCYACTGTTCCTGAATTGAATGAAATTATTGGKCAAGAWTTAGTTGACGAATTTRTCAGTGGTATTAAACTACCAGCAGAAGTTGGAAGTCAAGATGATGTTAACAATAGAAAATTGTTGCAAAAAGTGTTTGGTAAATTAATGAACACTGATGATGACGTTATAAAACAACAAACAGCTAAATTACTTGAAAGAACAGAMAGRGAACCTCAAGTGTTCAAGGATATTGATTCTAGATTACCRGAGTTAATACAARGATTAAACAAACAATTTCCTAATGACATCGGATTATTTTGTGGATGTCTCTTATTGAACCACGTTGCTTGAAAAAATTATCAATTTCCGTGTTGGATAAACAGAGATTGACTGAAAAATTCAATAAATTGGATAAATCCATTAAAGATAATTTGAAGGCTAAACAAAAAGAAGAAACCAAAAAGACTTTAGATGTGGTTAACAATTGGTTGAATGATAAAGAAAATGCTTCATCATTTTTGGTTGCTCACGTTCCAATTACTGCTAATGCCAAGGCAATCACTGAAGCCATTAATTTGATTAAAAAGCAAGATAAAACCAAATCAATTTATTTATTGACTGGTGAAACCGATAAAGTTGCTCATGGATGTTATGTTAGTGATGAAGCCATTGTCAAGGGTATTAATGCGAAAATCAAACCTTTTGAAGCACGAGCAATTAACTGGTCCACGGATCTTAATGCTGAGGTACATATTGAGCATTATATAAATATATTCAATTATGCACGATCATCTTGGGAGCCATTGGTTGAAAGTTGGCCAATAGCAGTTTACATGTCAAAATCCCGACACCCAAAGCCTCAATTATTAGTAGAGGTGATTTCTAGACAGGTAGCCCAAGTGACGCTTACATCCAAAGCAGTAGCATTGCTATCTCAAGTATCCGATTTGATTACTTCCGGAGAAAAATTAAAACCAAGAGGTGAAGATTACCCATACGTTATAGTGAATGAGACTGGTTTAGATTTGGAAGTTTGGAATGATGCAAAAGAATCCGAAACCAATGGAGTTCCAATTGTTTTACGTGCTGGTAAAGCTTTAGATGAAAGTAAAGTTGAAATTAGAATTCAATTTAAACCAGTCGCCAAGGGGATGTTTAAAGAAATTCAAAGAAATGAATTAGTTATTAGAGTACAACCAAATGAAGCCATTTATTTAAAAATTAATTCCAAAATCCCTGGAATTTCTACTGAAACTTCATTAACTGATTTAGATTTAACTTATGCTACTCGTTATTCTAAAGAYTTTTGGATTCCTGAAGCTTATGAAGCATTAATTAGAGATTGTTATTTAGGTAATCATTCTAATTTTGTTAGAGATGATGAATTGGATGTTTCTTGGAAATTATTTACTCCATTATTGAAT

>Mi11_vaginal_17/12/2012:IND:55:30.7

AATTATTGAAATGGAATGATATTCCATTGGCCCCACCAGACAAGATTTTGGGTATTTCTGAAGCTTATAACAATGATTCTAACCCTCAAAAAATCAATTTGGGGGTTGGTGCTTATAGAGATAATTCTGGTAAACCAATTATTTTCCCATCAGTTAAAAAAGCTGAAGAAATTTTATTGGGTAAAGAAACTGAAAAGGAATATACTGCCATTGTTGGTTCCAAAAATTTCCAATCAATTGTGAAAAATTTCATTTTCAACAATTCTAATAAAGATGCCAATGGTAAACAATTAATTGATGATGGTAGAATTGTTACTGCTCAAACCATCTCTGGTACTGGATCACTTAAACCTCTTATGGTGAAATTTTTGCTAAACATAGAGAACCAAATTTGGAAATTATTCGTGARGTTGTTGATTCCAAACATATTGTTTTTGATGTGTTGGCACAATTCTTAATCAATCCAGACCCATGGGTTGCCATTGCTGCCGCTGAAGTTTATGTCAGACGTTCATACCGTGCTTATGATTTGGGTAAAATTGAATATCATGTTAATGACAGACTTCCTATTGTTGAATGGAAATTCAAGTTGGCTAATATGGGAGCCGCTGGTGTAAACGATGCTCAACAGGCTGCTGCTGCCGGTGGCGATGATTCGACATCTATGAAACATGCAGCTTCTGTGTCTGATTTGACCTTTGTTGTTGATTATTCATAAAAGCGAAATCCCAGGATACACTCTCCCCGATAATCCAAAGTTCACCCTTGGTAATTTGTTTGTAATAATTGGAGTCTTGTTGGTTTGTATTTTAGCTGTTGTCTCTCTTTTGAGAAATATTAGTGAGTCGGCCTTGTTCAAGAAGAATGGGTATGAACCGTTGGATTCGGATCCTAGTGTCATGAACCTAAACTTCGAGCCTACAACATTGTCCTTTGAAGATATTAAATATGAGGTTACTGGTGGTCGACAAATTTTAAATGGAGTCTTTGGGTTTGTAAAACCAAGAGAATGTTTGGCTATAATGGGAGGTTCAGGTGCTGGTAAAACTACATTGTTGGATTTTAAACCTTTGGACCAATTGGCTAARACTTTGRCYACTGTTCCTGAATTGAATGAAATTATTGGKCAAGAWTTAGTTGACGAATTTRTCAGTGGTATTAAACTACCAGCAGAAGTTGGAAGTCAAGATGATGTTAACAATAGAAAATTGTTGCAAAAAGTGTTTGGTAAATTAATGAACACTGATGATGACGTTATAAAACAACAAACAGCTAAATTACTTGAAAGAACAGAMAGRGAACCTCAAGTGTTCAAGGATATTGATTCTAGATTACCRGAGTTAATACAARGATTAAACAAACAATTTCCTAATGACATCGGATTATTTTGTGGATGTCTCTTATTGAACCACGTTGCTTGAAAAAATTATCAATTTCCGTGTTGGATAAACAGAGATTGACTGAAAAATTCAATAAATTGGATAAATCCATTAAAGATAATTTGAAGGCTAAACAAAAAGAAGAAACCAAAAAGACTTTAGATGTGGTTAACAATTGGTTGAATGATAAAGAAAATGCTTCATCATTTTTGGTTGCTCACGTTCCAATTACTGCTAATGCCAAGGCAATCACTGAAGCCATTAATTTGATTAAAAAGCAAGATAAAACCAAATCAATTTATTTATTGACTGGTGAAACCGATAAAGTTGCTCATGGATGTTATGTTAGTGATGAAGCCATTGTCAAGGGTATTAATGCGAAAATCAAACCTTTTGAAGCACGAGCAATTAACTGGTCCACGGATCTTAATGCTGAGGTACATATTGAGCATTATATAAATATATTCAATTATGCACGATCATCTTGGGAGCCATTGGTTGAAAGTTGGCCAATAGCAGTTTACATGTCAAAATCCCGACACCCAAAGCCTCAATTATTAGTAGAGGTGATTTCTAGACAGGTAGCCCAAGTGACGCTTACATCCAAAGCAGTAGCATTGCTATCTCAAGTATCCGATTTGATTACTTCCGGAGAAAAATTAAAACCAAGAGGTGAAGATTACCCATACGTTATAGTGAATGAGACTGGTTTAGATTTGGAAGTTTGGAATGATGCAAAAGAATCCGAAACCAATGGAGTTCCAATTGTTTTACGTGCTGGTAAAGCTTTAGATGAAAGTAAAGTTGAAATTAGAATTCAATTTAAACCAGTCGCCAAGGGGATGTTTAAAGAAATTCAAAGAAATGAATTAGTTATTAGAGTACAACCAAATGAAGCCATTTATTTAAAAATTAATTCCAAAATCCCTGGAATTTCTACTGAAACTTCATTAACTGATTTAGATTTAACTTATGCTACTCGTTATTCTAAAGAYTTTTGGATTCCTGAAGCTTATGAAGCATTAATTAGAGATTGTTATTTAGGTAATCATTCTAATTTTGTTAGAGATGATGAATTGGATGTTTCTTGGAAATTATTTACTCCATTATTGAAT

>Mi12_oral_27/12/2012

AATTATTGAAATGGAATGATATTCCATTGGCYCCACCAGACAAGATTTTGGGTATTTCTGAAGCTTATAACAATGATTCTAACCCTCAAAAARTCAATTTGGGGGTTGGTGCTTATAGAGATAATTCTGGTAAACCAATTATTTTCCCATCAGTTAAAAAAGCTGAAGAAATTTTATTGGGTAAAGAAACTGAAAAGGAATATACTGCCATTGTTGGTTCCAAAAATTTCCAATCAATTGTGAAAAATTTCATTTTCAACAATTCTAATAAAGATGCCAATGGTAAACAATTAATTGATGATGGTAGAATTGTTACTGCTCAAACCATYTCTGGTACTGGATCACTTAAACCTCTTATGGTGAAATTTTTGCTAAACATAGAGAACCAAATTTGGAAATTATTCGTGAGGTTGTTGATTCCAAACATATTGTTTTTGATGTGTTGGCACAATTCTTAATCAATCCAGACCCATGGGTTGCCATTGCTGCCGCTGAAGTTTATGTCAGACGTTCATACCGTGCTTATGATTTGGGTAAAATTGAATATCATGTTAATGACAGACTTCCTATTGTTGAATGGAAATTCAAGTTGGCTAATATGGGAGCYGCTGGTGTAAACGATGCTCAACAGGCTGCTGCTGCCGGTGGCGATGATTCGACATCTATGAAACATGCAGCTTCTGTGTCTGATTTGACCTTTGTTGTTGATTATTCATAAAAGCGAAATCCCAGGATACACTCTCCCCGATAATCCAAAGTTCACCCTTGGTAATTTGTTTGTAATAATTGGAGTCTTGTTGGTTTGTATTTTAGCTGTTGTCTCTCTTTTGAGAAATATTAGTGAGTCGGCCTTGTTCAAGAAGAATGGGTATGAACCGTTGGATTCGGATCCTAGTRTCATGAACCTAAACTTCGAGCCTACAACATTGTCCTTTGAAGATATTAAATATGAGGTTACTGGTGGTCGACAAATTTTAAATGGAGTCTTTGGGTTTGTAAAACCAAGAGAATGTTTGGCTATAATGGGAGGTTCAGGTGCTGGTAAAACTACATTGTTGGATTTTAAACCTTTGGACCAATTGGCTAAGACTTTGACCACTGTTCCTGAATTGAATGAAATTATTGGGCAAGATTTAGTTGACGAATTTGTCAGTGGTATTAAACTACCAGCAGAAGTTGGAAGTCAAGATGATGTTAACAATAGAAAATTGTTGCAAAAAGTGTTTGGTAAATTAATGAACACTGATGATGACGTTATAAAACAACAAACAGCTAAATTACTTGAAAGAACAGACAGAGAACCTCAAGTGTTCAAGGATATTGATTCTAGATTACCGGAGTTAATACAAAGATTAAACAAACAATTTCCTAATGACATCGGATTATTTTGTGGATGTCTCTTATTGAACCACGTTGMTTGAAAAAATTATCAATTTCCGTGTTGGATAAACARAGATTGACTGAAAAATTCAATAAATTGGATAAATCCATYAAAGATAATTTGAAGGCTAAACAAAAAGAAGAAACCAAAAARACTTTAGATGTGGTTAAYAATTGGTTGAATGATAAAGAAAATGCTTCATCATTTTTGGTTGCTCACGTTCCAATTACTGCTAATGCCAAGGCAATCACTGAAGCCATTAATTTGATTAAAAAGCAAGATAAAACCAAATCAATTTATTTATTGACTGGTGAAACCGATAAAGTTGCTCATGGATGTTATGTTAGTGATGAAGCCATTGYCAAGGGTATTAATGCGAAAATCAAACCTTTTGAAGCACGAGCAATTAACTGGTCCACGGATCTTAATGCTGAGGTACATATTGAGCATTATATAAATATATTCAATTATGCACGATCATCTTGGGAGCCATTGGTTGAAAGTTGGCCAATAGCAGTTTACATGTCAAAATCCCGACACCCAAAGCCTCAATTATTAGTAGAGGTGATTTCTAGACAGGTAGCTCAAGTGACGCTTACATCCAAAGCAGTAGCATTGCTATCTCAAGTATCCGATTTGATTACTTCCAGAGAAAAATTAAAACCAAGAGGTGAAGATTACCCATACGTTATAGTGAATGAGACTGGTTTAGATTTGGAAGTTTGGAATGATGCAAATGAATCCGAAACCAATGGAGTTCCAATTGTTTTACGTGCTGGTAAAGCTTTAGATGAAAGTAAAGTTGAAATTAGAATTCAATTTAAACCAGTCGCCAAGGGGATGTTTAAAGAAATTCAAAGAAATGAATTAGTTATTAGAGTACAACCAAATGAAGCCATTTATTTAAAAATTAATTCCAAAATCCCTGGAATTTCTACTGAAACTTCATTAACTGATTTAGATTTAACTTATGCTACTCGTTATTCTAAAGACTTTTGGATTCCTGAAGCTTATGAAGCATTAATTAGAGATTGTTATTTAGGTAATCATTCTAATTTTGTTAGAGATGATGAATTGGATGTTTCTTGGAAATTATTTACTCCATTATTGAAT

>Mi12_vaginal_31/12/2012

AATTATTGAAATGGAATGATATTCCATTGGCTCCACCAGACAAAATTTTGGGTATTTCTGAAGCTTATAACAATGATTCTAACCCTCAAAAAATCAATTTGGGGGTTGGTGCTTATAGAGATAATTCCGGTAAACCAATTATTTTCCCATCAGTTAAAAAAGCTGAAGAAATTTTATTGGGTAAAGAAACTGAAAAGGAATATACTGCCATTGTTGGTTCCAAAAATTTCCAATCAATTGTGAAAAATTTCATTTTCAACAATTCTAATAAAGATGCCAATGGTAAACAATTAATTGATGATGGTAGAATTGTTACTGCTCAAACCATCTCTGGTACTGGATCACTTAAACCTCTTATGGTGAAATTTTTGCTAAACATAGAGAACCAAATTTGGAAATTATTCGTGAGGTTGTTGATTCCAAACATATTGTTTTTGATGTGTTGGCACAATTCTTAATCAATCCAGACCCATGGGTTGCCATTGCTGCCGCTGAAGTTTATGTCAGACGTTCATACCGTGCTTATGATTTGGGTAAAATTGAATATCATGTTAATGACAGACTTCCTATTGTTGAATGGAAATTCAAGTTGGCTAATATGGGAGCYGCTGGTGTAAACGATGCTCAACAGGCTGCTGCTGCCGGTGGCGATGATTCGACATCTATGAAACATGCAGCTTCTGTGTCTGATTTGACCTTTGTTGTTGATTATTCATAAAAGYGAAATYCCAGGATACACTCTCCCCGATAATCCAAAGTTCACCCTTGGTAATTTGTTTGTAATAATTGGRGTCTTGTTGGTTTGTGTTTTAGCTGTTGTCTCTCTTTTGAGAAATATTAGTGAGTCRGCCTTGTTCAAGAAGAATGGGTATGAACCGTTGGATTCRGATCCTAGTGTCATGAACCWAAACTTYGAGCCTACAACATTGTCCTTTGAAGATATTAAATATGAGGTTACTGGTGGTCGACAAATTTTAAATGGAGTCTTTGGGTTTGTAAAACCAAGAGAATGTTTGGCTATAATGGGAGGTTCAGGTGCTGGTAAAACTACATTGTTGGATTTTAAACCTTTGGACCAATTGGCTAAGACTTTGACCACTGTTCCTGAATTGAATGAAATTATTGGGCAAGATTTAGTTGACGAATTTGTCAGTGGTATTAAACTACCAGCAGAAGTTGGAAGTCAAGATGATGTTAACAATAGAAAATTGTTGCAAAAAGTGTTTGGTAAATTAATGAACACTGATGATGACGTTATAAAACAACAAACAGCTAAATTACTTGAAAGAACAGAMAGRGAACCTCAAGTGTTCAAGGATATTGATTCTAGATTACCRGAGTTAATACAARGATTAAACAAACAATTTCCTAATGACATCGGATTATTTTGTGGATGTCTCTTATTGAACCACGTTGMTTGAAAAAATTATCAATTTCCGTGTTGGATAAACARAGATTGACTGAAAAATTCAATAAATTGGATAAATCCATYAAAGATAATTTGAAGGCTAAACAAAAAGAAGAAACCAAAAARACTTTAGATGTGGTTAAYAATTGGTTGAATGATAAAGAAAATGCTTCATCATTTTTGGTTGCTCACGTTCCAATTACTGCTAATGCCAAGGCAATCACTGAAGCCATTAATTTGATTAAAAAGCAAGATAAAACCAAATCAATTTATTTATTGACTGGTGAAACCGATAAAGTTGCTCATGGATGTTATGTTAGTGATGAAGCCATTGYCAAGGGTATTAATGCGAAAATCAAACCTTTTGAAGCACGAGCAATTAACTGGTCMACGGATCTTAATGCTGAGGTACATATTGAGCATTATATAAATATATTCAATTATGCACGATCATCTTGGGAGCCATTGGTTGAAAGTTGGCCAATAGCAGTTTACATGTCAAAATCCCGACACCCAAAGCCTCAATTATTAGTAGAGGTGATTTCTAGACAGGTAGCTCAAGTGACGCTTACATCCAAAGCRGTAGCATTGCTATCTCAAGTATCCGATTTGATTACTTCCGRAGAAAAATTAAAACCAAGAGGTGAAGATTACCCATACRTTATAGTGAATGAGACTGGTTTAGATTTGGAAGTTTGGAATGATGCAAAWGAATCCGAAACCAATGGAGTTCCAATTGTTTTACGTGCTGGTAAAGCTTTAGATGAAAGTAAAGTTGAAATTAGAATTCAATTTAAACCAGTCGCCAAGGGGATGTTTAAAGAAATTCAAAGAAATGAATTAGTTATTAGAGTACAACCAAATGAAGCCATTTATTTAAAAATTAATTCCAAAATCCCTGGAATTTCTACTGAAACTTCATTAACTGATTTAGATTTAACTTATGCTACTCGTTATTCTAAAGAYTTTTGGATTCCTGAAGCTTATGAAGCATTAATTAGAGATTGTTATTTAGGTAATCATTCTAATTTTGTTAGAGATGATGAATTGGATGTTTCTTGGAAATTATTTACTCCATTATTGAAT

>Mi13_rectal_23/01/2013

AATTATTGAAATGGAATGATATTCCATTGGCTCCACCAGACAAAATTTTGGGTATTTCTGAAGCTTATAACAATGATTCTAACCCTCAAAAAATCAATTTGGGGGTTGGTGCTTATAGAGATAATTCCGGTAAACCAATTATTTTCCCATCAGTTAAAAAAGCTGAAGAAATTTTATTGGGTAAAGAAACTGAAAAGGAATATACTGCCATTGTTGGTTCCAAAAATTTCCAATCAATTGTGAAAAATTTCATTTTCAACAATTCTAATAAAGATGCCAATGGTAAACAATTAATTGATGATGGTAGAATTGTTACTGCTCAAACCATCTCTGGTACTGGATCACTTAAACCTCTTATGGTGAAATTTTTGCTAAACATAGAGAACCAAATTTGGAAATTATTCGTGAGGTTGTTGATTCCAAACATATTGTTTTTGATGTGTTGGCACAATTCTTAATCAATCCAGACCCATGGGTTGCCATTGCTGCCGCTGAAGTTTATGTCAGACGTTCATACCGTGCTTATGATTTGGGTAAAATTGAATATCATGTTAATGACAGACTTCCTATTGTTGAATGGAAATTCAAGTTGGCTAATATGGGAGCYGCTGGTGTAAACGATGCTCAACAGGCTGCTGCTGCCGGTGGCGATGATTCGACATCTATGAAACATGCAGCTTCTGTGTCTGATTTGACCTTTGTTGTTGATTATTCATAAAAGYGAAATYCCAGGATACACTCTCCCCGATAATCCAAAGTTCACCCTTGGTAATTTGTTTGTAATAATTGGRGTCTTGTTGGTTTGTRTTTTAGCTGTTGTCTCTCTTTTGAGAAATATTAGTGAGTCRGCCTTGTTCAAGAAGAATGGGTATGAACCGTTGGATTCRGATCCTAGTGTCATGAACCWAAACTTYGAGCCTACAACATTGTCCTTTGAAGATATTAAATATGAGGTTACTGGTGGTCGACAAATTTTAAATGGAGTCTTTGGGTTTGTAAAACCAAGAGAATGTTTGGCTATAATGGGAGGTTCAGGTGCTGGTAAAACTACATTGTTGGATTTTAAACCTTTGGACCAATTRGCTAAGACTTTGACCACTGTTCCTGAATTGAATGAAATTATTGGGCAAGATTTAGTTGACGAATTTGTCAGTGGTATTAAACTACCAGCAGAAGTTGGAAGTCAAGATGATGTTAACAATAGAAAATTGTTGCAAAAAGTGTTTGGTAAATTAATGAACACTGATGATGACGTTATAAAACAACAAACAGCTAAATTACTTGAAAGAACAGACAGAGAACCTCAAGTGTTCAAGGATATTGATTCTAGATTACCGGAGTTAATACAAAGATTAAACAAACAATTTCCTAATGACATCGGATTATTTTGTGGATGTCTCTTATTGAACCACGTTGMTTGAAAAAATTATCAATTTCCGTGTTGGATAAACARAGATTGACTGAAAAATTCAATAAATTGGATAAATCCATYAAAGATAATTTGAAGGCTAAACAAAAAGAAGAAACCAAAAARACTTTAGATGTGGTTAAYAATTGGTTGAATGATAAAGAAAATGCTTCATCATTTTTGGTTGCTCACGTTCCAATTACTGCTAATGCCAAGGCAATCACTGAAGCCATTAATTTGATTAAAAAGCAAGATAAAACCAAATCAATTTATTTATTGACTGGTGAAACCGATAAAGTTGCTCATGGATGTTATGTTAGTGATGAAGCCATTGYCAAGGGTATTAATGCGAAAATCAAACCTTTTGAAGCACGAGCAATTAACTGGTCCACGGATCTTAATGCTGAGGTACATATTGAGCATTATATAAATATATTCAATTATGCACGATCATCTTGGGAGCCATTGGTTGAAAGTTGGCCAATAGCAGTTTACATGTCAAAATCCCGACACCCAAAGCCTCAATTATTAGTAGAGGTGATTTCTAGACAGRTAGCTCAAGTGACGCTTACATCCAAAGCGGTAGCATTGCTATCTCAAGTATCCGATTTGATTACTTCCGRAGAAAAATTAAAACCAAGAGGTGAAGATTACCCATACGTTATAGTGAATGARACTGGTTTAGATTTGGAAGTTTGGAATGATGCAAAWGAATCCGAAACCAATGGAGTTCCAATTGTTTTACGTGCTGGTAAAGCTTTAGATGAAAGTAAAGTTGAAATTAGAATTCAATTTAAACCAGTCGCCAAGGGGATGTTTAAAGAAATTCAAAGAAATGAATTAGTTATTAGAGTACAACCAAATGAAGCCATTTATTTAAAAATTAATTCCAAAATCCCTGGAATTTCTACTGAAACTTCATTAACTGATTTAGATTTAACTTATGCTACTCGTTATTCTAAAGAYTTTTGGATTCCTGAAGCTTATGAAGCATTAATTAGAGATTGTTATTTAGGTAATCATTCTAATTTTGTTAGAGATGATGAATTGGATGTTTCTTGGAAATTATTTACTCCATTATTGAAT

>Mi13_vaginal_23/01/2013

AATTATTGAAATGGAATGATATTCCATTGGCTCCACCAGACAAAATTTTGGGTATTTCTGAAGCTTATAACAATGATTCTAACCCTCAAAAAATCAATTTGGGGGTTGGTGCTTATAGAGATAATTCCGGTAAACCAATTATTTTCCCATCAGTTAAAAAAGCTGAAGAAATTTTATTGGGTAAAGAAACTGAAAAGGAATATACTGCCATTGTTGGTTCCAAAAATTTCCAATCAATTGTGAAAAATTTCATTTTCAACAATTCTAATAAAGATGCCAATGGTAAACAATTAATTGATGATGGTAGAATTGTTACTGCTCAAACCATCTCTGGTACTGGATCACTTAAACCTCTTATGGTGAAATTTTTGCTAAACATAGAGAACCAAATTTGGAAATTATTCGTGAGGTTGTTGATTCCAAACATATTGTTTTTGATGTGTTGGCACAATTCTTAATCAATCCAGACCCATGGGTTGCCATTGCTGCCGCTGAAGTTTATGTCAGACGTTCATACCGTGCTTATGATTTGGGTAAAATTGAATATCATGTTAATGACAGACTTCCTATTGTTGAATGGAAATTCAAGTTGGCTAATATGGGAGCYGCTGGTGTAAACGATGCTCAACAGGCTGCTGCTGCCGGTGGCGATGATTCGACATCTATGAAACATGCAGCTTCTGTGTCTGATTTGACCTTTGTTGTTGATTATTCATAAAAGYGAAATYCCAGGATACACTCTCCCCGATAATCCAAAGTTCACCCTTGGTAATTTGTTTGTAATAATTGGRGTCTTGTTGGTTTGTRTTTTAGCTGTTGTCTCTCTTTTGAGAAATATTAGTGAGTCRGCCTTGTTCAAGAAGAATGGGTATGAACCGTTGGATTCRGATCCTAGTGTCATGAACCWAAACTTYGAGCCTACAACATTGTCCTTTGAAGATATTAAATATGAGGTTACTGGTGGTCGACAAATTTTAAATGGAGTCTTTGGGTTTGTAAAACCAAGAGAATGTTTGGCTATAATGGGAGGTTCAGGTGCTGGTAAAACTACATTGTTGGATTTTAAACCTTTGGACCAATTRGCTAAGACTTTGACCACTGTTCCTGAATTGAATGAAATTATTGGGCAAGATTTAGTTGACGAATTTGTCAGTGGTATTAAACTACCAGCAGAAGTTGGAAGTCAAGATGATGTTAACAATAGAAAATTGTTGCAAAAAGTGTTTGGTAAATTAATGAACACTGATGATGACGTTATAAAACAACAAACAGCTAAATTACTTGAAAGAACAGACAGAGAACCTCAAGTGTTCAAGGATATTGATTCTAGATTACCGGAGTTAATACAAAGATTAAACAAACAATTTCCTAATGACATCGGATTATTTTGTGGATGTCTCTTATTGAACCACGTTGMTTGAAAAAATTATCAATTTCCGTGTTGGATAAACARAGATTGACTGAAAAATTCAATAAATTGGATAAATCCATYAAAGATAATTTGAAGGCTAAACAAAAAGAAGAAACCAAAAARACTTTAGATGTGGTTAAYAATTGGTTGAATGATAAAGAAAATGCTTCATCATTTTTGGTTGCTCACGTTCCAATTACTGCTAATGCCAAGGCAATCACTGAAGCCATTAATTTGATTAAAAAGCAAGATAAAACCAAATCAATTTATTTATTGACTGGTGAAACCGATAAAGTTGCTCATGGATGTTATGTTAGTGATGAAGCCATTGYCAAGGGTATTAATGCGAAAATCAAACCTTTTGAAGCACGAGCAATTAACTGGTCCACGGATCTTAATGCTGAGGTACATATTGAGCATTATATAAATATATTCAATTATGCACGATCATCTTGGGAGCCATTGGTTGAAAGTTGGCCAATAGCAGTTTACATGTCAAAATCCCGACACCCAAAGCCTCAATTATTAGTAGAGGTGATTTCTAGACAGRTAGCTCAAGTGACGCTTACATCCAAAGCGGTAGCATTGCTATCTCAAGTATCCGATTTGATTACTTCCGRAGAAAAATTAAAACCAAGAGGTGAAGATTACCCATACGTTATAGTGAATGARACTGGTTTAGATTTGGAAGTTTGGAATGATGCAAAWGAATCCGAAACCAATGGAGTTCCAATTGTTTTACGTGCTGGTAAAGCTTTAGATGAAAGTAAAGTTGAAATTAGAATTCAATTTAAACCAGTCGCCAAGGGGATGTTTAAAGAAATTCAAAGAAATGAATTAGTTATTAGAGTACAACCAAATGAAGCCATTTATTTAAAAATTAATTCCAAAATCCCTGGAATTTCTACTGAAACTTCATTAACTGATTTAGATTTAACTTATGCTACTCGTTATTCTAAAGAYTTTTGGATTCCTGAAGCTTATGAAGCATTAATTAGAGATTGTTATTTAGGTAATCATTCTAATTTTGTTAGAGATGATGAATTGGATGTTTCTTGGAAATTATTTACTCCATTATTGAAT

>Mi14_oral_4/02/2013

AATTATTGAAATGGAATGATATTCCATTGGCYCCACCAGACAAGATTTTGGGTATTTCTGAAGCTTATAACAATGATTCTAACCCTCAAAAARTCAATTTGGGGGTTGGTGCTTATAGAGATAATTCTGGTAAACCAATTATTTTCCCATCAGTTAAAAAAGCTGAAGAAATTTTATTGGGTAAAGAAACTGAAAAGGAATATACTGCCATTGTTGGTTCCAAAAATTTCCAATCAATTGTGAAAAATTTCATTTTCAACAATTCTAATAAAGATGCCAATGGTAAACAATTAATTGATGATGGTAGAATTGTTACTGCTCAAACCATYTCTGGTACTGGATCACTTAAACCTCTTATGGTGAAATTTTTGCTAAACATAGAGAACCAAATTTGGAAATTATTCGTGAGGTTGTTGATTCCAAACATATTGTTTTTGATGTGTTGGCACAATTCTTAATCAATCCAGACCCATGGGTTGCCATTGCTGCCGCTGAAGTTTATGTCAGACGTTCATACCGTGCTTATGATTTGGGTAAAATTGAATATCATGTTAATGACAGACTTCCTATTGTTGAATGGAAATTCAAGTTGGCTAATATGGGAGCYGCTGGTGTAAACGATGCTCAACAGGCTGCTGCTGCCGGTGGCGATGATTCGACATCTATGAAACATGCAGCTTCTGTGTCTGATTTGACCTTTGTTGTTGATTATTCATAAAAGCGAAATCCCAGGATACACTCTCCCCGATAATCCAAAGTTCACCCTTGGTAATTTGTTTGTAATAATTGGAGTCTTGTTGGTTTGTATTTTAGCTGTTGTCTCTCTTTTGAGAAATATTAGTGAGTCGGCCTTGTTCAAGAAGAATGGGTATGAACCGTTGGATTCGGATCCTAGTRTCATGAACCTAAACTTCGAGCCTACAACATTGTCCTTTGAAGATATTAAATATGAGGTTACTGGTGGTCGACAAATTTTAAATGGAGTCTTTGGGTTTGTAAAACCAAGAGAATGTTTGGCTATAATGGGAGGTTCAGGTGCTGGTAAAACTACATTGTTGGATTTTAAACCTTTGGACCAATTGGCTAAGACTTTGACCACTGTTCCTGAATTGAATGAAATTATTGGGCAAGATTTAGTTGACGAATTTGTCAGTGGTATTAAACTACCAGCAGAAGTTGGAAGTCAAGATGATGTTAACAATAGAAAATTGTTGCAAAAAGTGTTTGGTAAATTAATGAACACTGATGATGACGTTATAAAACAACAAACAGCTAAATTACTTGAAAGAACAGACAGAGAACCTCAAGTGTTCAAGGATATTGATTCTAGATTACCGGAGTTAATACAAAGATTAAACAAACAATTTCCTAATGACATCGGATTATTTTGTGGATGTCTCTTATTGAACCACGTTGMTTGAAAAAATTATCAATTTCCGTGTTGGATAAACARAGATTGACTGAAAAATTCAATAAATTGGATAAATCCATYAAAGATAATTTGAAGGCTAAACAAAAAGAAGAAACCAAAAARACTTTAGATGTGGTTAAYAATTGGTTGAATGATAAAGAAAATGCTTCATCATTTTTGGTTGCTCACGTTCCAATTACTGCTAATGCCAAGGCAATCACTGAAGCCATTAATTTGATTAAAAAGCAAGATAAAACCAAATCAATTTATTTATTGACTGGTGAAACCGATAAAGTTGCTCATGGATGTTATGTTAGTGATGAAGCCATTGYCAAGGGTATTAATGCGAAAATCAAACCTTTTGAAGCACGAGCAATTAACTGGTCCACGGATCTTAATGCTGAGGTACATATTGAGCATTATATAAATATATTCAATTATGCACGATCATCTTGGGAGCCATTGGTTGAAAGTTGGCCAATAGCAGTTTACATGTCAAAATCCCGACACCCAAAGCCTCAATTATTAGTAGAGGTGATTTCTAGACAGGTAGCTCAAGTGACGCTTACATCCAAAGCAGTAGCATTGCTATCTCAAGTATCCGATTTGATTACTTCCAGAGAAAAATTAAAACCAAGAGGTGAAGATTACCCATACGTTATAGTGAATGAGACTGGTTTAGATTTGGAAGTTTGGAATGATGCAAATGAATCCGAAACCAATGGAGTTCCAATTGTTTTACGTGCTGGTAAAGCTTTAGATGAAAGTAAAGTTGAAATTAGAATTCAATTTAAACCAGTCGCCAAGGGGATGTTTAAAGAAATTCAAAGAAATGAATTAGTTATTAGAGTACAACCAAATGAAGCCATTTATTTAAAAATTAATTCCAAAATCCCTGGAATTTCTACTGAAACTTCATTAACTGATTTAGATTTAACTTATGCTACTCGTTATTCTAAAGACTTTTGGATTCCTGAAGCTTATGAAGCATTAATTAGAGATTGTTATTTAGGTAATCATTCTAATTTTGTTAGAGATGATGAATTGGATGTTTCTTGGAAATTATTTACTCCATTATTGAAT

>Mi14_urine_18/03/2013

AATTATTGAAATGGAATGATATTCCATTGGCTCCACCAGACAAAATTTTGGGTATTTCTGAAGCTTATAACAATGATTCTAACCCTCAAAAAATCAATTTGGGGGTTGGTGCTTATAGAGATAATTCCGGTAAACCAATTATTTTCCCATCAGTTAAAAAAGCTGAAGAAATTTTATTGGGTAAAGAAACTGAAAAGGAATATACTGCCATTGTTGGTTCCAAAAATTTCCAATCAATTGTGAAAAATTTCATTTTCAACAATTCTAATAAAGATGCCAATGGTAAACAATTAATTGATGATGGTAGAATTGTTACTGCTCAAACCATCTCTGGTACTGGATCACTTAAACCTCTTATGGTGAAATTTTTGCTAAACATAGAGAACCAAATTTGGAAATTATTCGTGAGGTTGTTGATTCCAAACATATTGTTTTTGATGTGTTGGCACAATTCTTAATCAATCCAGACCCATGGGTTGCCATTGCTGCCGCTGAAGTTTATGTCAGACGTTCATACCGTGCTTATGATTTGGGTAAAATTGAATATCATGTTAATGACAGACTTCCTATTGTTGAATGGAAATTCAAGTTGGCTAATATGGGAGCYGCTGGTGTAAACGATGCTCAACAGGCTGCTGCTGCCGGTGGCGATGATTCGACATCTATGAAACATGCAGCTTCTGTGTCTGATTTGACCTTTGTTGTTGATTATTCATAAAAGYGAAATYCCAGGATACACTCTCCCCGATAATCCAAAGTTCACCCTTGGTAATTTGTTTGTAATAATTGGRGTCTTGTTGGTTTGTRTTTTAGCTGTTGTCTCTCTTTTGAGAAATATTAGTGAGTCRGCCTTGTTCAAGAAGAATGGGTATGAACCGTTGGATTCRGATCCTAGTGTCATGAACCWAAACTTYGAGCCTACAACATTGTCCTTTGAAGATATTAAATATGAGGTTACTGGTGGTCGACAAATTTTAAATGGAGTCTTTGGGTTTGTAAAACCAAGAGAATGTTTGGCTATAATGGGAGGTTCAGGTGCTGGTAAAACTACATTGTTGGATTTTAAACCTTTGGACCAATTRGCTAAGACTTTGACCACTGTTCCTGAATTGAATGAAATTATTGGGCAAGATTTAGTTGACGAATTTGTCAGTGGTATTAAACTACCAGCAGAAGTTGGAAGTCAAGATGATGTTAACAATAGAAAATTGTTGCAAAAAGTGTTTGGTAAATTAATGAACACTGATGATGACGTTATAAAACAACAAACAGCTAAATTACTTGAAAGAACAGACAGAGAACCTCAAGTGTTCAAGGATATTGATTCTAGATTACCGGAGTTAATACAAAGATTAAACAAACAATTTCCTAATGACATCGGATTATTTTGTGGATGTCTCTTATTGAACCACGTTGMTTGAAAAAATTATCAATTTCCGTGTTGGATAAACAGAGATTGACTGAAAAATTCAATAAATTGGATAAATCCATCAAAGATAATTTGAAGGCTAAACAAAAAGAAGAAACCAAAAAAACTTTAGATGTGGTTAATAATTGGTTGAATGATAAAGAAAATGCTTCATCATTTTTGGTTGCTCACGTTCCAATTACTGCTAATGCCAAGGCAATCACTGAAGCCATTAATTTGATTAAAAAGCAAGATAAAACCAAATCAATTTATTTATTGACTGGTGAAACCGATAAAGTTGCTCATGGATGTTATGTTAGTGATGAAGCCATTGCCAAGGGTATTAATGCGAAAATCAAACCTTTTGAAGCACGAGCAATTAACTGGTCCACGGATCTTAATGCTGAGGTACATATTGAGCATTATATAAATATATTCAATTATGCACGATCATCTTGGGAGCCATTGGTTGAAAGTTGGCCAATAGCAGTTTACATGTCAAAATCCCGACACCCAAAGCCTCAATTATTAGTAGAGGTGATTTCTAGACAGRTAGCTCAAGTGACGCTTACATCCAAAGCGGTAGCATTGCTATCTCAAGTATCCGATTTGATTACTTCCGRAGAAAAATTAAAACCAAGAGGTGAAGATTACCCATACGTTATAGTGAATGARACTGGTTTAGATTTGGAAGTTTGGAATGATGCAAAWGAATCCGAAACCAATGGAGTTCCAATTGTTTTACGTGCTGGTAAAGCTTTAGATGAAAGTAAAGTTGAAATTAGAATTCAATTTAAACCAGTCGCCAAGGGGATGTTTAAAGAAATTCAAAGAAATGAATTAGTTATTAGAGTACAACCAAATGAAGCCATTTATTTAAAAATTAATTCCAAAATCCCTGGAATTTCTACTGAAACTTCATTAACTGATTTAGATTTAACTTATGCTACTCGTTATTCTAAAGAYTTTTGGATTCCTGAAGCTTATGAAGCATTAATTAGAGATTGTTATTTAGGTAATCATTCTAATTTTGTTAGAGATGATGAATTGGATGTTTCTTGGAAATTATTTACTCCATTATTGAAT

>Mi15_rectal_8/03/2013

AATTATTGAAATGGAATGATATTCCATTGGCYCCACCAGACAAGATTTTGGGTATTTCTGAAGCTTATAACAATGATTCTAACCCTCAAAAARTCAATTTGGGGGTTGGTGCTTATAGAGATAATTCTGGTAAACCAATTATTTTCCCATCAGTTAAAAAAGCTGAAGAAATTTTATTGGGTAAAGAAACTGAAAAGGAATATACTGCCATTGTTGGTTCCAAAAATTTCCAATCAATTGTGAAAAATTTCATTTTCAACAATTCTAATAAAGATGCCAATGGTAAACAATTAATTGATGATGGTAGAATTGTTACTGCTCAAACCATYTCTGGTACTGGATCACTTAAACCTCTTATGGTGAAATTTTTGCTAAACATAGAGAACCAAATTTGGAAATTATTCGTGAGGTTGTTGATTCCAAACATATTGTTTTTGATGTGTTGGCACAATTCTTAATCAATCCAGACCCATGGGTTGCCATTGCTGCCGCTGAAGTTTATGTCAGACGTTCATACCGTGCTTATGATTTGGGTAAAATTGAATATCATGTTAATGACAGACTTCCTATTGTTGAATGGAAATTCAAGTTGGCTAATATGGGAGCYGCTGGTGTAAACGATGCTCAACAGGCTGCTGCTGCCGGTGGCGATGATTCGACATCTATGAAACATGCAGCTTCTGTGTCTGATTTGACCTTTGTTGTTGATTATTCATAAAAGCGAAATCCCAGGATACACTCTCCCCGATAATCCAAAGTTCACCCTTGGTAATTTGTTTGTAATAATTGGAGTCTTGTTGGTTTGTATTTTAGCTGTTGTCTCTCTTTTGAGAAATATTAGTGAGTCGGCCTTGTTCAAGAAGAATGGGTATGAACCGTTGGATTCGGATCCTAGTRTCATGAACCTAAACTTCGAGCCTACAACATTGTCCTTTGAAGATATTAAATATGAGGTTACTGGTGGTCGACAAATTTTAAATGGAGTCTTTGGGTTTGTAAAACCAAGAGAATGTTTGGCTATAATGGGAGGTTCAGGTGCTGGTAAAACTACATTGTTGGATTTTAAACCTTTGGACCAATTGGCTAAGACTTTGACCACTGTTCCTGAATTGAATGAAATTATTGGGCAAGATTTAGTTGACGAATTTGTCAGTGGTATTAAACTACCAGCAGAAGTTGGAAGTCAAGATGATGTTAACAATAGAAAATTGTTGCAAAAAGTGTTTGGTAAATTAATGAACACTGATGATGACGTTATAAAACAACAAACAGCTAAATTACTTGAAAGAACAGACAGAGAACCTCAAGTGTTCAAGGATATTGATTCTAGATTACCGGAGTTAATACAAAGATTAAACAAACAATTTCCTAATGACATCGGATTATTTTGTGGATGTCTCTTATTGAACCACGTTGMTTGAAAAAATTATCAATTTCCGTGTTGGATAAACARAGATTGACTGAAAAATTCAATAAATTGGATAAATCCATYAAAGATAATTTGAAGGCTAAACAAAAAGAAGAAACCAAAAARACTTTAGATGTGGTTAAYAATTGGTTGAATGATAAAGAAAATGCTTCATCATTTTTGGTTGCTCACGTTCCAATTACTGCTAATGCCAAGGCAATCACTGAAGCCATTAATTTGATTAAAAAGCAAGATAAAACCAAATCAATTTATTTATTGACTGGTGAAACCGATAAAGTTGCTCATGGATGTTATGTTAGTGATGAAGCCATTGYCAAGGGTATTAATGCGAAAATCAAACCTTTTGAAGCACGAGCAATTAACTGGTCCACGGATCTTAATGCTGAGGTACATATTGAGCATTATATAAATATATTCAATTATGCACGATCATCTTGGGAGCCATTGGTTGAAAGTTGGCCAATAGCAGTTTACATGTCAAAATCCCGACACCCAAAGCCTCAATTATTAGTAGAGGTGATTTCTAGACAGGTAGCTCAAGTGACGCTTACATCCAAAGCAGTAGCATTGCTATCTCAAGTATCCGATTTGATTACTTCCAGAGAAAAATTAAAACCAAGAGGTGAAGATTACCCATACGTTATAGTGAATGAGACTGGTTTAGATTTGGAAGTTTGGAATGATGCAAATGAATCCGAAACCAATGGAGTTCCAATTGTTTTACGTGCTGGTAAAGCTTTAGATGAAAGTAAAGTTGAAATTAGAATTCAATTTAAACCAGTCGCCAAGGGGATGTTTAAAGAAATTCAAAGAAATGAATTAGTTATTAGAGTACAACCAAATGAAGCCATTTATTTAAAAATTAATTCCAAAATCCCTGGAATTTCTACTGAAACTTCATTAACTGATTTAGATTTAACTTATGCTACTCGTTATTCTAAAGACTTTTGGATTCCTGAAGCTTATGAAGCATTAATTAGAGATTGTTATTTAGGTAATCATTCTAATTTTGTTAGAGATGATGAATTGGATGTTTCTTGGAAATTATTTACTCCATTATTGAAT

>Mi15_rectal_29/05/2013

AATTATTGAAGTGGAATGATATTCCATTGGCTCCACCAGACAAAATTTTGGGTATTTCTGAAGCTTATAACAATGATTCTAACCCTCAAAAAATCAATTTGGGGGTTGGTGCTTATAGAGATAATTCCGGTAAACCAATTATTTTCCCATCAGTTAAAAAAGCTGAAGAAATTTTATTGGGTAAAGAAACTGAAAAGGAATATACTGCCATTGTTGGTTCCAAAAATTTCCAATCAATTGTGAAAAATTTCATTTTCAACAATTCTAATAAAGATGCCAATGGTAAACAATTAATTGATGATGGTAGAATTGTTACTGCTCAAACCATCTCTGGTACTGGATCACTTAAACCTCWTATGGTGAAATTTTTGCTAAACATAGAGAACCAAATTTGGAAATTATTCGTGAGGTTGTTGATTCCAAACATATTGTTTTTGATGTGTTGGCACAATTCTTAATCAATCCAGACCCATGGGTTGCCATTGCTGCCGCTGAAGTTTATGTCAGACGTTCATACCGTGCTTATGATTTGGGTAAAATTGAATATCATGTTAATGACAGACTTCCTATTGTTGAATGGAAATTCAAGTTGGCTAATATGGGAGCCGCTGGTGTAAACGATGCTCAACAGGCTGCTGCTGCCGGTGGCGATGATTCGACATCTATGAAACATGCAGCTTCTGTGTCTGATTTGACCTTTGTTGTTGATTATTCATAAAAGYGAAATYCCAGGATACACTCTCCCCGATAATCCAAAGTTCACCCTTGGTAATTTGTTTGTAATAATTGGRGTCTTGTTGGTTTGTRTTTTAGCTGTTGTCTCTCTTTTGAGAAATATTAGTGAGTCRGCCTTGTTCAAGAAGAATGGGTATGAACCGTTGGATTCRGATCCTAGTGTCATGAACCWAAACTTYGAGCCTACAACATTGTCCTTTGAAGATATTAAATATGAGGTTACTGGTGGTCGACAAATTTTAAATGGAGTCTTTGGGTTTGTAAAACCAAGAGAATGTTTGGCTATAATGGGAGGTTCAGGTGCTGGTAAAACTACATTGTTGGATTTTAAACCTTTGGACCAATTRGCTAAGACTTTGACCACTGTTCCTGAATTGAATGAAATTATTGGGCAAGATTTAGTTGACGAATTTGTCAGTGGTATTAAACTACCAGCAGAAGTTGGAAGTCAAGATGATGTTAACAATAGAAAATTGTTGCAAAAAGTGTTTGGTAAATTAATGAACACTGATGATGACGTTATAAAACAACAAACAGCTAAATTACTTGAAAGAACAGACAGAGAACCTCAAGTGTTCAAGGATATTGATTCTAGATTACCGGAGTTAATACAAAGATTAAACAAACAATTTCCTAATGACATCGGATTATTTTGTGGATGTCTCTTATTGAACCACGTTGCTTGAAAAAATTATCAATTTCCGTGTTGGATAAACARAGATTGACTGAAAAATTCAATAAATTGGATAAATCCATTAAAGATAATTTGAAGGCTAAACAAAAAGAAGAAACCAAAAAGACTTTAGATGTGGTTAACAATTGGTTGAATGATAAAGAAAATGCTTCATCATTTTTGGTTGCTCACGTTCCAATTACTGCTAATGCCAAGGCAATCACTGAAGCCATTAATTTGATTAAAAAGCAAGATAAAACCAAATCAATTTATTTATTGACTGGTGAAACCGATAAAGTTGCTCATGGATGTTATGTTAGTGATGAAGCCATTGTCAAGGGTATTAATGCGAAAATCAAACCTTTTGAAGCACGAGCAATTAACTGGTCCACGGATCTTAATGCTGAGGTACATATTGAGCATTATATAAATATATTCAATTATGCACGATCATCTTGGGAGCCATTGGTTGAAAGTTGGCCAATAGCAGTTTACATGTCAAAATCCCGACACCCAAAGCCTCAATTATTAGTAGAGGTGATTTCTAGACAGGTAGCYCAAGTGACGCTTACATCCAAAGCRGTAGCATTGCTATCTCAAGTATCCGATTTGATTACTTCCGGAGAAAAATTAAAACCAAGAGGTGAAGATTACCCATACGTTATAGTGAATGAGACTGGTTTAGATTTGGAAGTTTGGAATGATGCAAAWGAATCCGAAACCAATGGAGTTCCAATTGTTTTACGTGCTGGTAAAGCTTTAGATGAAAGTAAAGTTGAAATTAGAATTCAATTTAAACCAGTCGCCAAGGGGATGTTTAAAGAAATTCAAAGAAATGAATTAGTTATTAGAGTACAACCAAATGAAGCCATTTATTTAAAAATTAATTCCAAAATCCCTGGAATTTCTACTGAAACTTCATTAACTGATTTAGATTTAACTTATGCTACTCGTTATTCTAAAGACTTTTGGATTCCTGAAGCTTATGAAGCATTAATTAGAGATTGYTATTTAGGTAATCATTCTAATTTTGTTAGAGATGATGAATTGGATGTTTCTTGGAAATTATTTACTCCATTATTGAAT

>Mi15_vaginal_29/05/2013

AATTATTGAAATGGAATGATATTCCATTGGCYCCACCAGACAAGATTTTGGGTATTTCTGAAGCTTATAACAATGATTCTAACCCTCAAAAARTCAATTTGGGGGTTGGTGCTTATAGAGATAATTCTGGTAAACCAATTATTTTCCCATCAGTTAAAAAAGCTGAAGAAATTTTATTGGGTAAAGAAACTGAAAAGGAATATACTGCCATTGTTGGTTCCAAAAATTTCCAATCAATTGTGAAAAATTTCATTTTCAACAATTCTAATAAAGATGCCAATGGTAAACAATTAATTGATGATGGTAGAATTGTTACTGCTCAAACCATYTCTGGTACTGGATCACTTAAACCTCTTATGGTGAAATTTTTGCTAAACATAGAGAACCAAATTTGGAAATTATTCGTGAGGTTGTTGATTCCAAACATATTGTTTTTGATGTGTTGGCACAATTCTTAATCAATCCAGACCCATGGGTTGCCATTGCTGCCGCTGAAGTTTATGTCAGACGTTCATACCGTGCTTATGATTTGGGTAAAATTGAATATCATGTTAATGACAGACTTCCTATTGTTGAATGGAAATTCAAGTTGGCTAATATGGGAGCYGCTGGTGTAAACGATGCTCAACAGGCTGCTGCTGCCGGTGGCGATGATTCGACATCTATGAAACATGCAGCTTCTGTGTCTGATTTGACCTTTGTTGTTGATTATTCATAAAAGCGAAATCCCAGGATACACTCTCCCCGATAATCCAAAGTTCACCCTTGGTAATTTGTTTGTAATAATTGGAGTCTTGTTGGTTTGTATTTTAGCTGTTGTCTCTCTTTTGAGAAATATTAGTGAGTCGGCCTTGTTCAAGAAGAATGGGTATGAACCGTTGGATTCGGATCCTAGTRTCATGAACCTAAACTTCGAGCCTACAACATTGTCCTTTGAAGATATTAAATATGAGGTTACTGGTGGTCGACAAATTTTAAATGGAGTCTTTGGGTTTGTAAAACCAAGAGAATGTTTGGCTATAATGGGAGGTTCAGGTGCTGGTAAAACTACATTGTTGGATTTTAAACCTTTGGACCAATTGGCTAAGACTTTGACCACTGTTCCTGAATTGAATGAAATTATTGGGCAAGATTTAGTTGACGAATTTGTCAGTGGTATTAAACTACCAGCAGAAGTTGGAAGTCAAGATGATGTTAACAATAGAAAATTGTTGCAAAAAGTGTTTGGTAAATTAATGAACACTGATGATGACGTTATAAAACAACAAACAGCTAAATTACTTGAAAGAACAGACAGAGAACCTCAAGTGTTCAAGGATATTGATTCTAGATTACCGGAGTTAATACAAAGATTAAACAAACAATTTCCTAATGACATCGGATTATTTTGTGGATGTCTCTTATTGAACCACGTTGMTTGAAAAAATTATCAATTTCCGTGTTGGATAAACARAGATTGACTGAAAAATTCAATAAATTGGATAAATCCATYAAAGATAATTTGAAGGCTAAACAAAAAGAAGAAACCAAAAARACTTTAGATGTGGTTAAYAATTGGTTGAATGATAAAGAAAATGCTTCATCATTTTTGGTTGCTCACGTTCCAATTACTGCTAATGCCAAGGCAATCACTGAAGCCATTAATTTGATTAAAAAGCAAGATAAAACCAAATCAATTTATTTATTGACTGGTGAAACCGATAAAGTTGCTCATGGATGTTATGTTAGTGATGAAGCCATTGYCAAGGGTATTAATGCGAAAATCAAACCTTTTGAAGCACGAGCAATTAACTGGTCCACGGATCTTAATGCTGAGGTACATATTGAGCATTATATAAATATATTCAATTATGCACGATCATCTTGGGAGCCATTGGTTGAAAGTTGGCCAATAGCAGTTTACATGTCAAAATCCCGACACCCAAAGCCTCAATTATTAGTAGAGGTGATTTCTAGACAGGTAGCTCAAGTGACGCTTACATCCAAAGCAGTAGCATTGCTATCTCAAGTATCCGATTTGATTACTTCCAGAGAAAAATTAAAACCAAGAGGTGAAGATTACCCATACGTTATAGTGAATGAGACTGGTTTAGATTTGGAAGTTTGGAATGATGCAAATGAATCCGAAACCAATGGAGTTCCAATTGTTTTACGTGCTGGTAAAGCTTTAGATGAAAGTAAAGTTGAAATTAGAATTCAATTTAAACCAGTCGCCAAGGGGATGTTTAAAGAAATTCAAAGAAATGAATTAGTTATTAGAGTACAACCAAATGAAGCCATTTATTTAAAAATTAATTCCAAAATCCCTGGAATTTCTACTGAAACTTCATTAACTGATTTAGATTTAACTTATGCTACTCGTTATTCTAAAGACTTTTGGATTCCTGAAGCTTATGAAGCATTAATTAGAGATTGTTATTTAGGTAATCATTCTAATTTTGTTAGAGATGATGAATTGGATGTTTCTTGGAAATTATTTACTCCATTATTGAAT

>Mi16_oral_18/03/2013

AATTATTGAAATGGAATGATATTCCATTGGCCCCACCAGACAAGATTTTGGGTATTTCTGAAGCTTATAACAATGATTCTAACCCTCAAAAAATCAATTTGGGGGTTGGTGCTTATAGAGATAATTCTGGTAAACCAATTATTTTCCCATCAGTTAAAAAAGCTGAAGAAATTTTATTGGGTAAAGAAACTGAAAAGGAATATACTGCCATTGTTGGTTCCAAAAATTTCCAATCAATTGTGAAAAATTTCATTTTCAACAATTCTAATAAAGATGCCAATGGTAAACAATTAATTGATGATGGTAGAATTGTTACTGCTCAAACCATCTCTGGTACTGGATCACTTAAACCTCTTATGGTGAAATTTTTGCTAAACATAGAGAACCAAATTTGGAAATTATTCGTGARGTTGTTGATTCCAAACATATTGTTTTTGATGTGTTGGCACAATTCTTAATCAATCCAGACCCATGGGTTGCCATTGCTGCCGCTGAAGTTTATGTCAGACGTTCATACCGTGCTTATGATTTGGGTAAAATTGAATATCATGTTAATGACAGACTTCCTATTGTTGAATGGAAATTCAAGTTGGCTAATATGGGRGCCGCTGGTGTAAACGATGCTCAACAGGCTGCTGCTGCCGGTGGCGATGATTCGACATCTATGAAACATGCAGCTTCTGTGTCTGATTTGACCTTTGTTGTTGATTATTCATAAAAGCGAAATCCCAGGATACACTCTCCCCGATAATCCAAAGTTCACCCTTGGTAATTTGTTTGTAATAATTGGAGTCTTGTTGGTTTGTATTTTAGCTGTTGTCTCTCTTTTGAGAAATATTAGTGAGTCGGCCTTGTTCAAGAAGAATGGGTATGAACCGTTGGATTCGGATCCTAGTGTCATGAACCTAAACTTCGAGCCTACAACATTGTCCTTTGAAGATATTAAATATGAGGTTACTGGTGGTCGACAAATTTTAAATGGAGTCTTTGGGTTTGTAAAACCAAGAGAATGTTTGGCTATAATGGGAGGTTCAGGTGCTGGTAAAACTACATTGTTGGATTTTAAACCTTTGGACCAATTGGCTAARACTTTGRCYACTGTTCCTGAATTGAATGAAATTATTGGKCAAGAWTTAGTTGACGAATTTRTCAGTGGTATTAAACTACCAGCAGAAGTTGGAAGTCAAGATGATGTTAACAATAGAAAATTGTTGCAAAAAGTGTTTGGTAAATTAATGAACACTGATGATGACGTTATAAAACAACAAACAGCTAAATTACTTGAAAGAACAGAMAGRGAACCTCAAGTGTTCAAGGATATTGATTCTAGATTACCRGAGTTAATACAARGATTAAACAAACAATTTCCTAATGACATCGGATTATTTTGTGGATGTCTCTTATTGAACCACGTTGCTTGAAAAAATTATCAATTTCCGTGTTGGATAAACAGAGATTGACTGAAAAATTCAATAAATTGGATAAATCCATTAAAGATAATTTGAAGGCTAAACAAAAAGAAGAAACCAAAAAGACTTTAGATGTGGTTAACAATTGGTTGAATGATAAAGAAAATGCTTCATCATTTTTGGTTGCTCACGTTCCAATTACTGCTAATGCCAAGGCAATCACTGAAGCCATTAATTTGATTAAAAAGCAAGATAAAACCAAATCAATTTATTTATTGACTGGTGAAACCGATAAAGTTGCTCATGGATGTTATGTTAGTGATGAAGCCATTGTCAAGGGTATTAATGCGAAAATCAAACCTTTTGAAGCACGAGCAATTAACTGGTCCACGGATCTTAATGCTGAGGTACATATTGAGCATTATATAAATATATTCAATTATGCACGATCATCTTGGGAGCCATTGGTTGAAAGTTGGCCAATAGCAGTTTACATGTCAAAATCCCGACACCCAAAGCCTCAATTATTAGTAGAGGTGATTTCTAGACAGGTAGCCCAAGTGACGCTTACATCCAAAGCAGTAGCATTGCTATCTCAAGTATCCGATTTGATTACTTCCGGAGAAAAATTAAAACCAAGAGGTGAAGATTACCCATACGTTATAGTGAATGAGACTGGTTTAGATTTGGAAGTTTGGAATGATGCAAAAGAATCCGAAACCAATGGAGTTCCAATTGTTTTACGTGCTGGTAAAGCTTTAGATGAAAGTAAAGTTGAAATTAGAATTCAATTTAAACCAGTCGCCAAGGGGATGTTTAAAGAAATTCAAAGAAATGAATTAGTTATTAGAGTACAACCAAATGAAGCCATTTATTTAAAAATTAATTCCAAAATCCCTGGAATTTCTACTGAAACTTCATTAACTGATTTAGATTTAACTTATGCTACTCGTTATTCTAAAGAYTTTTGGATTCCTGAAGCTTATGAAGCATTAATTAGAGATTGTTATTTAGGTAATCATTCTAATTTTGTTAGAGATGATGAATTGGATGTTTCTTGGAAATTATTTACTCCATTATTGAAT

>Mi16_oral_18/03/2013:IND:15:110

AATTATTGAAATGGAATGATATTCCATTGGCTCCACCAGACAAAATTTTGGGTATTTCTGAAGCTTATAACAATGATTCTAACCCTCAAAAAATCAATTTGGGGGTTGGTGCTTATAGAGATAATTCCGGTAAACCAATTATTTTCCCATCAGTTAAAAAAGCTGAAGAAATTTTATTGGGTAAAGAAACTGAAAAGGAATATACTGCCATTGTTGGTTCCAAAAATTTCCAATCAATTGTGAAAAATTTCATTTTCAACAATTCTAATAAAGATGCCAATGGTAAACAATTAATTGATGATGGTAGAATTGTTACTGCTCAAACCATCTCTGGTACTGGATCACTTAAACCTCTTATGGTGAAATTTTTGCTAAACATAGAGAACCAAATTTGGAAATTATTCGTGARGTTGTTGATTCCAAACATATTGTTTTTGATGTGTTGGCACAATTCTTAATCAATCCAGACCCATGGGTTGCCATTGCTGCCGCTGAAGTTTATGTCAGACGTTCATACCGTGCTTATGATTTGGGTAAAATTGAATATCATGTTAATGACAGACTTCCTATTGTTGAATGGAAATTCAAGTTGGCTAATATGGGAGCTGCTGGTGTAAACGATGCTCAACAGGCTGCTGCTGCCGGTGGCGATGATTCGACATCTATGAAACATGCAGCTTCTGTGTCTGATTTGACCTTTGTTGTTGATTATTCATAAAAGYGAAATYCCAGGATACACTCTCCCCGATAATCCAAAGTTCACCCTTGGTAATTTGTTTGTAATAATTGGRGTCTTGTTGGTTTGTRTTTTAGCTGTTGTCTCTCTTTTGAGAAATATTAGTGAGTCRGCCTTGTTCAAGAAGAATGGGTATGAACCGTTGGATTCRGATCCTAGTGTCATGAACCWAAACTTYGAGCCTACAACATTGTCCTTTGAAGATATTAAATATGAGGTTACTGGTGGTCGACAAATTTTAAATGGAGTCTTTGGGTTTGTAAAACCAAGAGAATGTTTGGCTATAATGGGAGGTTCAGGTGCTGGTAAAACTACATTGTTGGATTTTAAACCTTTGGACCAATTGGCTAAGACTTTGACCACTGTTCCTGAATTGAATGAAATTATTGGGCAAGATTTAGTTGACGAATTTGTCAGTGGTATTAAACTACCAGCAGAAGTTGGAAGTCAAGATGATGTTAACAATAGAAAATTGTTGCAAAAAGTGTTTGGTAAATTAATGAACACTGATGATGACGTTATAAAACAACAAACAGCTAAATTACTTGAAAGAACAGACAGAGAACCTCAAGTGTTCAAGGATATTGATTCTAGATTACCGGAGTTAATACAAAGATTAAACAAACAATTTCCTAATGACATCGGATTATTTTGTGGATGTCTCTTATTGAACCACGTTGMTTGAAAAAATTATCAATTTCCGTGTTGGATAAACARAGATTGACTGAAAAATTCAATAAATTGGATAAATCCATYAAAGATAATTTGAAGGCTAAACAAAAAGAAGAAACCAAAAARACTTTAGATGTGGTTAAYAATTGGTTGAATGATAAAGAAAATGCTTCATCATTTTTGGTTGCTCACGTTCCAATTACTGCTAATGCCAAGGCAATCACTGAAGCCATTAATTTGATTAAAAAGCAAGATAAAACCAAATCAATTTATTTATTGACTGGTGAAACCGATAAAGTTGCTCATGGATGTTATGTTAGTGATGAAGCCATTGYCAAGGGTATTAATGCGAAAATCAAACCTTTTGAAGCACGAGCAATTAACTGGTCCACGGATCTTAATGCTGAGGTACATATTGAGCATTATATAAATATATTCAATTATGCACGATCATCTTGGGAGCCATTGGTTGAAAGTTGGCCAATAGCAGTTTACATGTCAAAATCCCGACACCCAAAGCCTCAATTATTAGTAGAGGTGATTTCTAGACAGGTAGCTCAAGTGACGCTTACATCCAAAGCGGTAGCATTGCTATCTCAAGTATCCGATTTGATTACTTCCGRAGAAAAATTAAAACCAAGAGGTGAAGATTACCCATACGTTATAGTGAATGAGACTGGTTTAGATTTGGAAGTTTGGAATGATGCAAAWGAATCCGAAACCAATGGAGTTCCAATTGTTTTACGTGCTGGTAAAGCTTTAGATGAAAGTAAAGTTGAAATTAGAATTCAATTTAAACCAGTCGCCAAGGGGATGTTTAAAGAAATTCAAAGAAATGAATTAGTTATTAGAGTACAACCAAATGAAGCCATTTATTTAAAAATTAATTCCAAAATCCCTGGAATTTCTACTGAAACTTCATTAACTGATTTAGATTTAACTTATGCTACTCGTTATTCTAAAGAYTTTTGGATTCCTGAAGCTTATGAAGCATTAATTAGAGATTGTTATTTAGGTAATCATTCTAATTTTGTTAGAGATGATGAATTGGATGTTTCTTGGAAATTATTTACTCCATTATTGAAT

>Mi16_oral_18/03/2013:IND:19:82.7

AATTATTGAAATGGAATGATATTCCATTGGCTCCACCAGACAAAATTTTGGGTATTTCTGAAGCTTATAACAATGATTCTAACCCTCAAAAAATCAATTTGGGGGTTGGTGCTTATAGAGATAATTCCGGTAAACCAATTATTTTCCCATCAGTTAAAAAAGCTGAAGAAATTTTATTGGGTAAAGAAACTGAAAAGGAATATACTGCCATTGTTGGTTCCAAAAATTTCCAATCAATTGTGAAAAATTTCATTTTCAACAATTCTAATAAAGATGCCAATGGTAAACAATTAATTGATGATGGTAGAATTGTTACTGCTCAAACCATCTCTGGTACTGGATCACTTAAACCTCTTATGGTGAAATTTTTGCTAAACATAGAGAACCAAATTTGGAAATTATTCGTGARGTTGTTGATTCCAAACATATTGTTTTTGATGTGTTGGCACAATTCTTAATCAATCCAGACCCATGGGTTGCCATTGCTGCCGCTGAAGTTTATGTCAGACGTTCATACCGTGCTTATGATTTGGGTAAAATTGAATATCATGTTAATGACAGACTTCCTATTGTTGAATGGAAATTCAAGTTGGCTAATATGGGAGCTGCTGGTGTAAACGATGCTCAACAGGCTGCTGCTGCCGGTGGCGATGATTCGACATCTATGAAACATGCAGCTTCTGTGTCTGATTTGACCTTTGTTGTTGATTATTCATAAAAGYGAAATYCCAGGATACACTCTCCCCGATAATCCAAAGTTCACCCTTGGTAATTTGTTTGTAATAATTGGRGTCTTGTTGGTTTGTRTTTTAGCTGTTGTCTCTCTTTTGAGAAATATTAGTGAGTCRGCCTTGTTCAAGAAGAATGGGTATGAACCGTTGGATTCRGATCCTAGTGTCATGAACCWAAACTTYGAGCCTACAACATTGTCCTTTGAAGATATTAAATATGAGGTTACTGGTGGTCGACAAATTTTAAATGGAGTCTTTGGGTTTGTAAAACCAAGAGAATGTTTGGCTATAATGGGAGGTTCAGGTGCTGGTAAAACTACATTGTTGGATTTTAAACCTTTGGACCAATTRGCTAAGACTTTGACCACTGTTCCTGAATTGAATGAAATTATTGGGCAAGATTTAGTTGACGAATTTGTCAGTGGTATTAAACTACCAGCAGAAGTTGGAAGTCAAGATGATGTTAACAATAGAAAATTGTTGCAAAAAGTGTTTGGTAAATTAATGAACACTGATGATGACGTTATAAAACAACAAACAGCTAAATTACTTGAAAGAACAGAMAGRGAACCTCAAGTGTTCAAGGATATTGATTCTAGATTACCRGAGTTAATACAARGATTAAACAAACAATTTCCTAATGACATCGGATTATTTTGTGGATGTCTCTTATTGAACCACGTTGMTTGAAAAAATTATCAATTTCCGTGTTGGATAAACARAGATTGACTGAAAAATTCAATAAATTGGATAAATCCATYAAAGATAATTTGAAGGCTAAACAAAAAGAAGAAACCAAAAARACTTTAGATGTGGTTAAYAATTGGTTGAATGATAAAGAAAATGCTTCATCATTTTTGGTTGCTCACGTTCCAATTACTGCTAATGCCAAGGCAATCACTGAAGCCATTAATTTGATTAAAAAGCAAGATAAAACCAAATCAATTTATTTATTGACTGGTGAAACCGATAAAGTTGCTCATGGATGTTATGTTAGTGATGAAGCCATTGYCAAGGGTATTAATGCGAAAATCAAACCTTTTGAAGCACGAGCAATTAACTGGTCCACGGATCTTAATGCTGAGGTACATATTGAGCATTATATAAATATATTCAATTATGCACGATCATCTTGGGAGCCATTGGTTGAAAGTTGGCCAATAGCAGTTTACATGTCAAAATCCCGACACCCAAAGCCTCAATTATTAGTAGAGGTGATTTCTAGACAGRTAGCTCAAGTGACGCTTACATCCAAAGCGGTAGCATTGCTATCTCAAGTATCCGATTTGATTACTTCCGRAGAAAAATTAAAACCAAGAGGTGAAGATTACCCATACGTTATAGTGAATGARACTGGTTTAGATTTGGAAGTTTGGAATGATGCAAAWGAATCCGAAACCAATGGAGTTCCAATTGTTTTACGTGCTGGTAAAGCTTTAGATGAAAGTAAAGTTGAAATTAGAATTCAATTTAAACCAGTCGCCAAGGGGATGTTTAAAGAAATTCAAAGAAATGAATTAGTTATTAGAGTACAACCAAATGAAGCCATTTATTTAAAAATTAATTCCAAAATCCCTGGAATTTCTACTGAAACTTCATTAACTGATTTAGATTTAACTTATGCTACTCGTTATTCTAAAGAYTTTTGGATTCCTGAAGCTTATGAAGCATTAATTAGAGATTGTTATTTAGGTAATCATTCTAATTTTGTTAGAGATGATGAATTGGATGTTTCTTGGAAATTATTTACTCCATTATTGAAT

>Mi16_rectal_25/03/2013

AATTATTGAAATGGAATGATATTCCATTGGCTCCACCAGACAAAATTTTGGGTATTTCTGAAGCTTATAACAAYGATTCTAACCCTCAAAAAATCAATTTGGGGGTTGGTGCTTATAGAGATAATTCCGGTAAACCAATTATTTTCCCATCAGTTAAAAAAGCTGAAGAAATTTTATTGGGTAAAGAAACTGAAAAGGAATATACTGCCATTGTTGGTTCCAAAAATTTCCAATCAATTGTGAAAAATTTCATTTTCAACAATTCTAATAAAGATGCCAATGGTAAACAATTAATTGATGATGGTAGAATTGTTACTGCTCAAACCATCTCTGGTACTGGATCACTTAAACCTCTTATGGTGAAATTTTTGCTAAACATAGAGAACCAAATTTGGAAATTATTCGTGAGGTTGTTGATTCCAAACATATTGTTTTTGATGTGTTGGCACAATTCTTAATCAATCCAGACCCATGGGTTGCCATTGCTGCCGCTGAAGTTTATGTCAGACGTTCATACCGTGCTTATGATTTGGGTAMAATTGAATATCATGTTAATGACAGACTTCCTATTGTTGAATGGAAATTCAAGTTGGCTAATATGGGAGCCGCTGGTGTAAACGATGCTCAACAGGCTGCTGCTGCTGGTGGCGATGATTCGACATCTATGAAACATGCAGCTTCTGTGTCTGATTTGACCTTTGTTGTTGATTATTCATAAAAGYGAAATYCCAGGATACACTCTCCCCGATAATCCAAAGTTCACCCTTGGTAATTTGTTTGTAATAATTGGRGTCTTGTTGGTTTGTRTTTTAGCTGTTGTCTCTCTTTTGAGAAATATTAGTGAGTCRGCCTTGTTCAAGAAGAATGGGTATGAACCGTTGGATTCRGATCCTAGTGTCATGAACCWAAACTTYGAGCCTACAACATTGTCCTTTGAAGATATTAAATATGAGGTTACTGGTGGTCGACAAATTTTAAATGGAGTCTTTGGGTTTGTAAAACCAAGAGAATGTTTGGCTATAATGGGAGGTTCAGGTGCTGGTAAAACTACATTGTTGGATTTTAAACCTTTGGACCAATTGGCTAAGACTTTGACCACTGTTCCTGAATTGAATGAAATTATTGGGCAAGATTTAGTTGACGAATTTGTCAGTGGTATTAAACTACCAGCAGAAGTTGGAAGTCAAGATGATGTTAACAATAGAAAATTGTTGCAAAAAGTGTTTGGTAAATTAATGAACACTGATGATGACGTTATAAAACAACAAACAGCTAAATTACTTGAAAGAACAGAAAGGGAACCTCAAGTGTTCAAGGATATTGATTCTAGATTACCAGAGTTAATACAAGGATTAAACAAACAATTTCCTAATGACATCGGATTATTTTGTGGATGTCTCTTATTGAACCACGTTGMTTGAAAAAATTATCAATTTCCGTGTTGGATAAACAAAGATTGACTGAAAAATTCAATAAATTGGATAAATCCATTAAAGATAATTTGAAGGCTAAACAAAAAGAAGAAACCAAAAAGACTTTAGATGTGGTTAACAATTGGTTGAATGATAAAGAAAATGCTTCATCATTTTTGGTTGCTCACGTTCCAATTACTGCTAATGCCAAGGCAATCACTGAAGCCATTAATTTGATTAAAAAGCAAGATAAAACCAAATCAATTTATTTATTGACTGGTGAAACAGATAAAGTTGCTCATGGATGTTATGTTAGTGATGAAGCCATTGCCAAGGGTATTAATGCGAAAATCAAACCTTTTGAAGCACGAGCAATTAACTGGTCAACGGATCTTAATGCTGAGGTACATATTGAGCATTATATAAATATATTCAATTATGCACGATCATCTTGGGAGCCATTGGTTGAAAGTTGGCCAATAGCAGTTTACATGTCAAAATCCCGACACCCAAAGCCTCAATTATTAGTAGAGGTGATTTCTAGACAGGTAGCTCAAGTGACGCTTACATCCAAAGCAGTAGCATTGCTATCTCAAGTATCCGATTTGATTACTTCCGGAGAAAAATTAAAACCAAGAGGTGAAGATTACCCATACATTATAGTGAATGAGACTGGTTTAGATTTGGAAGTTTGGAATGATGCAAAAGAATCCGAAACCAATGGAGTTCCAATTGTTTTACGTGCTGGTAAAGCTTTAGATGAAAGTAAAGTTGAAATTAGAATTCAATTTAAACCAGTCGCCAAGGGGATGTTTAAAGAAATTCAAAGAAATGAATTAGTTATTAGAGTACAACCAAATGAAGCCATTTATTTAAAAATTAATTCCAAAATCCCTGGAATTTCTACTGAAACTTCATTAACTGATTTAGATTTAACTTATGCTACTCGTTATTCTAAAGACTTTTGGATTCCTGAAGCTTATGAAGCATTAATTAGAGATTGTTATTTAGGTAATCATTCTAATTTTGTTAGAGATGATGAATTGGATGTTTCTTGGAAATTATTTACTCCATTATTGAAT

>Mi17_oral_6/07/2013

AATTATTGAAATGGAATGATATTCCATTGGCTCCACCAGACAAAATTTTGGGTATTTCTGAAGCTTATAACAATGATTCTAACCCTCAAAAAATCAATTTGGGGGTTGGTGCTTATAGAGATAATTCCGGTAAACCAATTATTTTCCCATCAGTTAAAAAAGCTGAAGAAATTTTATTGGGTAAAGAAACTGAAAAGGAATATACTGCCATTGTTGGTTCCAAAAATTTCCAATCAATTGTGAAAAATTTCATTTTCAACAATTCTAATAAAGATGCCAATGGTAAACAATTAATTGATGATGGTAGAATTGTTACTGCTCAAACCATCTCTGGTACTGGATCACTTAAACCTCTTATGGTGAAATTTTTGCTAAACATAGAGAACCAAATTTGGAAATTATTCGTGAGGTTGTTGATTCCAAACATATTGTTTTTGATGTGTTGGCACAATTCTTAATCAATCCAGACCCATGGGTTGCCATTGCTGCCGCTGAAGTTTATGTCAGACGTTCATACCGTGCTTATGATTTGGGTAAAATTGAATATCATGTTAATGACAGACTTCCTATTGTTGAATGGAAATTCAAGTTGGCTAATATGGGAGCYGCTGGTGTAAACGATGCTCAACAGGCTGCTGCTGCCGGTGGCGATGATTCGACATCTATGAAACATGCAGCTTCTGTGTCTGATTTGACCTTTGTTGTTGATTATTCATAAAAGYGAAATYCCAGGATACACTCTCCCCGATAATCCAAAGTTCACCCTTGGTAATTTGTTTGTAATAATTGGRGTCTTGTTGGTTTGTGTTTTAGCTGTTGTCTCTCTTTTGAGAAATATTAGTGAGTCRGCCTTGTTCAAGAAGAATGGGTATGAACCGTTGGATTCRGATCCTAGTGTCATGAACCWAAACTTYGAGCCTACAACATTGTCCTTTGAAGATATTAAATATGAGGTTACTGGTGGTCGACAAATTTTAAATGGAGTCTTTGGGTTTGTAAAACCAAGAGAATGTTTGGCTATAATGGGAGGTTCAGGTGCTGGTAAAACTACATTGTTGGATTTTAAACCTTTGGACCAATTGGCTAAGACTTTGACCACTGTTCCTGAATTGAATGAAATTATTGGGCAAGATTTAGTTGACGAATTTGTCAGTGGTATTAAACTACCAGCAGAAGTTGGAAGTCAAGATGATGTTAACAATAGAAAATTGTTGCAAAAAGTGTTTGGTAAATTAATGAACACTGATGATGACGTTATAAAACAACAAACAGCTAAATTACTTGAAAGAACAGAMAGRGAACCTCAAGTGTTCAAGGATATTGATTCTAGATTACCRGAGTTAATACAARGATTAAACAAACAATTTCCTAATGACATCGGATTATTTTGTGGATGTCTCTTATTGAACCACGTTGMTTGAAAAAATTATCAATTTCCGTGTTGGATAAACARAGATTGACTGAAAAATTCAATAAATTGGATAAATCCATYAAAGATAATTTGAAGGCTAAACAAAAAGAAGAAACCAAAAARACTTTAGATGTGGTTAAYAATTGGTTGAATGATAAAGAAAATGCTTCATCATTTTTGGTTGCTCACGTTCCAATTACTGCTAATGCCAAGGCAATCACTGAAGCCATTAATTTGATTAAAAAGCAAGATAAAACCAAATCAATTTATTTATTGACTGGTGAAACCGATAAAGTTGCTCATGGATGTTATGTTAGTGATGAAGCCATTGYCAAGGGTATTAATGCGAAAATCAAACCTTTTGAAGCACGAGCAATTAACTGGTCMACGGATCTTAATGCTGAGGTACATATTGAGCATTATATAAATATATTCAATTATGCACGATCATCTTGGGAGCCATTGGTTGAAAGTTGGCCAATAGCAGTTTACATGTCAAAATCCCGACACCCAAAGCCTCAATTATTAGTAGAGGTGATTTCTAGACAGGTAGCTCAAGTGACGCTTACATCCAAAGCRGTAGCATTGCTATCTCAAGTATCCGATTTGATTACTTCCGRAGAAAAATTAAAACCAAGAGGTGAAGATTACCCATACRTTATAGTGAATGAGACTGGTTTAGATTTGGAAGTTTGGAATGATGCAAAWGAATCCGAAACCAATGGAGTTCCAATTGTTTTACGTGCTGGTAAAGCTTTAGATGAAAGTAAAGTTGAAATTAGAATTCAATTTAAACCAGTCGCCAAGGGGATGTTTAAAGAAATTCAAAGAAATGAATTAGTTATTAGAGTACAACCAAATGAAGCCATTTATTTAAAAATTAATTCCAAAATCCCTGGAATTTCTACTGAAACTTCATTAACTGATTTAGATTTAACTTATGCTACTCGTTATTCTAAAGAYTTTTGGATTCCTGAAGCTTATGAAGCATTAATTAGAGATTGTTATTTAGGTAATCATTCTAATTTTGTTAGAGATGATGAATTGGATGTTTCTTGGAAATTATTTACTCCATTATTGAAT

>Mi17_oral_6/07/2013_b

AATTATTGAAATGGAATGATATTCCATTGGCTCCACCAGACAAAATTTTGGGTATTTCTGAAGCTTATAACAATGATTCTAACCCTCAAAAAATCAATTTGGGGGTTGGTGCTTATAGAGATAATTCCGGTAAACCAATTATTTTCCCATCAGTTAAAAAAGCTGAAGAAATTTTATTGGGTAAAGAAACTGAAAAGGAATATACTGCCATTGTTGGTTCCAAAAATTTCCAATCAATTGTGAAAAATTTCATTTTCAACAATTCTAATAAAGATGCCAATGGTAAACAATTAATTGATGATGGTAGAATTGTTACTGCTCAAACCATCTCTGGTACTGGATCACTTAAACCTCTTATGGTGAAATTTTTGCTAAACATAGAGAACCAAATTTGGAAATTATTCGTGAGGTTGTTGATTCCAAACATATTGTTTTTGATGTGTTGGCACAATTCTTAATCAATCCAGACCCATGGGTTGCCATTGCTGCCGCTGAAGTTTATGTCAGACGTTCATACCGTGCTTATGATTTGGGTAAAATTGAATATCATGTTAATGACAGACTTCCTATTGTTGAATGGAAATTCAAGTTGGCTAATATGGGAGCYGCTGGTGTAAACGATGCTCAACAGGCTGCTGCTGCCGGTGGCGATGATTCGACATCTATGAAACATGCAGCTTCTGTGTCTGATTTGACCTTTGTTGTTGATTATTCATAAAAGYGAAATYCCAGGATACACTCTCCCCGATAATCCAAAGTTCACCCTTGGTAATTTGTTTGTAATAATTGGRGTCTTGTTGGTTTGTGTTTTAGCTGTTGTCTCTCTTTTGAGAAATATTAGTGAGTCRGCCTTGTTCAAGAAGAATGGGTATGAACCGTTGGATTCRGATCCTAGTGTCATGAACCWAAACTTYGAGCCTACAACATTGTCCTTTGAAGATATTAAATATGAGGTTACTGGTGGTCGACAAATTTTAAATGGAGTCTTTGGGTTTGTAAAACCAAGAGAATGTTTGGCTATAATGGGAGGTTCAGGTGCTGGTAAAACTACATTGTTGGATTTTAAACCTTTGGACCAATTGGCTAAGACTTTGACCACTGTTCCTGAATTGAATGAAATTATTGGGCAAGATTTAGTTGACGAATTTGTCAGTGGTATTAAACTACCAGCAGAAGTTGGAAGTCAAGATGATGTTAACAATAGAAAATTGTTGCAAAAAGTGTTTGGTAAATTAATGAACACTGATGATGACGTTATAAAACAACAAACAGCTAAATTACTTGAAAGAACAGAMAGRGAACCTCAAGTGTTCAAGGATATTGATTCTAGATTACCRGAGTTAATACAARGATTAAACAAACAATTTCCTAATGACATCGGATTATTTTGTGGATGTCTCTTATTGAACCACGTTGMTTGAAAAAATTATCAATTTCCGTGTTGGATAAACARAGATTGACTGAAAAATTCAATAAATTGGATAAATCCATYAAAGATAATTTGAAGGCTAAACAAAAAGAAGAAACCAAAAARACTTTAGATGTGGTTAAYAATTGGTTGAATGATAAAGAAAATGCTTCATCATTTTTGGTTGCTCACGTTCCAATTACTGCTAATGCCAAGGCAATCACTGAAGCCATTAATTTGATTAAAAAGCAAGATAAAACCAAATCAATTTATTTATTGACTGGTGAAACCGATAAAGTTGCTCATGGATGTTATGTTAGTGATGAAGCCATTGYCAAGGGTATTAATGCGAAAATCAAACCTTTTGAAGCACGAGCAATTAACTGGTCMACGGATCTTAATGCTGAGGTACATATTGAGCATTATATAAATATATTCAATTATGCACGATCATCTTGGGAGCCATTGGTTGAAAGTTGGCCAATAGCAGTTTACATGTCAAAATCCCGACACCCAAAGCCTCAATTATTAGTAGAGGTGATTTCTAGACAGGTAGCTCAAGTGACGCTTACATCCAAAGCRGTAGCATTGCTATCTCAAGTATCCGATTTGATTACTTCCGRAGAAAAATTAAAACCAAGAGGTGAAGATTACCCATACRTTATAGTGAATGAGACTGGTTTAGATTTGGAAGTTTGGAATGATGCAAAWGAATCCGAAACCAATGGAGTTCCAATTGTTTTACGTGCTGGTAAAGCTTTAGATGAAAGTAAAGTTGAAATTAGAATTCAATTTAAACCAGTCGCCAAGGGGATGTTTAAAGAAATTCAAAGAAATGAATTAGTTATTAGAGTACAACCAAATGAAGCCATTTATTTAAAAATTAATTCCAAAATCCCTGGAATTTCTACTGAAACTTCATTAACTGATTTAGATTTAACTTATGCTACTCGTTATTCTAAAGAYTTTTGGATTCCTGAAGCTTATGAAGCATTAATTAGAGATTGTTATTTAGGTAATCATTCTAATTTTGTTAGAGATGATGAATTGGATGTTTCTTGGAAATTATTTACTCCATTATTGAAT

>Mi17_pharynx_6/07/2013

AATTATTGAAATGGAATGATATTCCATTGGCTCCACCAGACAAAATTTTGGGTATTTCTGAAGCTTATAACAATGATTCTAACCCTCAAAAAATCAATTTGGGGGTTGGTGCTTATAGAGATAATTCCGGTAAACCAATTATTTTCCCATCAGTTAAAAAAGCTGAAGAAATTTTATTGGGTAAAGAAACTGAAAAGGAATATACTGCCATTGTTGGTTCCAAAAATTTCCAATCAATTGTGAAAAATTTCATTTTCAACAATTCTAATAAAGATGCCAATGGTAAACAATTAATTGATGATGGTAGAATTGTTACTGCTCAAACCATCTCTGGTACTGGATCACTTAAACCTCTTATGGTGAAATTTTTGCTAAACATAGAGAACCAAATTTGGAAATTATTCGTGAGGTTGTTGATTCCAAACATATTGTTTTTGATGTGTTGGCACAATTCTTAATCAATCCAGACCCATGGGTTGCCATTGCTGCCGCTGAAGTTTATGTCAGACGTTCATACCGTGCTTATGATTTGGGTAAAATTGAATATCATGTTAATGACAGACTTCCTATTGTTGAATGGAAATTCAAGTTGGCTAATATGGGAGCYGCTGGTGTAAACGATGCTCAACAGGCTGCTGCTGCCGGTGGCGATGATTCGACATCTATGAAACATGCAGCTTCTGTGTCTGATTTGACCTTTGTTGTTGATTATTCATAAAAGYGAAATYCCAGGATACACTCTCCCCGATAATCCAAAGTTCACCCTTGGTAATTTGTTTGTAATAATTGGRGTCTTGTTGGTTTGTGTTTTAGCTGTTGTCTCTCTTTTGAGAAATATTAGTGAGTCRGCCTTGTTCAAGAAGAATGGGTATGAACCGTTGGATTCRGATCCTAGTGTCATGAACCWAAACTTYGAGCCTACAACATTGTCCTTTGAAGATATTAAATATGAGGTTACTGGTGGTCGACAAATTTTAAATGGAGTCTTTGGGTTTGTAAAACCAAGAGAATGTTTGGCTATAATGGGAGGTTCAGGTGCTGGTAAAACTACATTGTTGGATTTTAAACCTTTGGACCAATTGGCTAAGACTTTGACCACTGTTCCTGAATTGAATGAAATTATTGGGCAAGATTTAGTTGACGAATTTGTCAGTGGTATTAAACTACCAGCAGAAGTTGGAAGTCAAGATGATGTTAACAATAGAAAATTGTTGCAAAAAGTGTTTGGTAAATTAATGAACACTGATGATGACGTTATAAAACAACAAACAGCTAAATTACTTGAAAGAACAGAMAGRGAACCTCAAGTGTTCAAGGATATTGATTCTAGATTACCRGAGTTAATACAARGATTAAACAAACAATTTCCTAATGACATCGGATTATTTTGTGGATGTCTCTTATTGAACCACGTTGMTTGAAAAAATTATCAATTTCCGTGTTGGATAAACARAGATTGACTGAAAAATTCAATAAATTGGATAAATCCATYAAAGATAATTTGAAGGCTAAACAAAAAGAAGAAACCAAAAARACTTTAGATGTGGTTAAYAATTGGTTGAATGATAAAGAAAATGCTTCATCATTTTTGGTTGCTCACGTTCCAATTACTGCTAATGCCAAGGCAATCACTGAAGCCATTAATTTGATTAAAAAGCAAGATAAAACCAAATCAATTTATTTATTGACTGGTGAAACCGATAAAGTTGCTCATGGATGTTATGTTAGTGATGAAGCCATTGYCAAGGGTATTAATGCGAAAATCAAACCTTTTGAAGCACGAGCAATTAACTGGTCMACGGATCTTAATGCTGAGGTACATATTGAGCATTATATAAATATATTCAATTATGCACGATCATCTTGGGAGCCATTGGTTGAAAGTTGGCCAATAGCAGTTTACATGTCAAAATCCCGACACCCAAAGCCTCAATTATTAGTAGAGGTGATTTCTAGACAGGTAGCTCAAGTGACGCTTACATCCAAAGCRGTAGCATTGCTATCTCAAGTATCCGATTTGATTACTTCCGRAGAAAAATTAAAACCAAGAGGTGAAGATTACCCATACRTTATAGTGAATGAGACTGGTTTAGATTTGGAAGTTTGGAATGATGCAAAWGAATCCGAAACCAATGGAGTTCCAATTGTTTTACGTGCTGGTAAAGCTTTAGATGAAAGTAAAGTTGAAATTAGAATTCAATTTAAACCAGTCGCCAAGGGGATGTTTAAAGAAATTCAAAGAAATGAATTAGTTATTAGAGTACAACCAAATGAAGCCATTTATTTAAAAATTAATTCCAAAATCCCTGGAATTTCTACTGAAACTTCATTAACTGATTTAGATTTAACTTATGCTACTCGTTATTCTAAAGAYTTTTGGATTCCTGAAGCTTATGAAGCATTAATTAGAGATTGTTATTTAGGTAATCATTCTAATTTTGTTAGAGATGATGAATTGGATGTTTCTTGGAAATTATTTACTCCATTATTGAAT

>OD8807

AATTATTGAARTGGAATGATATTCCATTGGCTCCACCAGACAARATTTTGGGTATTTCTGAAGCTTATAACAATGATTCTAACCCTCAAAAARTCAATTTGGGGGTTGGTGCTTATAGAGATAATTCYGGTAAACCAATTATTTTCCCATCAGTTAAAAAAGCTGAAGAAATTTTATTGGGTAAAGAAACTGAAAAGGAATATACTGCCATTGTTGGTTCCAAAAATTTCCAATCAATTGTGAAAAATTTCATTTTCAACAATTCTAATAAAGATGCCAATGGTAAACAATTAATTGATGATGGTAGAATTGTTACTGCTCAAACCATCTCTGGTACTGGATCACTTAAACCTCTTATGGTGAAATTTTTGCTAAACATAGAGAACCAAATTTGGAAATTATTCGTGAGGTTGTTGATTCCAAACATATTGTTTTTGATGTGTTGGCACAATTCTTAATCAATCCAGACCCATGGGTTGCCATTGCTGCCGCTGAAGTTTATGTCAGACGTTCATACCGTGCTTATGATTTGGGTACAATTGAATATCATGTTAATGACAGACTTCCTATTGTTGAATGGAAATTCAAGTTGGCTAATATGGGAGCCGCTGGTGTAAACGATGCTCAACAGGCTGCTGCTGCTGGTGGCGATGATTCGACATCTATGAAACATGCAGCTTCTGTGTCTGATTTGACCTTTGTTGTTGATTATTCATAAAAGCGAAATCCCAGGATACACTCTCCCCGATAATCCAAAGTTCACCCTTGGTAATTTGTTTGTAATAATTGGAGTCTTGTTGGTTTGTATTTTAGCTGTTGTCTCTCTTTTGAGAAATATTAGTGAGTCGGCCTTGTTCAAGAAGAATGGGTATGAACCGTTGGATTCGGATCCTAGTGTCATGAACCTAAACTTCGAGCCTACAACATTGTCCTTTGAAGATATTAAATATGAGGTTACTGGTGGTCGACAAATTTTAAATGGAGTCTTTGGGTTTGTAAAACCAAGAGAATGTTTGGCTATAATGGGAGGTTCAGGTGCTGGTAAAACTACATTGTTGGATTTTAAACCTTTGGACCAATTGGCTAARACTTTGRCYACTGTTCCTGAATTGAATGAAATTATTGGKCAAGAWTTAGTTGACGAATTTRTCAGTGGTATTAAACTACCAGCAGAAGTTGGAAGTCAAGATGATGTTAACAATAGAAAATTGTTGCAAAAAGTGTTTGGTAAATTAATGAACACTGATGATGACGTTATAAAACAACAAACAGCTAAATTACTTGAAAGAACAGACAGAGAACCTCAAGTGTTCAAGGATATTGATTCTAGATTACCGGAGTTAATACAAAGATTAAACAAACAATTTCCTAATGACATCGGATTATTTTGTGGATGTCTCTTATTGAACCACGTTGATTGAAAAAATTATCAATTTCCGTGTTGGATAAACAGAGATTGACTGAAAAATTCAATAAATTGGATAAATCCATCAAAGATAATTTGAAGGCTAAACAAAAAGAAGAAACCAAAAAAACTTTAGATGTGGTTAATAATTGGTTGAATGATAAAGAAAATGCTTCATCATTTTTGGTTGCTCACGTTCCAATTACTGCTAATGCCAAGGCAATCACTGAAGCCATTAATTTGATTAAAAAGCAAGATAAAACCAAATCAATTTATTTATTGACTGGTGAAACCGATAAAGTTGCTCATGGATGTTATGTTAGTGATGAAGCCATTGCCAAGGGTATTAATGCGAAAATCAAACCTTTTGAAGCACGAGCAATTAACTGGTCCACGGATCTTAATGCTGAGGTACATATTGAGCATTATATAAATATATTCAATTATGCACGATCATCTTGGGAGCCATTGGTTGAAAGTTGGCCAATAGCAGTTTACATGTCAAAATCCCGACACCCAAAGCCTCAATTATTAGTAGAGGTGATTTCTAGACAGGTAGCCCAAGTGACGCTTACATCCAAAGCAGTAGCATTGCTATCTCAAGTATCCGATTTGATTACTTCCGGAGAAAAATTAAAACCAAGAGGTGAAGATTACCCATACGTTATAGTGAATGAGACTGGTTTAGATTTGGAAGTTTGGAATGATGCAAAAGAATCCGAAACCAATGGAGTTCCAATTGTTTTACGTGCTGGTAAAGCTTTAGATGAAAGTAAAGTTGAAATTAGAATTCAATTTAAACCAGTCGCCAAGGGGATGTTTAAAGAAATTCAAAGAAATGAATTAGTTATTAGAGTACAACCAAATGAAGCCATTTATTTAAAAATTAATTCCAAAATCCCTGGAATTTCTACTGAAACTTCATTAACTGATTTAGATTTAACTTATGCTACTCGTTATTCTAAAGAYTTTTGGATTCCTGAAGCTTATGAAGCATTAATTAGAGATTGYTATTTAGGTAATCATTCTAATTTTGTTAGAGATGATGAATTGGATGTTTCTTGGAAATTATTTACTCCATTATTGAAT

>OD8824

AATTATTGAAATGGAATGATATTCCATTGGCTCCACCAGACAAAATTTTGGGTATTTCTGAAGCTTATAACAAYGATTCTAACCCTCAAAAAATCAATTTGGGGGTTGGTGCTTATAGAGATAATTCCGGTAAACCAATTATTTTCCCATCAGTTAAAAAAGCTGAAGAAATTTTATTGGGTAAAGAAACTGAAAAGGAATATACTGCCATTGTTGGTTCCAAAAATTTCCAATCAATTGTGAAAAATTTCATTTTCAACAATTCTAATAAAGATGCCAATGGTAAACAATTAATTGATGATGGTAGAATTGTTACTGCTCAAACCATCTCTGGTACTGGATCACTTAAACCTCTTATGGTGAAATTTTTGCTAAACATAGAGAACCAAATTTGGAAATTATTCGTGAGGTTGTTGATTCCAAACATATTGTTTTTGATGTGTTGGCACAATTCTTAATCAATCCAGACCCATGGGTTGCCATTGCTGCCGCTGAAGTTTATGTCAGACGTTCATACCGTGCTTATGATTTGGGTAMAATTGAATATCATGTTAATGACAGACTTCCTATTGTTGAATGGAAATTCAAGTTGGCTAATATGGGAGCCGCTGGTGTAAACGATGCTCAACAGGCTGCTGCTGCTGGTGGCGATGATTCGACATCTATGAAACATGCAGCTTCTGTGTCTGATTTGACCTTTGTTGTTGATTATTCATAAAAGYGAAATYCCAGGATACACTCTCCCCGATAATCCAAAGTTCACCCTTGGTAATTTGTTTGTAATAATTGGRGTCTTGTTGGTTTGTRTTTTAGCTGTTGTCTCTCTTTTGAGAAATATTAGTGAGTCRGCCTTGTTCAAGAAGAATGGGTATGAACCGTTGGATTCRGATCCTAGTGTCATGAACCWAAACTTYGAGCCTACAACATTGTCCTTTGAAGATATTAAATATGAGGTTACTGGTGGTCGACAAATTTTAAATGGAGTCTTTGGGTTTGTAAAACCAAGAGAATGTTTGGCTATAATGGGAGGTTCAGGTGCTGGTAAAACTACATTGTTGGATTTTAAACCTTTGGACCAATTGGCTAAGACTTTGACCACTGTTCCTGAATTGAATGAAATTATTGGGCAAGATTTAGTTGACGAATTTGTCAGTGGTATTAAACTACCAGCAGAAGTTGGAAGTCAAGATGATGTTAACAATAGAAAATTGTTGCAAAAAGTGTTTGGTAAATTAATGAACACTGATGATGACGTTATAAAACAACAAACAGCTAAATTACTTGAAAGAACAGAAAGGGAACCTCAAGTGTTCAAGGATATTGATTCTAGATTACCAGAGTTAATACAAGGATTAAACAAACAATTTCCTAATGACATCGGATTATTTTGTGGATGTCTCTTATTGAACCACGTTGMTTGAAAAAATTATCAATTTCCGTGTTGGATAAACAAAGATTGACTGAAAAATTCAATAAATTGGATAAATCCATTAAAGATAATTTGAAGGCTAAACAAAAAGAAGAAACCAAAAARACTTTAGATGTGGTTAACAATTGGTTGAATGATAAAGAAAATGCTTCATCATTTTTGGTTGCTCACGTTCCAATTACTGCTAATGCCAAGGCAATCACTGAAGCCATTAATTTGATTAAAAAGCAAGATAAAACCAAATCAATTTATTTATTGACTGGTGAAACAGATAAAGTTGCTCATGGATGTTATGTTAGTGATGAAGCCATTGCCAAGGGTATTAATGCGAAAATCAAACCTTTTGAAGCACGAGCAATTAACTGGTCAACGGATCTTAATGCTGAGGTACATATTGAGCATTATATAAATATATTCAATTATGCACGATCATCTTGGGAGCCATTGGTTGAAAGTTGGCCAATAGCAGTTTACATGTCAAAATCCCGACACCCAAAGCCTCAATTATTAGTAGAGGTGATTTCTAGACAGGTAGCTCAAGTGACGCTTACATCCAAAGCAGTAGCATTGCTATCTCAAGTATCCGATTTGATTACTTCCGGAGAAAAATTAAAACCAAGAGGTGAAGATTACCCATACATTATAGTGAATGAGACTGGTTTAGATTTGGAAGTTTGGAATGATGCAAAAGAATCCGAAACCAATGGAGTTCCAATTGTTTTACGTGCTGGTAAAGCTTTAGATGAAAGTAAAGTTGAAATTAGAATTCAATTTAAACCAGTCGCCAAGGGGATGTTTAAAGAAATTCAAAGAAATGAATTAGTTATTAGAGTACAACCAAATGAAGCCATTTATTTAAAAATTAATTCCAAAATCCCTGGAATTTCTACTGAAACTTCATTAACTGATTTAGATTTAACTTATGCTACTCGTTATTCTAAAGACTTTTGGATTCCTGAAGCTTATGAAGCATTAATTAGAGATTGTTATTTAGGTAATCATTCTAATTTTGTTAGAGATGATGAATTGGATGTTTCTTGGAAATTATTTACTCCATTATTGAAT

>OD8911

AATTATTGAARTGGAATGATATTCCATTGGCTCCACCAGACAARATTTTGGGTATTTCTGAAGCTTATAACAATGATTCTAACCCTCAAAAARTCAATTTGGGGGTTGGTGCTTATAGAGATAATTCYGGTAAACCAATTATTTTCCCATCAGTTAAAAAAGCTGAAGAAATTTTATTGGGTAAAGAAACTGAAAAGGAATATACTGCCATTGTTGGTTCCAAAAATTTCCAATCAATTGTGAAAAATTTCATTTTCAACAATTCTAATAAAGATGCCAATGGTAAACAATTAATTGATGATGGTAGAATTGTTACTGCTCAAACCATCTCTGGTACTGGATCACTTAAACCTCTTATGGTGAAATTTTTGCTAAACATAGAGAACCAAATTTGGAAATTATTCGTGAGGTTGTTGATTCCAAACATATTGTTTTTGATGTGTTGGCACAATTCTTAATCAATCCAGACCCATGGGTTGCCATTGCTGCCGCTGAAGTTTATGTCAGACGTTCATACCGTGCTTATGATTTGGGTAMAATTGAATATCATGTTAATGACAGACTTCCTATTGTTGAATGGAAATTCAAGTTGGCTAATATGGGAGCCGCTGGTGTAAACGATGCTCAACAGGCTGCTGCTGCYGGTGGCGATGATTCGACATCTATGAAACATGCAGCTTCTGTGTCTGATTTGACCTTTGTTGTTGATTATTCATRAAAGCGAAATCCCAGGATACACTCTCCCCGATAATCCAAAGTTCACCCTTGGTAATTTGTTTGTAATAATTGGAGTCTTGTTGGTTTGTATTTTAGCTGTTGTCTCTCTTTTGAGAAATATTAGTGAGTCGGCCTTGTTCAAGAAGAATGGGTATGAACCGTTGGATTCGGATCCTAGTGTCATGAACCTAAACTTCGAGCCTACAACATTGTCCTTTGAAGATATTAAATATGAGGTTACTGGTGGTCGACAAATTTTAAATGGAGTCTTTGGGTTTGTAAAACCAAGAGAATGTTTGGCTATAATGGGAGGTTCAGGTGCTGGTAAAACTACATTGTTGGATTTTAAACCTTTGGACCAATTGGCTAARACTTTGRCYACTGTTCCTGAATTGAATGAAATTATTGGKCAAGAWTTAGTTGACGAATTTRTCAGTGGTATTAAACTACCAGCAGAAGTTGGAAGTCAAGATGATGTTAACAATAGAAAATTGTTGCAAAAAGTGTTTGGTAAATTAATGAACACTGATGATGACGTTATAAAACAACAAACAGCTAAATTACTTGAAAGAACAGACAGAGAACCTCAAGTGTTCAAGGATATTGATTCTAGATTACCGGAGTTAATACAAAGATTAAACAAACAATTTCCTAATGACATCGGATTATTTTGTGGATGTCTCTTATTGAACCACGTTGATTGAAAAAATTATCAATTTCCGTGTTGGATAAACAGAGATTGACTGAAAAATTCAATAAATTGGATAAATCCATCAAAGATAATTTGAAGGCTAAACAAAAAGAAGAAACCAAAAAAACTTTAGATGTGGTTAATAATTGGTTGAATGATAAAGAAAATGCTTCATCATTTTTGGTTGCTCACGTTCCAATTACTGCTAATGCCAAGGCAATCACTGAAGCCATTAATTTGATTAAAAAGCAAGATAAAACCAAATCAATTTATTTATTGACTGGTGAAACCGATAAAGTTGCTCATGGATGTTATGTTAGTGATGAAGCCATTGCCAAGGGTATTAATGCGAAAATCAAACCTTTTGAAGCACGAGCAATTAACTGGTCCACGGATCTTAATGCTGAGGTACATATTGAGCATTATATAAATATATTCAATTATGCACGATCATCTTGGGAGCCATTGGTTGAAAGTTGGCCAATAGCAGTTTACATGTCAAAATCCCGACACCCAAAGCCTCAATTATTAGTAGAGGTGATTTCTAGACAGGTAGCYCAAGTGACGCTTACATCCAAAGCAGTAGCATTGCTATCTCAAGTATCCGATTTGATTACTTCCRGAGAAAAATTAAAACCAAGAGGTGAAGATTACCCATACGTKATAGTGAATGAGACTGGTTTAGATTTGGAAGTTTGGAATGATGCAAAWGAATYCGAAACCAATGGAGTTCCAATTGTTTTACGTGCTGGTAAAGCTTTAGATGAAAGTAAAGTTGAAATTAGAATTCAATTTAAACCAGTCGCCAAGGGGATGTTTAAAGAAATTCAAAGAAATGAATTAGTTATTAGAGTACAACCAAATGAAGCCATTTATTTAAAAATTAATTCCAAAATCCCTGGAATTTCTACTGAAACTTCATTAACTGATTTAGATTTAACTTATGCTACTCGTTATTCTAAAGAYTTTTGGATTCCTGAAGCTTATGAAGCATTAATTAGAGATTGYTATTTAGGTAATCATTCTAATTTTGTTAGAGATGATGAATTGGATGTTTCTTGGAAATTATTTACTCCATTATTGAAT

>OD8916

AATTATTGAARTGGAATGATATTCCATTGGCTCCACCAGACAAGATTTTGGGTATTTCTGAAGCTTATAACAATGATTCTAACCCTCAAAAAGTCAATTTGGGGGTTGGTGCTTATAGAGATAATTCTGGTAAACCAATTATTTTCCCATCAGTTAAAAAAGCTGAAGAAATTTTATTGGGTAAAGAAACTGAAAAGGAATATACTGCCATTGTTGGTTCCAAAAATTTCCAATCAATTGTGAAAAATTTCATTTTCAACAATTCTAATAAAGATGCCAATGGTAAACAATTAATTGATGATGGTAGAATTGTTACTGCTCAAACCATCTCTGGTACTGGATCACTTAAACCTCTTATGGTGAAATTTTTGCTAAACATAGAGAACCAAATTTGGAAATTATTCGTGAGGTTGTTGATTCCAAACATATTGTTTTTGATGTGTTGGCACAATTCTTAATCAATCCAGACCCATGGGTTGCCATTGCTGCCGCTGAAGTTTATGTCAGACGTTCATACCGTGCTTATGATTTGGGTAAAATTGAATATCATGTTAATGACAGACTTCCTATTGTTGAATGGAAATTCAAGTTGGCTAATATGGGAGCCGCTGGTGTAAACGATGCTCAACAGGCTGCTGCTGCYGGTGGCGATGATTCGACATCTATGAAACATGCAGCTTCTGTGTCTGATTTGACCTTTGTTGTTGATTATTCATRAAAGCGAAATCCCAGGATACACTCTCCCCGATAATCCAAAGTTCACCCTTGGTAATTTGTTTGTAATAATTGGAGTCTTGTTGGTTTGTATTTTAGCTGTTGTCTCTCTTTTGAGAAATATTAGTGAGTCGGCCTTGTTCAAGAAGAATGGGTATGAACCGTTGGATTCGGATCCTAGTGTCATGAACCTAAACTTCGAGCCTACAACATTGTCCTTTGAAGATATTAAATATGAGGTTACTGGTGGTCGACAAATTTTAAATGGAGTCTTTGGGTTTGTAAAACCAAGAGAATGTTTGGCTATAATGGGAGGTTCAGGTGCTGGTAAAACTACATTGTTGGATTTTAAACCTTTGGACCAATTGGCTAARACTTTGRCYACTGTTCCTGAATTGAATGAAATTATTGGKCAAGAWTTAGTTGACGAATTTRTCAGTGGTATTAAACTACCAGCAGAAGTTGGAAGTCAAGATGATGTTAACAATAGAAAATTGTTGCAAAAAGTGTTTGGTAAATTAATGAACACTGATGATGACGTTATAAAACAACAAACAGCTAAATTACTTGAAAGAACAGACAGAGAACCTCAAGTGTTCAAGGATATTGATTCTAGATTACCGGAGTTAATACAAAGATTAAACAAACAATTTCCTAATGACATCGGATTATTTTGTGGATGTCTCTTATTGAACCACGTTGATTGAAAAAATTATCAATTTCCGTGTTGGATAAACAGAGATTGACTGAAAAATTCAATAAATTGGATAAATCCATCAAAGATAATTTGAAGGCTAAACAAAAAGAAGAAACCAAAAAAACTTTAGATGTGGTTAATAATTGGTTGAATGATAAAGAAAATGCTTCATCATTTTTGGTTGCTCACGTTCCAATTACTGCTAATGCCAAGGCAATCACTGAAGCCATTAATTTGATTAAAAAGCAAGATAAAACCAAATCAATTTATTTATTGACTGGTGAAACCGATAAAGTTGCTCATGGATGTTATGTTAGTGATGAAGCCATTGCCAAGGGTATTAATGCGAAAATCAAACCTTTTGAAGCACGAGCAATTAACTGGTCCACGGATCTTAATGCTGAGGTACATATTGAGCATTATATAAATATATTCAATTATGCACGATCATCTTGGGAGCCATTGGTTGAAAGTTGGCCAATAGCAGTTTACATGTCAAAATCCCGACACCCAAAGCCTCAATTATTAGTAGAGGTGATTTCTAGACAGGTAGCTCAAGTGACGCTTACATCCAAAGCAGTAGCATTGCTATCTCAAGTATCCGATTTGATTACTTCCRGAGAAAAATTAAAACCAAGAGGTGAAGATTACCCATACGTKATAGTGAATGAGACTGGTTTAGATTTGGAAGTTTGGAATGATGCAAAWGAATYCGAAACCAATGGAGTTCCAATTGTTTTACGTGCTGGTAAAGCTTTAGATGAAAGTAAAGTTGAAATTAGAATTCAATTTAAACCAGTCGCCAAGGGGATGTTTAAAGAAATTCAAAGAAATGAATTAGTTATTAGAGTACAACCAAATGAAGCCATTTATTTAAAAATTAATTCCAAAATCCCTGGAATTTCTACTGAAACTTCATTAACTGATTTAGATTTAACTTATGCTACTCGTTATTCTAAAGAYTTTTGGATTCCTGAAGCTTATGAAGCATTAATTAGAGATTGYTATTTAGGTAATCATTCTAATTTTGTTAGAGATGATGAATTGGATGTTTCTTGGAAATTATTTACTCCATTATTGAAT

>RIHO9

AATTATTGAARTGGAATGATATTCCATTGGCTCCACCAGACAARATTTTGGGTATTTCTGAAGCTTATAACAATGATTCTAACCCTCAAAAARTCAATTTGGGGGTTGGTGCTTATAGAGATAATTCYGGTAAACCAATTATTTTCCCATCAGTTAAAAAAGCTGAAGAAATTTTATTGGGTAAAGAAACTGAAAAGGAATATACTGCCATTGTTGGTTCCAAAAATTTCCAATCAATTGTGAAAAATTTCATTTTCAACAATTCTAATAAAGATGCCAATGGTAAACAATTAATTGATGATGGTAGAATTGTTACTGCTCAAACCATCTCTGGTACTGGATCACTTAAACCTCTTATGGTGAAATTTTTGCTAAACATAGAGAACCAAATTTGGAAATTATTCGTGAGGTTGTTGATTCCAAACATATTGTTTTTGATGTGTTGGCACAATTCTTAATCAATCCAGACCCATGGGTTGCCATTGCTGCCGCTGAAGTTTATGTCAGACGTTCATACCGTGCTTATGATTTGGGTAAAATTGAATATCATGTTAATGACAGACTTCCTATTGTTGAATGGAAATTCAAGTTGGCTAATATGGGAGCCGCTGGTGTAAACGATGCTCAACAGGCTGCTGCTGCCGGTGGCGATGATTCGACATCTATGAAACATGCAGCTTCTGTGTCTGATTTGACCTTTGTTGTTGATTATTCATRAAAGCGAAATCCCAGGATACACTCTCCCCGATAATCCAAAGTTCACCCTTGGTAATTTGTTTGTAATAATTGGAGTCTTGTTGGTTTGTATTTTAGCTGTTGTCTCTCTTTTGAGAAATATTAGTGAGTCGGCCTTGTTCAAGAAGAATGGGTATGAACCGTTGGATTCGGATCCTAGTGTCATGAACCWAAACTTCGAGCCTACAACATTGTCCTTTGAAGATATTAAATATGAGGTTACTGGTGGTCGACAAATTTTAAATGGAGTCTTTGGGTTTGTAAAACCAAGAGAATGTTTGGCTATAATGGGAGGTTCAGGTGCTGGTAAAACTACATTGTTGGATTTTAAACCTTTGGACCAATTGGCTAARACTTTGRCYACTGTTCCTGAATTGAATGAAATTATTGGKCAAGAWTTAGTTGACGAATTTRTCAGTGGTATTAAACTACCAGCAGAAGTTGGAAGTCAAGATGATGTTAACAATAGAAAATTGTTGCAAAAAGTGTTTGGTAAATTAATGAACACTGATGATGACGTTATAAAACAACAAACAGCTAAATTACTTGAAAGAACAGACAGAGAACCTCAAGTGTTCAAGGATATTGATTCTAGATTACCGGAGTTAATACAAAGATTAAACAAACAATTTCCTAATGACATCGGATTATTTTGTGGATGTCTCTTATTGAACCACGTTGATTGAAAAAATTATCAATTTCCGTGTTGGATAAACAGAGATTGACTGAAAAATTCAATAAATTGGATAAATCCATCAAAGATAATTTGAAGGCTAAACAAAAAGAAGAAACCAAAAAAACTTTAGATGTGGTTAATAATTGGTTGAATGATAAAGAAAATGCTTCATCATTTTTGGTTGCTCACGTTCCAATTACTGCTAATGCCAAGGCAATCACTGAAGCCATTAATTTGATTAAAAAGCAAGATAAAACCAAATCAATTTATTTATTGACTGGTGAAACCGATAAAGTTGCTCATGGATGTTATGTTAGTGATGAAGCCATTGCCAAGGGTATTAATGCGAAAATCAAACCTTTTGAAGCACGAGCAATTAACTGGTCCACGGATCTTAATGCTGAGGTACATATTGAGCATTATATAAATATATTCAATTATGCACGATCATCTTGGGAGCCATTGGTTGAAAGTTGGCCAATAGCAGTTTACATGTCAAAATCCCGACACCCAAAGCCTCAATTATTAGTAGAGGTGATTTCTAGACAGGTAGCYCAAGTGACGCTTACATCCAAAGCAGTAGCATTGCTATCTCAAGTATCCGATTTGATTACTTCCRGAGAAAAATTAAAACCAAGAGGTGAAGATTACCCATACGTKATAGTGAATGAGACTGGTTTAGATTTGGAAGTTTGGAATGATGCAAAWGAATYCGAAACCAATGGAGTTCCAATTGTTTTACGTGCTGGTAAAGCTTTAGATGAAAGTAAAGTTGAAATTAGAATTCAATTTAAACCAGTCGCCAAGGGGATGTTTAAAGAAATTCAAAGAAATGAATTAGTTATTAGAGTACAACCAAATGAAGCCATTTATTTAAAAATTAATTCCAAAATCCCTGGAATTTCTACTGAAACTTCATTAACTGATTTAGATTTAACTTATGCTACTCGTTATTCTAAAGAYTTTTGGATTCCTGAAGCTTATGAAGCATTAATTAGAGATTGYTATTTAGGTAATCATTCTAATTTTGTTAGAGATGATGAATTGGATGTTTCTTGGAAATTATTTACTCCATTATTGAAT

>RIHO10

AATTATTGAARTGGAATGATATTCCATTGGCTCCACCAGACAARATTTTGGGTATTTCTGAAGCTTATAACAATGATTCTAACCCTCAAAAARTCAATTTGGGGGTTGGTGCTTATAGAGATAATTCYGGTAAACCAATTATTTTCCCATCAGTTAAAAAAGCTGAAGAAATTTTATTGGGTAAAGAAACTGAAAAGGAATATACTGCCATTGTTGGTTCCAAAAATTTCCAATCAATTGTGAAAAATTTCATTTTCAACAATTCTAATAAAGATGCCAATGGTAAACAATTAATTGATGATGGTAGAATTGTTACTGCTCAAACCATCTCTGGTACTGGATCACTTAAACCTCTTATGGTGAAMTTTTTGCTAAACATAGAGAACCAAATTTGGAAATTATTCGTGAGGTTGTTGATTCCAAACATATTGTTTTTGATGTGTTGGCACAATTCTTAATCAATCCAGACCCATGGGTTGCCATTGCTGCCGCTGAAGTTTATGTCAGACGTTCATACCGTGCTTATGATTTGGGTAMAATTGAATATCATGTTAATGACAGACTTCCTATTGTTGAATGGAAATTCAAGTTGGCTAATATGGGAGCCGCTGGTGTAAACGATGCTCAACAGGCTGCTGCTGCYGGTGGCGATGATTCGACATCTATGAAACATGCAGCTTCTGTGTCTGATTTGACCTTTGTTGTTGATTATTCATRAAAGCGAAATCCCAGGATACACTCTCCCCGATAATCCAAAGTTCACCCTTGGTAATTTGTTTGTAATAATTGGAGTCTTGTTGGTTTGTATTTTAGCTGTTGTCTCTCTTTTGAGAAATATTAGTGAGTCGGCCTTGTTCAAGAAGAATGGGTATGAACCGTTGGATTCGGATCCTAGTGTCATGAACCTAAACTTCGAGCCTACAACATTGTCCTTTGAAGATATTAAATATGAGGTTACTGGTGGTCGACAAATTTTAAATGGAGTCTTTGGGTTTGTAAAACCAAGAGAATGTTTGGCTATAATGGGAGGTTCAGGTGCTGGTAAAACTACATTGTTGGATTTTAAACCTTTGGACCAATTGGCTAARACTTTGRCYACTGTTCCTGAATTGAATGAAATTATTGGKCAAGAWTTAGTTGACGAATTTRTCAGTGGTATTAAACTACCAGCAGAAGTTGGAAGTCAAGATGATGTTAACAATAGAAAATTGTTGCAAAAAGTGTTTGGTAAATTAATGAACACTGATGATGACGTTATAAAACAACAAACAGCTAAATTACTTGAAAGAACAGACAGAGAACCTCAAGTGTTCAAGGATATTGATTCTAGATTACCGGAGTTAATACAAAGATTAAACAAACAATTTCCTAATGACATCGGATTATTTTGTGGATGTCTCTTATTGAACCACGTTGATTGAAAAAATTATCAATTTCCGTGTTGGATAAACAGAGATTGACTGAAAAATTCAATAAATTGGATAAATCCATCAAAGATAATTTGAAGGCTAAACAAAAAGAAGAAACCAAAAAAACTTTAGATGTGGTTAATAATTGGTTGAATGATAAAGAAAATGCTTCATCATTTTTGGTTGCTCACGTTCCAATTACTGCTAATGCCAAGGCAATCACTGAAGCCATTAATTTGATTAAAAAGCAAGATAAAACCAAATCAATTTATTTATTGACTGGTGAAACCGATAAAGTTGCTCATGGATGTTATGTTAGTGATGAAGCCATTGCCAAGGGTATTAATGCGAAAATCAAACCTTTTGAAGCACGAGCAATTAACTGGTCCACGGATCTTAATGCTGAGGTACATATTGAGCATTATATAAATATATTCAATTATGCACGATCATCTTGGGAGCCATTGGTTGAAAGTTGGCCAATAGCAGTTTACATGTCAAAATCCCGACACCCAAAGCCTCAATTATTAGTAGAGGTGATTTCTAGACAGGTAGCYCAAGTGACGCTTACATCCAAAGCAGTAGCATTGCTATCTCAAGTATCCGATTTGATTACTTCCRGAGAAAAATTAAAACCAAGAGGTGAAGATTACCCATACGTKATAGTGAATGAGACTGGTTTAGATTTGGAAGTTTGGAATGATGCAAAWGAATYCGAAACCAATGGAGTTCCAATTGTTTTACGTGCTGGTAAAGCTTTAGATGAAAGTAAAGTTGAAATTAGAATTCAATTTAAACCAGTCGCCAAGGGGATGTTTAAAGAAATTCAAAGAAATGAATTAGTTATTAGAGTACAACCAAATGAAGCCATTTATTTAAAAATTAATTCCAAAATCCCTGGAATTTCTACTGAAACTTCATTAACTGATTTAGATTTAACTTATGCTACTCGTTATTCTAAAGAYTTTTGGATTCCTGAAGCTTATGAAGCATTAATTAGAGATTGYTATTTAGGTAATCATTCTAATTTTGTTAGAGATGATGAATTGGATGTTTCTTGGAAATTATTTACTCCATTATTGAAT

>RIHO13

AATTATTGAAATGGAATGATATTCCATTGGCTCCACCAGACAAGATTTTGGGTATTTCTGAAGCTTATAACAATGATTCTAACCCTCAAAAAGTCAATTTGGGGGTTGGTGCTTATAGAGATAATTCTGGTAAACCAATTATTTTCCCATCAGTTAAAAAAGCTGAAGAAATTTTATTGGGTAAAGAAACTGAAAAGGAATATACTGCCATTGTTGGTTCCAAAAATTTCCAATCAATTGTGAAAAATTTCATTTTCAACAATTCTAATAAAGATGCCAATGGTAAACAATTAATTGATGATGGTAGAATTGTTACTGCTCAAACCATCTCTGGTACTGGATCACTTAAACCTCTTATGGTGAAATTTTTGCTAAACATAGAGAACCAAATTTGGAAATTATTCGTGAGGTTGTTGATTCCAAACATATTGTTTTTGATGTGTTGGCACAATTCTTAATCAATCCAGACCCATGGGTTGCCATTGCTGCCGCTGAAGTTTATGTCAGACGTTCATACCGTGCTTATGATTTGGGTAMAATTGAATATCATGTTAATGACAGACTTCCTATTGTTGAATGGAAATTCAAGTTGGCTAATATGGGAGCCGCTGGTGTAAACGATGCTCAACAGGCTGCTGCTGCYGGTGGCGATGATTCGACATCTATGAAACATGCAGCTTCTGTGTCTGATTTGACCTTTGTTGTTGATTATTCATRAAAGCGAAATCCCAGGATACACTCTCCCCGATAATCCAAAGTTCACCCTTGGTAATTTGTTTGTAATAATTGGAGTCTTGTTGGTTTGTATTTTAGCTGTTGTCTCTCTTTTGAGAAATATTAGTGAGTCGGCCTTGTTCAAGAAGAATGGGTATGAACCGTTGGATTCGGATCCTAGTGTCATGAACCTAAACTTCGAGCCTACAACATTGTCCTTTGAAGATATTAAATATGAGGTTACTGGTGGTCGACAAATTTTAAATGGAGTCTTTGGGTTTGTAAAACCAAGAGAATGTTTGGCTATAATGGGAGGTTCAGGTGCTGGTAAAACTACATTGTTGGATTTTAAACCTTTGGACCAATTGGCTAAGACTTTGRCCACTGTTCCTGAATTGAATGAAATTATTGGGCAAGATTTAGTTGACGAATTTRTCAGTGGTATTAAACTACCAGCAGAAGTTGGAAGTCAAGATGATGTTAACAATAGAAAATTGTTGCAAAAAGTGTTTGGTAAATTAATGAACACTGATGATGACGTTATAAAACAACAAACAGCTAAATTACTTGAAAGAACAGACAGAGAACCTCAAGTGTTCAAGGATATTGATTCTAGATTACCGGAGTTAATACAAAGATTAAACAAACAATTTCCTAATGACATCGGATTATTTTGTGGATGTCTCTTATTGAACCACGTTGATTGAAAAAATTATCAATTTCCGTGTTGGATAAACAGAGATTGACTGAAAAATTCAATAAATTGGATAAATCCATCAAAGATAATTTGAAGGCTAAACAAAAAGAAGAAACCAAAAAAACTTTAGATGTGGTTAATAATTGGTTGAATGATAAAGAAAATGCTTCATCATTTTTGGTTGCTCACGTTCCAATTACTGCTAATGCCAAGGCAATCACTGAAGCCATTAATTTGATTAAAAAGCAAGATAAAACCAAATCAATTTATTTATTGACTGGTGAAACCGATAAAGTTGCTCATGGATGTTATGTTAGTGATGAAGCCATTGCCAAGGGTATTAATGCGAAAATCAAACCTTTTGAAGCACGAGCAATTAACTGGTCCACGGATCTTAATGCTGAGGTACATATTGAGCATTATATAAATATATTCAATTATGCACGATCATCTTGGGAGCCATTGGTTGAAAGTTGGCCAATAGCAGTTTACATGTCAAAATCCCGACACCCAAAGCCTCAATTATTAGTAGAGGTGATTTCTAGACAGGTAGCYCAAGTGACGCTTACATCCAAAGCAGTAGCATTGCTATCTCAAGTATCCGATTTGATTACTTCCRGAGAAAAATTAAAACCAAGAGGTGAAGATTACCCATACGTKATAGTGAATGAGACTGGTTTAGATTTGGAAGTTTGGAATGATGCAAAWGAATYCGAAACCAATGGAGTTCCAATTGTTTTACGTGCTGGTAAAGCTTTAGATGAAAGTAAAGTTGAAATTAGAATTCAATTTAAACCAGTCGCCAAGGGGATGTTTAAAGAAATTCAAAGAAATGAATTAGTTATTAGAGTACAACCAAATGAAGCCATTTATTTAAAAATTAATTCCAAAATCCCTGGAATTTCTACTGAAACTTCATTAACTGATTTAGATTTAACTTATGCTACTCGTTATTCTAAAGAYTTTTGGATTCCTGAAGCTTATGAAGCATTAATTAGAGATTGYTATTTAGGTAATCATTCTAATTTTGTTAGAGATGATGAATTGGATGTTTCTTGGAAATTATTTACTCCATTATTGAAT

>RIHO16

AATTATTGAARTGGAATGATATTCCATTGGCTCCACCAGACAARATTTTGGGTATTTCTGAAGCTTATAACAATGATTCTAACCCTCAAAAARTCAATTTGGGGGTTGGTGCTTATAGAGATAATTCYGGTAAACCAATTATTTTCCCATCAGTTAAAAAAGCTGAAGAAATTTTATTGGGTAAAGAAACTGAAAAGGAATATACTGCCATTGTTGGTTCCAAAAATTTCCAATCAATTGTGAAAAATTTCATTTTCAACAATTCTAATAAAGATGCCAATGGTAAACAATTAATTGATGATGGTAGAATTGTTACTGCTCAAACCATCTCTGGTACTGGATCACTTAAACCTCTTATGGTGAAATTTTTGCTAAACATAGAGAACCAAATTTGGAAATTATTCGTGAGGTTGTTGATTCCAAACATATTGTTTTTGATGTGTTGGCACAATTCTTAATCAATCCAGACCCATGGGTTGCCATTGCTGCCGCTGAAGTTTATGTCAGACGTTCATACCGTGCTTATGATTTGGGTAMAATTGAATATCATGTTAATGACAGACTTCCTATTGTTGAATGGAAATTCAAGTTGGCTAATATGGGAGCCGCTGGTGTAAACGATGCTCAACAGGCTGCTGCTGCYGGTGGCGATGATTCGACATCTATGAAACATGCAGCTTCTGTGTCTGATTTGACCTTTGTTGTTGATTATTCATRAAAGCGAAATCCCAGGATACACTCTCCCCGATAATCCAAAGTTCACCCTTGGTAATTTGTTTGTAATAATTGGAGTCTTGTTGGTTTGTATTTTAGCTGTTGTCTCTCTTTTGAGAAATATTAGTGAGTCGGCCTTGTTCAAGAAGAATGGGTATGAACCGTTGGATTCGGATCCTAGTGTCATGAACCTAAACTTCGAGCCTACAACATTGTCCTTTGAAGATATTAAATATGAGGTTACTGGTGGTCGACAAATTTTAAATGGAGTCTTTGGGTTTGTAAAACCAAGAGAATGTTTGGCTATAATGGGAGGTTCAGGTGCTGGTAAAACTACATTGTTGGATTTTAAACCTTTGGACCAATTGGCTAARACTTTGRCYACTGTTCCTGAATTGAATGAAATTATTGGKCAAGAWTTAGTTGACGAATTTRTCAGTGGTATTAAACTACCAGCAGAAGTTGGAAGTCAAGATGATGTTAACAATAGAAAATTGTTGCAAAAAGTGTTTGGTAAATTAATGAACACTGATGATGACGTTATAAAACAACAAACAGCTAAATTACTTGAAAGAACAGACAGAGAACCTCAAGTGTTCAAGGATATTGATTCTAGATTACCGGAGTTAATACAAAGATTAAACAAACAATTTCCTAATGACATCGGATTATTTTGTGGATGTCTCTTATTGAACCACGTTGATTGAAAAAATTATCAATTTCCGTGTTGGATAAACAGAGATTGACTGAAAAATTCAATAAATTGGATAAATCCATCAAAGATAATTTGAAGGCTAAACAAAAAGAAGAAACCAAAAAAACTTTAGATGTGGTTAATAATTGGTTGAATGATAAAGAAAATGCTTCATCATTTTTGGTTGCTCACGTTCCAATTACTGCTAATGCCAAGGCAATCACTGAAGCCATTAATTTGATTAAAAAGCAAGATAAAACCAAATCAATTTATTTATTGACTGGTGAAACCGATAAAGTTGCTCATGGATGTTATGTTAGTGATGAAGCCATTGCCAAGGGTATTAATGCGAAAATCAAACCTTTTGAAGCACGAGCAATTAACTGGTCCACGGATCTTAATGCTGAGGTACATATTGAGCATTATATAAATATATTCAATTATGCACGATCATCTTGGGAGCCATTGGTTGAAAGTTGGCCAATAGCAGTTTACATGTCAAAATCCCGACACCCAAAGCCTCAATTATTAGTAGAGGTGATTTCTAGACAGGTAGCYCAAGTGACGCTTACATCCAAAGCAGTAGCATTGCTATCTCAAGTATCCGATTTGATTACTTCCRGAGAAAAATTAAAACCAAGAGGTGAAGATTACCCATACGTKATAGTGAATGAGACTGGTTTAGATTTGGAAGTTTGGAATGATGCAAAWGAATYCGAAACCAATGGAGTTCCAATTGTTTTACGTGCTGGTAAAGCTTTAGATGAAAGTAAAGTTGAAATTAGAATTCAATTTAAACCAGTCGCCAAGGGGATGTTTAAAGAAATTCAAAGAAATGAATTAGTTATTAGAGTACAACCAAATGAAGCCATTTATTTAAAAATTAATTCCAAAATCCCTGGAATTTCTACTGAAACTTCATTAACTGATTTAGATTTAACTTATGCTACTCGTTATTCTAAAGAYTTTTGGATTCCTGAAGCTTATGAAGCATTAATTAGAGATTGYTATTTAGGTAATCATTCTAATTTTGTTAGAGATGATGAATTGGATGTTTCTTGGAAATTATTTACTCCATTATTGAAT

>SC5314

AATTATTGAARTGGAATGATATTCCATTGGCTCCACCAGACAARATTTTGGGTATTTCTGAAGCTTATAACAATGATTCTAACCCTCAAAAARTCAATTTGGGGGTTGGTGCTTATAGAGATAATTCYGGTAAACCAATTATTTTCCCATCAGTTAAAAAAGCTGAAGAAATTTTATTGGGTAAAGAAACTGAAAAGGAATATACTGCCATTGTTGGTTCCAAAAATTTCCAATCAATTGTGAAAAATTTCATTTTCAACAATTCTAATAAAGATGCCAATGGTAAACAATTAATTGATGATGGTAGAATTGTTACTGCTCAAACCATCTCTGGTACTGGATCACTTAAACCTCTTATGGTGAAATTTTTGCTAAACATAGAGAACCAAATTTGGAAATTATTCGTGAGGTTGTTGATTCCAAACATATTGTTTTTGATGTGTTGGCACAATTCTTAATCAATCCAGACCCATGGGTTGCCATTGCTGCCGCTGAAGTTTATGTCAGACGTTCATACCGTGCTTATGATTTGGGTAAAATTGAATATCATGTTAATGACAGACTTCCTATTGTTGAATGGAAATTCAAGTTGGCTAATATGGGAGCCGCTGGTGTAAACGATGCTCAACAGGCTGCTGCTGCCGGTGGCGATGATTCGACATCTATGAAACATGCAGCTTCTGTGTCTGATTTGACCTTTGTTGTTGATTATTCATRAAAGCGAAATCCCAGGATACACTCTCCCCGATAATCCAAAGTTCACCCTTGGTAATTTGTTTGTAATAATTGGAGTCTTGTTGGTTTGTATTTTAGCTGTTGTCTCTCTTTTGAGAAATATTAGTGAGTCGGCCTTGTTCAAGAAGAATGGGTATGAACCGTTGGATTCGGATCCTAGTGTCATGAACCTAAACTTCGAGCCTACAACATTGTCCTTTGAAGATATTAAATATGAGGTTACTGGTGGTCGACAAATTTTAAATGGAGTCTTTGGGTTTGTAAAACCAAGAGAATGTTTGGCTATAATGGGAGGTTCAGGTGCTGGTAAAACTACATTGTTGGATTTTAAACCTTTGGACCAATTGGCTAAAACTTTGGCTACTGTTCCTGAATTGAATGAAATTATTGGTCAAGAATTAGTTGACGAATTTATCAGTGGTATTAAACTACCAGCAGAAGTTGGAAGTCAAGATGATGTTAACAATAGAAAATTGTTGCAAAAAGTGTTTGGTAAATTAATGAACACTGATGATGACGTTATAAAACAACAAACAGCTAAATTACTTGAAAGAACAGACAGAGAACCTCAAGTGTTCAAGGATATTGATTCTAGATTACCGGAGTTAATACAAAGATTAAACAAACAATTTCCTAATGACATCGGATTATTTTGTGGATGTCTCTTATTGAACCACGTTGATTGAAAAAATTATCAATTTCCGTGTTGGATAAACAGAGATTGACTGAAAAATTCAATAAATTGGATAAATCCATCAAAGATAATTTGAAGGCTAAACAAAAAGAAGAAACCAAAAAAACTTTAGATGTGGTTAATAATTGGTTGAATGATAAAGAAAATGCTTCATCATTTTTGGTTGCTCACGTTCCAATTACTGCTAATGCCAAGGCAATCACTGAAGCCATTAATTTGATTAAAAAGCAAGATAAAACCAAATCAATTTATTTATTGACTGGTGAAACCGATAAAGTTGCTCATGGATGTTATGTTAGTGATGAAGCCATTGCCAAGGGTATTAATGCGAAAATCAAACCTTTTGAAGCACGAGCAATTAACTGGTCCACGGATCTTAATGCTGAGGTACATATTGAGCATTATATAAATATATTCAATTATGCACGATCATCTTGGGAGCCATTGGTTGAAAGTTGGCCAATAGCAGTTTACATGTCAAAATCCCGACACCCAAAGCCTCAATTATTAGTAGAGGTGATTTCTAGACAGGTAGCTCAAGTGACGCTTACATCCAAAGCAGTAGCATTGCTATCTCAAGTATCCGATTTGATTACTTCCAGAGAAAAATTAAAACCAAGAGGTGAAGATTACCCATACGTGATAGTGAATGAGACTGGTTTAGATTTGGAAGTTTGGAATGATGCAAATGAATTCGAAACCAATGGAGTTCCAATTGTTTTACGTGCTGGTAAAGCTTTAGATGAAAGTAAAGTTGAAATTAGAATTCAATTTAAACCAGTCGCCAAGGGGATGTTTAAAGAAATTCAAAGAAATGAATTAGTTATTAGAGTACAACCAAATGAAGCCATTTATTTAAAAATTAATTCCAAAATCCCTGGAATTTCTACTGAAACTTCATTAACTGATTTAGATTTAACTTATGCTACTCGTTATTCTAAAGAYTTTTGGATTCCTGAAGCTTATGAAGCATTAATTAGAGATTGYTATTTAGGTAATCATTCTAATTTTGTTAGAGATGATGAATTGGATGTTTCTTGGAAATTATTTACTCCATTATTGAAT

>sw-17c

AATTATTGAAATGGAATGATATTCCATTGGCTCCACCAGACAAAATTTTGGGTATTTCTGAAGCTTATAACAATGATTCTAACCCTCAAAAAATCAATTTGGGGGTTGGTGCTTATAGAGATAATTCCGGTAAACCAATTATTTTCCCATCAGTTAAAAAAGCTGAAGAAATTTTATTGGGTAAAGAAACTGAAAAGGAATATACTGCCATTGTTGGTTCCAAAAATTTCCAATCAATTGTGAAAAATTTCATTTTCAACAATTCTAATAAAGATGCCAATGGTAAACAATTAATTGATGATGGTAGAATTGTTACTGCTCAAACCATCTCTGGTACTGGATCACTTAAACCTCTTATGGTGAAATTTTTGCTAAACATAGAGAACCAAATTTGGAAATTATTCGTGAGGTTGTTGATTCCAAACATATTGTTTTTGATGTGTTGGCACAATTCTTAATCAATCCAGACCCATGGGTTGCCATTGCTGCCGCTGAAGTTTATGTCAGACGTTCATACCGTGCTTATGATTTGGGTAAAATTGAATATCATGTTAATGACAGACTTCCTATTGTTGAATGGAAATTCAAGTTGGCTAATATGGGAGCYGCTGGTGTAAACGATGCTCAACAGGCTGCTGCTGCCGGTGGCGATGATTCGACATCTATGAAACATGCAGCTTCTGTGTCTGATTTGACCTTTGTTGTTGATTATTCATAAAAGYGAAATYCCAGGATACACTCTCCCCGATAATCCAAAGTTCACCCTTGGTAATTTGTTTGTAATAATTGGRGTCTTGTTGGTTTGTGTTTTAGCTGTTGTCTCTCTTTTGAGAAATATTAGTGAGTCRGCCTTGTTCAAGAAGAATGGGTATGAACCGTTGGATTCGGATCCTAGTGTCATGAACCTAAACTTCGAGCCTACAACATTGTCCTTTGAAGATATTAAATATGAGGTTACTGGTGGTCGACAAATTTTAAATGGAGTCTTTGGGTTTGTAAAACCAAGAGAATGTTTGGCTATAATGGGAGGTTCAGGTGCTGGTAAAACTACATTGTTGGATTTTAAACCTTTGGACCAATTGGCTAAGACTTTGACCACTGTTCCTGAATTGAATGAAATTATTGGGCAAGATTTAGTTGACGAATTTGTCAGTGGTATTAAACTACCAGCAGAAGTTGGAAGTCAAGATGATGTTAACAATAGAAAATTGTTGCAAAAAGTGTTTGGTAAATTAATGAACACTGATGATGACGTTATAAAACAACAAACAGCTAAATTACTTGAAAGAACAGAMAGRGAACCTCAAGTGTTCAAGGATATTGATTCTAGATTACCRGAGTTAATACAARGATTAAACAAACAATTTCCTAATGACATCGGATTATTTTGTGGATGTCTCTTATTGAACCACGTTGMTTGAAAAAATTATCAATTTCCGTGTTGGATAAACARAGATTGACTGAAAAATTCAATAAATTGGATAAATCCATYAAAGATAATTTGAAGGCTAAACAAAAAGAAGAAACCAAAAAAACTTTAGATGTGGTTAAYAATTGGTTGAATGATAAAGAAAATGCTTCATCATTTTTGGTTGCTCACGTTCCAATTACTGCTAATGCCAAGGCAATCACTGAAGCCATTAATTTGATTAAAAAGCAAGATAAAACCAAATCAATTTATTTATTGACTGGTGAAACCGATAAAGTTGCTCATGGATGTTATGTTAGTGATGAAGCCATTGYCAAGGGTATTAATGCGAAAATCAAACCTTTTGAAGCACGAGCAATTAACTGGTCAACGGATCTTAATGCTGAGGTACATATTGAGCATTATATAAATATATTCAATTATGCACGATCATCTTGGGAGCCATTGGTTGAAAGTTGGCCAATAGCAGTTTACATGTCAAAATCCCGACACCCAAAGCCTCAATTATTAGTAGAGGTGATTTCTAGACAGGTAGCTCAAGTGACGCTTACATCCAAAGCAGTAGCATTGCTATCTCAAGTATCCGATTTGATTACTTCCGGAGAAAAATTAAAACCAAGAGGTGAAGATTACCCATACATTATAGTGAATGAGACTGGTTTAGATTTGGAAGTTTGGAATGATGCAAAAGAATCCGAAACCAATGGAGTTCCAATTGTTTTACGTGCTGGTAAAGCTTTAGATGAAAGTAAAGTTGAAATTAGAATTCAATTTAAACCAGTCGCCAAGGGGATGTTTAAAGAAATTCAAAGAAATGAATTAGTTATTAGAGTACAACCAAATGAAGCCATTTATTTAAAAATTAATTCCAAAATCCCTGGAATTTCTACTGAAACTTCATTAACTGATTTAGATTTAACTTATGCTACTCGTTATTCTAAAGAYTTTTGGATTCCTGAAGCTTATGAAGCATTAATTAGAGATTGTTATTTAGGTAATCATTCTAATTTTGTTAGAGATGATGAATTGGATGTTTCTTGGAAATTATTTACTCCATTATTGAAT

>var1.1vag

AATTATTGAARTGGAATGATATTCCATTGGCTCCACCAGACAARATTTTGGGTATTTCTGAAGCTTATAACAATGATTCTAACCCTCAAAAARTCAATTTGGGGGTTGGTGCTTATAGAGATAATTCYGGTAAACCAATTATTTTCCCATCAGTTAAAAAAGCTGAAGAAATTTTATTGGGTAAAGAAACTGAAAAGGAATATACTGCCATTGTTGGTTCCAAAAATTTCCAATCAATTGTGAAAAATTTCATTTTCAACAATTCTAATAAAGATGCCAATGGTAAACAATTAATTGATGATGGTAGAATTGTTACTGCTCAAACCATCTCTGGTACTGGATCACTTAAACCTCTTATGGTGAAATTTTTGCTAAACATAGAGAACCAAATTTGGAAATTATTCGTGAGGTTGTTGATTCCAAACATATTGTTTTTGATGTGTTGGCACAATTCTTAATCAATCCAGACCCATGGGTTGCCATTGCTGCCGCTGAAGTTTATGTCAGACGTTCATACCGTGCTTATGATTTGGGTAMAATTGAATATCATGTTAATGACAGACTTCCTATTGTTGAATGGAAATTCAAGTTGGCTAATATGGGAGCCGCTGGTGTAAACGATGCTCAACAGGCTGCTGCTGCYGGTGGCGATGATTCGACATCTATGAAACATGCAGCTTCTGTGTCTGATTTGACCTTTGTTGTTGATTATTCATRAAAGCGAAATCCCAGGATACACTCTCCCCGATAATCCAAAGTTCACCCTTGGTAATTTGTTTGTAATAATTGGAGTCTTGTTGGTTTGTATTTTAGCTGTTGTCTCTCTTTTGAGAAATATTAGTGAGTCGGCCTTGTTCAAGAAGAATGGGTATGAACCGTTGGATTCGGATCCTAGTGTCATGAACCTAAACTTCGAGCCTACAACATTGTCCTTTGAAGATATTAAATATGAGGTTACTGGTGGTCGACAAATTTTAAATGGAGTCTTTGGGTTTGTAAAACCAAGAGAATGTTTGGCTATAATGGGAGGTTCAGGTGCTGGTAAAACTACATTGTTGGATTTTAAACCTTTGGACCAATTGGCTAAGACTTTGACCACTGTTCCTGAATTGAATGAAATTATTGGGCAAGATTTAGTTGACGAATTTGTCAGTGGTATTAAACTACCAGCAGAAGTTGGAAGTCAAGATGATGTTAACAATAGAAAATTGTTGCAAAAAGTGTTTGGTAAATTAATGAACACTGATGATGACGTTATAAAACAACAAACAGCTAAATTACTTGAAAGAACAGACAGAGAACCTCAAGTGTTCAAGGATATTGATTCTAGATTACCGGAGTTAATACAAAGATTAAACAAACAATTTCCTAATGACATCGGATTATTTTGTGGATGTCTCTTATTGAACCACGTTGATTGAAAAAATTATCAATTTCCGTGTTGGATAAACAGAGATTGACTGAAAAATTCAATAAATTGGATAAATCCATCAAAGATAATTTGAAGGCTAAACAAAAAGAAGAAACCAAAAAAACTTTAGATGTGGTTAATAATTGGTTGAATGATAAAGAAAATGCTTCATCATTTTTGGTTGCTCACGTTCCAATTACTGCTAATGCCAAGGCAATCACTGAAGCCATTAATTTGATTAAAAAGCAAGATAAAACCAAATCAATTTATTTATTGACTGGTGAAACCGATAAAGTTGCTCATGGATGTTATGTTAGTGATGAAGCCATTGCCAAGGGTATTAATGCGAAAATCAAACCTTTTGAAGCACGAGCAATTAACTGGTCCACGGATCTTAATGCTGAGGTACATATTGAGCATTATATAAATATATTCAATTATGCACGATCATCTTGGGAGCCATTGGTTGAAAGTTGGCCAATAGCAGTTTACATGTCAAAATCCCGACACCCAAAGCCTCAATTATTAGTAGAGGTGATTTCTAGACAGGTAGCYCAAGTGACGCTTACATCCAAAGCAGTAGCATTGCTATCTCAAGTATCCGATTTGATTACTTCCRGAGAAAAATTAAAACCAAGAGGTGAAGATTACCCATACGTKATAGTGAATGAGACTGGTTTAGATTTGGAAGTTTGGAATGATGCAAAWGAATYCGAAACCAATGGAGTTCCAATTGTTTTACGTGCTGGTAAAGCTTTAGATGAAAGTAAAGTTGAAATTAGAATTCAATTTAAACCAGTCGCCAAGGGGATGTTTAAAGAAATTCAAAGAAATGAATTAGTTATTAGAGTACAACCAAATGAAGCCATTTATTTAAAAATTAATTCCAAAATCCCTGGAATTTCTACTGAAACTTCATTAACTGATTTAGATTTAACTTATGCTACTCGTTATTCTAAAGAYTTTTGGATTCCTGAAGCTTATGAAGCATTAATTAGAGATTGYTATTTAGGTAATCATTCTAATTTTGTTAGAGATGATGAATTGGATGTTTCTTGGAAATTATTTACTCCATTATTGAAT

>var1.3vag

AATTATTGAAATGGAATGATATTCCATTGGCTCCACCAGACAAGATTTTGGGTATTTCTGAAGCTTATAACAATGATTCTAACCCTCAAAAAGTCAATTTGGGGGTTGGTGCTTATAGAGATAATTCTGGTAAACCAATTATTTTCCCATCAGTTAAAAAAGCTGAAGAAATTTTATTGGGTAAAGAAACTGAAAAGGAATATACTGCCATTGTTGGTTCCAAAAATTTCCAATCAATTGTGAAAAATTTCATTTTCAACAATTCTAATAAAGATGCCAATGGTAAACAATTAATTGATGATGGTAGAATTGTTACTGCTCAAACCATCTCTGGTACTGGATCACTTAAACCTCTTATGGTGAAATTTTTGCTAAACATAGAGAACCAAATTTGGAAATTATTCGTGAGGTTGTTGATTCCAAACATATTGTTTTTGATGTGTTGGCACAATTCTTAATCAATCCAGACCCATGGGTTGCCATTGCTGCCGCTGAAGTTTATGTCAGACGTTCATACCGTGCTTATGATTTGGGTAMAATTGAATATCATGTTAATGACAGACTTCCTATTGTTGAATGGAAATTCAAGTTGGCTAATATGGGAGCCGCTGGTGTAAACGATGCTCAACAGGCTGCTGCTGCYGGTGGCGATGATTCGACATCTATGAAACATGCAGCTTCTGTGTCTGATTTGACCTTTGTTGTTGATTATTCATRAAAGCGAAATCCCAGGATACACTCTCCCCGATAATCCAAAGTTCACCCTTGGTAATTTGTTTGTAATAATTGGAGTCTTGTTGGTTTGTATTTTAGCTGTTGTCTCTCTTTTGAGAAATATTAGTGAGTCGGCCTTGTTCAAGAAGAATGGGTATGAACCGTTGGATTCGGATCCTAGTGTCATGAACCTAAACTTCGAGCCTACAACATTGTCCTTTGAAGATATTAAATATGAGGTTACTGGTGGTCGACAAATTTTAAATGGAGTCTTTGGGTTTGTAAAACCAAGAGAATGTTTGGCTATAATGGGAGGTTCAGGTGCTGGTAAAACTACATTGTTGGATTTTAAACCTTTGGACCAATTGGCTAARACTTTGRCYACTGTTCCTGAATTGAATGAAATTATTGGKCAAGAWTTAGTTGACGAATTTRTCAGTGGTATTAAACTACCAGCAGAAGTTGGAAGTCAAGATGATGTTAACAATAGAAAATTGTTGCAAAAAGTGTTTGGTAAATTAATGAACACTGATGATGACGTTATAAAACAACAAACAGCTAAATTACTTGAAAGAACAGACAGAGAACCTCAAGTGTTCAAGGATATTGATTCTAGATTACCGGAGTTAATACAAAGATTAAACAAACAATTTCCTAATGACATCGGATTATTTTGTGGATGTCTCTTATTGAACCACGTTGATTGAAAAAATTATCAATTTCCGTGTTGGATAAACAGAGATTGACTGAAAAATTCAATAAATTGGATAAATCCATCAAAGATAATTTGAAGGCTAAACAAAAAGAAGAAACCAAAAAAACTTTAGATGTGGTTAATAATTGGTTGAATGATAAAGAAAATGCTTCATCATTTTTGGTTGCTCACGTTCCAATTACTGCTAATGCCAAGGCAATCACTGAAGCCATTAATTTGATTAAAAAGCAAGATAAAACCAAATCAATTTATTTATTGACTGGTGAAACCGATAAAGTTGCTCATGGATGTTATGTTAGTGATGAAGCCATTGCCAAGGGTATTAATGCGAAAATCAAACCTTTTGAAGCACGAGCAATTAACTGGTCCACGGATCTTAATGCTGAGGTACATATTGAGCATTATATAAATATATTCAATTATGCACGATCATCTTGGGAGCCATTGGTTGAAAGTTGGCCAATAGCAGTTTACATGTCAAAATCCCGACACCCAAAGCCTCAATTATTAGTAGAGGTGATTTCTAGACAGGTAGCYCAAGTGACGCTTACATCCAAAGCAGTAGCATTGCTATCTCAAGTATCCGATTTGATTACTTCCRGAGAAAAATTAAAACCAAGAGGTGAAGATTACCCATACGTKATAGTGAATGAGACTGGTTTAGATTTGGAAGTTTGGAATGATGCAAAWGAATYCGAAACCAATGGAGTTCCAATTGTTTTACGTGCTGGTAAAGCTTTAGATGAAAGTAAAGTTGAAATTAGAATTCAATTTAAACCAGTCGCCAAGGGGATGTTTAAAGAAATTCAAAGAAATGAATTAGTTATTAGAGTACAACCAAATGAAGCCATTTATTTAAAAATTAATTCCAAAATCCCTGGAATTTCTACTGAAACTTCATTAACTGATTTAGATTTAACTTATGCTACTCGTTATTCTAAAGAYTTTTGGATTCCTGAAGCTTATGAAGCATTAATTAGAGATTGYTATTTAGGTAATCATTCTAATTTTGTTAGAGATGATGAATTGGATGTTTCTTGGAAATTATTTACTCCATTATTGAAT

>var1.5vag

AATTATTGAAATGGAATGATATTCCATTGGCTCCACCAGACAAAATTTTGGGTATTTCTGAAGCTTATAACAACGATTCTAACCCTCAAAAAATCAATTTGGGGGTTGGTGCTTATAGAGATAATTCCGGTAAACCAATTATTTTCCCATCAGTTAAAAAAGCTGAAGAAATTTTATTGGGTAAAGAAACTGAAAAGGAATATACTGCCATTGTTGGTTCCAAAAATTTCCAATCAATTGTGAAAAATTTCATTTTCAACAATTCTAATAAAGATGCCAATGGTAAACAATTAATTGATGATGGTAGAATTGTTACTGCTCAAACCATCTCTGGTACTGGATCACTTAAACCTCTTATGGTGAAATTTTTGCTAAACATAGAGAACCAAATTTGGAAATTATTCGTGAGGTTGTTGATTCCAAACATATTGTTTTTGATGTGTTGGCACAATTCTTAATCAATCCAGACCCATGGGTTGCCATTGCTGCCGCTGAAGTTTATGTCAGACGTTCATACCGTGCTTATGATTTGGGTACAATTGAATATCATGTTAATGACAGACTTCCTATTGTTGAATGGAAATTCAAGTTGGCTAATATGGGAGCCGCTGGTGTAAACGATGCTCAACAGGCTGCTGCTGCTGGTGGCGATGATTCGACATCTATGAAACATGCAGCTTCTGTGTCTGATTTGACCTTTGTTGTTGATTATTCATAAAAGCGAAATCCCAGGATACACTCTCCCCGATAATCCAAAGTTCACCCTTGGTAATTTGTTTGTAATAATTGGAGTCTTGTTGGTTTGTRTTTTAGCTGTTGTCTCTCTTTTGAGAAATATTAGTGAGTCGGCCTTGTTCAAGAAGAATGGGTATGAACCGTTGGATTCGGATCCTAGTGTCATGAACCTAAACTTCGAGCCTACAACATTGTCCTTTGAAGATATTAAATATGAGGTTACTGGTGGTCGACAAATTTTAAATGGAGTCTTTGGGTTTGTAAAACCAAGAGAATGTTTGGCTATAATGGGAGGTTCAGGTGCTGGTAAAACTACATTGTTGGATTTTAAACCTTTGGACCAATTGGCTAAGACTTTGACCACTGTTCCTGAATTGAATGAAATTATTGGGCAAGATTTAGTTGACGAATTTGTCAGTGGTATTAAACTACCAGCAGAAGTTGGAAGTCAAGATGATGTTAACAATAGAAAATTGTTGCAAAAAGTGTTTGGTAAATTAATGAACACTGATGATGACGTTATAAAACAACAAACAGCTAAATTACTTGAAAGAACAGACAGAGAACCTCAAGTGTTCAAGGATATTGATTCTAGATTACCGGAGTTAATACAAAGATTAAACAAACAATTTCCTAATGACATCGGATTATTTTGTGGATGTCTCTTATTGAACCACGTTGMTTGAAAAAATTATCAATTTCCGTGTTGGATAAACAAAGATTGACTGAAAAATTCAATAAATTGGATAAATCCATYAAAGATAATTTGAAGGCTAAACAAAAAGAAGAAACCAAAAARACTTTAGATGTGGTTAAYAATTGGTTGAATGATAAAGAAAATKCTTCATCATTTTTGGTTGCTCACGTTCCAATTACTGCTAATGCCAAGGCAATCACTGAAGCCATTAATTTGATTAAAAAGCAAGATAAAACCAAATCAATTTATTTATTGACTGGTGAAACCGATAAAGTTGCTCATGGATGTTATGTTAGTGATGAAGCCATTGTCAAGGGTATTAATGCGAAAATCAAACCTTTTGAAGCACGAGCAATTAACTGGTCCACGGATCTTAATGCTGAGGTACATATTGAGCATTATATAAATATATTCAATTATGCACGATCATCTTGGGAGCCATTGGTTGAAAGTTGGCCAATAGCAGTTTACATGTCAAAATCCCGACACCCAAAGCCTCAATTATTAGTAGAGGTGATTTCTAGACAGGTAGCTCAAGTGACGCTTACATCCAAAGCRGTAGCATTGCTATCTCAAGTATCCGATTTGATTACTTCCRGAGAAAAATTAAAACCAAGAGGTGAAGATTACCCATACGTKATAGTGAATGAGACTGGTTTAGATTTGGAAGTTTGGAATGATGCAAATGAATYCGAAACCAATGGAGTTCCAATTGTTTTACGTGCTGGTAAAGCTTTAGATGAAAGTAAAGTTGAAATTAGAATTCAATTTAAACCAGTCGCCAAGGGGATGTTTAAAGAAATTCAAAGAAATGAATTAGTTATTAGAGTACAACCAAATGAAGCCATTTATTTAAAAATTAATTCCAAAATCCCTGGAATTTCTACTGAAACTTCATTAACTGATTTAGATTTAACTTATGCTACTCGTTATTCTAAAGACTTTTGGATTCCTGAAGCTTATGAAGCATTAATTAGAGATTGYTATTTAGGTAATCATTCTAATTTTGTTAGAGATGATGAATTGGATGTTTCTTGGAAATTATTTACTCCATTATTGAAT

>var1.7vul

AATTATTGAAATGGAATGATATTCCATTGGCTCCACCAGACAAAATTTTGGGTATTTCTGAAGCTTATAACAAYGATTCTAACCCTCAAAAAATCAATTTGGGGGTTGGTGCTTATAGAGATAATTCYGGTAAACCAATTATTTTCCCATCAGTTAAAAAAGCTGAAGAAATTTTATTGGGTAAAGAAACTGAAAAGGAATATACTGCCATTGTTGGTTCCAAAAATTTCCAATCAATTGTGAAAAATTTYATTTTCAACAATTCTAATAAAGATGCCAATGGTAAACAATTAATTGATGATGGTAGAATTGTTACTGCTCAAACCATCTCTGGTACTGGATCACTTAAACCTCTTATGGTGAAATTTTTGCTAAACATAGAGAACCAAATTTGGAAATTATTCGTGAGGTTGTTGATTCCAAACATATTGTTTTTGATGTGTTGGCACAATTCTTAATCAATCCAGACCCATGGGTTGCCATTGCTGCCGCTGAAGTTTATGTCAGACGTTCATACCGTGCTTATGATTTGGGTAMAATTGAATATCATGTTAATGACAGACTTCCTATTGTTGAATGGAAATTCAAGTTGGCTAATATGGGAGCYGCTGGTGTAAACGATGCTCAACAGGCTGCTGCTGCYGGTGGCGATGATTCGACATCTATGAAACATGCAGCTTCTGTGTCTGATTTGACCTTTGTTGTTGATTATTCATAAAAGCGAAATCCCAGGATACACTCTCCCCGATAATCCAAAGTTCACCCTTGGTAATTTGTTTGTAATAATTGGAGTCTTGTTGGTTTGTATTTTAGCTGTTGTCTCTCTTTTGAGAAATATTAGTGAGTCGGCCTTGTTCAAGAAGAATGGGTATGAACCGTTGGATTCGGATCCTAGTGTCATGAACCTAAACTTCGAGCCTACAACATTGTCCTTTGAAGATATTAAATATGAGGTTACTGGTGGTCGACAAATTTTAAATGGAGTCTTTGGGTTTGTAAAACCAAGAGAATGTTTGGCTATAATGGGAGGTTCRGGTGCTGGTAAAACTACATTGTTGGATTTTAAACCTTTGGACCAATTGGCTAARACTTTGRCYACTGTTCCTGAATTGAATGAAATTATTGGKCAAGAWTTAGTTGACGAATTTRTCAGTGGTATTAAACTACCAGCAGAAGTTGGAAGTCAAGATGATGTTAACAATAGAAAATTGTTGCAAAAAGTGTTTGGTAAATTAATGAACACTGATGATGACGTTATAAAACAACAAACAGCTAAATTACTTGAAAGAACAGACAGAGAACCTCAAGTGTTCAAGGATATTGATTCTAGATTACCGGAGTTAATACAAAGATTAAACAAACAATTTCCTAATGACATCGGATTATTTTGTGGATGTCTCTTATTGAACCACGTTGMTTGAAAAAATTATCAATTTCCGTGTTGGATAAACAAAGATTGACTGAAAAATTCAATAAATTGGATAAATCCATTAAAGATAATTTGAAGGCTAAACAAAAAGAAGAAACCAAAAAGACTTTAGATGTGGTTAACAATTGGTTGAATGATAAAGAAAATGCTTCATCATTTTTGGTTGCTCACGTTCCAATTACTGCTAATGCCAAGGCAATCACTGAAGCCATTAATTTGATTAAAAAGCAAGATAAAACCAAATCAATTTATTTATTGACTGGTGAAACCGATAAAGTTGCTCATGGATGTTATGTTAGTGATGAAGCCATTGYCAAGGGTATTAATGCGAAAATCAAACCTTTTGAAGCACGAGCAATTAACTGGTCCACGGATCTTAATGCTGAGGTACATATTGAGCATTATATAAATATATTCAATTATGCACGATCATCTTGGGAGCCATTGGTTGAAAGTTGGCCAATAGCAGTTTACATGTCAAAATCCCGACACCCAAAGCCTCAATTATTAGTAGAGGTGATTTCTAGACAGGTAGCTCAAGTGACGCTTACATCCAAAGCAGTAGCATTGCTATCTCAAGTATCCGATTTGATTACTTCCRGAGAAAAATTAAAACCAAGAGGTGAAGATTACCCATACGTKATAGTGAATGAGACTGGTTTAGATTTGGAAGTTTGGAATGATGCAAAWGAATCCGAAACCAATGGAGTTCCAATTGTTTTACGTGCTGGTAAAGCTTTAGATGAAAGTAAAGTTGAAATTAGAATTCAATTTAAACCAGTCGCCAAGGGGATGTTTAAAGAAATTCAAAGAAATGAATTAGTTATTAGAGTACAACCAAATGAAGCCATTTATTTAAAAATTAATTCCAAAATCCCTGGAATTTCTACTGAAACTTCATTAACTGATTTAGATTTAACTTATGCTACTCGTTATTCTAAAGATTTTTGGATTCCTGAAGCTTATGAAGCATTAATTAGAGATTGTTATTTAGGTAATCATTCTAATTTTGTTAGAGATGATGAATTGGATGTTTCTTGGAAATTATTTACTCCATTATTGAAT

>var1.10vag

AATTATTGAARTGGAATGATATTCCATTGGCTCCACCAGACAARATTTTGGGTATTTCTGAAGCTTATAACAATGATTCTAACCCTCAAAAARTCAATTTGGGGGTTGGTGCTTATAGAGATAATTCYGGTAAACCAATTATTTTCCCATCAGTTAAAAAAGCTGAAGAAATTTTATTGGGTAAAGAAACTGAAAAGGAATATACTGCCATTGTTGGTTCCAAAAATTTCCAATCAATTGTGAAAAATTTCATTTTCAACAATTCTAATAAAGATGCCAATGGTAAACAATTAATTGATGATGGTAGAATTGTTACTGCTCAAACCATCTCTGGTACTGGATCACTTAAACCTCTTATGGTGAAATTTTTGCTAAACATAGAGAACCAAATTTGGAAATTATTCGTGAGGTTGTTGATTCCAAACATATTGTTTTTGATGTGTTGGCACAATTCTTAATCAATCCAGACCCATGGGTTGCCATTGCTGCCGCTGAAGTTTATGTCAGACGTTCATACCGTGCTTATGATTTGGGTAMAATTGAATATCATGTTAATGACAGACTTCCTATTGTTGAATGGAAATTCAAGTTGGCTAATATGGGAGCCGCTGGTGTAAACGATGCTCAACAGGCTGCTGCTGCYGGTGGCGATGATTCGACATCTATGAAACATGCAGCTTCTGTGTCTGATTTGACCTTTGTTGTTGATTATTCATRAAAGCGAAATCCCAGGATACACTCTCCCCGATAATCCAAAGTTCACCCTTGGTAATTTGTTTGTAATAATTGGAGTCTTGTTGGTTTGTATTTTAGCTGTTGTCTCTCTTTTGAGAAATATTAGTGAGTCGGCCTTGTTCAAGAAGAATGGGTATGAACCGTTGGATTCGGATCCTAGTGTCATGAACCTAAACTTCGAGCCTACAACATTGTCCTTTGAAGATATTAAATATGAGGTTACTGGTGGTCGACAAATTTTAAATGGAGTCTTTGGGTTTGTAAAACCAAGAGAATGTTTGGCTATAATGGGAGGTTCAGGTGCTGGTAAAACTACATTGTTGGATTTTAAACCTTTGGACCAATTGGCTAARACTTTGRCYACTGTTCCTGAATTGAATGAAATTATTGGKCAAGAWTTAGTTGACGAATTTRTCAGTGGTATTAAACTACCAGCAGAAGTTGGAAGTCAAGATGATGTTAACAATAGAAAATTGTTGCAAAAAGTGTTTGGTAAATTAATGAACACTGATGATGACGTTATAAAACAACAAACAGCTAAATTACTTGAAAGAACAGACAGAGAACCTCAAGTGTTCAAGGATATTGATTCTAGATTACCGGAGTTAATACAAAGATTAAACAAACAATTTCCTAATGACATCGGATTATTTTGTGGATGTCTCTTATTGAACCACGTTGATTGAAAAAATTATCAATTTCCGTGTTGGATAAACAGAGATTGACTGAAAAATTCAATAAATTGGATAAATCCATCAAAGATAATTTGAAGGCTAAACAAAAAGAAGAAACCAAAAAAACTTTAGATGTGGTTAATAATTGGTTGAATGATAAAGAAAATGCTTCATCATTTTTGGTTGCTCACGTTCCAATTACTGCTAATGCCAAGGCAATCACTGAAGCCATTAATTTGATTAAAAAGCAAGATAAAACCAAATCAATTTATTTATTGACTGGTGAAACCGATAAAGTTGCTCATGGATGTTATGTTAGTGATGAAGCCATTGCCAAGGGTATTAATGCGAAAATCAAACCTTTTGAAGCACGAGCAATTAACTGGTCCACGGATCTTAATGCTGAGGTACATATTGAGCATTATATAAATATATTCAATTATGCACGATCATCTTGGGAGCCATTGGTTGAAAGTTGGCCAATAGCAGTTTACATGTCAAAATCCCGACACCCAAAGCCTCAATTATTAGTAGAGGTGATTTCTAGACAGGTAGCYCAAGTGACGCTTACATCCAAAGCAGTAGCATTGCTATCTCAAGTATCCGATTTGATTACTTCCRGAGAAAAATTAAAACCAAGAGGTGAAGATTACCCATACGTKATAGTGAATGAGACTGGTTTAGATTTGGAAGTTTGGAATGATGCAAAWGAATYCGAAACCAATGGAGTTCCAATTGTTTTACGTGCTGGTAAAGCTTTAGATGAAAGTAAAGTTGAAATTAGAATTCAATTTAAACCAGTCGCCAAGGGGATGTTTAAAGAAATTCAAAGAAATGAATTAGTTATTAGAGTACAACCAAATGAAGCCATTTATTTAAAAATTAATTCCAAAATCCCTGGAATTTCTACTGAAACTTCATTAACTGATTTAGATTTAACTTATGCTACTCGTTATTCTAAAGACTTTTGGATTCCTGAAGCTTATGAAGCATTAATTAGAGATTGCTATTTAGGTAATCATTCTAATTTTGTTAGAGATGATGAATTGGATGTTTCTTGGAAATTATTTACTCCATTATTGAAT

>var1.10vag:IND:17:7.1

AATTATTGAARTGGAATGATATTCCATTGGCTCCACCAGACAARATTTTGGGTATTTCTGAAGCTTATAACAATGATTCTAACCCTCAAAAARTCAATTTGGGGGTTGGTGCTTATAGAGATAATTCYGGTAAACCAATTATTTTCCCATCAGTTAAAAAAGCTGAAGAAATTTTATTGGGTAAAGAAACTGAAAAGGAATATACTGCCATTGTTGGTTCCAAAAATTTCCAATCAATTGTGAAAAATTTCATTTTCAACAATTCTAATAAAGATGCCAATGGTAAACAATTAATTGATGATGGTAGAATTGTTACTGCTCAAACCATCTCTGGTACTGGATCACTTAAACCTCTTATGGTGAAATTTTTGCTAAACATAGAGAACCAAATTTGGAAATTATTCGTGAGGTTGTTGATTCCAAACATATTGTTTTTGATGTGTTGGCACAATTCTTAATCAATCCAGACCCATGGGTTGCCATTGCTGCCGCTGAAGTTTATGTCAGACGTTCATACCGTGCTTATGATTTGGGTAMAATTGAATATCATGTTAATGACAGACTTCCTATTGTTGAATGGAAATTCAAGTTGGCTAATATGGGAGCCGCTGGTGTAAACGATGCTCAACAGGCTGCTGCTGCYGGTGGCGATGATTCGACATCTATGAAACATGCAGCTTCTGTGTCTGATTTGACCTTTGTTGTTGATTATTCATRAAAGCGAAATCCCAGGATACACTCTCCCCGATAATCCAAAGTTCACCCTTGGTAATTTGTTTGTAATAATTGGAGTCTTGTTGGTTTGTATTTTAGCTGTTGTCTCTCTTTTGAGAAATATTAGTGAGTCGGCCTTGTTCAAGAAGAATGGGTATGAACCGTTGGATTCGGATCCTAGTGTCATGAACCTAAACTTCGAGCCTACAACATTGTCCTTTGAAGATATTAAATATGAGGTTACTGGTGGTCGACAAATTTTAAATGGAGTCTTTGGGTTTGTAAAACCAAGAGAATGTTTGGCTATAATGGGAGGTTCAGGTGCTGGTAAAACTACATTGTTGGATTTTAAACCTTTGGACCAATTGGCTAARACTTTGRCYACTGTTCCTGAATTGAATGAAATTATTGGKCAAGAWTTAGTTGACGAATTTRTCAGTGGTATTAAACTACCAGCAGAAGTTGGAAGTCAAGATGATGTTAACAATAGAAAATTGTTGCAAAAAGTGTTTGGTAAATTAATGAACACTGATGATGACGTTATAAAACAACAAACAGCTAAATTACTTGAAAGAACAGACAGAGAACCTCAAGTGTTCAAGGATATTGATTCTAGATTACCGGAGTTAATACAAAGATTAAACAAACAATTTCCTAATGACATCGGATTATTTTGTGGATGTCTCTTATTGAACCACGTTGATTGAAAAAATTATCAATTTCCGTGTTGGATAAACAGAGATTGACTGAAAAATTCAATAAATTGGATAAATCCATCAAAGATAATTTGAAGGCTAAACAAAAAGAAGAAACCAAAAAAACTTTAGATGTGGTTAATAATTGGTTGAATGATAAAGAAAATGCTTCATCATTTTTGGTTGCTCACGTTCCAATTACTGCTAATGCCAAGGCAATCACTGAAGCCATTAATTTGATTAAAAAGCAAGATAAAACCAAATCAATTTATTTATTGACTGGTGAAACCGATAAAGTTGCTCATGGATGTTATGTTAGTGATGAAGCCATTGCCAAGGGTATTAATGCGAAAATCAAACCTTTTGAAGCACGAGCAATTAACTGGTCCACGGATCTTAATGCTGAGGTACATATTGAGCATTATATAAATATATTCAATTATGCACGATCATCTTGGGAGCCATTGGTTGAAAGTTGGCCAATAGCAGTTTACATGTCAAAATCCCGACACCCAAAGCCTCAATTATTAGTAGAGGTGATTTCTAGACAGGTAGCYCAAGTGACGCTTACATCCAAAGCAGTAGCATTGCTATCTCAAGTATCCGATTTGATTACTTCCRGAGAAAAATTAAAACCAAGAGGTGAAGATTACCCATACGTKATAGTGAATGAGACTGGTTTAGATTTGGAAGTTTGGAATGATGCAAAWGAATYCGAAACCAATGGAGTTCCAATTGTTTTACGTGCTGGTAAAGCTTTAGATGAAAGTAAAGTTGAAATTAGAATTCAATTTAAACCAGTCGCCAAGGGGATGTTTAAAGAAATTCAAAGAAATGAATTAGTTATTAGAGTACAACCAAATGAAGCCATTTATTTAAAAATTAATTCCAAAATCCCTGGAATTTCTACTGAAACTTCATTAACTGATTTAGATTTAACTTATGCTACTCGTTATTCTAAAGACTTTTGGATTCCTGAAGCTTATGAAGCATTAATTAGAGATTGCTATTTAGGTAATCATTCTAATTTTGTTAGAGATGATGAATTGGATGTTTCTTGGAAATTATTTACTCCATTATTGAAT

>var1.10vag:IND:26:8.6

AATTATTGAARTGGAATGATATTCCATTGGCTCCACCAGACAARATTTTGGGTATTTCTGAAGCTTATAACAATGATTCTAACCCTCAAAAARTCAATTTGGGGGTTGGTGCTTATAGAGATAATTCYGGTAAACCAATTATTTTCCCATCAGTTAAAAAAGCTGAAGAAATTTTATTGGGTAAAGAAACTGAAAAGGAATATACTGCCATTGTTGGTTCCAAAAATTTCCAATCAATTGTGAAAAATTTCATTTTCAACAATTCTAATAAAGATGCCAATGGTAAACAATTAATTGATGATGGTAGAATTGTTACTGCTCAAACCATCTCTGGTACTGGATCACTTAAACCTCTTATGGTGAAATTTTTGCTAAACATAGAGAACCAAATTTGGAAATTATTCGTGAGGTTGTTGATTCCAAACATATTGTTTTTGATGTGTTGGCACAATTCTTAATCAATCCAGACCCATGGGTTGCCATTGCTGCCGCTGAAGTTTATGTCAGACGTTCATACCGTGCTTATGATTTGGGTAMAATTGAATATCATGTTAATGACAGACTTCCTATTGTTGAATGGAAATTCAAGTTGGCTAATATGGGAGCCGCTGGTGTAAACGATGCTCAACAGGCTGCTGCTGCYGGTGGCGATGATTCGACATCTATGAAACATGCAGCTTCTGTGTCTGATTTGACCTTTGTTGTTGATTATTCATRAAAGCGAAATCCCAGGATACACTCTCCCCGATAATCCAAAGTTCACCCTTGGTAATTTGTTTGTAATAATTGGAGTCTTGTTGGTTTGTATTTTAGCTGTTGTCTCTCTTTTGAGAAATATTAGTGAGTCGGCCTTGTTCAAGAAGAATGGGTATGAACCGTTGGATTCGGATCCTAGTGTCATGAACCTAAACTTCGAGCCTACAACATTGTCCTTTGAAGATATTAAATATGAGGTTACTGGTGGTCGACAAATTTTAAATGGAGTCTTTGGGTTTGTAAAACCAAGAGAATGTTTGGCTATAATGGGAGGTTCAGGTGCTGGTAAAACTACATTGTTGGATTTTAAACCTTTGGACCAATTGGCTAARACTTTGRCYACTGTTCCTGAATTGAATGAAATTATTGGKCAAGAWTTAGTTGACGAATTTRTCAGTGGTATTAAACTACCAGCAGAAGTTGGAAGTCAAGATGATGTTAACAATAGAAAATTGTTGCAAAAAGTGTTTGGTAAATTAATGAACACTGATGATGACGTTATAAAACAACAAACAGCTAAATTACTTGAAAGAACAGACAGAGAACCTCAAGTGTTCAAGGATATTGATTCTAGATTACCGGAGTTAATACAAAGATTAAACAAACAATTTCCTAATGACATCGGATTATTTTGTGGATGTCTCTTATTGAACCACGTTGATTGAAAAAATTATCAATTTCCGTGTTGGATAAACAGAGATTGACTGAAAAATTCAATAAATTGGATAAATCCATCAAAGATAATTTGAAGGCTAAACAAAAAGAAGAAACCAAAAAAACTTTAGATGTGGTTAATAATTGGTTGAATGATAAAGAAAATGCTTCATCATTTTTGGTTGCTCACGTTCCAATTACTGCTAATGCCAAGGCAATCACTGAAGCCATTAATTTGATTAAAAAGCAAGATAAAACCAAATCAATTTATTTATTGACTGGTGAAACCGATAAAGTTGCTCATGGATGTTATGTTAGTGATGAAGCCATTGCCAAGGGTATTAATGCGAAAATCAAACCTTTTGAAGCACGAGCAATTAACTGGTCCACGGATCTTAATGCTGAGGTACATATTGAGCATTATATAAATATATTCAATTATGCACGATCATCTTGGGAGCCATTGGTTGAAAGTTGGCCAATAGCAGTTTACATGTCAAAATCCCGACACCCAAAGCCTCAATTATTAGTAGAGGTGATTTCTAGACAGGTAGCYCAAGTGACGCTTACATCCAAAGCAGTAGCATTGCTATCTCAAGTATCCGATTTGATTACTTCCRGAGAAAAATTAAAACCAAGAGGTGAAGATTACCCATACGTKATAGTGAATGAGACTGGTTTAGATTTGGAAGTTTGGAATGATGCAAAWGAATYCGAAACCAATGGAGTTCCAATTGTTTTACGTGCTGGTAAAGCTTTAGATGAAAGTAAAGTTGAAATTAGAATTCAATTTAAACCAGTCGCCAAGGGGATGTTTAAAGAAATTCAAAGAAATGAATTAGTTATTAGAGTACAACCAAATGAAGCCATTTATTTAAAAATTAATTCCAAAATCCCTGGAATTTCTACTGAAACTTCATTAACTGATTTAGATTTAACTTATGCTACTCGTTATTCTAAAGACTTTTGGATTCCTGAAGCTTATGAAGCATTAATTAGAGATTGCTATTTAGGTAATCATTCTAATTTTGTTAGAGATGATGAATTGGATGTTTCTTGGAAATTATTTACTCCATTATTGAAT

>var1.10vag:IND:29:8.3

AATTATTGAARTGGAATGATATTCCATTGGCTCCACCAGACAARATTTTGGGTATTTCTGAAGCTTATAACAATGATTCTAACCCTCAAAAARTCAATTTGGGGGTTGGTGCTTATAGAGATAATTCYGGTAAACCAATTATTTTCCCATCAGTTAAAAAAGCTGAAGAAATTTTATTGGGTAAAGAAACTGAAAAGGAATATACTGCCATTGTTGGTTCCAAAAATTTCCAATCAATTGTGAAAAATTTCATTTTCAACAATTCTAATAAAGATGCCAATGGTAAACAATTAATTGATGATGGTAGAATTGTTACTGCTCAAACCATCTCTGGTACTGGATCACTTAAACCTCTTATGGTGAAATTTTTGCTAAACATAGAGAACCAAATTTGGAAATTATTCGTGAGGTTGTTGATTCCAAACATATTGTTTTTGATGTGTTGGCACAATTCTTAATCAATCCAGACCCATGGGTTGCCATTGCTGCCGCTGAAGTTTATGTCAGACGTTCATACCGTGCTTATGATTTGGGTAMAATTGAATATCATGTTAATGACAGACTTCCTATTGTTGAATGGAAATTCAAGTTGGCTAATATGGGAGCCGCTGGTGTAAACGATGCTCAACAGGCTGCTGCTGCYGGTGGCGATGATTCGACATCTATGAAACATGCAGCTTCTGTGTCTGATTTGACCTTTGTTGTTGATTATTCATRAAAGCGAAATCCCAGGATACACTCTCCCCGATAATCCAAAGTTCACCCTTGGTAATTTGTTTGTAATAATTGGAGTCTTGTTGGTTTGTATTTTAGCTGTTGTCTCTCTTTTGAGAAATATTAGTGAGTCGGCCTTGTTCAAGAAGAATGGGTATGAACCGTTGGATTCGGATCCTAGTGTCATGAACCTAAACTTCGAGCCTACAACATTGTCCTTTGAAGATATTAAATATGAGGTTACTGGTGGTCGACAAATTTTAAATGGAGTCTTTGGGTTTGTAAAACCAAGAGAATGTTTGGCTATAATGGGAGGTTCAGGTGCTGGTAAAACTACATTGTTGGATTTTAAACCTTTGGACCAATTGGCTAARACTTTGRCYACTGTTCCTGAATTGAATGAAATTATTGGKCAAGAWTTAGTTGACGAATTTRTCAGTGGTATTAAACTACCAGCAGAAGTTGGAAGTCAAGATGATGTTAACAATAGAAAATTGTTGCAAAAAGTGTTTGGTAAATTAATGAACACTGATGATGACGTTATAAAACAACAAACAGCTAAATTACTTGAAAGAACAGACAGAGAACCTCAAGTGTTCAAGGATATTGATTCTAGATTACCGGAGTTAATACAAAGATTAAACAAACAATTTCCTAATGACATCGGATTATTTTGTGGATGTCTCTTATTGAACCACGTTGATTGAAAAAATTATCAATTTCCGTGTTGGATAAACAGAGATTGACTGAAAAATTCAATAAATTGGATAAATCCATCAAAGATAATTTGAAGGCTAAACAAAAAGAAGAAACCAAAAAAACTTTAGATGTGGTTAATAATTGGTTGAATGATAAAGAAAATGCTTCATCATTTTTGGTTGCTCACGTTCCAATTACTGCTAATGCCAAGGCAATCACTGAAGCCATTAATTTGATTAAAAAGCAAGATAAAACCAAATCAATTTATTTATTGACTGGTGAAACCGATAAAGTTGCTCATGGATGTTATGTTAGTGATGAAGCCATTGCCAAGGGTATTAATGCGAAAATCAAACCTTTTGAAGCACGAGCAATTAACTGGTCCACGGATCTTAATGCTGAGGTACATATTGAGCATTATATAAATATATTCAATTATGCACGATCATCTTGGGAGCCATTGGTTGAAAGTTGGCCAATAGCAGTTTACATGTCAAAATCCCGACACCCAAAGCCTCAATTATTAGTAGAGGTGATTTCTAGACAGGTAGCYCAAGTGACGCTTACATCCAAAGCAGTAGCATTGCTATCTCAAGTATCCGATTTGATTACTTCCRGAGAAAAATTAAAACCAAGAGGTGAAGATTACCCATACGTKATAGTGAATGAGACTGGTTTAGATTTGGAAGTTTGGAATGATGCAAAWGAATYCGAAACCAATGGAGTTCCAATTGTTTTACGTGCTGGTAAAGCTTTAGATGAAAGTAAAGTTGAAATTAGAATTCAATTTAAACCAGTCGCCAAGGGGATGTTTAAAGAAATTCAAAGAAATGAATTAGTTATTAGAGTACAACCAAATGAAGCCATTTATTTAAAAATTAATTCCAAAATCCCTGGAATTTCTACTGAAACTTCATTAACTGATTTAGATTTAACTTATGCTACTCGTTATTCTAAAGACTTTTGGATTCCTGAAGCTTATGAAGCATTAATTAGAGATTGCTATTTAGGTAATCATTCTAATTTTGTTAGAGATGATGAATTGGATGTTTCTTGGAAATTATTTACTCCATTATTGAAT

>var1.10vag:IND:55:6.0

AATTATTGAARTGGAATGATATTCCATTGGCTCCACCAGACAARATTTTGGGTATTTCTGAAGCTTATAACAATGATTCTAACCCTCAAAAARTCAATTTGGGGGTTGGTGCTTATAGAGATAATTCYGGTAAACCAATTATTTTCCCATCAGTTAAAAAAGCTGAAGAAATTTTATTGGGTAAAGAAACTGAAAAGGAATATACTGCCATTGTTGGTTCCAAAAATTTCCAATCAATTGTGAAAAATTTCATTTTCAACAATTCTAATAAAGATGCCAATGGTAAACAATTAATTGATGATGGTAGAATTGTTACTGCTCAAACCATCTCTGGTACTGGATCACTTAAACCTCTTATGGTGAAATTTTTGCTAAACATAGAGAACCAAATTTGGAAATTATTCGTGAGGTTGTTGATTCCAAACATATTGTTTTTGATGTGTTGGCACAATTCTTAATCAATCCAGACCCATGGGTTGCCATTGCTGCCGCTGAAGTTTATGTCAGACGTTCATACCGTGCTTATGATTTGGGTAMAATTGAATATCATGTTAATGACAGACTTCCTATTGTTGAATGGAAATTCAAGTTGGCTAATATGGGAGCCGCTGGTGTAAACGATGCTCAACAGGCTGCTGCTGCYGGTGGCGATGATTCGACATCTATGAAACATGCAGCTTCTGTGTCTGATTTGACCTTTGTTGTTGATTATTCATRAAAGCGAAATCCCAGGATACACTCTCCCCGATAATCCAAAGTTCACCCTTGGTAATTTGTTTGTAATAATTGGAGTCTTGTTGGTTTGTATTTTAGCTGTTGTCTCTCTTTTGAGAAATATTAGTGAGTCGGCCTTGTTCAAGAAGAATGGGTATGAACCGTTGGATTCGGATCCTAGTGTCATGAACCTAAACTTCGAGCCTACAACATTGTCCTTTGAAGATATTAAATATGAGGTTACTGGTGGTCGACAAATTTTAAATGGAGTCTTTGGGTTTGTAAAACCAAGAGAATGTTTGGCTATAATGGGAGGTTCAGGTGCTGGTAAAACTACATTGTTGGATTTTAAACCTTTGGACCAATTGGCTAARACTTTGRCYACTGTTCCTGAATTGAATGAAATTATTGGKCAAGAWTTAGTTGACGAATTTRTCAGTGGTATTAAACTACCAGCAGAAGTTGGAAGTCAAGATGATGTTAACAATAGAAAATTGTTGCAAAAAGTGTTTGGTAAATTAATGAACACTGATGATGACGTTATAAAACAACAAACAGCTAAATTACTTGAAAGAACAGACAGAGAACCTCAAGTGTTCAAGGATATTGATTCTAGATTACCGGAGTTAATACAAAGATTAAACAAACAATTTCCTAATGACATCGGATTATTTTGTGGATGTCTCTTATTGAACCACGTTGATTGAAAAAATTATCAATTTCCGTGTTGGATAAACAGAGATTGACTGAAAAATTCAATAAATTGGATAAATCCATCAAAGATAATTTGAAGGCTAAACAAAAAGAAGAAACCAAAAAAACTTTAGATGTGGTTAATAATTGGTTGAATGATAAAGAAAATGCTTCATCATTTTTGGTTGCTCACGTTCCAATTACTGCTAATGCCAAGGCAATCACTGAAGCCATTAATTTGATTAAAAAGCAAGATAAAACCAAATCAATTTATTTATTGACTGGTGAAACCGATAAAGTTGCTCATGGATGTTATGTTAGTGATGAAGCCATTGCCAAGGGTATTAATGCGAAAATCAAACCTTTTGAAGCACGAGCAATTAACTGGTCCACGGATCTTAATGCTGAGGTACATATTGAGCATTATATAAATATATTCAATTATGCACGATCATCTTGGGAGCCATTGGTTGAAAGTTGGCCAATAGCAGTTTACATGTCAAAATCCCGACACCCAAAGCCTCAATTATTAGTAGAGGTGATTTCTAGACAGGTAGCYCAAGTGACGCTTACATCCAAAGCAGTAGCATTGCTATCTCAAGTATCCGATTTGATTACTTCCRGAGAAAAATTAAAACCAAGAGGTGAAGATTACCCATACGTKATAGTGAATGAGACTGGTTTAGATTTGGAAGTTTGGAATGATGCAAAWGAATYCGAAACCAATGGAGTTCCAATTGTTTTACGTGCTGGTAAAGCTTTAGATGAAAGTAAAGTTGAAATTAGAATTCAATTTAAACCAGTCGCCAAGGGGATGTTTAAAGAAATTCAAAGAAATGAATTAGTTATTAGAGTACAACCAAATGAAGCCATTTATTTAAAAATTAATTCCAAAATCCCTGGAATTTCTACTGAAACTTCATTAACTGATTTAGATTTAACTTATGCTACTCGTTATTCTAAAGACTTTTGGATTCCTGAAGCTTATGAAGCATTAATTAGAGATTGCTATTTAGGTAATCATTCTAATTTTGTTAGAGATGATGAATTGGATGTTTCTTGGAAATTATTTACTCCATTATTGAAT

>W43

AATTATTGAARTGGAATGATATTCCATTGGCTCCACCAGACAARATTTTGGGTATTTCTGAAGCTTATAACAATGATTCTAACCCTCAAAAAGTCAATTTGGGGGTTGGTGCTTATAGAGATAATTCTGGTAAACCAATTATTTTCCCATCAGTTAAAAAAGCTGAAGAAATTTTATTGGGTAAAGAAACTGAAAAGGAATATACTGCCATTGTTGGTTCCAAAAATTTCCAATCAATTGTGAAAAATTTCATTTTCAACAATTCTAATAAAGATGCCAATGGTAAACAATTAATTGATGATGGTAGAATTGTTACTGCTCAAACCATCTCTGGTACTGGATCACTTAAACCTCTTATGGTGAAATTTTTGCTAAACATAGAGAACCAAATTTGGAAATTATTCGTGAGGTTGTTGATTCCAAACATATTGTTTTTGATGTGTTGGCACAATTCTTAATCAATCCAGACCCATGGGTTGCCATTGCTGCCGCTGAAGTTTATGTCAGACGTTCATACCGTGCTTATGATTTGGGTACAATTGAATATCATGTTAATGACAGACTTCCTATTGTTGAATGGAAATTCAAGTTGGCTAATATGGGAGCCGCTGGTGTAAACGATGCTCAACAGGCTGCTGCTGCYGGTGGCGATGATTCGACATCTATGAAACATGCAGCTTCTGTGTCTGATTTGACCTTTGTTGTTGATTATTCATRAAAGCGAAATCCCAGGATACACTCTCCCCGATAATCCAAAGTTCACCCTTGGTAATTTGTTTGTAATAATTGGAGTCTTGTTGGTTTGTATTTTAGCTGTTGTCTCTCTTTTGAGAAATATTAGTGAGTCGGCCTTGTTCAAGAAGAATGGGTATGAACCGTTGGATTCGGATCCTAGTGTCATGAACCTAAACTTCGAGCCTACAACATTGTCCTTTGAAGATATTAAATATGAGGTTACTGGTGGTCGACAAATTTTAAATGGAGTCTTTGGGTTTGTAAAACCAAGAGAATGTTTGGCTATAATGGGAGGTTCAGGTGCTGGTAAAACTACATTGTTGGATTTTAAACCTTTGGACCAATTGGCTAARACTTTGRCYACTGTTCCTGAATTGAATGAAATTATTGGKCAAGAWTTAGTTGACGAATTTRTCAGTGGTATTAAACTACCAGCAGAAGTTGGAAGTCAAGATGATGTTAACAATAGAAAATTGTTGCAAAAAGTGTTTGGTAAATTAATGAACACTGATGATGACGTTATAAAACAACAAACAGCTAAATTACTTGAAAGAACAGACAGAGAACCTCAAGTGTTCAAGGATATTGATTCTAGATTACCGGAGTTAATACAAAGATTAAACAAACAATTTCCTAATGACATCGGATTATTTTGTGGATGTCTCTTATTGAACCACGTTGATTGAAAAAATTATCAATTTCCGTGTTGGATAAACAGAGATTGACTGAAAAATTCAATAAATTGGATAAATCCATCAAAGATAATTTGAAGGCTAAACAAAAAGAAGAAACCAAAAAAACTTTAGATGTGGTTAATAATTGGTTGAATGATAAAGAAAATGCTTCATCATTTTTGGTTGCTCACGTTCCAATTACTGCTAATGCCAAGGCAATCACTGAAGCCATTAATTTGATTAAAAAGCAAGATAAAACCAAATCAATTTATTTATTGACTGGTGAAACCGATAAAGTTGCTCATGGATGTTATGTTAGTGATGAAGCCATTGCCAAGGGTATTAATGCGAAAATCAAACCTTTTGAAGCACGAGCAATTAACTGGTCCACGGATCTTAATGCTGAGGTACATATTGAGCATTATATAAATATATTCAATTATGCACGATCATCTTGGGAGCCATTGGTTGAAAGTTGGCCAATAGCAGTTTACATGTCAAAATCCCGACACCCAAAGCCTCAATTATTAGTAGAGGTGATTTCTAGACAGGTAGCYCAAGTGACGCTTACATCCAAAGCAGTAGCATTGCTATCTCAAGTATCCGATTTGATTACTTCCRGAGAAAAATTAAAACCAAGAGGTGAAGATTACCCATACGTKATAGTGAATGAGACTGGTTTAGATTTGGAAGTTTGGAATGATGCAAAWGAATYCGAAACCAATGGAGTTCCAATTGTTTTACGTGCTGGTAAAGCTTTAGATGAAAGTAAAGTTGAAATTAGAATTCAATTTAAACCAGTCGCCAAGGGGATGTTTAAAGAAATTCAAAGAAATGAATTAGTTATTAGAGTACAACCAAATGAAGCCATTTATTTAAAAATTAATTCCAAAATCCCTGGAATTTCTACTGAAACTTCATTAACTGATTTAGATTTAACTTATGCTACTCGTTATTCTAAAGAYTTTTGGATTCCTGAAGCTTATGAAGCATTAATTAGAGATTGYTATTTAGGTAATCATTCTAATTTTGTTAGAGATGATGAATTGGATGTTTCTTGGAAATTATTTACTCCATTATTGAAT

>YSsU123:IND:45:3.7

AATTATTGAAATGGAATGATATTCCATTGGCYCCACCAGACAAGATTTTGGGTATTTCTGAAGCTTATAACAATGATTCTAACCCTCAAAAAATCAATTTGGGGGTTGGTGCTTATAGAGATAATTCTGGTAAACCAATTATTTTCCCATCAGTTAAAAAAGCTGAAGAAATTTTATTGGGTAAAGAAACTGAAAAGGAATATACTGCCATTGTTGGTTCCAAAAATTTCCAATCAATTGTGAAAAATTTCATTTTCAACAATTCTAATAAAGATGCCAATGGTAAACAATTAATTGATGATGGTAGAATTGTTACTGCTCAAACCATCTCTGGTACTGGATCACTTAAACCTCTTATGGTGAAATTTTTGCTAAACATAGAGAACCAAATTTGGAAATTATTCGTGAGGTTGTTGATTCCAAACATATTGTTTTTGATGTGTTGGCACAATTCTTAATCAATCCAGACCCATGGGTTGCCATTGCTGCCGCTGAAGTTTATGTCAGACGTTCATACCGTGCTTATGATTTGGGTAAAATTGAATATCATGTTAATGACAGACTTCCTATTGTTGAATGGAAATTCAAGTTGGCTAATATGGGAGCTGCTGGTGTAAACGATGCTCAACAGGCTGCTGCTGCCGGTGGCGATGATTCGACATCTATGAAACATGCAGCTTCTGTGTCTGATTTGACCTTTGTTGTTGATTATTCATAAAAGCGAAATCCCAGGATACACTCTCCCCGATAATCCAAAGTTCACCCTTGGTAATTTGTTTGTAATAATTGGAGTCTTGTTGGTTTGTATTTTAGCTGTTGTCTCTCTTTTGAGAAATATTAGTGAGTCGGCCTTGTTCAAGAAGAATGGGTATGAACCGTTGGATTCGGATCCTAGTGTCATGAACCTAAACTTCGAGCCTACAACATTGTCCTTTGAAGATATTAAATATGAGGTTACTGGTGGTCGACAAATTTTAAATGGAGTCTTTGGGTTTGTAAAACCAAGAGAATGTTTGGCTATAATGGGAGGTTCAGGTGCTGGTAAAACTACATTGTTGGATTTTAAACCTTTGGACCAATTGGCTAAAACTTTGGCTACTGTTCCTGAATTGAATGAAATTATTGGTCAAGAATTAGTTGACGAATTTRTCAGTGGTATTAAACTACCAGCAGAAGTTGGAAGTCAAGATGATGTTAACAATAGAAAATTGTTGCAAAAAGTGTTTGGTAAATTAATGAACACTGATGATGACGTTATAAAACAACAAACAGCTAAATTACTTGAAAGAACAGACAGAGAACCTCAAGTGTTCAAGGATATTGATTCTAGATTACCGGAGTTAATACAAAGATTAAACAAACAATTTCCTAATGACATCGGATTATTTTGTGGATGTCTCTTATTGAACCACGTTGCTTGAAAAAATTATCAATTTCCGTGTTGGATAAACARAGATTGACTGAAAAATTCAATAAATTGGATAAATCCATTAAAGATAATTTGAAGGCTAAACAAAAAGAAGAAACCAAAAAGACTTTAGATGTGGTTAACAATTGGTTGAATGATAAAGAAAATGCTTCATCATTTTTGGTTGCTCACGTTCCAATTACTGCTAATGCCAAGGCAATCACTGAAGCCATTAATTTGATTAAAAAGCAAGATAAAACCAAATCAATTTATTTATTGACTGGTGAAACCGATAAAGTTGCTCATGGATGTTATGTTAGTGATGAAGCCATTGTCAAGGGTATTAATGCGAAAATCAAACCTTTTGAAGCACGAGCAATTAACTGGTCCACGGATCTTAATGCTGAGGTACATATTGAGCATTATATAAATATATTCAATTATGCACGATCATCTTGGGAGCCATTGGTTGAAAGTTGGCCAATAGCAGTTTACATGTCAAAATCCCGACACCCAAAGCCTCAATTATTAGTAGAGGTGATTTCTAGACAGGTAGCYCAAGTGACGCTTACATCCAAAGCAGTAGCATTGCTATCTCAAGTATCCGATTTGATTACTTCCRGAGAAAAATTAAAACCAAGAGGTGAAGATTACCCATACGTKATAGTGAATGAGACTGGTTTAGATTTGGAAGTTTGGAATGATGCAAAWGAATCCGAAACCAATGGAGTTCCAATTGTTTTACGTGCTGGTAAAGCTTTAGATGAAAGTAAAGTTGAAATTAGAATTCAATTTAAACCAGTCGCCAAGGGGATGTTTAAAGAAATTCAAAGAAATGAATTAGTTATTAGAGTACAACCAAATGAAGCCATTTATTTAAAAATTAATTCCAAAATCCCTGGRATTTCTACTGAAACTTCATTAACTGATTTAGATTTAACTTATGCTACTCGTTATTCTAAAGAYTTTTGGATTCCTGAAGCTTATGAAGCATTAATTAGAGATTGTTATTTAGGTAATCATTCTAATTTTGTTAGAGATGATGAATTGGATGTTTCTTGGAAATTATTTACTCCATTATTGAAT

>YsU123

AATTATTGAAATGGAATGATATTCCATTGGCYCCACCAGACAAGATTTTGGGTATTTCTGAAGCTTATAACAATGATTCTAACCCTCAAAAAATCAATTTGGGGGTTGGTGCTTATAGAGATAATTCTGGTAAACCAATTATTTTCCCATCAGTTAAAAAAGCTGAAGAAATTTTATTGGGTAAAGAAACTGAAAAGGAATATACTGCCATTGTTGGTTCCAAAAATTTCCAATCAATTGTGAAAAATTTCATTTTCAACAATTCTAATAAAGATGCCAATGGTAAACAATTAATTGATGATGGTAGAATTGTTACTGCTCAAACCATCTCTGGTACTGGATCACTTAAACCTCTTATGGTGAAATTTTTGCTAAACATAGAGAACCAAATTTGGAAATTATTCGTGAGGTTGTTGATTCCAAACATATTGTTTTTGATGTGTTGGCACAATTCTTAATCAATCCAGACCCATGGGTTGCCATTGCTGCCGCTGAAGTTTATGTCAGACGTTCATACCGTGCTTATGATTTGGGTAAAATTGAATATCATGTTAATGACAGACTTCCTATTGTTGAATGGAAATTCAAGTTGGCTAATATGGGAGCTGCTGGTGTAAACGATGCTCAACAGGCTGCTGCTGCCGGTGGCGATGATTCGACATCTATGAAACATGCAGCTTCTGTGTCTGATTTGACCTTTGTTGTTGATTATTCATAAAAGCGAAATCCCAGGATACACTCTCCCCGATAATCCAAAGTTCACCCTTGGTAATTTGTTTGTAATAATTGGAGTCTTGTTGGTTTGTATTTTAGCTGTTGTCTCTCTTTTGAGAAATATTAGTGAGTCGGCCTTGTTCAAGAAGAATGGGTATGAACCGTTGGATTCGGATCCTAGTGTCATGAACCTAAACTTCGAGCCTACAACATTGTCCTTTGAAGATATTAAATATGAGGTTACTGGTGGTCGACAAATTTTAAATGGAGTCTTTGGGTTTGTAAAACCAAGAGAATGTTTGGCTATAATGGGAGGTTCAGGTGCTGGTAAAACTACATTGTTGGATTTTAAACCTTTGGACCAATTGGCTAAAACTTTGGCTACTGTTCCTGAATTGAATGAAATTATTGGTCAAGAATTAGTTGACGAATTTRTCAGTGGTATTAAACTACCAGCAGAAGTTGGAAGTCAAGATGATGTTAACAATAGAAAATTGTTGCAAAAAGTGTTTGGTAAATTAATGAACACTGATGATGACGTTATAAAACAACAAACAGCTAAATTACTTGAAAGAACAGACAGAGAACCTCAAGTGTTCAAGGATATTGATTCTAGATTACCGGAGTTAATACAAAGATTAAACAAACAATTTCCTAATGACATCGGATTATTTTGTGGATGTCTCTTATTGAACCACGTTGCTTGAAAAAATTATCAATTTCCGTGTTGGATAAACARAGATTGACTGAAAAATTCAATAAATTGGATAAATCCATTAAAGATAATTTGAAGGCTAAACAAAAAGAAGAAACCAAAAAGACTTTAGATGTGGTTAACAATTGGTTGAATGATAAAGAAAATGCTTCATCATTTTTGGTTGCTCACGTTCCAATTACTGCTAATGCCAAGGCAATCACTGAAGCCATTAATTTGATTAAAAAGCAAGATAAAACCAAATCAATTTATTTATTGACTGGTGAAACCGATAAAGTTGCTCATGGATGTTATGTTAGTGATGAAGCCATTGTCAAGGGTATTAATGCGAAAATCAAACCTTTTGAAGCACGAGCAATTAACTGGTCCACGGATCTTAATGCTGAGGTACATATTGAGCATTATATAAATATATTCAATTATGCACGATCATCTTGGGAGCCATTGGTTGAAAGTTGGCCAATAGCAGTTTACATGTCAAAATCCCGACACCCAAAGCCTCAATTATTAGTAGAGGTGATTTCTAGACAGGTAGCYCAAGTGACGCTTACATCCAAAGCAGTAGCATTGCTATCTCAAGTATCCGATTTGATTACTTCCRGAGAAAAATTAAAACCAAGAGGTGAAGATTACCCATACGTKATAGTGAATGAGACTGGTTTAGATTTGGAAGTTTGGAATGATGCAAAWGAATCCGAAACCAATGGAGTTCCAATTGTTTTACGTGCTGGTAAAGCTTTAGATGAAAGTAAAGTTGAAATTAGAATTCAATTTAAACCAGTCGCCAAGGGGATGTTTAAAGAAATTCAAAGAAATGAATTAGTTATTAGAGTACAACCAAATGAAGCCATTTATTTAAAAATTAATTCCAAAATCCCTGGRATTTCTACTGAAACTTCATTAACTGATTTAGATTTAACTTATGCTACTCGTTATTCTAAAGAYTTTTGGATTCCTGAAGCTTATGAAGCATTAATTAGAGATTGTTATTTAGGTAATCATTCTAATTTTGTTAGAGATGATGAATTGGATGTTTCTTGGAAATTATTTACTCCATTATTGAAT

>YsU123:IND:8:4.6

AATTATTGAAATGGAATGATATTCCATTGGCTCCACCAGACAAGATTTTGGGTATTTCTGAAGCTTATAACAATGATTCTAACCCTCAAAAAATCAATTTGGGGGTTGGTGCTTATAGAGATAATTCTGGTAAACCAATTATTTTCCCATCAGTTAAAAAAGCTGAAGAAATTTTATTGGGTAAAGAAACTGAAAAGGAATATACTGCCATTGTTGGTTCCAAAAATTTCCAATCAATTGTGAAAAATTTCATTTTCAACAATTCTAATAAAGATGCCAATGGTAAACAATTAATTGATGATGGTAGAATTGTTACTGCTCAAACCATCTCTGGTACTGGATCACTTAAACCTCTTATGGTGAAATTTTTGCTAAACATAGAGAACCAAATTTGGAAATTATTCGTGAGGTTGTTGATTCCAAACATATTGTTTTTGATGTGTTGGCACAATTCTTAATCAATCCAGACCCATGGGTTGCCATTGCTGCCGCTGAAGTTTATGTCAGACGTTCATACCGTGCTTATGATTTGGGTAAAATTGAATATCATGTTAATGACAGACTTCCTATTGTTGAATGGAAATTCAAGTTGGCTAATATGGGAGCTGCTGGTGTAAACGATGCTCAACAGGCTGCTGCTGCCGGTGGCGATGATTCGACATCTATGAAACATGCAGCTTCTGTGTCTGATTTGACCTTTGTTGTTGATTATTCATAAAAGCGAAATCCCAGGATACACTCTCCCCGATAATCCAAAGTTCACCCTTGGTAATTTGTTTGTAATAATTGGAGTCTTGTTGGTTTGTATTTTAGCTGTTGTCTCTCTTTTGAGAAATATTAGTGAGTCGGCCTTGTTCAAGAAGAATGGGTATGAACCGTTGGATTCGGATCCTAGTGTCATGAACCTAAACTTCGAGCCTACAACATTGTCCTTTGAAGATATTAAATATGAGGTTACTGGTGGTCGACAAATTTTAAATGGAGTCTTTGGGTTTGTAAAACCAAGAGAATGTTTGGCTATAATGGGAGGTTCAGGTGCTGGTAAAACTACATTGTTGGATTTTAAACCTTTGGACCAATTGGCTAAAACTTTGGCTACTGTTCCTGAATTGAATGAAATTATTGGTCAAGAATTAGTTGACGAATTTRTCAGTGGTATTAAACTACCAGCAGAAGTTGGAAGTCAAGATGATGTTAACAATAGAAAATTGTTGCAAAAAGTGTTTGGTAAATTAATGAACACTGATGATGACGTTATAAAACAACAAACAGCTAAATTACTTGAAAGAACAGACAGAGAACCTCAAGTGTTCAAGGATATTGATTCTAGATTACCGGAGTTAATACAAAGATTAAACAAACAATTTCCTAATGACATCGGATTATTTTGTGGATGTCTCTTATTGAACCACGTTGCTTGAAAAAATTATCAATTTCCGTGTTGGATAAACAGAGATTGACTGAAAAATTCAATAAATTGGATAAATCCATTAAAGATAATTTGAAGGCTAAACAAAAAGAAGAAACCAAAAAGACTTTAGATGTGGTTAACAATTGGTTGAATGATAAAGAAAATGCTTCATCATTTTTGGTTGCTCACGTTCCAATTACTGCTAATGCCAAGGCAATCACTGAAGCCATTAATTTGATTAAAAAGCAAGATAAAACCAAATCAATTTATTTATTGACTGGTGAAACCGATAAAGTTGCTCATGGATGTTATGTTAGTGATGAAGCCATTGTCAAGGGTATTAATGCGAAAATCAAACCTTTTGAAGCACGAGCAATTAACTGGTCCACGGATCTTAATGCTGAGGTACATATTGAGCATTATATAAATATATTCAATTATGCACGATCATCTTGGGAGCCATTGGTTGAAAGTTGGCCAATAGCAGTTTACATGTCAAAATCCCGACACCCAAAGCCTCAATTATTAGTAGAGGTGATTTCTAGACAGGTAGCYCAAGTGACGCTTACATCCAAAGCAGTAGCATTGCTATCTCAAGTATCCGATTTGATTACTTCCRGAGAAAAATTAAAACCAAGAGGTGAAGATTACCCATACGTKATAGTGAATGAGACTGGTTTAGATTTGGAAGTTTGGAATGATGCAAAWGAATCCGAAACCAATGGAGTTCCAATTGTTTTACGTGCTGGTAAAGCTTTAGATGAAAGTAAAGTTGAAATTAGAATTCAATTTAAACCAGTCGCCAAGGGGATGTTTAAAGAAATTCAAAGAAATGAATTAGTTATTAGAGTACAACCAAATGAAGCCATTTATTTAAAAATTAATTCCAAAATCCCTGGAATTTCTACTGAAACTTCATTAACTGATTTAGATTTAACTTATGCTACTCGTTATTCTAAAGACTTTTGGATTCCTGAAGCTTATGAAGCATTAATTAGAGATTGYTATTTAGGTAATCATTCTAATTTTGTTAGAGATGATGAATTGGATGTTTCTTGGAAATTATTTACTCCATTATTGAAT

>YsU123:IND:10:6.8
[truncated: 10,032 more chars]
