# Supplementary material for: Multi-Locus Next-Generation Sequence Typing of DNA Extracted From Pooled Colonies Detects Multiple Unrelated Candida albicans Strains in a Significant Proportion of Patient Samples
Source: Front Microbiol. 2018 Jun 5;9:1179. doi: 10.3389/fmicb.2018.01179 (PMC5996278; doi:10.3389/fmicb.2018.01179)
Supplement: Supplementary file 8 [file Data_Sheet_3.docx]

**Supplementary Data Sheet S3. Approximating the genotypes of three strains in a sample**

When three strains are present in a sample (sc, B1, B2), DALMATIONS will not be able to infer a strain B frequency and genotype with a low UDI. However, one output of DALMATIONS is a table showing discrepancies between actual base calls and base calls expected for strain B at each informative site at each tested frequency. If strain B in reality represents two strains (B1 and B2) then there should be one frequency (F1) at which for a number of positions, those at which only B1 differs from the sc strain, expectations and observed base calls are a close match. At a second frequency (F2), expectations and observed base calls should be a close match for B2-specific polymorhisms and at a third frequency (F3) there should be good matches at postions at which B1 and B2 are identical, but both differ from the sc strain. F1 is the frequency of strain B1, F2 the frequency of strain B2 and F3 the frequency of strain B1 plus strain B2. Therefore 1 - F3 equals the frequency of the sc strain. Knowing F3 and the genotype of the sc strain, sc strain base calls can be removed from the data and frequencies F1 and F2 increased proportionally so that F1plus F2 equals 100 %. Using the tri9.xsl excel template (a part of the DALMATIONS download) with these adjusted frequencies, the genotypes of strains B1 and B2 most consistent with the data can then be inferred for each position.

Since 1-F3 equals the frequency of the sc strain and the single colony picked is most likely from the most common strain, F3 is most likely the smallest of the three strain frequencies that explain data well for some polymorphisms. It may however be necessary in some cases to carry out the procedure above three times, designating each of three frequencies as F3. The correct choice should be the only one that produces B1 and B2 genotypes that match the data well.
