# Supplementary figures and images for: Multi-Locus Next-Generation Sequence Typing of DNA Extracted From Pooled Colonies Detects Multiple Unrelated Candida albicans Strains in a Significant Proportion of Patient Samples
Source: Front Microbiol. 2018 Jun 5;9:1179. doi: 10.3389/fmicb.2018.01179 (PMC5996278; doi:10.3389/fmicb.2018.01179)

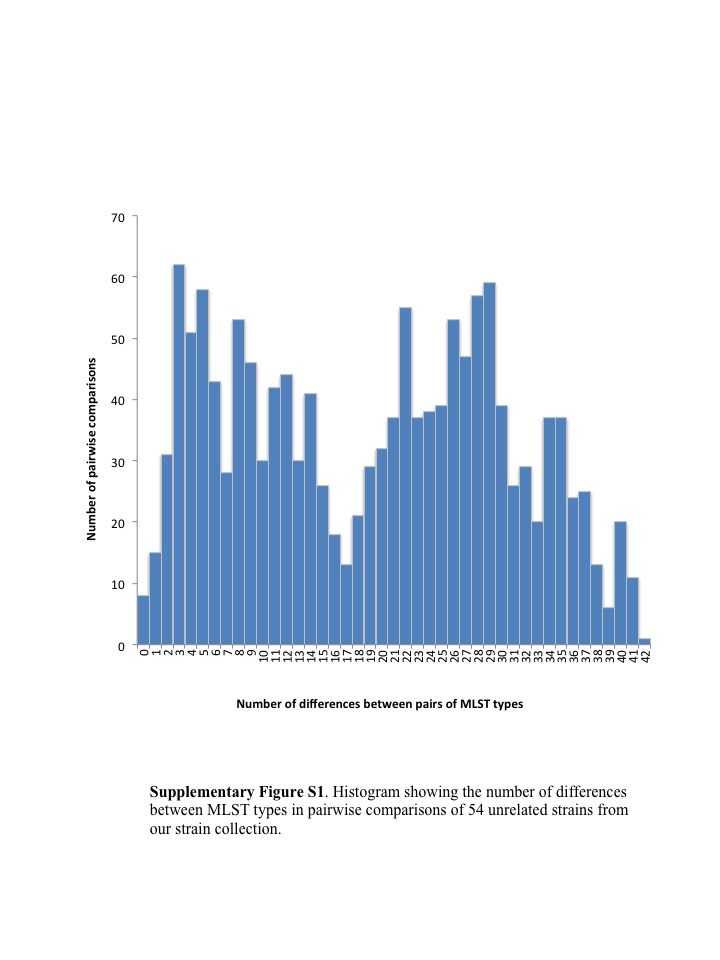

Supplement: Supplementary file 9 [file Image_1.JPEG]
